# Catalytic Markovnikov hydrophosphorylation of unactivated olefins via a radical-polar crossover rearrangement

**DOI:** 10.1038/s41467-026-72870-2

**Published:** 2026-05-16

**Authors:** Yi-Fan Li, Hong-Chen Wang, Bing Han

**Affiliations:** State Key Laboratory of Natural Product Chemistry, College of Chemistry and Chemical Engineering, Lanzhou University, Lanzhou, People’s Republic of China

**Keywords:** Synthetic chemistry methodology, Reaction mechanisms, Homogeneous catalysis

## Abstract

The phosphorylation of alkenes offers a diverse handle for obtaining organophosphoryls, but its regioselectivity still faces challenges, especially the radical Markovnikov hydrophosphorylation of alkenes. Herein, we develop a radical Markovnikov hydrophosphorylation of unactivated alkenes by a sequential reaction of (bis)homoallylic alcohols, Ph_2_PCl, and water under visible light-driven triple catalysis. The protocol initiates from a Co-H species-mediated metal-hydride atom transfer, followed by a water-mediated radical-polar crossover phosphinite rearrangement. This reaction features mild reaction conditions, excellent regio-/stereoselectivity, and good natural products compatibility. The successful radical deuterophosphonation of olefins by using D_2_O to replace H_2_O further demonstrates the synthetic value of the protocol. This protocol not only broadens the mode of phosphinite rearrangement, but also provides a successful example for the radical activation of water and its three-atom splitting and utilization in organic conversion.

## Abstract (web summary)

Organophosphoryl compounds are important motifs, however regioselective phosphorylation of alkenes remains challenging. Here, the authors report a visible light driven triple catalytic strategy enabling radical Markovnikov hydrophosphorylation of unactivated alkenes via sequential reaction of (bis)homoallylic alcohols, Ph₂PCl, and water.

## Introduction

**Fig. 1:**
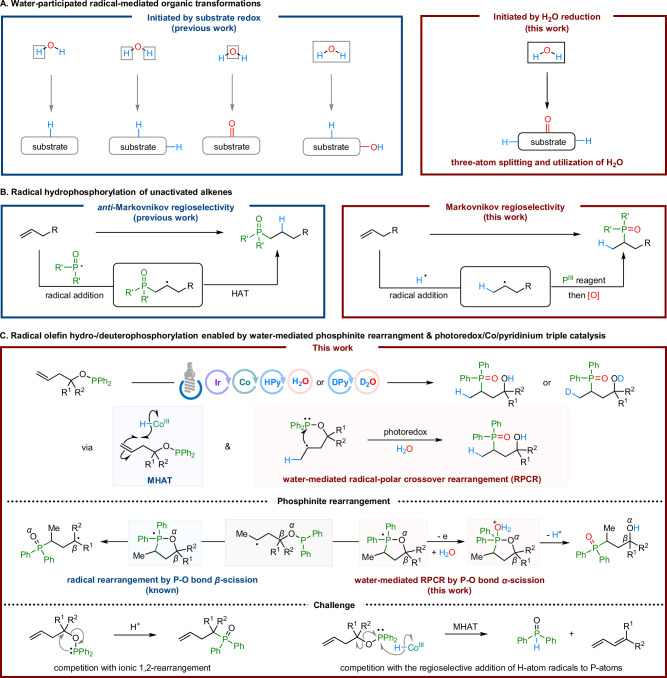
Radical Markovnikov hydro- and deuterophosphorylation of alkenes. **A** Water-participated radical-mediated organic transformation. **B** Radical hydrophosphinylation of unactivated alkenes. **C** Radical olefin hydro-/deuterophosphinylation enabled by water-mediated phosphinite rearrangement & photoredox/Co/pyridinium triple catalysis.

Water, as one of the most abundant, clean, and renewable substances, is an ideal source of both hydrogen and oxygen, and plays a crucial role in hydrogenation and oxidation processes of biosynthesis in nature^1,2^. The utilization of water as a green source of hydrogen and oxygen in organic conversion also provides a paradigm for the development of sustainable chemistry^3–16^. However, most reactions involve ionic mechanisms catalyzed by Lewis or Brønsted acids^3–7^. Therefore, the development of alternative types of water-mediated organic reactions is of great significance. Recently, with the emergence of controllable redox strategies mediated by visible light catalysis and electrosynthesis, radical-mediated organic transformations involving water have developed rapidly^8–16^. However, these conversions are mainly triggered by the redox reaction of organic substrates, while those initiated by the redox activation of water are still very limited. In addition, these reactions usually can only partially utilize oxygen or hydrogen atoms in water molecules, with relatively low utilization rates. To our knowledge, the complete incorporation of all three atoms (H, H, O) of a water molecule into an organic compound by the radical-mediated water activation and splitting has not yet been realized (Fig. 1A).

Organophosphoryls play an indispensable role in a wide range of fields such as materials, biochemistry, and catalysis due to their unique physical and chemical properties^17–20^. The installation of phosphorus-containing groups on alkenes through radical pathways provides an effective approach to obtaining such scaffolds^21–23^, but controlling the regioselectivity of this process remains a fundamental challenge, especially in the radical hydrophosphorylation of alkenes. The anti-Markovnikov selectivity has been successfully achieved by adding an easily generated electrophilic phosphoryl radical at the terminal position of alkenes and through the subsequent hydrogen-atom transfer (HAT) process (Fig. 1B)^24^. However, the Markovnikov selectivity caused by adding a hydrogen radical at the terminal position of the alkenes, followed by the radical C-P bond construction, has not yet been achieved. One important reason is that it is still not easy to install a phosphoryl group onto the inner side of olefins by a radical Arbuzov reaction^25–29^, due to the competitive addition of the attacking functionality radical onto the alkene and the phosphite, as well as the inert addition ability of the formed secondary/tertiary carbon radical onto the phosphite. In a few successful cases, special pre-synthetic P^III^ reagents are still needed as a secondary alkyl radical trap^30^, followed by further converting P^III^ to phosphoryl with additional oxygenating reagents. Therefore, it will be of great significance to achieve the currently unattainable radical Markovnikov hydrophosphonylation of olefins using commercially available simple trivalent phosphorus reagents. Furthermore, considering that the radical Markovnikov hydrophosphorylation of olefins involves both the hydrogenation of olefins and the oxygenation of trivalent phosphorus, the function of water as an ideal source of hydrogen and oxygen perfectly meets this requirement and also provides an opportunity to achieve this goal.

Recently, the Co^III^-H species-mediated metal-hydride atom transfer (MHAT) reaction provides a powerful tool for the generation of hydrogen atoms and the realization of the Markovnikov hydrofunctionalization reaction of alkenes^31–48^. Although most reactions use additional hydrogen gas, boranes and silanes etc. as the terminal hydrogen sources^39–43^, reactions that utilize protons as hydrogen sources have also received increasing attention^44–48^. Among them, Ohmiya et al. realized a Markovnikov olefin hydroetherization using alcohols as the substrate as well as the hydrogen source under a triple catalysis of photoredox/cobalt/Brønsted acid catalysis^48^. Inspired by this elegant work, we assumed that this catalytic system may also be feasible in the activation of water molecules to realize the olefin hydrophosphonylation reaction. Herein, we develop a radical Markovnikov hydrophosphorylation of alkenes by a sequential reaction of (bis)homoallylic alcohols, Ph_2_PCl, and water, employing this photoredox/cobalt/pyridinium triple-catalytic system (Fig. 1C). This protocol employs commercially available Ph_2_PCl as the phosphorylating reagent and water as both green hydrogen and oxygen sources. The reaction initiates from MHAT between a Co^III^-H species derived from the proton reduction of water and the in situ formed unsaturated phosphinite, followed by a water-mediated radical-polar crossover rearrangement (RPCR)^49–51^ of phosphinite. Unlike the known radical-promoted rearrangement^52–59^ of phosphinite, which is achieved through the *β-*scission of the P-O bond^60–63^, the present water-mediated RPCR experiences the *α-*scission of the P-O bond. We demonstrate the wide substrate scope, excellent regio- and diastereoselectivity, as well as good functional group tolerance and complex natural product compatibility of this protocol by altering the structures of (bis)homoallylic alcohols and Ar_2_PCl. The synthetic value of the protocol is also showcased by the successful olefin deuterophosphorylation with excellent deuteration regioselectivity and ratio by using D_2_O to replace H_2_O. This reaction not only develops a water-mediated RPCR of phosphinite but also provides a strategy for the efficient utilization of water molecules in organic synthesis.

## Results and discussion

The study was commenced with a two-step sequential reaction, that is, the condensation of homoallylic alcohol **1a** and Ph_2_PCl under Et_3_N conditions, followed by the addition of H_2_O, photocatalyst, cobalt catalyst, and pyridinium salt catalyst and the irradiation with blue LEDs (455 nm, 6 W) under Ar atmosphere at room temperature (Table 1). To our delight, when Ir(ppy)_3_, **Co-1**, and **HPy-1** were used as the combined catalysts, the desired olefin hydrophosphorylation smoothly took place and produced the corresponding product **2a** in 75% yield (Table 1, entry 1). Photocatalyst, cobalt catalyst, and pyridinium salt catalyst were essential for an efficient reaction, and the reaction cannot proceed smoothly without any of them (Table 1, entry 2). Other commercially available cobalt catalysts, such as **Co-2** and **Co-3**, were also effective but gave lower yields of **2a** (Table 1, entries 3 and 4). To further enhance the reaction yield, we also tested photocatalysts such as 10-phenyl-10*H*-phenothiazine (PTH) and 2,4,5,6-tetra(9*H*-carbazol-9-yl) isophthalonitrile (4CzIPN), but the **2a** yield obtained was not satisfactory (Table 1, entries 5 and 6). The pyridinium salts screening indicated that **HPy-1** with ClO_4_^-^ as the counterion had the best effect, while the others **HPy-2**-**4** with OTf^-^, PF_6_^-^, and Cl^-^ as the different counterions, respectively, only gave moderate yields (Table 1, entries 7-9). Notably, the reaction by directly utilizing the parent Brønsted acids as the catalyst cannot proceed (Table 1, entry 10). DMA was proven to be the best one in the solvent screening (Table 1, entry 11). In addition, light irradiation was essential for an efficient reaction since no reaction occurred in the dark (Table 1, entry 12). Significantly, water was an indispensable reactant, and the reaction can hardly proceed without it (Table 1, entry 13).

**Table 1:** Optimization of reaction conditions


Entry	Variation of conditions^a^	Yield (%)^b^
1	none	75
2	w/o **Ir(ppy)**_**3**_ *or* **Co-1** *or* **HPy-1**	0
3	**Co-2** instead of **Co-1**	59
4	**Co-3** instead of **Co-1**	50
5	**PTH** instead of **Ir(ppy)**_**3**_	29
6	**4CzIPN** instead of **Ir(ppy)**_**3**_	45
7	**HPy-2** instead of **HPy-1**	48
8	**HPy-3** instead of **HPy-1**	52
9	**HPy-4** instead of **HPy-1**	45
10	parent Brönsted acids HClO_4_ / HOTf / HPF_6_ / HCl instead of **HPy**	0
11	MeCN *or* CH_2_Cl_2_ *or* THF instead of DMA	<47
12	in the dark	0
13	w/o H_2_O	<5

^a^Standard reaction conditions: **step 1**: alcohol **1a** (0.20 mmol), DMAP (20 mol%), Et_3_N (1.2 equiv.), and R_2_PCl (1.1 equiv.) in dry THF (1 mL) were stirred at 0  °C to room temperature under argon atmosphere for 1 h; **step 2**: phosphinite **1a’,** **Co-1** (5.0 mol%), **HPy-1** (20 mol%), Ir(ppy)_3_ (1 mol%), DMA (2 mL) and H_2_O (1.5 equiv.) were stirred under argon atmosphere with irradiation of blue LEDs (455 nm, 6 W) at room temperature for 14 h.

^b^Isolated yield. DMAP = 4-dimethylaminopyridine.

**Fig. 2:**
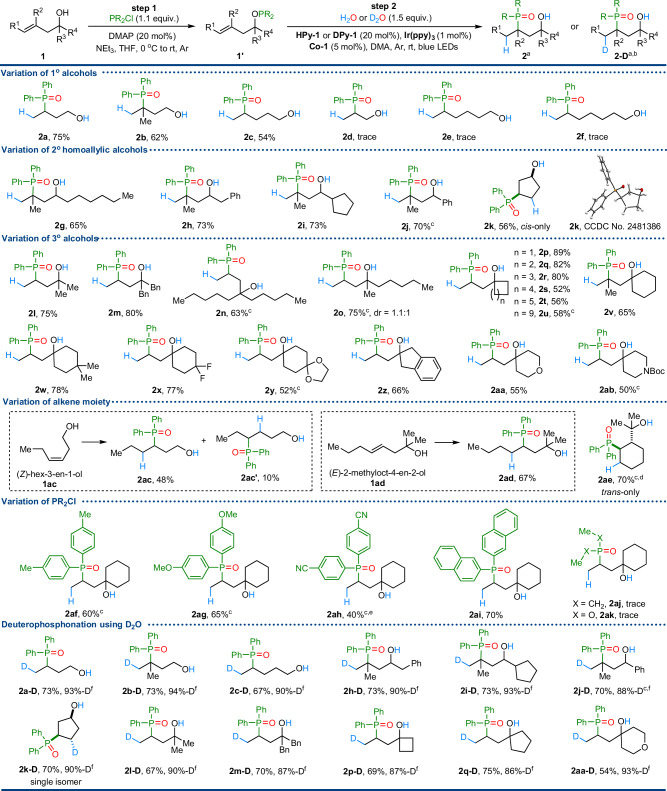
Reaction scope. ^a^Standard reaction conditions **A**: **step 1**: alcohol (0.20 mmol), DMAP (20 mol%), Et_3_N (1.2 equiv.), and R_2_PCl (1.1 equiv.) in dry THF (1 mL) were stirred at 0 °C to room temperature under argon for 1-5 h; **step 2**: phosphinite **1’,** **Co-1** (5.0 mol%), **HPy-1** (20 mol%), Ir(ppy)_3_ (1 mol%), DMA (2 mL) and H_2_O (1.5 equiv.) were stirred under argon atmosphere with irradiation of blue LEDs (455 nm, 6 W) at room temperature for 14 h. Isolated yield. Diastereomer ratio was determined by ^1^H NMR. ^b^Standard reaction conditions **B**: **DPy-1** and D_2_O were used instead of **HPy-1** and H_2_O. ^c^The product yield was calculated based on phosphinite **1’**. ^d^The *trans*-configuration was determined by NOE experiments. ^e^**HPy-1** (40 mol%) was used. ^f^Deuteration ratio was determined by ^1^H NMR.

With the optimal conditions established (Table 1, entry 1), we explored the protocol scope by varying olefinic alcohols and R_2_PCl (Fig. 2). Firstly, the structure of olefinic alcohols was explored. Primary homoallylic alcohol and its 4-methyl substitute reacted very well in the protocol and provided the corresponding **2a** and **2b** in 75% and 62% yields, respectively. Bishomoallylic alcohol was also a good candidate and successfully transformed to the corresponding product **2c** in 54% yield. Unfortunately, other homologs, such as allyl alcohol, hex-5-ene-1-alcohol, and hept-6-ene-1-alcohol, which have shorter or longer carbon chains between olefin and hydroxyl moieties, only produced trace amounts of the corresponding products **2d**-**2f**. These results indicate that the formation of five-membered or six-membered phosphorous heterocycles is the decisive factor for an effective reaction during the phosphinite rearrangement process. Secondary homoallylic alcohols bearing different alkyl substitutes such as *n*-hexyl, benzyl, cyclopentyl were all suitable for the conversion, giving rise to the desired hydrophosphorylation products **2g**-**2i** in good yields. In addition, the phenyl-substituted counterpart was also well transformed in the reaction, as demonstrated in the case of **2j**. Notably, when cyclopent-3-en-1-ol, a typical cyclic secondary homoallylic alcohol, participated in the reaction, the diastereospecific product **2k** was generated in the form of a sole *cis*-isomer with a yield of 56%. The cis-configuration of **2k** was confirmed by X-ray single crystal diffraction analysis. The formation of cis-configuration also reveals that the phosphorous migration involves the cleavage of the P-O(C) bond rather than the C-O bond. In addition to primary and secondary alcohols, tertiary alcohols were also good reaction partners. As demonstrated in the cases of **2l**-**2n**, symmetric chain homoallylic alcohols bearing *gem-*dimethyl, *gem-*dibenzyl and *gem-*dipentyl were also converted very well in the reaction. When an asymmetric one was involved in the protocol, the product **2o** was produced in 75% yield as a couple of diastereoisomers with a ratio of 1:1.1. In addition, cyclic tertiary alcohols with different ring sizes, including small, medium and large rings, were all suitable for the reaction, delivering the corresponding products **2p**-**2u** in good to excellent yields. Furthermore, when isobutylene incorporated cyclohexanol was involved in the reaction, the corresponding product **2 v** was also obtained in 65% yield. Significantly, *gem*-difluoro, *gem*-dimethyl and spiro ketal-substituted, as well as O, N-atom-embedded tertiary cyclohexyl alcohols were all good candidates, as demonstrated in the cases **2w**-**2ab**, showcasing good functional group tolerance with fluoro, acetal, ether, and amine. Next, we investigated the influence on the reaction by varying the structure of the olefin moiety. In addition to terminal alkenes such as α-olefins and 1,1-disubstituted olefins, acyclic and cyclic internal alkenes were also suitable for the approach. When (*Z*)-hex-3-en-1-ol participated in the reaction, the reaction showed good regioselectivity by producing **2ac** in 48% yield, accompanied by the formation of another regioisomer, **2ac’** in 10% yield. When (*E*)-2-methyloct-4-en-2-ol was involved in the reaction, it only regioselectively produced **2ad** in 67% yield, without observing another regioisomer. Apparently, the good regioselectivity can be attributed to the stereo-hindrance effect between the phosphite group and the Co-H complex. The larger the phosphinite group, the greater the steric hindrance between them, and the more difficult it is for the alkene carbon atoms closer to the phosphinite to undergo the MHAT reaction. When cycloalkenes represented by (±)-2-(cyclohex-2-en-1-yl) propan-2-ol participated in the reaction, the product **2ae** was regioselectively generated in 70% yield with the exclusive *trans-*configuration. This excellent stereoselectivity may be due to the fact that in phosphinite rearrangement involving fused rings, the *cis*-fused 5, 6-bicyclic radical intermediates are more easily formed than the trans-fused counterparts. Next, the scope of R_2_PCl was also explored. Diphenylchlorophosphines bearing electron-donating substituents such as Me and MeO groups on the *para*- position of the phenyl ring converted very well and gave the corresponding products **2af** and **2ag** in good yields, whereas the *para*-CN substituted counterpart afforded the corresponding product **2ah** in 40% yield. Obviously, this reaction is sensitive to the electronic effect of the substituents on the phenyl ring, and the electron-withdrawing group will lead to relatively low reaction efficiency. Besides, the reaction was also compatible with dinaphthylchlorophosphine and delivered the corresponding product **2ai** in 70% yield. Unfortunately, dialkyl-/dialkoxychlorophosphines represented by diethyl and diethoxy were not good partners, yielding merely trace amounts of **2aj** and **2ak**, probably due to the high oxidation potential of the corresponding phosphoranyl radical. It is noteworthy that this protocol was also applicable to the olefin deuterophosphorylation by using D_2_O and **DPy-1** to replace H_2_O and **HPy-1**, respectively. Consequently, the highly efficient and site-selective synthesis of the deuterated products was realized as demonstrated in the representative cases of **2a-c-D,** **2h-m-D,** **2p-D,** **2q-D**, and **2aa-D** in good yields with excellent deuteration ratios.

**Fig. 3:**
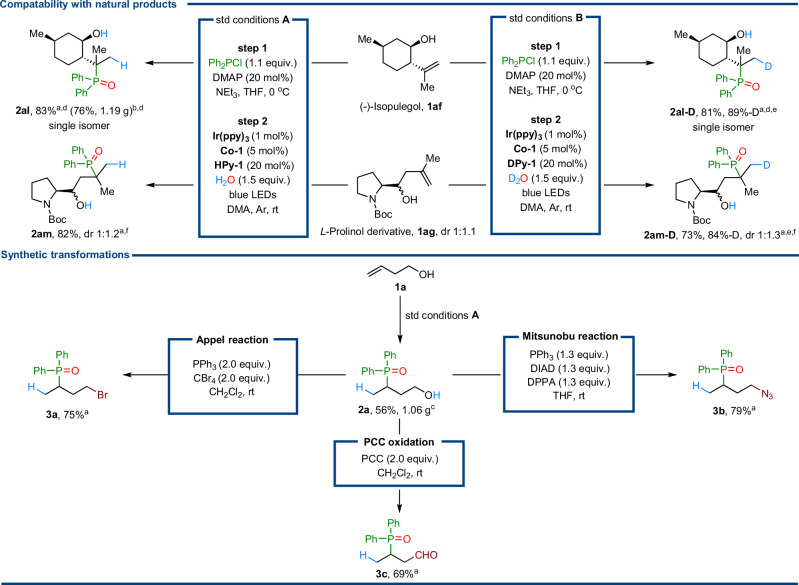
Practicality and follow-up transformations. ^a^Reaction was conducted on 0.2 mmol scale. Isolated yields. ^b^Reaction was conducted on 4.4 mmol scale. Isolated yields. ^c^Reaction was conducted on 7.0 mmol scale. Isolated yields. ^d^Configuration was determined by the coupling constant of ^1^H NMR. ^e^Deuteration ratio was determined by ^1^H NMR. ^f^Diastereomer ratio was determined by ^1^H NMR. DIAD = Diisopropyl azodicarboxylate, DPPA = diphenylphosphoryl azide, PCC = pyridinium chlorochromate.

The late-stage modification of natural products and their derivatives further proved the applicability of the approach with complex scaffolds (Fig. 3). When isopulegol, a typical natural monoterpenoid, was involved in the protocol, the olefin hydrophosphorylation and deuterophosphorylation were successfully achieved under the standard conditions by affording the corresponding products **2al** and **2al-D** in excellent yields. In addition, when olefin tethered *L*-prolinol was subjected to the optimal conditions, the desired hydro-/deuterophosphonated products **2am** and **2am-D** were produced in good yields. Both cases showed excellent site-selectivity and good deuteration ratio, promising the broad applicability of this protocol in natural product modification. The practicability of the protocol was also demonstrated by gram-scale reactions, affording 1.06 g of **2a** and 1.19 g of **2al** in 56% and 76% yields, respectively. In addition, the product's usefulness was also proved by the derivatization of **2a**. Under the conditions of Appel reaction, Mitsunobu reaction and PCC oxidation, the hydroxyl group of **2a** can be respectively converted into bromo, azido and aldehyde groups, easily generating a series of valuable downstream products **3a–c**.

**Fig. 4:**
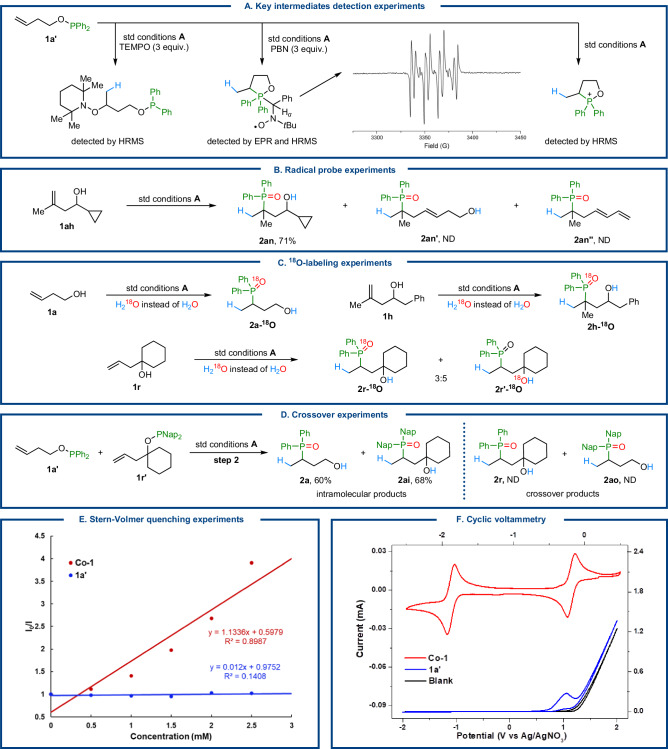
Mechanistic studies. **A** Key intermediates detection experiments. Key intermediates were detected by a high-resolution mass spectrometer (HRMS). A radical signal was detected by electron paramagnetic resonance (EPR), hyperfine-coupled splitting constants were calculated to be a_N_ = 14.52 G, a_P_ = 14.52 G, a_H*α*_ = 4.02 G. TEMPO = 2,2,6,6-tetramethylpiperidin-1-oxy radical, PBN = *N*-tert-butyl-*α*-phenylnitrone. **B** Radical probe experiment. **C** ^18^O isotope labeling experiment by using H_2_^18^O. **D** Crossover experiments. **E** Stern-Volmer quenching experiments. **F** Cyclic voltammetry.

To elucidate the reaction mechanism more deeply, a series of control experiments were conducted (Fig. 4). When 2,2,6,6-tetramethylpiperidin-1-oxy (TEMPO) was used as the C-atom-centered radical scavenger, the sample reaction was totally inhibited and the TEMPO-trapped alkyl-adduct was detected by high resolution mass spectrometer (HRMS, see Fig. S3 in the Supplementary Information), which indicates that the reaction involves a radical process (Fig. 4A). When *N*-tert-butyl-*α*-phenylnitrone (PBN), a spin trapping reagent, was involved in the sample reaction, a radical adduct of cyclic P-atom-centered radical intermediate with PBN was detected by electron paramagnetic resonance (EPR) experiment as well as HRMS (see Fig. S5 in the Supplementary Information). In addition, the quaternary phosphonium ion intermediate was also detected by HRMS (see Fig. S6 in the Supplementary Information) by directly using the sample reaction mixture. These results fully indicate that the reaction involves a radical cyclization of an alkyl radical derived from MHAT onto the P-atom as well as a further oxidation process of the phosphoranyl radical to the cyclic quaternary phosphonium. When cyclopropyl tethered homoallylic alcohol **1an** was subjected to the standard conditions, the reaction only gave the cyclopropyl ring remained product **2an** in 71% yield, and the ring-opening products were not detected (Fig. 4B). This result strongly implies that the present rearrangement does not involve the traditional radical cleavage of the C-O bond caused by the *β*-scission of phosphoranyl radicals, as no alkyl radical is generated after the rearrangement. In addition, to confirm the O-atom source of the phosphoryl moiety and further clarify the rearrangement processes, ^18^O isotopic tracing experiments were carried out by using H_2_^18^O instead of H_2_O under the standard conditions (Fig. 4C). The results clearly indicate that for primary and secondary alcohols, the O-atom of the phosphoryl moiety is almost derived from H_2_^18^O rather than the hydroxyl group of alcohols, indicating that the reaction mainly involves the cleavage of the P-O bond, with almost no cleavage of the C-O bond. While for tertiary alcohols, the O-atom of the phosphoryl moiety is derived from both H_2_^18^O and the hydroxyl group, which indicates that in addition to the cleavage of the P-O bond, the cleavage of the C-O bond is also feasible due to the formation of a relatively stable tertiary carbocation (see the Supplementary Information for details of ^18^O-labeling experiments and the corresponding mass spectra Fig. S7-S9). These results fully confirm that the reaction does indeed involve a water-mediated RPCR of phosphinite through P-O(C) bond cleavage as the key link. Crossover experiments by subjecting **1a’** and **1r’** to the standard conditions further confirm that the reaction involves phosphinite rearrangement as only intramolecular products were obtained and the crossover products were not detected (Fig. 4D). Stern-Volmer quenching experiments clearly indicate that the reaction initiation step begins from quenching the excited state of photocatalyst Ir(PPy)_3_ by the cobalt catalyst **Co-1** rather than the substrate **1a’** (Fig. 4E). Cyclic voltammetry analysis further suggests that this initiation step experiences the reduction of **Co-1** by the excited state of Ir(PPy)_3_ and does not involve the oxidation of **1a’** (Fig. 4F).

**Fig. 5:**
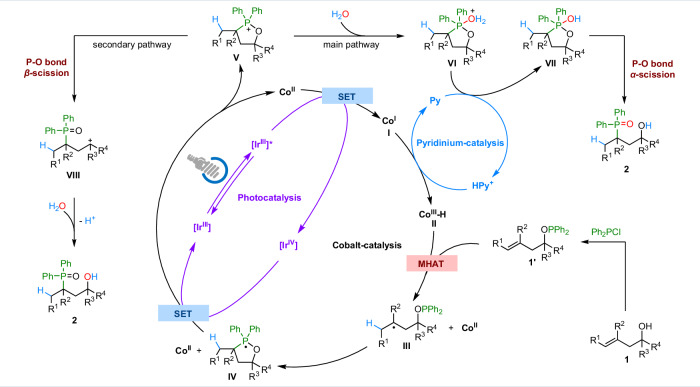
Proposed mechanism. Mechanism for radical Markovnikov hydrophosphorylation of olefins via a triple-catalytic water-mediated phosphinite rearrangement.

On the basis of our experiment observations and literature reports, a proposed mechanism for this triple-catalyzed radical Markovnikov hydrophosphorylation of olefins is illustrated in Fig. 5. The reaction initiates from the excitation of photocatalyst [Ir^III^] to its excited state [Ir^III^]* under visible light irradiation, and the latter subsequently reduces cobalt catalyst Co^II^ to Co^I^ species **I** via a single-electron transfer (SET) process, while itself is oxidized to [Ir^IV^]. **I** then reacts with pyridinium **HPy**^**+**^ to produce Co^III^-H species **II**, accompanied by the release of pyridine **Py**. Co^III^-H initiates an MHAT process with substrates **1’**, generating the alkyl radical **III** and regenerating the cobalt catalyst. Intramolecular radical cyclization of **III** would give the cyclic P-atom-centered radical intermediate **IV**. The SET oxidation of **IV** by [Ir^IV^] produces the quaternary phosphonium ion intermediate **V**, accompanied by the regeneration of photocatalyst [Ir^III^]. **V** is immediately trapped by H_2_O to produce phosphoranyl intermediate **VI**. Finally, **VI** is deprotonated by **Py** to yield **VII**, which subsequently undergoes the P-O bond *α*-scission to yield the product **2**. Alternatively, **2** could also be generated by the secondary pathway of a C-O bond cleavage of the intermediate **V** according to ^18^O isotope tracing experiments, especially for tertiary alcohols.

In summary, we have successfully developed a visible-light-driven triple catalytic approach for the radical Markovnikov hydrophosphorylation of unactivated alkenes by a sequential reaction of (bis)homoallylic alcohols, Ph_2_PCl, and water. The protocol initiates from a radical Markovnikov MHAT between Co-H species and the in situ formed unsaturated phosphinites derived from the condensation of (bis)homoallylic alcohols and Ph_2_PCl, followed by a water-mediated radical-ionic crossover phosphinite rearrangement to regioselectively install the in-situ generated phosphoryl to the inner side of the terminal olefins. Notably, the value and significance of the protocol are further reflected in the successful Markovnikov deuterophosphorylation of alkenes by using D_2_O to replace H_2_O, which showcases good deuteration regioselectivity and ratio. This protocol not only broadens the boundaries of the rearrangement reaction of phosphorous, but also affords a radical-ionic crossover activation mode of water by the complete splitting and utilization of all three atoms of a water molecule. Further exploration of water-mediated radical reactions and radical rearrangement modes of phosphinite for synthetic purposes is ongoing in our laboratory.

## Methods

### General procedure for hydrophosphorylation of olefin (Conditions A)

#### General step 1

To a glass tube equipped with a magnetic stir bar, olefinic alcohols **1** (0.2 mmol, 1.0 equiv.), anhydrous THF (1 mL), 4-dimethylaminopyridine (DMAP, 20.0 mol%), and Et_3_N (0.24 mmol, 1.2 equiv.) were added under argon atmosphere, followed by R_2_PCl (0.22 mmol, 1.1 equiv.) dropwise by microsyringe at 0 °C. The mixture was further stirred at room temperature (about 1–5 h) until olefinic alcohols were completely consumed, as monitored by TLC. The reaction solution was filtered through a neutral alumina column under an argon atmosphere. The column was further washed by a mixed solvent (petroleum/ethyl acetate, 50:1) under an argon atmosphere. The combined filtrate was concentrated under vacuum to give the corresponding phosphinite **1’**.

#### General step 2

To an oven-dried transparent glass tube equipped with a magnetic stir bar, **Co-1** (5.0 mol%), **HPy-1** (20.0 mol%), and Ir(ppy)_3_ (1.0 mol%) were added. The reaction tube was evacuated and back-filled with argon for three times, and then the obtained phosphinite **1’** in step 1 was dissolved in DMA (2 mL) and transferred to the reaction tube. Subsequently, H_2_O (0.3 mmol, 1.5 equiv.) was added by microsyringe under an argon atmosphere. The reaction mixture was stirred at room temperature for 14 h under blue LEDs (*λ*_max_ = 455 nm, 6 W) irradiation. Upon reaction completion, monitored by TLC, the mixture was diluted with EtOAc. The combined organic layers were washed with brine, dried over MgSO_4_, filtered and the solvents were removed under reduced pressure. The crude product was purified by column chromatography (gradient eluent of ethyl acetate and petroleum ether) to give the corresponding product.

### General procedure for deuterophosphorylation of olefin (Conditions B)

#### General step1

To a glass tube equipped with a magnetic stir bar, olefinic alcohols **1** (0.2 mmol, 1.0 equiv.), anhydrous THF (1 mL), 4-dimethylaminopyridine (DMAP, 20.0 mol%), and Et_3_N (0.24 mmol, 1.2 equiv.) were added under argon atmosphere, followed by R_2_PCl (0.22 mmol, 1.1 equiv.) dropwise by microsyringe at 0 ^o^C. The mixture was further stirred at room temperature (about 1–5 h) until olefinic alcohols were completely consumed, as monitored by TLC. The reaction solution was filtered through a neutral alumina column under an argon atmosphere. The column was further washed by a mixed solvent (petroleum/ethyl acetate, 50:1) under an argon atmosphere. The combined filtrate was concentrated under vacuum to give the corresponding phosphinite **1’**.

#### General step 2

To an oven-dried transparent glass tube equipped with a magnetic stir bar, **Co-1** (5.0 mol%), **DPy-1** (20.0 mol%), and Ir(ppy)_3_ (1.0 mol%) were added, and the reaction tube was evacuated and back-filled with argon for 3 times. Under argon atmosphere, the obtained phosphinite **1’** in step 1 was dissolved in DMA (2 mL) and transferred to the reaction tube, followed by adding D_2_O (0.3 mmol, 1.5 equiv.) by microsyringe. The reaction mixture was stirred at room temperature for 14 h under blue LEDs (*λ*_max_ = 455 nm, 6 W) irradiation. Upon reaction completion, monitored by TLC, the mixture was diluted with EtOAc. The combined organic layers were washed with brine, dried over MgSO_4_, filtered and the solvents were removed under reduced pressure. The crude product was purified by column chromatography (gradient eluent of ethyl acetate and petroleum ether) to give the corresponding product.

## Supplementary information

Supplementary Information

Peer Review file

## Data Availability

All information relating to optimization studies, experimental procedures, mechanistic studies, NMR spectra, and high-resolution mass spectrometry is available in Supplementary Information. Crystallographic data for the structure reported in this study have been deposited at the Cambridge Crystallographic Data Center (CCDC) under deposition number 2481386 (for **2k**). These data can be obtained free of charge from The Cambridge Crystallographic Data Center via www.ccdc.cam.ac.uk/structures/. The authors declare that all other data supporting the findings of this study are available within the paper and its supplementary information files. All data are available from the corresponding author, Bing Han, upon request.
