# Supplementary material for: Catalytic Markovnikov hydrophosphorylation of unactivated olefins via a radical-polar crossover rearrangement
Source: Nat Commun. 2026 May 16;17:6500. doi: 10.1038/s41467-026-72870-2 (PMC13376919; doi:10.1038/s41467-026-72870-2)
Supplement: Supplementary file 1 — Supplementary Information [file 41467_2026_72870_MOESM1_ESM.pdf]

Supplementary Information

Catalytic Markovnikov Hydrophosphorylation of Unactivated Olefins via a Radical-Polar Crossover Rearrangement

Yi-Fan Li[1,#], Hong-Chen Wang[1,#], and Bing Han[1,*]

[1]State Key Laboratory of Nature Product Chemistry, College of Chemistry and Chemical Engineering, Lanzhou University, Lanzhou 730000, China; *Email: hanb@lzu.edu.cn

Contents

1 General information……………………………………………………………………………………….S2

2 General setup...……………………………………………………………..…………………………......S2

3 Preparation of starting material olefinic alcohols……………………………………………………..…S3

4 General procedures for hydro(deutro)phosphorylation of olefins……..…………………………….S6

5 Gram-scale synthesis...……….……………………….S7

6 Control experiments and mechanism investigation…………………………………………..……..S9

6.1 Key intermediates detection experiments……..…………………..……………………….S9

6.2 Radical probe experiment..…………………………..…………….………………………..S11

6.3 ^{18}O-labeling experiments………………………..…………………………...………...…..S11

6.4 Crossover experiment…...……….....S14

6.5 UV-Vis absorption spectroscopy………………………..…….……………………………..…S15

6.6 Cyclic voltammetry studies…………………………………….………………………………S15

6.7 Stern-Volmer quenching experiments…………………………..……………………………S16

7 X-Ray single-crystal diffraction study for 2k..………………………………………..………………S17

8 Analytical data for products…………………………..…………..……………………………………S18

9 Copies of ^{1}H, ^{2}H, ^{13}C, ^{19}F and ^{31}P NMR spectra……………………………………………………S40

10 Supplementary References………………………………..................……………………..…S230

1 General information

All reagents were purchased from commercial suppliers and used without further purification. Column chromatography purification of products was accomplished using forced-flow chromatography on Silica Gel (200-300 mesh). Analytical TLC was performed with silica gel GF254 plates, and the products were visualized by UV detection. ^{1}H, ^{13}C, ^{19}F and ^{31}P NMR spectra were recorded in $CDCl_3$ on a Bruker AVANCE NEO 400 MHz spectrometer. ^{2}H NMR spectra were recorded in $CHCl_3$ on a Bruker AVANCE NEO 600 MHz spectrometer. Chemical shifts are given in ppm relative to the residual solvent peak (^{1}H NMR: $CHCl_3$ δ = 7.26; TMS = 0.0; ^{2}H NMR: $CDCl_3$ δ = 7.26; ^{13}C NMR: $CDCl_3$ δ = 77.00) with multiplicity (br = broad, s = singlet, d = doublet, t = triplet, q = quartet, m = multiplet), coupling constants (in Hz) and integration. High-resolution mass spectrometry (HRMS) analyses were performed on a Thermo Scientific Exploris 120 mass spectrometer with electrospray ionization (ESI). Gas chromatography-mass spectra (GC-MS) were measured on a Trace DSQ mass spectrometer with electron ionization (EI). Data collections for crystal structure were performed at room temperature (149.92 K) using MoKα radiation on a Bruker APEXII diffractometer. Integration of the frames and data reduction was carried out using SAINT. The structure was solved by direct methods using SHELXS-97. Electron paramagnetic resonance (EPR) experiments were performed on a Bruker ER200DSRC10/12 EPR spectrometer and data were recorded on X band. The calculated hyperfine splitting g values are obtained by using 2,2-diphenyl-1-picrylhydrazyl (DPPH) as a standard. Cyclic voltammetry (CV) experiments were performed using an AUTOLAB Electrochemical workstation. UV-Vis absorption spectra were recorded on a Lambda 950+Refle UV/Vis spectrometer. Photochemical reactions were carried out with 6 W blue LEDs and 30 W blue LEDs obtained from Wuhan Jiushang Technology Co., Ltd and Suzhou Youjing photoelectric Technology Co., Ltd, respectively.

2 General setup

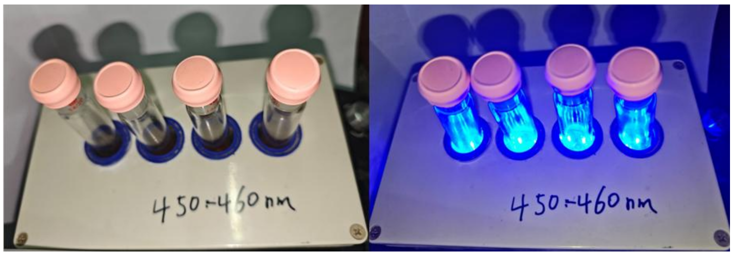

Figure S1. General setup for the reaction. Blue LEDs (λ_{max} = 455 nm, 4×6 W) were used as the light source.

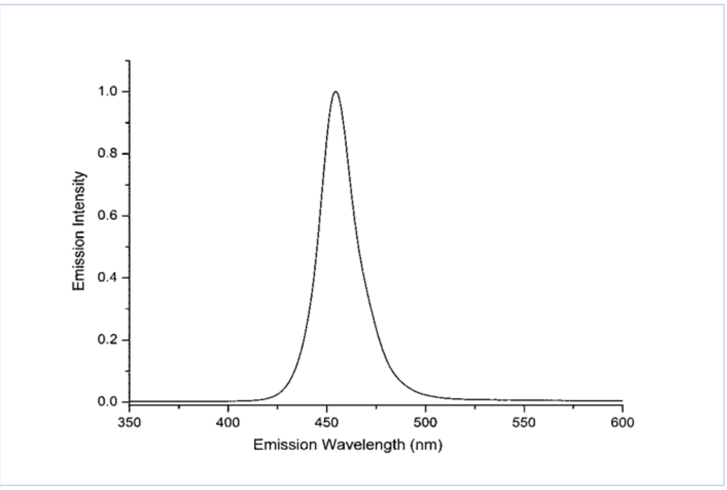

Figure S2. Emission spectrum of the blue LED (λ_{max}= 455 nm).

3 Preparation of starting material olefinic alcohols

Olefinic alcohols **1a**-**1f**, **1h**, **1j**-**1l**, **1p**-**1y**, **1aa**-**1ad**, **1af** and **1ah** were commercially available and purchased from the supplier.

A general Barbier reaction procedure

Olefinic alcohols **1g**, **1i**, **1m**-**1o**, **1z**, **1ae**, and **1ag** were synthesized by a general Barbier reaction procedure as shown below.

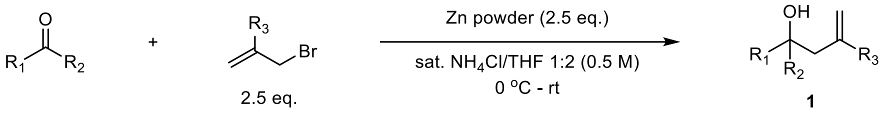

To a solution of aldehydes or ketones (10.0 mmol) and zinc powder (1.63 g, 25.0 mmol) in saturated NH_4Cl solution/THF (1:2; 20 mL) was added olefinic (25.0 mmol) at 0 °C. Then reaction mixture was moved to room temperature and stirred for 8 h. After this time, reaction mixture was filtered through Celite and extracted with EtOAc three times. The combined organic layer was washed with brine, dried over $MgSO_4$, and concentrated in vacuo. The crude mixture was purified by flash chromatography using a mixed solvent of petroleum ether (PE) and ethyl acetate (EA) as the eluent.

2-methyldec-1-en-4-ol (1g)

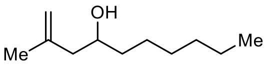

This compound was synthesized by **a general Barbier reaction procedure**; Column chromatography conditions (PE/EA = 40:1 to 10:1), R_f = 0.20 (PE/EA = 10:1), Colorless liquid, 1.11 g, 66%; ^{1}H NMR (400 MHz, $CDCl_3$): δ 4.87 (s, 1H), 4.79 (s, 1H), 3.75-3.69 (m, 1H), 2.20 (dd, J_1 = 14.0 Hz, J_2 = 4.0 Hz, 1H), 2.11 (dd, J_1 = 14.0 Hz, J_2 = 9.2 Hz, 1H), 1.97 (s, 1H), 1.76 (s, 3H), 1.49-1.45 (m, 3H), 1.33-1.28 (m, 7H), 0.89 (t,

J = 6.8 Hz, 3H); ^{13}C NMR (100 MHz, $CDCl_3$): δ 142.8, 113.2, 68.6, 46.1, 37.1, 31.8, 29.3, 25.6, 22.5, 22.3, 14.0; HRMS (ESI) m/z: $[M+H]^+$ Calcd for $C_{11}H_{23}O^+$ 171.1743, Found 171.1737.

1-cyclopentyl-3-methylbut-3-en-1-ol (1i)

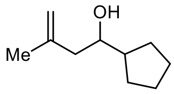

This compound was synthesized by **a general Barbier reaction procedure;** Column chromatography conditions (PE/EA = 40:1 to 10:1), R_f = 0.20 (PE/EA = 10:1), Yellow oil, 1.08 g, 70%; ^{1}H NMR (400 MHz, $CDCl_3$): δ 4.89-4.88 (m, 1H), 4.81-4.80 (m, 1H), 3.55-3.50 (m, 1H), 2.27 (dd, J_1 = 14.0 Hz, J_2 = 2.4 Hz, 1H), 2.06 (dd, J_1 = 14.0 Hz, J_2 = 10.0 Hz, 1H), 1.92-1.81 (m, 2H), 1.78-1.75 (m, 4H), 1.72-1.67 (m, 1H), 1.65-1.52 (m, 4H), 1.46-1.39 (m, 1H), 1.28-1.21 (m, 1H); ^{13}C NMR (100 MHz, $CDCl_3$): δ 143.1, 113.3, 72.5, 46.0, 45.1, 29.1, 28.6, 25.4, 25.61, 22.3; HRMS (ESI) m/z: $[M+H]^+$ Calcd for $C_{10}H_{19}O^+$ 155.1430, Found 155.1428.

2-benzyl-1-phenylpent-4-en-2-ol (1m)

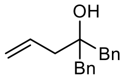

This compound was synthesized by **a general Barbier reaction procedure;** Column chromatography conditions (PE/EA = 40:1 to 10:1), R_f = 0.20 (PE/EA = 10:1), Colorless liquid, 2.02 g, 80%; ^{1}H NMR (400 MHz, $CDCl_3$): δ 7.30-7.19 (m, 10H), 5.98-5.88 (m, 1H), 5.18-5.06 (m, 2H), 2.79-2.77 (m, 4H), 2.15-2.13 (m, 2H), 1.54 (s, 1H); ^{13}C NMR (100 MHz, $CDCl_3$): δ 137.2, 134.0, 130.7, 128.1, 126.4, 118.9, 73.8, 45.5, 43.0; HRMS (ESI) m/z: $[M+H]^+$ Calcd for $C_{18}H_{21}O^+$ 253.1587, Found 253.1582.

6-allylundecan-6-ol (1n)

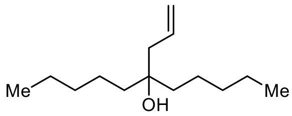

This compound was synthesized by **a general Barbier reaction procedure;** Column chromatography conditions (PE/EA = 40:1 to 15:1), R_f = 0.30 (PE/EA = 10:1), Colorless liquid, 1.27 g, 60%; ^{1}H NMR (400 MHz, $CDCl_3$): δ 5.89-5.79 (m, 1H), 5.14-5.08 (m, 2H), 2.21 (d, J = 7.2 Hz, 2H), 1.44-1.26 (m, 17H), 0.89 (t, J = 7.2 Hz, 6H); ^{13}C NMR (100 MHz, $CDCl_3$): δ 134.0, 118.3, 73.9, 43.9, 39.1, 32.4, 23.1, 22.6, 14.0; HRMS (ESI) m/z: $[M+H]^+$ Calcd for $C_{14}H_{29}O^+$ 213.2213, Found 213.2206.

4-methylnon-1-en-4-ol (1o)

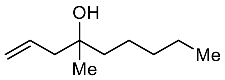

This compound was synthesized by **a general Barbier reaction procedure;** Column chromatography conditions (PE/EA = 40:1 to 15:1), R_f = 0.30 (PE/EA = 10:1), Colorless liquid, 0.97 g, 62%; ^{1}H NMR (400 MHz, $CDCl_3$): δ 5.91-5.81 (m, 1H), 5.14-5.08 (m, 2H), 2.22 (d, J = 6.4 Hz, 2H), 1.68 (s, 1H), 1.45-1.43 (m, 2H), 1.35-1.27 (m, 6H), 1.16 (s, 3H), 0.89 (t, J = 6.8 Hz, 3H); ^{13}C NMR (100 MHz, $CDCl_3$): δ 134.1, 118.3, 72.1, 46.2, 41.7, 32.3, 26.6, 23.4, 22.5, 13.9; HRMS (ESI) m/z: [M+H]$^+$ Calcd for $C_{10}H_{21}O^+$ 157.1587, Found 157.1585.

2-allyl-2,3-dihydro-1H-inden-2-ol (1z)

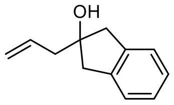

This compound was synthesized by **a general Barbier reaction procedure;** Column chromatography conditions (PE/EA = 40:1 to 10:1), R_f = 0.2 (PE/EA = 10:1), Yellow oil, 1.46 g, 84%; ^{1}H NMR (400 MHz, $CDCl_3$): δ 7.21-7.12 (m, 4H), 5.99-5.88 (m, 1H), 5.19-5.15 (m, 2H), 3.04 (d, J = 16.4 Hz, 2H), 2.90 (d, J = 16.4 Hz, 2H), 2.47 (d, J = 7.2 Hz, 2H), 2.14 (s, 1H); ^{13}C NMR (100 MHz, $CDCl_3$): δ 141.1, 133.9, 126.5, 124.9, 118.8, 81.4, 46.3, 44.9; HRMS (ESI) m/z: [M+H]$^+$ Calcd for $C_{12}H_{15}O^+$ 175.1117, Found 175.1112.

2-(cyclohex-2-en-1-yl)propan-2-ol (1ae)

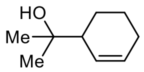

This compound was synthesized by **a general Barbier reaction procedure**; Column chromatography conditions (PE/EA = 40:1 to 15:1), R_f = 0.25 (PE/EA = 10:1), Colorless liquid, 0.84 g, 60%; ^{1}H NMR (400 MHz, $CDCl_3$): δ 5.84-5.81 (m, 1H), 5.76-5.73 (m, 1H), 2.18-2.15 (m, 1H), 1.98-1.96 (m, 2H), 1.85-1.80 (m, 2H), 1.67 (s, 1H), 1.55-1.45 (m, 1H), 1.34-1.24 (m, 1H), 1.21(s, 3H), 1.16 (s, 3H); ^{13}C NMR (100 MHz, $CDCl_3$): δ 129.6, 127.5, 72.9, 46.8, 27.7, 26.1, 25.1, 24.5, 22.2; HRMS (ESI) m/z: [M+H]$^+$ Calcd for $C_9H_{17}O^+$ 141.1274, Found 141.1271.

tert-butyl (S)-2-(1-hydroxy-3-methylbut-3-en-1-yl)pyrrolidine-1-carboxylate (1ag)

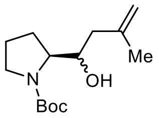

This compound was synthesized by **a general Barbier reaction procedure**; Column chromatography conditions (PE/EA = 40:1 to 5:1), R_f = 0.4 (PE/EA = 5:1), dr = 1:1.1, Colorless liquid, 2.22 g, 87%; ^{1}H NMR (400 MHz, $CDCl_3$): δ 4.84-4.79 (m, 4.2H), 4.09 (br, 2H), 3.87 (br, 2.2H), 3.56-3.51 (m, 2.1H), 3.30-3.24 (m, 2.1H), 2.12-2.10 (m, 4.2H), 1.94-1.70 (m, 14.7H), 1.47 (s, 18.9H); ^{13}C NMR (100 MHz, $CDCl_3$): δ 155.6,

154.9, 142.7, 142.6, 112.6, 79.4, 70.2, 69.6, 62.3, 61.7, 47.6, 42.0, 41.3, 28.3, 26.6, 25.5, 24.0, 22.3; HRMS (ESI) m/z: $[M+H]^+$ Calcd for $C_{14}H_{26}NO_3^+$ 256.1907, Found 256.1911.

4 General procedures for hydro(deutro)phosphorylation of olefins

General procedure for the olefin hydrophosphorylation (Standard conditions A)

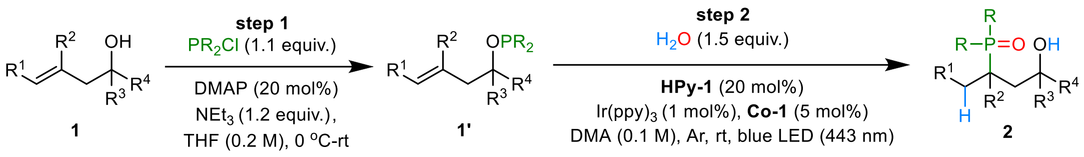

General step 1: To a glass tube equipped with a magnetic stir bar, olefinic alcohols **1** (0.2 mmol, 1.0 equiv.), anhydrous THF (1 mL), 4-dimethylaminopyridine (DMAP, 20.0 mol%), and Et_3N (0.24 mmol, 1.2 equiv.) were added and stirred under argon atmosphere, followed by adding R_2PCl (0.22 mmol, 1.1 equiv.) dropwise using microsyringe at 0 °C. The mixture was stirred at room temperature until olefinic alcohols were completely consumed as monitored by TLC. The reaction solution was filtered by neutral alumina column under argon atmosphere. The column was further washed by a mixed solvent of petroleum ether (PE) and ethyl acetate (EA) as eluent (PE/EA, 50:1) under argon atmosphere. The combined filtrate was concentrated under vacuum to give the corresponding phosphinite **1'**.

General step 2: To an oven-dried transparent glass tube equipped with a magnetic stir bar, **Co-1** (5.0 mol%), **HPy-1** (20.0 mol%), and $Ir(ppy)_3$ (1.0 mol%) were added, and the reaction tube was evacuated and back-filled with argon for 3 times. Under argon atmosphere, the obtained phosphinite **1'** in step 1 was dissolved in DMA (2 mL) and transferred in the reaction tube, followed by adding H_2O (0.3 mmol, 1.5 equiv.) by microsyringe. The reaction mixture was stirred at room temperature for 14 h under blue LEDs (λ_{max} = 455 nm, 6 W) irradiation. Upon reaction completion monitored by TLC, the mixture was diluted with EtOAc. The combined organic layers were washed with brine, dried over $MgSO_4$, filtered and the solvents were removed under reduced pressure. The crude product was purified by column chromatography (gradient eluent of ethyl acetate and petroleum ether) to give the corresponding product. The identity and purity of the product were determined by ^1H, ^{13}C, ^{19}F and ^{31}P NMR spectroscopic analysis.

General procedure for the olefin deuterophosphorylation (Standard conditions B)

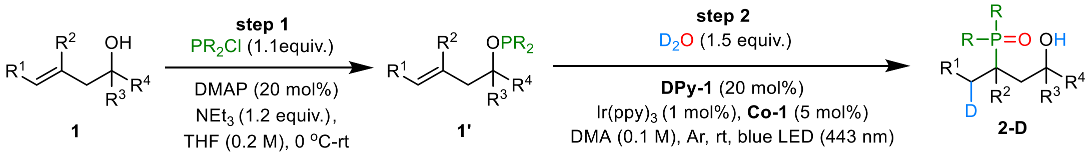

General step 1: To a glass tube equipped with a magnetic stir bar, olefinic alcohols **1** (0.2 mmol, 1.0 equiv.), anhydrous THF (1 mL), 4-dimethylaminopyridine (DMAP, 20.0 mol%), and Et_3N (0.24 mmol, 1.2 equiv.) were added and stirred under argon atmosphere, followed by adding R_2PCl (0.22 mmol, 1.1 equiv.) dropwise using microsyringe at 0 °C. The mixture was stirred at room temperature until olefinic alcohols were completely consumed as monitored by TLC. The reaction solution was filtered by neutral alumina column under argon atmosphere. The column was further washed by a mixed solvent of petroleum ether (PE) and ethyl acetate (EA) as eluent (PE/EA, 50:1) under argon atmosphere. The combined filtrate was concentrated under vacuum to give the corresponding phosphinite **1'**.

General step 2: To an oven-dried transparent glass tube equipped with a magnetic stir bar, **Co-1** (5.0 mol%), **HPy-1** (20.0 mol%), and $Ir(ppy)_3$ (1.0 mol%) were added, and the reaction tube was evacuated and back-filled with argon for 3 times. Under argon atmosphere, the obtained phosphinite **1'** in step 1 was dissolved in DMA (2 mL) and transferred in the reaction tube, followed by adding D_2O (0.3 mmol, 1.5 equiv.) by microsyringe. The reaction mixture was stirred at room temperature for 14 h under blue LEDs (λ_{max} = 455 nm, 6 W) irradiation. Upon reaction completion monitored by TLC, the mixture was diluted with EtOAc. The combined organic layers were washed with brine, dried over $MgSO_4$, filtered and the solvents were removed under reduced pressure. The crude product was purified by column chromatography (gradient eluent of ethyl acetate and petroleum ether) to give the corresponding product. The identity and purity of the product were confirmed by ^1H, ^2H, ^{13}C, and ^{31}P NMR spectroscopic analysis.

5 Gram-scale synthesis

Gram-scale synthesis of 2a

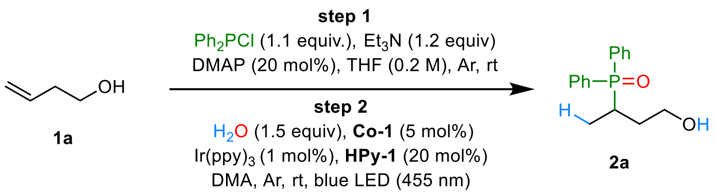

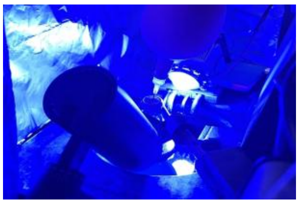

General step 1: To a glass bottle equipped with a magnetic stir bar, homoallylic alcohol **1a** (7.0 mmol, 1.0 equiv.), anhydrous THF (20 mL), 4-dimethylaminopyridine (DMAP, 20.0 mol%), and Et_3N (8.4 mmol, 1.2 equiv.) were added and stirred under argon atmosphere, followed by adding Ph_2PCl (7.7 mmol, 1.1 equiv.) dropwise using microsyringe at 0 °C. The mixture was stirred at room temperature until homoallylic alcohol was completely consumed as monitored by TLC. The reaction solution was filtered by neutral alumina column under argon atmosphere. The column was further washed by a mixed solvent (PE/EA,

50:1) under argon atmosphere. The combined filtrate was concentrated under vacuum to give the corresponding phosphinite **1a'**.

General step 2: To an oven-dried transparent glass tube equipped with a magnetic stir bar, **Co-1** (5.0 mol%), **HPy-1** (20.0 mol%), and Ir(ppy)$_3$ (1.0 mol%) were added, and the reaction tube was evacuated and back-filled with argon for 3 times. Under argon atmosphere, the obtained phosphinite **1a'** in step 1 was dissolved in DMA (50 mL) and transferred in the reaction tube, followed by adding H_2O (13.5 mmol, 1.5 equiv.) by microsyringe. The reaction mixture was stirred at room temperature for 16 h under blue LEDs (λ_{max} = 455 nm, 30 W) irradiation. Upon reaction completion monitored by TLC, the mixture was diluted with EtOAc. The combined organic layers were washed with brine, dried over $MgSO_4$, filtered and the solvents were removed under reduced pressure. The crude product was purified by column chromatography (gradient eluent of ethyl acetate and petroleum ether) to give the corresponding product **2a** (1.06 g, 3.89 mmol, 56% yield). The identity and purity of the product was confirmed by ^{1}H, ^{13}C and ^{31}P NMR spectroscopic analysis.

Gram-scale synthesis of 2al

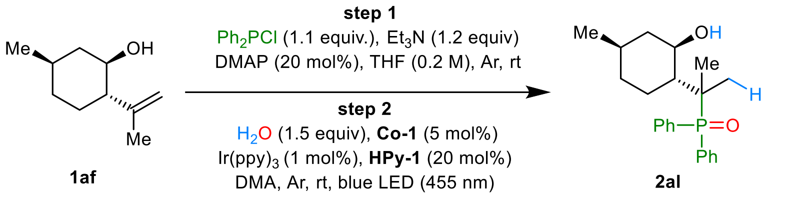

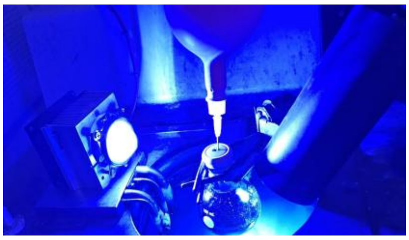

General step 1: To a glass bottle equipped with a magnetic stir bar, (-)-isopulegol **1af** (4.4 mmol, 1.0 equiv.), anhydrous THF (15 mL), 4-dimethylaminopyridine (DMAP, 20.0 mol%), and Et_3N (5.3 mmol, 1.2 equiv.) were added and stirred under argon atmosphere, followed by adding R_2PCl (4.8 mmol, 1.1 equiv.) dropwise using microsyringe at 0 °C. The mixture was stirred at room temperature until isopulegol was completely consumed as monitored by TLC. The reaction solution was filtered by neutral alumina column under argon atmosphere. The column was further washed by a mixed solvent (PE/EA, 50:1) under argon atmosphere. The combined filtrate was concentrated under vacuum to give the corresponding phosphinite **1af'**.

General step 2: To an oven-dried transparent glass tube equipped with a magnetic stir bar, **Co-1** (5.0 mol%), **DPy-1** (20.0 mol%), and Ir(ppy)$_3$ (1.0 mol%) were added, and the reaction tube was evacuated and back-filled with argon for 3 times. Under argon atmosphere, the obtained phosphinite **1af'** in step 1

was dissolved in DMA (40 mL) and transferred in the reaction tube, followed by adding H_2O (6.6 mmol, 1.5 equiv.) by microsyringe. The reaction mixture was stirred at room temperature for 16 h under blue LEDs (λ_{max} = 455 nm, 30 W) irradiation. Upon reaction completion monitored by TLC, the mixture was diluted with EtOAc. The combined organic layers were washed with brine, dried over $MgSO_4$, filtered and the solvents were removed under reduced pressure. The crude product was purified by flash column chromatography (gradient eluent of ethyl acetate and petroleum ether) to give the corresponding product **2al** (1.18 g, 3.30 mmol, 76% yield). The identity and purity of the product was confirmed by ^{1}H, ^{13}C and ^{31}P NMR spectroscopic analysis.

6 Control experiments and mechanism investigation

6.1 Key intermediates detection experiments

TEMPO trapping experiment

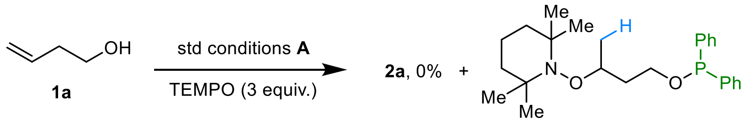

In the sample reaction of **1a**, TEMPO (2,2,6,6-tetramethyl-piperidine-*N*-oxyl, 0.6 mmol, 3.0 equiv.) was added to step 2 of standard conditions **A**, and stirred at room temperature for 12 h under irradiation with 6 W blue LEDs (λ_{max} = 455 nm). The adduct produced by TEMPO trapping alkyl radicals was detected by high-resolution mass spectrometry (HRMS) with electrospray ionization (ESI).

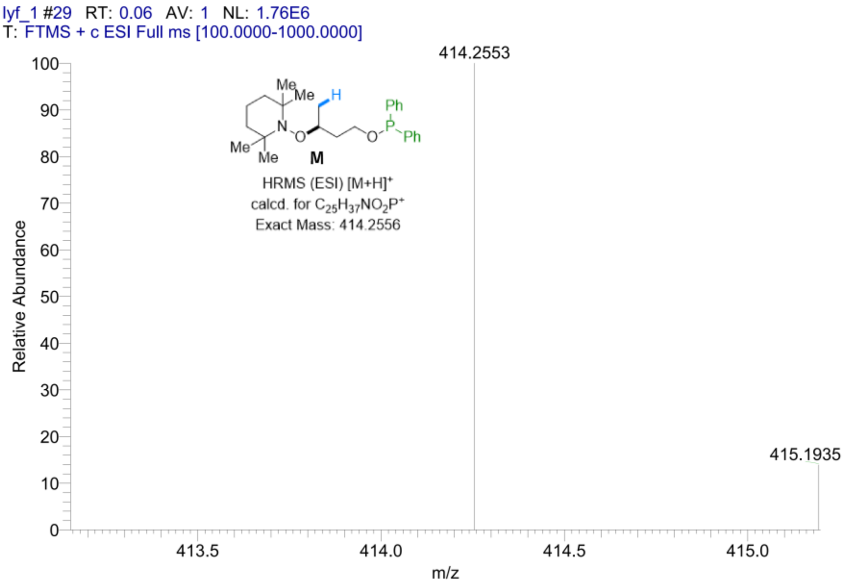

Figure S3. HRMS spectrum of the TEMPO-alkyl radical adduct.

Detection of the cyclic phosphoranyl radical by EPR experiments

In the sample reaction of **1a**, PBN (*N*-tert-butyl-*α*-phenylnitrone, 0.3 mmol, 53.1 mg) was added to step 2 of standard conditions **A,** and stirred at room temperature for 1.5 h under irradiation with 6 W blue LEDs (λ_{max} = 455 nm). The octuplet radical signal of PBN-trapped cyclic phosphoranyl radical was detected by EPR (electron paramagnetic resonance) experiment. In addition, the PBN-trapped cyclic phosphoranyl radical was also detected by HRMS with electrospray ionization (ESI).

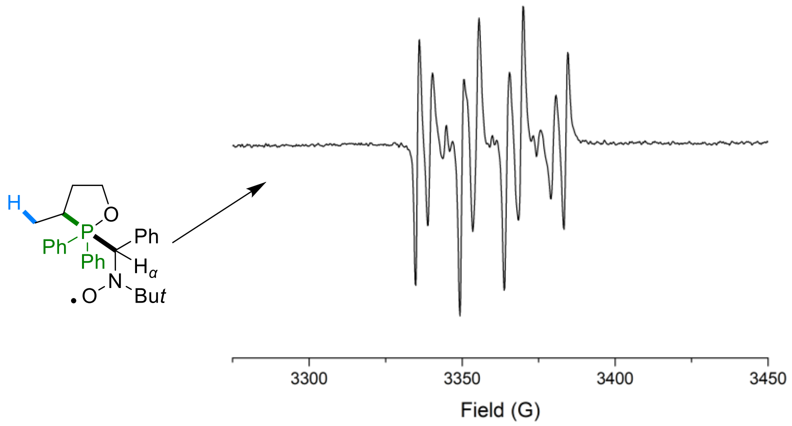

Figure S4. EPR signal of PBN-trapped cyclic phosphoranyl radical (X band, 9.4 GHz). The hyperfine coupling constants are calculated to be a_P = 14.52 G; a_N = 14.52 G; $a_{H\alpha}$ = 4.02 G; g = 2.0069.

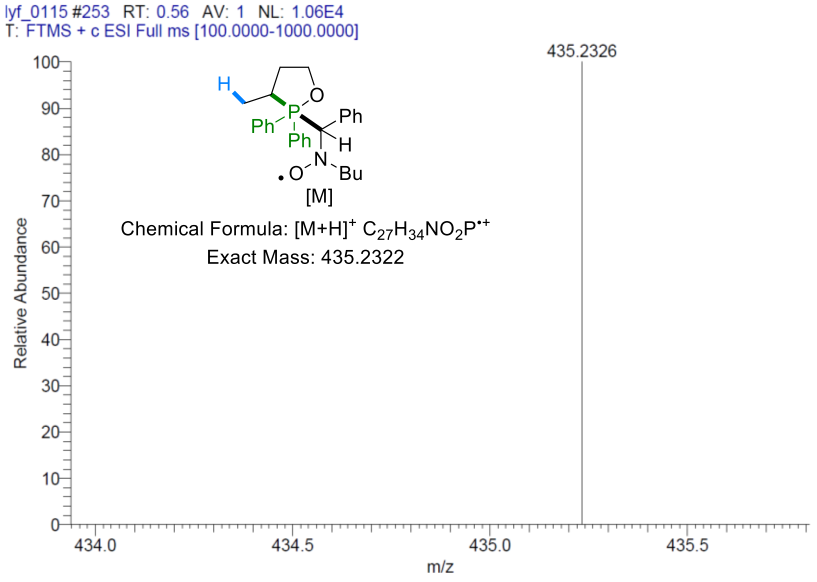

Figure S5. HRMS spectrum of the PBN-trapped cyclic phosphoranyl radical adduct.

Detection of the cyclic quaternary phosphonium ion

When **1a** was subjected to light irradiation under standard condition **A** for 1.5 h, the reaction solution was directly used for mass spectrometry detection, and a cyclic quaternary phosphonium ion was detected by HRMS with electrospray ionization (ESI) as shown below.

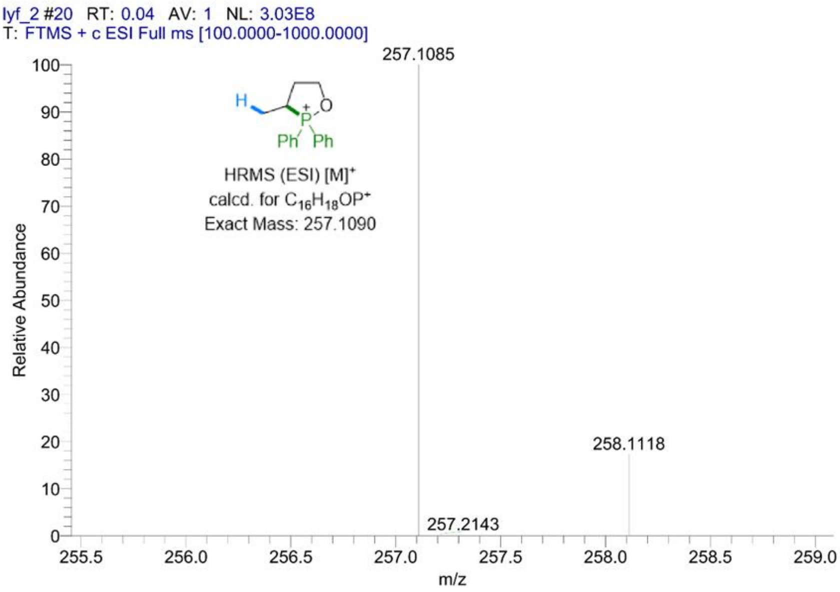

Figure S6. HRMS (ESI) spectrum of the PBN-cyclic cyclic phosphoranyl radical adduct.

6.2 Radical probe experiment

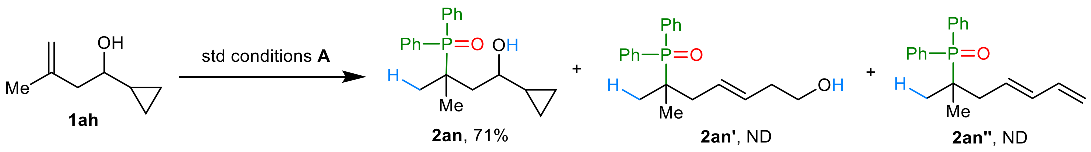

When olefinic alcohol **1ah** was subjected to the **standard conditions A**, the reaction gave the corresponding product **2an** in 71% yield and without the observation of the ring-opening products The identity and purity of the product **2an** were determined by ^{1}H, ^{13}C, and ^{31}P NMR spectroscopic analysis.

6.3 ^{18}O-labeling experiments

Determination method for the ^{18}O source of primary alcohol product

Compounds **2a-^{18}O** and **2a'-^{18}O** were obtained by the general procedure of **conditions A** in which $H_2{}^{18}O$ participates in the reaction instead of H_2O (31.8 mg, 0.166 mmol, 70% yield, **2a-^{18}O/2a'-^{18}O** = 10:1).

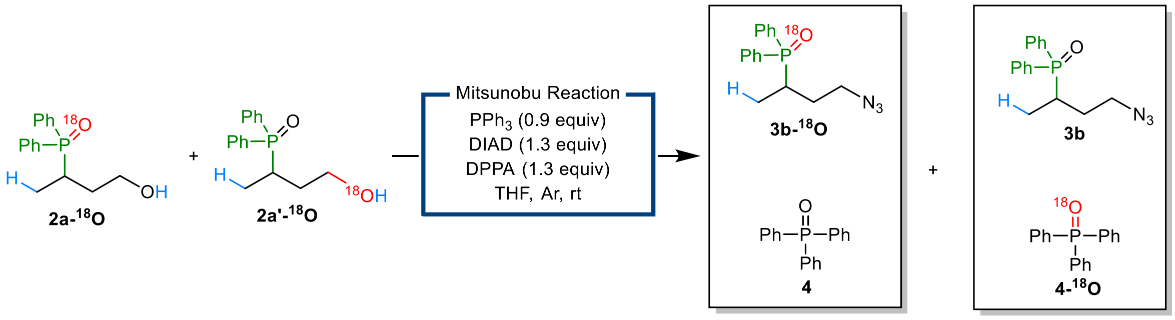

To a solution of **2a-^{18}O** and **2a'-^{18}O** mixture (0.2 mmol, 1.0 equiv.) in dry THF (2 mL) was added recrystallized PPh_3 (0.9 equiv.), DIAD (1.3 equiv.), and then DPPA (1.3 equiv.) was added dropwise at 0

°C under an argon atmosphere. The mixture was further stirred at room temperature until the starting material PPh_3 was consumed completely as monitored by TLC analysis. MS analysis with electron ionization (EI) gives a ratio of **4-^{18}O/4** = 1:10, which indicates that their parent molecular ratio is **2a-^{18}O/2a'-^{18}O** = 10:1.

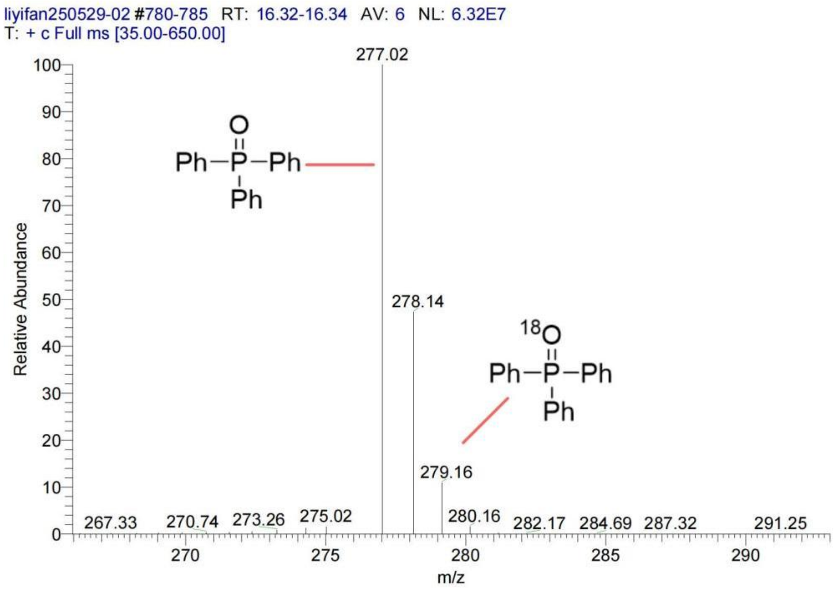

Figure S7. Mass spectrum of the mixture of **4-^{18}O** and **4** derived from **2a-^{18}O and 2a'-^{18}O**.

Determination method for the ^{18}O source of secondary alcohol product

The compounds **2ah-^{18}O/2ah'-^{18}O** were obtained by the general procedure of **conditions A** in which $H_2{}^{18}O$ participates in the reaction instead of H_2O (118.1 mg, 0.31 mmol, 72% yield, **2ah-^{18}O/2ah'-^{18}O** = 20:1).

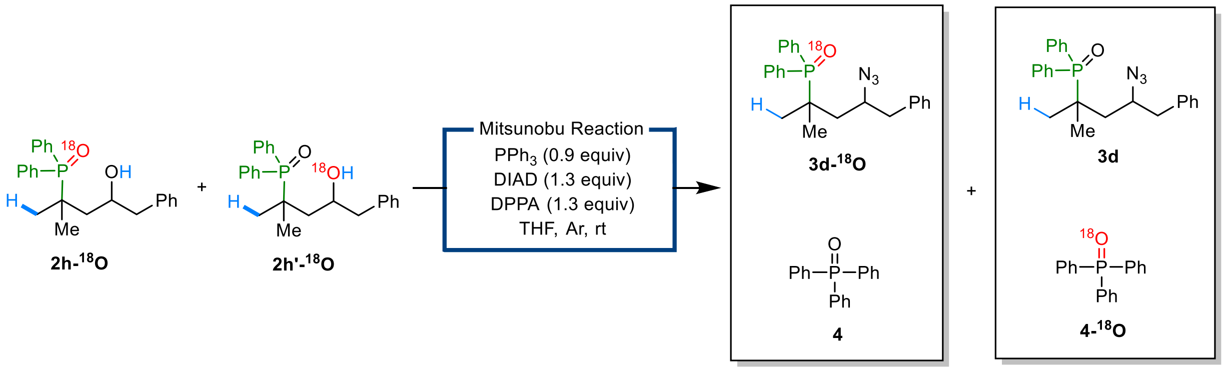

To a solution of **2h-^{18}O/2h'-^{18}O** mixture (0.2 mmol, 1.0 equiv.) in dry THF (2 mL) was added recrystallized PPh_3 (0.9 equiv.), DIAD (1.3 equiv.), and then DPPA (1.3 equiv.) was added dropwise at 0 °C under an argon atmosphere. The mixture was further stirred at room temperature until the starting material PPh_3

was consumed completely as monitored by TLC analysis. MS analysis with electron ionization (EI) gives a ratio of **4-^{18}O/4** = 1:20, which indicates that their parent molecular ratio is **2h-^{18}O/2h'-^{18}O** = 20:1.

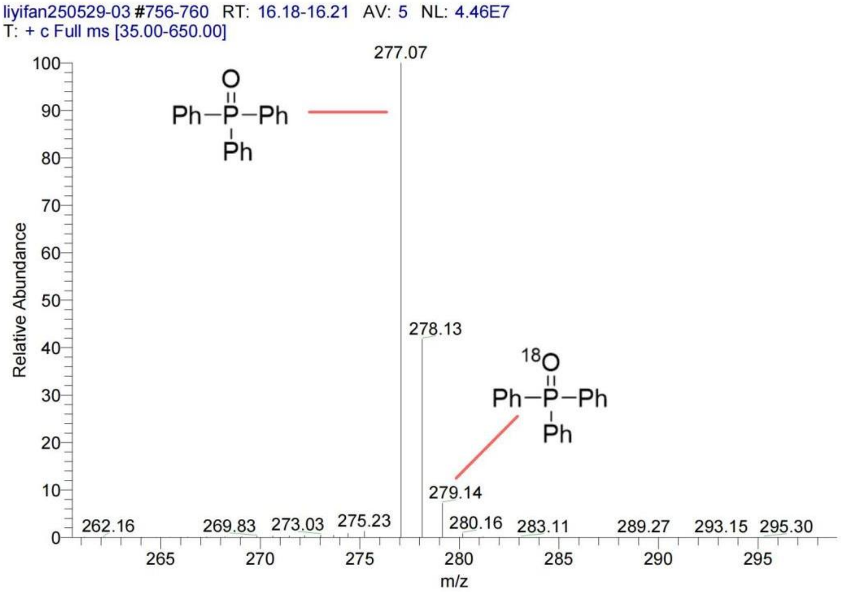

Figure S8. Mass spectrum of the mixture of **4-^{18}O** and **4** derived from **2ah-^{18}O and 2ah'-^{18}O**.

Determination method for the ^{18}O source of tertiary alcohol product

The compounds **2r-^{18}O and 2r'-^{18}O** were obtained by the general procedure in which $H_2{}^{18}O$ participates in the reaction instead of H_2O (75.9 mg, 0.22 mmol, 79% yield, **2r-^{18}O/2r'-^{18}O** = 6:10).

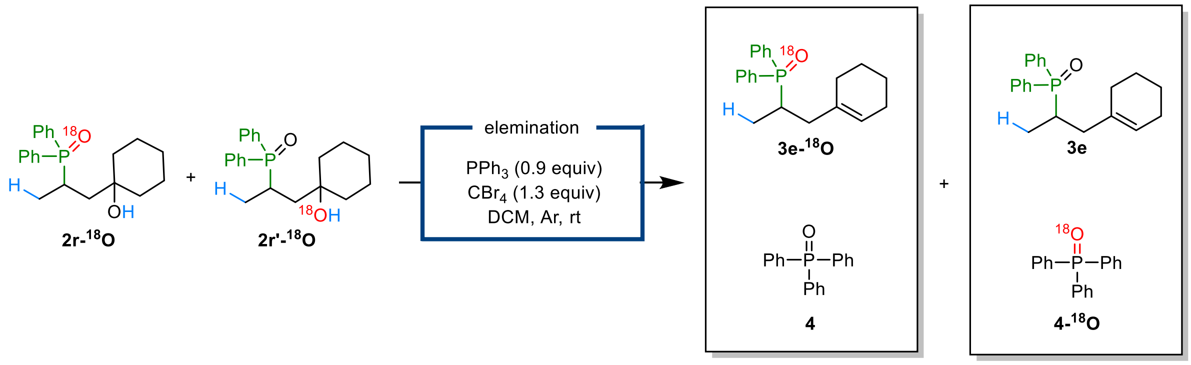

To a solution of **2r-^{18}O/2r'-^{18}O** mixture (0.2 mmol, 1.0 equiv.) in dry THF (2 mL) was added recrystallized PPh_3 (0.9 equiv.), DIAD (1.3 equiv.), and then DPPA (1.3 equiv.) was added dropwise at 0 °C under an argon atmosphere. The mixture was further stirred at room temperature until the starting material PPh_3 was consumed completely as monitored by TLC analysis. MS analysis with electron ionization (EI) gives a ratio of **4-^{18}O/4** = 10:6, which indicates that their parent molecular ratio is **2r-^{18}O/2r'-^{18}O** = 6:10.

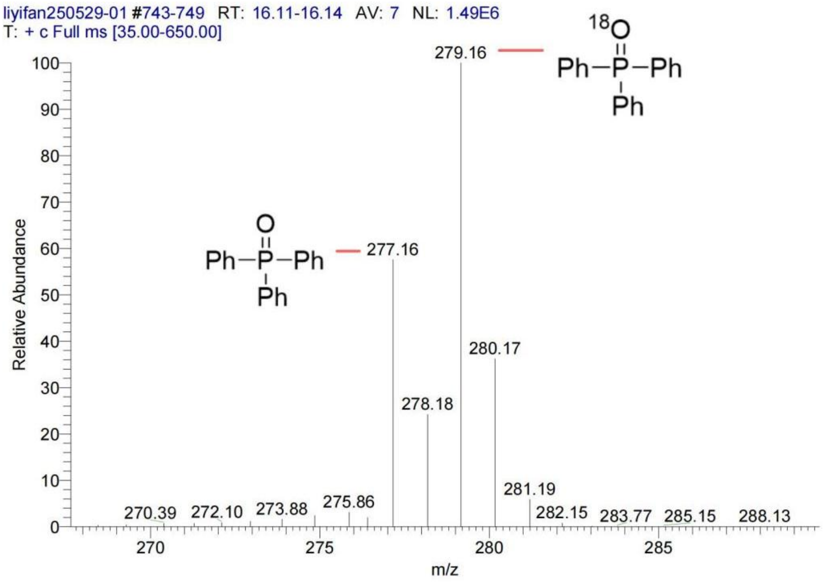

Figure S9. Mass spectrum of the mixture of **4-^{18}O** and **4** derived from **2r-^{18}O and 2r'-^{18}O**.

6.4 Crossover experiment

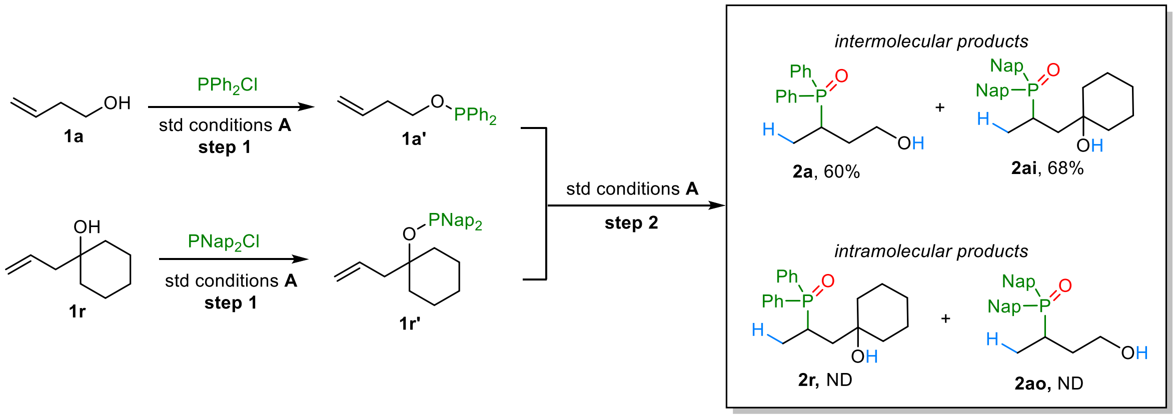

According to the first step of **General Procedure A**, but-3-en-1-ol **1a** and 1-allylcyclohexan-1-ol **1r** were converted to the corresponding intermediates **1a'** and **1r'**, respectively. Then in a glovebox, to an oven-dried 4 mL screw cap vial was added **1a'** (51.2 mg, 0.20 mmol, 0.50 equiv.), **1r'** (84.8 mg, 0.20 mmol, 0.50 equiv.), Then **Co-1** (5.0 mol%), **HPy-1** (20.0 mol%), $Ir(ppy)_3$ (1.0 mol%) were added. The flask was evacuated and back-filled with argon for 3 times. Subsequently, DMA (2 mL) and H_2O (0.3 mmol, 1.5 equiv.) was added under argon. The reaction mixture was allowed to stir at room temperature for 12 h under irradiation from 6 W blue LEDs (λ_{max} = 455 nm). After completion, the mixture was diluted with EtOAc. The combined organic layers were washed with brine, dried over $MgSO_4$, filtered and the solvents were removed under reduced pressure. At the end of the reaction, **2r** and **2ao** were not detected in crude ^{1}H NMR spectrum. The yields of **2a** and **2ai** were determined based on crude ^{1}H NMR spectrum with dibromomethane as an internal standard.

6.5 UV-Vis absorption spectroscopy

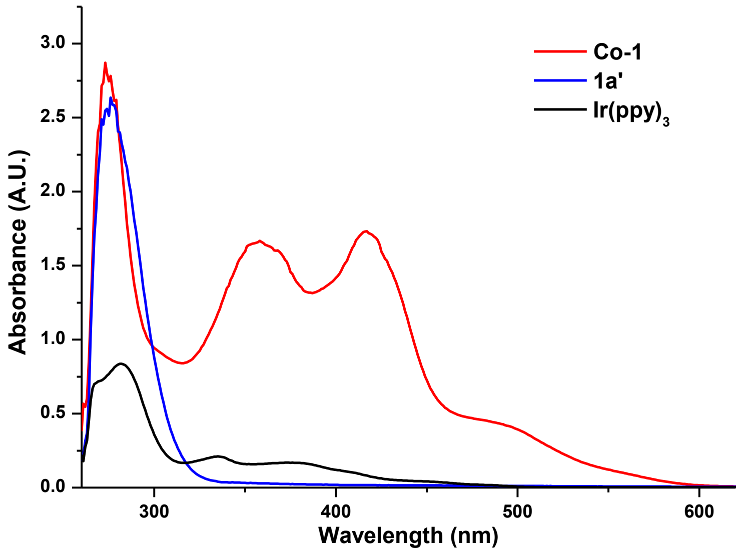

Figure S10. UV-Vis absorption spectra of photocatalyst $Ir(ppy)_3$ (0.05 mM), **Co-1** (0.25 mM), and **1a'** (5 mM) in deoxygenated DMA.

6.6 Cyclic voltammetry studies

Cyclic voltammetry (CV) experiments[1,2] were conducted in a 10 mL glass vial fitted with a glassy carbon working electrode (3 mm in diameter), an $Ag/AgNO_3$ reference electrode, and a platinum wire counter electrode. The electrolyte of $^{n}Bu_4NBF_4$ (0.1 M) in DMA (10.0 mL) was sparged with argon for 3-5 minutes before data collection. The scan rate is 100 mV/s. The measured cyclic voltammogram of **1a'** is shown in Figure S11. The measured the reduction potentials of **1a'** by cyclic voltammetry is greater than -2.0 V and the oxidation peak potentials of it is +1.09 V.

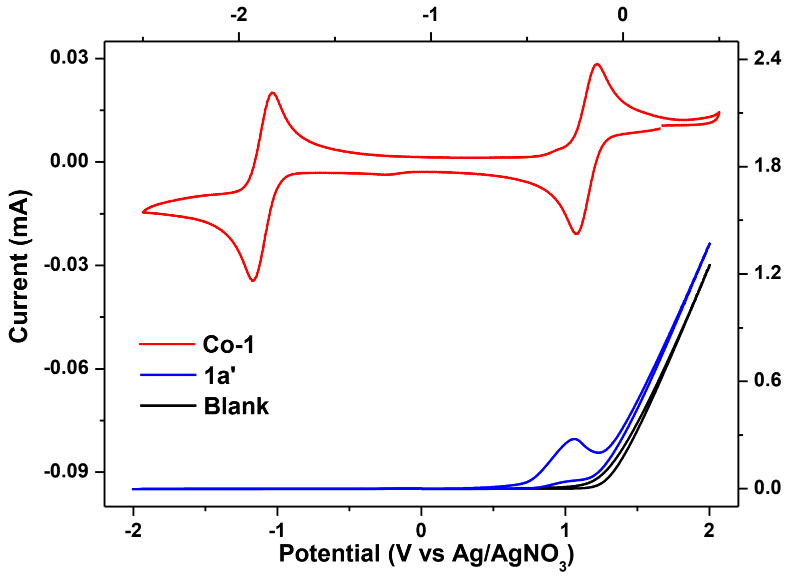

Figure S11. Cyclic voltammograms: **1a'** (10.0 mM).

6.7 Stern-Volmer quenching Experiments

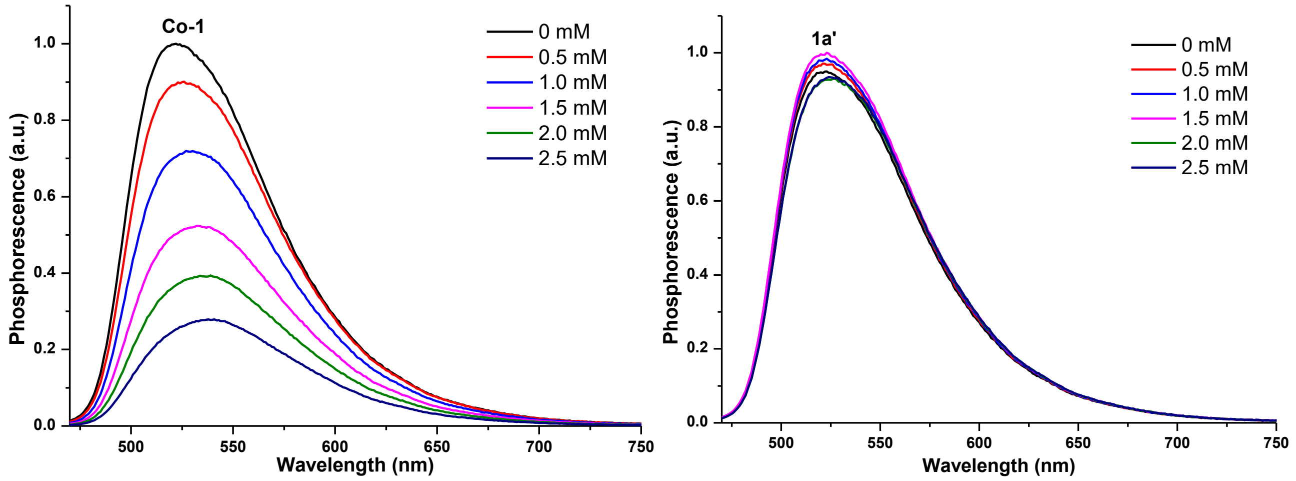

Figure S12. Fluorescence emission spectra of $Ir(ppy)_3$ (0.05 mM in DMA) with different concentration of **Co-1**, and **1a'**.

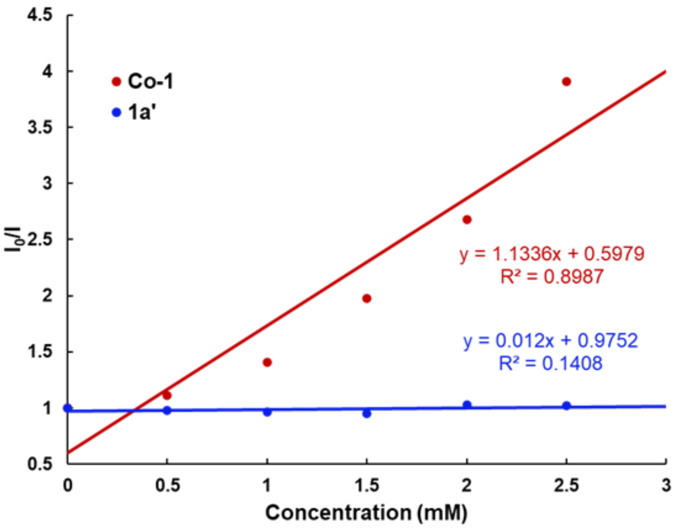

Figure S13. Stern-Volmer plot for the fluorescence quenching of $Ir(ppy)_3$ (0.05 mM in DMA) by **Co-1**, and **1a'**.

Stern-Volmer iuminescence quenching analysis[3] was conducted on a Jasco FP-8300 spectrofluorometer. The following parameters were employed: Excitation bandwidth = 5nm, data interval = 1 nm, scan speed = 2000 nm/min. The solution of $Ir(ppy)_3$ (0.05 mM in DMA) were excited at λ_{ex} = 455 nm and the emission was collected at 523 nm. The substrates **Co-1**, and **1a'** were dissolved in DMA (500 mM), respectively. For each quenching experiment, 3 μL of the stock solution were titrated to a solution (3 mL) of $Ir(ppy)_3$. The addition of 3 μL stock solution refers to an increase of the quencher concentration of 0.5 mM. I_0 is the luminescence intensity without the quencher, I is the intensity in the presence of the quencher.

7 X-Ray single-crystal diffraction study for 2k

Sigle crystal Preparation: Crystallization of **2k** was performed in a 5 mL glass vial dissolving 20 mg of compound in 2 mL solvent [80% CH_2Cl_2/petroleum ether (v/v)] and kept the vial in dark room for slow evaporation covered with cotton. After four days crystal was formed as colorless cubic shape.

Data collections and solution method: Data collections for crystal structure were performed at room temperature (149.92 K) using MoKα radiation on a Bruker APEXII diffractometer. Integration of the frames and data reduction was carried out using SAINT. The structure was solved by direct methods using SHELXS-97.

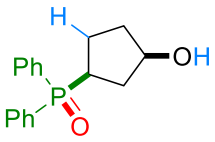

2k

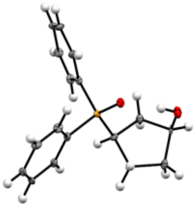

X-ray of **2k**
CCDC No. 2481386

Figure S14. ORTEP plot of compound **2k** with 30% ellipsoid probability.

Table S1. The crystal data and refinement results of compound **2k**.

Compound number	**2k**
CCDC number	2481386
Formula	$C_{17}H_{19}O_2P$
Formula weight	286.29
Temperature/K	149.92(11)
Crystal system	orthorhombic
Space group	$Pn2_1a$
a/Å	15.9893(2)
b/Å	15.6525(2)
c/Å	5.69580(10)
α/°	90
β/°	90
γ/°	90
Volume/Å^3	1425.50(4)
Z	4
ρ_{calc}g/cm^3	1.334
μ/mm^{-1}	1.692
F(000)	608.0
Crystal size/mm^3	0.09 × 0.08 × 0.07
Radiation	Cu Kα (λ = 1.54184)
2Θ range for data collection/°	12.432 to 151.538
Index ranges	-19 ≤ h ≤ 20, -19 ≤ k ≤ 13, -6 ≤ l ≤ 5
Goodness-of-fit on F^2	1.098
Final R indexes [I>=2σ (I)]	R_1 = 0.0309, wR_2 = 0.0846

8 Analytical data for products

(4-hydroxybutan-2-yl)diphenylphosphine oxide (2a)

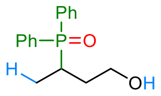

This compound was synthesized by **Standard conditions A**; Column chromatography conditions (EA), R_f = 0.10 (EA); White solid; 41 mg, 75%; ^{1}H NMR (400 MHz, $CDCl_3$): δ 7.83-7.75 (m, 4H), 7.48-7.42 (m, 6H), 4.80 (s, 1H), 3.75-3.63 (m, 2H), 2.81-2.78 (m, 1H), 1.97-1.87 (m, 1H), 1.69-1.68 (m, 1H), 1.16 (dd, J_1 =16.4 Hz, J_2 =6.8 Hz, 3H); ^{13}C NMR (100 MHz, $CDCl_3$): δ 131.5 (d, $J_{C\text{-}P}$ = 95.2 Hz), 131.44 (d, $J_{C\text{-}P}$ = 3.0 Hz), 131.41 (d, $J_{C\text{-}P}$ = 2.6 Hz), 130.8 (d, $J_{C\text{-}P}$ = 8.6 Hz), 128.5 (d, $J_{C\text{-}P}$ = 10.8 Hz), 128.4 (d, $J_{C\text{-}P}$ = 10.8 Hz), 58.4 (d, $J_{C\text{-}P}$ = 10.0 Hz), 32.0, 28.6 (d, $J_{C\text{-}P}$ = 72.0 Hz), 11.7; ^{31}P NMR (162 MHz, $CDCl_3$): δ 39.3; HRMS (ESI) m/z: $[M+H]^+$ Calcd for $C_{16}H_{20}O_2P^+$ 275.1195, Found 275.1184.

(4-hydroxy-2-methylbutan-2-yl)diphenylphosphine oxide (2b)

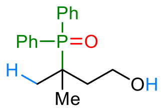

This compound was synthesized by **Standard conditions A**; Column chromatography conditions (EA), R_f = 0.10 (EA); White solid; 36 mg, 62%; ^{1}H NMR (400 MHz, $CDCl_3$): δ 8.00-7.96 (m, 4H), 7.55-7.47 (m, 6H), 5.35 (s, 1H), 3.76-3.73 (m, 2H), 1.82 (td, J_1 = 10.8 Hz, J_2 = 5.2 Hz, 2H), 1.28 (d, J = 15.2 Hz, 6H); ^{13}C NMR (100 MHz, $CDCl_3$): δ 132.3 (d, $J_{C\text{-}P}$ = 8.2 Hz), 131.8 (d, $J_{C\text{-}P}$ =2.7 Hz), 130.0 (d, $J_{C\text{-}P}$ = 91.5 Hz), 128.4 (d, $J_{C\text{-}P}$ = 11.0 Hz), 58.0 (d, $J_{C\text{-}P}$ = 3.0 Hz), 42.6, 37.1 (d, $J_{C\text{-}P}$ = 68.0 Hz), 23.8; ^{31}P NMR (162 MHz, $CDCl_3$): δ 41.7; HRMS (ESI) m/z: $[M+H]^+$ Calcd for $C_{17}H_{22}O_2P^+$ 289.1352, Found 289.1342.

(5-hydroxypentan-2-yl)diphenylphosphine oxide (2c)

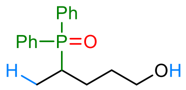

This compound was synthesized by **Standard conditions A**; Column chromatography conditions (EA), R_f = 0.10 (EA); White solid; 31 mg, 54%; ^{1}H NMR (400 MHz, $CDCl_3$): δ 7.77-7.75 (m, 4H), 7.44-7.42 (m, 6H), 4.41 (s, 1H) , 3.54 (s, 2H), 2.45-2.43 (m, 1H), 1.76 (s, 2H), 1.49 (s, 2H), 1.13 (dd, J_1 = 16.8 Hz, J_2 = 6.4 Hz, 3H); ^{13}C NMR (100 MHz, $CDCl_3$): δ131.7 (d, $J_{C\text{-}P}$ = 94.4 Hz), 131.6 (d, $J_{C\text{-}P}$ = 94.7 Hz), 131.4 (d, $J_{C\text{-}P}$ = 1.8 Hz), 131.3 (d, $J_{C\text{-}P}$ = 2.2 Hz), 130.8 (d, $J_{C\text{-}P}$ = 9.3 Hz), 128.4 (d, $J_{C\text{-}P}$ = 10.9 Hz), 128.3 (d, $J_{C\text{-}P}$ = 11.0 Hz), 61.5, 31.4 (d, $J_{C\text{-}P}$ = 71.0 Hz), 30.1 (d, $J_{C\text{-}P}$ = 12.0 Hz), 25.1, 11.9; ^{31}P NMR (162 MHz, $CDCl_3$): δ 38.3; HRMS (ESI) m/z: $[M+H]^+$ Calcd for $C_{17}H_{22}O_2P^+$ 289.1352, Found 289.1340.

(4-hydroxy-2-methyldecan-2-yl)diphenylphosphine oxide (2g)

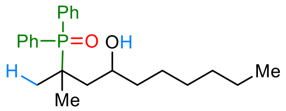

This compound was synthesized by **Standard conditions A**; Column chromatography conditions (PE/EA = 5:1 to 1:1), R_f = 0.15 (PE/EA = 1:1); White solid; 51 mg, 65%; ^{1}H NMR (400 MHz, $CDCl_3$): δ 8.02-7.94 (m, 4H), 7.57-7.48 (m, 6H), 6.02 (s, 1H), 3.86-3.84 (m, 1H), 1.92-1.83 (m, 1H), 1.50-1.40 (m, 3H), 1.38-1.25 (m, 14H), 0.88-0.84 (m, 3H); ^{13}C NMR (100 MHz, $CDCl_3$): δ 132.4 (d, J_{C-P} = 8.0 Hz), 132.3 (d, J_{C-P} = 8.0 Hz), 131.7 (d, J_{C-P} = 2.3 Hz), 130.1 (d, J_{C-P} = 90.3 Hz), 130.0 (d, J_{C-P} = 89.9 Hz), 128.4 (d, J_{C-P} = 10.8 Hz), 128.3 (d, J_{C-P} = 10.9 Hz), 66.6 (d, J_{C-P} = 2.0 Hz), 48.4, 38.3, 37.0 (d, J_{C-P} = 70.0 Hz), 31.8, 29.3, 26.7, 26.0, 22.6, 22.2, 14.1; ^{31}P NMR (162 MHz, $CDCl_3$): 42.7; HRMS (ESI) m/z: $[M+Na]^+$ Calcd for $C_{23}H_{33}NaO_2P^+$ 395.2110, Found 395.2098.

(4-hydroxy-2-methyl-5-phenylpentan-2-yl)diphenylphosphine oxide (2h)

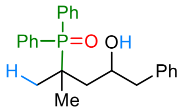

This compound was synthesized by **Standard conditions A**; Column chromatography conditions (PE/EA = 10:1 to 4:1), R_f = 0.15 (PE/EA = 3:1); White solid; 55 mg, 73%; ^{1}H NMR (400 MHz, $CDCl_3$): δ 7.99-7.90 (m, 4H), 7.53-7.47 (m, 6H), 7.27-7.15 (m, 5H), 6.00 (s, 1H), 4.16 (dd, J_1 =15.6 Hz, J_2 =7.2 Hz, 1H), 2.93 (dd, J_1 =13.6 Hz, J_2 = 6.0 Hz, 1H), 2.55 (dd, J_1 =13.6 Hz, J_2 = 7.2 Hz, 1H), 1.89-1.80 (m ,1H), 1.51 (dd, J_1 =24.4 Hz, J_2 =15.2 Hz, 1H), 1.23-1.16 (m ,6H); ^{13}C NMR (100 MHz, $CDCl_3$): δ 139.1, 132.4 (d, J_{C-P} = 8.1 Hz), 132.2 (d, J_{C-P} = 8.1 Hz), 131.7 (d, J_{C-P} = 1.5 Hz), 130.0 (d, J_{C-P} = 92.1 Hz), 129.9 (d, J_{C-P} = 90.4 Hz), 129.3, 128.4 (d, J_{C-P} = 11.0 Hz), 128.3 (d, J_{C-P} = 10.9 Hz), 128.2, 126.0, 68.2 (d, J = 2.0 Hz), 46.5, 44.4, 36.8 (d, J = 68.0 Hz), 26.2, 22.1; ^{31}P NMR (162 MHz, $CDCl_3$): 42.5; HRMS (ESI) m/z: $[M+H]^+$ Calcd for $C_{24}H_{28}O_2P^+$ 379.1821, Found 379.1810.

(4-cyclopentyl-4-hydroxy-2-methylbutan-2-yl)diphenylphosphine oxide (2i)

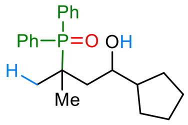

This compound was synthesized by **Standard conditions A**; Column chromatography conditions (PE/EA = 10:1 to 3:1), R_f = 0.10 (PE/EA = 2:1); White solid; 52 mg, 73%; ^{1}H NMR (400 MHz, $CDCl_3$): δ 8.02-7.94 (m, 4H), 7.55-7.48 (m, 6H), 5.93 (s, 1H), 3.65 (dd, J_1 =8.0 Hz, J_2 =8.0 Hz, 1H), 1.89-1.78 (m, 3H),

1.60-1.47 (m, 7H), 1.30-1.26 (m, 6H), 1.15-1.11 (m, 1H); ^{13}C NMR (100 MHz, $CDCl_3$): δ 132.4 (d, J_{C-P} = 8.1 Hz), 132.3 (d, J_{C-P} = 8.2 Hz), 131.75 (d, J_{C-P} = 2.3 Hz),131.72 (d, J_{C-P} = 2.6 Hz), 130.2 (d, J_{C-P} = 92.0 Hz), 130.0 (d, J_{C-P} = 90.2 Hz), 128.4 (d, J_{C-P} = 11.0 Hz), 128.3 (d, J_{C-P} = 10.8 Hz), 70.9, 47.2, 46.5, 36.9 (d, J_{C-P} = 68.0 Hz), 29.7, 29.0, 26.8, 26.75, 25.7, 22.4; ^{31}P NMR (162 MHz, $CDCl_3$): 42.7; HRMS (ESI) m/z: $[M+H]^+$ Calcd for $C_{22}H_{30}O_2P^+$ 357.1978, Found 357.1968.

(4-hydroxy-2-methyl-4-phenylbutan-2-yl)diphenylphosphine oxide (2j)

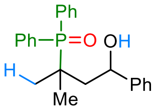

This compound was synthesized by **Standard conditions A**; Column chromatography conditions (PE/EA = 10:1 to 3:1), R_f = 0.10 (PE/EA = 3:1); White solid; 51 mg, 70%; ^{1}H NMR (400 MHz, $CDCl_3$): *δ* 8.07-7.97 (m, 4H), 7.59-7.50 (m, 6H), 7.33-7.26 (m, 4H), 7.21-7.17 (m, 1H), 6.51 (s, 1H), 4.99 (d, *J* =10.0 Hz, 1H), 2.22-2.13 (m, 1H), 1.68-1.58 (m, 1H), 1.43 (d, *J* = 14.8 Hz, 3H), 1.29 (d, *J* = 15.6 Hz, 3H); ^{13}C NMR (100 MHz, $CDCl_3$): *δ* 145.7, 132.5 (d, J_{C-P} = 8.0 Hz), 132.3 (d, J_{C-P} = 7.9 Hz), 131.91 (d, J_{C-P} = 2.9 Hz), 131.88 (d, J_{C-P} = 2.9 Hz), 129.9 (d, J_{C-P} = 92.3 Hz), 129.7 (d, J_{C-P} = 91.5 Hz), 128.4 (d, J_{C-P} = 10.2 Hz), 128.1, 126.8, 125.7, 69.8 (d, J_{C-P} = 1.0 Hz), 50.9, 37.4 (d, J_{C-P} = 68.0 Hz), 26.6, 22.0; ^{31}P NMR (162 MHz, $CDCl_3$): 42.5; HRMS (ESI) m/z: $[M+H]^+$ Calcd for $C_{23}H_{26}O_2P^+$ 365.1665, Found 365.1654.

((1S*,3R*)-3-hydroxycyclopentyl)diphenylphosphine oxide (2k)

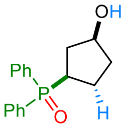

This compound was synthesized by **Standard conditions A**; Column chromatography conditions (PE/EA = 10:1 to 2:1), R_f = 0.10 (PE/EA = 2:1); White solid; 32 mg, 56%; ^{1}H NMR (400 MHz, $CDCl_3$): *δ* 7.83-7.76 (m, 4H), 7.55-7.45 (m, 6H), 5.00 (s, 1H), 4.30 (s, 1H), 3.04-2.98 (m, 1H), 2.16-1.80 (m, 5H), 1.79-1.72 (m, 1H); ^{13}C NMR (100 MHz, $CDCl_3$): *δ* 132.3 (d, J_{C-P} = 96.6 Hz), 131.72 (d, J_{C-P} = 2.7 Hz), 131.66 (d, J_{C-P} = 97.6 Hz), 131.65 (d, J_{C-P} = 2.7 Hz), 130.84 (d, J_{C-P} = 8.9 Hz), 130.75 (d, J_{C-P} = 9.0 Hz), 128.60 (d, J_{C-P} = 11.4 Hz), 128.59 (d, J_{C-P} = 11.6 Hz), 72.7 (d, J_{C-P} = 1.0 Hz), 36.50 (d, J_{C-P} = 5.0 Hz), 35.7 (d, J_{C-P} = 1.0 Hz), 34.7 (d, J_{C-P} = 71.0 Hz), 23.3 (d, J_{D-P} = 3.0 Hz); ^{31}P NMR (162 MHz, $CDCl_3$): 39.5; HRMS (ESI) m/z: $[M+H]^+$ Calcd for $C_{17}H_{20}O_2P^+$ 287.1195, Found 287.1186.

(4-hydroxy-4-methylpentan-2-yl)diphenylphosphine oxide (2l)

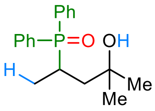

This compound was synthesized by **Standard conditions A**; Column chromatography conditions (PE/EA = 5:1 to 1:1), R_f = 0.10 (PE/EA = 1:1); White solid; 45 mg, 75%; ^{1}H NMR (400 MHz, $CDCl_3$): δ 7.83-7.78 (m, 4H), 7.55-7.44 (m, 6H), 3.73 (s, 1H), 2.86-2.80 (m, 1H), 1.99 (td, J_1 = 15.6 Hz, J_2 = 4.8 Hz, 1H), 1.59-1.51 (m, 1H), 1.26-1.18 (m, 9H); ^{13}C NMR (100 MHz, $CDCl_3$): δ 131.8 (d, $J_{C\text{-}P}$ = 8.5 Hz), 131.70 (d, $J_{C\text{-}P}$ = 2.4 Hz), 131.66 (d, $J_{C\text{-}P}$ = 95.4 Hz), 131.6 (d, $J_{C\text{-}P}$ = 2.6 Hz), 131.2 (d, $J_{C\text{-}P}$ = 8.7 Hz), 130.2 (d, $J_{C\text{-}P}$ = 94.1 Hz), 128.5 (d, $J_{C\text{-}P}$ = 11.4 Hz), 128.3 (d, $J_{C\text{-}P}$ = 11.1 Hz), 69.8 (d, $J_{C\text{-}P}$ = 8.0 Hz), 43.2, 30.6, 29.0, 28.5 (d, $J_{C\text{-}P}$ = 71.0 Hz), 16.2; ^{31}P NMR (162 MHz, CDCl3): 41.0; HRMS (ESI) m/z: $[M+H]^+$ Calcd for $C_{18}H_{24}O_2P^+$ 303.1508, Found 303.1500.

(4-benzyl-4-hydroxy-5-phenylpentan-2-yl)diphenylphosphine oxide (2m)

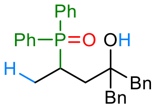

This compound was synthesized by **Standard conditions A**; Column chromatography conditions (PE/EA = 10:1 to 3:1), R_f = 0.10 (PE/EA = 3:1); White solid; 73 mg, 80%; ^{1}H NMR (400 MHz, $CDCl_3$): *δ* 7.56-7.46 (m, 4H), 7.40-7.25 (m, 14H), 7.20-7.19 (m, 2H), 4.85 (s, 1H), 2.90-2.82 (m, 3H), 2.72-2.61 (m, 2H), 2.06-1.96 (m, 1H), 1.41- 1.32 (m, 1H), 0.94 (dd, J_1 = 17.6 Hz, J_2 = 7.6 Hz, 3H); ^{13}C NMR (100 MHz, $CDCl_3$): *δ* 138.1, 137.6, 132.6 (d, $J_{C\text{-}P}$ = 8.5 Hz), 131.8 (d, $J_{C\text{-}P}$ = 2.4 Hz), 131.7 (d, $J_{C\text{-}P}$ = 2.6 Hz), 131.3 (d, $J_{C\text{-}P}$ = 9.0 Hz), 131.1 (d, $J_{C\text{-}P}$ = 96.2 Hz), 131.0, 130.8, 128.58 (d, $J_{C\text{-}P}$ = 96.6 Hz),128.54 (d, $J_{C\text{-}P}$ = 11.5 Hz), 128.01 (d, $J_{C\text{-}P}$ = 11.8 Hz), 127.98,127.95,126.3, 126.1 72.8 (d, $J_{C\text{-}P}$ = 3.0 Hz), 47.8, 47.6, 38.0, 26.9 (d, $J_{C\text{-}P}$ = 70.0 Hz), 16.9; ^{31}P NMR (162 MHz, $CDCl_3$): 43.0; HRMS (ESI) m/z: $[M+H]^+$ Calcd for $C_{30}H_{32}O_2P^+$ 455.2134, Found 455.2128.

(4-hydroxy-4-pentylnonan-2-yl)diphenylphosphine oxide (2n)

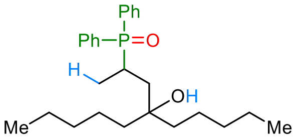

This compound was synthesized by **Standard conditions A**; Column chromatography conditions (PE/EA = 10:1 to 2:1), R_f = 0.10 (PE/EA = 2:1); White solid; 52 mg, 63%; ^{1}H NMR (400 MHz, $CDCl_3$): *δ* 7.83-7.78 (m, 4H), 7.56-7.45 (m, 6H), 3.11 (s, 1H), 2.81-2.79 (m, 1H), 1.96-1.87 (m, 1H), 1.57-1.45 (m, 3H), 1.35-1.06 (m, 17H), 0.90-0.85 (m, 6H); ^{13}C NMR (100 MHz, $CDCl_3$): *δ* 132.05 (d, $J_{C\text{-}P}$ = 9.3 Hz), 131.99 (d,

$J_{C\text{-}P}$ =96.2 Hz), 131.8 (d, $J_{C\text{-}P}$ =3.9 Hz), 131.3 (d, $J_{C\text{-}P}$ = 9.3 Hz), 130.4 (d, $J_{C\text{-}P}$ = 92.3 Hz), 128.6 (d, $J_{C\text{-}P}$ =10.6 Hz), 128.4 (d, $J_{C\text{-}P}$ =11.2 Hz), 73.3 (d, $J_{C\text{-}P}$ = 7.0 Hz), 40.0, 39.5, 38.7, 32.4, 27.8 (d, $J_{C\text{-}P}$ = 69.0 Hz), 23.3 (d, $J_{C\text{-}P}$ = 44.0 Hz), 22.6 (d, $J_{C\text{-}P}$ = 1.0 Hz), 16.6, 14.1; ^{31}P NMR (162 MHz, $CDCl_3$): 41.1; HRMS (ESI) m/z: $[M+H]^+$ Calcd for $C_{26}H_{40}O_2P^+$ 415.2760, Found 415.2760.

(4-hydroxy-4-methylnonan-2-yl)diphenylphosphine oxide (2o)

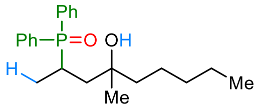

This compound was synthesized by **Standard conditions A**; Column chromatography conditions (PE/EA = 10:1 to 1:1), R_f = 0.10 (PE/EA = 1:1); White solid; 48 mg, 63%, dr = 1.1:1; ^{1}H NMR (400 MHz, $CDCl_3$): δ 7.83-7.78 (m, 4H), 7.56-7.46 (m, 6H), 2.88-2.75 (m, 2H), 2.03-1.86 (m, 1H), 1.63-1.42 (m, 3H), 1.39-1.19 (m, 11H), 1.09 (s, 1H), 0.89-0.85 (m, 3H) ^{13}C NMR (100 MHz, $CDCl_3$): δ 131.99 (d, $J_{C\text{-}P}$ = 8.0 Hz), 131.95 (d, $J_{C\text{-}P}$ = 8.0 Hz), 131.88 (d, $J_{C\text{-}P}$ = 95.0 Hz), 131.77 (d, $J_{C\text{-}P}$ = 2.0 Hz), 131.74 (d, $J_{C\text{-}P}$ = 2.0 Hz), 131.3 (d, $J_{C\text{-}P}$ = 9.0 Hz), 131.27 (d, $J_{C\text{-}P}$ = 9.0 Hz), 130.3 (d, $J_{C\text{-}P}$ = 95.0 Hz), 128.6 (d, $J_{C\text{-}P}$ = 11.0 Hz), 128.4 (d, $J_{C\text{-}P}$ = 12.0 Hz), 71.8 (d, $J_{C\text{-}P}$ = 7.0 Hz), 71.5 (d, $J_{C\text{-}P}$ = 6.0 Hz), 43.8, 41.9, 41.7, 41.6, 32.4, 28.3 (d, $J_{C\text{-}P}$ = 70.0 Hz), 28.0 (d, $J_{C\text{-}P}$ = 71.0 Hz), 27.6, 26.4, 23.8, 23.5, 22.6, 16.6, 16.3, 14.0; ^{31}P NMR (162 MHz, $CDCl_3$): 41.1, 41.0; HRMS (ESI) m/z: $[M+H]^+$ Calcd for $C_{22}H_{32}O_2P^+$ 359.2134, Found 359.2126.

(1-(1-hydroxycyclobutyl)propan-2-yl)diphenylphosphine oxide (2p)

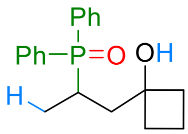

This compound was synthesized by **Standard conditions A**; Column chromatography conditions (PE/EA = 5:1 to 1:1), R_f = 0.15 (PE/EA = 1:1); White solid; 56 mg, 89%; ^{1}H NMR (400 MHz, $CDCl_3$): δ 7.82-7.77 (m, 4H), 7.56-7.45 (m, 6H), 4.29 (s, 1H), 2.84-2.73 (m, 1H), 2.12-2.00 (m, 4H), 1.90-1.69 (m, 3H), 1.47-1.36 (m, 1H), 1.26-1.20 (m, 3H); ^{13}C NMR (100 MHz, $CDCl_3$): δ 131.9 (d, $J_{C\text{-}P}$ = 8.5 Hz), 131.80 (d, $J_{C\text{-}P}$ = 2.3 Hz), 131.77 (d, $J_{C\text{-}P}$ = 2.5 Hz), 131.6 (d, $J_{C\text{-}P}$ = 95.7 Hz), 131.2 (d, $J_{C\text{-}P}$ = 8.8 Hz), 130.0 (d, $J_{C\text{-}P}$ = 94.6 Hz), 128.6 (d, $J_{C\text{-}P}$ = 11.4 Hz), 128.4 (d, $J_{C\text{-}P}$ = 11.2 Hz), 74.3 (d, $J_{C\text{-}P}$ = 8.0 Hz), 39.3, 37.0, 36.1, 29.3 (d, $J_{C\text{-}P}$ = 70.0 Hz), 15.7, 12.5; ^{31}P NMR (162 MHz, $CDCl_3$): 40.8; HRMS (ESI) m/z: $[M+H]^+$ Calcd for $C_{19}H_{24}O_2P^+$ 315.1508, Found 315.1500.

(1-(1-hydroxycyclopentyl)propan-2-yl)diphenylphosphine oxide (2q)

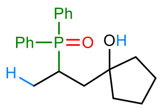

This compound was synthesized by **Standard conditions A**; Column chromatography conditions (PE/EA = 5:1 to 1:1), R_f = 0.10 (PE/EA = 1:1); White solid; 54 mg, 82%; ^{1}H NMR (400 MHz, $CDCl_3$): δ 7.83-7.78 (m, 4H), 7.55-7.48 (m, 6H), 3.20 (s, 1H), 2.88-2.84 (m, 1H), 2.17-2.08 (m, 1H), 1.80-1.76 (m, 3H), 1.69-1.53 (m, 5H), 1.49-1.38 (m, 1H), 1.27-1.20 (m, 3H); ^{13}C NMR (100 MHz, $CDCl_3$): 131.92 (d, J_{C-P} = 96.4 Hz), δ 131.87 (d, J_{C-P} = 8.9 Hz), 131.73 (d, J_{C-P} = 2.1 Hz), 131.69 (d, J_{C-P} = 1.7 Hz), 131.2 (d, J_{C-P} = 8.7 Hz), 130.3 (d, J_{C-P} = 94.1 Hz), 128.6 (d, J_{C-P} = 11.1 Hz), 128.4 (d, J_{C-P} = 11.1 Hz), 81.3 (d, J_{C-P} = 8.0 Hz), 41.5, 41.1, 39.5, 29.7 (d, J_{C-P} = 70.0 Hz), 23.7, 23.2, 16.0; ^{31}P NMR (162 MHz, $CDCl_3$): 40.7; HRMS (ESI) m/z: $[M+H]^+$ Calcd for $C_{20}H_{26}O_2P^+$ 329.1665, Found 329.1656**.**

(1-(1-hydroxycyclohexyl)propan-2-yl)diphenylphosphine oxide (2r)

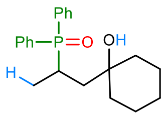

This compound was synthesized by **Standard conditions A**; Column chromatography conditions (PE/EA = 5:1 to 1:1), R_f = 0.15 (PE/EA = 1:1); White solid; 55 mg, 80%; ^{1}H NMR (400 MHz, $CDCl_3$): δ 7.84-7.78 (m, 4H), 7.55-7.45 (m, 6H), 3.16 (s, 1H), 2.88-2.82 (m, 1H), 1.93-1.84 (m, 1H), 1.70-1.50 (m, 6H), 1.43-1.32 (m, 3H), 1.29-1.19 (m, 5H); ^{13}C NMR (100 MHz, $CDCl_3$): δ 131.94 (d, J_{C-P} = 95.6 Hz), 131.85 (d, J_{C-P} = 8.3 Hz), 131.7 (d, J_{C-P} = 2.1 Hz), 131.6 (d, J_{C-P} = 2.1 Hz), 131.3 (d, J_{C-P} = 8.7 Hz), 130.5 (d, J_{C-P} = 94.3 Hz), 128.5 (d, J_{C-P} = 11.2 Hz), 128.4 (d, J_{C-P} = 11.1 Hz), 70.4 (d, J_{C-P} = 7.0 Hz), 42.3, 38.9, 37.1, 27.5 (d, J_{C-P} = 71.0 Hz), 25.7, 22.2, 22.1 16.4; ^{31}P NMR (162 MHz, $CDCl_3$): 40.7; HRMS (ESI) m/z: $[M+H]^+$ Calcd for $C_{21}H_{28}O_2P^+$ 343.1821, Found 343.1813**.**

(1-(1-hydroxycycloheptyl)propan-2-yl)diphenylphosphine oxide (2s)

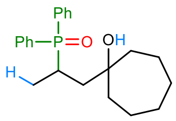

This compound was synthesized by **Standard conditions A**; Column chromatography conditions (PE/EA = 5:1 to 1:1), R_f = 0.15 (PE/EA = 1:1); White solid; 37 mg, 52%; ^{1}H NMR (400 MHz, $CDCl_3$): δ 7.82-7.78 (m, 4H), 7.56-7.47 (m, 6H), 3.25 (s, 1H), 2.88-2.77 (m, 1H), 1.99-1.86 (m, 2H), 1.80-1.51 (m, 9H), 1.45-1.18 (m, 6H); ^{13}C NMR (100 MHz, $CDCl_3$): δ 132.1 (d, J_{C-P} = 8.0 Hz), 131.9 (d, J_{C-P} = 95.0 Hz), 131.8 (d, J_{C-P} = 3.0 Hz), 131.7 (d, J_{C-P} = 3.0 Hz), 131.3 (d, J_{C-P} = 8.0 Hz), 130.1 (d, J_{C-P} = 93.0 Hz), 128.6 (d, J_{C-P} = 11.0 Hz), 128.4 (d, J_{C-P} = 10.0 Hz), 74.5 (d, J_{C-P} = 7.0 Hz), 43.0, 42.4, 40.6, 30.02, 30.01, 28.0 (d, J_{C-P} =

70.0 Hz), 22.3, 22.2, 16.8; ^{31}P NMR (162 MHz, $CDCl_3$): 41.1; HRMS (ESI) m/z: $[M+H]^+$ Calcd for $C_{22}H_{30}O_2P^+$ 357.1978, Found 357.1970.

(1-(1-hydroxycyclooctyl)propan-2-yl)diphenylphosphine oxide (2t)

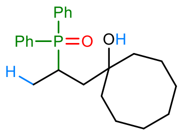

This compound was synthesized by **Standard conditions A**; Column chromatography conditions (PE/EA = 5:1 to 1:1), R_f = 0.15 (PE/EA = 1:1); White solid; 41 mg, 56%; ^{1}H NMR (400 MHz, $CDCl_3$): δ 7.83-7.79 (m, 4H), 7.53-7.46 (m, 6H), 2.97 (s, 1H), 2.89-2.78 (m, 1H), 1.92-1.81 (m, 2H), 1.72-1.58 (m, 8H), 1.43-1.19 (m, 9H); ^{13}C NMR (100 MHz, $CDCl_3$): δ 132.0 (d, J_{C-P} = 95.5 Hz), 131.9 (d, J_{C-P} = 8.4 Hz), 131.7 (d, J_{C-P} = 2.7 Hz), 131.6 (d, J_{C-P} = 2.5 Hz), 131.3 (d, J_{C-P} = 8.7 Hz), 130.5 (d, J_{C-P} = 93.8 Hz), 128.5 (d, J_{C-P} = 11.2 Hz), 128.4 (d, J_{C-P} = 11.1 Hz), 74.0 (d, J_{C-P} = 7.0 Hz), 41.1, 37.7, 35.0, 28.3, 27.9, 27.8 (d, J_{C-P} = 71.0 Hz), 24.9, 22.4, 22.0, 16.4; ^{31}P NMR (162 MHz, $CDCl_3$): 41.0; HRMS (ESI) m/z: $[M+H]^+$ Calcd for $C_{23}H_{32}O_2P^+$ 371.2134, Found 371.2129.

(1-(1-hydroxycyclododecyl)propan-2-yl)diphenylphosphine oxide (2u)

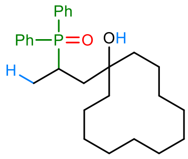

This compound was synthesized by **Standard conditions A**; Column chromatography conditions (PE/EA = 5:1 to 1:1), R_f = 0.15 (PE/EA = 1:1); White solid; 49 mg, 58%; ^{1}H NMR (400 MHz, $CDCl_3$): δ 7.83-7.77 (m, 4H), 7.57-7.45 (m, 6H), 3.56 (s, 1H), 2.91-2.79 (m, 1H), 1.86 (td, J_1 = 15.6 Hz, J_2 = 5.6 Hz, 1H), 1.68-1.18 (m, 26H); ^{13}C NMR (100 MHz, $CDCl_3$): δ 132.2 (d, J_{C-P} = 8.4 Hz), 131.9 (d, J_{C-P} = 96.1 Hz), 131.78 (d, J_{C-P} = 2.8 Hz), 131.75 (d, J_{C-P} = 2.7 Hz), 131.4 (d, J_{C-P} = 8.8 Hz), 129.8 (d, J_{C-P} = 93.6 Hz), 128.6 (d, J_{C-P} = 11.2 Hz), 128.4 (d, J_{C-P} = 11.2 Hz), 74.0 (d, J_{C-P} = 6.0 Hz), 41.1, 36.0, 33.9, 28.0 (d, J_{C-P} = 70.0 Hz), 26.52, 26.45, 25.9, 22.6, 22.5, 22.12, 22.06, 19.8, 19.4, 16.9; ^{31}P NMR (162 MHz, $CDCl_3$): 41.4; HRMS (ESI) m/z: $[M+H]^+$ Calcd for $C_{27}H_{40}O_2P^+$ 427.2760, Found 427.2749.

(1-(1-hydroxycyclohexyl)-2-methylpropan-2-yl)diphenylphosphine oxide (2v)

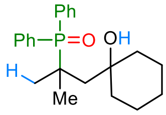

This compound was synthesized by **Standard conditions A**; Column chromatography conditions (PE/EA = 5:1 to 1:1), R_f = 0.15 (PE/EA = 1:1); White solid; 46 mg, 65%; ^{1}H NMR (400 MHz, $CDCl_3$): δ 7.99-7.94 (m, 4H), 7.53-7.46 (m, 6H), 5.48 (s, 1H), 1.86-1.78 (m, 4H), 1.73-1.63 (m, 2H), 1.58-1.53 (m, 1H), 1.43-1.29 (m, 10H), 1.20-1.12 (m, 1H); ^{13}C NMR (100 MHz, $CDCl_3$): δ 132.4 (d, $J_{C\text{-}P}$ = 8.0 Hz), 132.1 (d, $J_{C\text{-}P}$ = 95.4 Hz)131.6 (d, $J_{C\text{-}P}$ = 2.2 Hz), 130.6 (d, $J_{C\text{-}P}$ = 90.4 Hz), 128.3 (d, $J_{C\text{-}P}$ = 10.5 Hz), 70.6 (d, $J_{C\text{-}P}$ = 3.0 Hz), 53.2, 39.8, 38.5 (d, $J_{C\text{-}P}$ = 67.0 Hz), 26.4, 25.9, 22.3; ^{31}P NMR (162 MHz, $CDCl_3$): 43.5; HRMS (ESI) m/z: $[M+H]^+$ Calcd for $C_{22}H_{30}O_2P^+$ 357.1978, Found 357.1969.

(1-(1-hydroxy-4,4-dimethylcyclohexyl)propan-2-yl)diphenylphosphine oxide (2w)

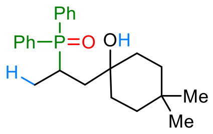

This compound was synthesized by **Standard conditions A**; Column chromatography conditions (PE/EA = 5:1 to 1:1), R_f = 0.20 (PE/EA = 1:1); White solid; 58 mg, 78%; ^{1}H NMR (400 MHz, $CDCl_3$): δ 7.75-7.71 (m, 4H), 7.47-7.37 (m, 6H), 3.09 (s, 1H), 2.82-2.74 (m, 1H), 1.83 (td, J_1 = 15.6 Hz, J_2 = 4.8 Hz, 1H), 1.58-1.24 (m, 7H), 1.18-1.12 (m, 3H), 1.08-0.96 (m, 2H), 0.83 (s, 3H), 0.74 (s, 3H); ^{13}C NMR (100 MHz, $CDCl_3$): δ 131.92 (d, $J_{C\text{-}P}$ = 95.7 Hz), 131.88 (d, $J_{C\text{-}P}$ = 8.3 Hz), 131.7 (d, $J_{C\text{-}P}$ = 2.6 Hz), 131.6 (d, $J_{C\text{-}P}$ = 2.1 Hz), 131.3 (d, $J_{C\text{-}P}$ = 8.5 Hz), 130.4 (d, $J_{C\text{-}P}$ = 93.3 Hz), 128.5 (d, $J_{C\text{-}P}$ = 11.6 Hz), 128.4 (d, $J_{C\text{-}P}$ = 11.7 Hz), 70.2 (d, $J_{C\text{-}P}$ = 8.0 Hz), 42.2, 34.9, 34.8, 33.2, 30.4, 29.6, 27.7 (d, $J_{C\text{-}P}$ = 71.0 Hz), 25.8, 16.4; ^{31}P NMR (162 MHz, $CDCl_3$): 40.9; HRMS (ESI) m/z: $[M+H]^+$ Calcd for $C_{23}H_{32}O_2P^+$ 371.2134, Found 371.2127.

(1-(4,4-difluoro-1-hydroxycyclohexyl)propan-2-yl)diphenylphosphine oxide (2x)

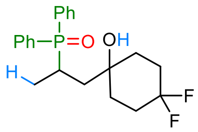

This compound was synthesized by **Standard conditions A**; Column chromatography conditions (PE/EA = 5:1 to 1:1), R_f = 0.15 (PE/EA = 1:1); White solid; 58 mg, 77%; ^{1}H NMR (400 MHz, $CDCl_3$): δ 7.80-7.74 (m, 4H), 7.61-7.48 (m, 6H), 4.86 (s, 1H), 2.94-2.86 (m, 1H), 2.28-2.12 (m, 2H), 2.04-1.84 (m, 4H), 1.73-1.69 (m, 1H), 1.61-1.49 (m, 3H), 1.16 (dd, J_1 = 17.6 Hz, J_2 = 7.2 Hz, 3H); ^{13}C NMR (100 MHz, $CDCl_3$): δ 132.5 (d, $J_{C\text{-}P}$ = 8.5 Hz), 132.2 (d, $J_{C\text{-}P}$ = 5.1 Hz), 131.23 (d, $J_{C\text{-}P}$ = 97.0 Hz), 131.22 (d, $J_{C\text{-}P}$ = 8.8 Hz), 128.8 (d, $J_{C\text{-}P}$ = 11.3 Hz), 128.5 (d, $J_{C\text{-}P}$ = 11.2 Hz), 128.42 (d, $J_{C\text{-}P}$ = 88.8 Hz), 124.0 (t, $J_{C\text{-}F}$ = 237.0 Hz), 67.9, 44.0, 35.6 (d, $J_{C\text{-}P}$ = 9.0 Hz), 32.8 (d, $J_{C\text{-}P}$ = 9.0 Hz), 29.6 (t, $J_{C\text{-}F}$ = 24.0 Hz), 29.5 (t, $J_{C\text{-}F}$ = 24.0 Hz),

28.8, 28.1, 17.3; ^{19}F NMR (376 MHz, $CDCl_3$) δ -92.33 (d, J = 233.9, 1F), -104.19 (d, J = 233.1, 1F); ^{31}P NMR (162 MHz, $CDCl_3$): 42.6; HRMS (ESI) m/z: $[M+H]^+$ Calcd for $C_{21}H_{26}F_2O_2P^+$ 379.1633, Found 379.1624.

(1-(8-hydroxy-1,4-dioxaspiro[4.5]decan-8-yl)propan-2-yl)diphenylphosphine oxide (2y)

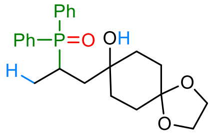

This compound was synthesized by **Standard conditions A**; Column chromatography conditions (PE/EA = 5:1 to 1:1), R_f = 0.15 (PE/EA = 1:1); White solid; 42 mg, 52%; ^1H NMR (400 MHz, $CDCl_3$): δ 7.81-7.76 (m, 4H), 7.56-7.46 (m, 6H), 3.98-3.90 (m, 5H), 2.94-2.82 (m, 1H), 2.01-1.89 (m, 4H), 1.67-1.50 (m, 6H), 1.18 (dd, J_1 = 17.6 Hz, J_2 = 7.6 Hz, 3H); ^{13}C NMR (100 MHz, $CDCl_3$): δ 132.2 (d, $J_{C\text{-}P}$ = 8.5 Hz), 131.93 (d, $J_{C\text{-}P}$ = 2.4 Hz), 131.86 (d, $J_{C\text{-}P}$ = 2.5 Hz), 131.7 (d, $J_{C\text{-}P}$ = 96.2 Hz), 131.1 (d, $J_{C\text{-}P}$ = 8.7 Hz), 129.6 (d, $J_{C\text{-}P}$ = 94.2 Hz), 128.7 (d, $J_{C\text{-}P}$ = 11.1 Hz), 128.4 (d, $J_{C\text{-}P}$ = 11.3 Hz), 109.0, 69.0 (d, $J_{C\text{-}P}$ = 6.0 Hz), 64.2, 64.1, 43.5, 36.7, 34.0, 30.5, 30.4, 28.2 (d, $J_{C\text{-}P}$ = 70.0 Hz), 16.9; ^{31}P NMR (162 MHz, $CDCl_3$): 41.7; HRMS (ESI) m/z: $[M+H]^+$ Calcd for $C_{23}H_{30}O_4P^+$ 401.1876, Found 401.1868.

(1-(2-hydroxy-2,3-dihydro-1H-inden-2-yl)propan-2-yl)diphenylphosphine oxide (2z)

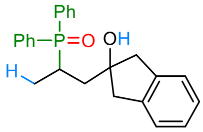

This compound was synthesized by **Standard conditions A**; Column chromatography conditions (PE/EA = 10:1 to 2:1), R_f = 0.15 (PE/EA = 2:1); White solid; 50 mg, 66%; ^1H NMR (400 MHz, $CDCl_3$): δ 7.84-7.79 (m, 4H), 7.54-7.47 (m, 6H), 7.18-7.12 (m, 4H), 4.11 (s, 1H), 3.09-2.84 (m, 5H), 2.26 (td, J_2 = 14.8 Hz, J_2 = 4.4 Hz, 1H), 1.90-1.81 (m, 1H), 1.27 (dd, J_1 = 17.6 Hz, J_2 = 7.2 Hz, 3H); ^{13}C NMR (100 MHz, $CDCl_3$): δ 131.87 (d, $J_{C\text{-}P}$ = 8.3 Hz), 131.85 (d, $J_{C\text{-}P}$ = 3.7 Hz), 131.81 (d, $J_{C\text{-}P}$ = 4.1 Hz), 131.5 (d, $J_{C\text{-}P}$ = 96.9 Hz), 131.2 (d, $J_{C\text{-}P}$ = 8.7 Hz), 130.1 (d, $J_{C\text{-}P}$ = 95.2 Hz), 128.7 (d, $J_{C\text{-}P}$ = 11.0 Hz), 128.4 (d, $J_{C\text{-}P}$ = 10.9 Hz), 81.4 (d, $J_{C\text{-}P}$ = 8.0 Hz), 48.3, 46.7, 40.6, 29.0 (d, $J_{C\text{-}P}$ = 71.0 Hz), 15.8; ^{31}P NMR (162 MHz, $CDCl_3$): 40.9; HRMS (ESI) m/z: $[M+H]^+$ Calcd for $C_{24}H_{26}O_2P^+$ 377.1665, Found 377.1657.

(1-(4-hydroxytetrahydro-2H-pyran-4-yl)propan-2-yl)diphenylphosphine oxide (2aa)

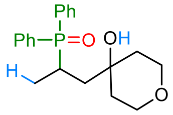

This compound was synthesized by **Standard conditions A**; Column chromatography conditions (PE/EA = 5:1 to EA), R_f = 0.10 (EA); White solid; 38 mg, 55%; ^{1}H NMR (400 MHz, $CDCl_3$): δ 7.81-7.75 (m, 4H), 7.59-7.48 (m, 6H), 4.69 (s, 1H), 3.85-3.65 (m, 4H), 2.93-2.89 (m, 1H), 1.96 (td, J_1 = 15.2 Hz, J_2 = 6.4 Hz, 1H), 1.74 (d, J = 12.8 Hz,1H), 1.65-1.51 (m, 4H), 1.20 (dd, J_1 = 17.6 Hz, J_2 = 7.6 Hz, 3H); ^{13}C NMR (100 MHz, $CDCl_3$): δ 132.2 (d, J_{C-P} = 8.3 Hz), 132.0 (d, J_{C-P} = 2.2 Hz), 131.9 (d, J_{C-P} = 1.9 Hz), 131.5 (d, J_{C-P} = 114.6 Hz), 131.2 (d, J_{C-P} = 8.8 Hz), 129.0 (d, J_{C-P} = 119.3 Hz), 128.7 (d, J_{C-P} = 11.4 Hz), 128.4 (d, J_{C-P} = 11.0 Hz), 67.3 (d, J_{C-P} = 5.0 Hz), 63.8, 63.7, 44.3, 39.1, 37.2, 27.6 (d, J_{C-P} = 71.0 Hz), 17.0; ^{31}P NMR (162 MHz, $CDCl_3$): 41.9; HRMS (ESI) m/z: $[M+H]^+$ Calcd for $C_{20}H_{26}O_3P^+$ 345.1614, Found 345.1607.

tert-butyl-4-(2-(diphenylphosphoryl)propyl)-4-hydroxypiperidine-1-carboxylate (2ab)

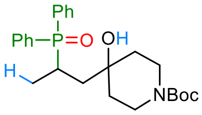

This compound was synthesized by **Standard conditions A**; Column chromatography conditions (PE/EA = 5:1 to EA), R_f = 0.10 (EA); White solid; 44 mg, 50%; ^{1}H NMR (400 MHz, $CDCl_3$): δ 7.80-7.75 (m, 4H), 7.59-7.48 (m, 6H), 4.75 (s, 1H), 3.78 (s, 2H), 3.18 (s, 2H), 2.94-2.86 (m, 1H), 1.96 (td, J_1 = 15.2 Hz, J_2 = 7.2 Hz, 1H), 1.79 (d, J = 12.8 Hz,1H), 1.60-1.51 (m, 2H), 1.45-1.33 (m, 11H), 1.17 (dd, J_1 = 17.2 Hz, J_2 = 7.2 Hz, 3H); ^{13}C NMR (100 MHz, $CDCl_3$): δ 154.8, 132.2 (d, J_{C-P} = 8.6 Hz), 132.0 (d, J_{C-P} = 2.3 Hz), 131.9 (d, J_{C-P} = 2.3 Hz), 131.4 (d, J_{C-P} = 96.5 Hz), 131.2 (d, J_{C-P} = 8.6 Hz), 129.1 (d, J_{C-P} = 98.3 Hz), 128.7 (d, J_{C-P} = 11.7 Hz), 128.4 (d, J_{C-P} = 11.2 Hz), 79.1, 68.0 (d, J_{C-P} = 5.0 Hz), 44.0, 40.0, 39.4, 38.3, 36.3, 28.4, 27.8 (d, J_{C-P} = 70.0 Hz), 17.0; ^{31}P NMR (162 MHz, $CDCl_3$): 42.1; HRMS (ESI) m/z: $[M+H]^+$ Calcd for $C_{25}H_{35}NO_4P^+$ 444.2298, Found 444.2297.

(1-hydroxyhexan-3-yl)diphenylphosphine oxide (2ac)

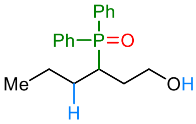

This compound was synthesized by **Standard conditions A**; Column chromatography conditions (PE/EA = 5:1 to EA), R_f = 0.10 (EA); White solid; 29 mg, 48%; ^{1}H NMR (400 MHz, $CDCl_3$): δ 7.83-7.79 (m, 4H), 7.52-7.44 (m, 6H), 4.79 (s, 1H), 3.79-3.74 (m, 1H), 3.61-3.55 (m, 1H), 2.58-2.54 (m, 1H), 2.01-1.82 (m, 2H), 1.64-1.44 (m, 3H), 1.26-1.17 (m, 1H), 0.82 (t, J = 7.2 Hz, 3H); ^{13}C NMR (100 MHz, $CDCl_3$): δ 131.9 (d, J_{C-P} = 94.8 Hz), 131.61 (d, J_{C-P} = 2.7 Hz), 131.58 (d, J_{C-P} = 2.9 Hz), 131.11 (d, J_{C-P} = 91.9 Hz), 131.05 (d, J_{C-P} = 8.6 Hz), 130.9 (d, J_{C-P} = 8.6 Hz), 128.6 (d, J_{C-P} = 11.4 Hz), 128.5 (d, J_{C-P} = 11.2 Hz), 58.7 (d, J_{C-P}

= 5.0 Hz), 35.0 (d, $J_{C\text{-}P}$ = 69.0 Hz), 29.8, 28.2, 20.7 (d, $J_{C\text{-}P}$ = 12.0 Hz), 13.7; ^{31}P NMR (162 MHz, $CDCl_3$): 39.7; HRMS (ESI) m/z: $[M+H]^+$ Calcd for $C_{18}H_{24}O_2P^+$ 303.1508, Found 303.1500.

(6-hydroxyhexan-3-yl)diphenylphosphine oxide (2ac')

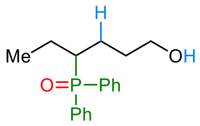

This compound was synthesized by **Standard conditions A**; Column chromatography conditions (PE/EA = 5:1 to EA), R_f = 0.15 (EA); White solid; 6 mg, 10%; ^{1}H NMR (400 MHz, $CDCl_3$): δ 7.81-7.76 (m, 4H), 7.50-7.44 (m, 6H), 3.55 (t, J = 6.0 Hz, 2H), 2.86 (s, 1H), 2.30-2.22 (m, 1H), 1.87-1.49 (m, 6H), 0.95 (t, J = 7.2 Hz, 3H); ^{13}C NMR (100 MHz, $CDCl_3$): δ 132.7 (d, $J_{C\text{-}P}$ = 93.8 Hz), 132.1 (d, $J_{C\text{-}P}$ = 93.7 Hz), 131.52 (d, $J_{C\text{-}P}$ = 3.0 Hz), 131.48 (d, $J_{C\text{-}P}$ = 3.9 Hz), 131.1 (d, $J_{C\text{-}P}$ = 8.6 Hz), 130.9 (d, $J_{C\text{-}P}$ = 8.6 Hz), 128.6 (d, $J_{C\text{-}P}$ = 10.6 Hz), 128.5 (d, $J_{C\text{-}P}$ = 10.2 Hz), 62.1, 38.3 (d, $J_{C\text{-}P}$ = 70.0 Hz), 30.9 (d, $J_{C\text{-}P}$ = 7.0 Hz), 23.2, 20.9, 12.5 (d, $J_{C\text{-}P}$ = 10.6 Hz); ^{31}P NMR (162 MHz, $CDCl_3$): 37.6; HRMS (ESI) m/z: $[M+H]^+$ Calcd for $C_{18}H_{24}O_2P^+$ 303.1508, Found 303.1501.

(2-hydroxy-2-methyloctan-4-yl)diphenylphosphine oxide (2ad)

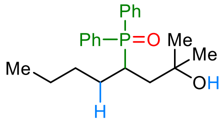

This compound was synthesized by **Standard conditions A**; Column chromatography conditions (PE/EA = 5:1 to 1:1), R_f = 0.10 (PE/EA = 1:1); White solid; 46 mg, 67%; ^{1}H NMR (400 MHz, $CDCl_3$): δ 7.89-7.83 (m, 4H), 7.54-7.43 (m, 6H), 3.25 (d, J = 5.6 Hz, 1H), 2.47 (s, 1H), 1.85 (d, J = 18.8 Hz, 1H), 1.46-1.38 (m, 1H), 1.21-0.98 (m, 12H), 0.75 (t, J = 6.8 Hz, 3H); ^{13}C NMR (100 MHz, $CDCl_3$): δ 131.72 (d, $J_{C\text{-}P}$ = 7.5 Hz), 131.66 (d, $J_{C\text{-}P}$ = 91.2 Hz), 131.5 (d, $J_{C\text{-}P}$ = 1.7 Hz), 131.4 (d, $J_{C\text{-}P}$ = 2.3 Hz), 128.4 (d, $J_{C\text{-}P}$ = 10.8 Hz), 128.37 (d, $J_{C\text{-}P}$ = 10.4 Hz), 73.8 (d, $J_{C\text{-}P}$ = 9.0 Hz), 46.2, 30.6 (d, $J_{C\text{-}P}$ = 72.0 Hz), 30.4, 29.6, 24.5, 23.0, 14.4, 10.5; ^{31}P NMR (162 MHz, $CDCl_3$): 39.0; HRMS (ESI) m/z: $[M+H]^+$ Calcd for $C_{21}H_{30}O_2P^+$ 345.1978, Found 345.1969.

((1R*,2S*)-2-(2-hydroxypropan-2-yl)cyclohexyl)diphenylphosphine oxide (2ae)

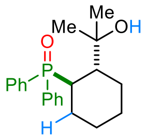

This compound was synthesized by **Standard conditions A**; Column chromatography conditions (PE/EA = 5:1 to 1:1), R_f = 0.25 (PE/EA = 1:1); White solid; 48 mg, 70%; ^1H NMR (400 MHz, $CDCl_3$): δ 7.86-7.71 (m, 4H), 7.52-7.42 (m, 6H), 6.01 (s, 1H), 3.12-3.10 (m, 1H), 2.12-2.06 (m, 1H), 1.93-1.62 (m, 5H), 1.33-1,13 (m, 8H), 0.81 (s, 1H); ^{13}C NMR (100 MHz, $CDCl_3$): δ 134.2 (d, J_{C-P} = 95.6 Hz), 133.4 (d, J_{C-P} = 93.8 Hz), 131.9 (d, J_{C-P} = 9.4 Hz), 131.6 (d, J_{C-P} = 2.3 Hz), 131.4 (d, J_{C-P} = 2.3 Hz), 130.4 (d, J_{C-P} = 8.1 Hz), 128.8 (d, J_{C-P} = 10.8 Hz), 128.4 (d, J_{C-P} = 10.9 Hz), 70.7, 52.2, 38.4 (d, J_{C-P} = 66.0 Hz), 29.78, 29.75, 27.0, 26.4, 25.2, 22.6; ^{31}P NMR (162 MHz, $CDCl_3$): 37.5; HRMS (ESI) m/z: $[M+H]^+$ Calcd for $C_{21}H_{28}O_2P^+$ 343.1821, Found 343.1812.

(1-(1-hydroxycyclohexyl)propan-2-yl)di-p-tolylphosphine oxide (2af)

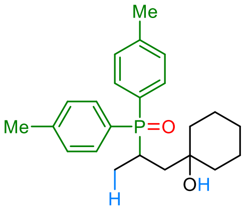

This compound was synthesized by **Standard conditions A**; Column chromatography conditions (PE/EA = 5:1 to 1:1), R_f = 0.15 (PE/EA = 1:1); White solid; 44 mg, 60%; ^1H NMR (400 MHz, $CDCl_3$): δ 7.69-7.65 (m, 4H), 7.29-7.27 (m, 4H), 3.25 (s, 1H), 2.85-2.77 (m, 1H), 2.40-2.39 (m, 6H), 1.87 (td, J_1 = 12.0 Hz, J_2 = 5.6 Hz, 1H), 1.67-1.50 (m, 5H), 1.43-1.25 (m, 6H), 1.21-1.15 (m, 3H); ^{13}C NMR (100 MHz, $CDCl_3$): δ 142.2 (d, J_{C-P} = 3.0 Hz), 141.1 (d, J_{C-P} = 3.0 Hz), 132.1 (d, J_{C-P} = 8.5 Hz), 131.3 (d, J_{C-P} = 9.2 Hz), 129.3 (d, J_{C-P} = 11.6 Hz), 129.1 (d, J_{C-P} = 11.6 Hz), 128.8 (d, J_{C-P} = 95.3 Hz), 127.0 (d, J_{C-P} = 96.5 Hz), 70.4 (d, J_{C-P} = 7.0 Hz), 42.6, 39.2, 37.0, 27.7 (d, J_{C-P} = 72.0 Hz), 25.9, 22.3 (d, J_{C-P} = 8.0 Hz), 21.5, 16.7; ^{31}P NMR (162 MHz, $CDCl_3$): 41.4; HRMS (ESI) m/z: $[M+H]^+$ Calcd for $C_{23}H_{32}O_2P^+$ 371.2134, Found 371.2124.

(1-(1-hydroxycyclohexyl)propan-2-yl)bis(4-methoxyphenyl)phosphine oxide (2ag)

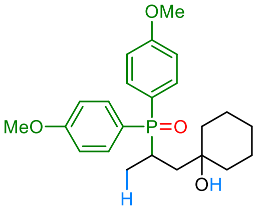

This compound was synthesized by **Standard conditions A**; Column chromatography conditions (PE/EA = 5:1 to 1:1), R_f = 0.15 (EA); White solid; 52 mg, 65%; ^1H NMR (400 MHz, $CDCl_3$): δ 7.73-7.68 (m, 4H), 7.00-6.96 (m, 4H), 3.85-3.84 (m, 6H), 3.63 (s, 1H), 2.83-2.75 (m, 1H), 1.87 (td, J_1 = 15.6 Hz, J_2 = 5.6 Hz, 1H), 1.72-1.52 (m, 6H), 1.44-1.41 (m, 3H), 1.38-1.21 (m, 5H); ^{13}C NMR (100 MHz, $CDCl_3$): δ 162.20 (d,

$J_{C\text{-}P}$ = 3.0 Hz), 162.17 (d, $J_{C\text{-}P}$ = 3.0 Hz), 133.9 (d, $J_{C\text{-}P}$ = 10.0 Hz), 133.1 (d, $J_{C\text{-}P}$ = 10.0 Hz), 123.2 (d, $J_{C\text{-}P}$ = 102.0 Hz), 121.2 (d, $J_{C\text{-}P}$ = 101.0 Hz), 114.1 (d, $J_{C\text{-}P}$ = 12.0 Hz), 113.9 (d, $J_{C\text{-}P}$ = 12.0 Hz), 70.2 (d, $J_{C\text{-}P}$ = 6.0 Hz), 55.3, 42.7, 39.2, 36.9, 28.3 (d, $J_{C\text{-}P}$ = 72.0 Hz), 25.8, 22.2 (d, $J_{C\text{-}P}$ = 9.0 Hz), 16.8; ^{31}P NMR (162 MHz, $CDCl_3$): 41.2; HRMS (ESI) m/z: $[M+H]^+$ Calcd for $C_{23}H_{32}O_4P^+$ 403.2033, Found 403.2022.

4,4'-((1-(1-hydroxycyclohexyl)propan-2-yl)phosphoryl)dibenzonitrile (2ah)

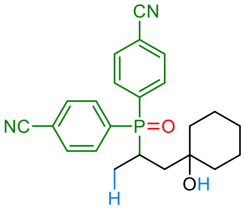

This compound was synthesized by **Standard conditions A**; Column chromatography conditions (PE/EA = 5:1 to EA), R_f = 0.10 (EA); White solid; 31 mg, 40%; ^{1}H NMR (400 MHz, $CDCl_3$): δ 8.02-7.90 (m, 4H), 7.80-7.78 (m, 4H), 2.93-2.88 (m, 1H), 2.31 (s, 1H), 1.85-1.76 (m, 1H), 1.67-1.39 (m, 8H), 1.31-1.25 (m, 6H); ^{13}C NMR (100 MHz, $CDCl_3$): δ 136.9 (d, $J_{C\text{-}P}$ = 92.5 Hz), 136.5 (d, $J_{C\text{-}P}$ = 91.6 Hz), 132.2 (d, $J_{C\text{-}P}$ = 11 Hz), 131.88 (d, $J_{C\text{-}P}$ = 8.8 Hz), 131.87 (d, $J_{C\text{-}P}$ = 8.7 Hz), 117.6, 115.93 (d, $J_{C\text{-}P}$ = 2.0 Hz), 115.88 (d, $J_{C\text{-}P}$ = 3.0 Hz), 71.1 (d, $J_{C\text{-}P}$ = 10.0 Hz), 41.2, 38.6, 37.3, 26.8 (d, $J_{C\text{-}P}$ = 72.0 Hz), 25.5, 21.97, 21.95, 15.5 (d, $J_{C\text{-}P}$ = 2.0 Hz); ^{31}P NMR (162 MHz, $CDCl_3$): 38.0; HRMS (ESI) m/z: $[M+H]^+$ Calcd for $C_{23}H_{26}N_2O_2P^+$ 393.1726, Found 393.1718.

(1-(1-hydroxycyclohexyl)propan-2-yl)di(naphthalen-2-yl)phosphine oxide (2ai)

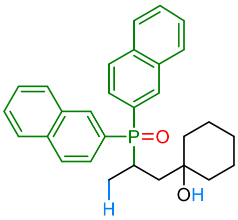

This compound was synthesized by **Standard conditions A**; Column chromatography conditions (PE/EA = 5:1 to 1:1), R_f = 0.10 (PE/EA = 1:1); White solid; 62 mg, 70%; ^{1}H NMR (400 MHz, $CDCl_3$): δ 8.51 (t, J = 11.6 Hz, 2H), 7.95-7.75 (m, 8H), 7.60-7.52 (m, 4H), 3.11-3.04 (m, 1H), 2.77 (s, 1H), 1.99 (td, J_1 = 16.0 Hz, J_2 = 4.0 Hz, 1H), 1.71-1.42 (m, 8H), 1.36-1.26 (m, 6H); ^{13}C NMR (100 MHz, $CDCl_3$): δ 134.6 (d, $J_{C\text{-}P}$ = 2.2 Hz), 134.1 (d, $J_{C\text{-}P}$ = 7.9 Hz), 133.4 (d, $J_{C\text{-}P}$ = 7.8 Hz), 132.6, 132.5, 129.2 (d, $J_{C\text{-}P}$ = 95.3 Hz), 128.91, 128.86, 128.4 (d, $J_{C\text{-}P}$ = 11.6 Hz), 128.2 (d, $J_{C\text{-}P}$ = 11.6 Hz), 128.10, 128.09, 128.04 (d, $J_{C\text{-}P}$ = 93.9 Hz)127.7 (d, $J_{C\text{-}P}$ = 1.8 Hz), 126.90, 128.86, 126.3 (d, $J_{C\text{-}P}$ = 10.1 Hz), 126.0 (d, $J_{C\text{-}P}$ = 9.5 Hz), 70.8 (d, $J_{C\text{-}P}$ = 8.0 Hz), 42.3, 39.0, 37.2, 27.4 (d, $J_{C\text{-}P}$ = 71.0 Hz), 25.7, 22.19, 22.16, 16.38 (d, $J_{C\text{-}P}$ = 1.0 Hz); ^{31}P

NMR (162 MHz, $CDCl_3$): 40.9; HRMS (ESI) m/z: $[M+H]^+$ Calcd for $C_{29}H_{32}O_2P^+$ 443.2134, Found 443.2124.

(4-hydroxybutan-2-yl-1-d)diphenylphosphine oxide (2a-D)

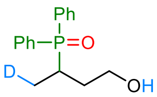

This compound was synthesized by **Standard conditions B**; Column chromatography conditions (PE/EA = 5:1 to EA), R_f = 0.10 (EA); White solid; 40 mg, 73%; ^1H NMR (400 MHz, $CDCl_3$): *δ* 7.83-7.77 (m, 4H), 7.53-7.45 (m, 6H), 4.22 (s, 1H), 3.81-3.76 (m, 1H), 3.66-3.60 (m, 1H), 2.79-2.70 (m, 1H), 1.96-1.75 (m, 2H), 1.21-1.14 (m, 2.07H, 93% D); ^2H NMR (92 MHz, $CHCl_3$): *δ* 1.19; ^{13}C NMR (100 MHz, $CDCl_3$): *δ* 131.65 (d, $J_{C\text{-}P}$ = 2.7 Hz), 131.63 (d, $J_{C\text{-}P}$ = 2.8 Hz), 131.62 (d, $J_{C\text{-}P}$ = 98.1 Hz), 131.3 (d, $J_{C\text{-}P}$ = 95.9 Hz), 130.1 (d, $J_{C\text{-}P}$ = 9.1 Hz), 131.0 (d, $J_{C\text{-}P}$ = 8.9 Hz), 128.7 (d, $J_{C\text{-}P}$ = 10.7 Hz), 128.6 (d, $J_{C\text{-}P}$ = 10.6 Hz), 58.6 (d, $J_{C\text{-}P}$ = 9 Hz), 32.5, 29.4 (d, $J_{C\text{-}P}$ = 71.0 Hz), 11.8 (t, $J_{C\text{-}D}$ = 20.0 Hz); ^{31}P NMR (162 MHz, $CDCl_3$): δ 37.9; HRMS (ESI) m/z: $[M+H]^+$ Calcd for $C_{16}H_{19}DO_2P^+$ 276.1258, Found 276.1250.

(4-hydroxy-2-methylbutan-2-yl-1-d)diphenylphosphine oxide (2b-D)

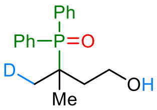

This compound was synthesized by **Standard conditions B**; Column chromatography conditions (PE/EA = 5:1 to 1:1), R_f = 0.10 (PE/EA = 1:1); White solid; 42 mg, 73%; ^1H NMR (400 MHz, $CDCl_3$): *δ* 8.00-7.95 (m, 4H), 7.55-7.49 (m, 6H), 5.48 (s, 1H), 3.74 (s, 2H), 1.86-1.79 (m, 2H), 1.29-1.24 (m, 5.06H, 94% D); ^2H NMR (92 MHz, $CHCl_3$): *δ* 1.27; ^{13}C NMR (100 MHz, $CDCl_3$): *δ* 132.2 (d, $J_{C\text{-}P}$ = 8.1 Hz), 131.6 (d, $J_{C\text{-}P}$ = 2.1 Hz), 129.9 (d, $J_{C\text{-}P}$ = 91.3 Hz), 128.3 (d, $J_{C\text{-}P}$ = 10.9 Hz), 57.9 (d, $J_{C\text{-}P}$ = 4.0 Hz), 42.0, 36.8 (d, $J_{C\text{-}P}$ = 69.0 Hz), 23.2 (t, $J_{C\text{-}D}$ = 19.0 Hz); ^{31}P NMR (162 MHz, $CDCl_3$): δ 41.6; HRMS (ESI) m/z: $[M+H]^+$ Calcd for $C_{17}H_{21}DO_2P^+$ 290.1415, Found 290.1406.

(5-hydroxypentan-2-yl-1-d)diphenylphosphine oxide (2c-D)

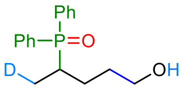

This compound was synthesized by **Standard conditions B**; Column chromatography conditions (PE/EA = 5:1 to EA), R_f = 0.10 (EA); White solid; 39 mg, 67%; ^1H NMR (400 MHz, $CDCl_3$): *δ* 7.80-7.74 (m, 4H), 7.50-7.42 (m, 6H), 3.66 (s, 1H) , 3.56-3.54 (m, 2H), 2.44-2.41 (m, 1H), 1.83-1.71 (m, 2H), 1.53-1.43 (m, 2H), 1.17-1.10 (m, 2.10H, 90% D) ; ^2H NMR (92 MHz, $CHCl_3$): *δ* 1.13; ^{13}C NMR (100 MHz, $CDCl_3$): *δ*

131.9 (d, J_{C-P} = 93.2 Hz), 131.8 (d, J_{C-P} = 94.5 Hz), 131.5 (d, J_{C-P} = 2.1 Hz), 131.4 (d, J_{C-P} = 2.6 Hz), 130.95 (d, J_{C-P} = 8.6 Hz), 130.88 (d, J_{C-P} = 8.7 Hz), 128.54 (d, J_{C-P} = 10.9 Hz), 128.45 (d, J_{C-P} = 10.7 Hz), 61.8, 31.5 (d, J_{C-P} = 72.0 Hz), 30.3 (d, J_{C-P} = 11.0 Hz), 25.2, 11.8 (t, J_{C-D} = 20.0 Hz); ^{31}P NMR (162 MHz, $CDCl_3$): δ 37.9; HRMS (ESI) m/z: $[M+H]^+$ Calcd for $C_{17}H_{21}DO_2P^+$ 290.1415, Found 290.1406.

(4-hydroxy-2-methyl-5-phenylpentan-2-yl-1-d)diphenylphosphine oxide (2h-D)

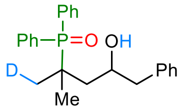

This compound was synthesized by **Standard conditions B**; Column chromatography conditions (PE/EA = 10:1 to 4:1), R_f = 0.15 (PE/EA = 3:1); White solid; 55 mg, 73%; ^{1}H NMR (400 MHz, $CDCl_3$): *δ* 8.06-7.97 (m, 4H), 7.62-7.51 (m, 6H), 7.34-7.30 (m, 2H), 7.25-7.22 (m, 3H), 6.05 (s, 1H), 4.27-4.21 (m, 1H), 3.00 (dd, J_1 =13.2 Hz, J_2 =6.0 Hz, 1H), 2.63 (dd, J_1 =13.6 Hz, J_2 = 7.2 Hz, 1H), 1.96-1.87 (m, 1H), 1.63-1.54 (m, 1H), 1.30-1.22 (m, 5.10H, 90% D); ^{2}H NMR (92 MHz, $CHCl_3$): *δ* 1.19 ^{13}C NMR (100 MHz, $CDCl_3$): *δ* 139.2, 132.5 (d, J_{C-P} = 8.1 Hz), 132.3 (d, J_{C-P} = 7.9 Hz), 131.88 (d, J_{C-P} = 2.2 Hz), 131.86 (d, J_{C-P} = 2.0 Hz), 130.1 (d, J_{C-P} = 91.8 Hz), 130.0 (d, J_{C-P} = 90.3), 129.4, 128.51 (d, J_{C-P} = 10.9 Hz), 128.46 (d, J_{C-P} = 10.9 Hz), 128.3, 126.1, 68.3 (d, J_{C-P} = 3.0 Hz), 46.5, 44.5, 36.9 (d, J_{C-P} = 68.0 Hz), 26.3, 26.0 (t, J_{C-D} = 19.0 Hz), 22.2, 21.9 (t, J_{C-D} = 19.0 Hz); ^{31}P NMR (162 MHz, $CDCl_3$): 42.5; HRMS (ESI) m/z: $[M+H]^+$ Calcd for $C_{24}H_{27}DO_2P^+$ 380.1884, Found 380.1877.

(4-cyclopentyl-4-hydroxy-2-methylbutan-2-yl-1-d)diphenylphosphine oxide (2i-D)

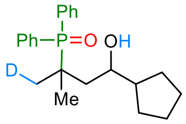

This compound was synthesized by **Standard conditions B**; Column chromatography conditions (PE/EA = 10:1 to 4:1), R_f = 0.10 (PE/EA = 2:1); White solid; 52 mg, 73%; ^{1}H NMR (400 MHz, $CDCl_3$): *δ* 8.03-7.94 (m, 4H), 7.55-7.46 (m, 6H), 5.93 (s, 1H), 3.67-3.63 (m, 1H), 1.92-1.76 (m, 3H), 1.61-1.46 (m, 7H), 1.30-1.24 (m, 5.07H, 93% D), 1.18-1.11 (m, 1H); ^{2}H NMR (92 MHz, $CHCl_3$): *δ* 1.32; ^{13}C NMR (100 MHz, $CDCl_3$): δ 132.4 (d, J_{C-P} = 8.2 Hz), 132.3 (d, J_{C-P} = 8.0 Hz), 131.71 (d, J_{C-P} = 2.8 Hz), 131.68 (d, J_{C-P} = 2.7 Hz), 130.2 (d, J_{C-P} = 91.4 Hz), 130.1 (d, J_{C-P} = 90.6 Hz), 128.4 (d, J_{C-P} = 10.9 Hz), 128.3 (d, J_{C-P} = 10.9 Hz), 70.9 (d, J_{C-P} = 1.0 Hz), 47.1, 46.6, 36.8 (d, J_{C-P} = 68.0 Hz), 29.6, 28.9, 26.7, 26.6, 26.4 (t, J_{C-D} = 19.0 Hz), 25.7, 22.3, 22.1 (d, J_{C-D} = 19.0 Hz); ^{31}P NMR (162 MHz, $CDCl_3$): 42.7; HRMS (ESI) m/z: $[M+H]^+$ Calcd for $C_{22}H_{29}DO_2P^+$ 358.2041, Found 358.2030.

(4-hydroxy-2-methyl-4-phenylbutan-2-yl-1-d)diphenylphosphine oxide (2j-D)

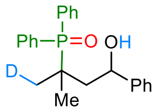

This compound was synthesized by **Standard conditions B**; Column chromatography conditions (PE/EA = 10:1 to 4:1), R_f = 0.10 (PE/EA = 3:1); White solid; 51 mg, 70%; ^{1}H NMR (400 MHz, $CDCl_3$): δ 8.05-7.99 (m, 4H), 7.59-7.51 (m, 6H), 7.32-7.26 (m, 4H), 7.21-7.18 (m, 1H), 6.09 (s, 1H), 4.99 (d, J =7.2 Hz, 1H), 2.20-2.14 (m, 1H), 1.67-1.60 (m, 1H), 1.45-1.41 (m, 2.48H), 1.31-1.26 (m, 2.64H, 88% D); ^{2}H NMR (92 MHz, $CHCl_3$): δ 1.46, 1.31; ^{13}C NMR (100 MHz, $CDCl_3$): δ 145.7, 132.5 (d, J_{C-P} = 7.9 Hz), 132.3 (d, J_{C-P} = 8.1 Hz), 131.90 (d, J_{C-P} = 3.0 Hz), 131.87 (d, J_{C-P} = 2.9 Hz), 129.8 (d, J_{C-P} = 92.4 Hz), 129.7 (d, J_{C-P} = 90.6 Hz), 128.3 (d, J_{C-P} = 11.0 Hz), 128.1, 126.8, 125.7, 69.7 (d, J_{C-P} = 2.0 Hz), 50.4, 37.2 (d, J_{C-P} = 68.0 Hz), 26.0 (t, J_{C-D} = 23.0 Hz), 21.9; ^{31}P NMR (162 MHz, $CDCl_3$): 42.5; HRMS (ESI) m/z: $[M+H]^+$ Calcd for $C_{23}H_{25}DO_2P^+$ 366.1728, Found 366.1715.

((1S*,3R*)-3-hydroxycyclopentyl-5-d)diphenylphosphine oxide (2k-D)

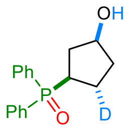

This compound was synthesized by **Standard conditions B**; Column chromatography conditions (PE/EA = 10:1 to 2:1), R_f = 0.10 (PE/EA = 2:1); White solid; 40 mg, 70%; ^{1}H NMR (400 MHz, $CDCl_3$): δ 7.84-7.75 (m,4H), 7.55-7.43 (m, 6H), 4.91 (s, 1H), 4.31 (s, 1H), 3.02-2.99 (m, 1H), 2.16-1.87 (m, 4.10H, 90% D), 1.78-1.71 (m, 1H); ^{2}H NMR (92 MHz, $CHCl_3$): δ 1.91; ^{13}C NMR (100 MHz, $CDCl_3$): δ 132.3 (d, J_{C-P} = 96.5 Hz), 131.75 (d, J_{C-P} = 2.5 Hz), 131.69 (d, J_{C-P} = 2.7 Hz), 131.68 (d, J_{C-P} = 97.5 Hz), 130.9 (d, J_{C-P} = 8.8 Hz), 130.8 (d, J_{C-P} = 8.8 Hz), 128.63 (d, J_{C-P} = 11.6 Hz), 128.62 (d, J_{C-P} = 11.5 Hz), 72.8, 36.5(d, J_{C-P} = 5.0 Hz), 35.8(d, J_{C-P} = 2.0 Hz), 34.6 (d, J_{C-P} = 71.0 Hz), 23.0 (t, J_{C-D} = 20.0 Hz); ^{31}P NMR (162 MHz, $CDCl_3$): 39.5; HRMS (ESI) m/z: $[M+H]^+$ Calcd for $C_{17}H_{19}DO_2P^+$ 288.1258, Found 288.1260.

(4-hydroxy-4-methylpentan-2-yl-1-d)diphenylphosphine oxide (2l-D)

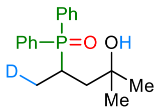

This compound was synthesized by **Standard conditions B**; Column chromatography conditions (PE/EA = 5:1 to 1:1), R_f = 0.10 (PE/EA = 1:1); White solid; 41 mg, 67%; ^{1}H NMR (400 MHz, $CDCl_3$): δ 7.85-7.78 (m,4H), 7.53-7.43 (m, 6H), 4.09 (s, 1H), 2.89-2.80 (m, 1H), 2.02-1.93 (m, 1H), 1.61-1.53 (m, 1H),

1.28-1.17 (m, 8.10H, 90% D); ^{2}H NMR (92 MHz, $CHCl_3$): δ 1.22; ^{13}C NMR (100 MHz, $CDCl_3$): δ 131.62 (d, J_{C-P} = 96.3 Hz), 131.61 (d, J_{C-P} = 8.7 Hz), 131.51 (d, J_{C-P} = 4.9 Hz), 131.47 (d, J_{C-P} = 2.5 Hz), 130.4 (d, J_{C-P} = 94.0 Hz), 128.4 (d, J_{C-P} = 10.9 Hz), 128.2 (d, J_{C-P} = 11.2 Hz), 69.6 (d, J_{C-P} = 8.0 Hz), 43.9, 30.4, 29.2, 28.3 (d, J_{C-P} = 70.0 Hz), 15.6 (t, J_{C-D} = 20.0 Hz); ^{31}P NMR (162 MHz, $CDCl_3$): 41.1; HRMS (ESI) m/z: $[M+H]^+$ Calcd for $C_{18}H_{23}DO_2P^+$ 304.1571, Found 304.1560.

(4-benzyl-4-hydroxy-2-methyl-5-phenylpentan-2-yl-1-d)diphenylphosphine oxide (2m-D)

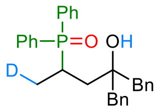

This compound was synthesized by **Standard conditions B**; Column chromatography conditions (PE/EA = 10:1 to 3:1), R_f = 0.10 (PE/EA = 3:1); White solid; 64 mg, 70%; ^{1}H NMR (400 MHz, $CDCl_3$): δ 7.56-7.46 (m,4H), 7.40-7.24 (m, 14H), 7.20-7.18 (m, 2H), 4.82 (s, 1H), 2.90 (s, 2H), 2.85-2.82 (d, 1H), 2.70-2.61 (m, 2H), 2.06-1.96 (m, 1H), 1.41-1.33 (m, 1H), 0.98-0.90 (m, 2.13H, 87% D); ^{2}H NMR (92 MHz, $CHCl_3$): δ 0.96; ^{13}C NMR (100 MHz, $CDCl_3$): δ 138.1, 137.6, 132.6 (d, J_{C-P} = 8.4 Hz), 131.8 (d, J_{C-P} = 2.1 Hz), 131.7 (d, J_{C-P} = 2.3 Hz), 131.3 (d, J_{C-P} = 8.9 Hz), 131.1 (d, J_{C-P} = 96.1 Hz), 131.0, 130.8, 128.55 (d, J_{C-P} = 11.2 Hz), 128.53 (d, J_{C-P} = 94.5 Hz), 128.00 (d, J_{C-P} = 12.2 Hz), 127.95, 126.3,126.1, 72.8 (d, J_{C-P} = 3.0 Hz), 47.7 (d, J_{C-P} = 20.0 Hz), 37.9, 26.8 (d, J_{C-P} = 69.0 Hz), 16.7 (t, J_{C-D} = 20.0 Hz); ^{31}P NMR (162 MHz, $CDCl_3$): 42.9; HRMS (ESI) m/z: $[M+H]^+$ Calcd for $C_{30}H_{31}DO_2P^+$ 456.2197, Found 456.2197.

(1-(1-hydroxycyclobutyl)propan-2-yl-3-d)diphenylphosphine oxide (2p-D)

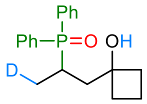

This compound was synthesized by **Standard conditions B**; Column chromatography conditions (PE/EA = 5:1 to 1:1), R_f = 0.15 (PE/EA = 1:1); White solid; 43 mg, 69%; ^{1}H NMR (400 MHz, $CDCl_3$): δ 7.81-7.77 (m,4H), 7.56-7.45 (m, 6H), 4.56 (s, 1H), 2.82-2.73 (m, 1H), 2.12-2.00 (m, 4H), 1.91-1.69 (m, 3H), 1.48-1.36 (m, 1H), 1.26-1.18 (m, 2,13H, 87% D); ^{2}H NMR (92 MHz, $CHCl_3$): δ 1.23; ^{13}C NMR (100 MHz, $CDCl_3$): δ 131.9 (d, J_{C-P} = 8.4 Hz), 131.78 (d, J_{C-P} = 2.0 Hz), 131.76 (d, J_{C-P} = 2.5 Hz), 131.2 (d, J_{C-P} = 8.6 Hz), 130.1 (d, J_{C-P} = 97.1 Hz), 128.6 (d, J_{C-P} = 11.3 Hz), 128.4 (d, J_{C-P} = 10.7 Hz), 74.4 (d, J_{C-P} = 7.0 Hz), 39.3, 37.0, 36.2, 29.2 (d, J_{C-P} = 70.0 Hz), 15.4 (t, J_{C-D} = 19.0 Hz), 12.5; ^{31}P NMR (162 MHz, $CDCl_3$): 40.9; HRMS (ESI) m/z: $[M+H]^+$ Calcd for $C_{19}H_{23}DO_2P^+$ 316.1571, Found 316.1564.

(1-(1-hydroxycyclopentyl)propan-2-yl-3-d)diphenylphosphine oxide (2q-D)

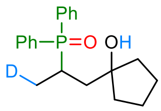

This compound was synthesized by **Standard conditions B**; Column chromatography conditions (PE/EA = 5:1 to 1:1), R_f = 0.15 (PE/EA = 1:1); White solid; 49 mg, 75%; R_f = 0.10 (1:1 PE/EA); ^{1}H NMR (400 MHz, $CDCl_3$): δ 7.83-7.78 (m,4H), 7.55-7.45 (m, 6H), 3.45 (s, 1H), 2.90-2.81 (m, 1H), 2.17-2.08 (m, 1H), 1.87-1.73 (m, 3H), 1.69-1.53 (m, 5H), 1.48-1.38 (m, 1H), 1.27-1.19 (m, 2.14H, 86% D); ^{2}H NMR (92 MHz, $CHCl_3$): δ 1.24; ^{13}C NMR (100 MHz, $CDCl_3$): δ 131.9 (d, $J_{C\text{-}P}$ = 95.9 Hz), 131.7 (d, $J_{C\text{-}P}$ = 8.8 Hz), 131.65 (d, $J_{C\text{-}P}$ = 2.6 Hz), 131.59 (d, $J_{C\text{-}P}$ = 2.3 Hz), 131.2 (d, $J_{C\text{-}P}$ = 8.9 Hz), 130.5 (d, $J_{C\text{-}P}$ = 93.7 Hz), 128.6 (d, $J_{C\text{-}P}$ = 11.3 Hz), 128.4 (d, $J_{C\text{-}P}$ = 10.9 Hz), 81.2 (d, $J_{C\text{-}P}$ = 8.0 Hz), 41.2, 41.0, 39.5, 29.4 (d, $J_{C\text{-}P}$ = 71.0 Hz), 23.7, 23.1, 15.6 (t, $J_{C\text{-}D}$ = 20.0 Hz); ^{31}P NMR (162 MHz, $CDCl_3$): 40.7; HRMS (ESI) m/z: $[M+H]^+$ Calcd for $C_{20}H_{25}DO_2P^+$ 330.1728, Found 330.1719.

(1-(4-hydroxytetrahydro-2H-pyran-4-yl)propan-2-yl-3-d)diphenylphosphine oxide (2aa-D)

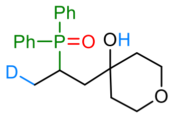

This compound was synthesized by **Standard conditions B**; Column chromatography conditions (PE/EA = 5:1 to EA), R_f = 0.10 (EA); White solid; 37 mg, 54%; ^{1}H NMR (400 MHz, $CDCl_3$): δ 7.81-7.75 (m,4H), 7.57-7.46 (m, 6H), 4.67 (s, 1H), 3.85-3.79 (m, 2H), 3.73-3.64 (m, 2H), 2.95-2.85 (m, 1H), 2.00-1.91 (m, 1H), 1.75-1.72 (m, 1H), 1.65-1.59 (m, 2H), 1.56-1.51 (m, 2H), 1.22-1.15 (m, 2.07H, 93% D); ^{2}H NMR (92 MHz, $CHCl_3$): δ 1.18; ^{13}C NMR (100 MHz, $CDCl_3$): δ 132.2 (d, $J_{C\text{-}P}$ = 8.7 Hz), 132.02 (d, $J_{C\text{-}P}$ = 2.7 Hz), 131.97 (d, $J_{C\text{-}P}$ = 2.4 Hz), 131.4 (d, $J_{C\text{-}P}$ = 97.1 Hz), 131.7 (d, $J_{C\text{-}P}$ = 9.0 Hz), 129.1 (d, $J_{C\text{-}P}$ = 94.0 Hz), 128.7 (d, $J_{C\text{-}P}$ = 11.3 Hz), 128.4 (d, $J_{C\text{-}P}$ = 11.5 Hz), 67.3 (d, $J_{C\text{-}P}$ = 6.0 Hz), 63.9, 63.8, 44.4, 39.2, 37.2, 27.6 (d, $J_{C\text{-}P}$ = 70.0 Hz), 16.7 (t, $J_{C\text{-}D}$ = 20.0 Hz); ^{31}P NMR (162 MHz, $CDCl_3$): 41.6; HRMS (ESI) m/z: $[M+H]^+$ Calcd for $C_{20}H_{25}DO_3P^+$ 346.1677, Found 346.1668.

(2-((1R,2R,4R)-2-hydroxy-4-methylcyclohexyl)propan-2-yl)diphenylphosphine oxide (2al)

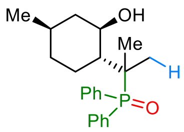

This compound was synthesized by **Standard conditions A**; Column chromatography conditions (PE/EA = 20:1 to 5:1), R_f = 0.10 (PE/EA = 5:1); White solid; 59 mg, 83%; ^{1}H NMR (400 MHz, $CDCl_3$): δ 8.01-7.90 (m,4H), 7.57-7.46(m, 6H), 6.82 (d, J = 1.6 Hz, 1H), 3.58-3.54 (m, 1H), 1.98-1.94 (m, 1H), 1.77-1.72 (m,

1H), 1.62-1.56 (m, 2H), 1.33-1.29 (m, 7H), 0.98-0.82 (m, 5H), 0.76-0.65 (m, 1H); ^{13}C NMR (100 MHz, $CDCl_3$): δ 132.6 (d, $J_{C\text{-}P}$ = 8.1 Hz), 132.3 (d, $J_{C\text{-}P}$ = 8.0 Hz), 131.6 (d, $J_{C\text{-}P}$ = 1.1 Hz), 130.9 (d, $J_{C\text{-}P}$ = 92.5 Hz), 130.2 (d, $J_{C\text{-}P}$ = 89.7 Hz), 128.4 (d, $J_{C\text{-}P}$ = 10.9 Hz), 128.2 (d, $J_{C\text{-}P}$ = 11.0 Hz), 70.1, 50.1, 44.4, 44.0 (d, $J_{C\text{-}P}$ = 65.0 Hz), 34.7, 30.9, 25.9 (d, $J_{C\text{-}P}$ = 7.0 Hz), 25.1, 21.7, 18.8; ^{31}P NMR (162 MHz, $CDCl_3$): 45.3; HRMS (ESI) m/z: $[M+H]^+$ Calcd for $C_{22}H_{30}O_2P^+$ 357.1978, Found 357.1970.

(2-((1R,2R,4R)-2-hydroxy-4-methylcyclohexyl)propan-2-yl-1-d)diphenylphosphine oxide (2al-D)

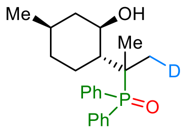

This compound was synthesized by **Standard conditions B**; Column chromatography conditions (PE/EA = 20:1 to 5:1), R_f = 0.10 (PE/EA = 5:1); White solid; 58 mg, 81%; ^{1}H NMR (400 MHz, $CDCl_3$): δ 8.01-7.90 (m,4H), 7.56-7.44(m, 6H), 6.82 (s, 1H), 3.59-3.53 (m, 1H), 1.98-1.94 (m, 1H), 1.77-1.73 (m, 1H), 1.62-1.53 (m, 2H), 1.33-1.28 (m, 6.11 H, 89% D), 0.97-0.82 (m, 5H), 0.75-0.65 (m, 1H); ^{2}H NMR (92 MHz, $CHCl_3$): δ 1.31; ^{13}C NMR (100 MHz, $CDCl_3$): δ 132.5 (d, $J_{C\text{-}P}$ = 8.1 Hz), 132.2 (d, $J_{C\text{-}P}$ = 8.1 Hz), 131.55 (d, $J_{C\text{-}P}$ = 2.0 Hz), 131.53 (d, $J_{C\text{-}P}$ = 2.3 Hz), 130.9 (d, $J_{C\text{-}P}$ = 92.5 Hz), 130.1 (d, $J_{C\text{-}P}$ = 89.8 Hz), 128.3 (d, $J_{C\text{-}P}$ = 10.9 Hz), 128.1 (d, $J_{C\text{-}P}$ = 11.6 Hz), 70.1, 50.03, 50.00, 44.4, 39.9 (d, $J_{C\text{-}P}$ = 65.0 Hz), 34.6, 30.9, 25.8 (d, $J_{C\text{-}P}$ = 7.0 Hz), 25.0, 24.7 (t, $J_{C\text{-}D}$ = 20.0 Hz), 18.8, 18.5 (t, $J_{C\text{-}D}$ = 20.0 Hz); ^{31}P NMR (162 MHz, $CDCl_3$): 45.3; HRMS (ESI) m/z: $[M+H]^+$ Calcd for $C_{22}H_{29}DO_2P^+$ 358.2041, Found 358.2029.

tert-butyl(*S*)-2-(3-(diphenylphosphoryl)-1-hydroxy-3-methylbutyl)pyrrolidine-1-carboxylate (2am)

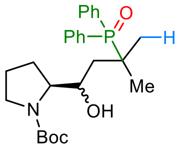

This compound was synthesized by **Standard conditions A**; Column chromatography conditions (PE/EA =5:1 to EA), R_f = 0.10 (EA); White solid; 75 mg, 82%, dr = 1:1.2; ^{1}H NMR (400 MHz, $CDCl_3$): δ 8.00-7.93 (m,4H), 7.50-7.49 (m, 6H), 5.80-5.79 (m, 1H), 3.97 (s, 1H), 3.76-3.61 (m, 1H), 3.44 (s, 1H), 3.22-3.20 (m, 1H), 2.08-1.54 (m, 6H), 1.42-1.27 (m, 15H); ^{13}C NMR (100 MHz, $CDCl_3$): δ 155.6, 154.9, 132.4 (d, $J_{C\text{-}P}$ = 8.0 Hz), 132.2 (d, $J_{C\text{-}P}$ = 8.1 Hz), 131.6, 130.3 (d, $J_{C\text{-}P}$ = 89.0 Hz), 129.9 (d, $J_{C\text{-}P}$ = 90.5 Hz), 128.3 (d, $J_{C\text{-}P}$ = 9.1 Hz), 79.2, 68.4, 67.7, 63.2, 62.5, 47.4, 46.5, 44.2, 41.5, 36.6 (d, $J_{C\text{-}P}$ = 66.0 Hz), 36.5, 28.4, 26.7, 26.4, 24.8, 24.0, 23.1, 22.1, 21.9; ^{31}P NMR (162 MHz, $CDCl_3$): 42.4, 41.5; HRMS (ESI) m/z: $[M+H]^+$ Calcd for $C_{26}H_{37}NO_4P^+$ 458.2455, Found 458.2446.

tert-butyl(2*S*)-2-(3-(diphenylphosphoryl)-1-hydroxy-3-methylbutyl-4-d)pyrrolidine-1-carboxylate (2am-D)

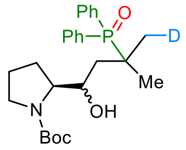

This compound was synthesized by **Standard conditions B**; Column chromatography conditions (PE/EA = 5:1 to EA), R_f = 0.10 (EA); White solid; 66 mg, 73%, dr = 1:1.3; ^{1}H NMR (400 MHz, $CDCl_3$): δ 8.00-7.93 (m, 4H), 7.54-7.50 (m, 6H), 5.52 (s, 1H), 3.96 (s, 1H), 3.76-3.61 (m, 1H), 3.45 (s, 1H), 3.24-3.20 (m, 1H), 2.08-1.60 (m, 6H), 1.41-1.25 (m, 14.16H, 84% D); ^{2}H NMR (92 MHz, $CHCl_3$): δ 1.29; ^{13}C NMR (100 MHz, $CDCl_3$): δ 155.4, 154.7, 132.2 (d, J_{C-P} = 8.0 Hz), 132.0 (d, J_{C-P} = 8.0 Hz), 131.6, 131.4, 130.3 (d, J_{C-P} = 91.8 Hz), 130.1 (d, J_{C-P} = 89.6 Hz), 129.8 (d, J_{C-P} = 92.6 Hz), 128.2, 128.1, 79.0, 68.2, 67.5, 63.1, 62.4, 47.2, 46.4, 43.9, 41.3, 36.6 (d, J_{C-P} = 68.0 Hz), 36.4 (d, J_{C-P} = 70.0 Hz), 28.3, 26.5, 26.4, 26.1, 24.6, 24.1 (t, J_{C-D} = 20.0 Hz), 23.0, 21.9, 21.4 (d, J_{C-D} = 20.0 Hz); ^{31}P NMR (162 MHz, $CDCl_3$): 42.4, 41.5; HRMS (ESI) m/z: $[M+H]^+$ Calcd for $C_{26}H_{36}DNO_4P^+$ 459.2517, Found 459.2504.

(4-cyclopropyl-4-hydroxy-2-methylbutan-2-yl)diphenylphosphine oxide (2an)

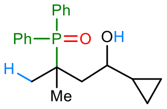

This compound was synthesized by **Standard conditions A**; Column chromatography conditions (PE/EA = 5:1 to 1:1), R_f = 0.20 (PE/EA = 1:1); White solid; 47 mg, 71%; ^{1}H NMR (400 MHz, $CDCl_3$): δ 8.03-7.94 (m,4H), 7.60-7.50 (m, 6H), 5.95 (s, 1H), 3.11 (t, J = 9.2 Hz, 1H), 2.11-2.02 (m, 1H), 1.77-1.67 (m, 1H), 1.32-1.24 (m, 6H), 0.89-0.81 (m, 1H), 0.55-0.50 (m, 1H), 0.48-0.35 (m, 2H), 0.09-0.03 (m, 1H); ^{13}C NMR (100 MHz, $CDCl_3$): δ 132.4 (d, J_{C-P} = 8.4 Hz), 132.2 (d, J_{C-P} = 8.1 Hz), 131.73 (d, J_{C-P} = 2.6 Hz), 131.69 (d, J_{C-P} = 2.5 Hz), 130.0 (d, J_{C-P} = 91.6 Hz), 129.8 (d, J_{C-P} = 90.4 Hz), 128.4 (d, J_{C-P} = 11.1 Hz), 128.3 (d, J_{C-P} = 11.0 Hz), 71.8 (d, J_{C-P} = 2.0 Hz), 47.8, 36.8 (d, J_{C-P} = 68.0 Hz), 26.3 (d, J_{C-P} = 2.0 Hz), 22.0, 18.4, 3.6, 2.4; ^{31}P NMR (162 MHz, $CDCl_3$): 42.5; HRMS (ESI) m/z: $[M+H]^+$ Calcd for $C_{20}H_{26}O_2P^+$ 329.1665, Found 329.1654.

(4-bromobutan-2-yl)diphenylphosphine oxide (3a)

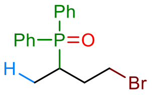

The title compound was synthesized by the following process.[4] To a stirred solution of compound **2a** (0.2 mmol, 1.0 equiv.) in CH_2Cl_2 (2 mL), PPh_3 (2.0 equiv.) and CBr_4 (2.0 equiv.) were added. The mixture was further stirred at room temperature until **2a** was consumed completely as monitored by TLC analysis, and then the solution was concentrated under reduced pressure. The crude product was purified by column chromatography to give the corresponding product **3a**.

Column chromatography conditions (PE/EA = 20:1 to 5:1), R_f = 0.20 (PE/EA = 5:1); White solid; 50.6 mg, 75%; ^{1}H NMR (400 MHz, $CDCl_3$): δ 7.86-7.78 (m, 4H), 7.53-7.48 (m, 6H), 3.58-3.53 (m, 1H), 3.48-3.42 (m, 1H), 2.78-2.71 (m, 1H), 2.18-2.10 (m, 1H), 2.09-1.99 (m, 1H), 1.19-1.13 (m, 3H); ^{13}C NMR (100 MHz, $CDCl_3$): δ 132.0 (d, J_{C-P} = 95.8 Hz), 131.8 (d, J_{C-P} = 2.1 Hz), 131.7 (d, J_{C-P} = 1.9 Hz), 131.7 (d, J_{C-P} = 94.1 Hz), 130.99 (d, J_{C-P} = 8.4 Hz), 130.96 (d, J_{C-P} = 8.8 Hz), 128.8 (d, J_{C-P} = 11.4 Hz), 128.7 (d, J_{C-P} = 11.0 Hz), 31.9 (d, J_{C-P} = 15.0 Hz), 31.7, 30.1 (d, J_{C-P} = 72.0 Hz), 11.1 (d, J_{C-P} = 3.0 Hz); ^{31}P NMR (162 MHz, $CDCl_3$): δ 36.3; HRMS (ESI) m/z: $[M+H]^+$ Calcd for $C_{16}H_{19}BrOP^+$ 337.0351, Found 337.0340.

(4-azidobutan-2-yl)diphenylphosphine oxide (3b)

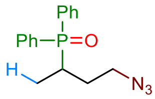

The title compound was synthesized by the following process.[5] To a stirred solution of compound **2a** (0.2 mmol, 1.0 equiv.) in dry THF (2 mL), PPh_3 (1.3 equiv.) and diisopropyl azodicarboxylate (DIAD, 1.3 equiv.) were added, and then diphenylphosphoryl azide (DPPA) (1.3 equiv.) was added dropwise at 0 °C. The mixture was further stirred at room temperature until the **2a** was consumed completely as monitored by TLC analysis, and then the solution was concentrated under reduced pressure. The crude product was purified by column chromatography to give the corresponding product **3b**.

Column chromatography conditions (PE/EA = 20:1 to 5:1), R_f = 0.10 (PE/EA = 5:1); White solid; 47.4 mg, 79%; ^{1}H NMR (400 MHz, $CDCl_3$): δ 7.85-7.78 (m, 4H), 7.55-7.45 (m, 6H), 3.49-3.44 (m, 1H), 3.36-3.29 (m, 1H), 2.63-2.54 (m, 1H), 2.00-1.89 (m, 1H), 1.76-1.66 (m, 1H), 1.20-1.15 (m, 3H); ^{13}C NMR (100 MHz, $CDCl_3$): δ131.8 (d, J_{C-P} = 95.5 Hz), 131.75 (d, J_{C-P} = 3.0 Hz),131.73 (d, J_{C-P} =94.7 Hz), 131.6 (d, J_{C-P} = 2.4 Hz), 131.3 (d, J_{C-P} = 9.8 Hz), 130.93 (d, J_{C-P} = 8.6 Hz), 130.91 (d, J_{C-P} = 8.6 Hz), 128.8 (d, J_{C-P} = 11.5 Hz), 128.6 (d, J_{C-P} = 11.5 Hz), 48.9 (d, J_{C-P} = 13.0 Hz), 29.0 (d, J_{C-P} = 73.0 Hz), 28.4, 11.7 (d, J_{C-P} = 3.0 Hz); ^{31}P NMR (162 MHz, $CDCl_3$): δ 36.4; HRMS (ESI) m/z: $[M+H]^+$ Calcd for $C_{16}H_{19}N_3OP^+$ 300.1260, Found 300.1247.

3-(diphenylphosphoryl)butanal (3c)

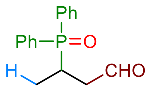

The title compound was synthesized by the following process.[6] To a stirred solution of compound **2a** (0.2 mmol, 1.0 equiv.) in CH_2Cl_2 (2 mL) was added pyridinium chlorochromate (PCC, 2.0 equiv.). The mixture was further stirred at room temperature until **2a** was consumed completely as monitored by TLC analysis, and then the solution was concentrated under reduced pressure. The crude product was purified by flash column chromatography (gradient eluent of ethyl acetate and petroleum ether) to give the corresponding product **3c**.

Column chromatography conditions (PE/EA = 20:1 to 5:1), R_f = 0.10 (PE/EA = 4:1); White solid; 37.7 mg, 69%; ^{1}H NMR (400 MHz, $CDCl_3$): δ 9.72 (s, 1H), 7.81 (m, 4H), 7.48 (m, 6H), 3.11 (s, 1H), 2.72 (s, 2H), 1.18-1.16 (m, 3H); ^{13}C NMR (100 MHz, $CDCl_3$): δ 199.4 (d, $J_{C\text{-}P}$ = 14.0 Hz), 131.8 (d, $J_{C\text{-}P}$ = 9.2 Hz), 130.91 (d, $J_{C\text{-}P}$ = 9.4 Hz), 130.89 (d, $J_{C\text{-}P}$ = 9.6 Hz), 130.88 (d, $J_{C\text{-}P}$ = 98.9 Hz), 130.8 (d, $J_{C\text{-}P}$ = 9.2 Hz), 128.7 (d, $J_{C\text{-}P}$ = 13.4 Hz), 128.5 (d, $J_{C\text{-}P}$ = 11.6 Hz), 43.5, 26.2 (d, $J_{C\text{-}P}$ = 71.0 Hz), 13.0; ^{31}P NMR (162 MHz, $CDCl_3$): 36.9; HRMS (ESI) m/z: $[M+H]^+$ Calcd for $C_{16}H_{18}O_2P^+$ 273.1039, Found 273.1028.

9 Copies of ^{1}H, ^{2}H, ^{13}C, ^{19}F and ^{31}P NMR spectra

^{1}H NMR (400 MHz, $CDCl_3$)

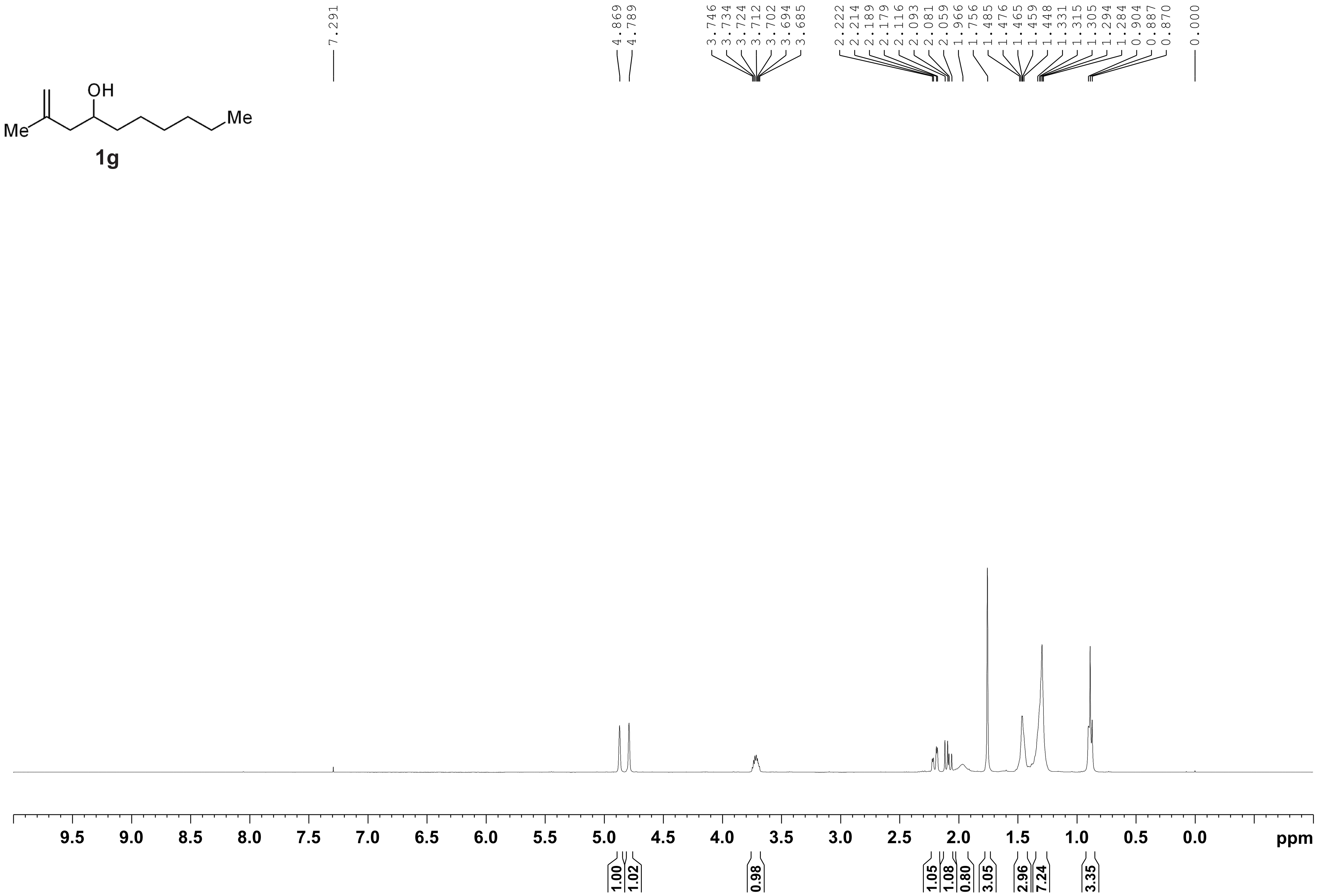

1g

^{13}C NMR (100.6 MHz, $CDCl_3$)

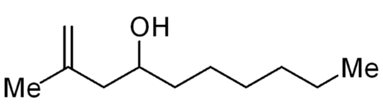

1g

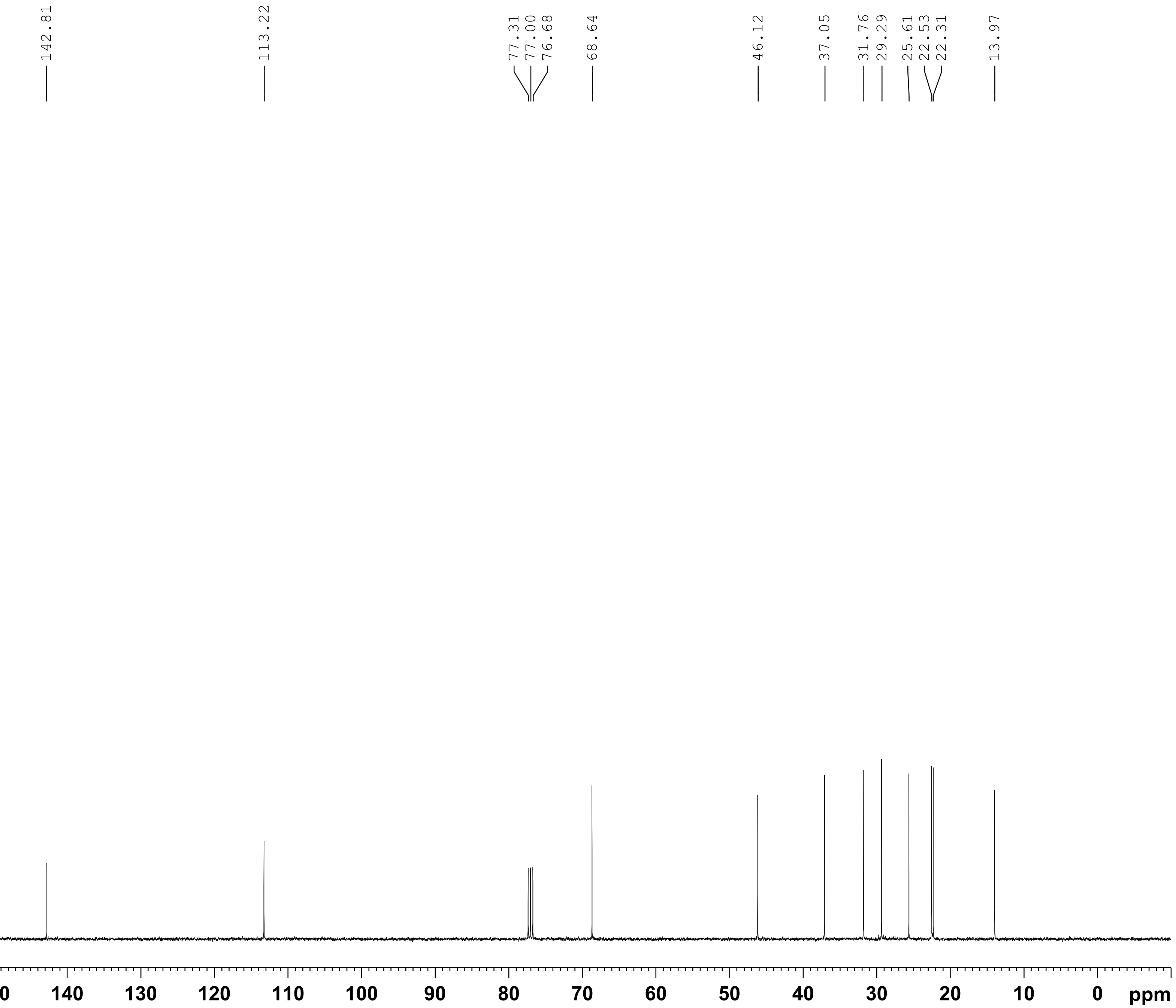

^{1}H NMR (400 MHz, $CDCl_3$)

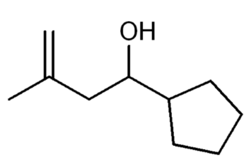

1i

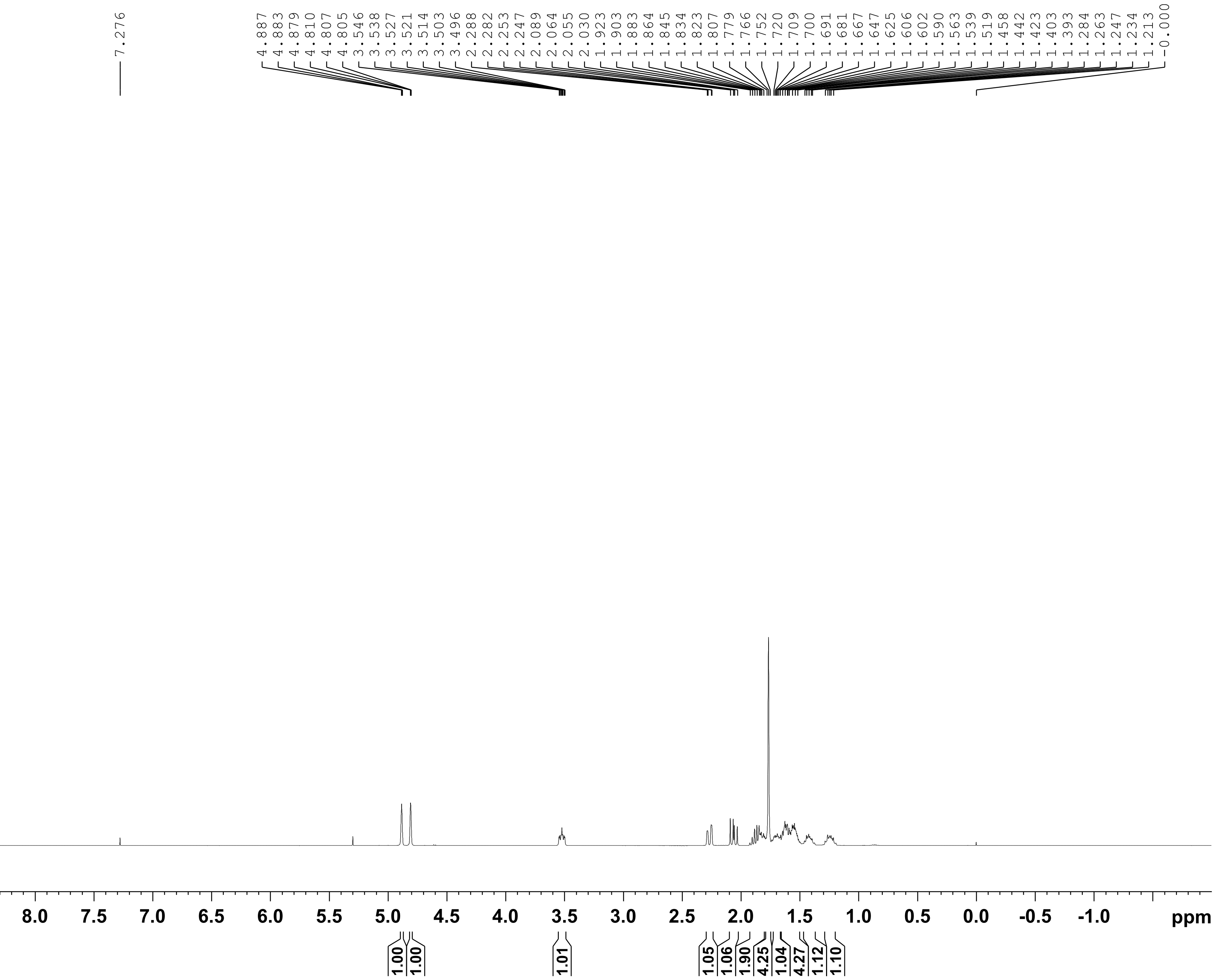

^{13}C NMR (100.6 MHz, CDCl$_3$)

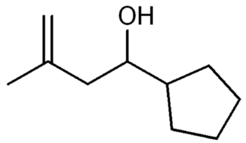

1i

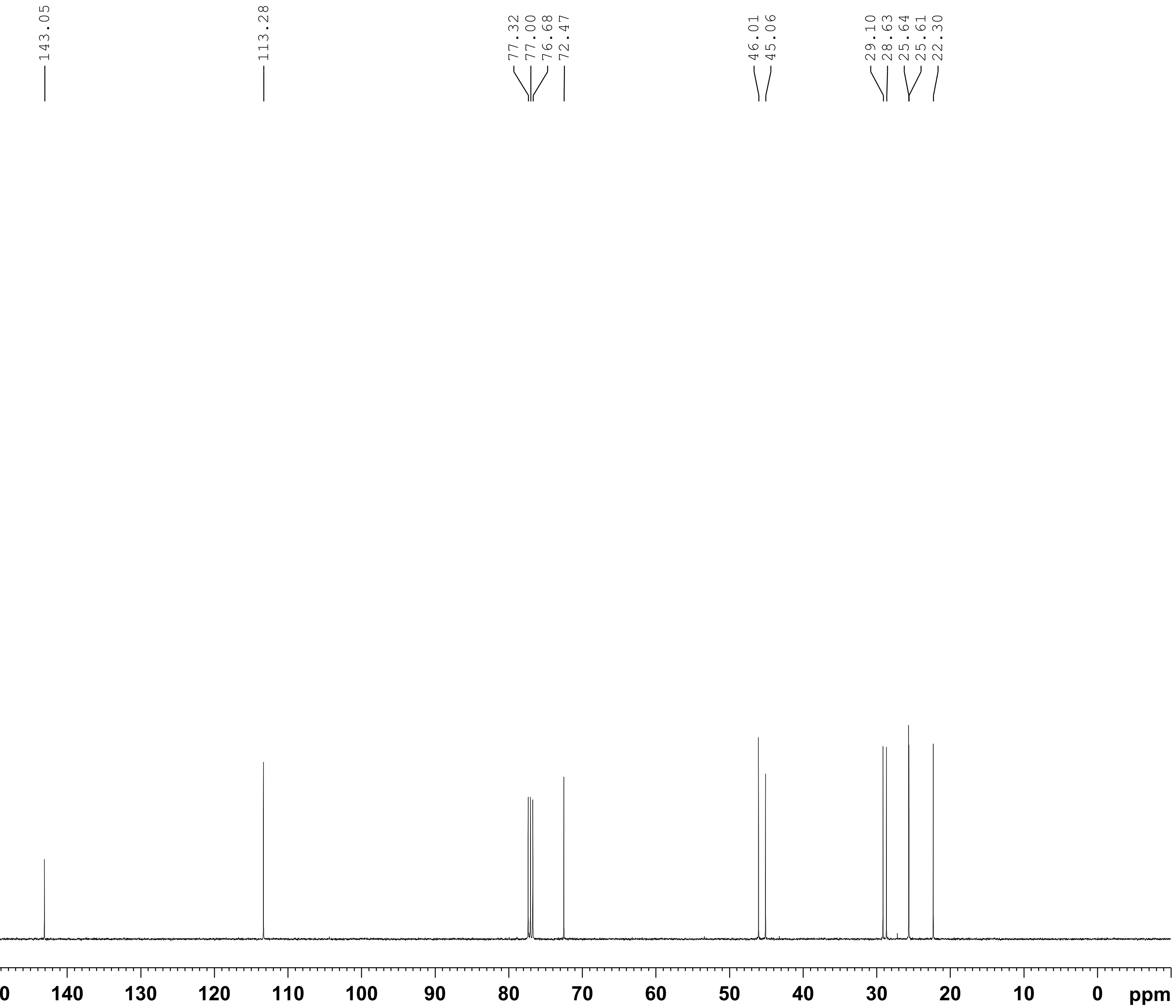

^{1}H NMR (400 MHz, $CDCl_3$)

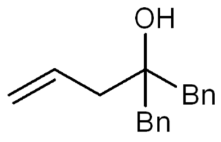

1m

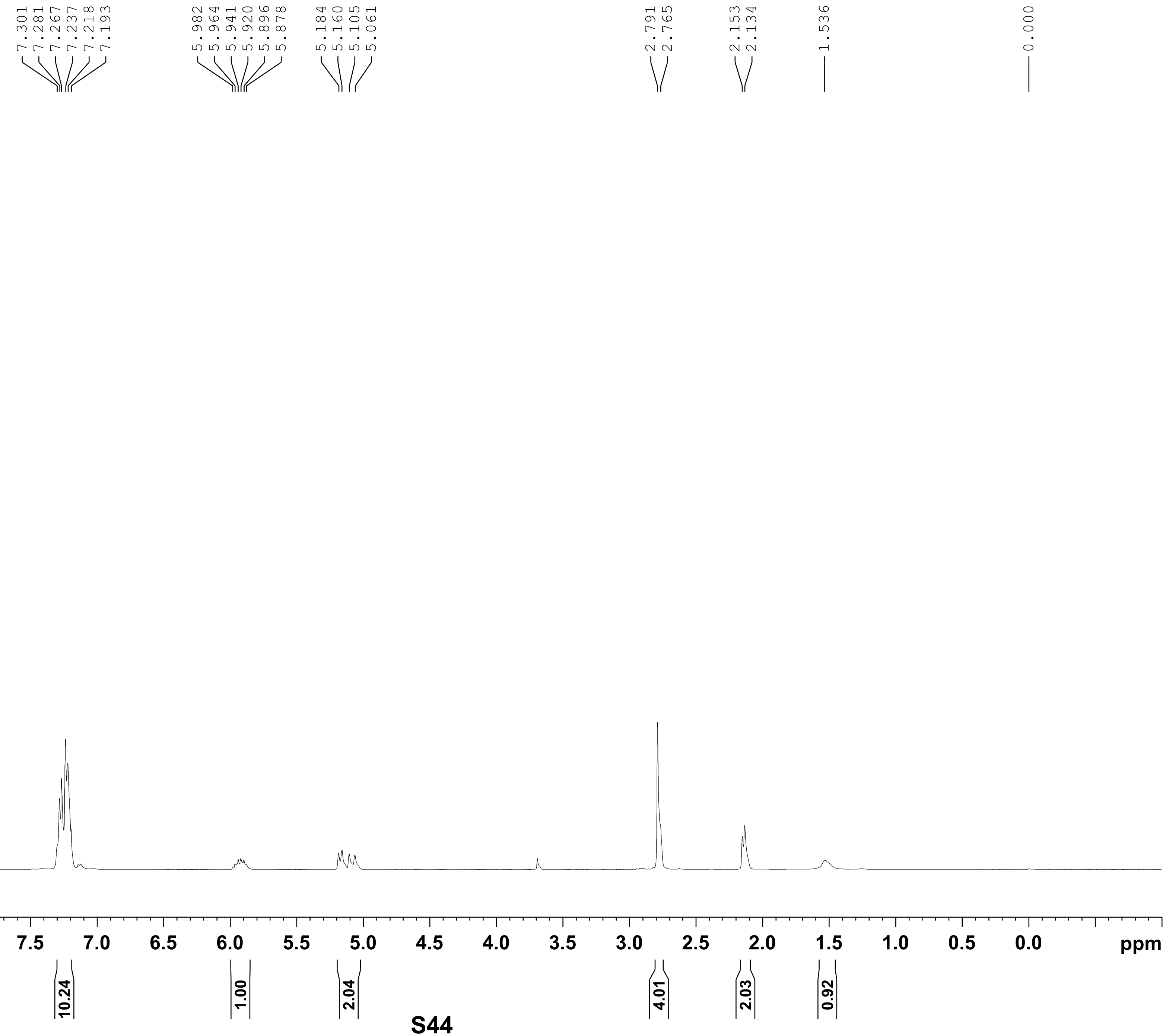

^{13}C NMR (100.6 MHz, $CDCl_3$)

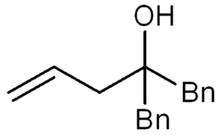

1m

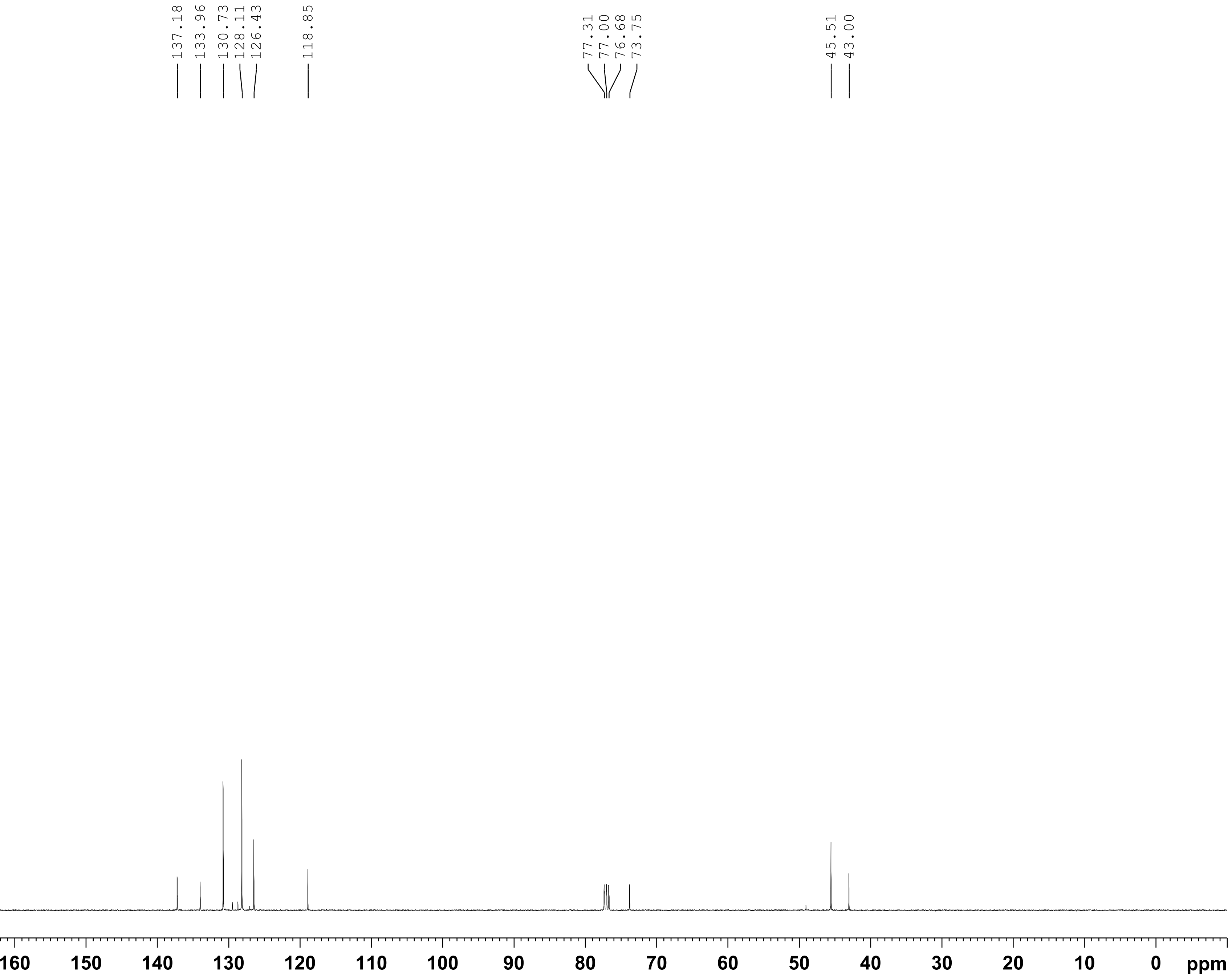

^{1}H NMR (400 MHz, $CDCl_3$)

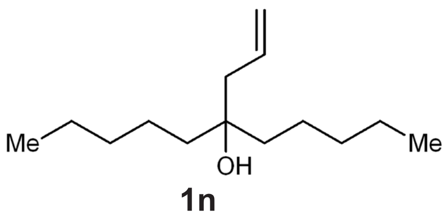

1n

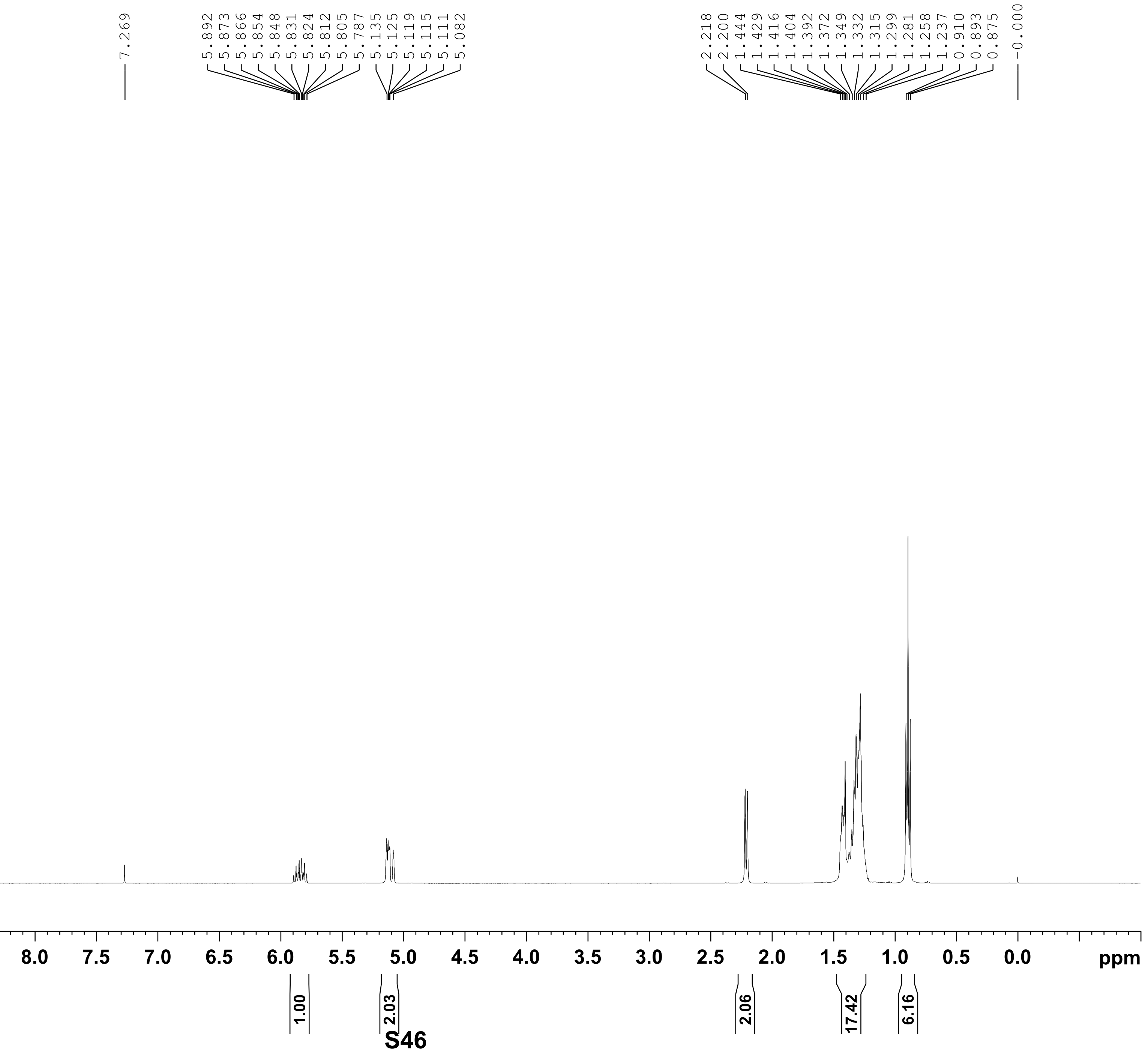

^{13}C NMR (100.6 MHz, $CDCl_3$)

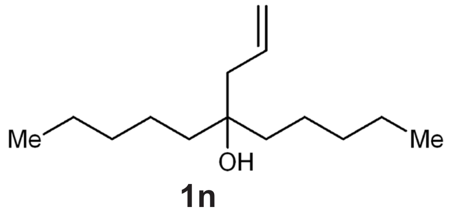

1n

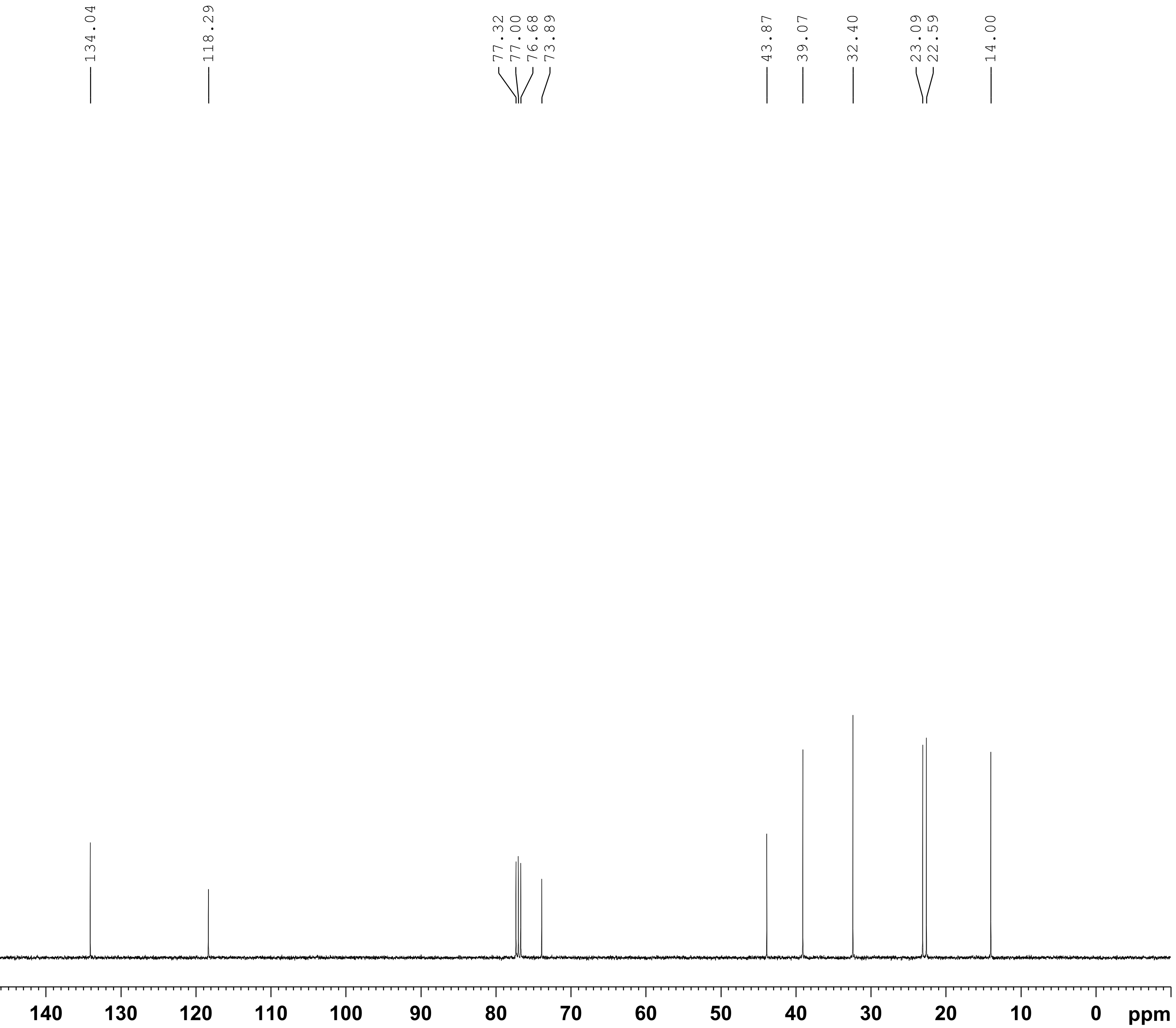

^{1}H NMR (400 MHz, $CDCl_3$)

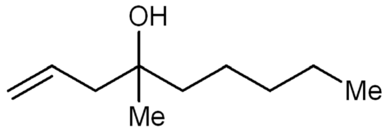

1o

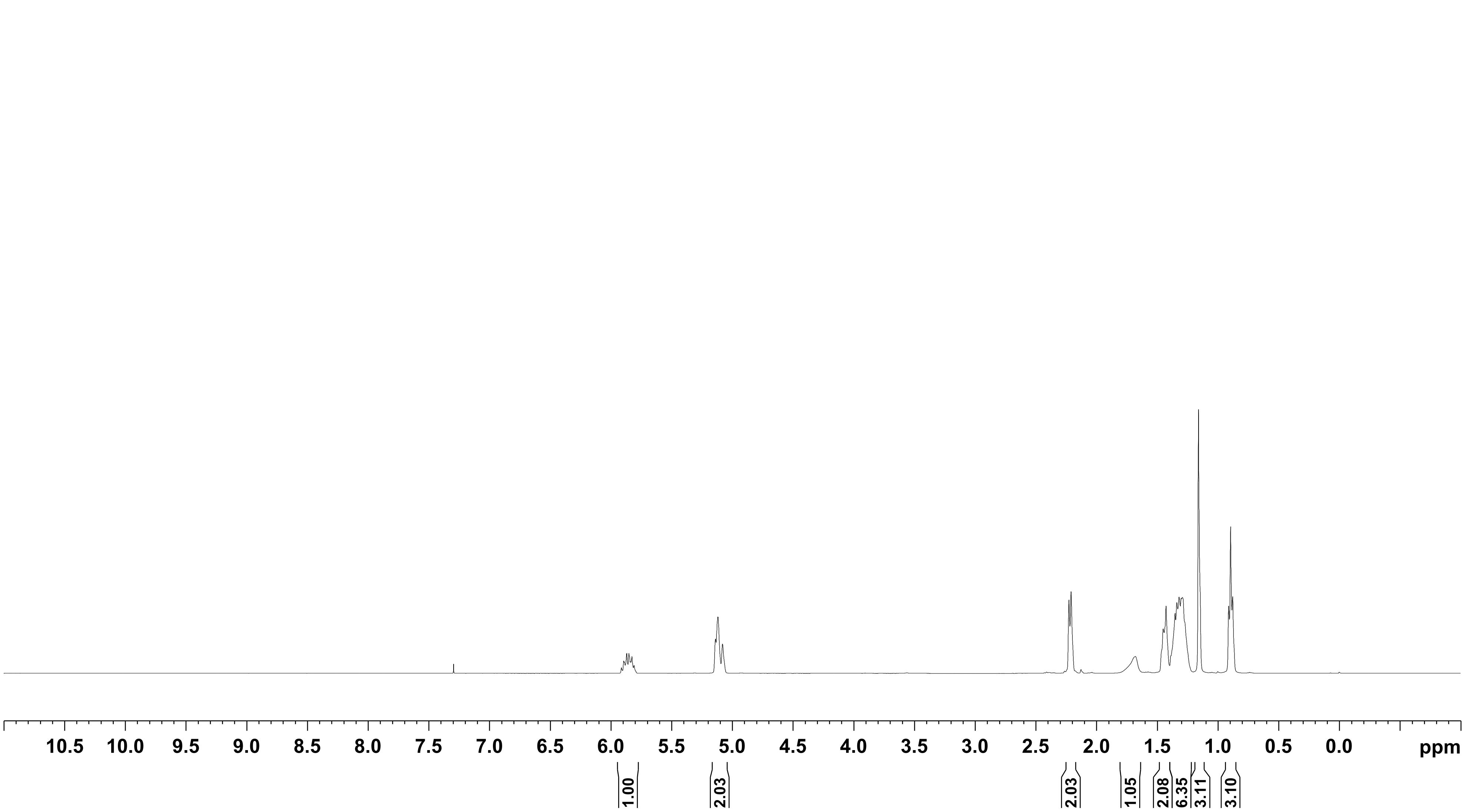

^{13}C NMR (100.6 MHz, $CDCl_3$)

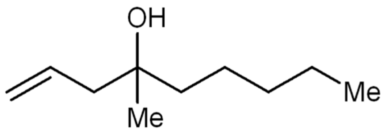

1o

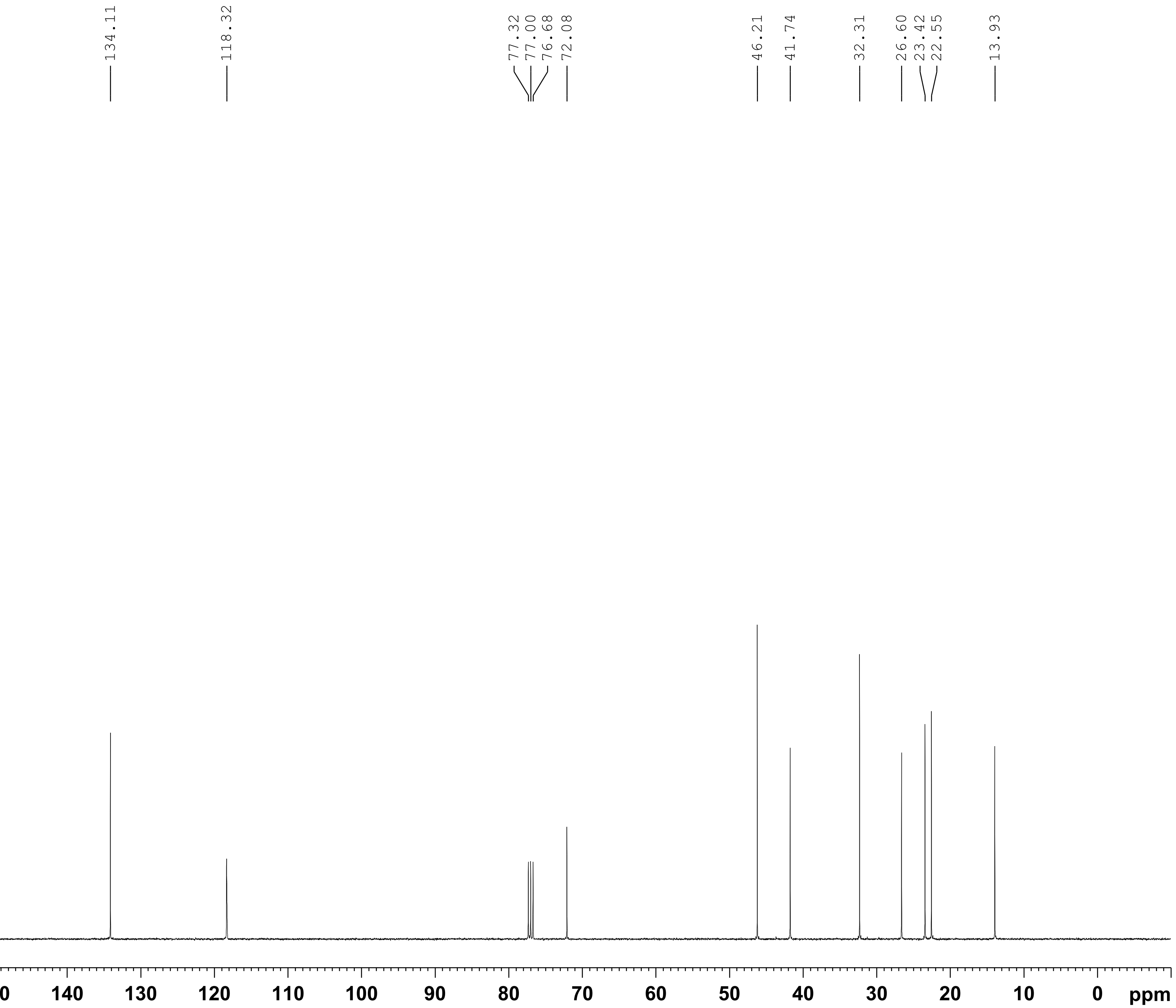

^{1}H NMR (400 MHz, $CDCl_3$)

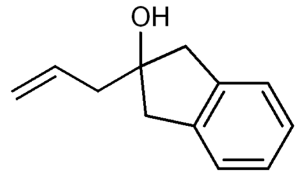

1z

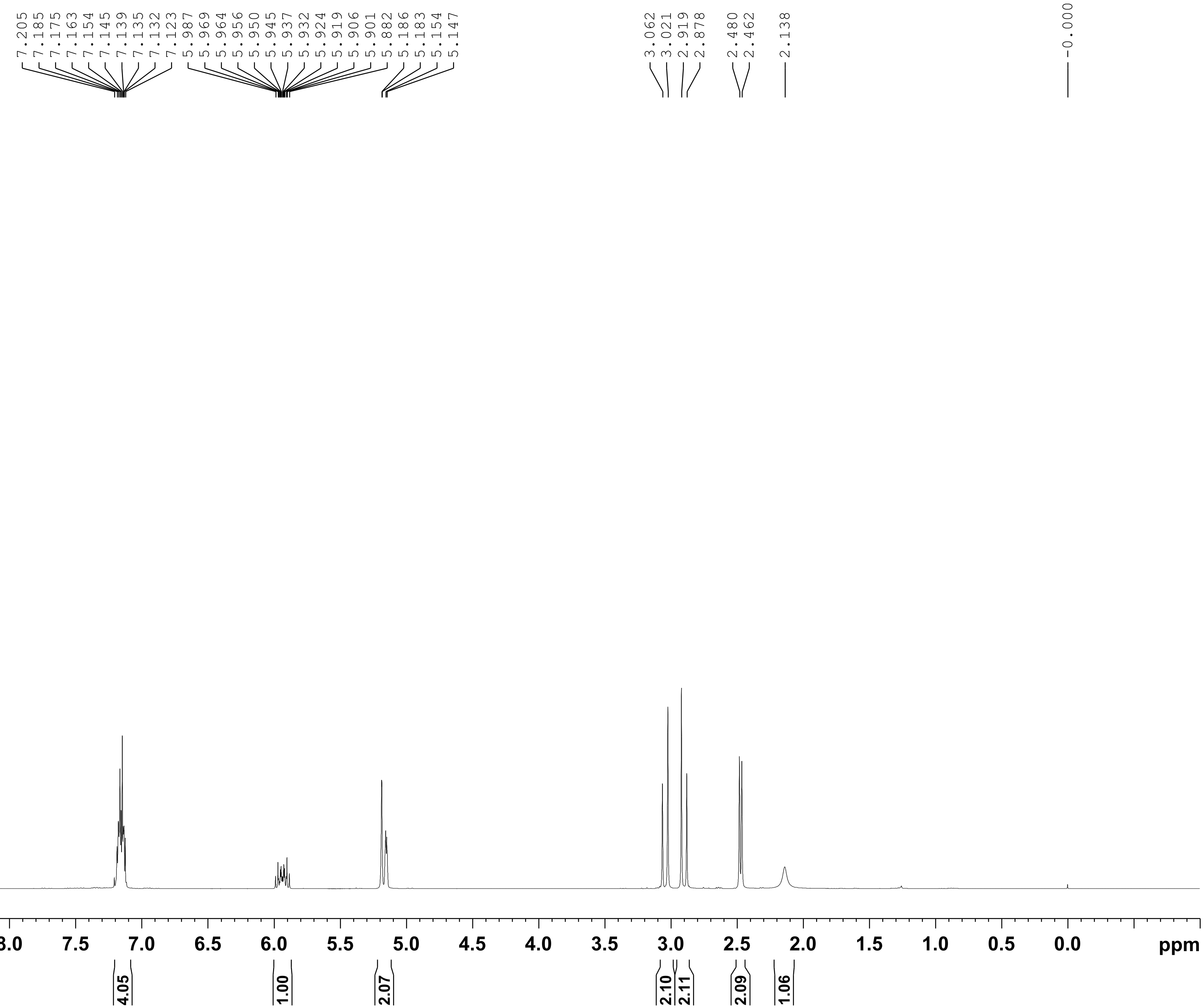

^{13}C NMR (100.6 MHz, $CDCl_3$)

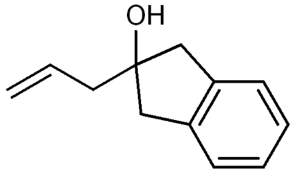

1z

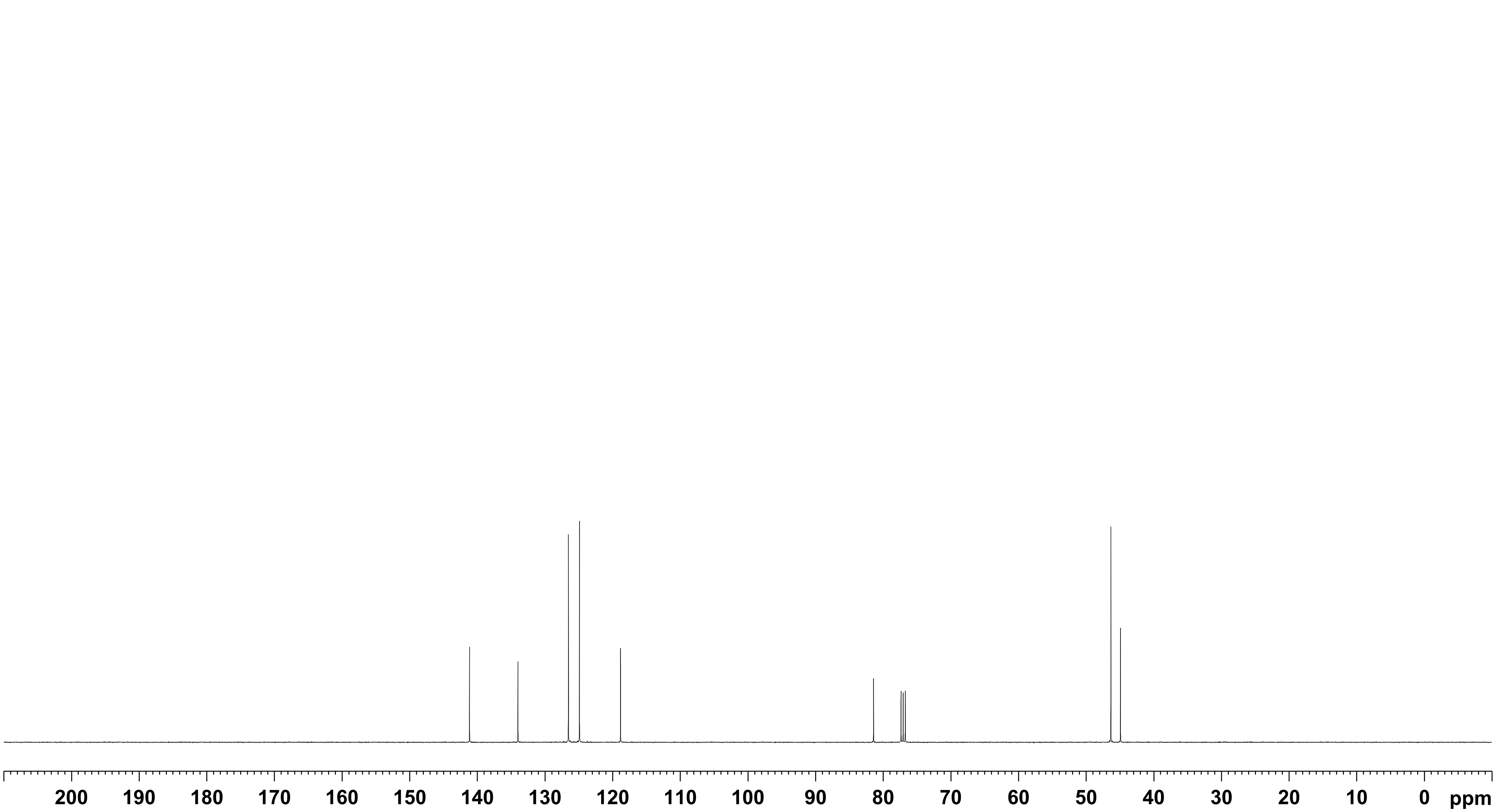

^{1}H NMR (400 MHz, $CDCl_3$)

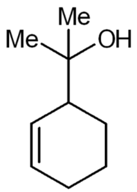

1ae

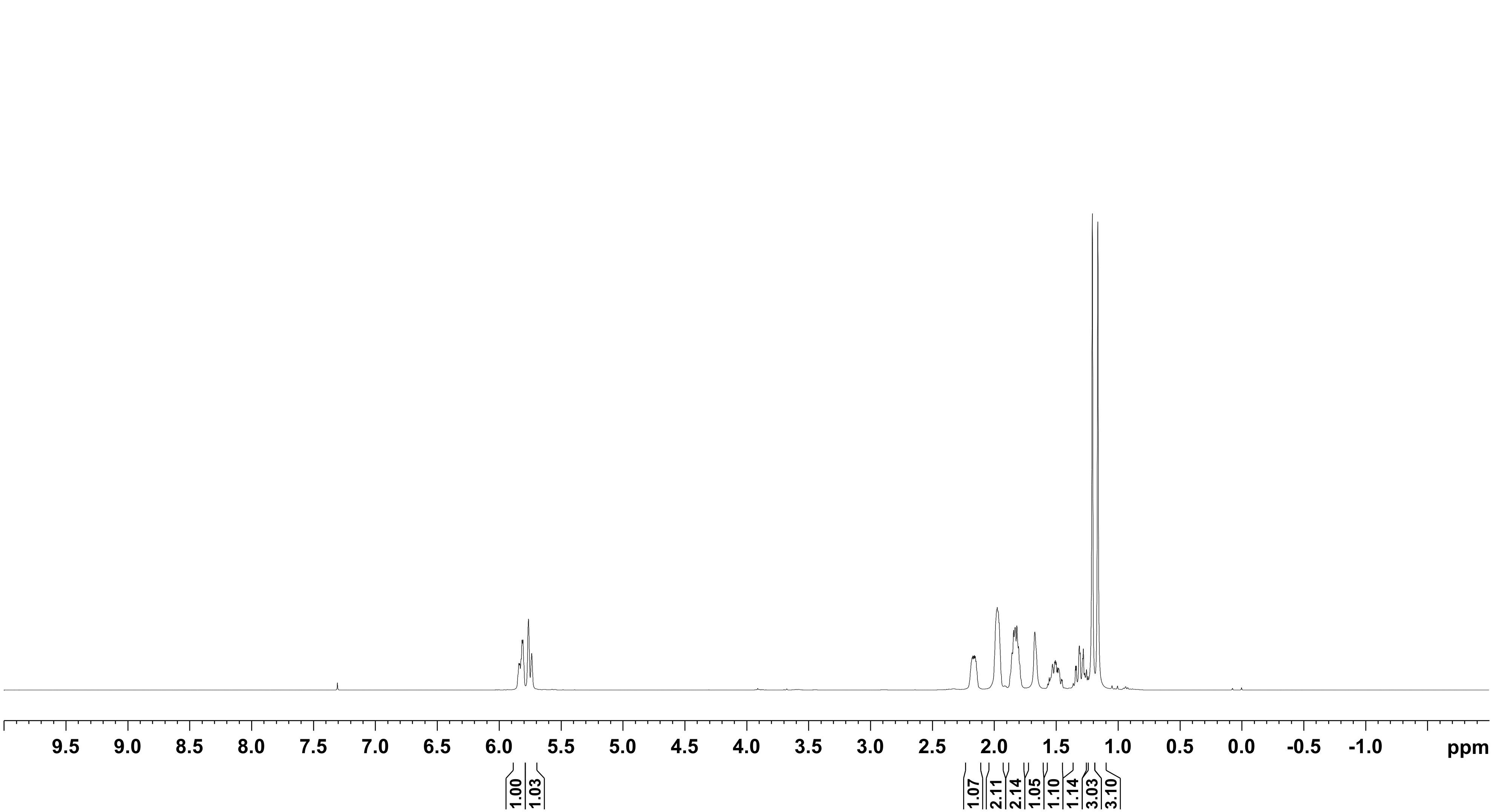

^{13}C NMR (100.6 MHz, $CDCl_3$)

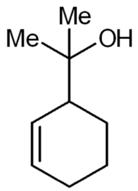

1ae

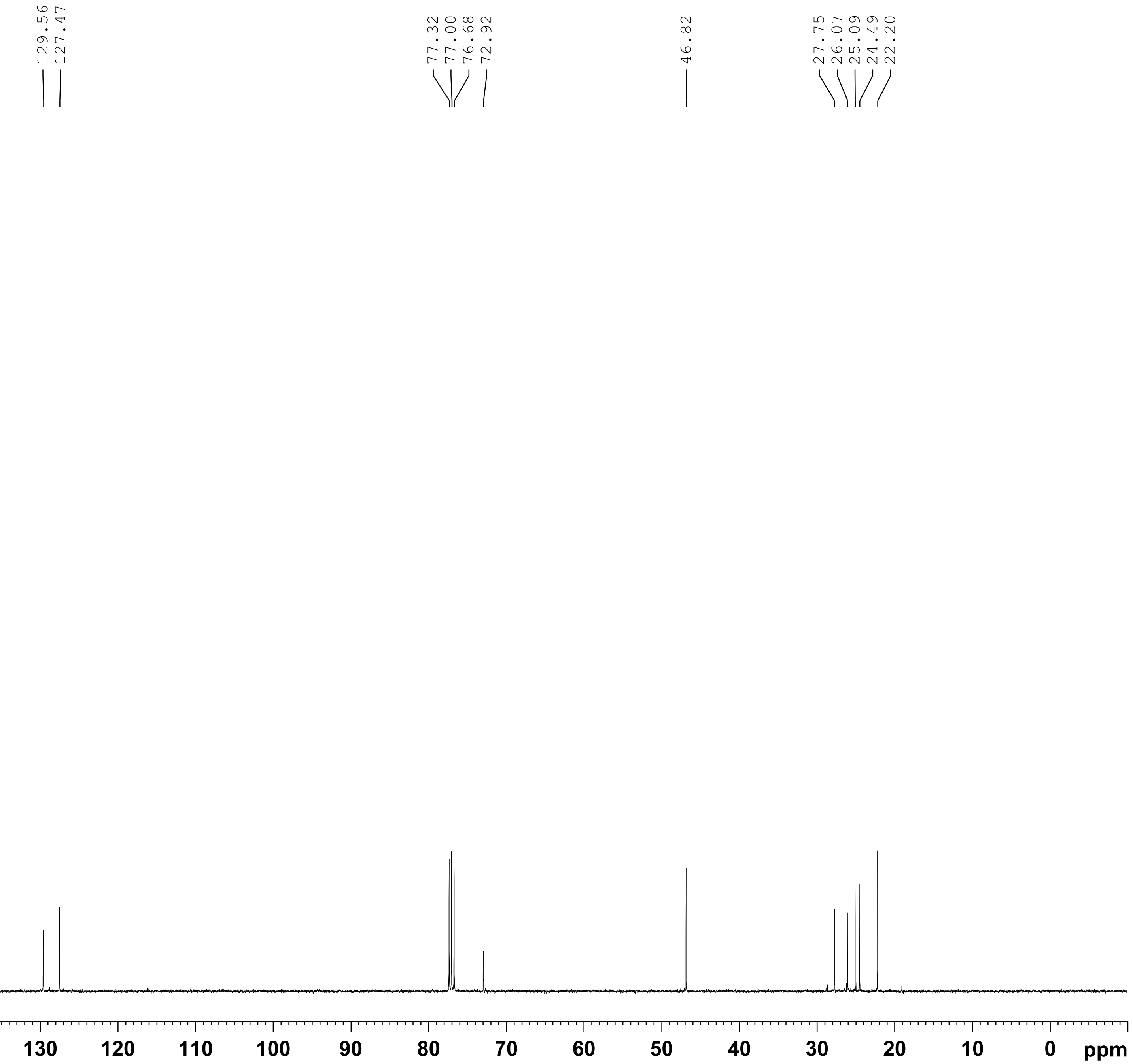

^{1}H NMR (400 MHz, $CDCl_3$)

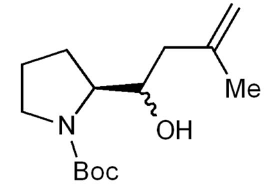

1ag

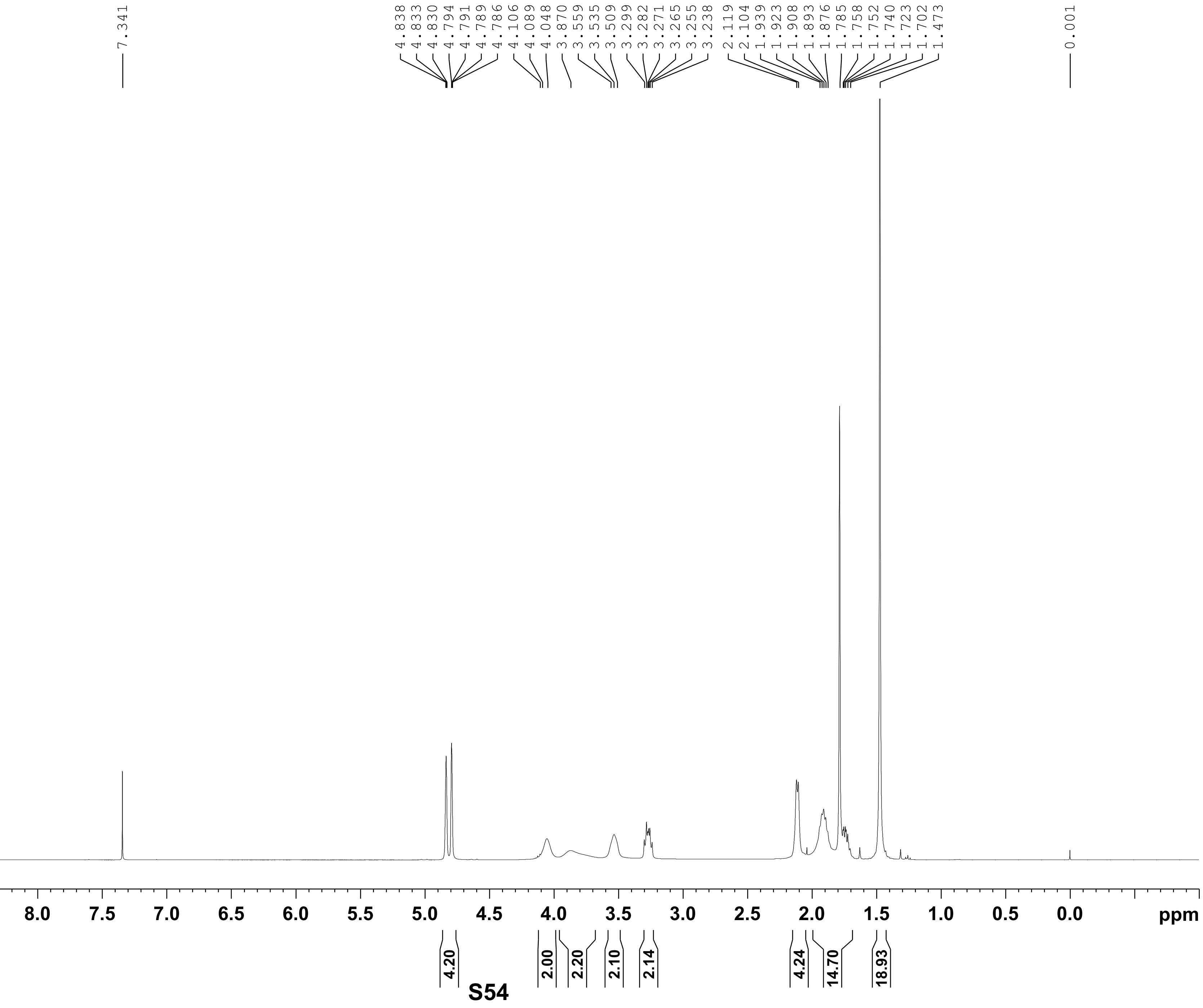

^{13}C NMR (100.6 MHz, $CDCl_3$)

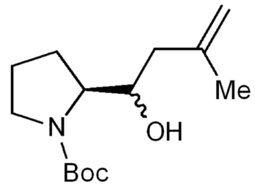

1ag

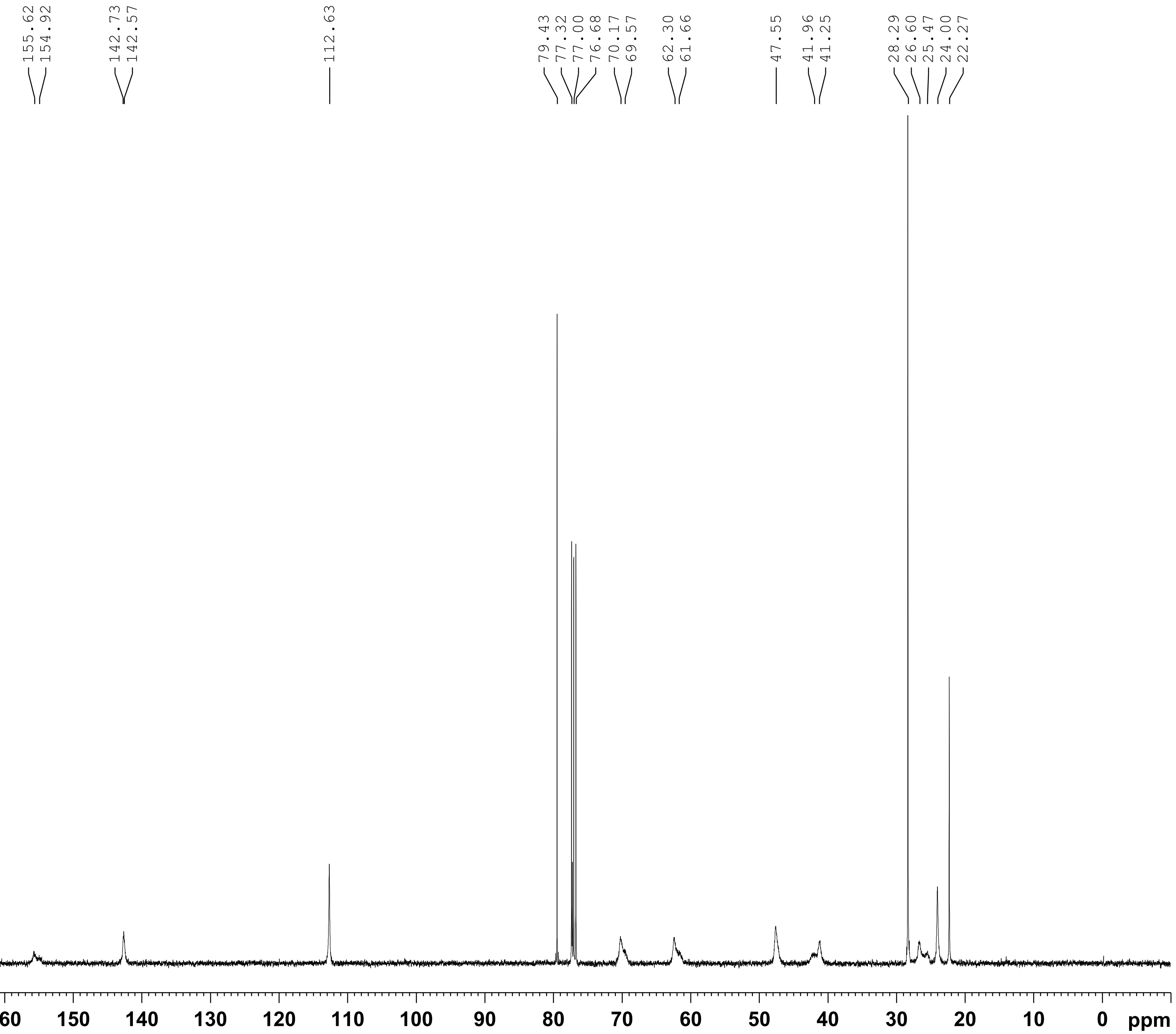

^{1}H NMR (400 MHz, $CDCl_3$)

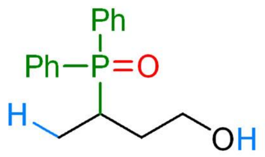

2a

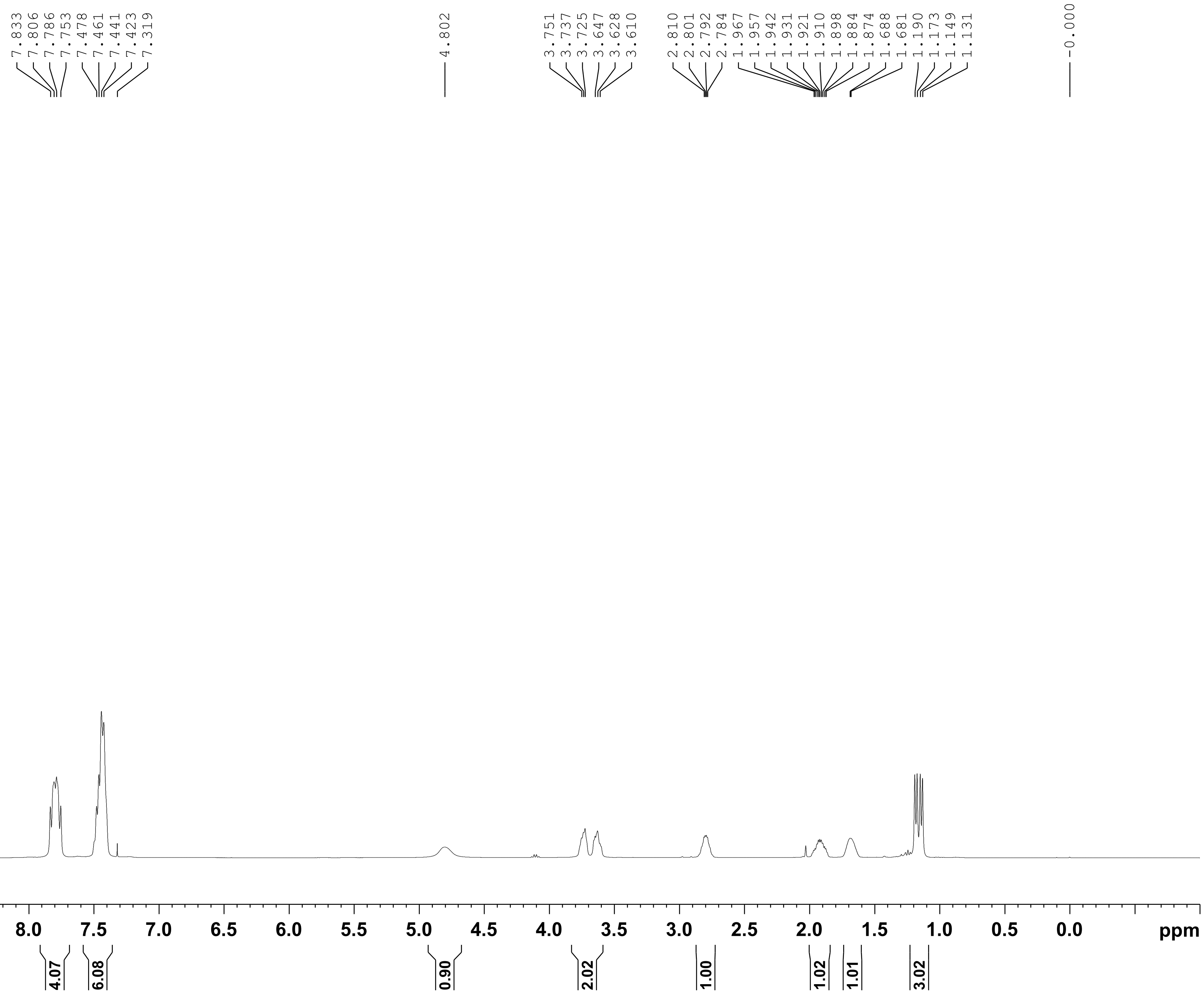

^{13}C NMR (100.6 MHz, $CDCl_3$)

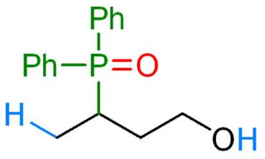

2a

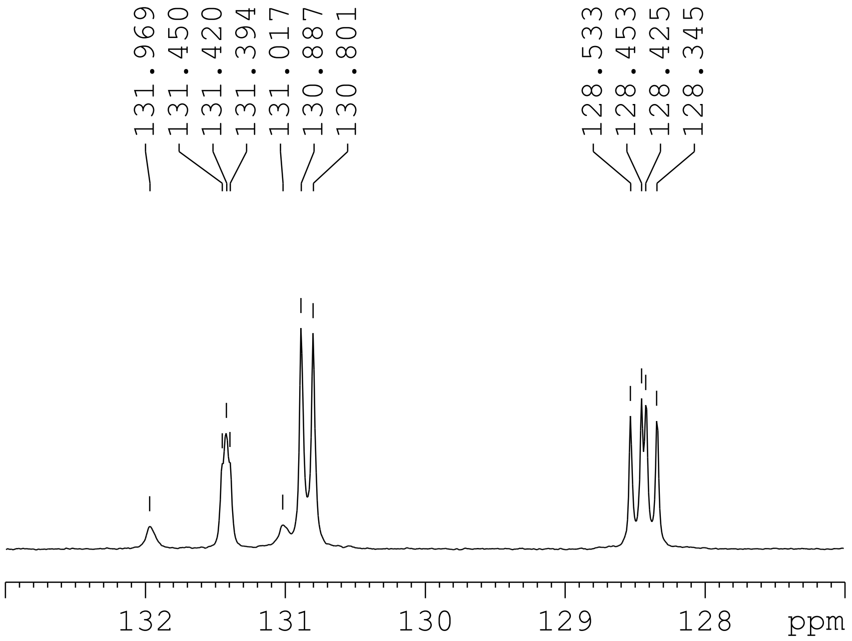

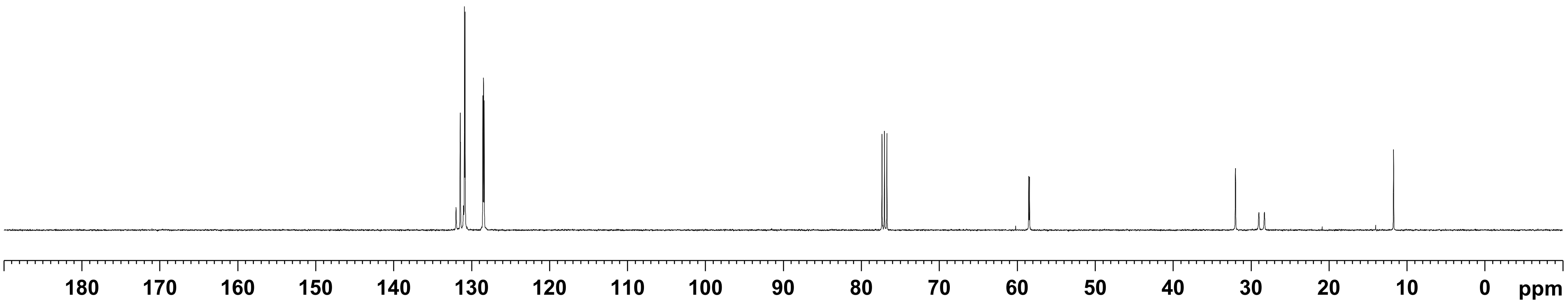

^{31}P NMR (162 MHz, $CDCl_3$)

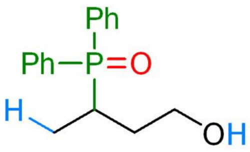

2a

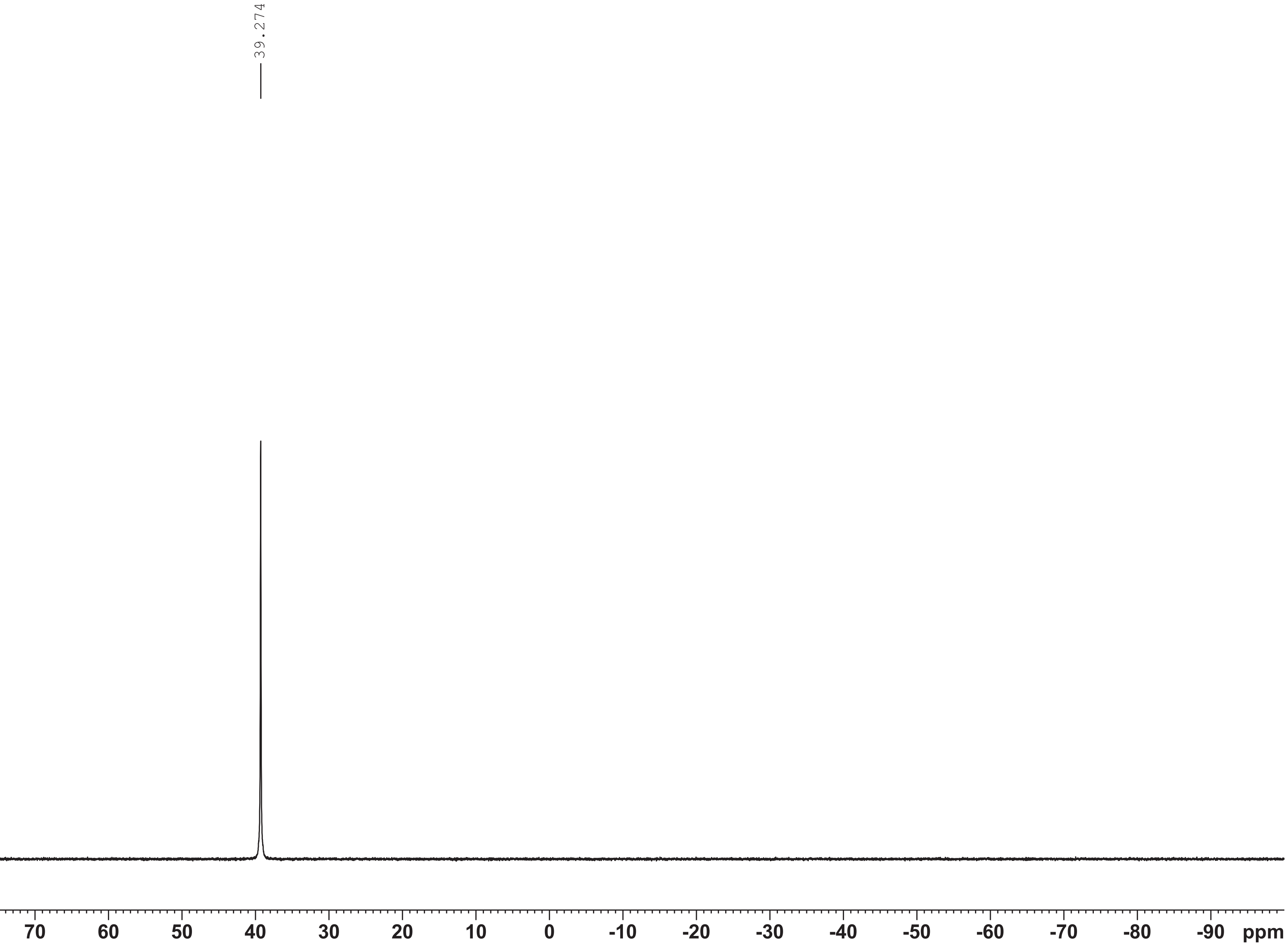

^{1}H NMR (400 MHz, $CDCl_3$)

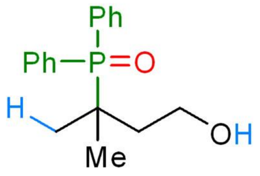

2b

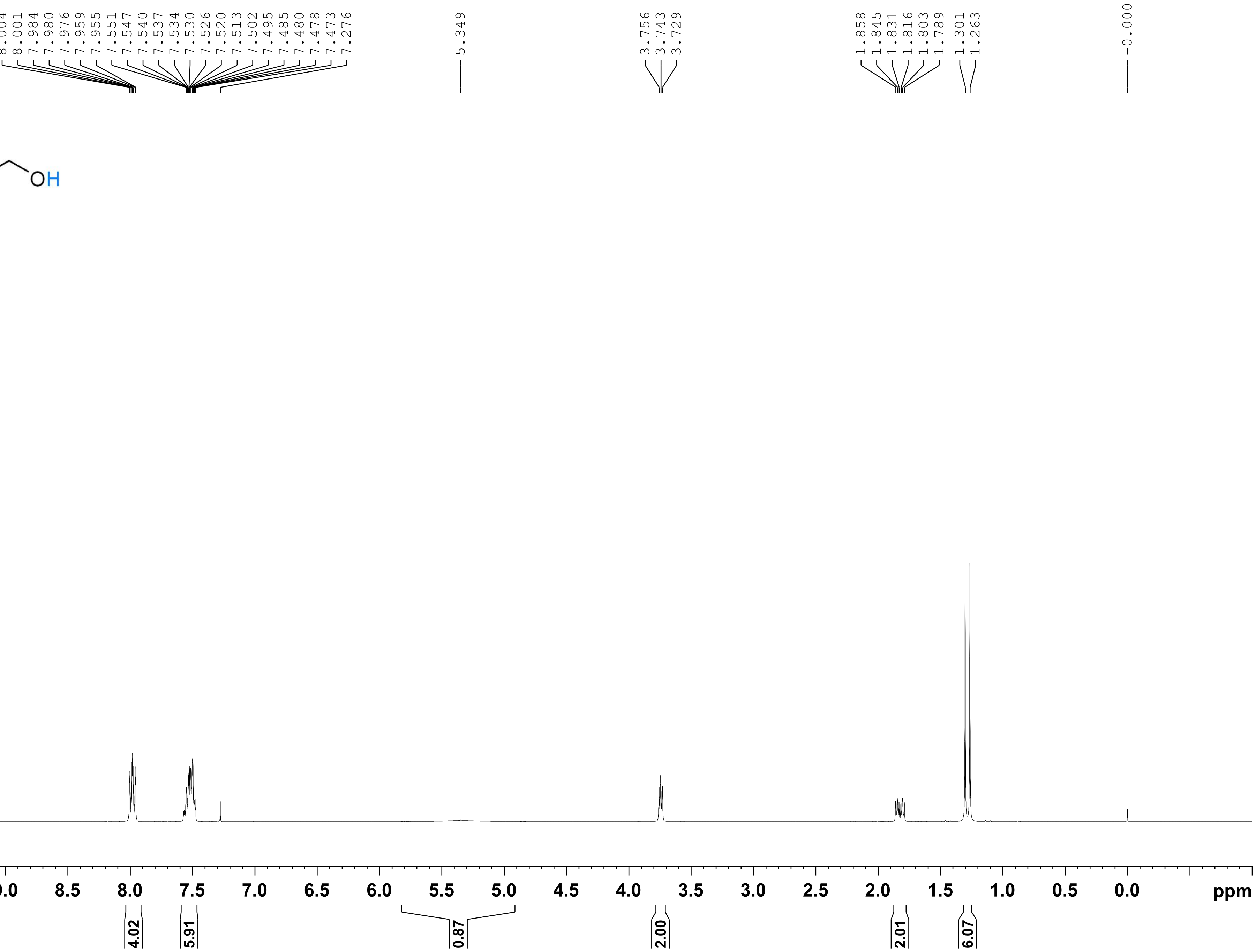

^{13}C NMR (100.6 MHz, $CDCl_3$)

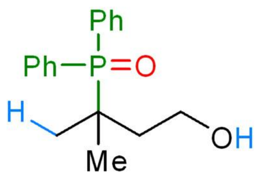

2b

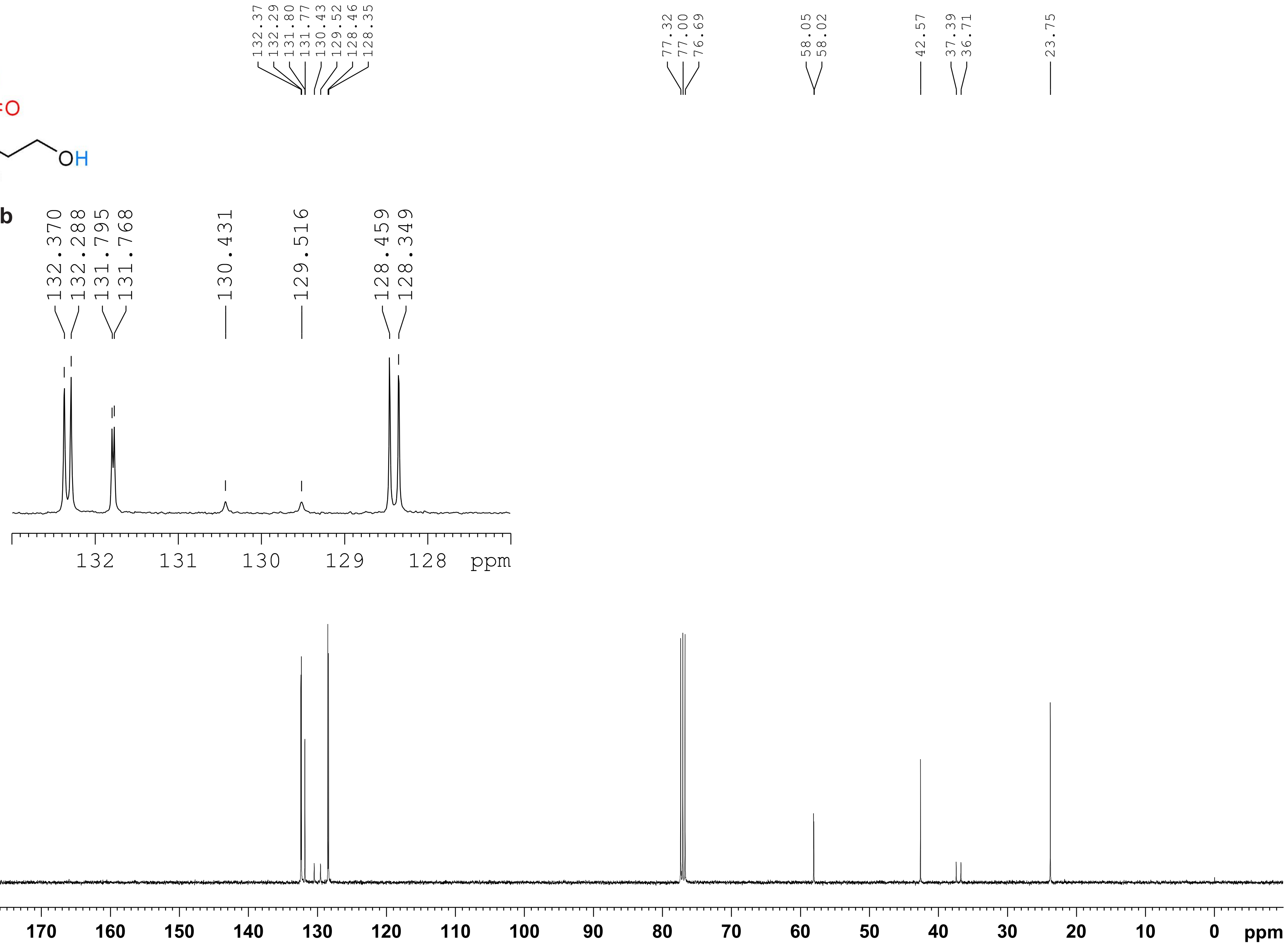

^{31}P NMR (162 MHz, $CDCl_3$)

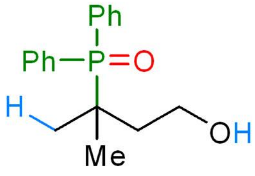

2b

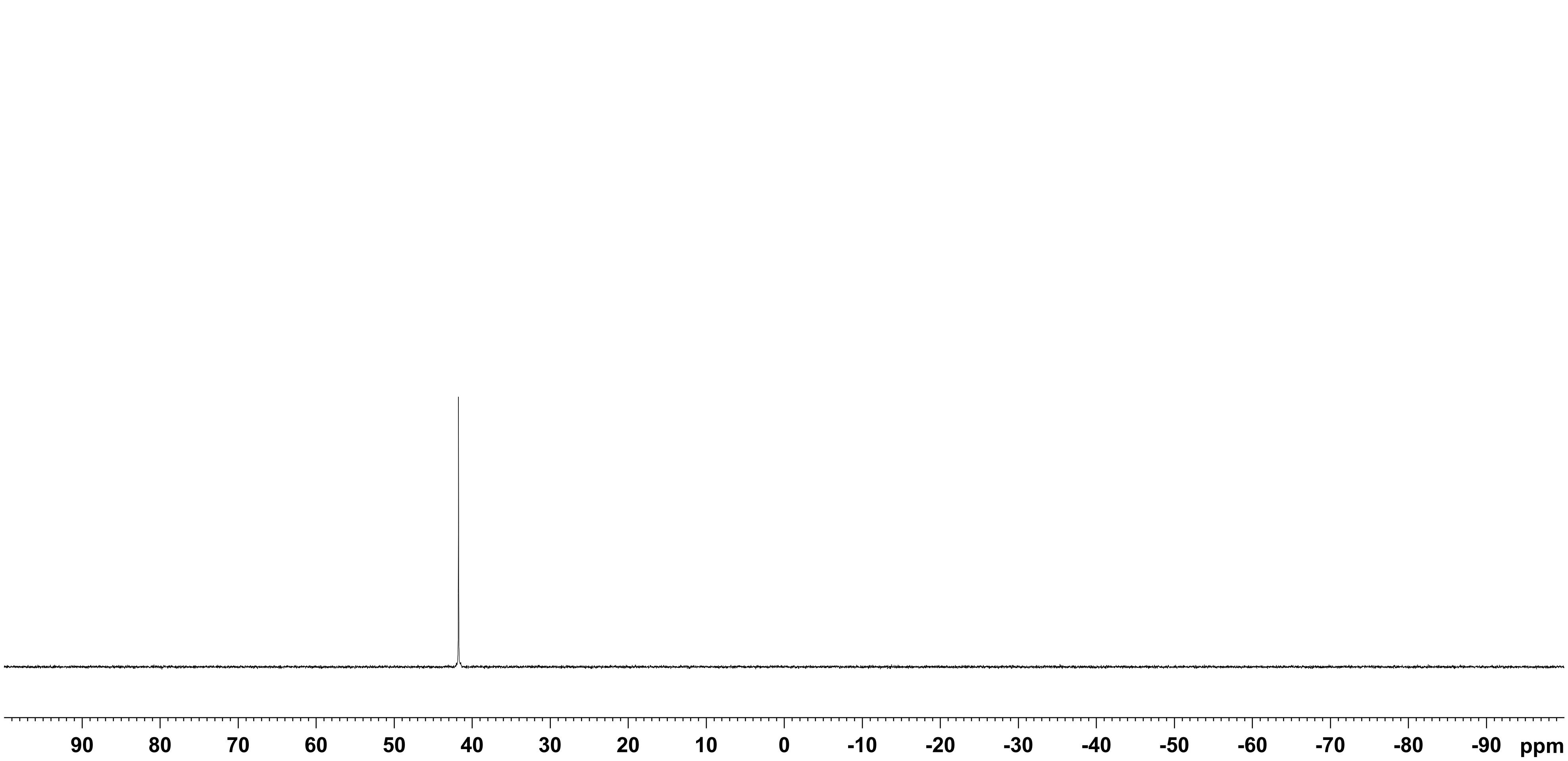

^{1}H NMR (400 MHz, $CDCl_3$)

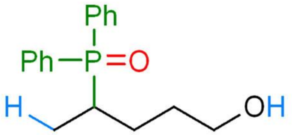

2c

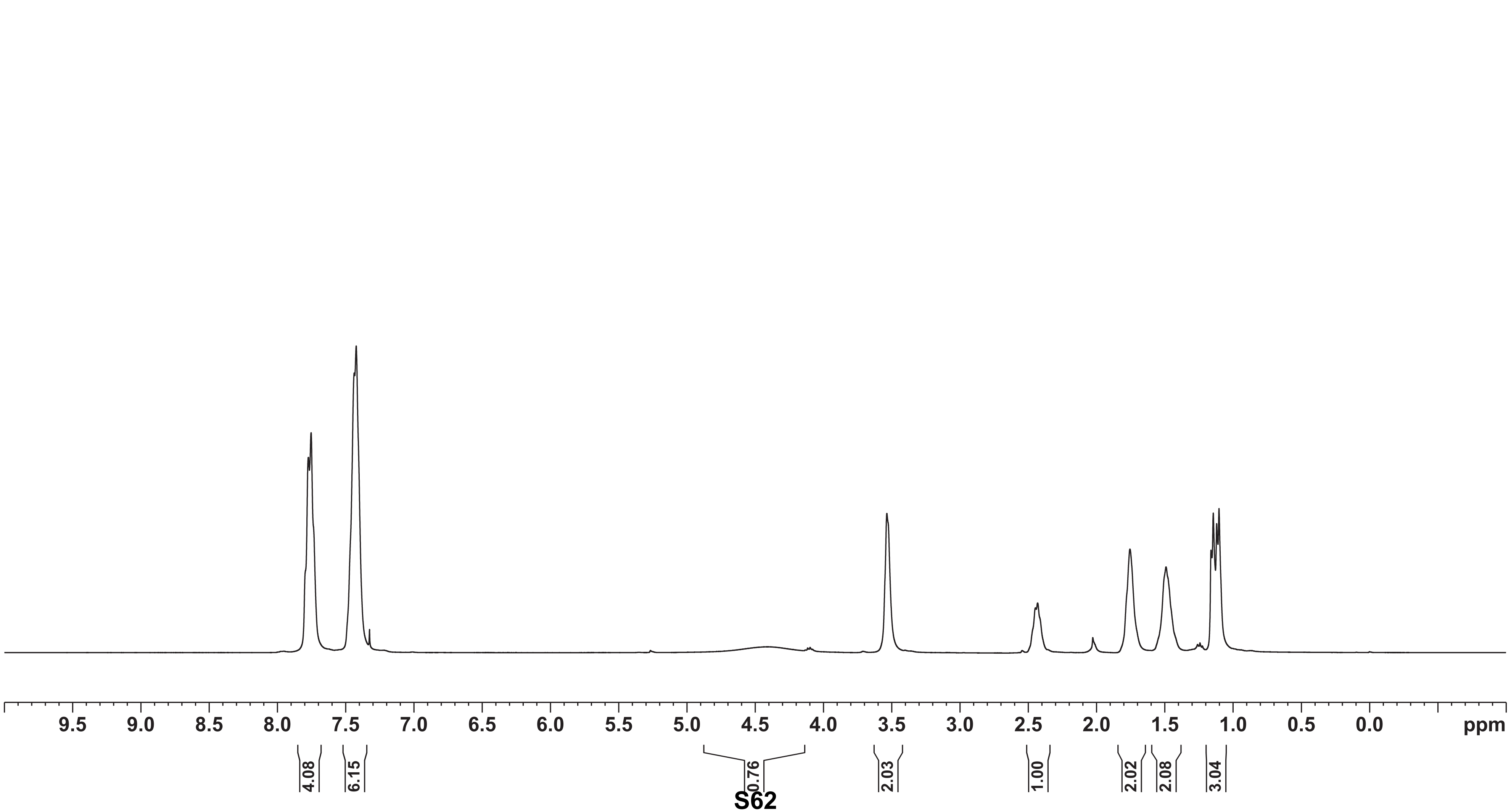

^{13}C NMR (100.6 MHz, CDCl$_3$)

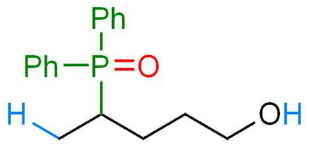

2c

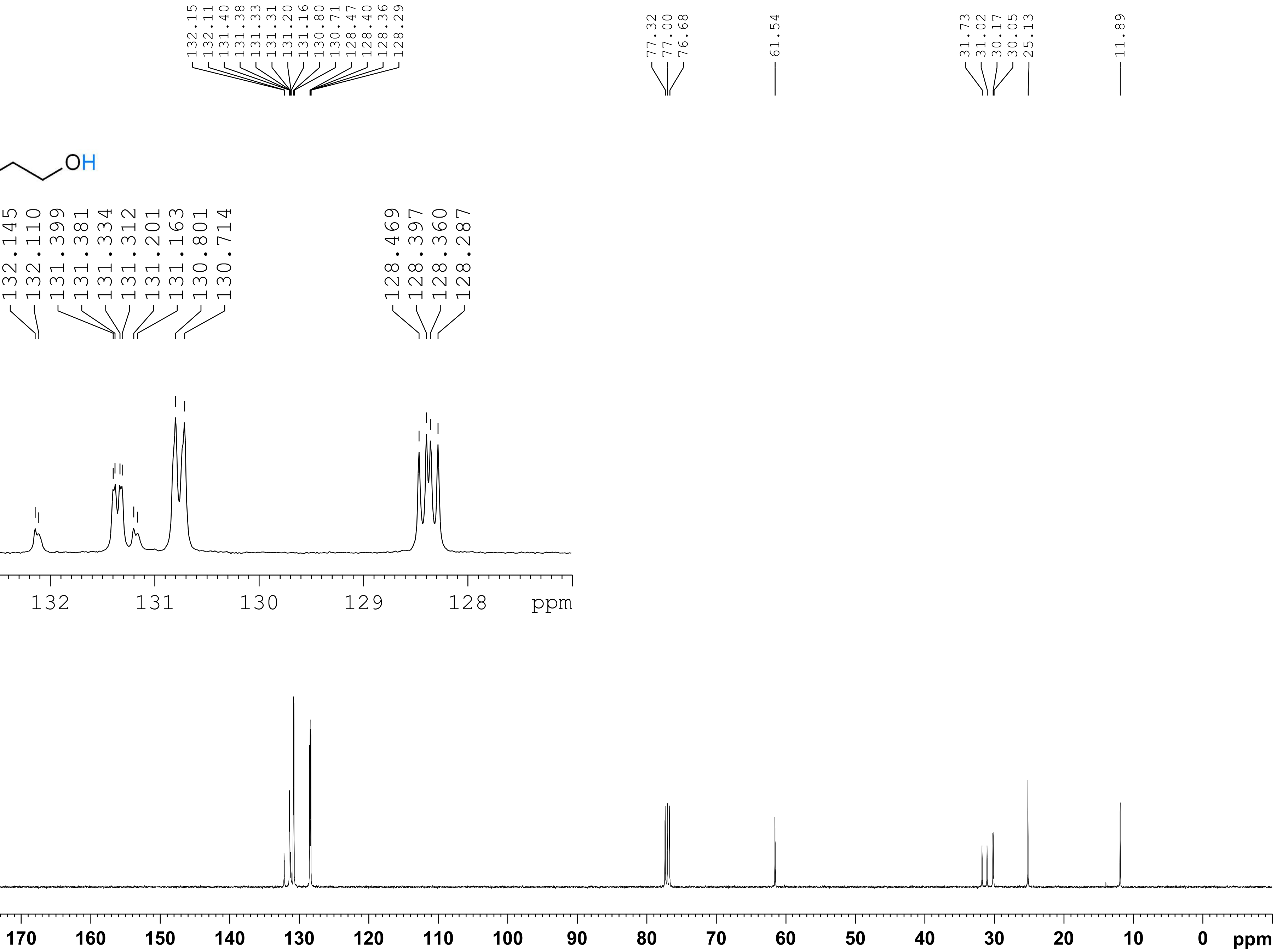

^{31}P NMR (162 MHz, $CDCl_3$)

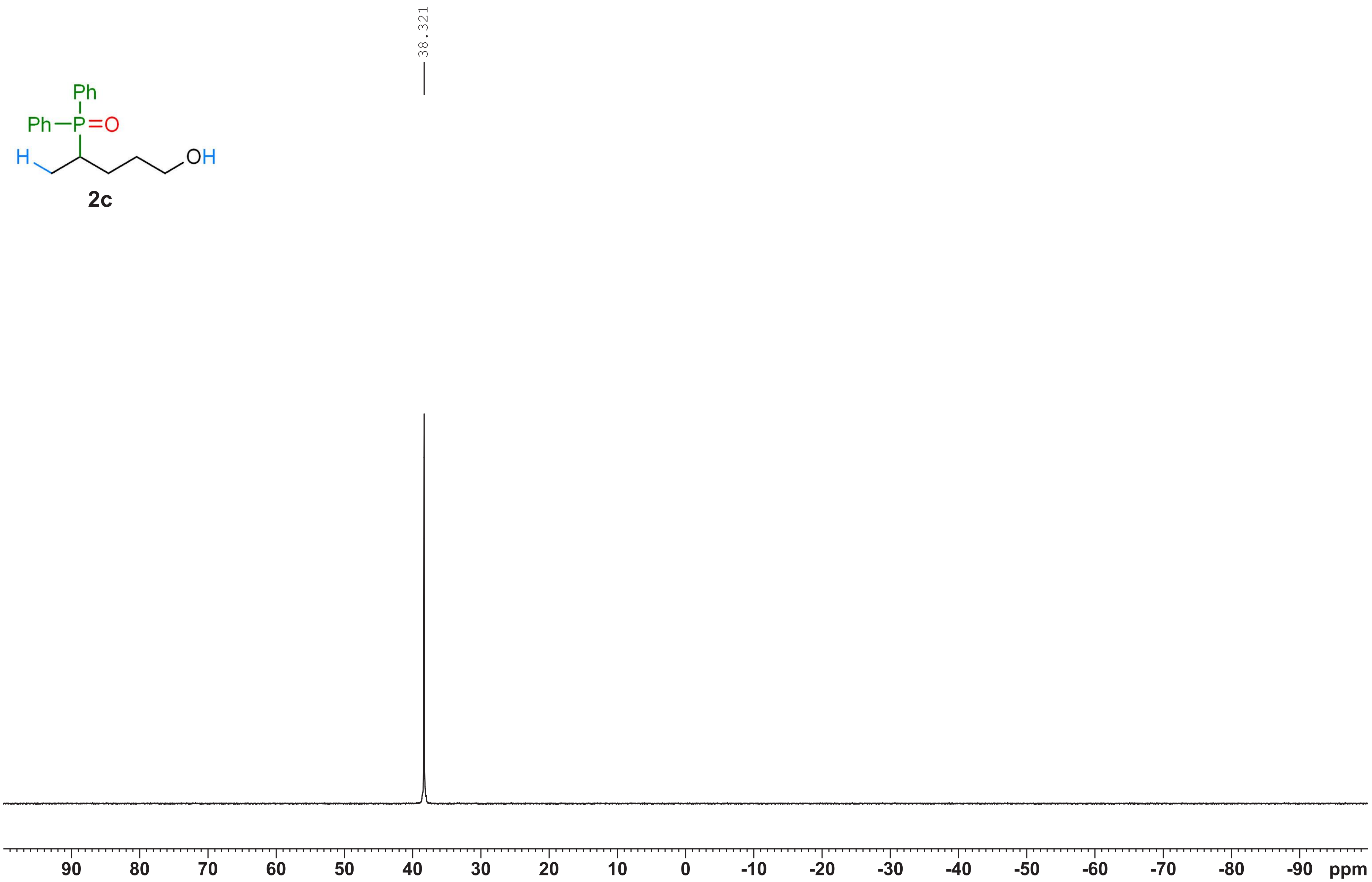

^{1}H NMR (400 MHz, $CDCl_3$)

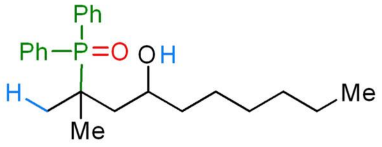

2g

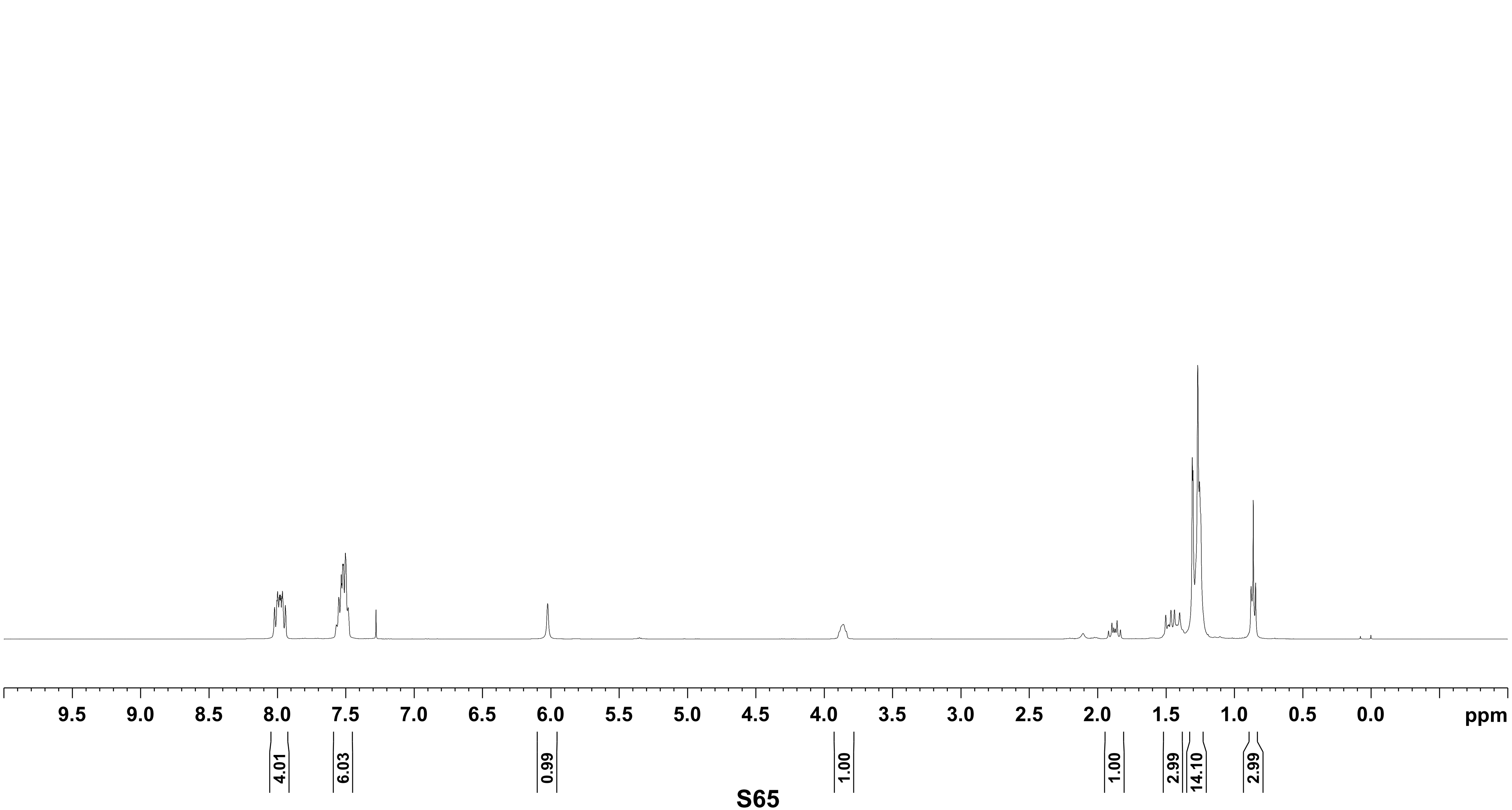

^{13}C NMR (100.6 MHz, $CDCl_3$)

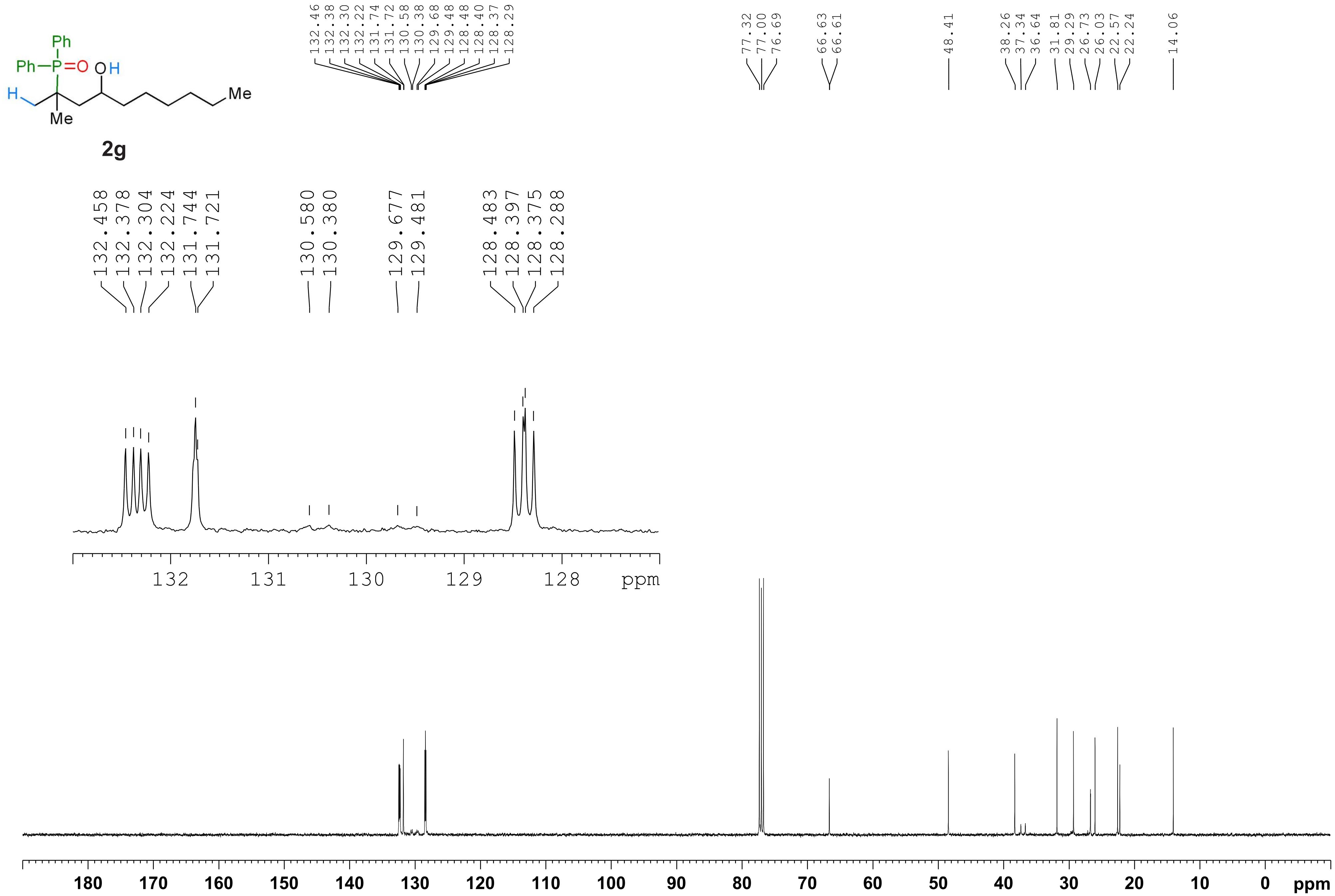

^{31}P NMR (162 MHz, $CDCl_3$)

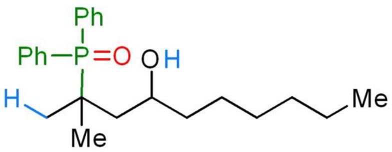

2g

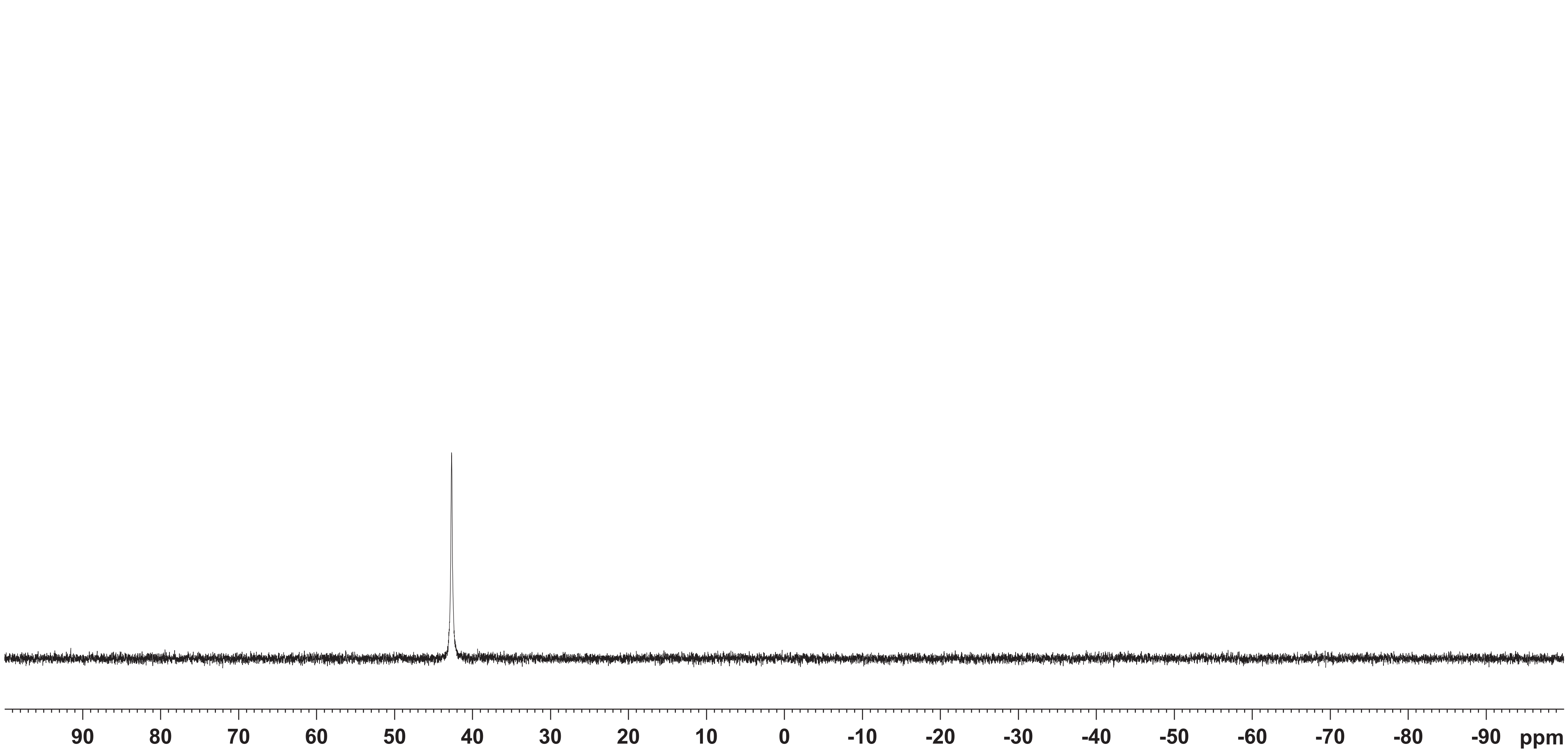

¹H NMR (400 MHz, $CDCl_3$)

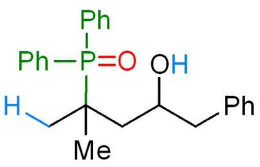

2h

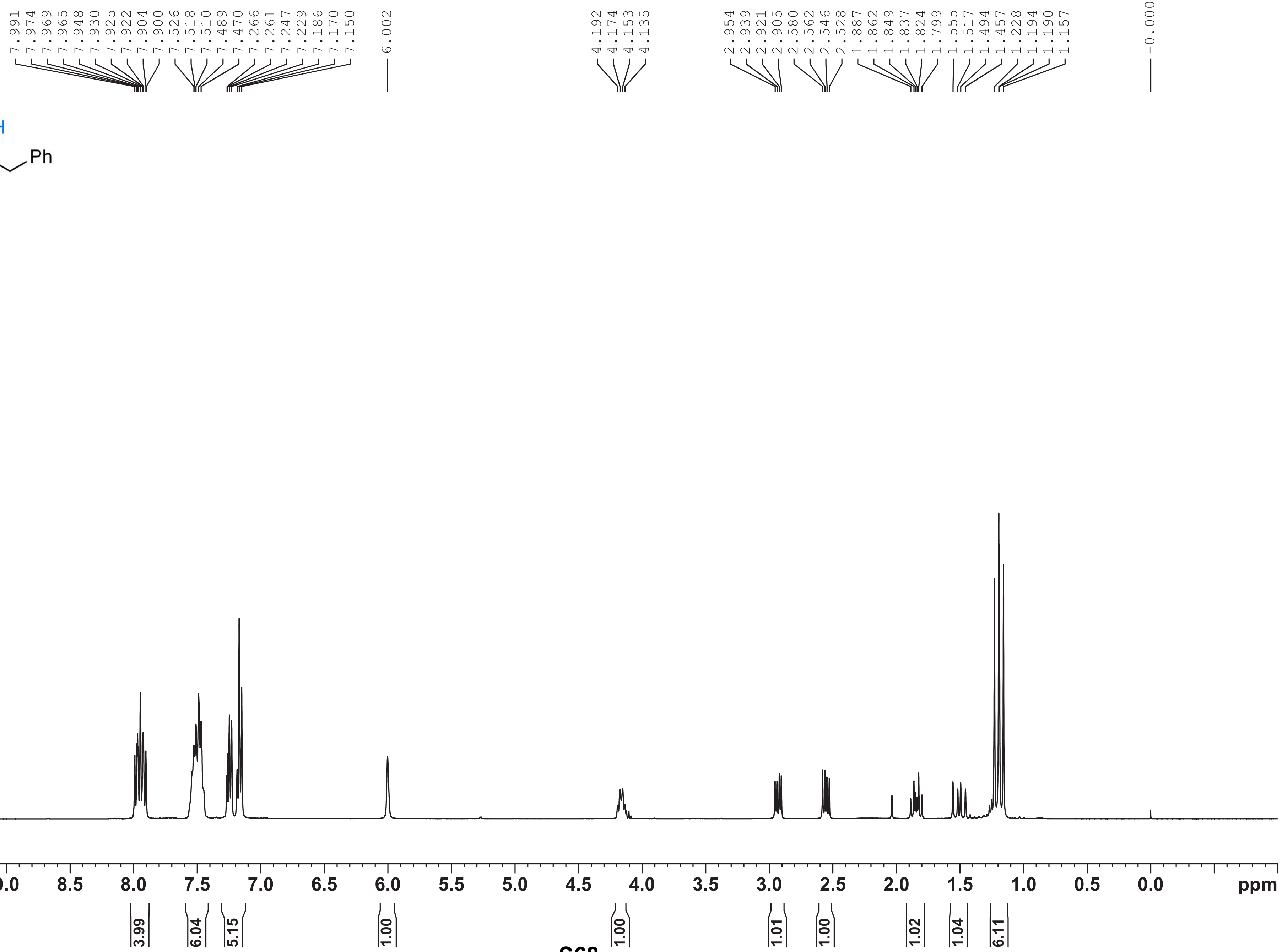

^{13}C NMR (100.6 MHz, CDCl$_3$)

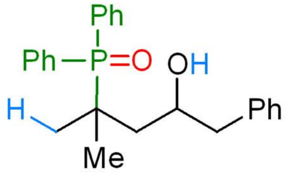

2h

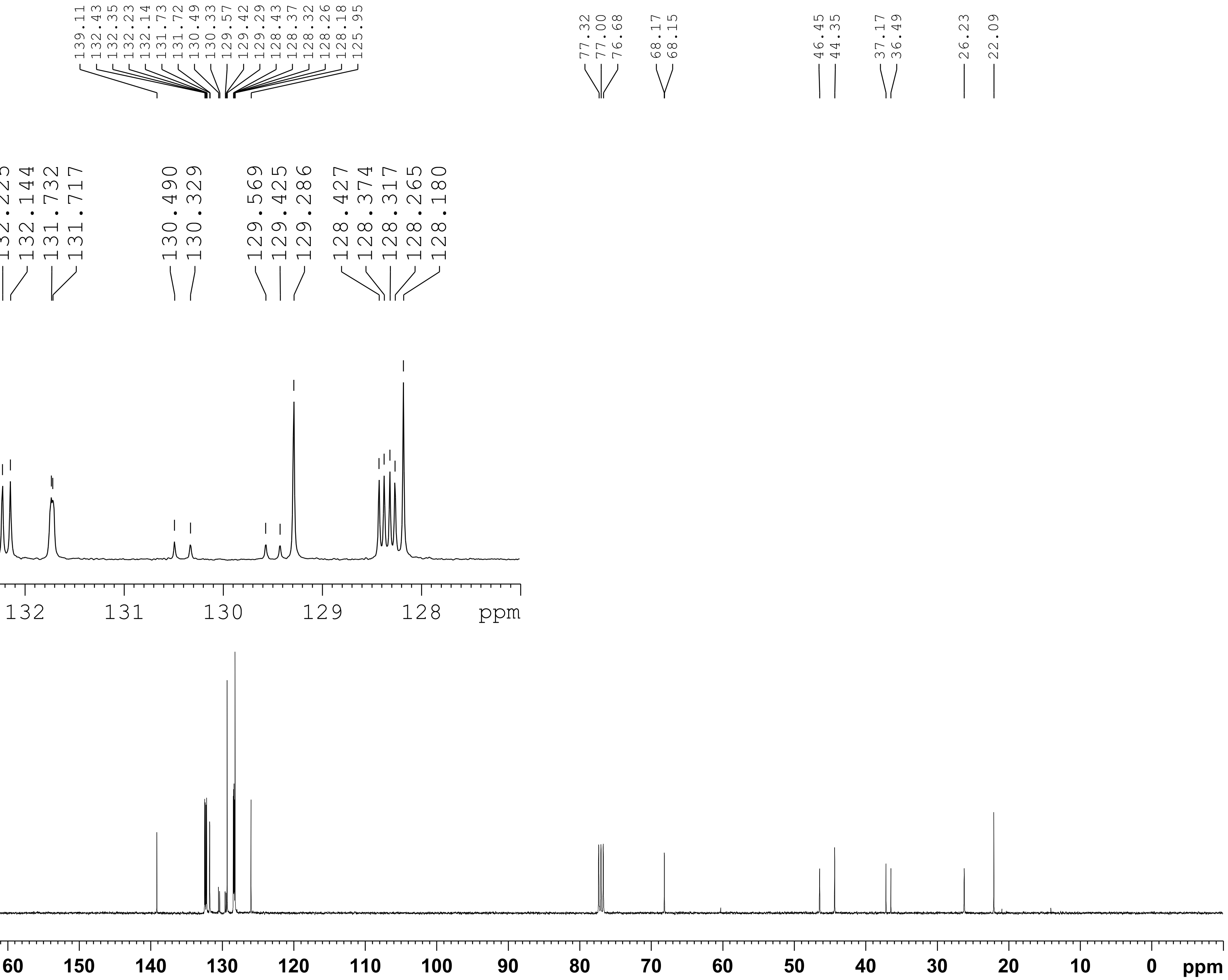

^{31}P NMR (162 MHz, $CDCl_3$)

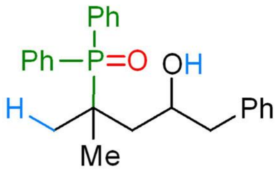

2h

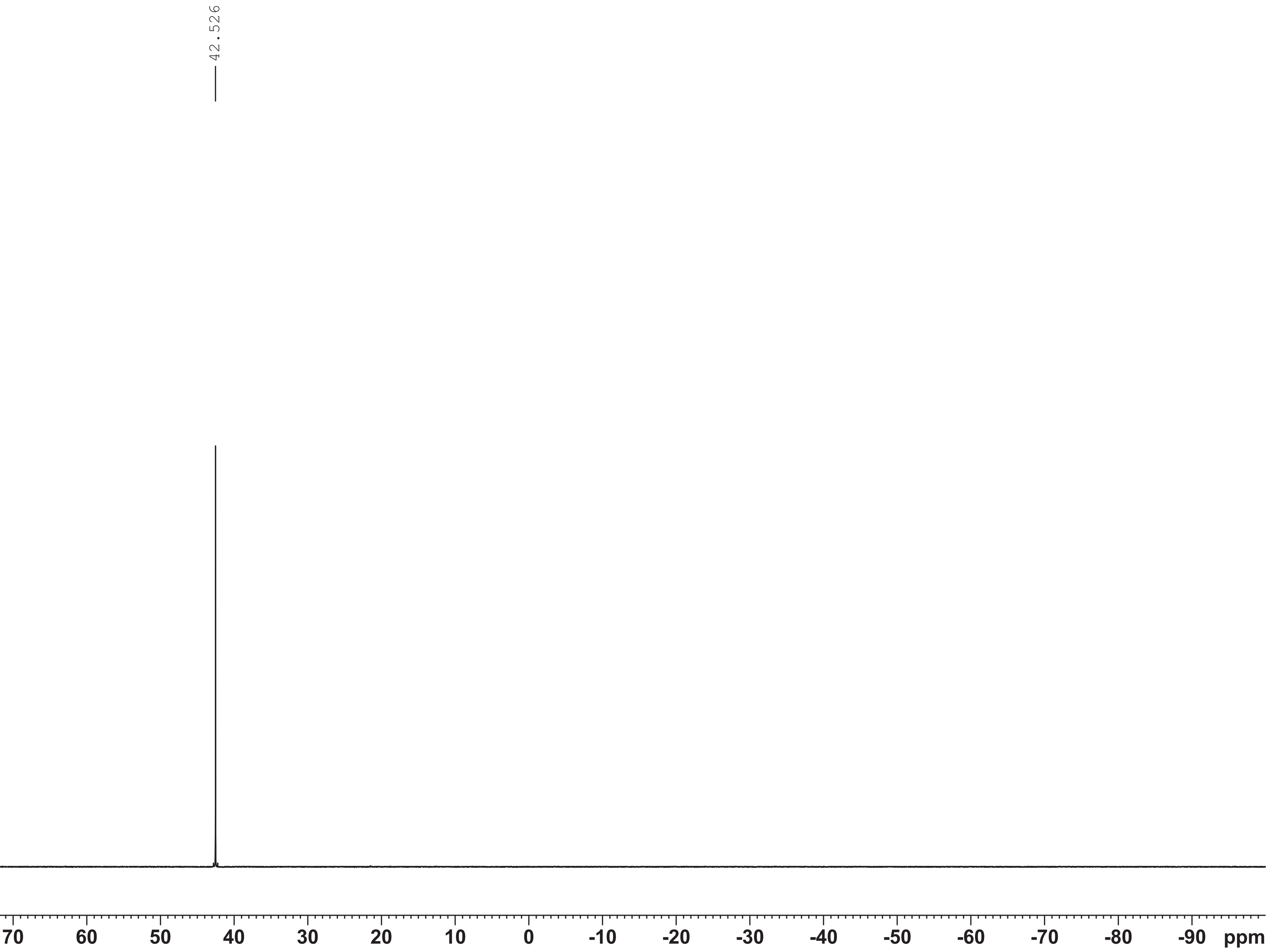

^{1}H NMR (400 MHz, $CDCl_3$)

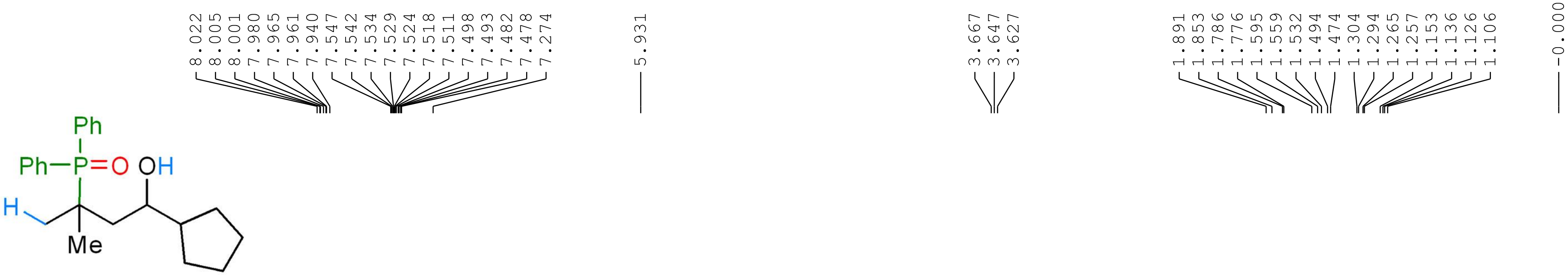

2i

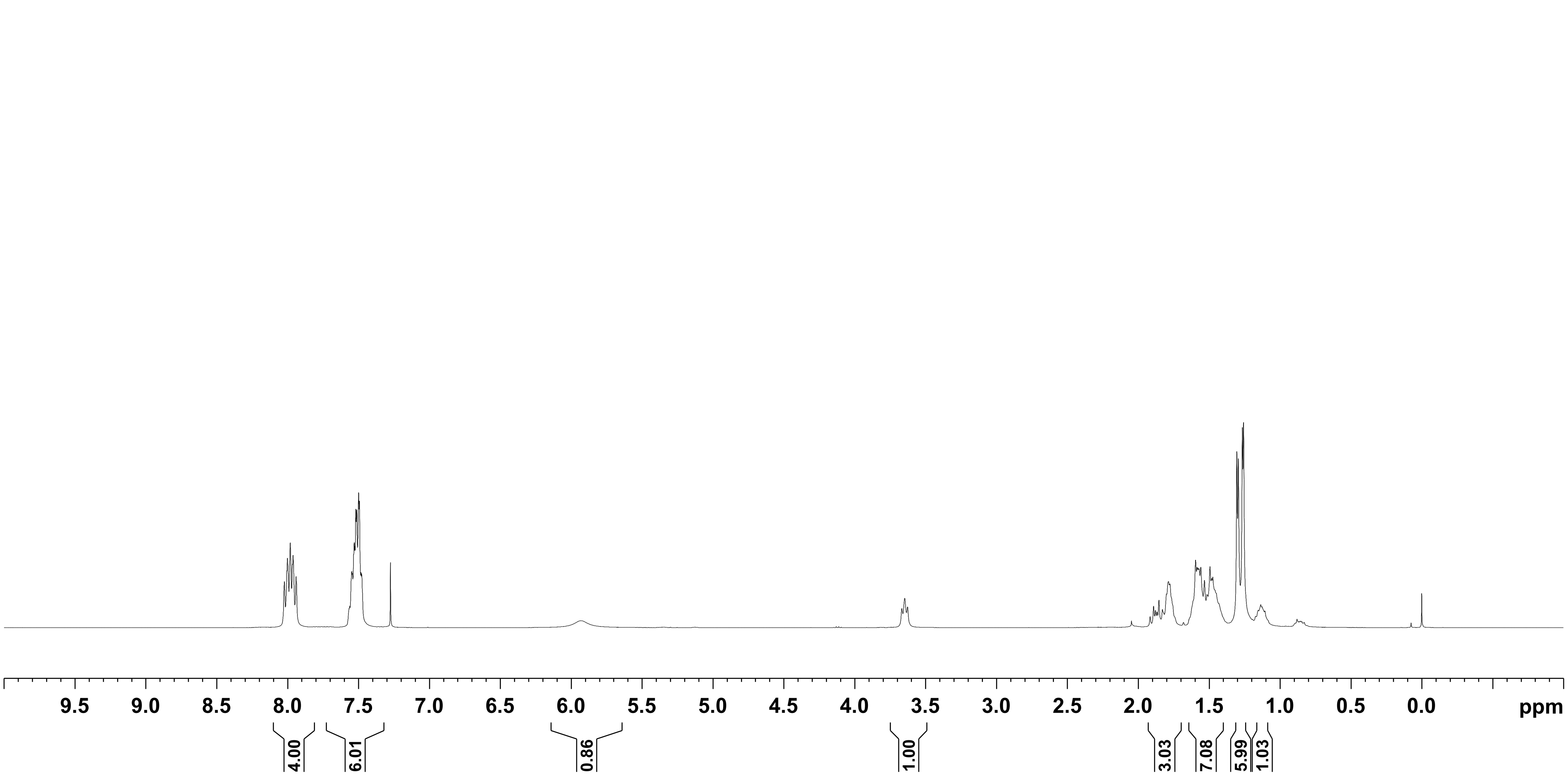

^{13}C NMR (100.6 MHz, $CDCl_3$)

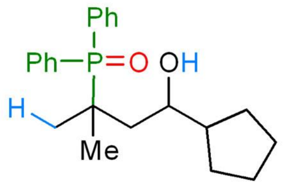

2i

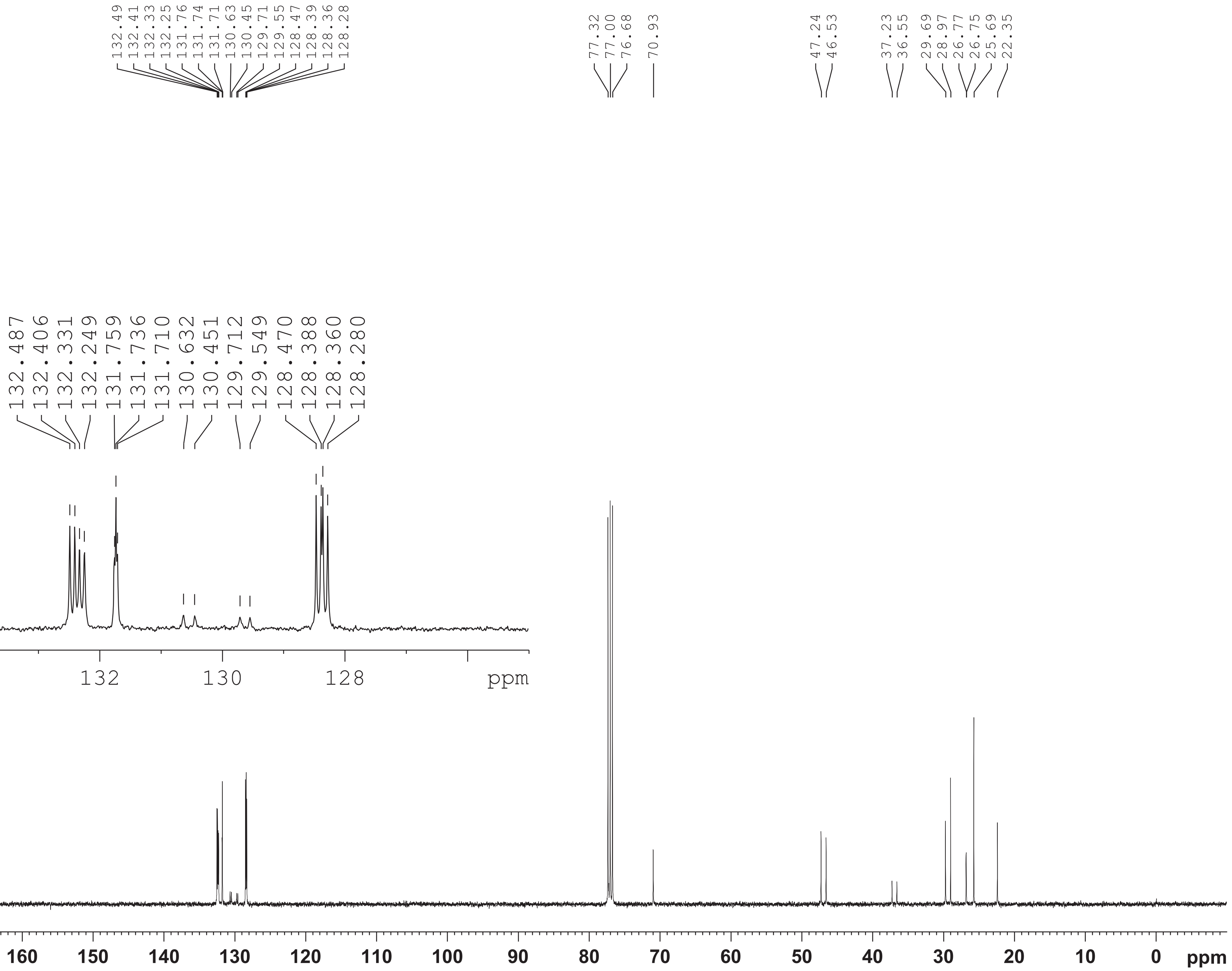

^{31}P NMR (162 MHz, $CDCl_3$)

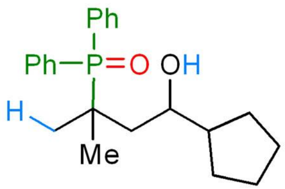

2i

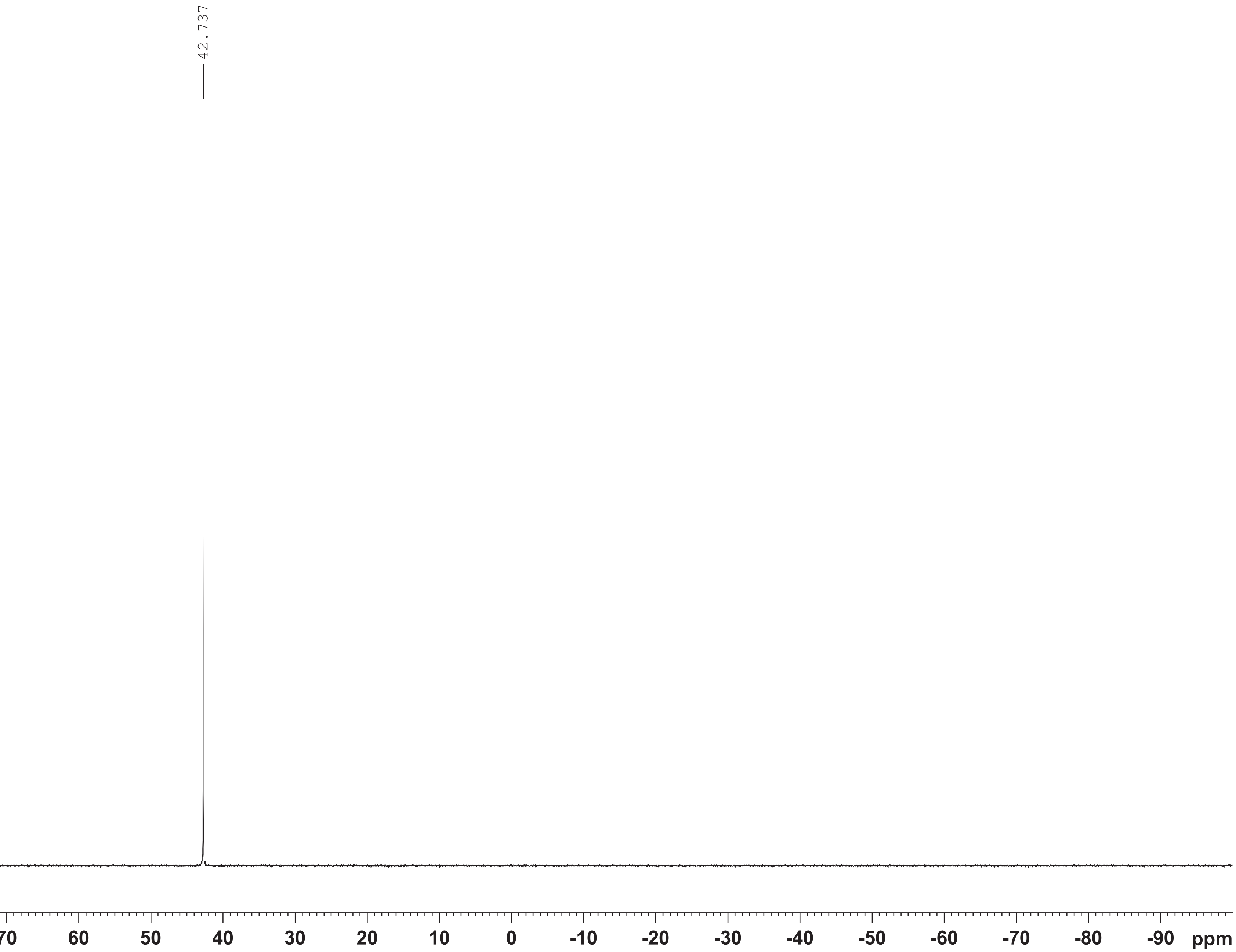

^{1}H NMR (400 MHz, $CDCl_3$)

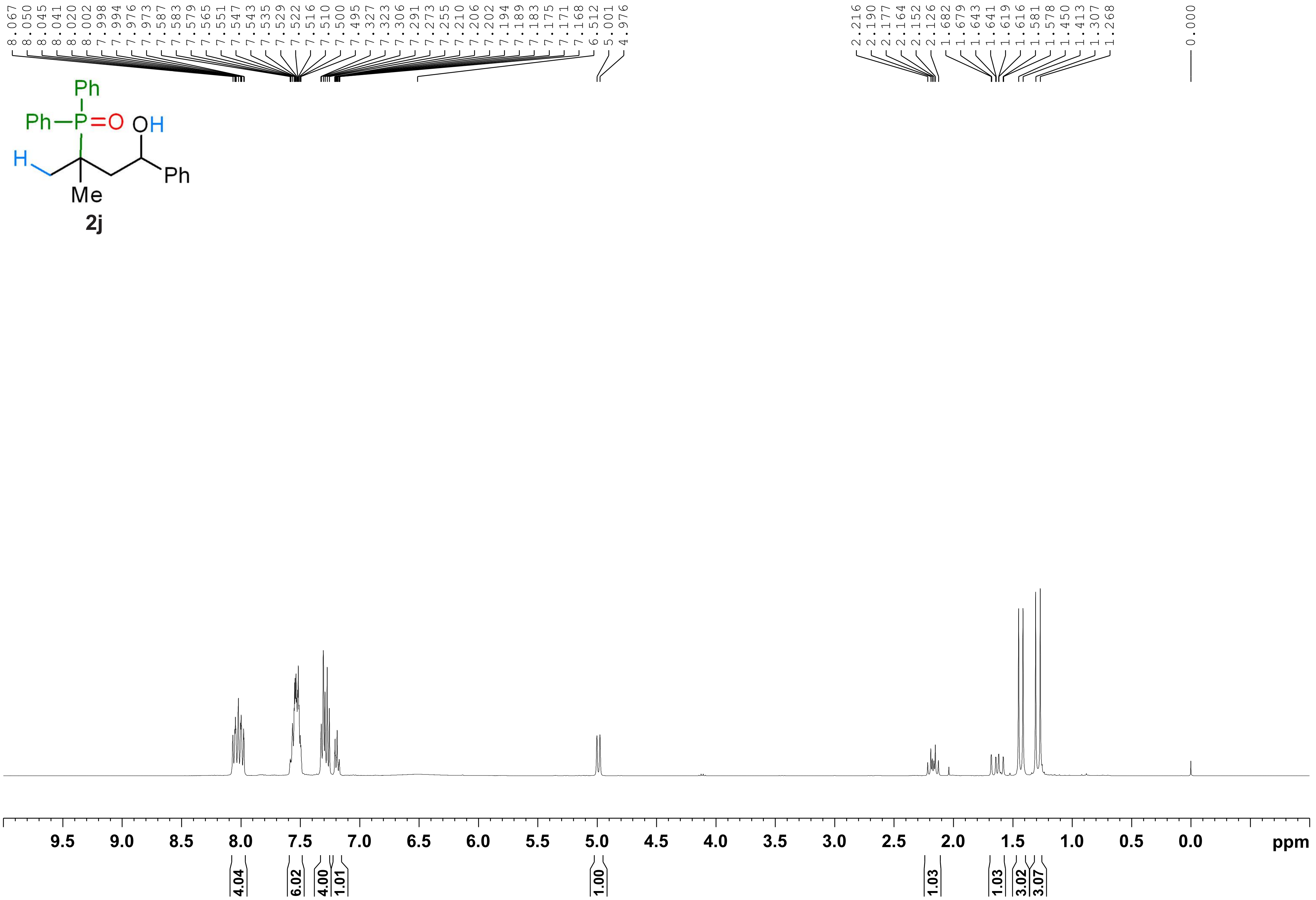

^{13}C NMR (100.6 MHz, CDCl$_3$)

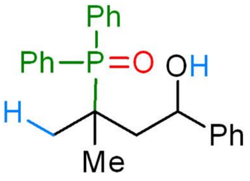

2j

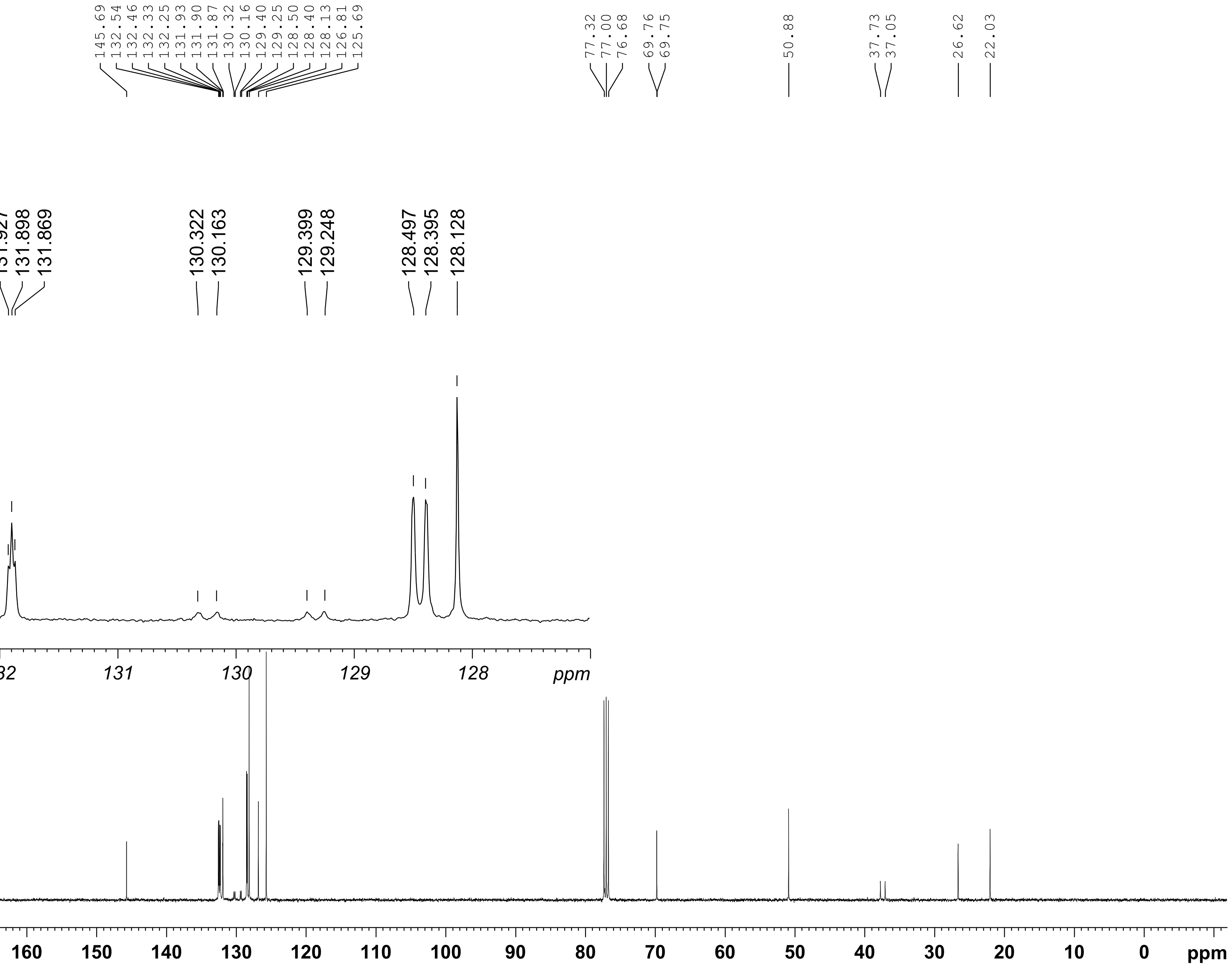

^{31}P NMR (162 MHz, $CDCl_3$)

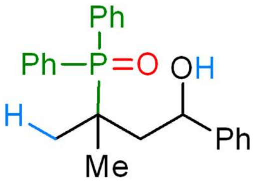

2j

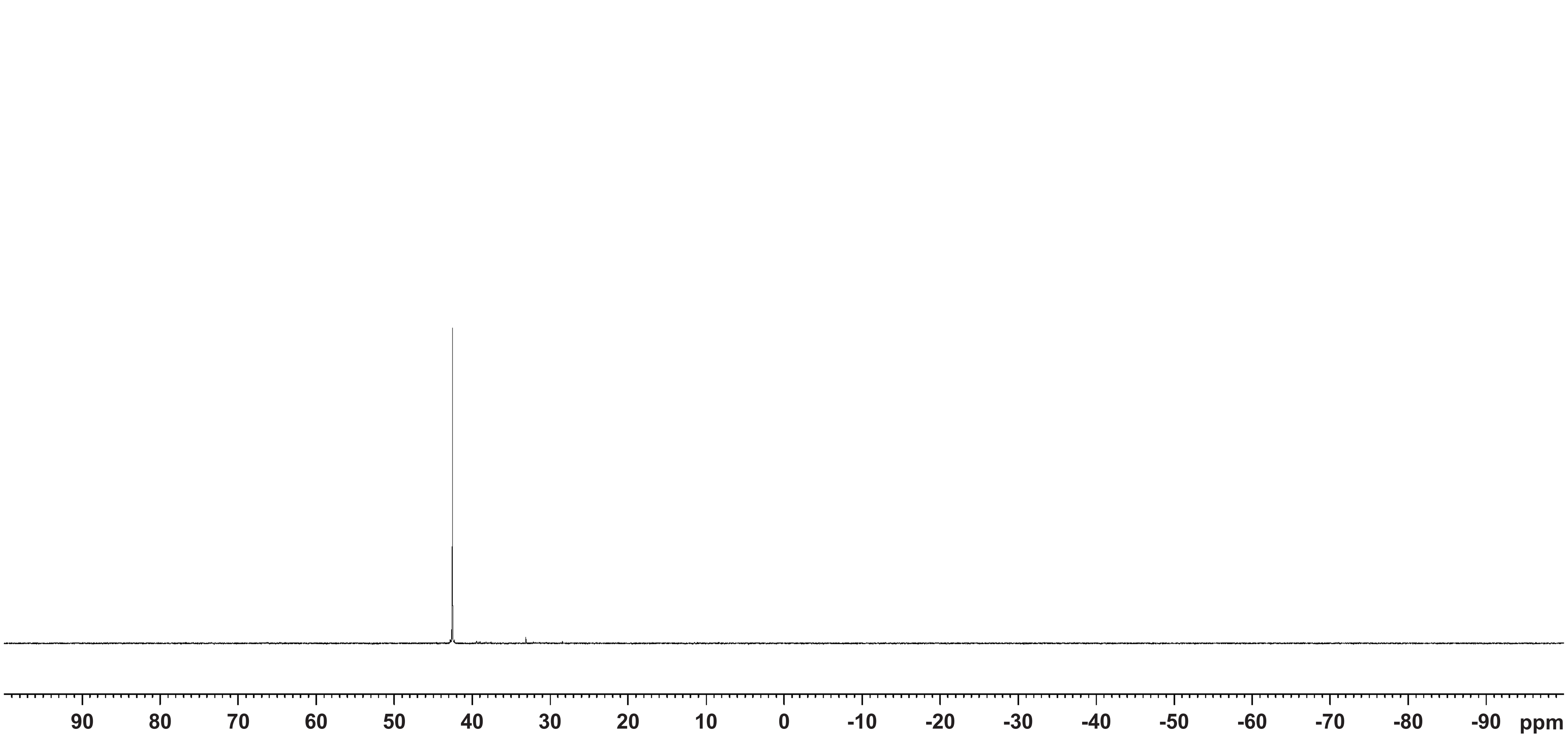

^{1}H NMR (400 MHz, $CDCl_3$)

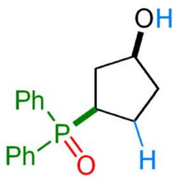

2k

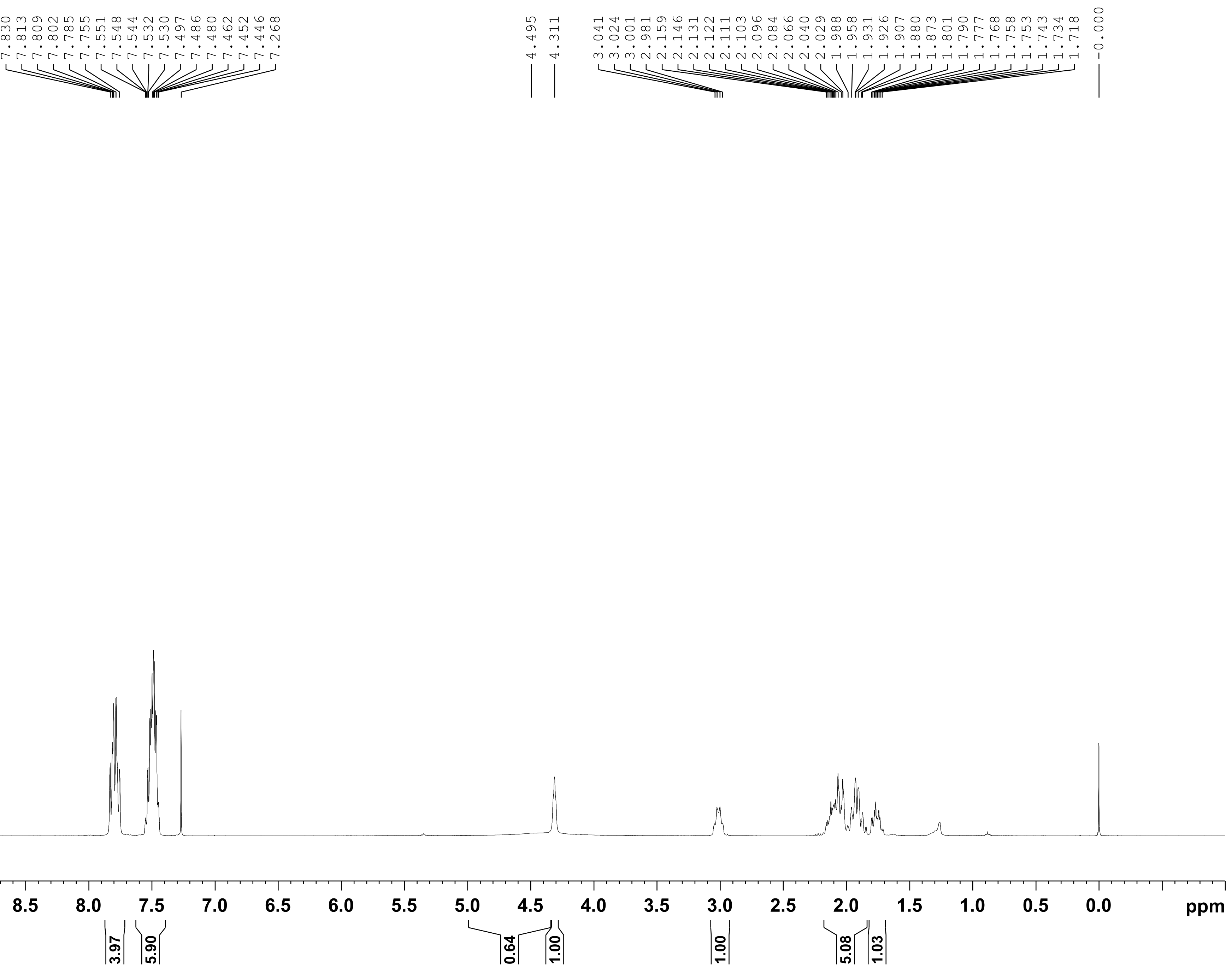

^{13}C NMR (100.6 MHz, $CDCl_3$)

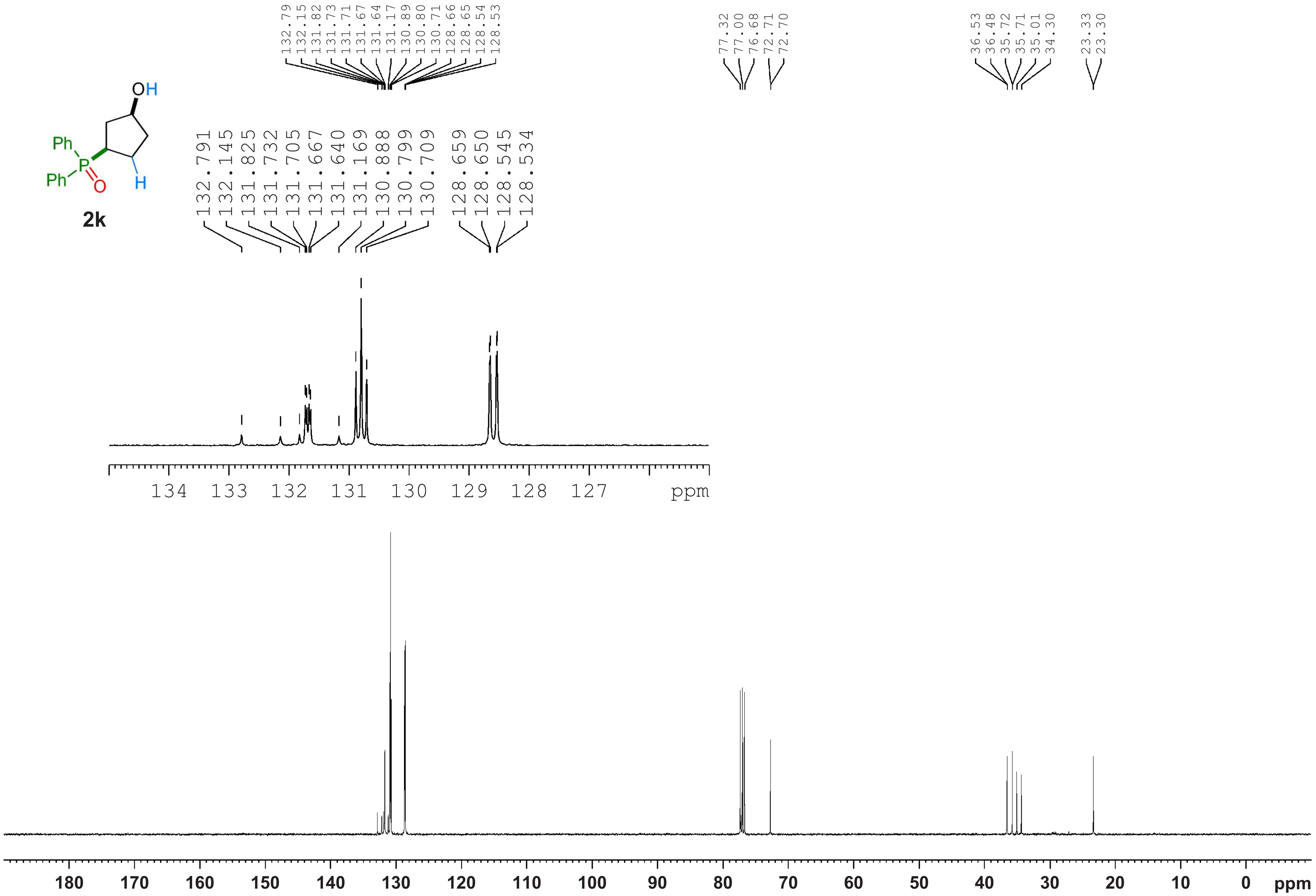

^{31}P NMR (162 MHz, $CDCl_3$)

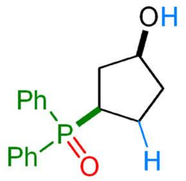

2k

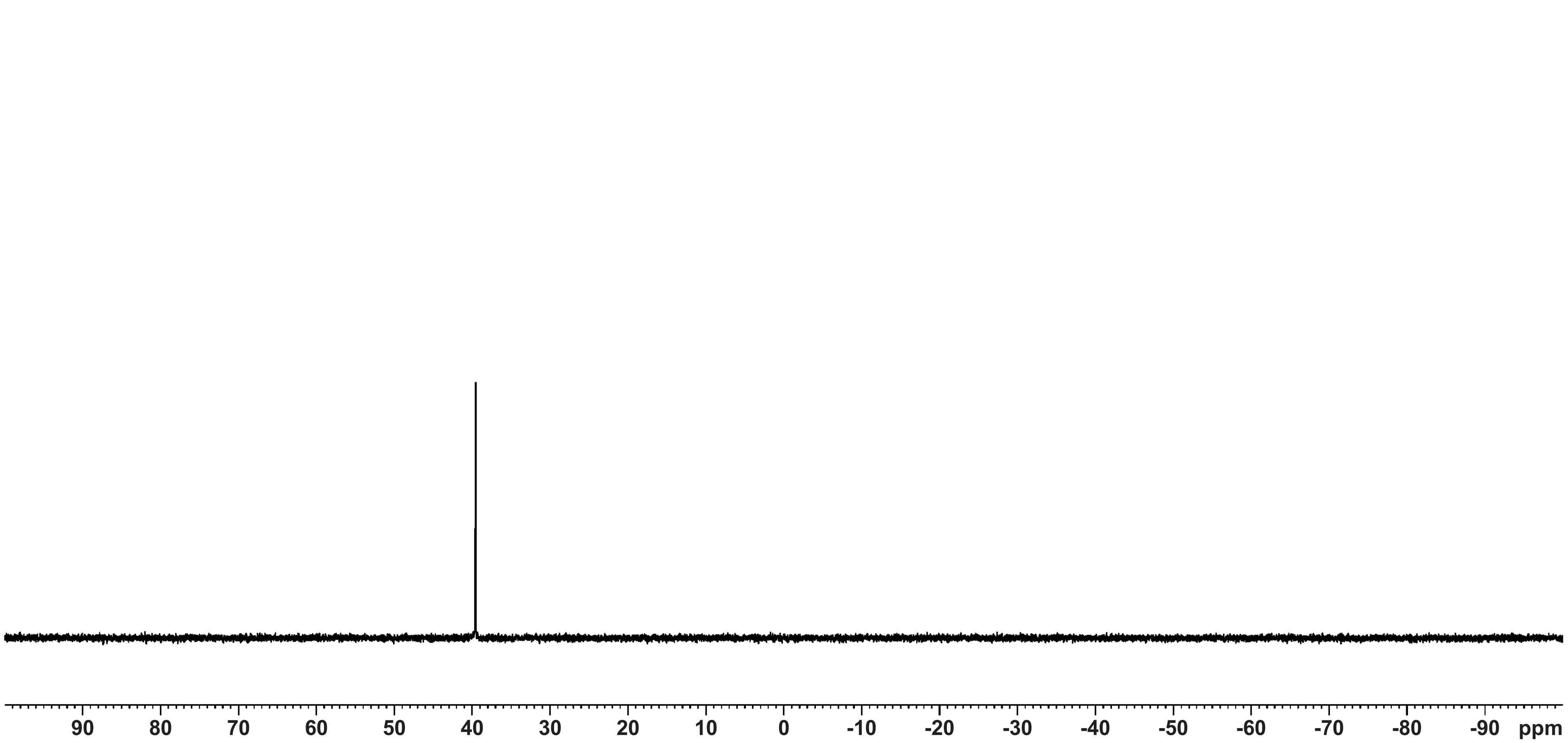

^1H NMR (400 MHz, $CDCl_3$)

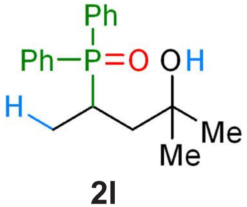

2l

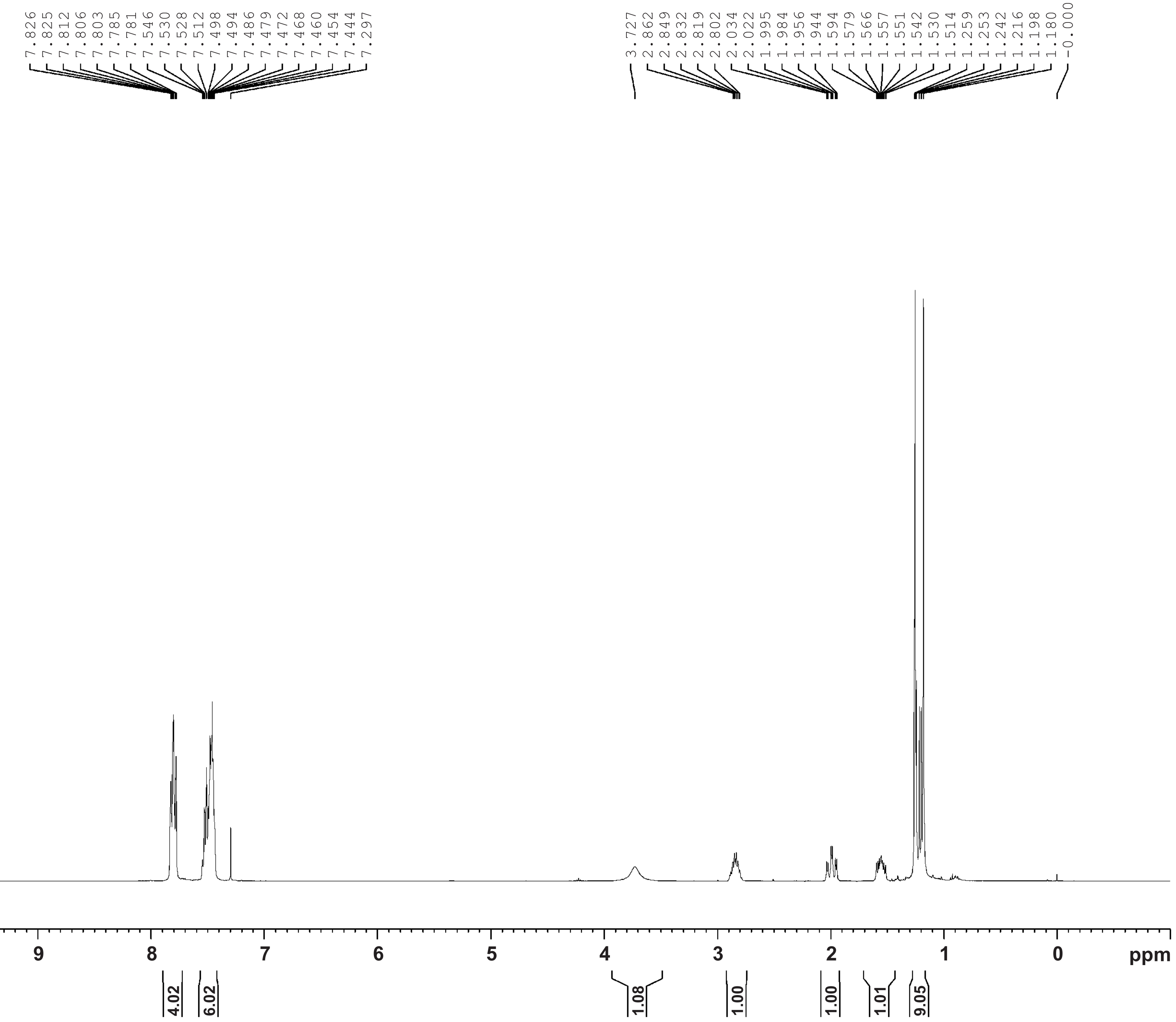

^{13}C NMR (100.6 MHz, CDCl$_3$)

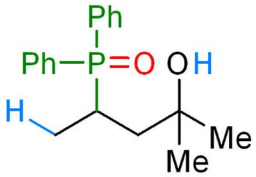

2l

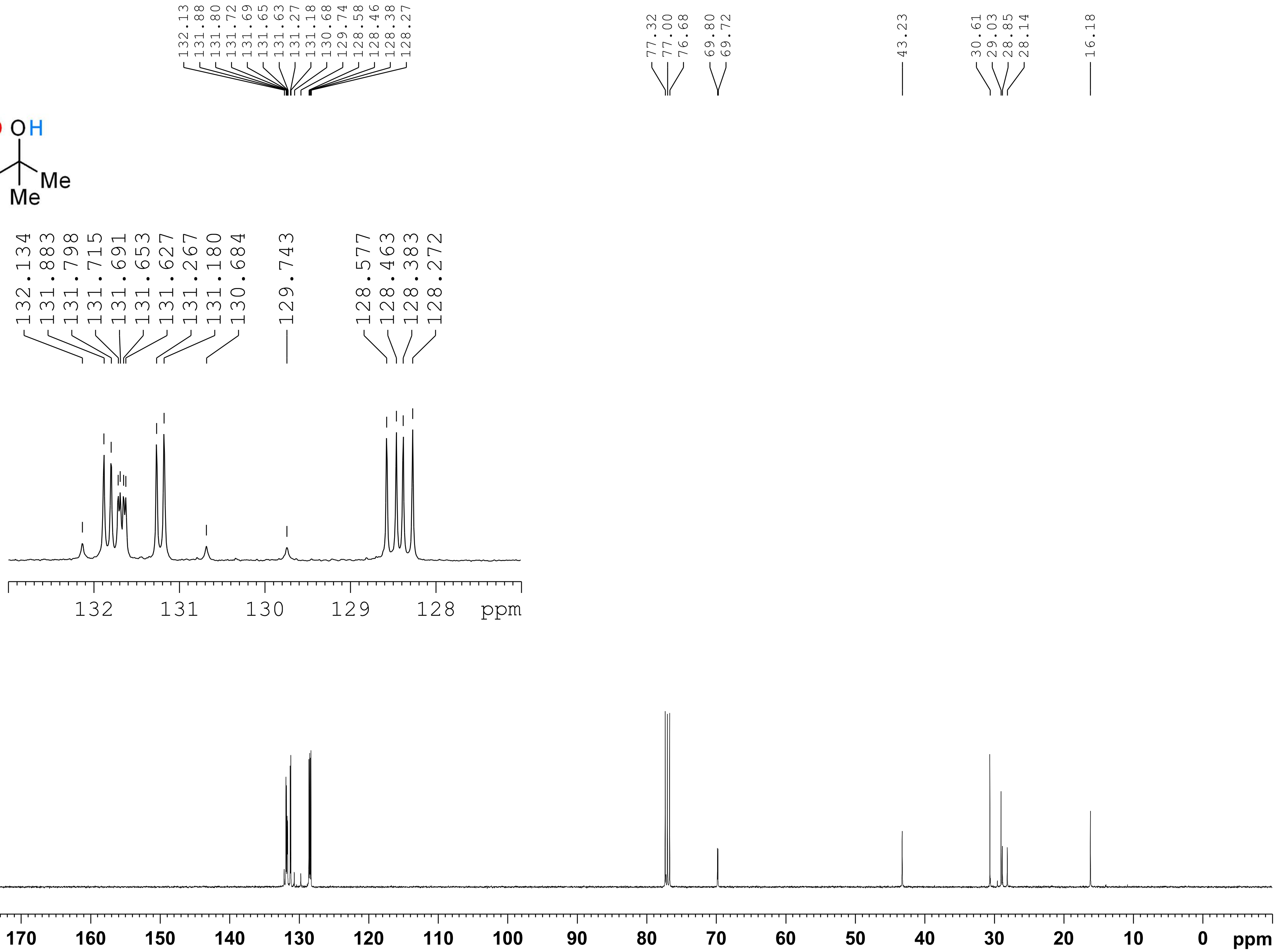

^{31}P NMR (162 MHz, $CDCl_3$)

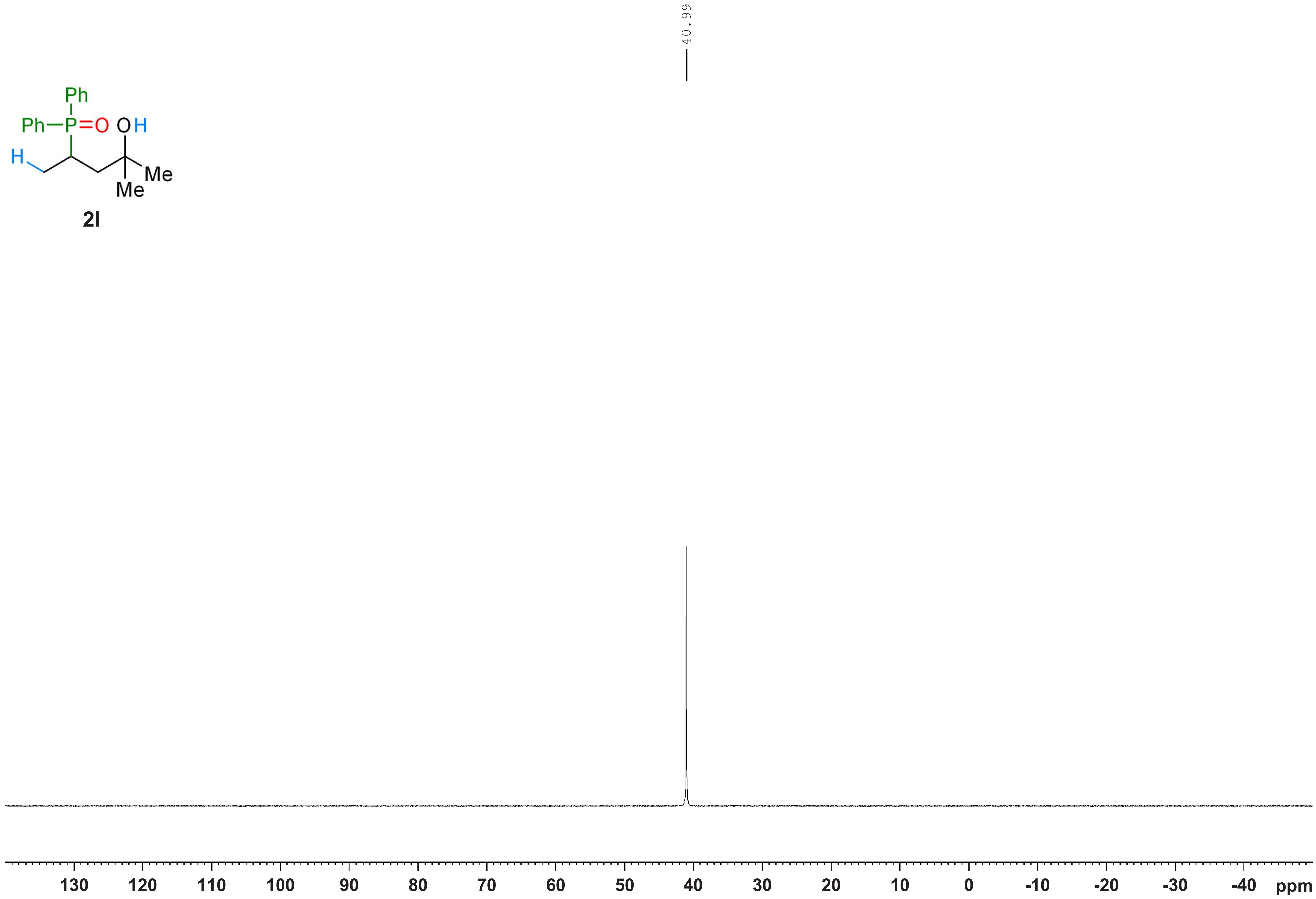

^{1}H NMR (400 MHz, $CDCl_3$)

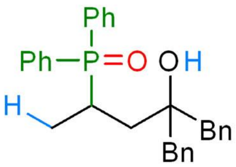

2m

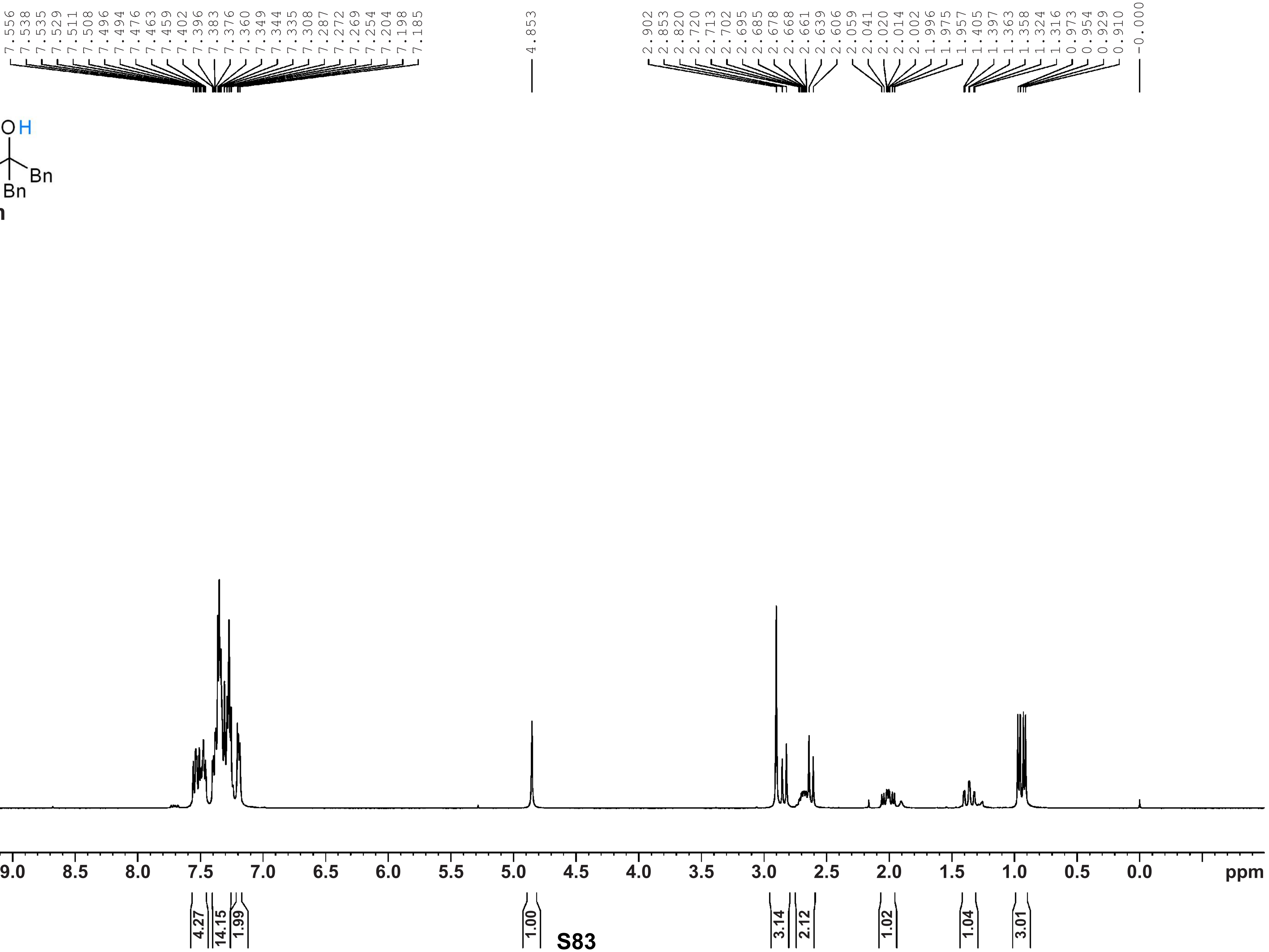

^{13}C NMR (100.6 MHz, $CDCl_3$)

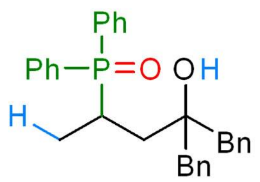

2m

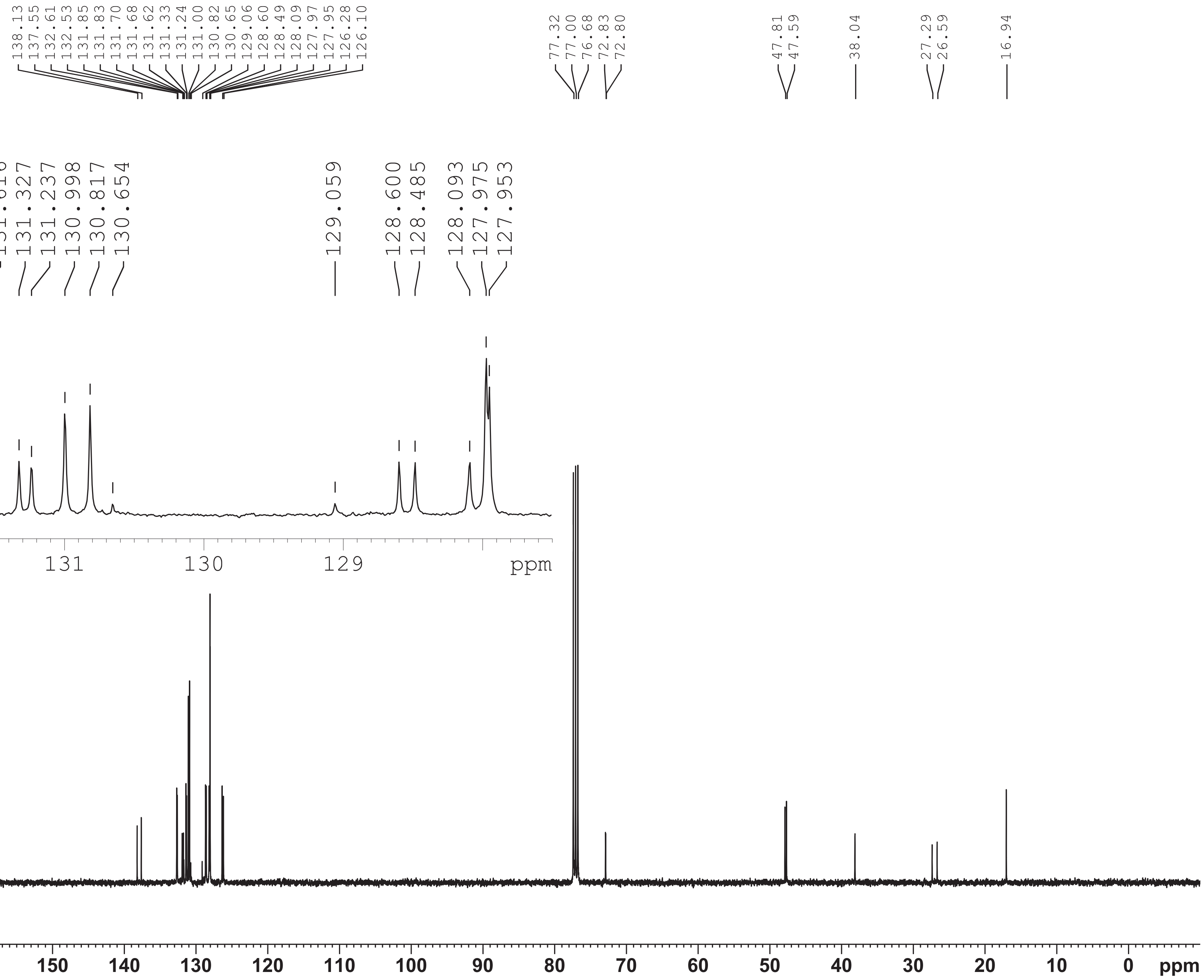

^{31}P NMR (162 MHz, CDCl$_3$)

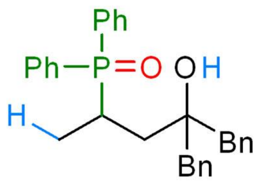

2m

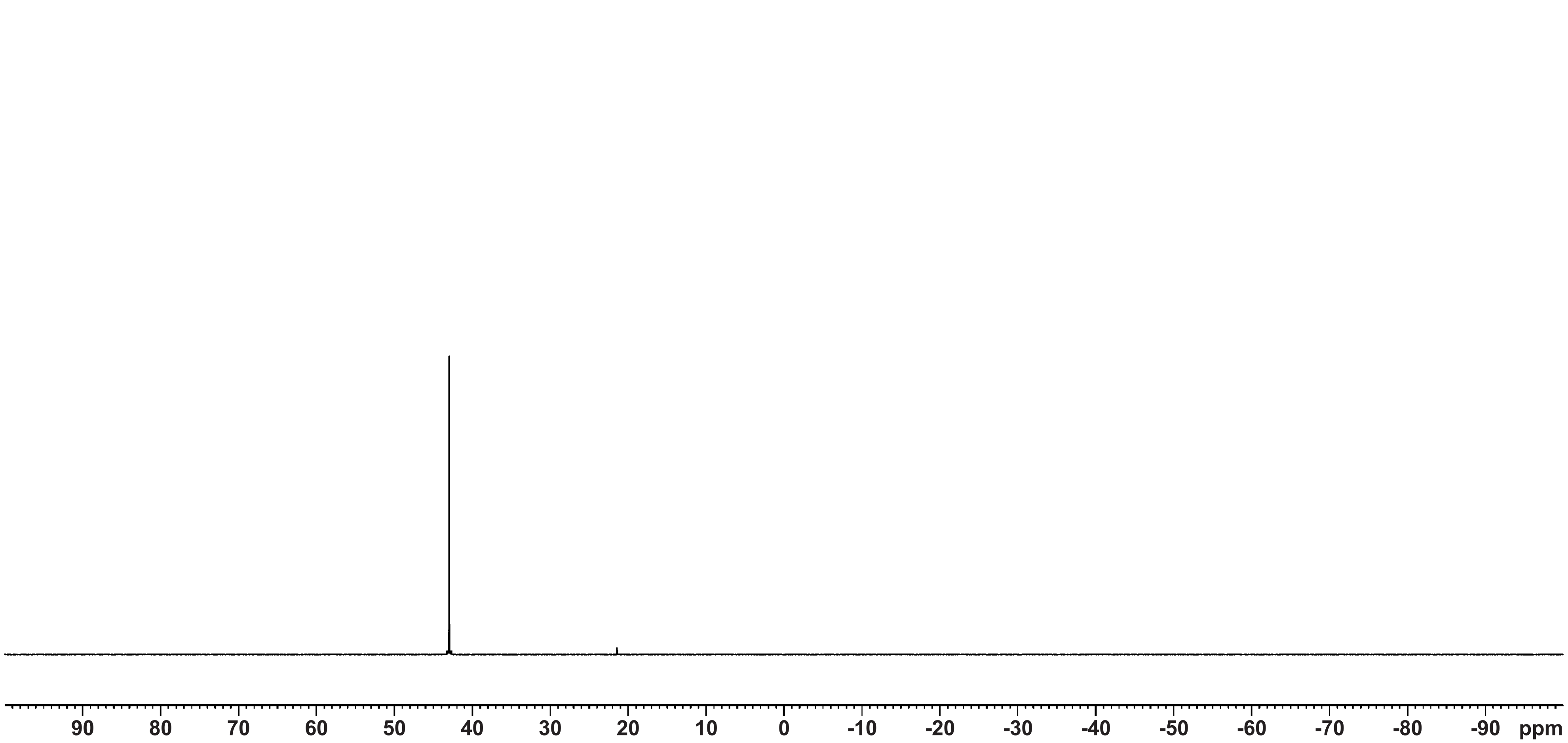

^{1}H NMR (400 MHz, $CDCl_3$)

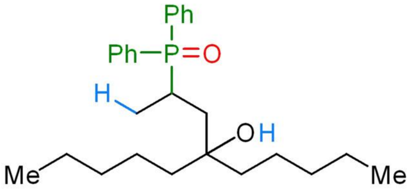

2n

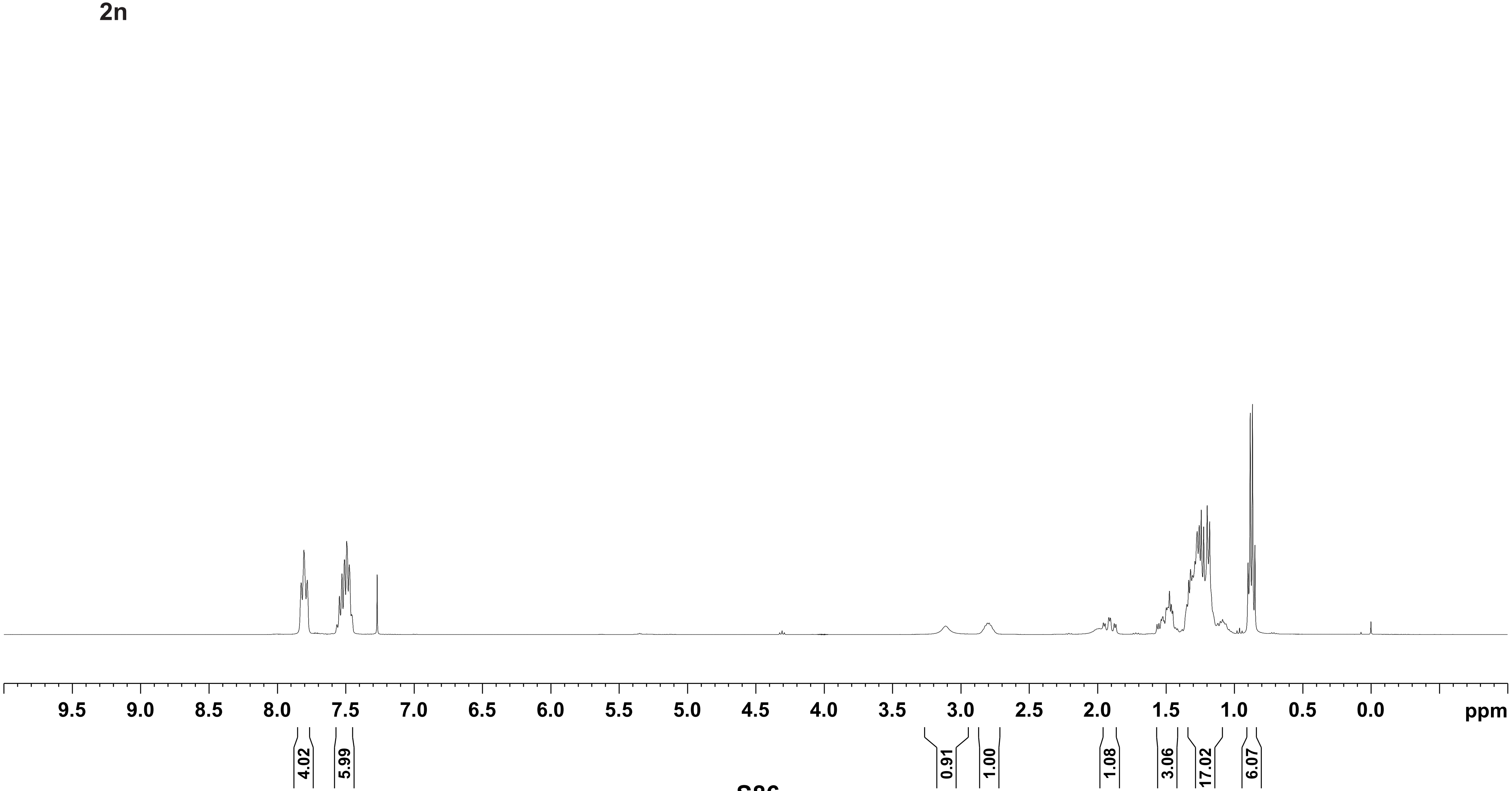

^{13}C NMR (100.6 MHz, $CDCl_3$)

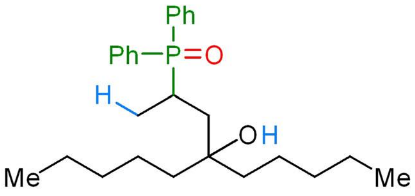

2n

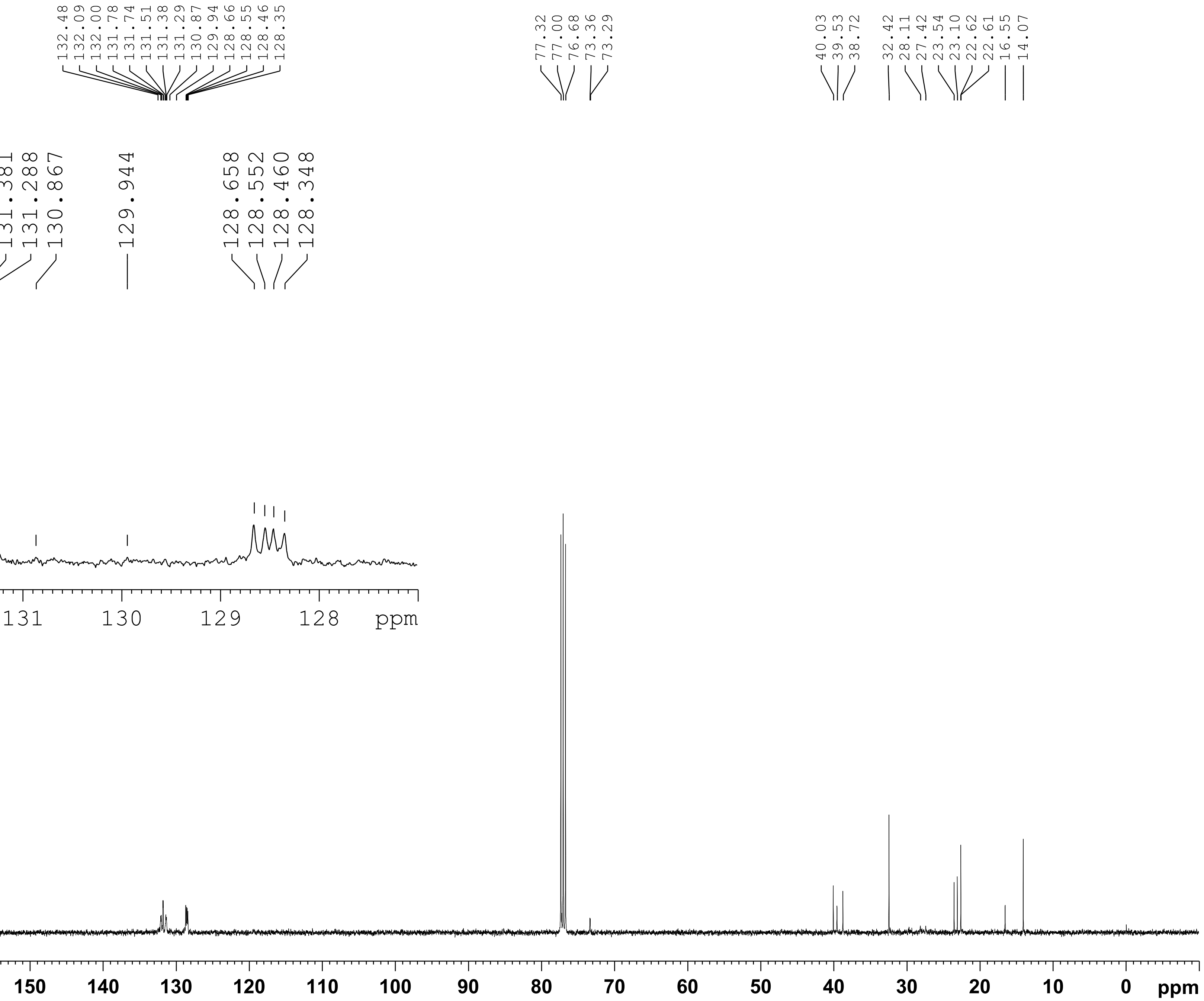

^{31}P NMR (162 MHz, $CDCl_3$)

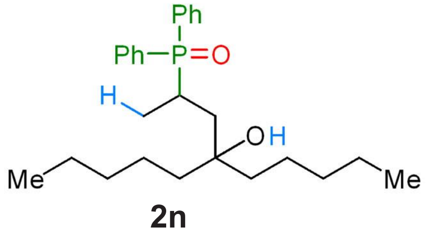

2n

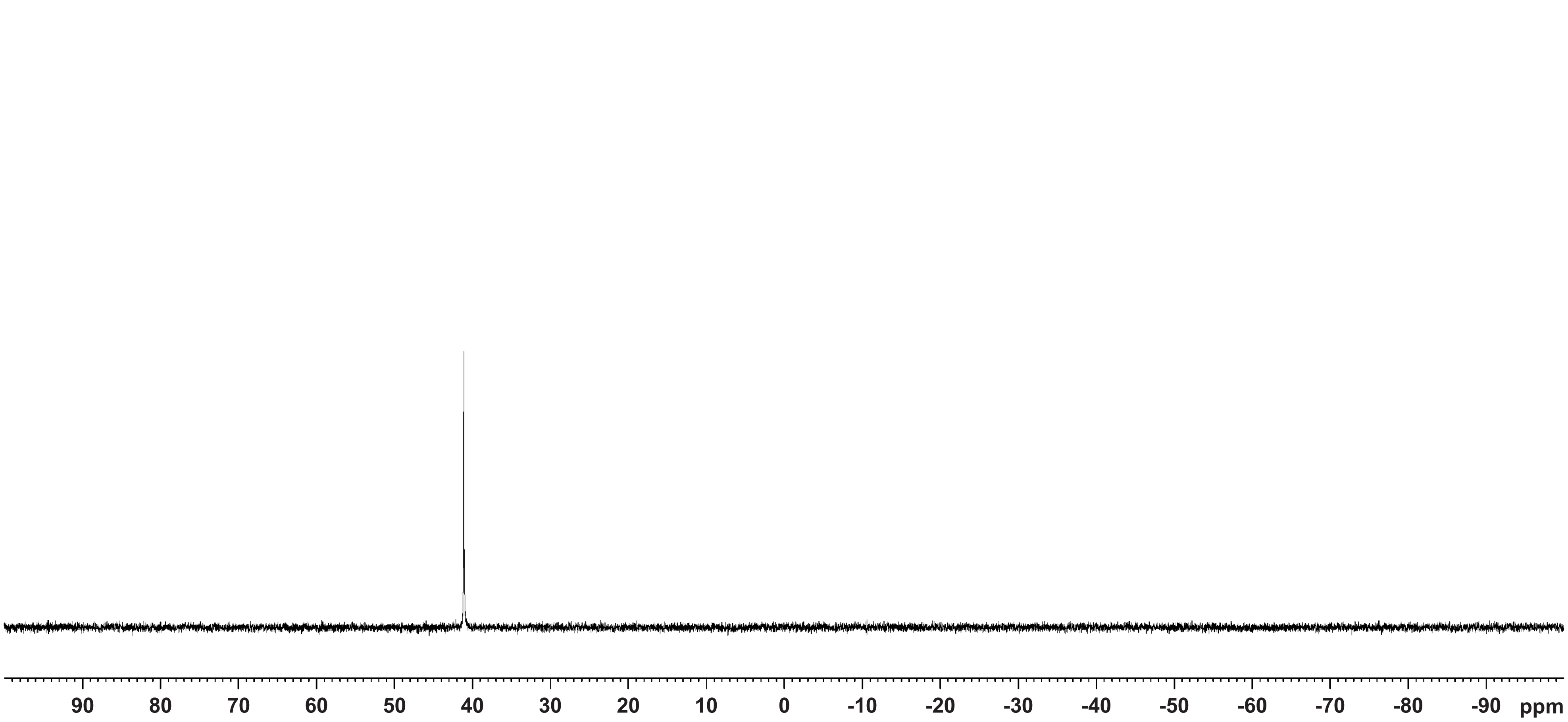

^{1}H NMR (400 MHz, $CDCl_3$)

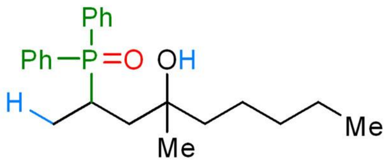

2o

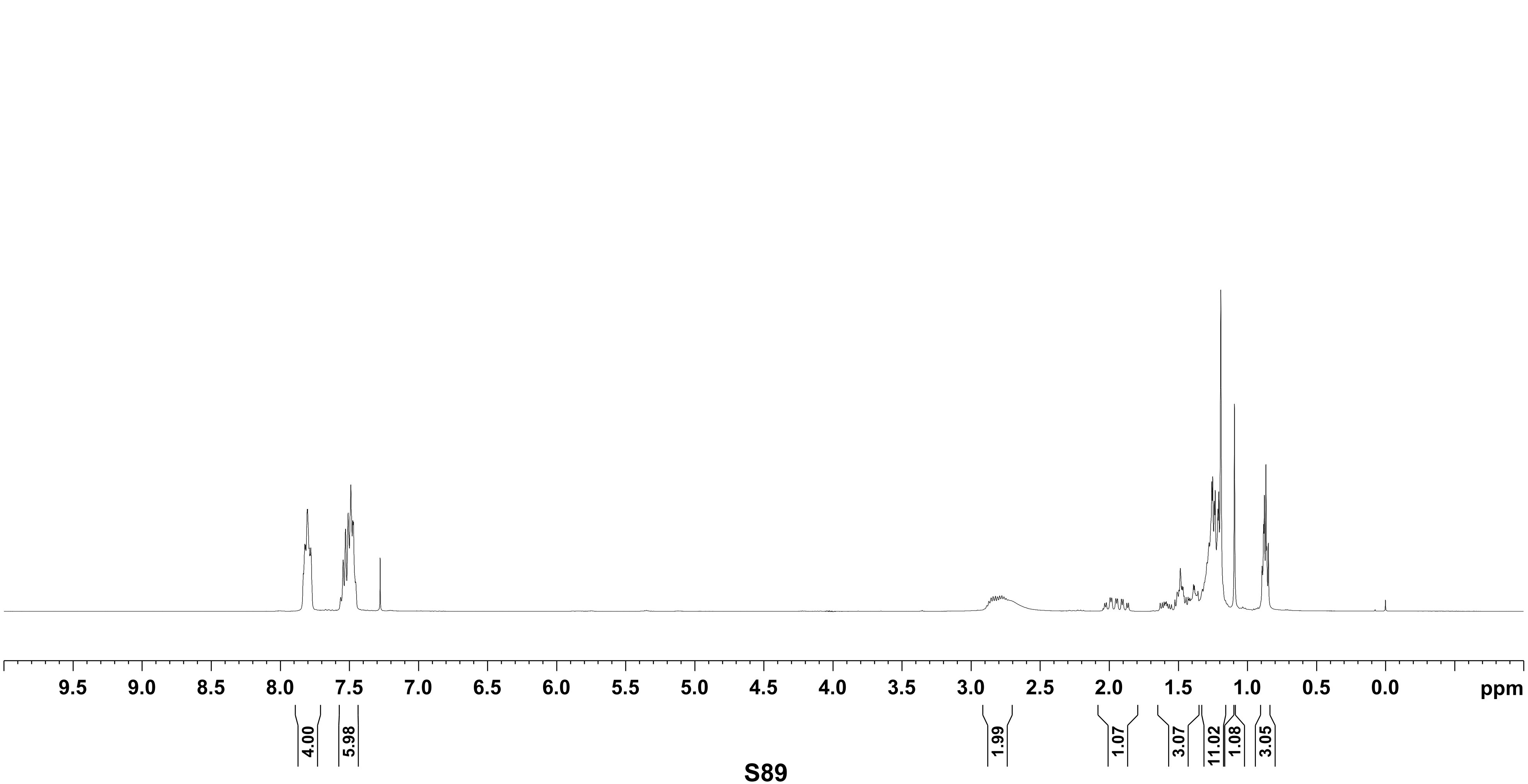

^{13}C NMR (100.6 MHz, $CDCl_3$)

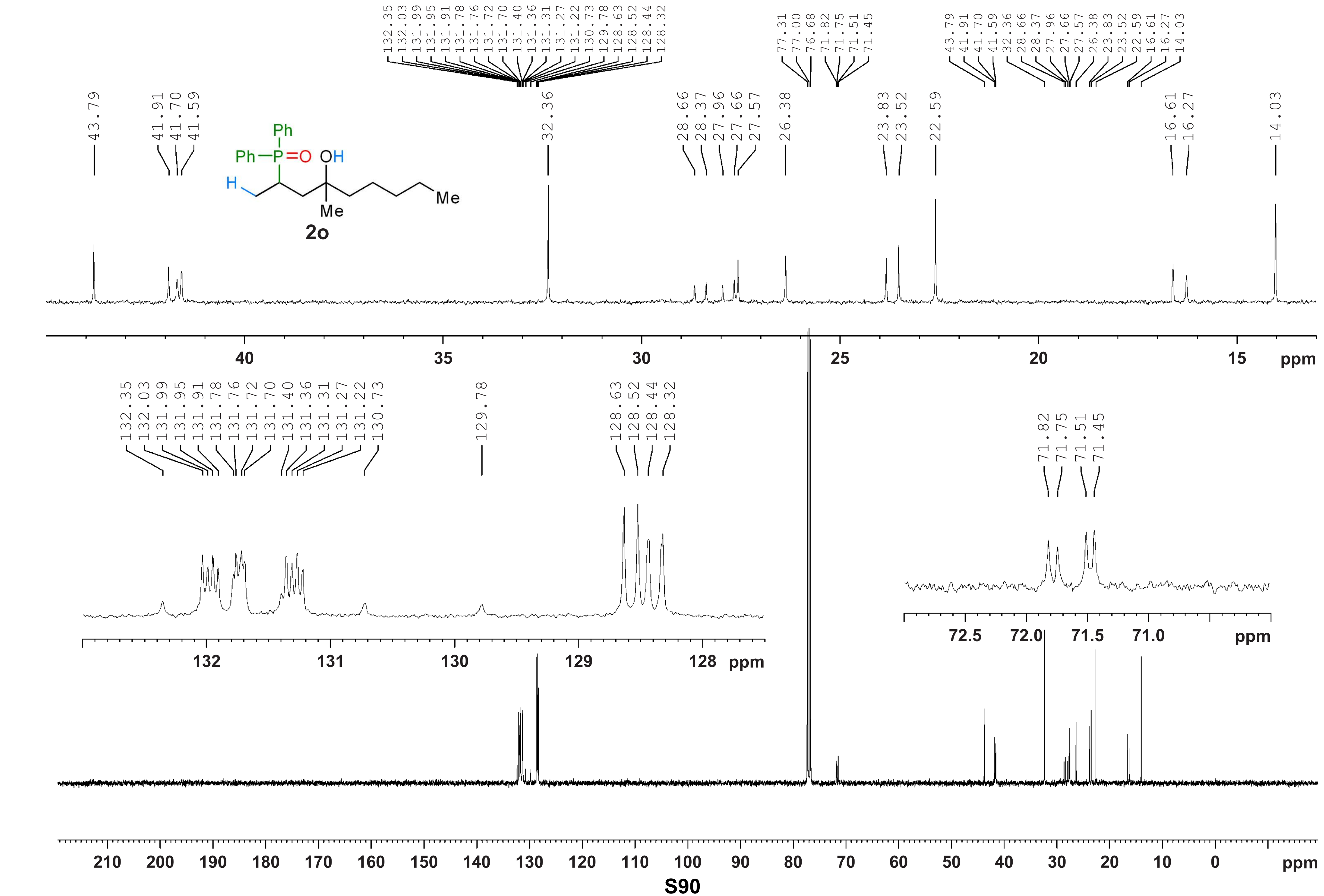

^{31}P NMR (162 MHz, CDCl$_3$)

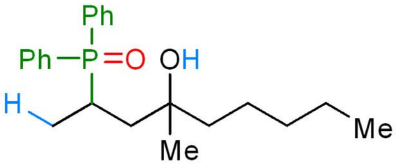

2o

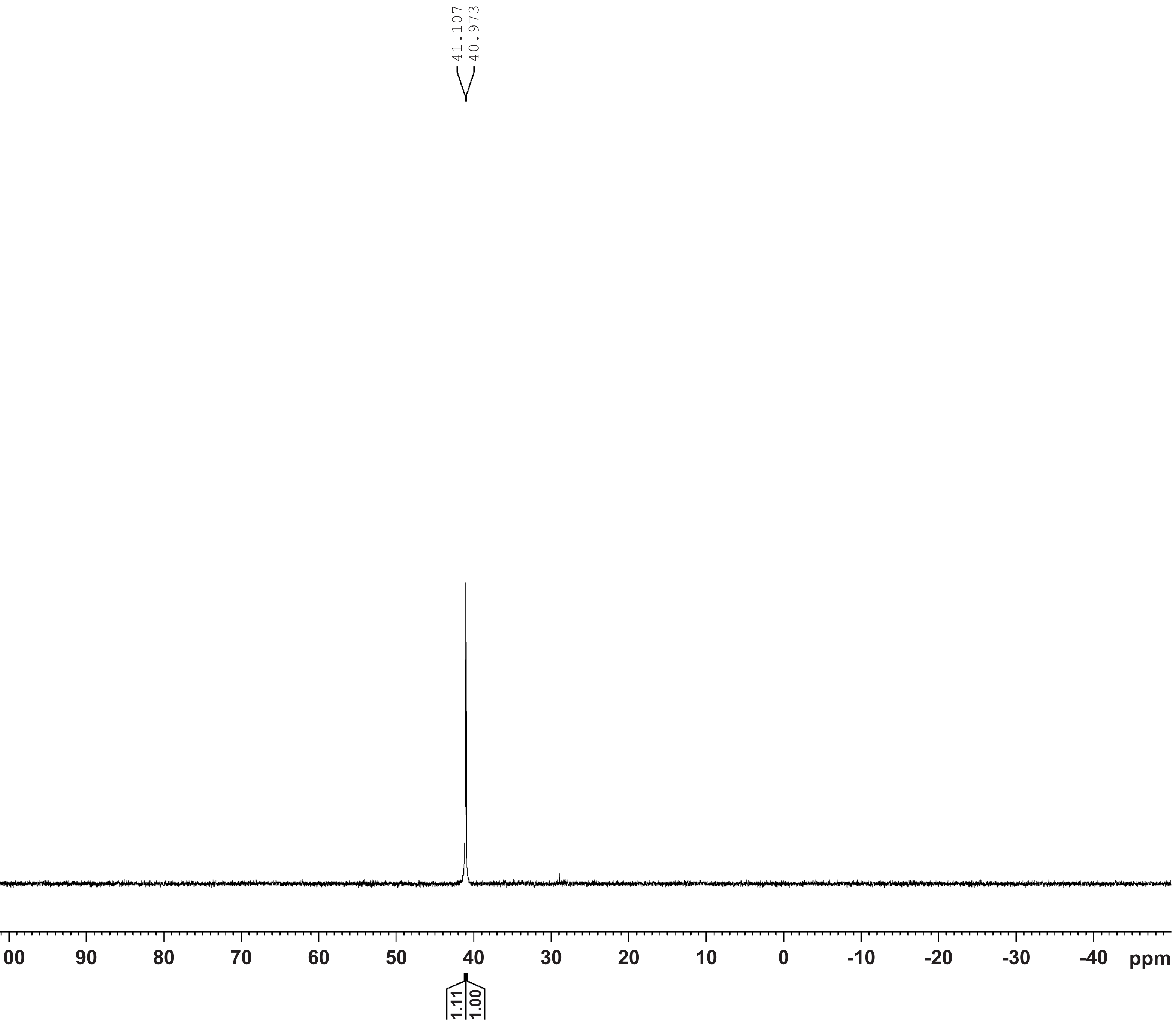

^{1}H NMR (400 MHz, $CDCl_3$)

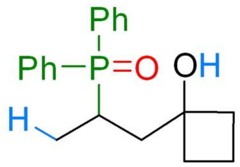

2p

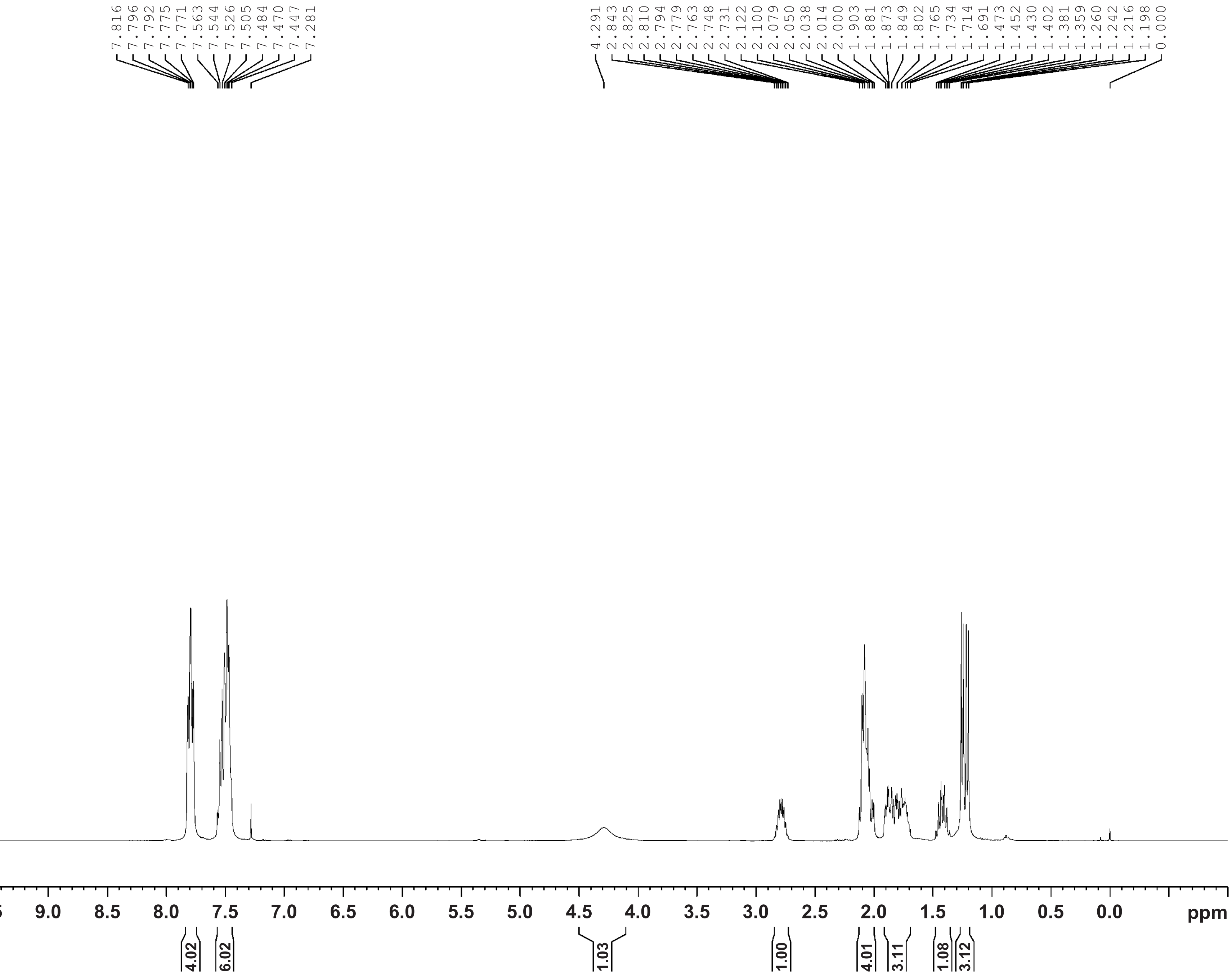

^{13}C NMR (100.6 MHz, $CDCl_3$)

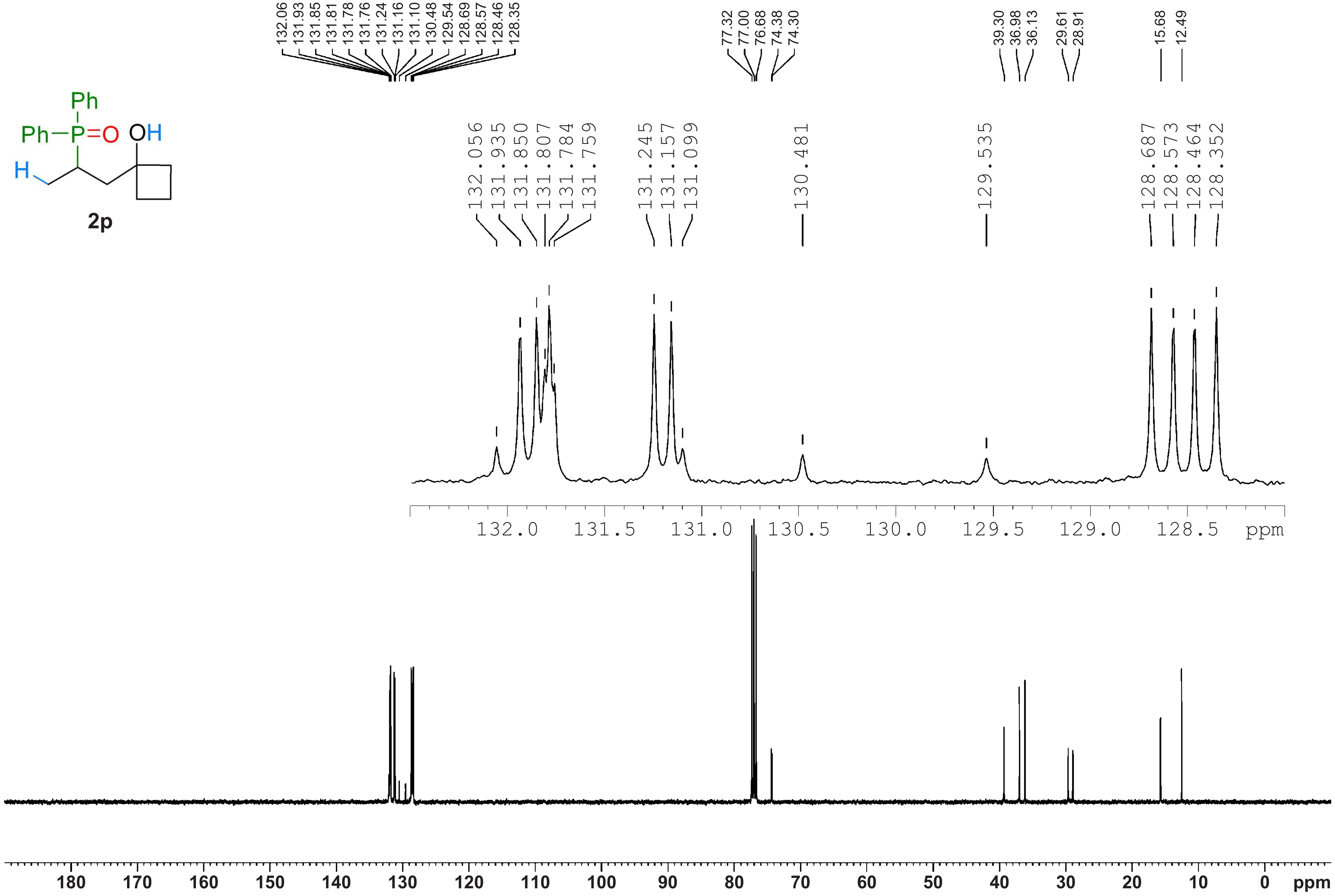

2p

^{31}P NMR (162 MHz, $CDCl_3$)

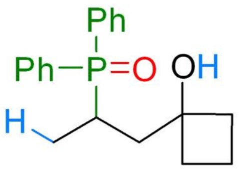

2p

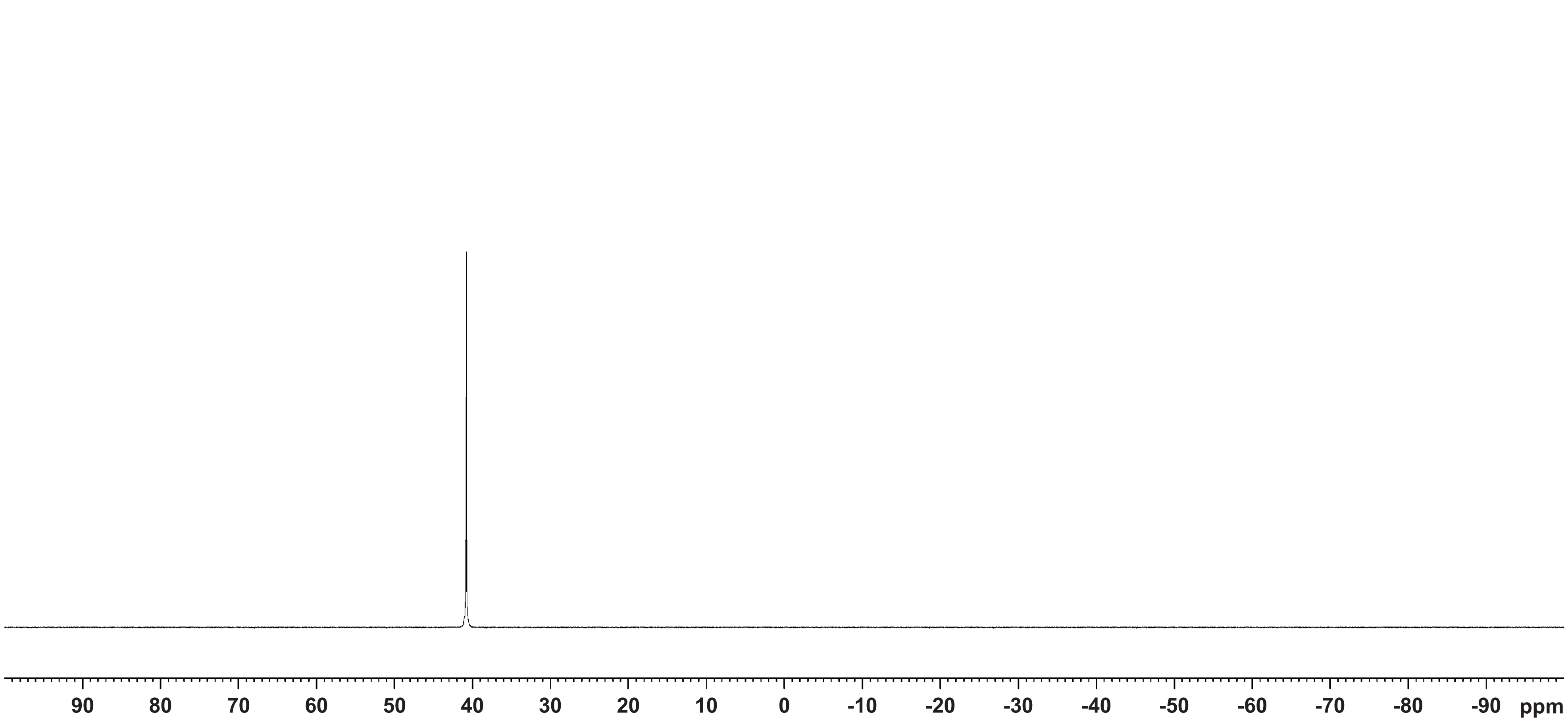

^{1}H NMR (400 MHz, $CDCl_3$)

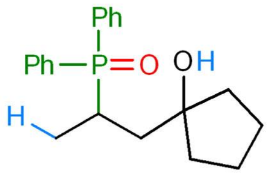

2q

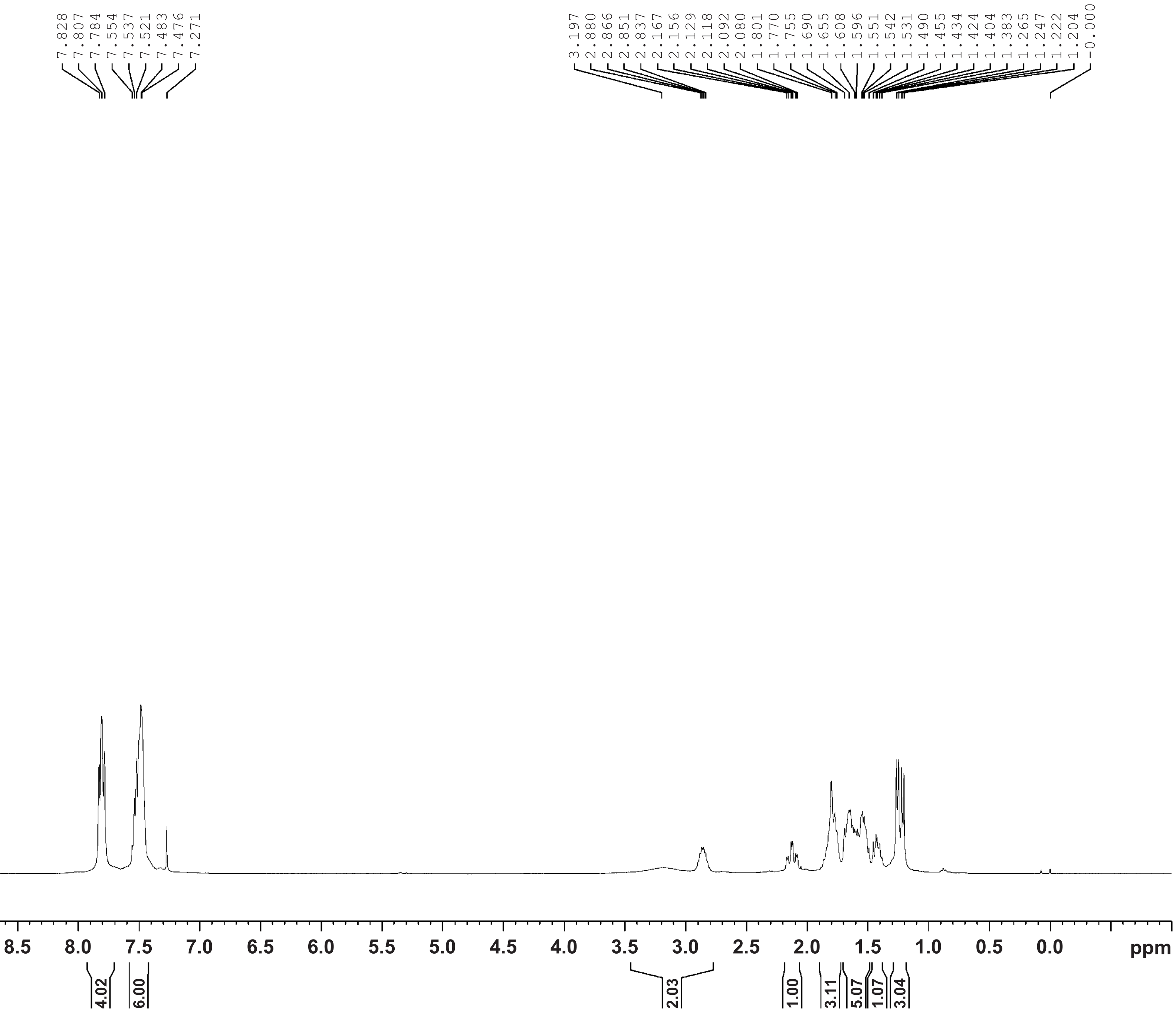

^{13}C NMR (100.6 MHz, $CDCl_3$)

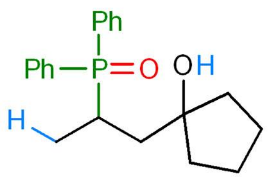

2q

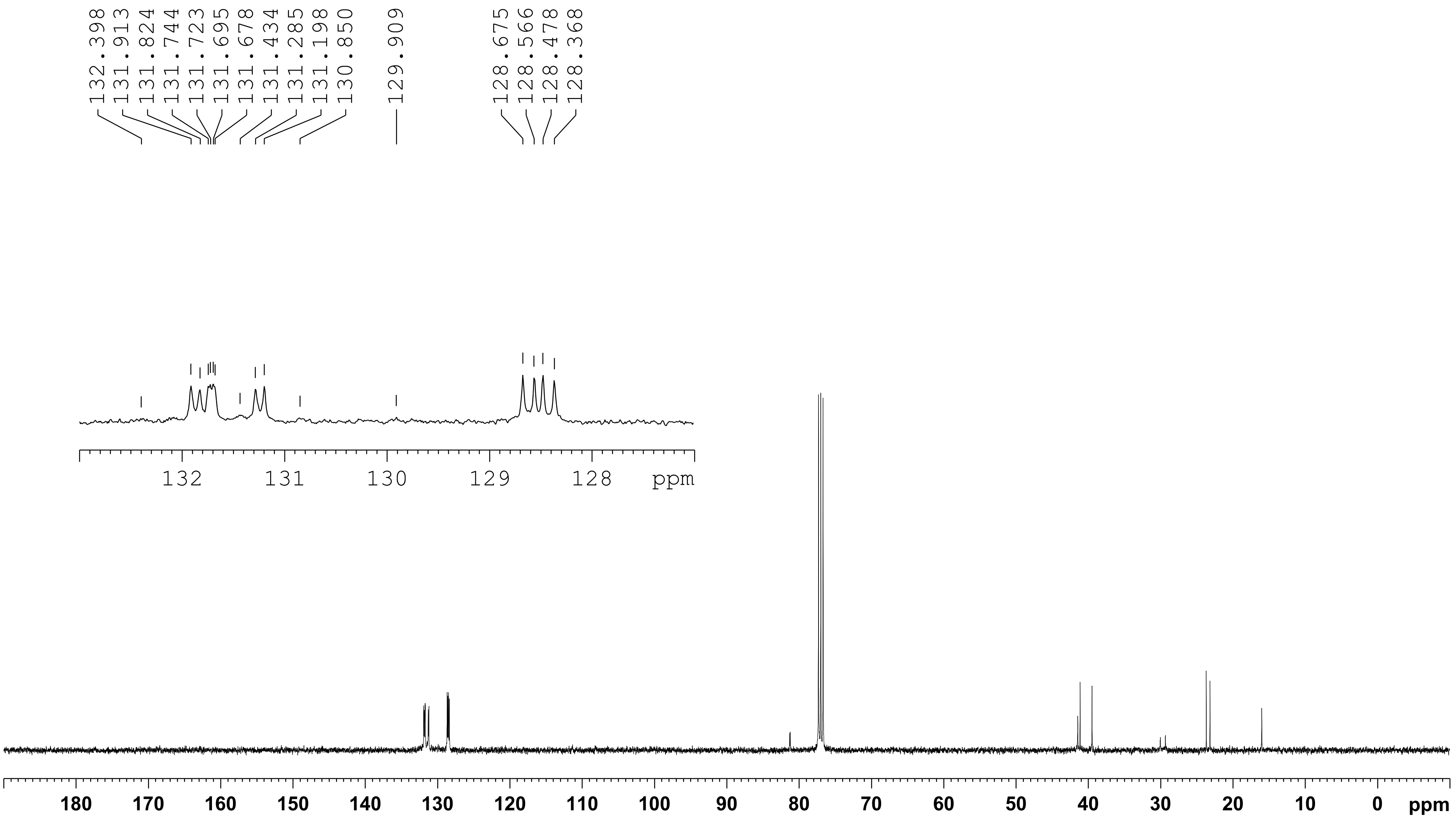

^{31}P NMR (162 MHz, $CDCl_3$)

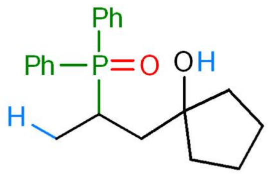

2q

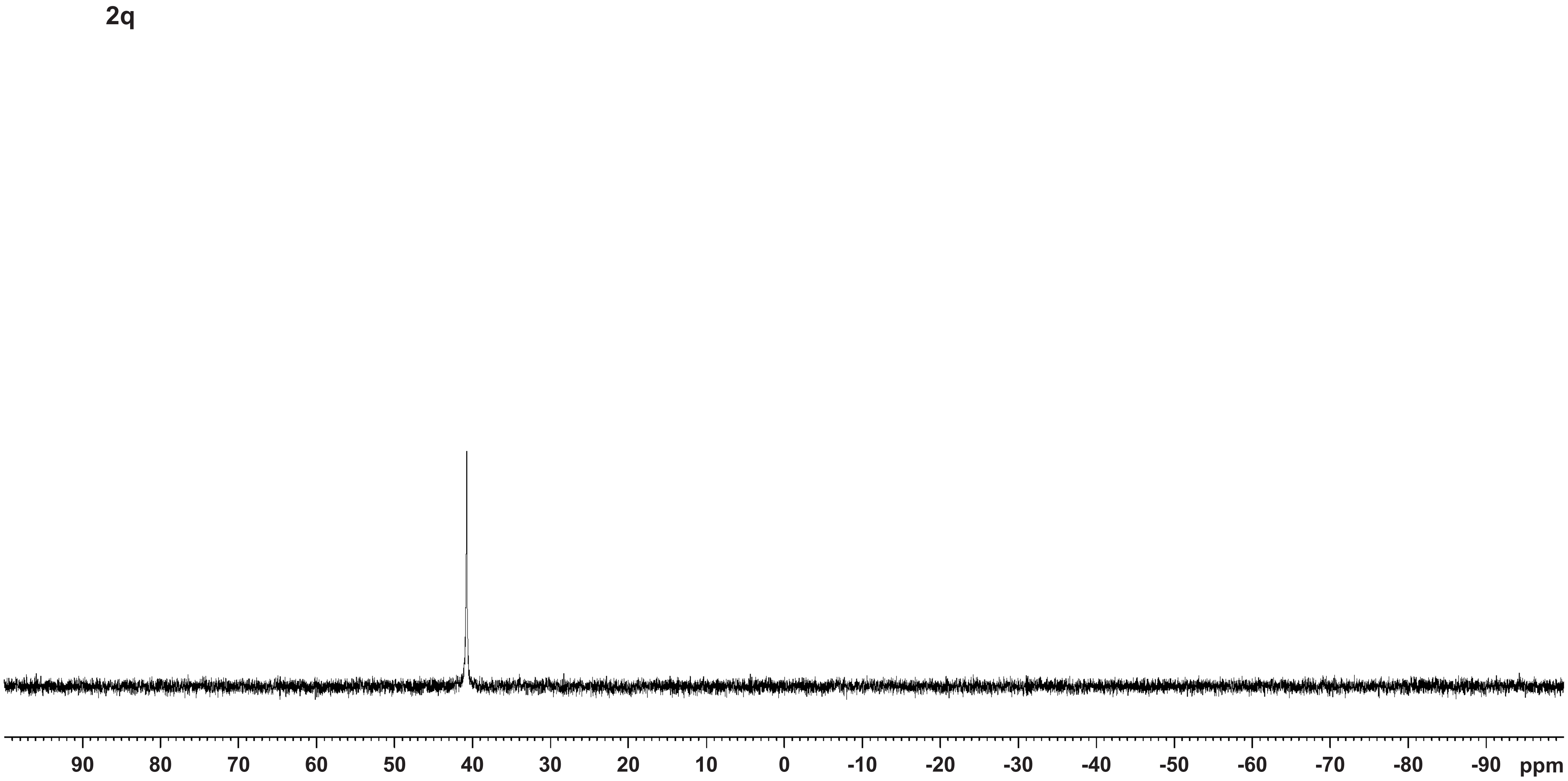

^{1}H NMR (400 MHz, $CDCl_3$)

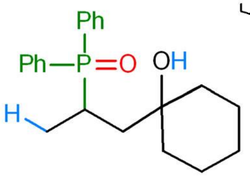

2r

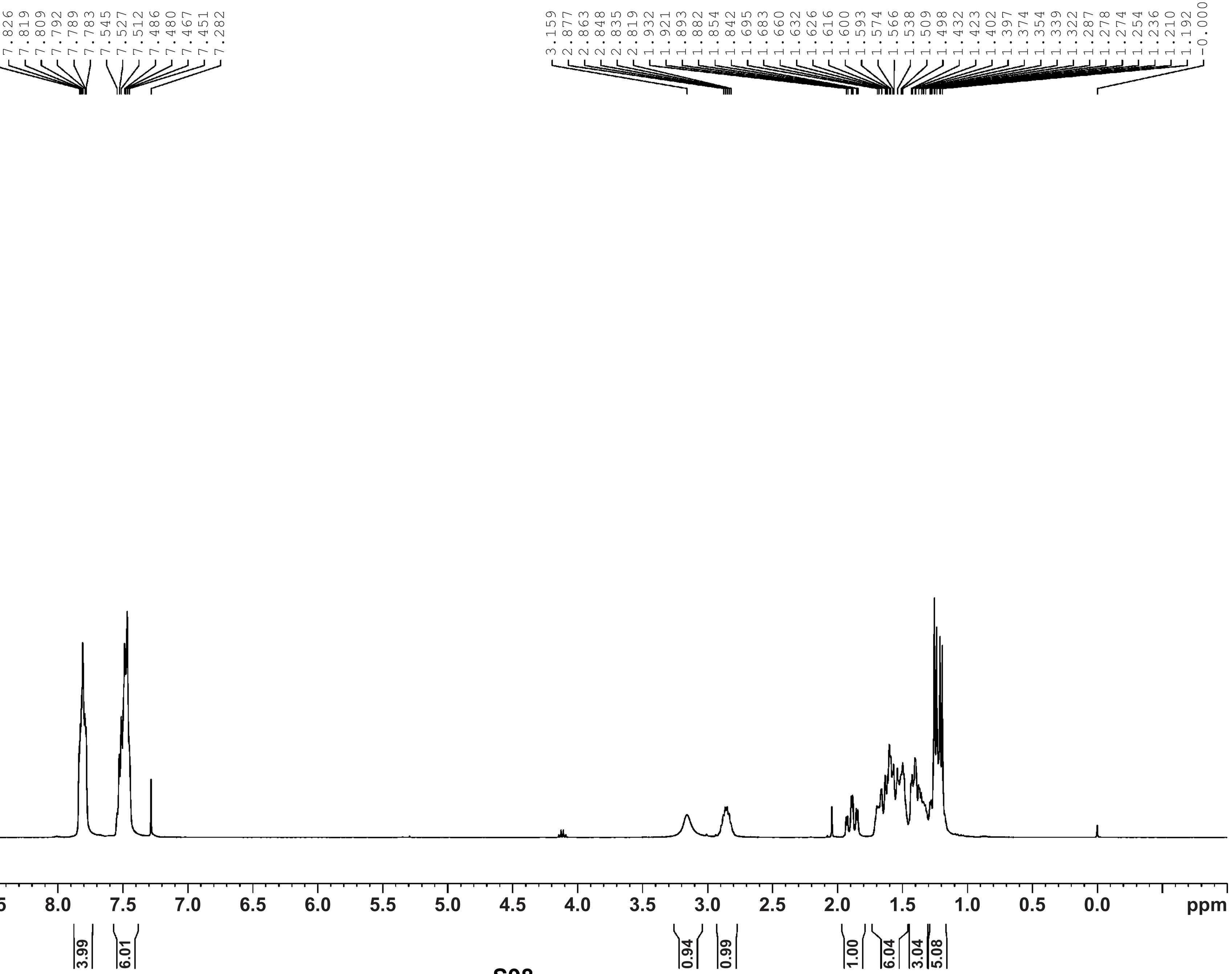

^{13}C NMR (100.6 MHz, $CDCl_3$)

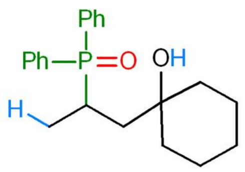

2r

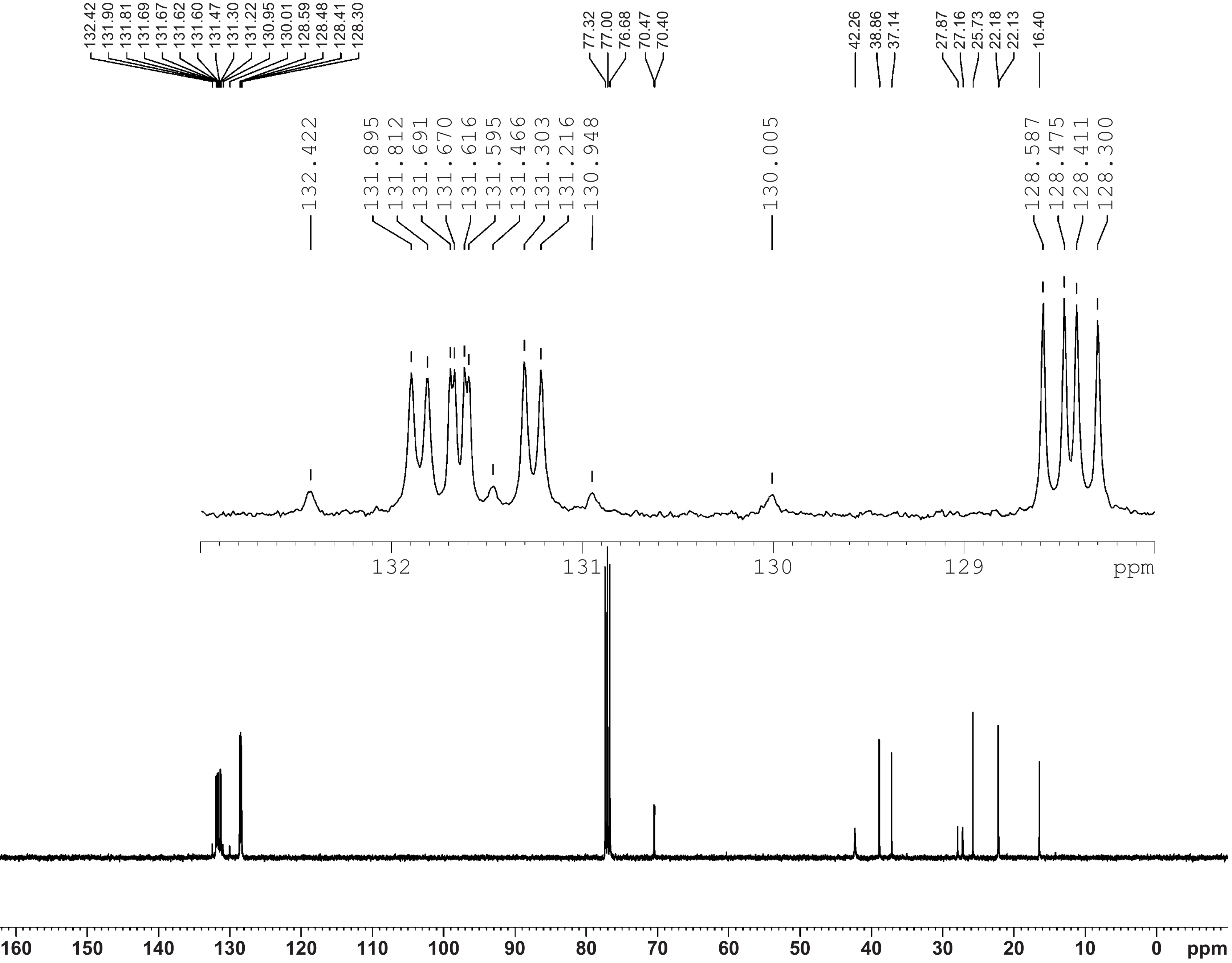

^{31}P NMR (162 MHz, $CDCl_3$)

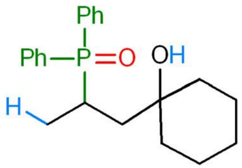

2r

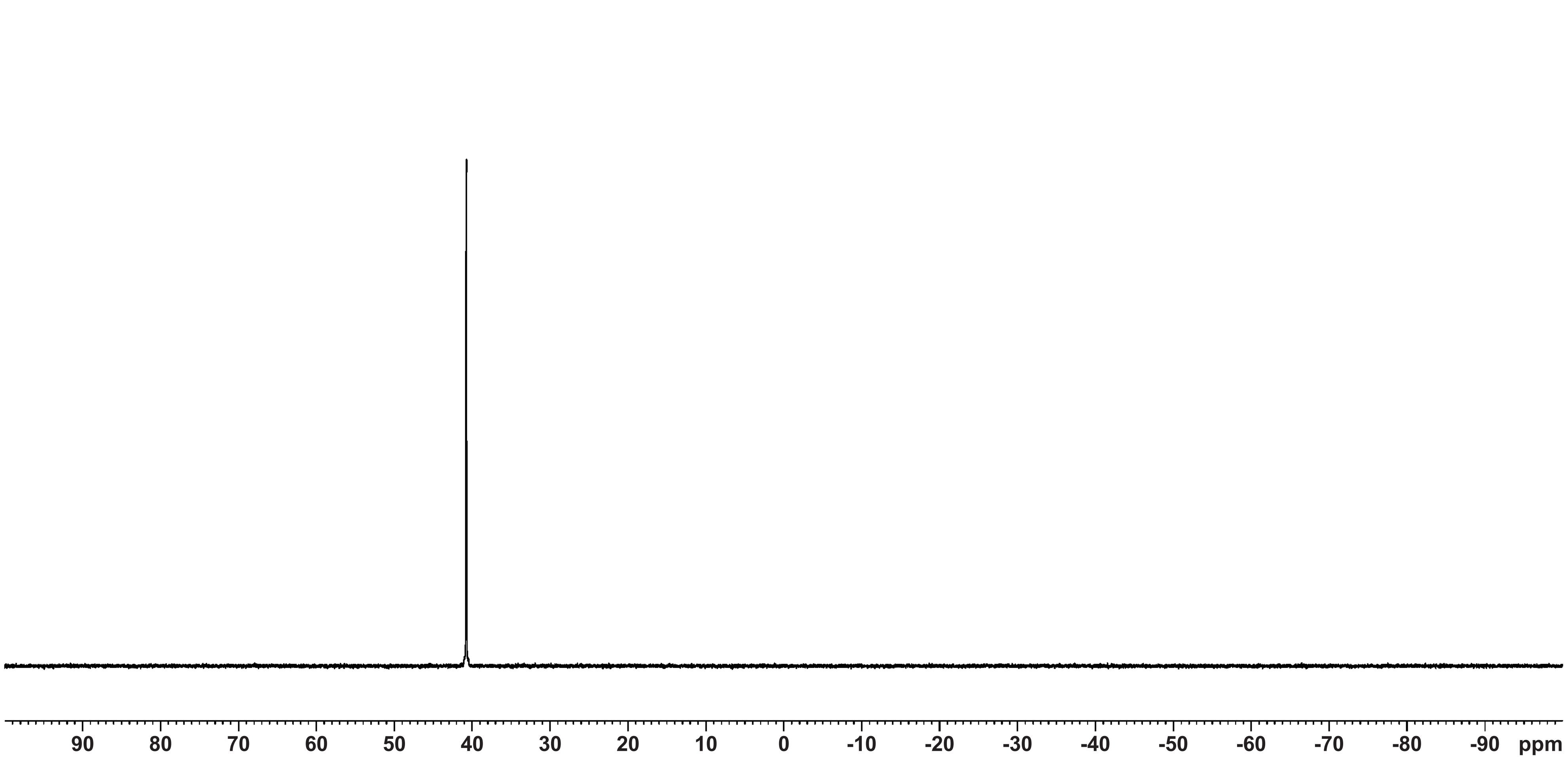

^{1}H NMR (400 MHz, $CDCl_3$)

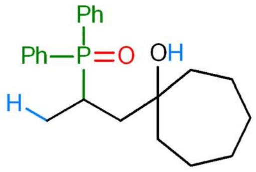

2s

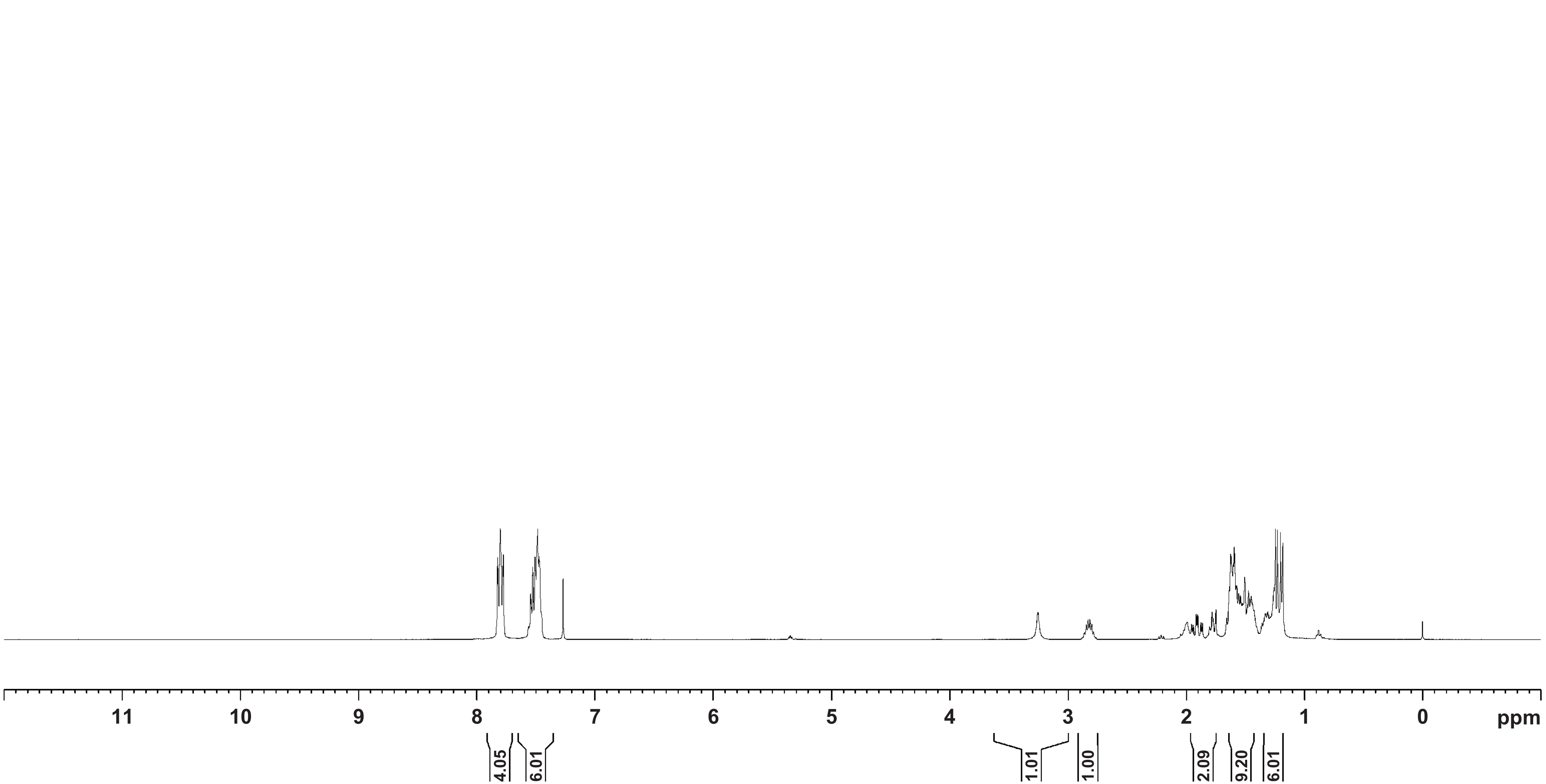

^{13}C NMR (100.6 MHz, $CDCl_3$)

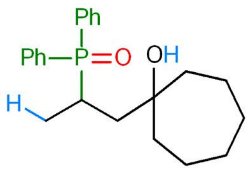

2s

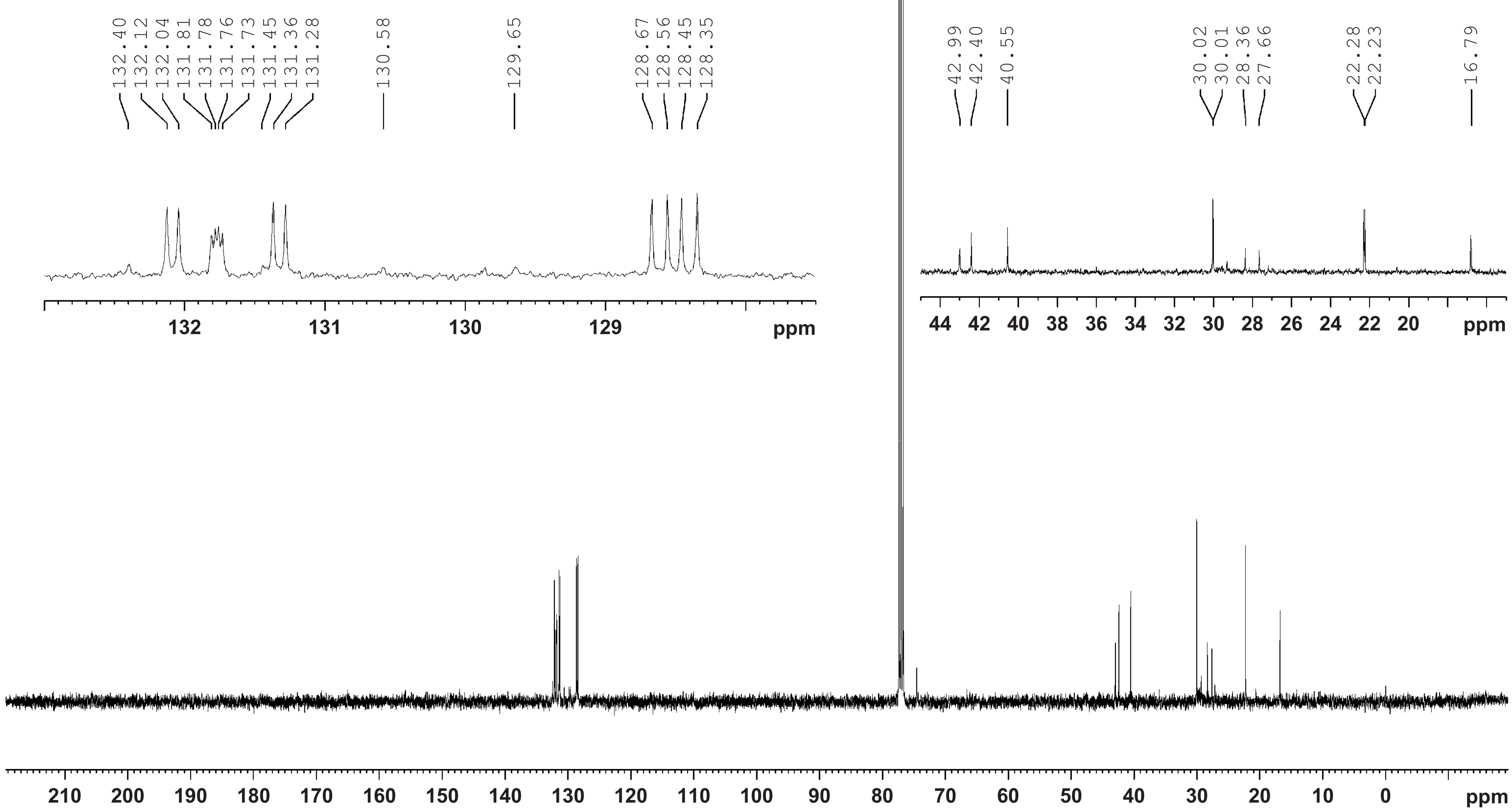

^{31}P NMR (162 MHz, CDCl$_3$)

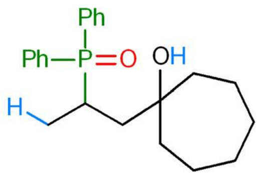

2s

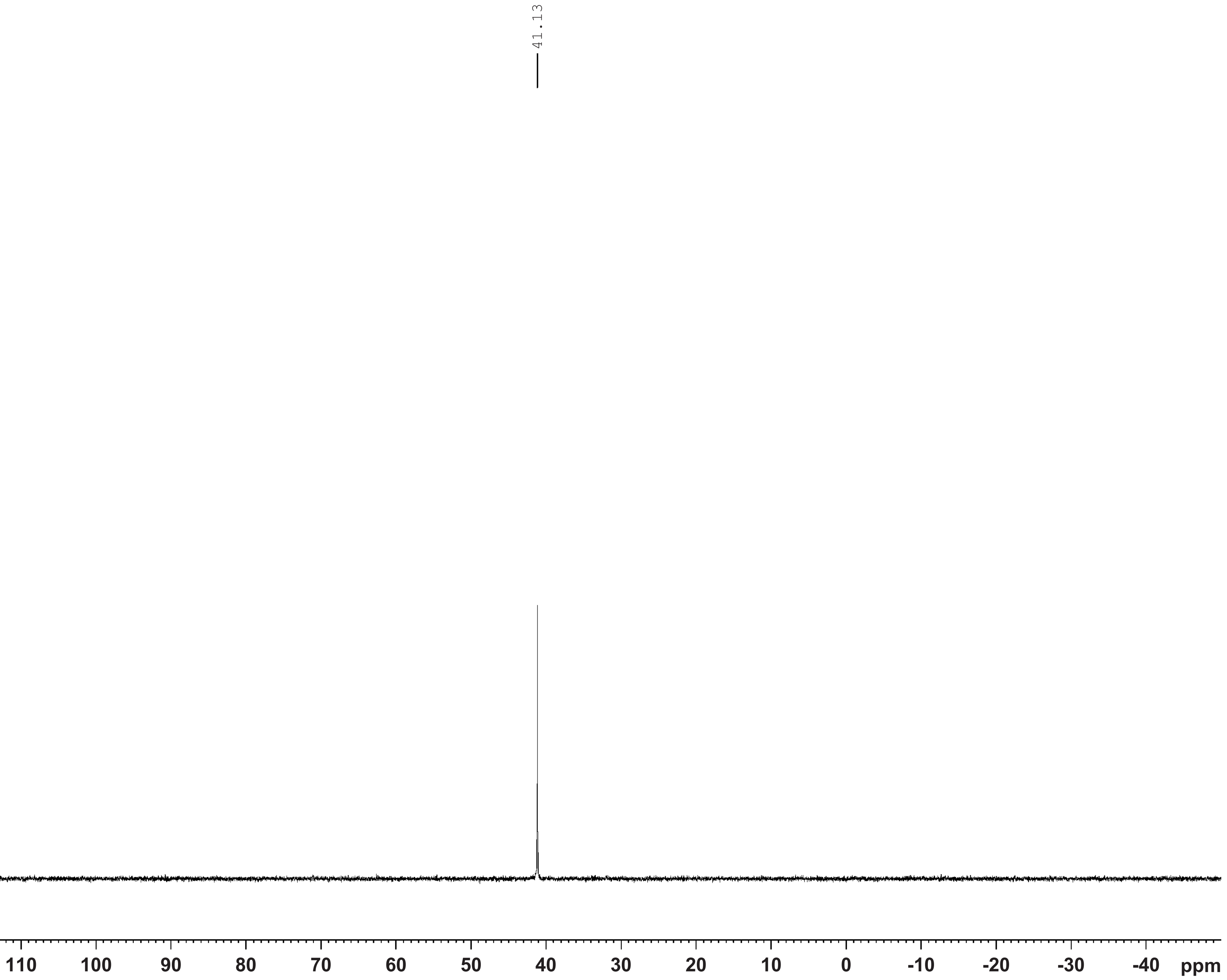

^{1}H NMR (400 MHz, $CDCl_3$)

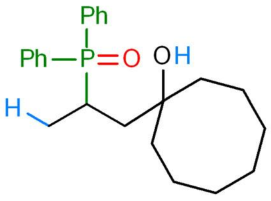

2t

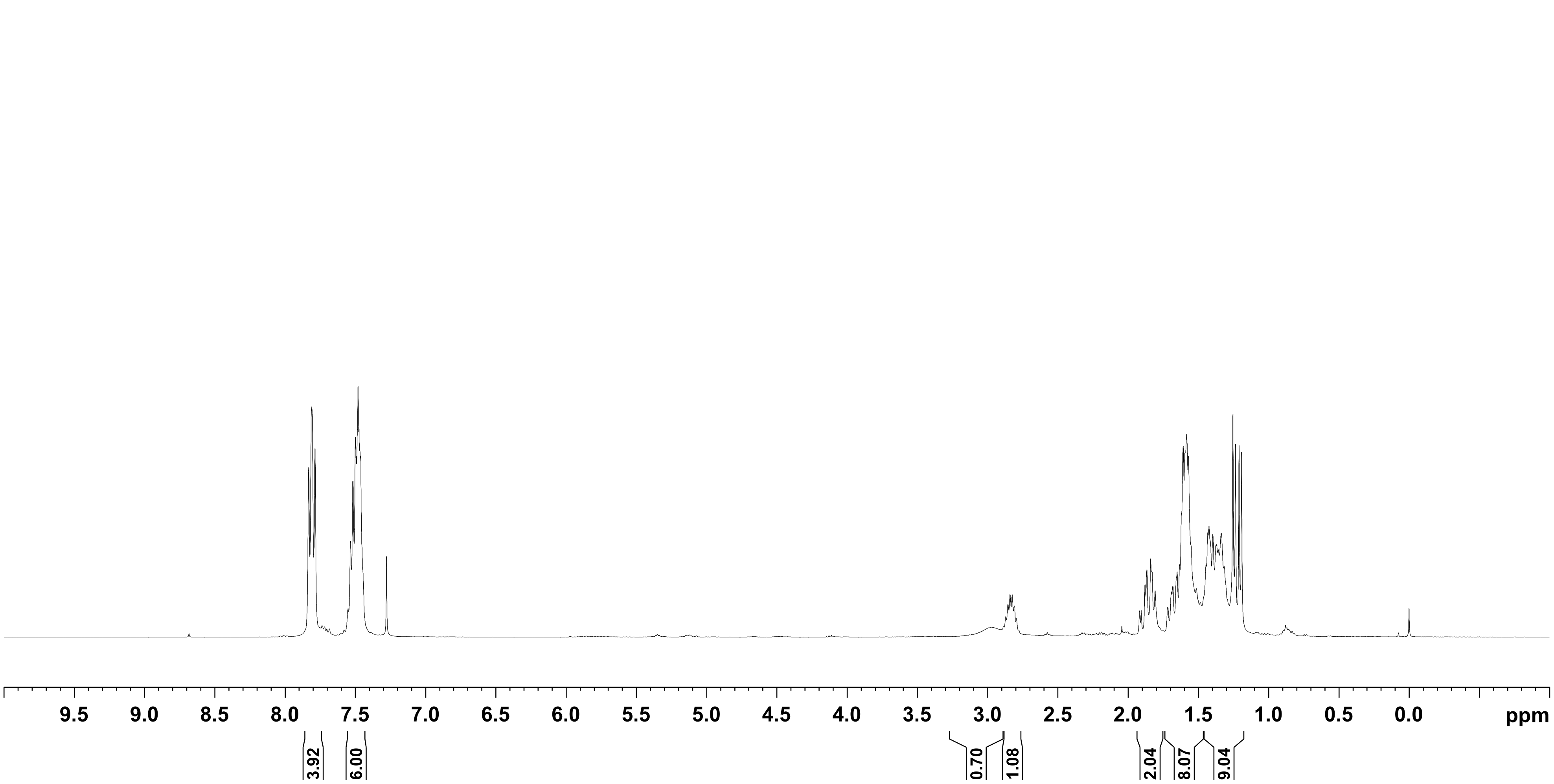

^{13}C NMR (100.6 MHz, $CDCl_3$)

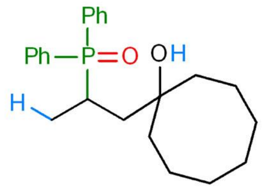

2t

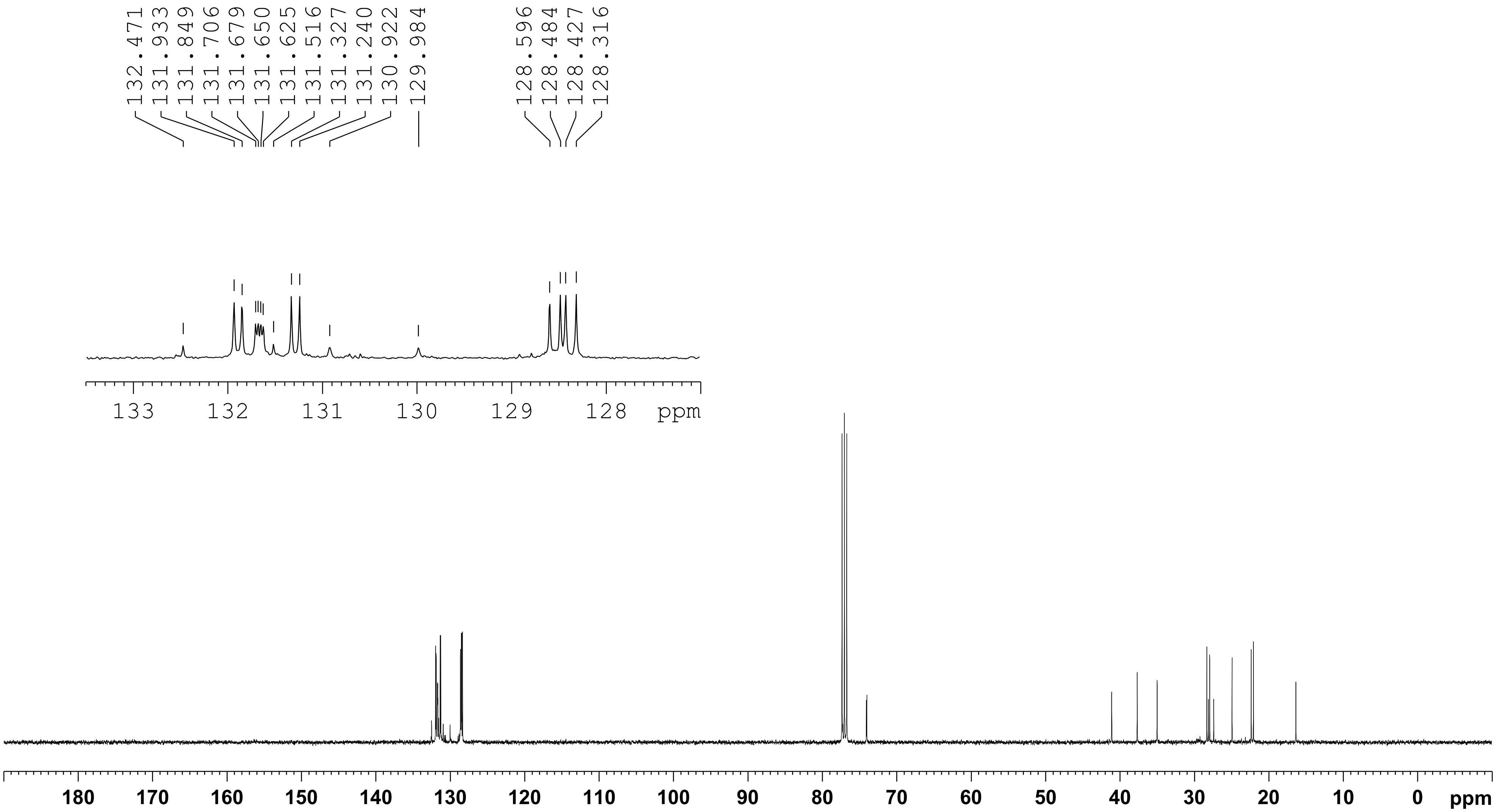

^{31}P NMR (162 MHz, $CDCl_3$)

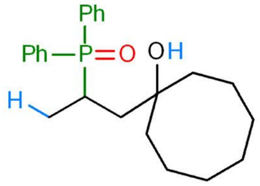

2t

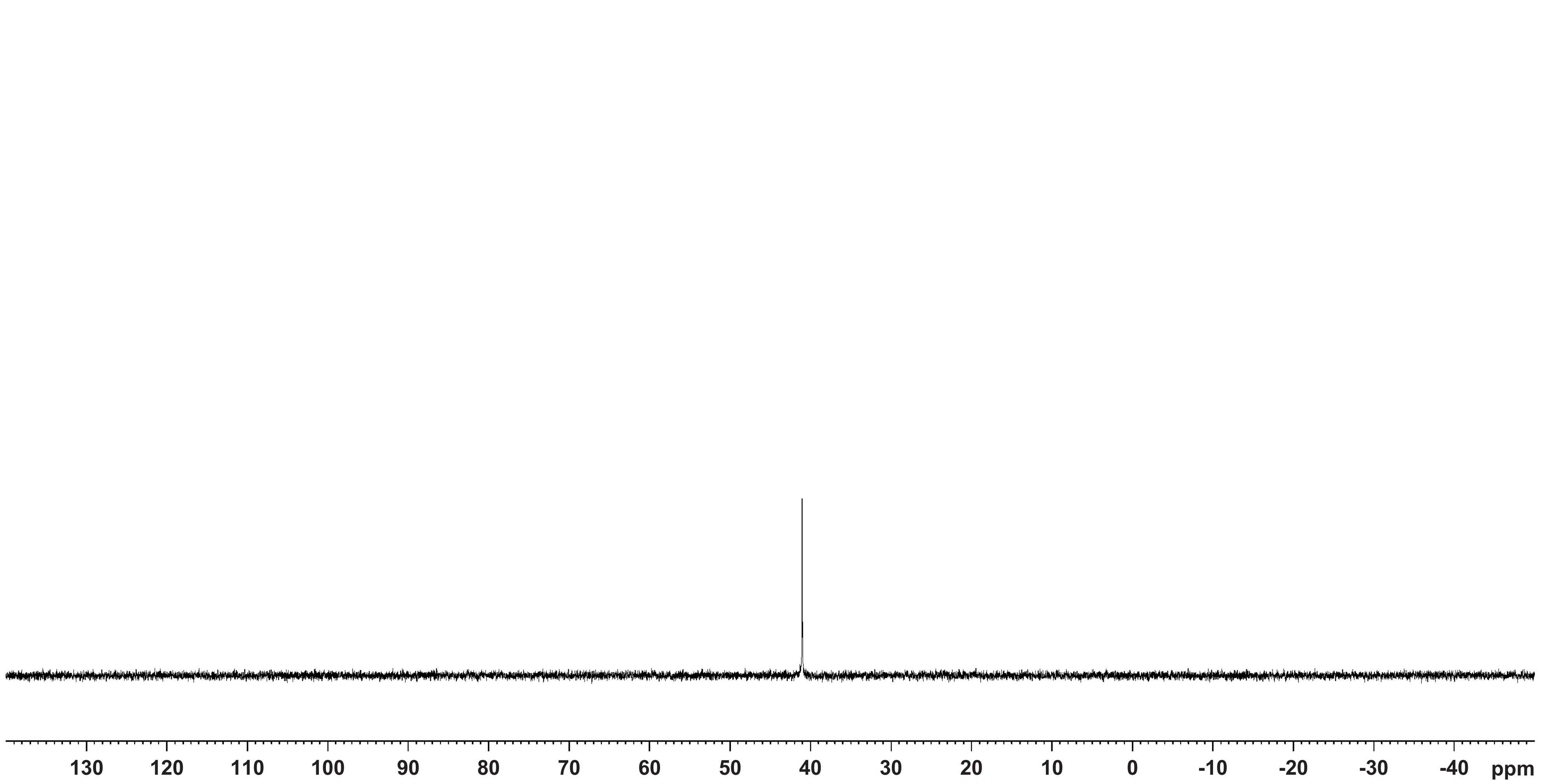

^{1}H NMR (400 MHz, $CDCl_3$)

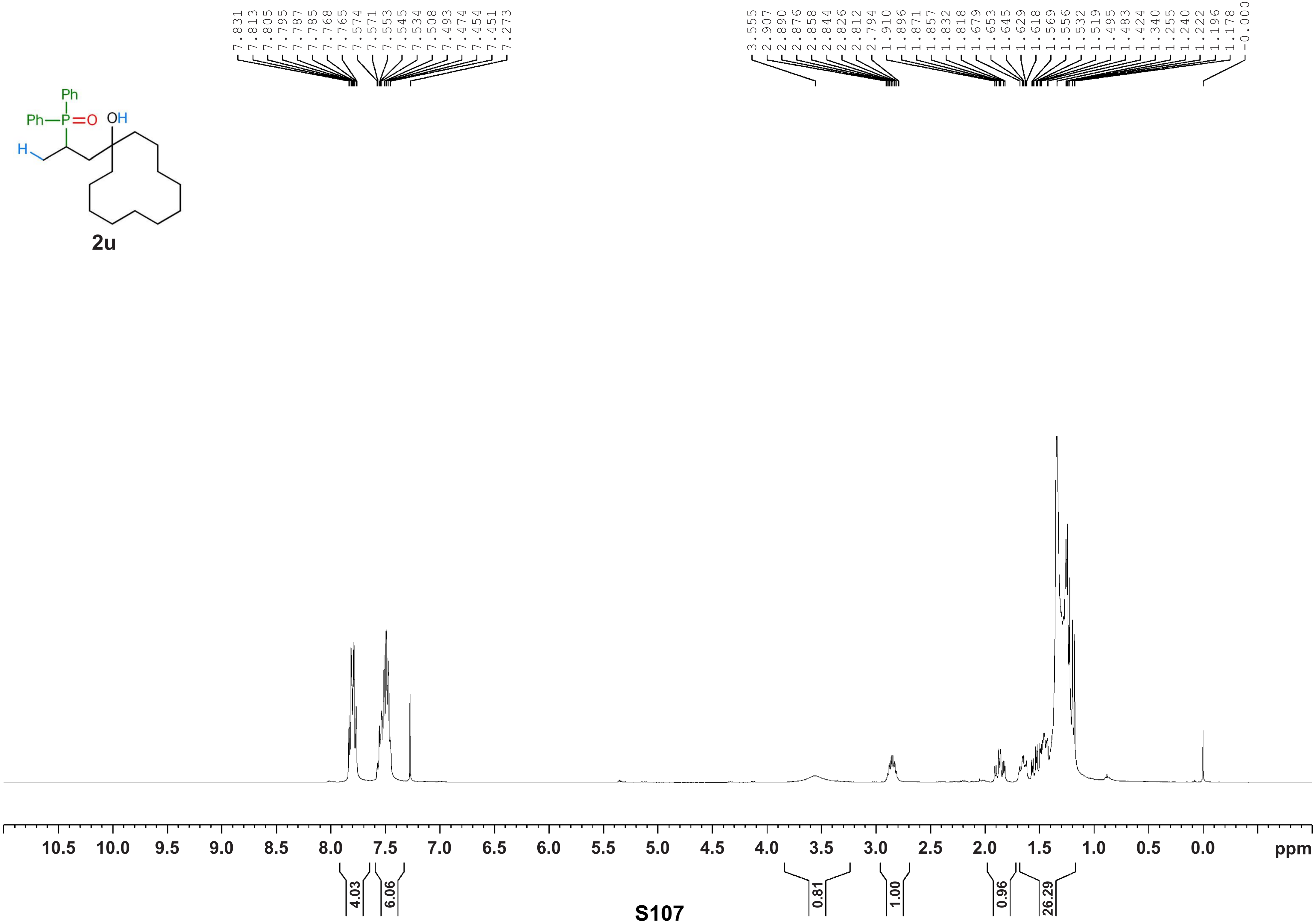

^{13}C NMR (100.6 MHz, $CDCl_3$)

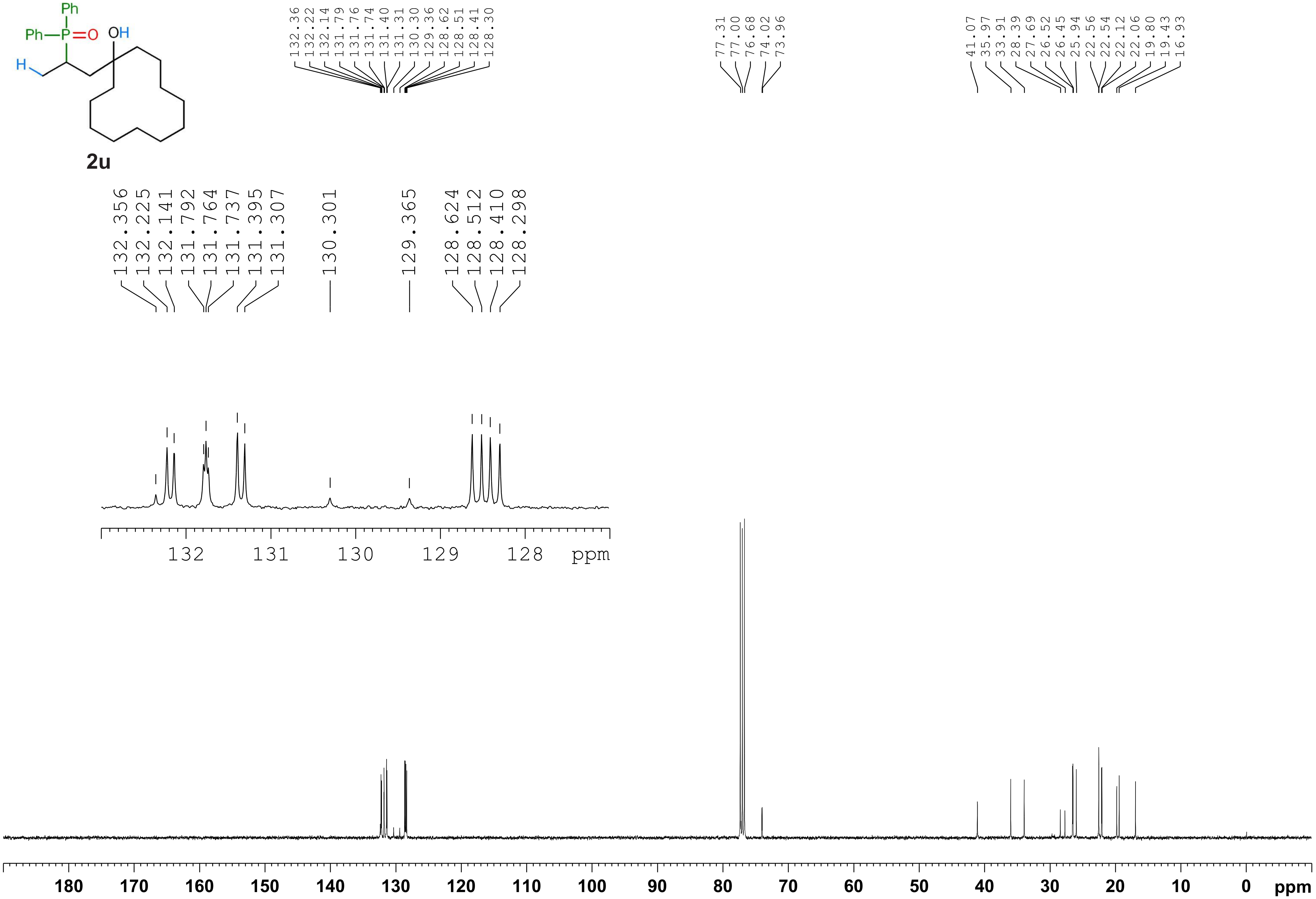

^{31}P NMR (162 MHz, $CDCl_3$)

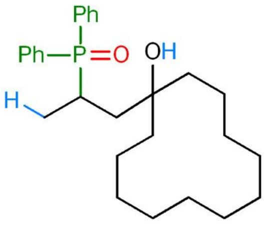

2u

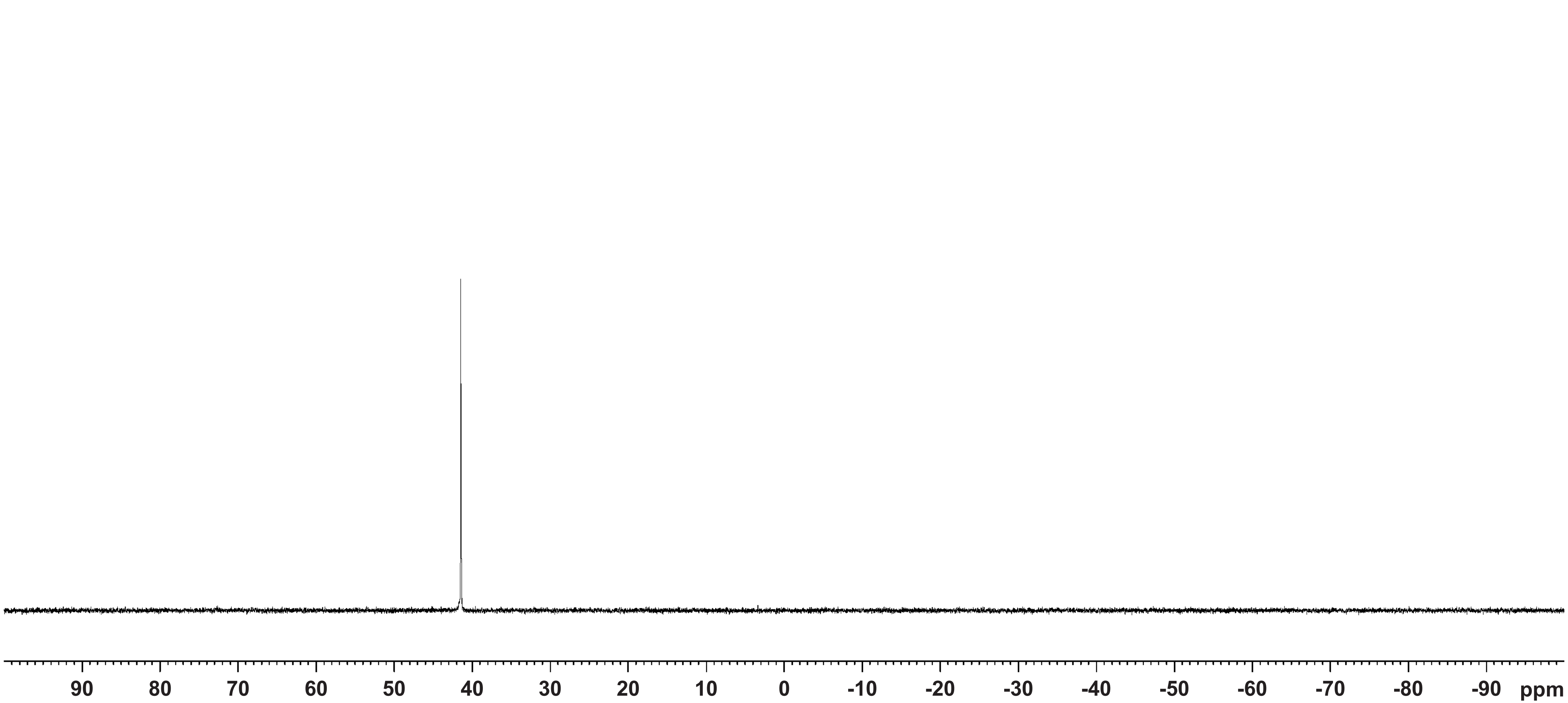

^{1}H NMR (400 MHz, $CDCl_3$)

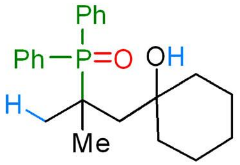

2v

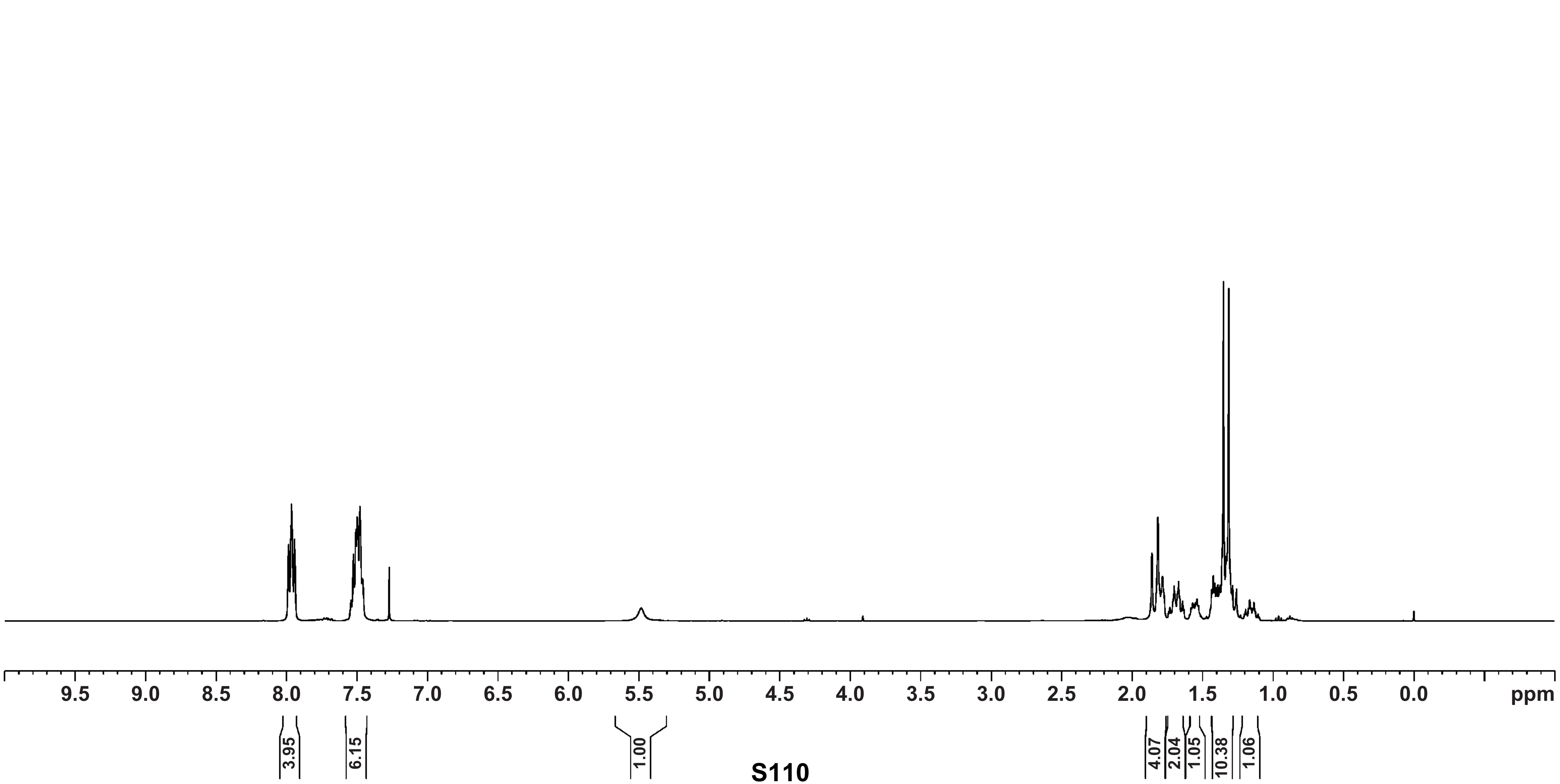

^{13}C NMR (100.6 MHz, $CDCl_3$)

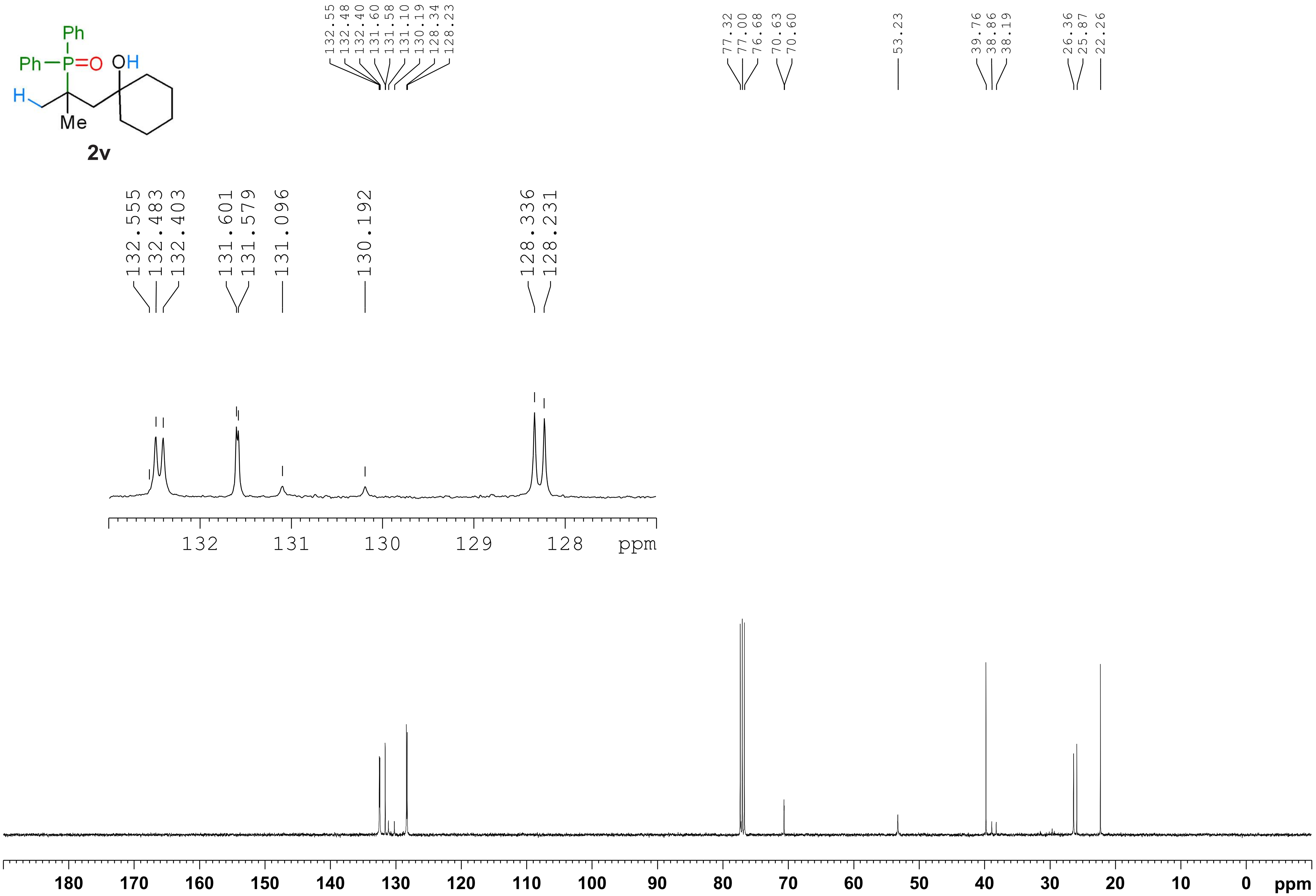

^{31}P NMR (162 MHz, $CDCl_3$)

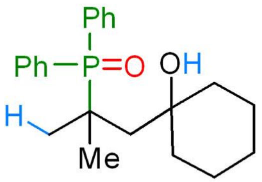

2v

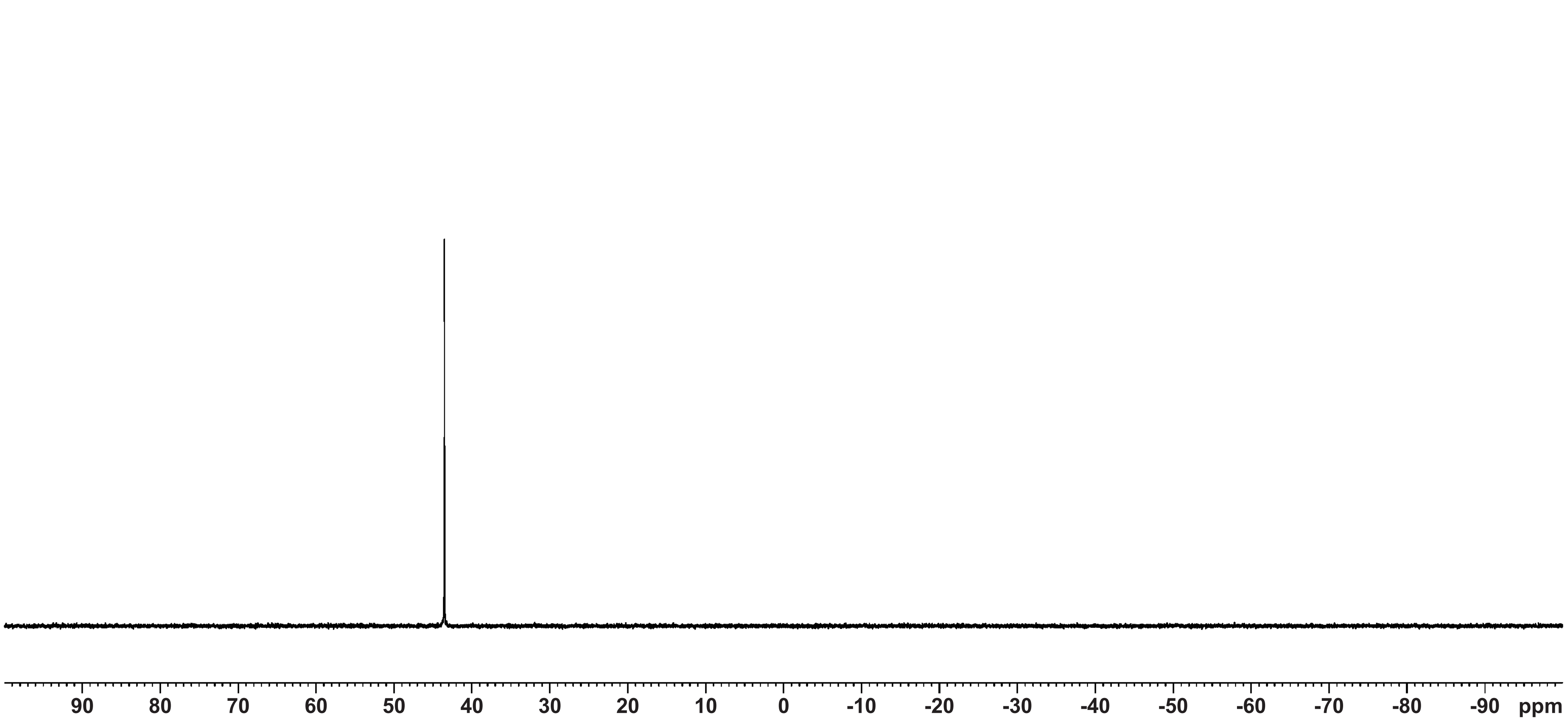

^{1}H NMR (400 MHz, $CDCl_3$)

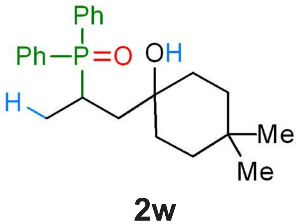

2w

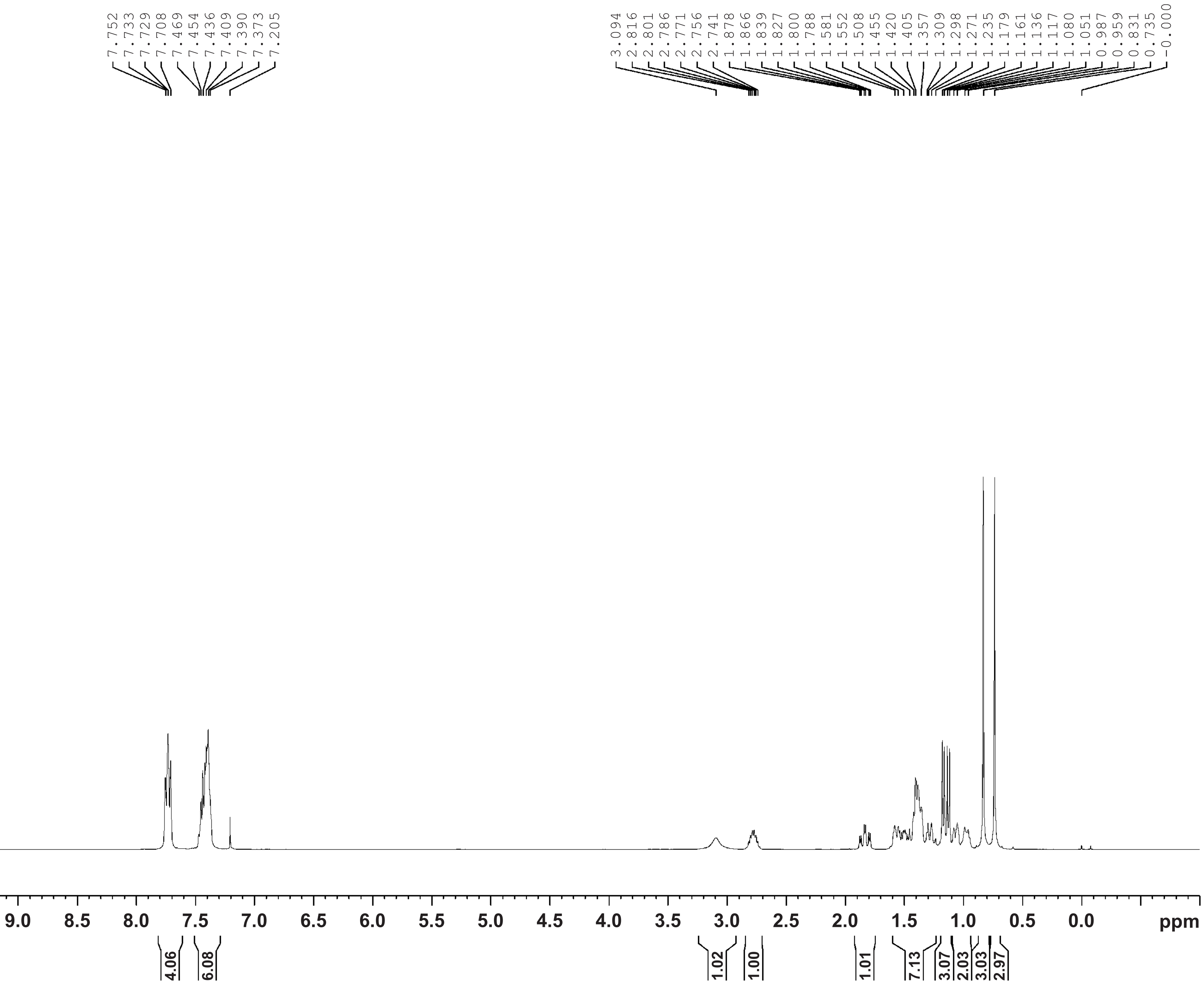

^{13}C NMR (100.6 MHz, $CDCl_3$)

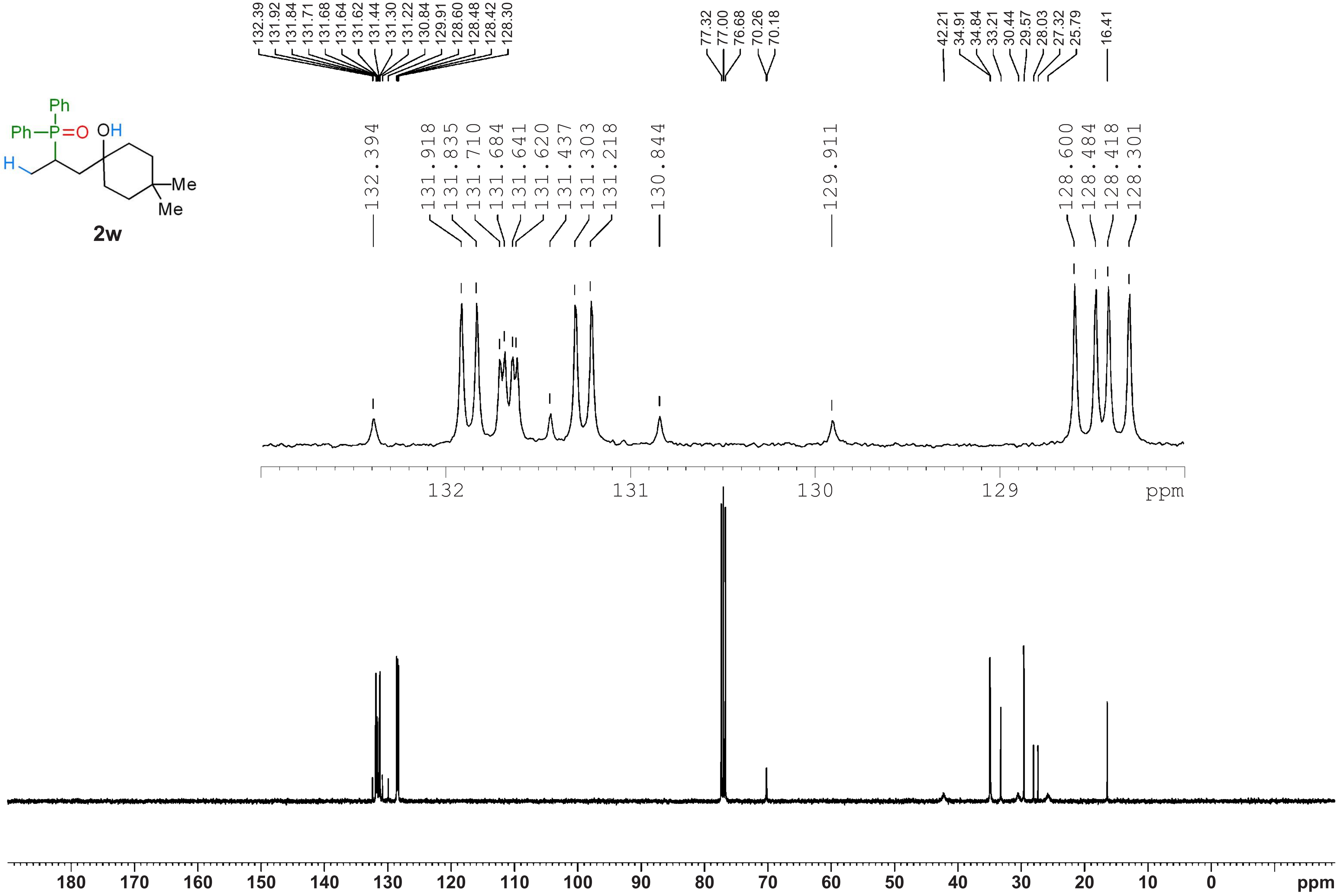

^{31}P NMR (162 MHz, $CDCl_3$)

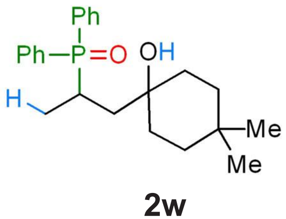

2w

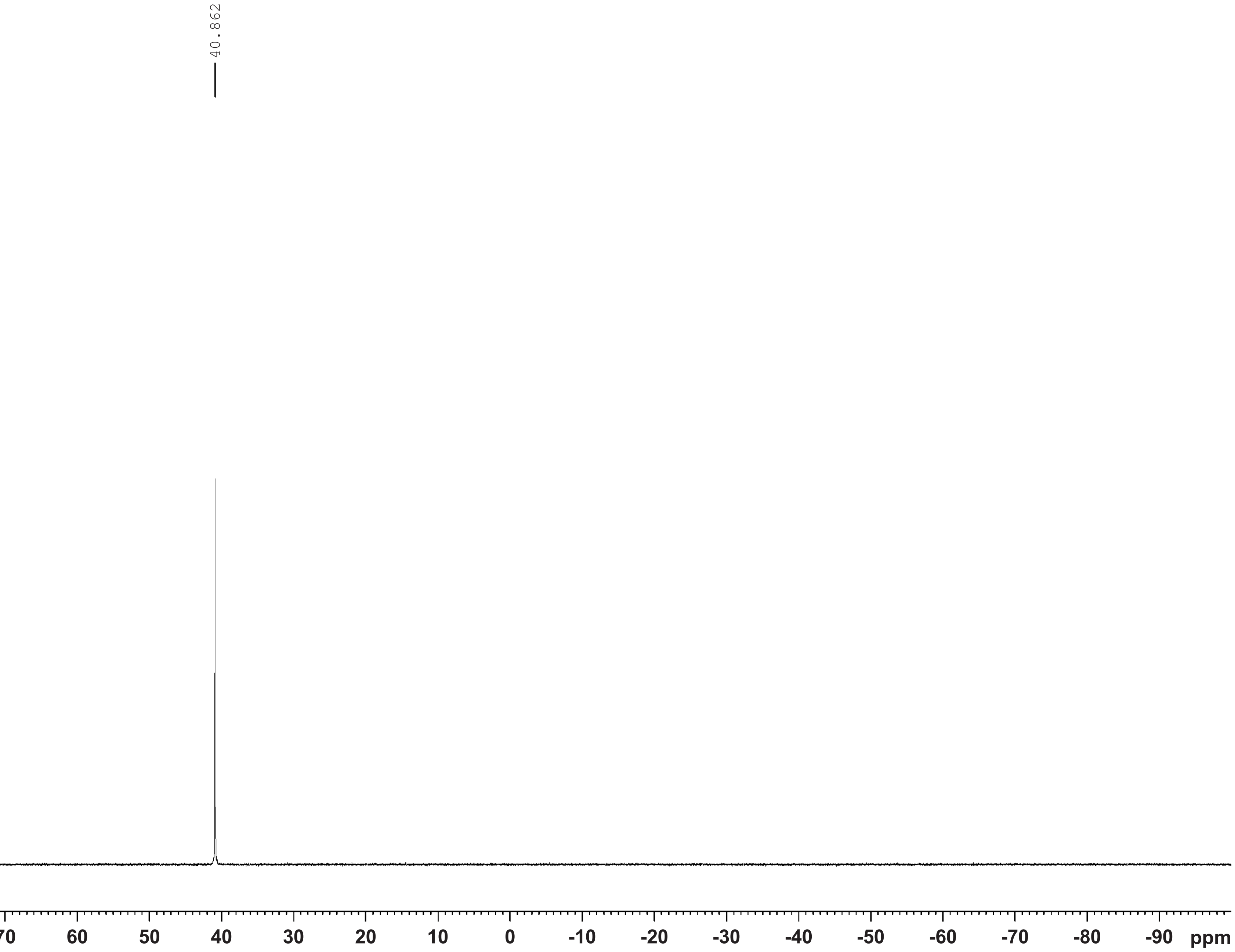

^{1}H NMR (400 MHz, $CDCl_3$)

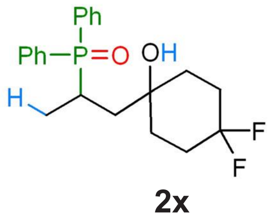

2x

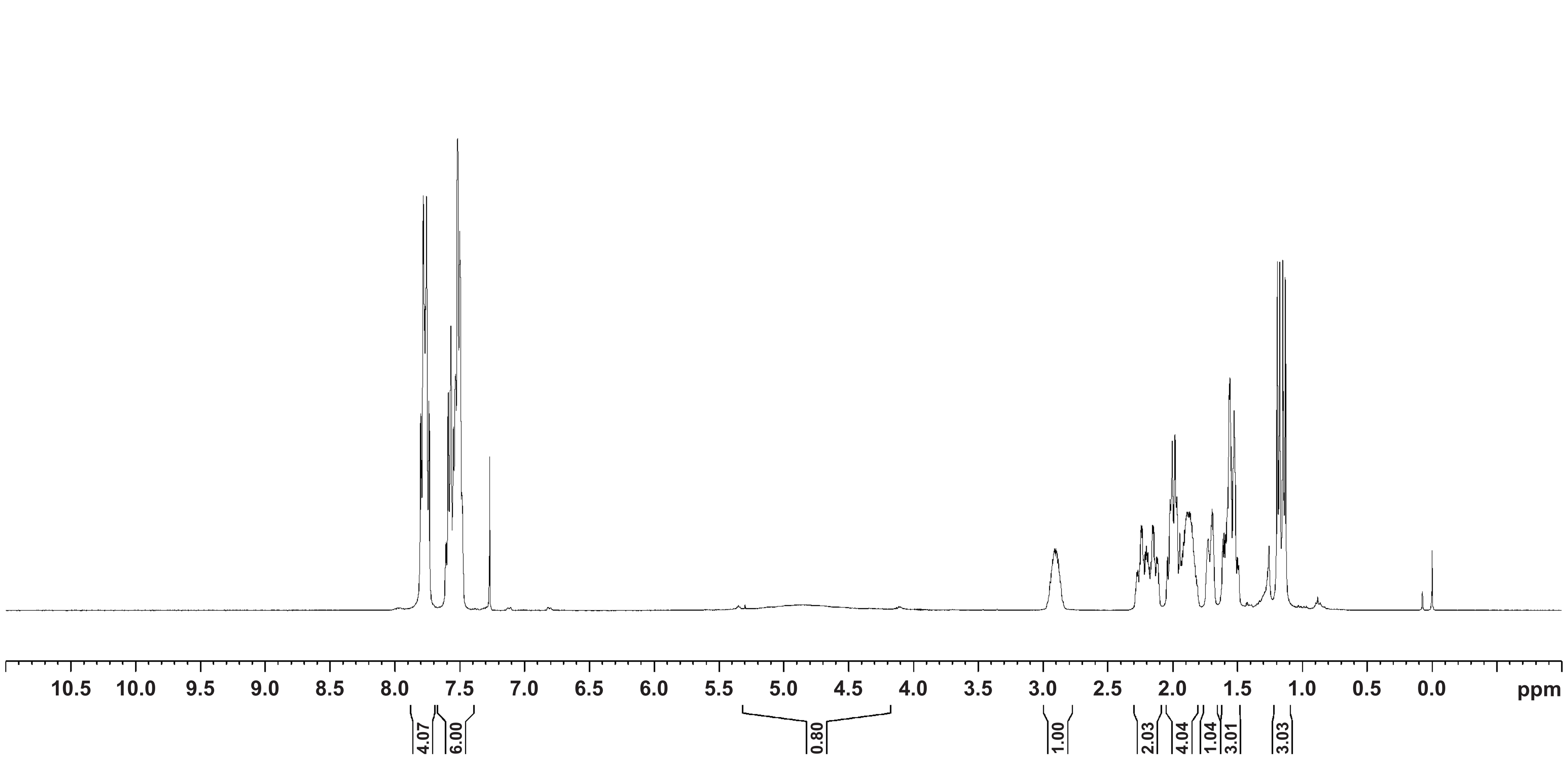

^{13}C NMR (100.6 MHz, $CDCl_3$)

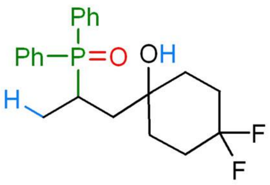

2x

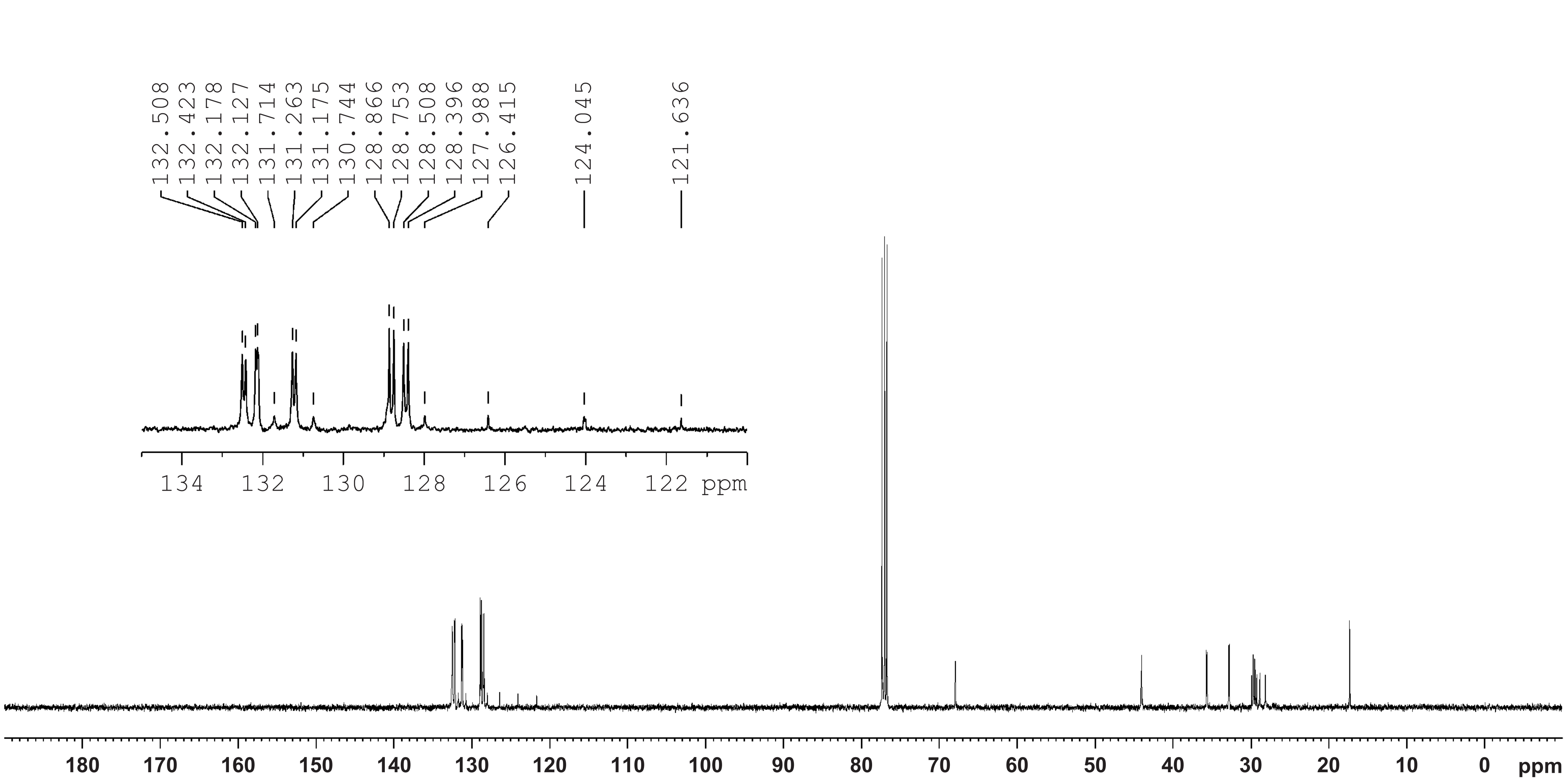

^{19}F NMR (376 MHz, $CDCl_3$)

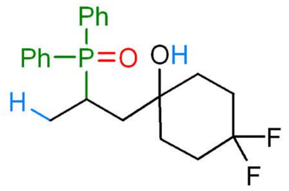

2x

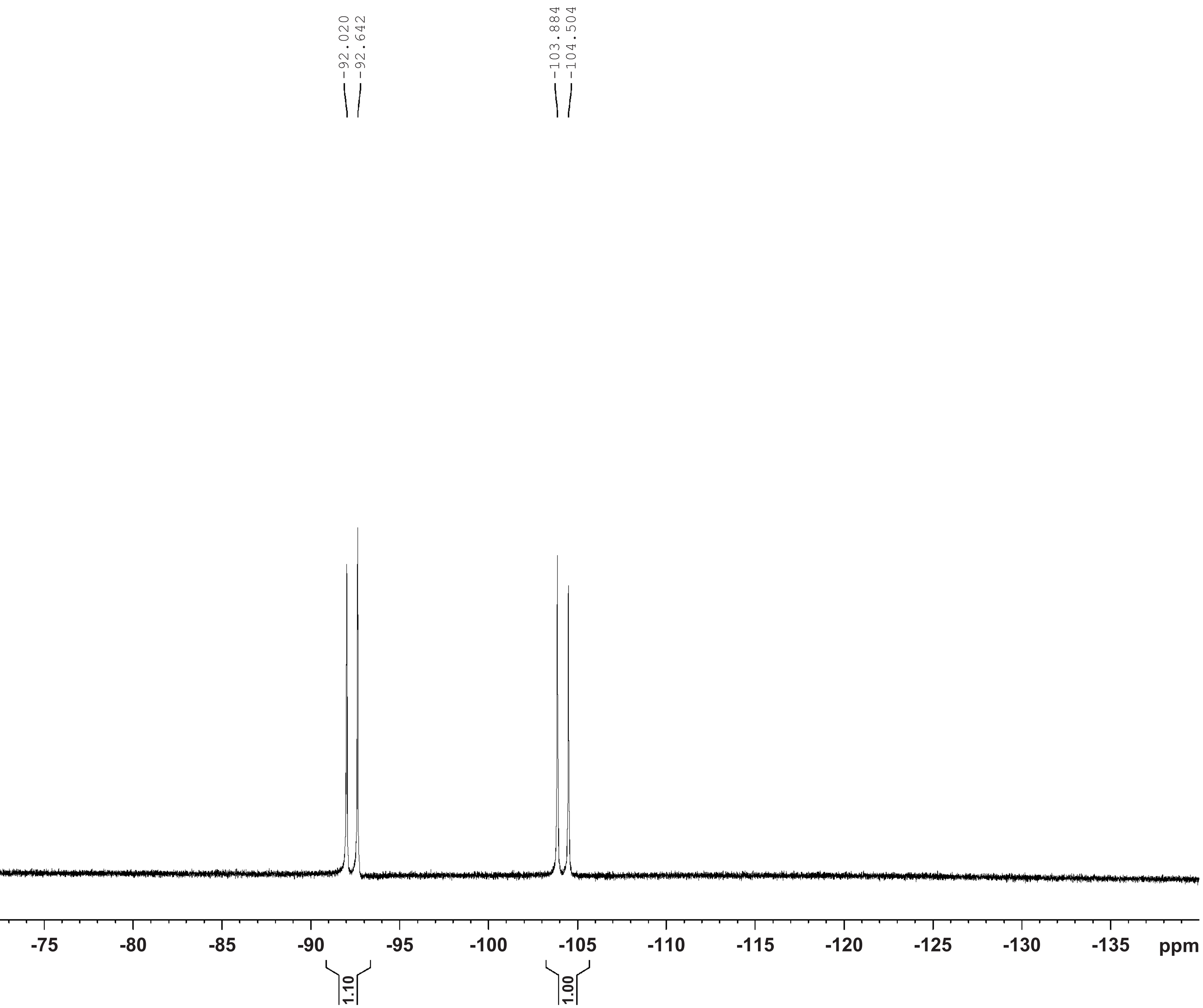

^{31}P NMR (162 MHz, $CDCl_3$)

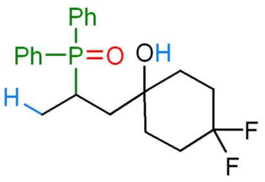

2x

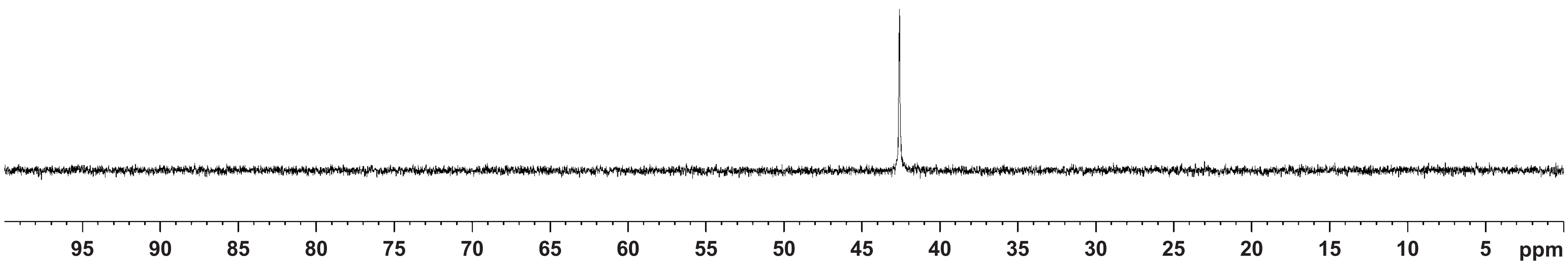

^{1}H NMR (400 MHz, $CDCl_3$)

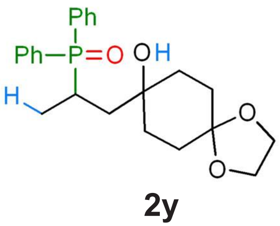

2y

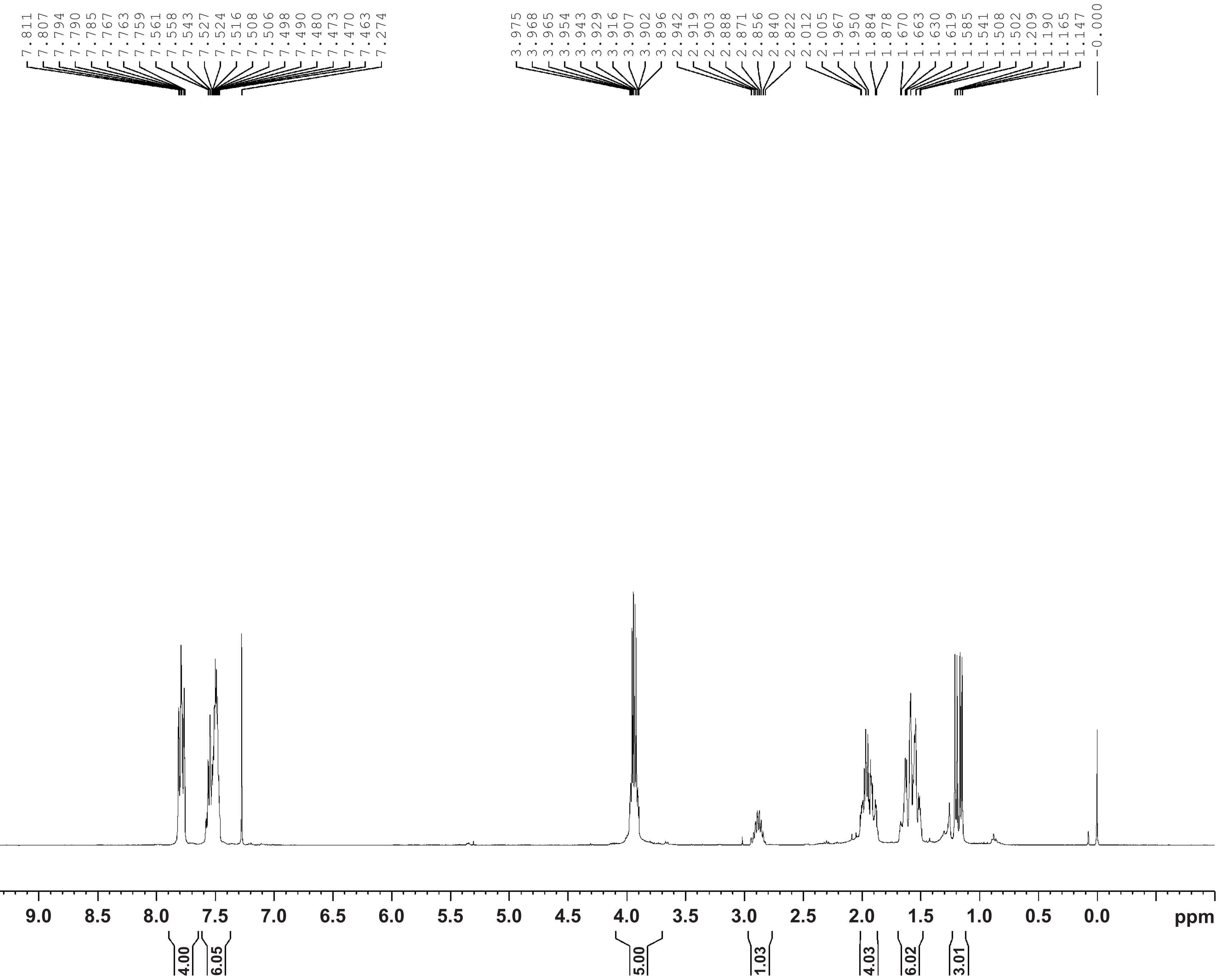

^{13}C NMR (100.6 MHz, $CDCl_3$)

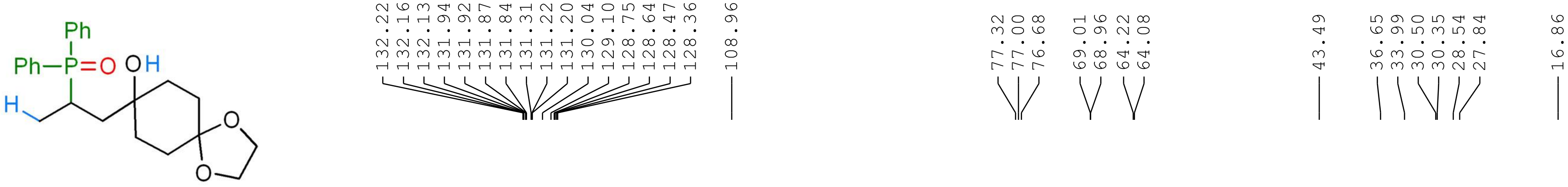

2y

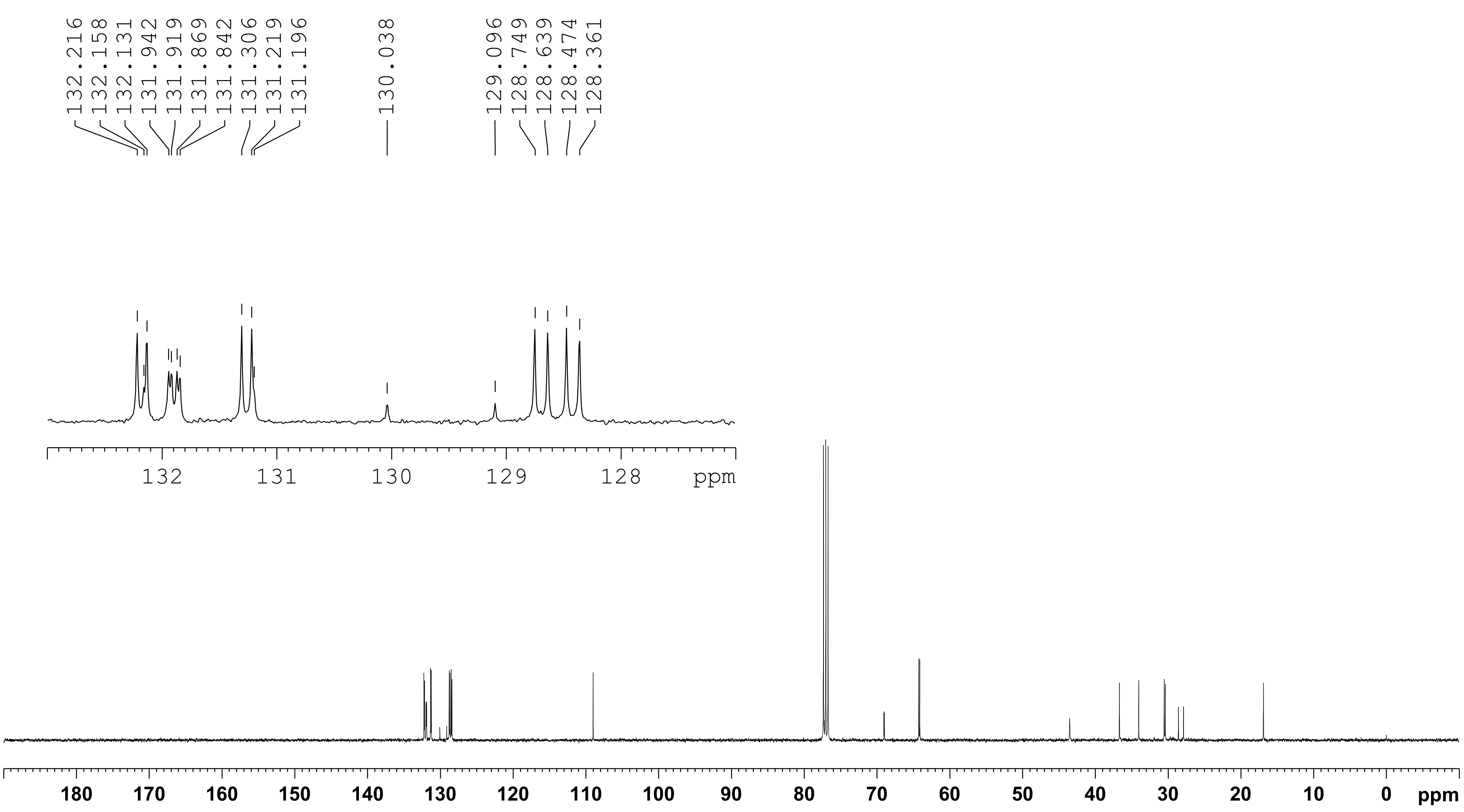

^{31}P NMR (162 MHz, CDCl$_3$)

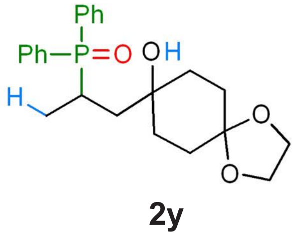

2y

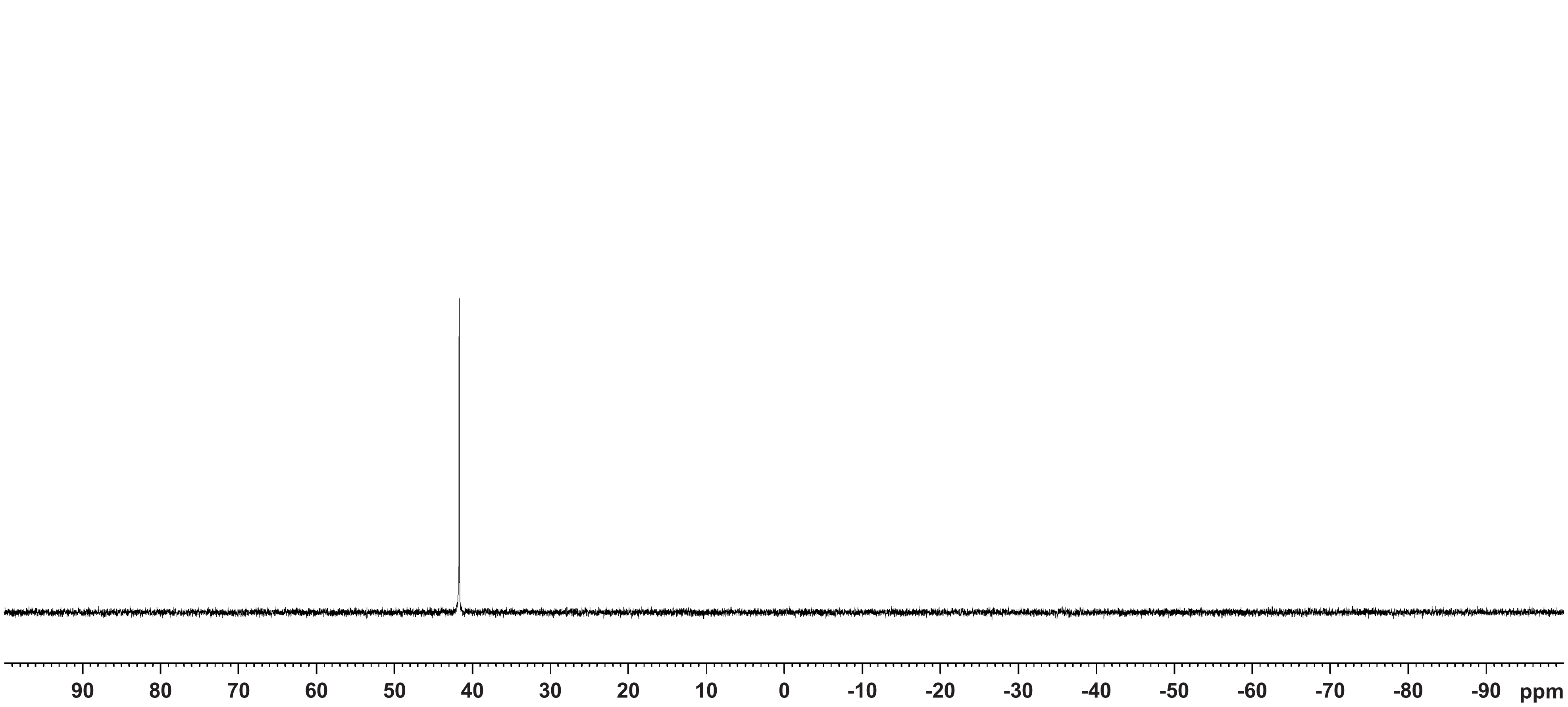

^{1}H NMR (400 MHz, $CDCl_3$)

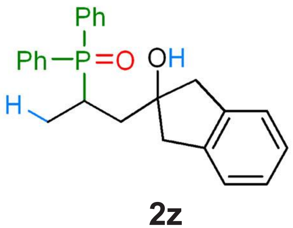

2z

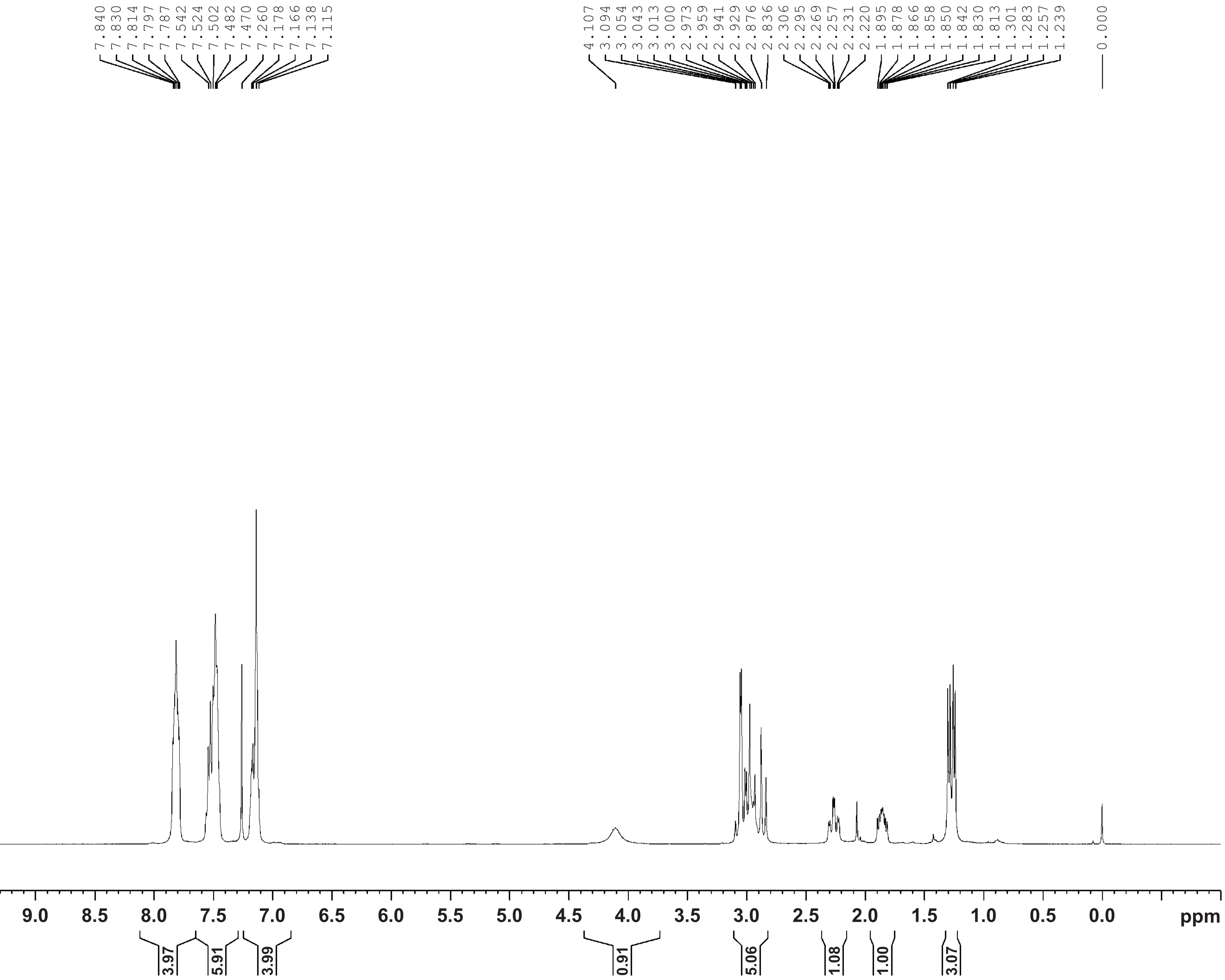

^{13}C NMR (100.6 MHz, $CDCl_3$)

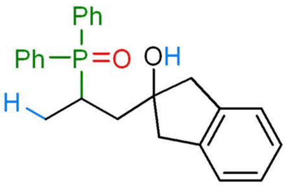

2z

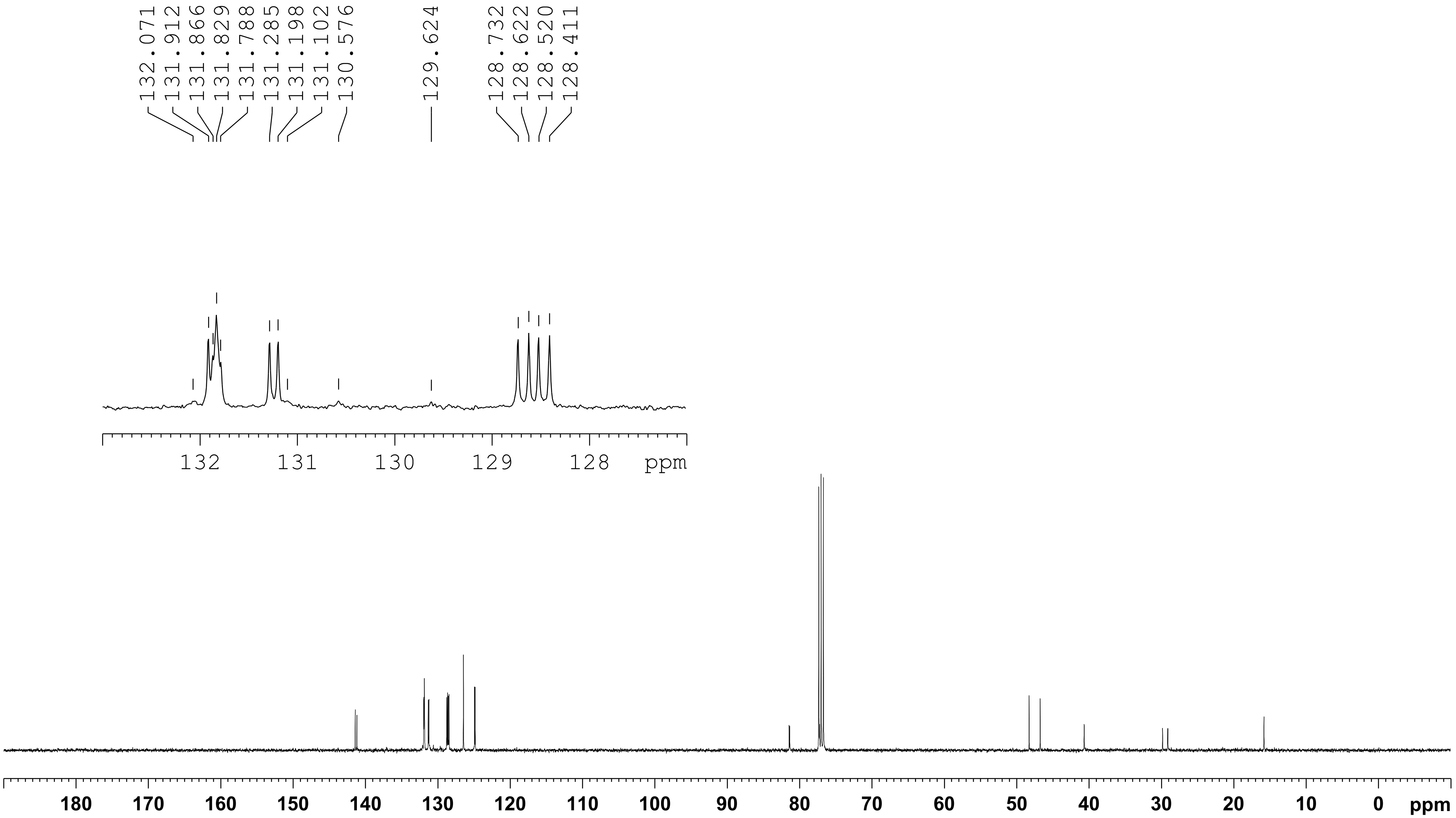

^{31}P NMR (162 MHz, CDCl$_3$)

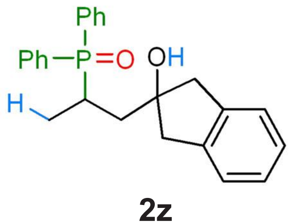

2z

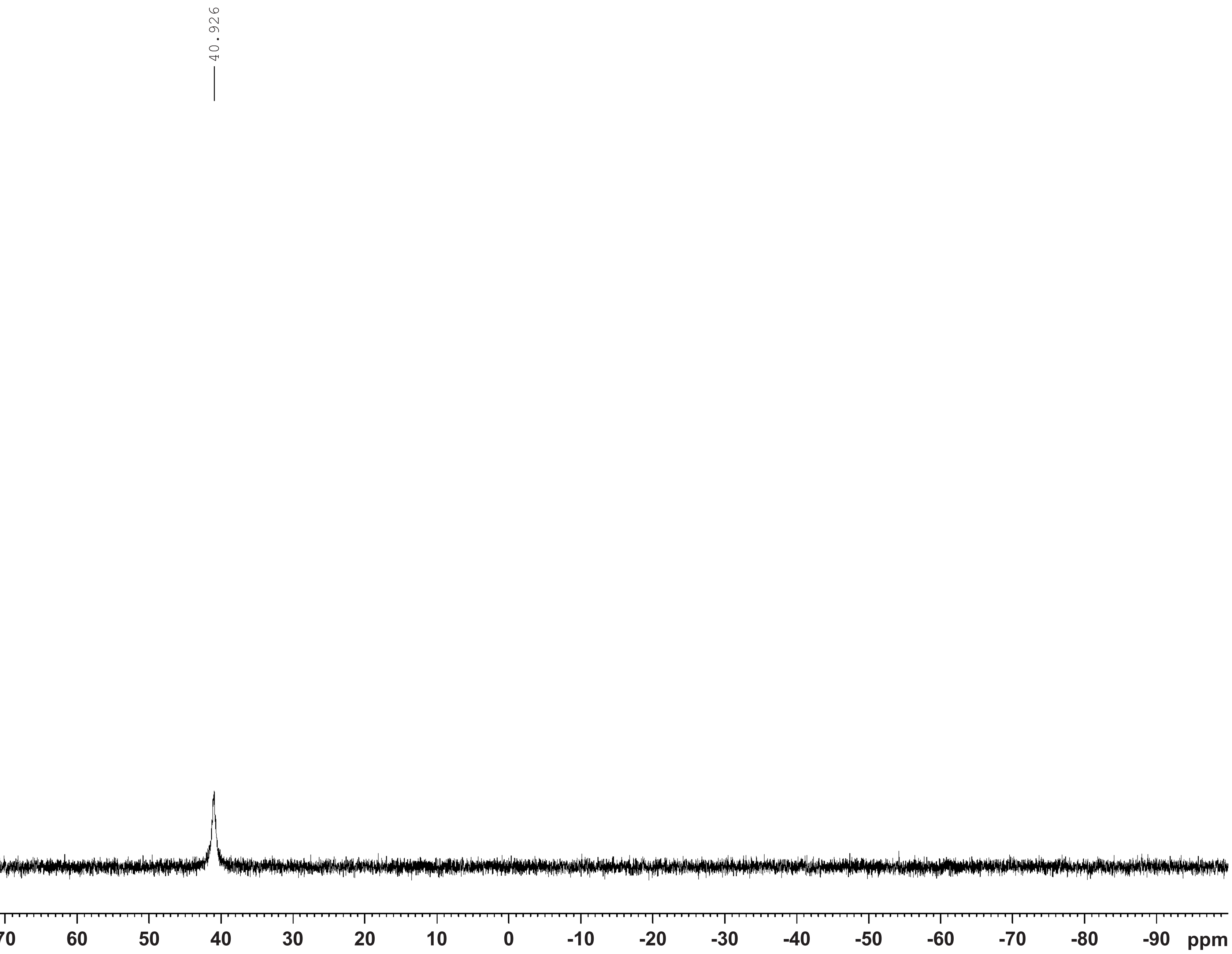

^{1}H NMR (400 MHz, $CDCl_3$)

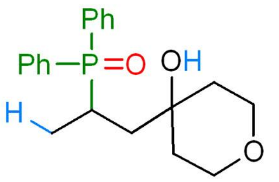

2aa

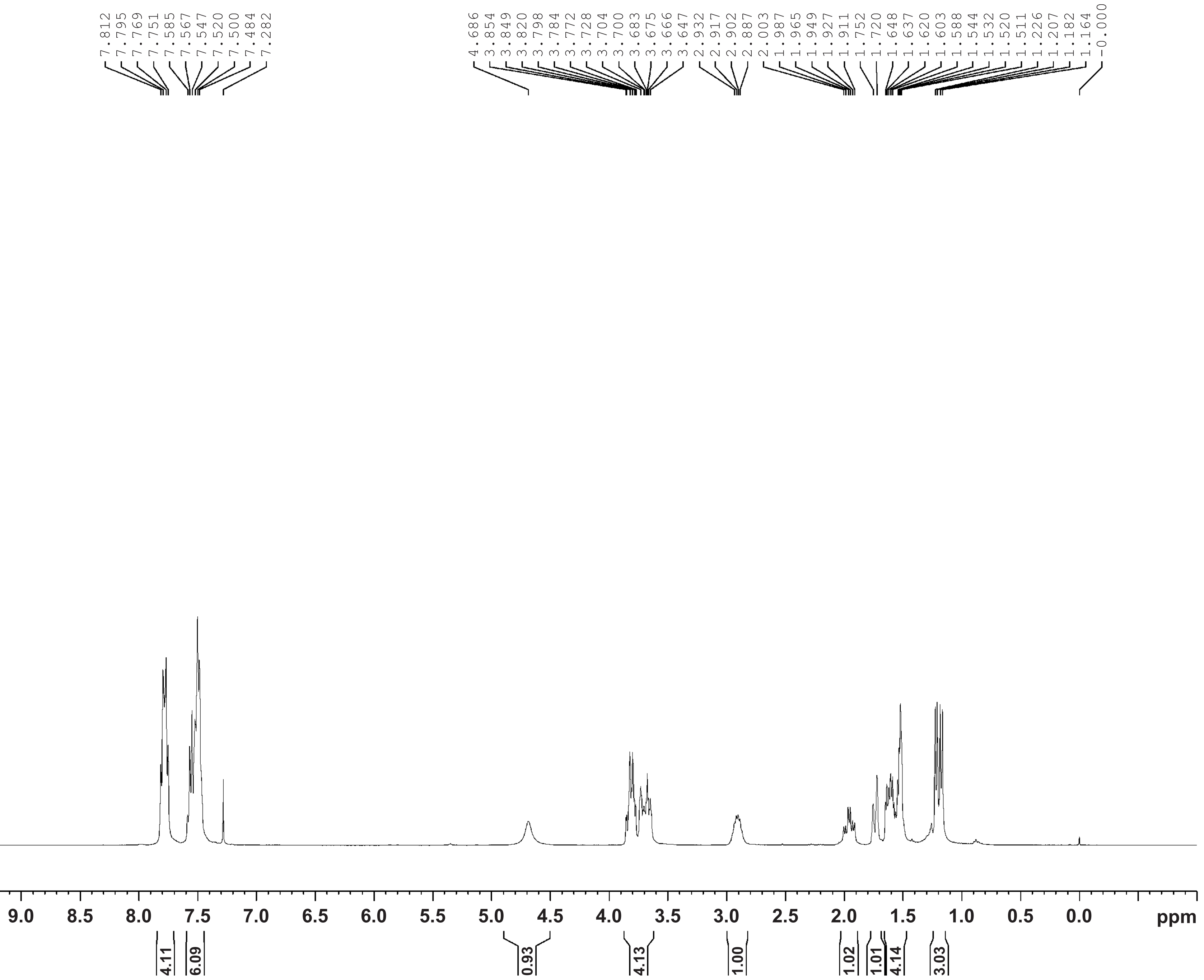

^{13}C NMR (100.6 MHz, $CDCl_3$)

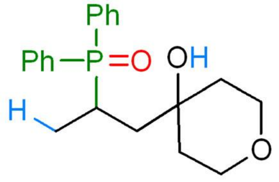

2aa

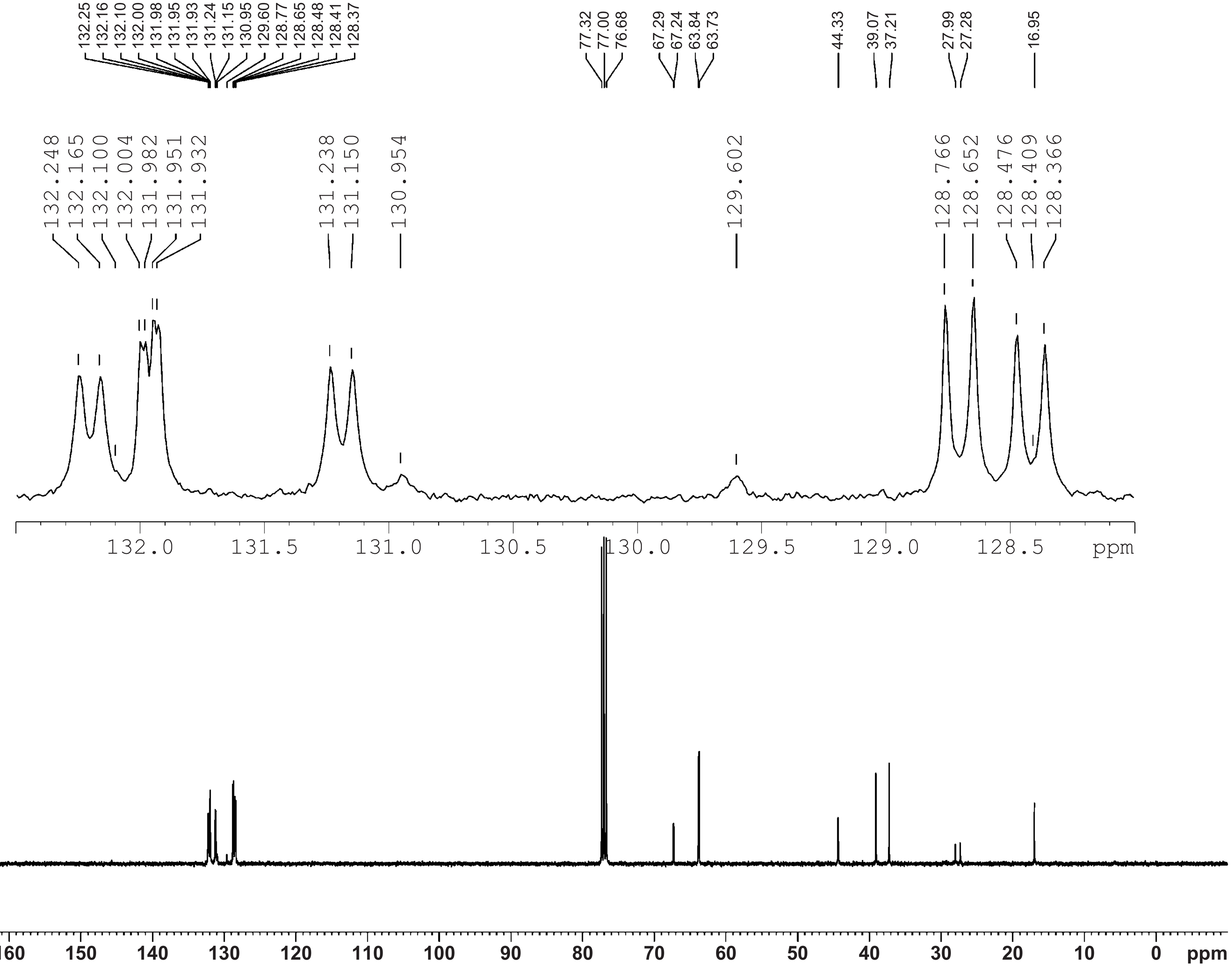

^{31}P NMR (162 MHz, $CDCl_3$)

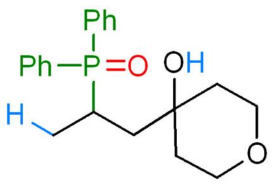

2aa

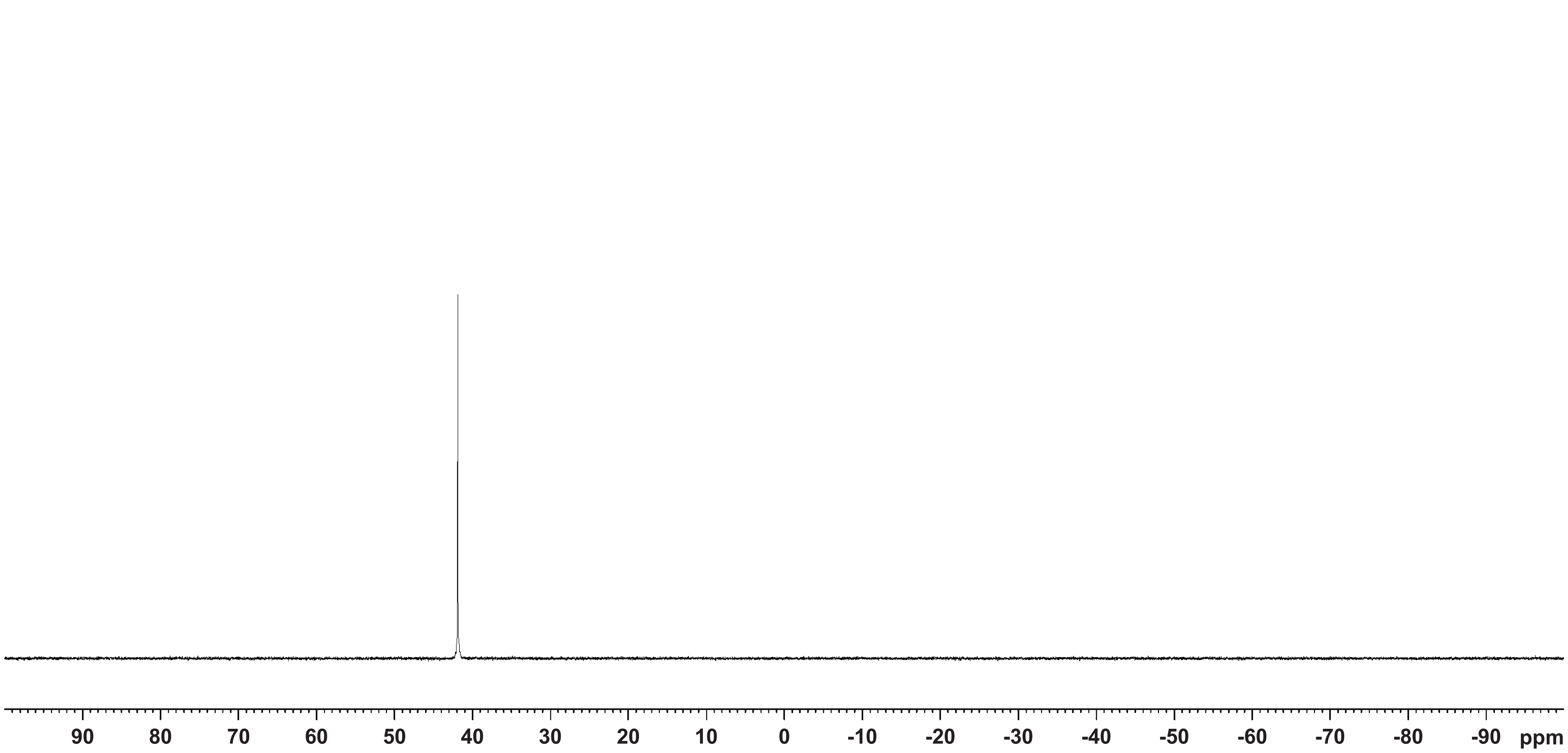

^{1}H NMR (400 MHz, $CDCl_3$)

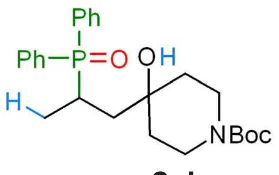

2ab

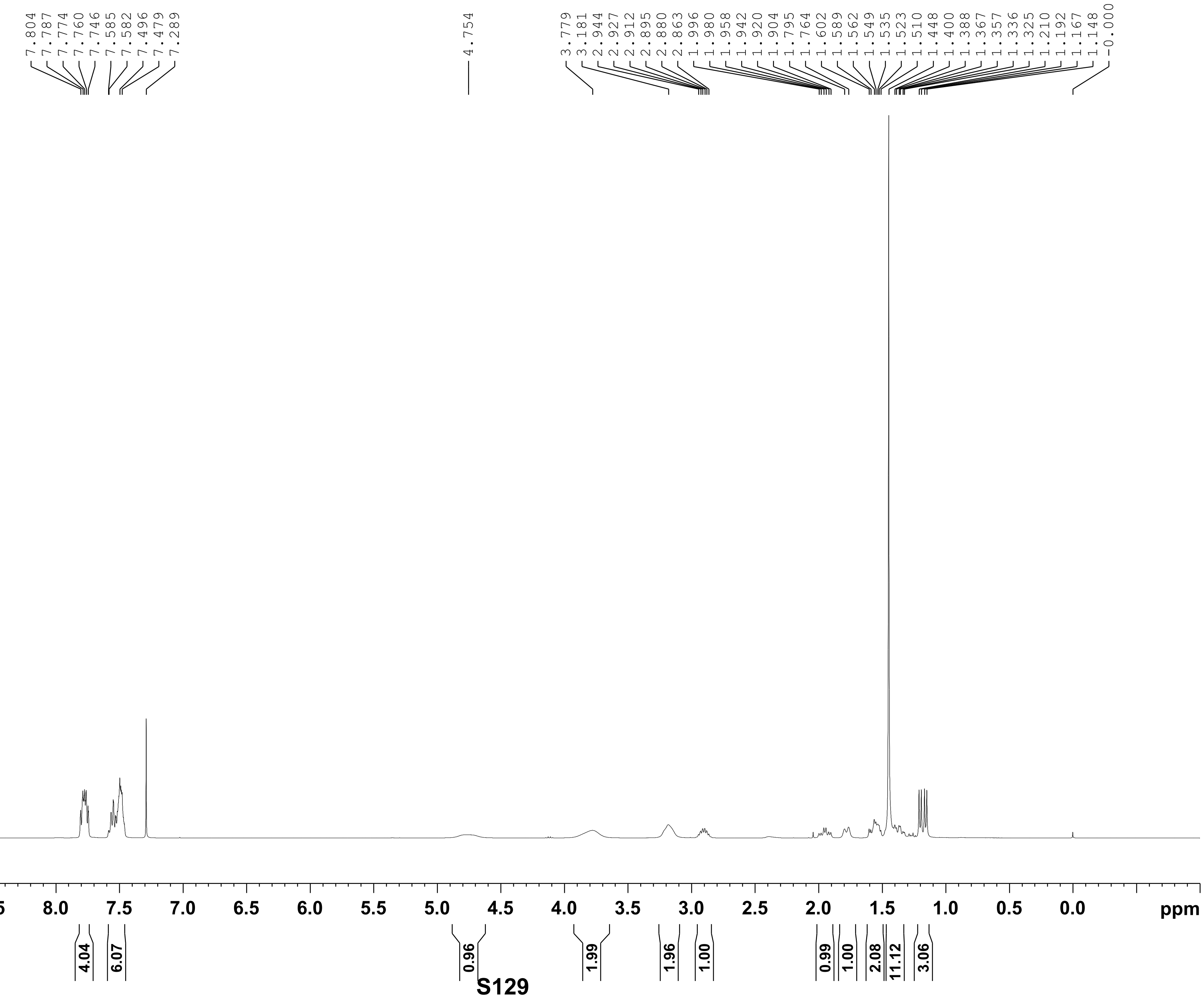

^{13}C NMR (100.6 MHz, $CDCl_3$)

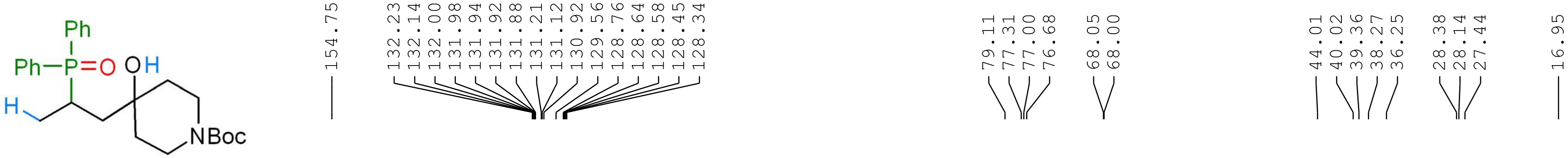

2ab

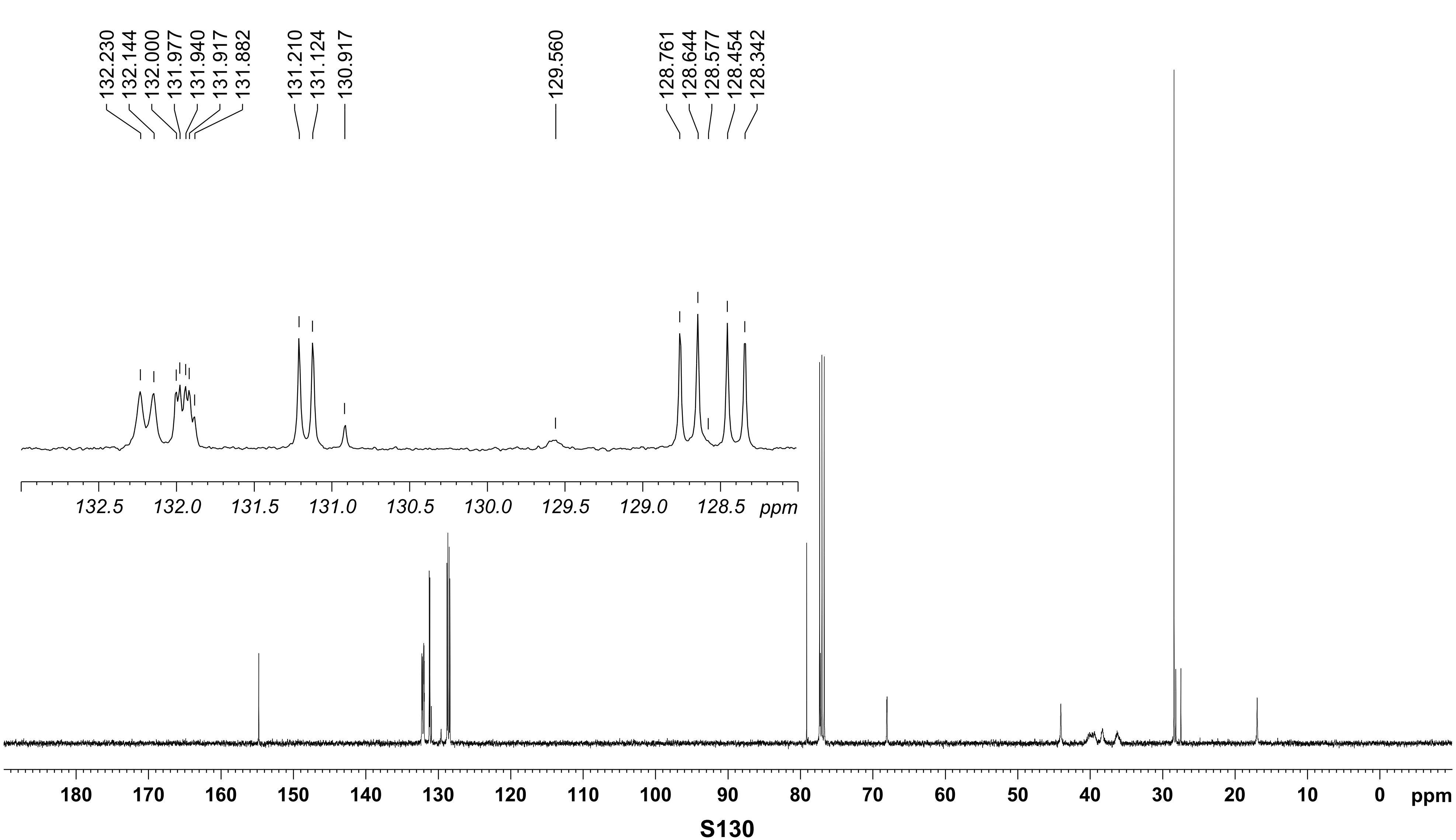

^{31}P NMR (162 MHz, CDCl$_3$)

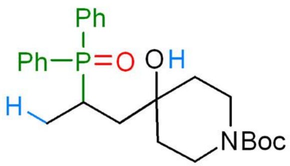

2ab

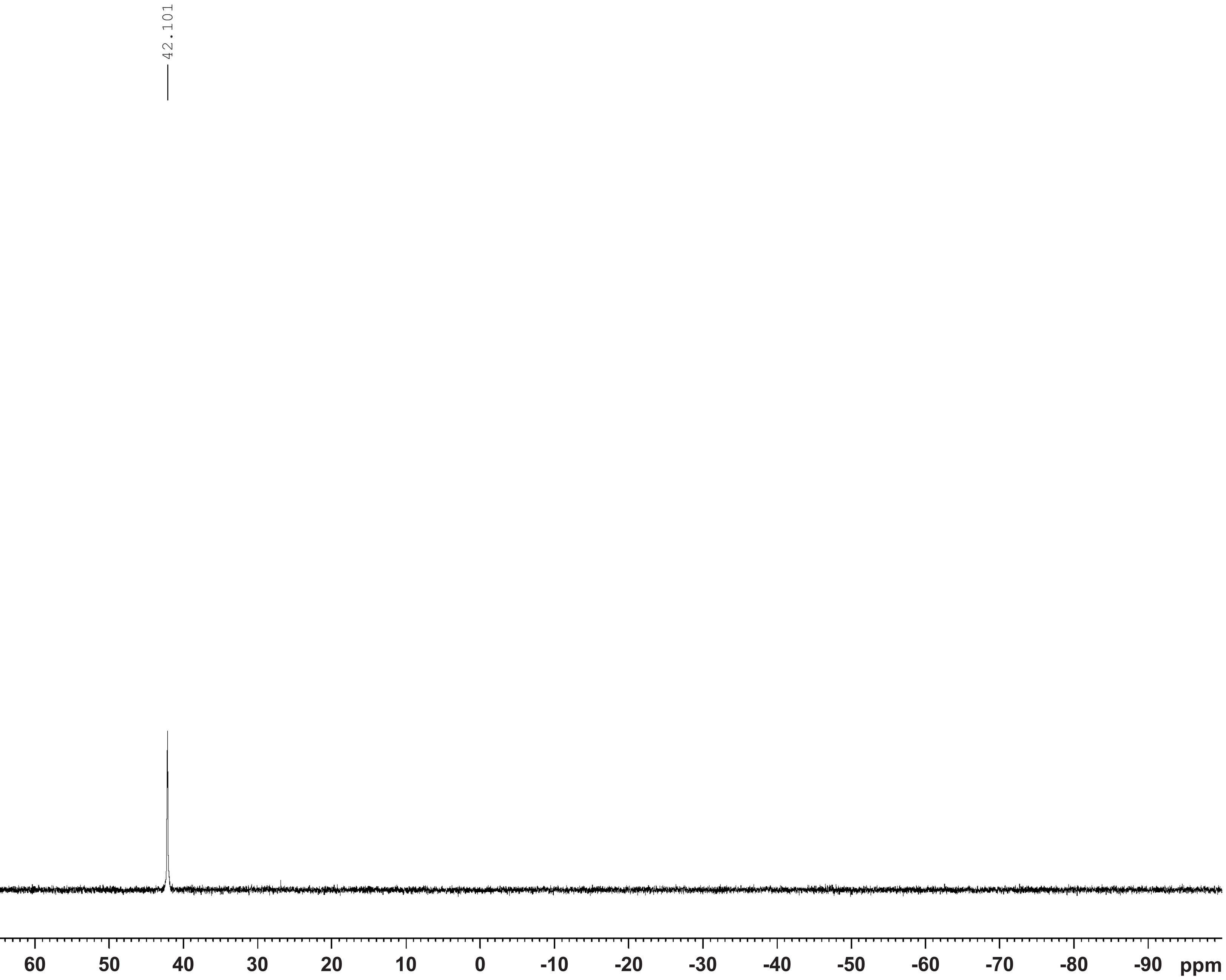

^{1}H NMR (400 MHz, $CDCl_3$)

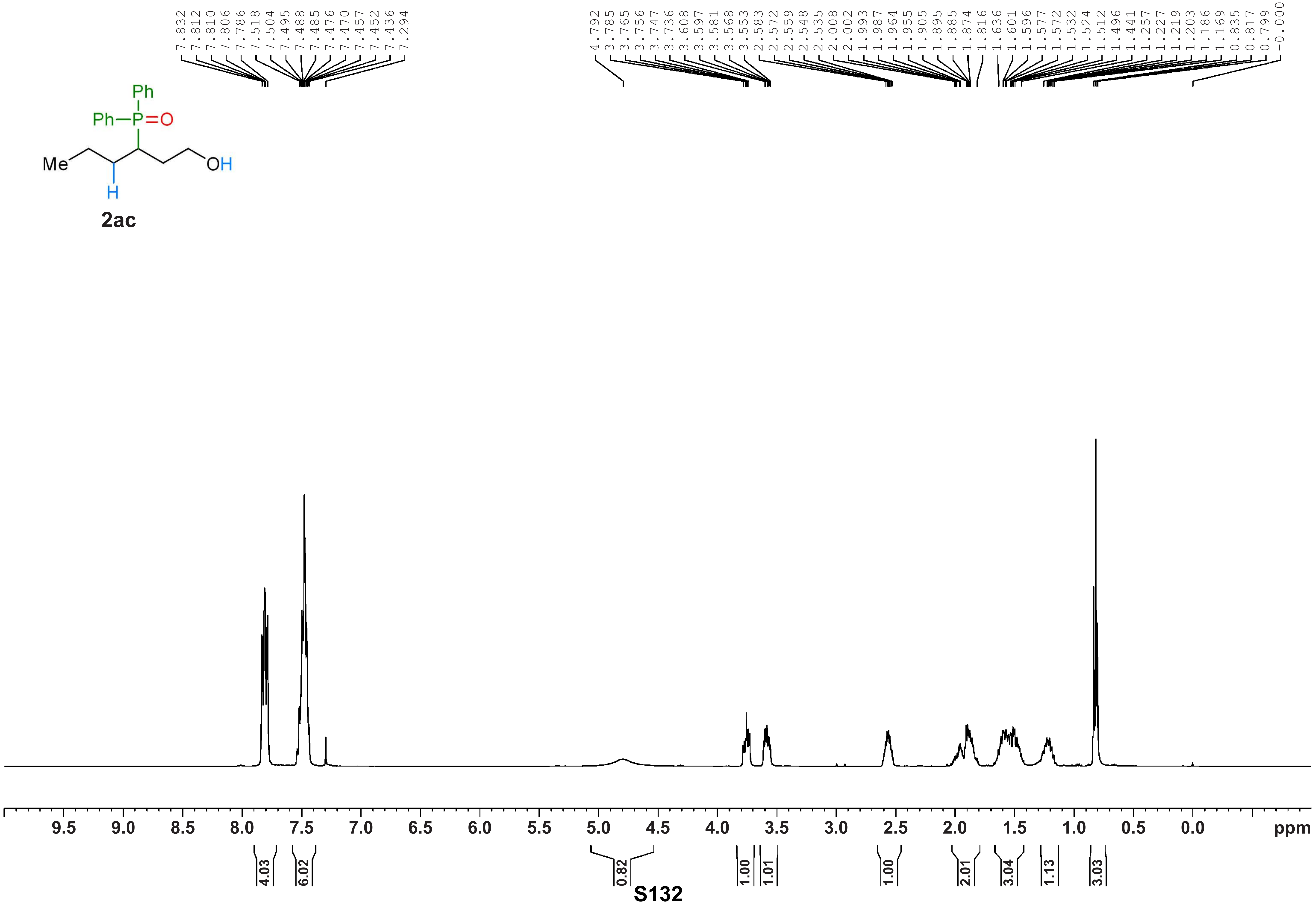

^{13}C NMR (100.6 MHz, $CDCl_3$)

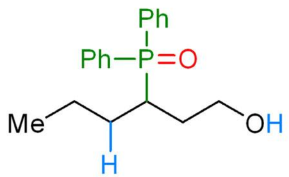

2ac

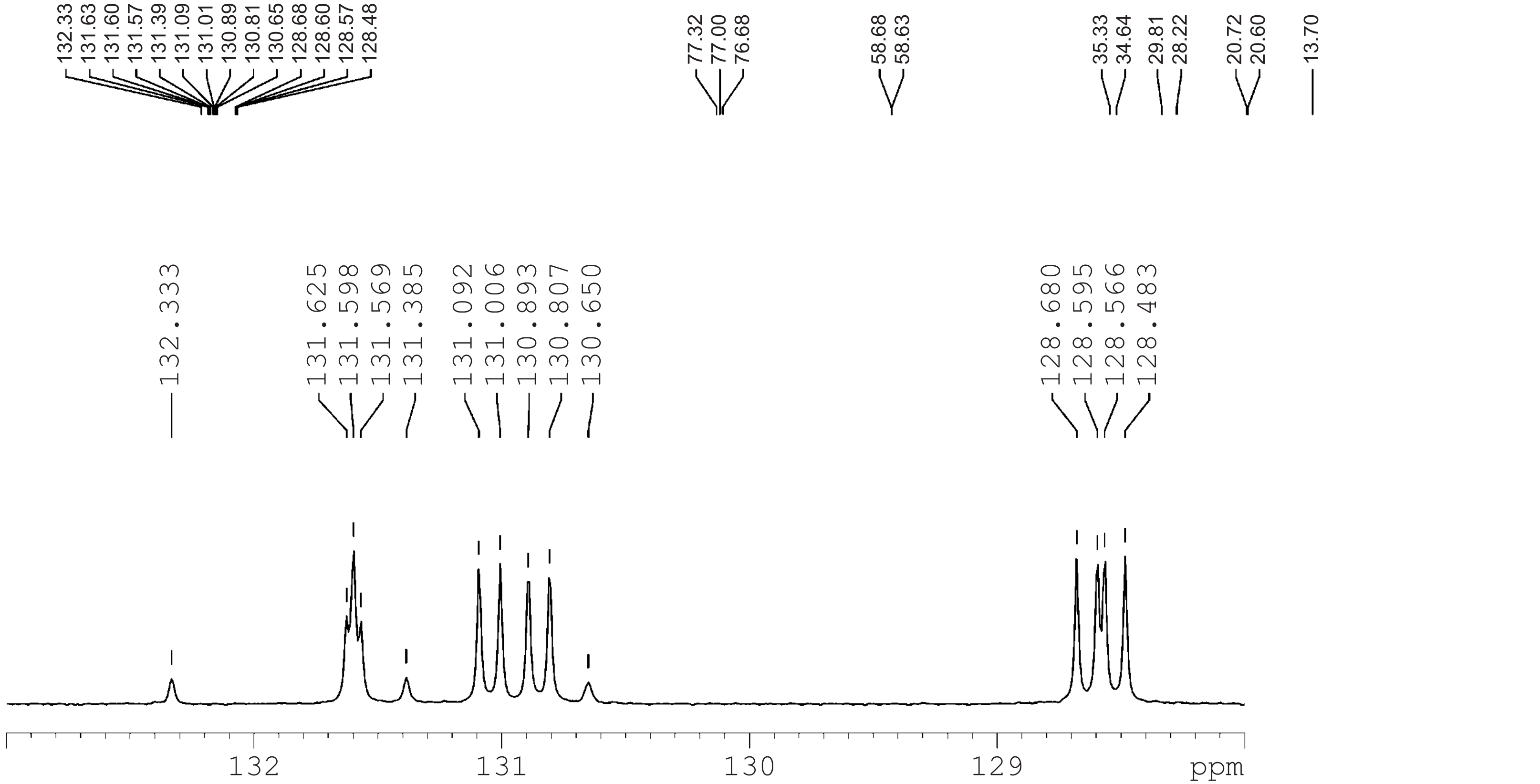

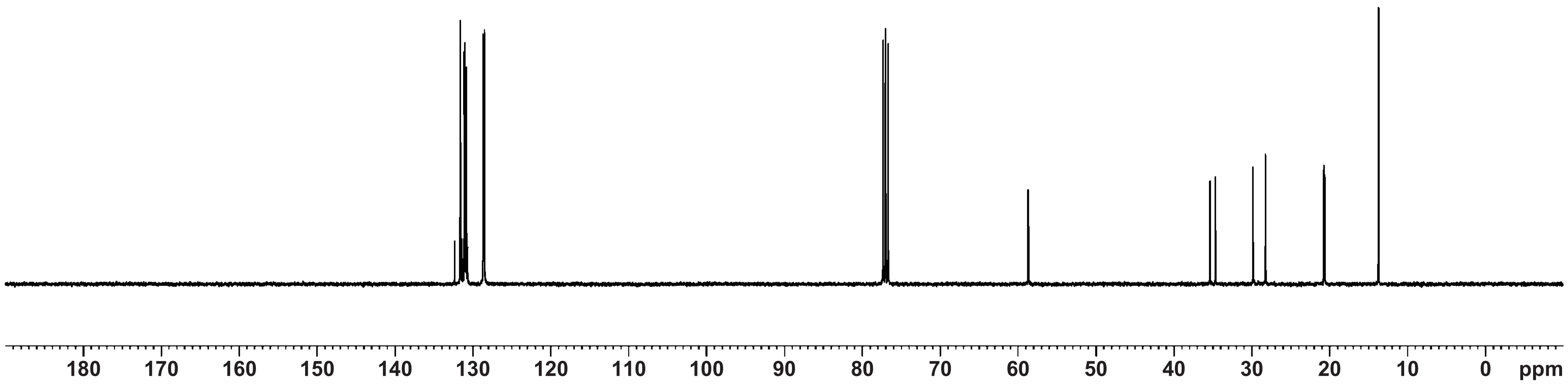

^{31}P NMR (162 MHz, $CDCl_3$)

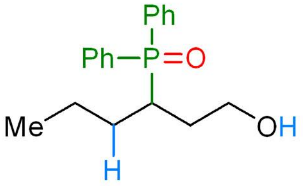

2ac

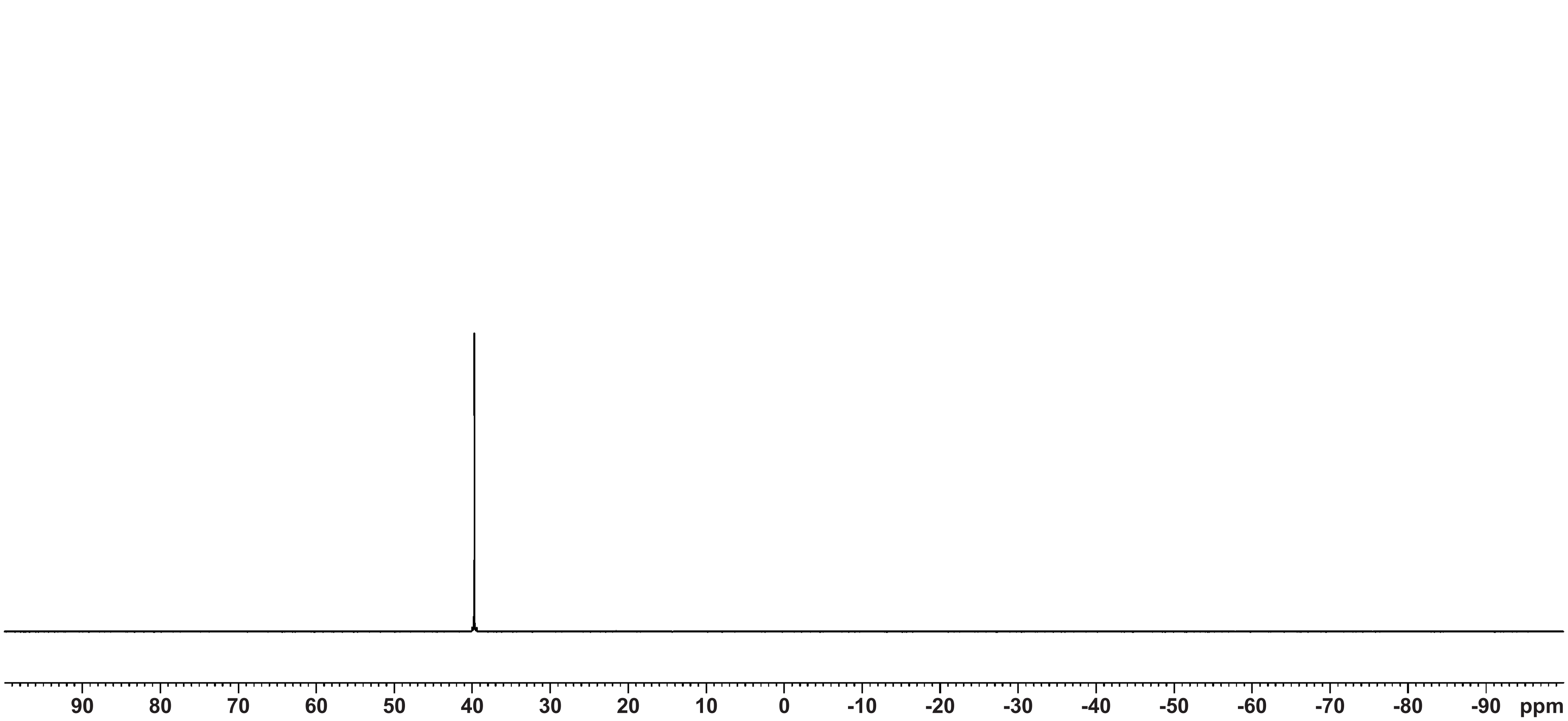

^{1}H NMR (400 MHz, $CDCl_3$)

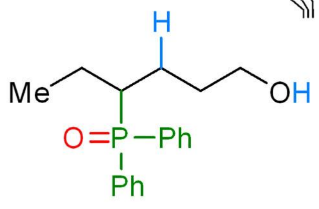

2ac'

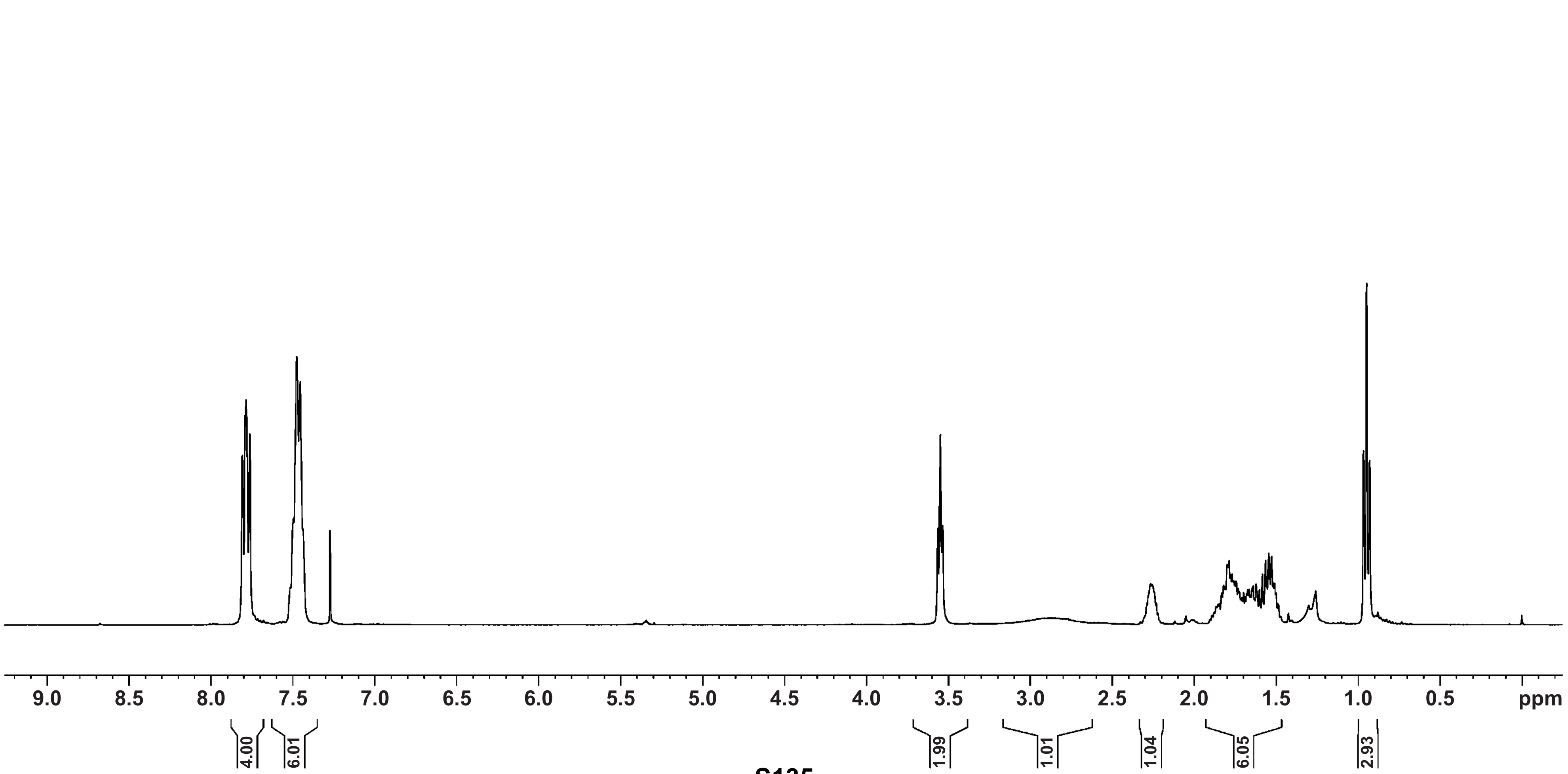

^{13}C NMR (100.6 MHz, $CDCl_3$)

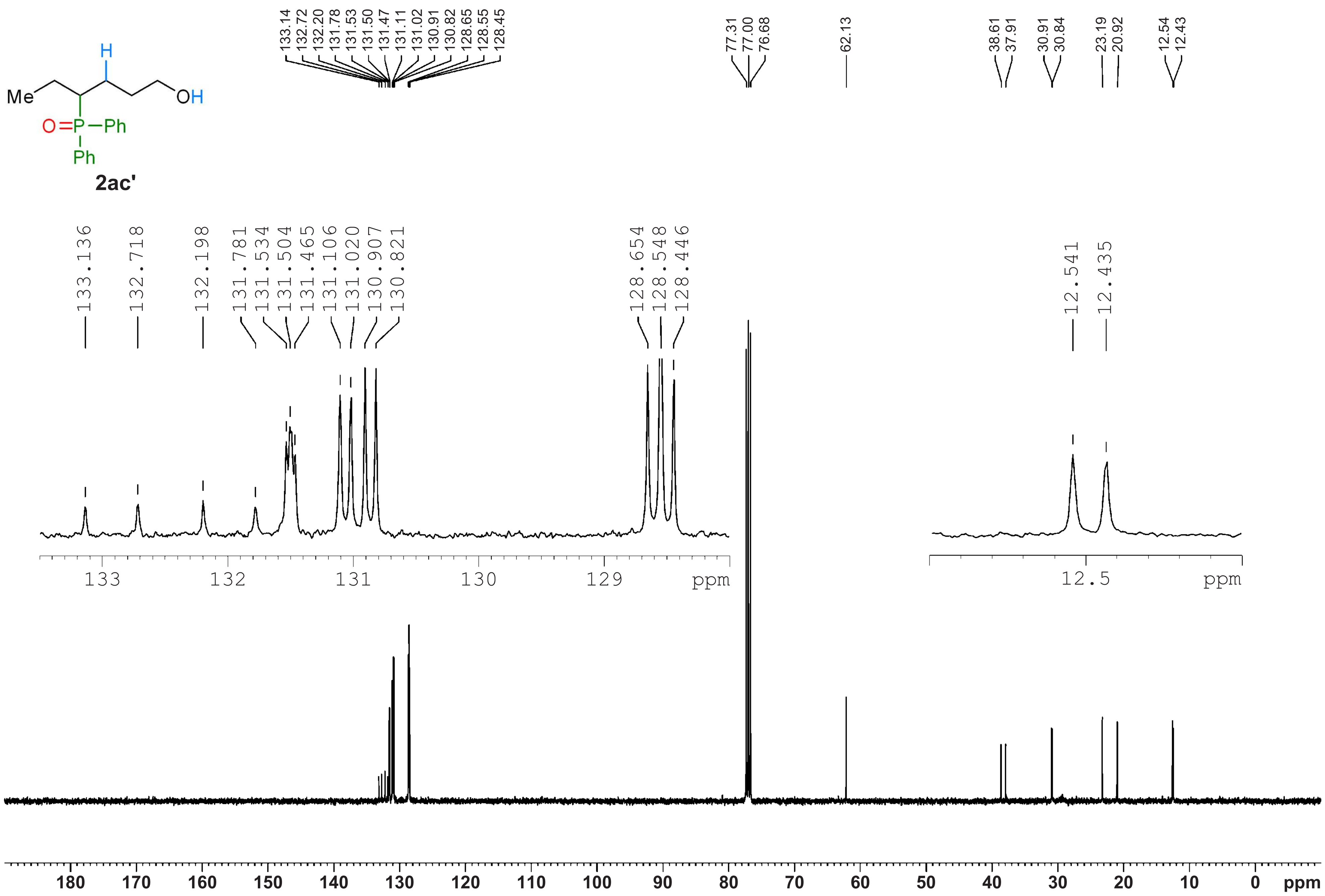

^{31}P NMR (162 MHz, $CDCl_3$)

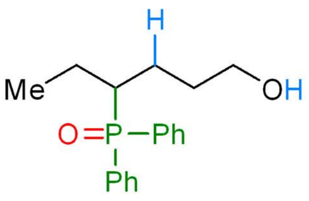

2ac'

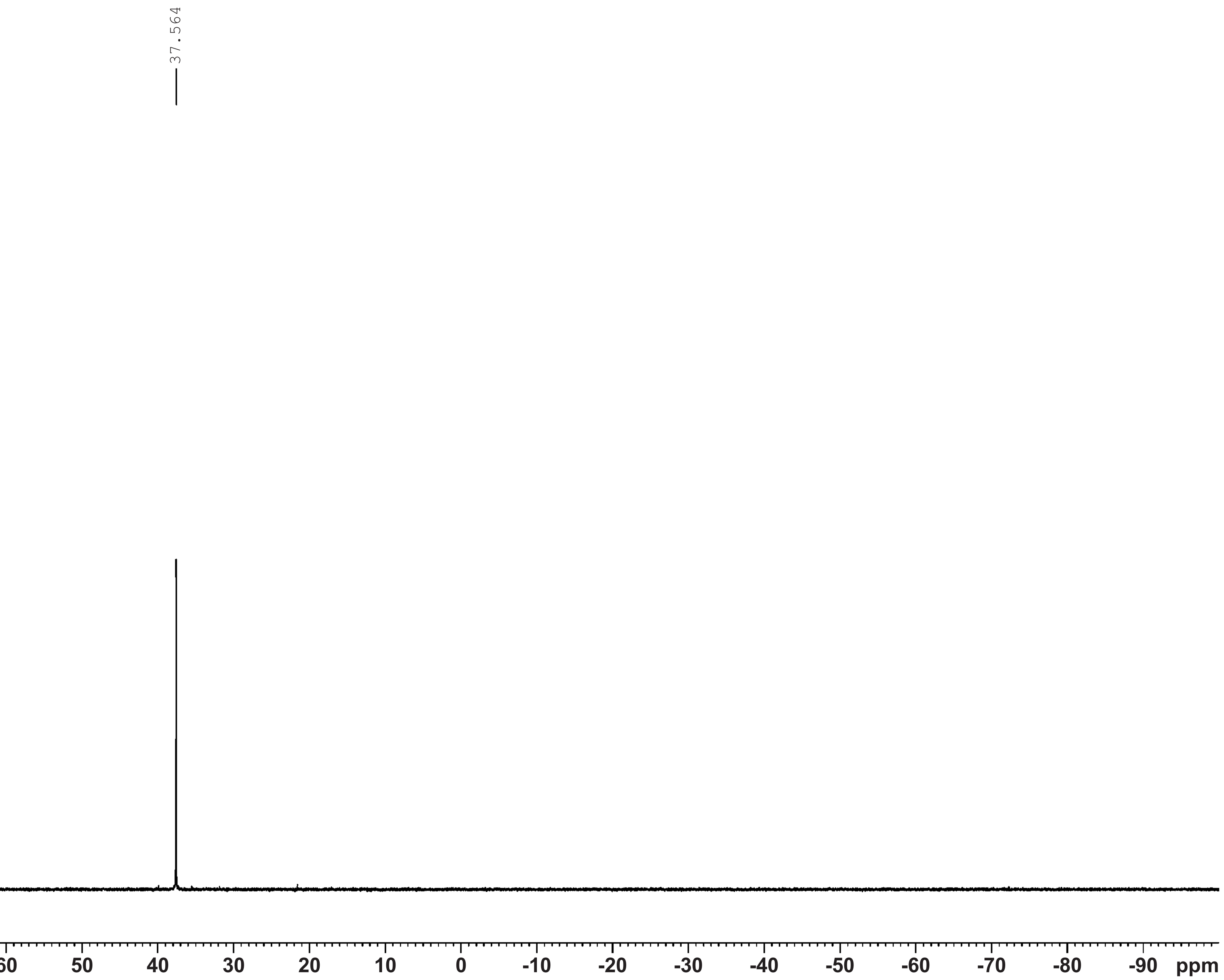

^{1}H NMR (400 MHz, $CDCl_3$)

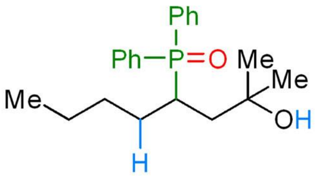

2ad

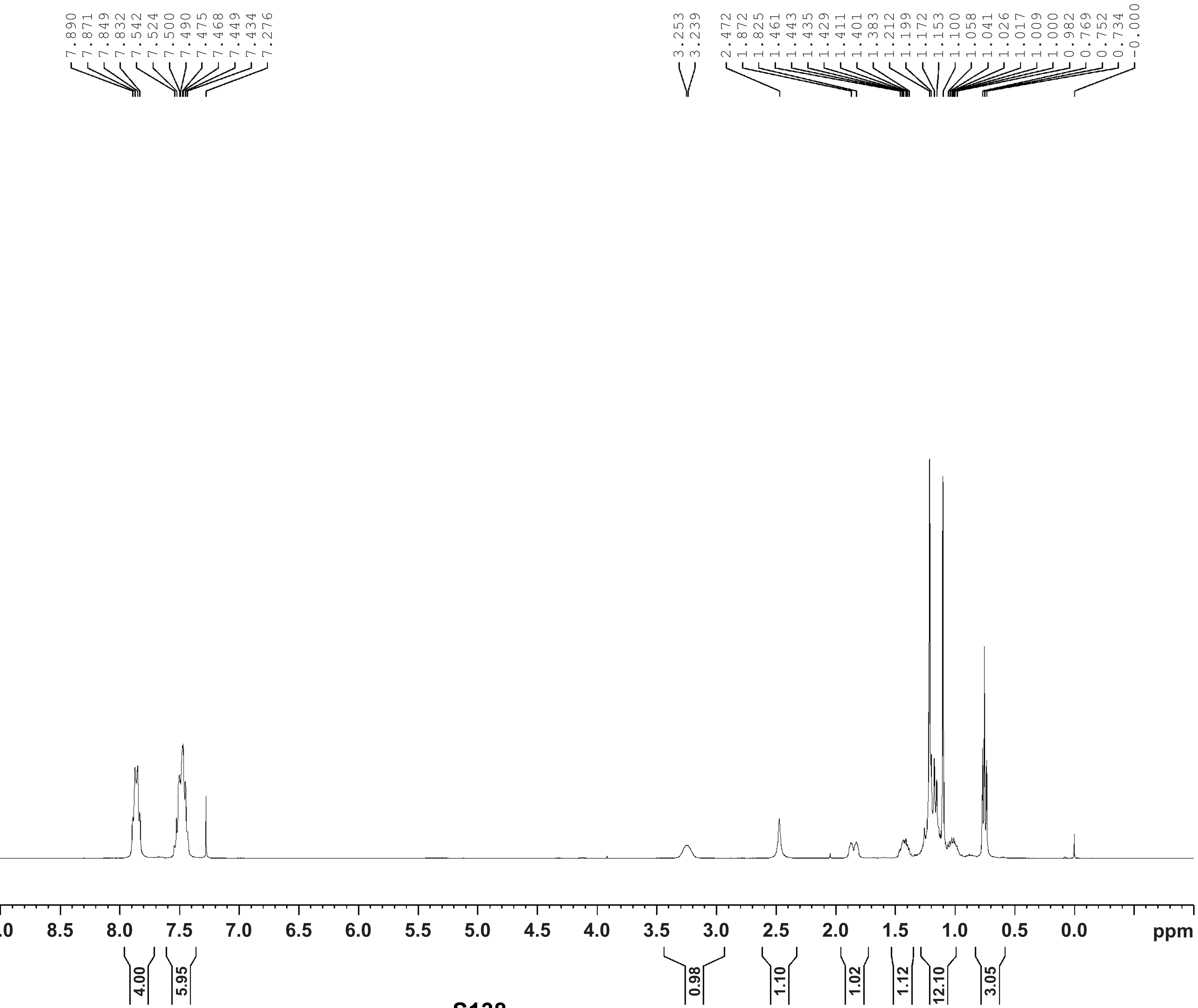

^{13}C NMR (100.6 MHz, $CDCl_3$)

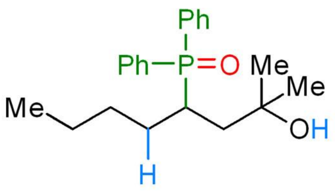

2ad

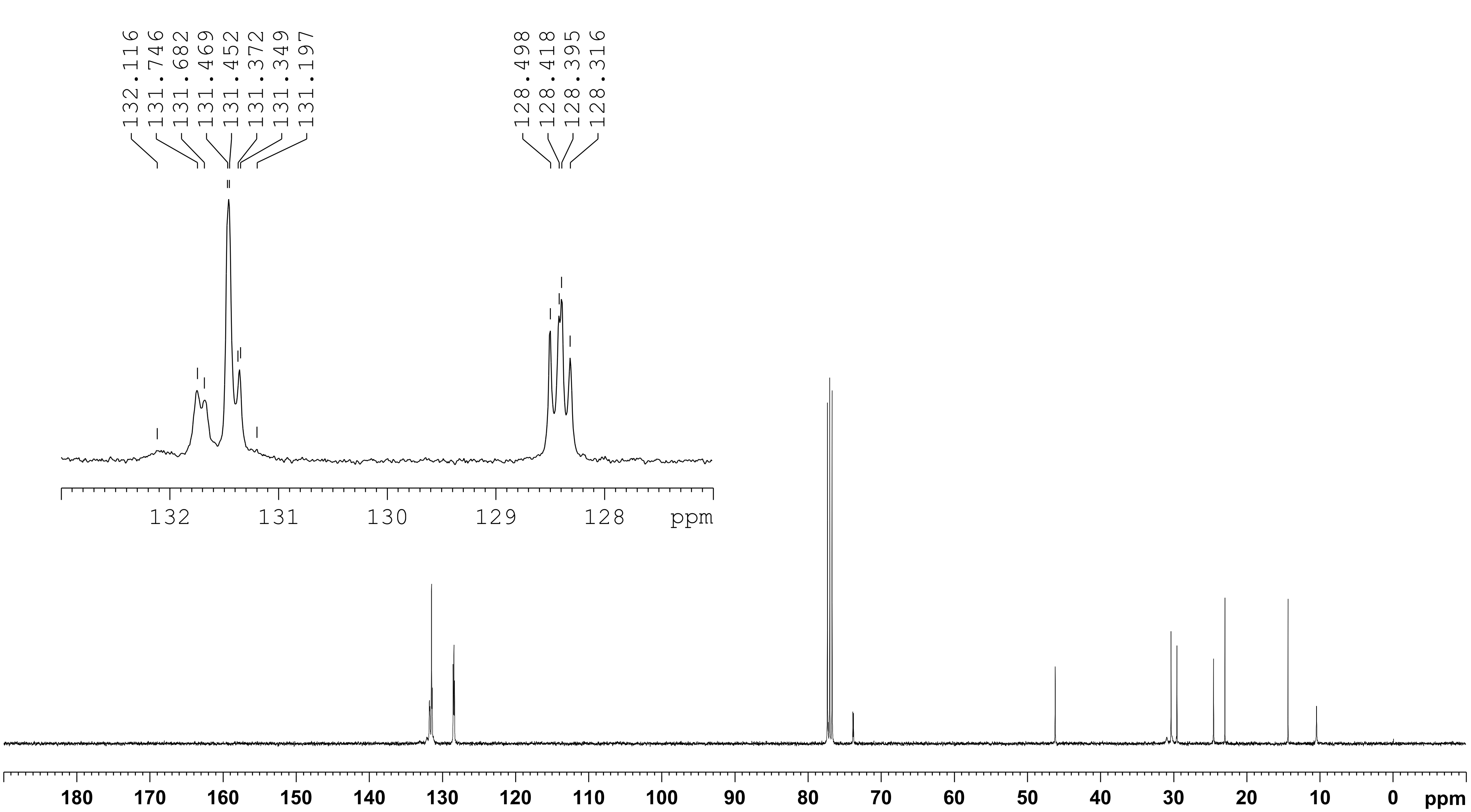

^{31}P NMR (162 MHz, $CDCl_3$)

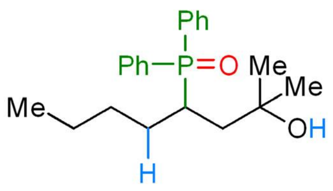

2ad

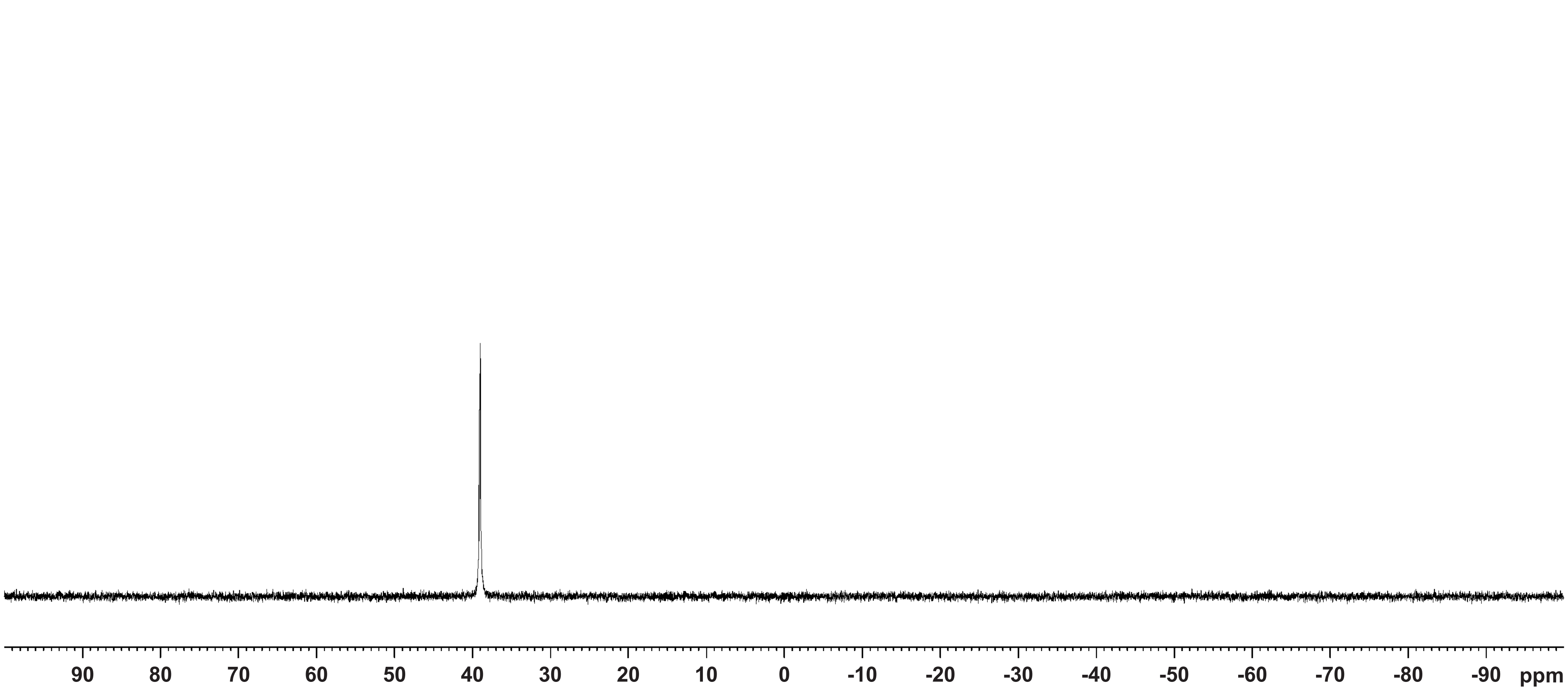

^{1}H NMR (400 MHz, $CDCl_3$)

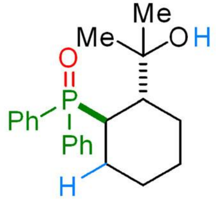

2ae

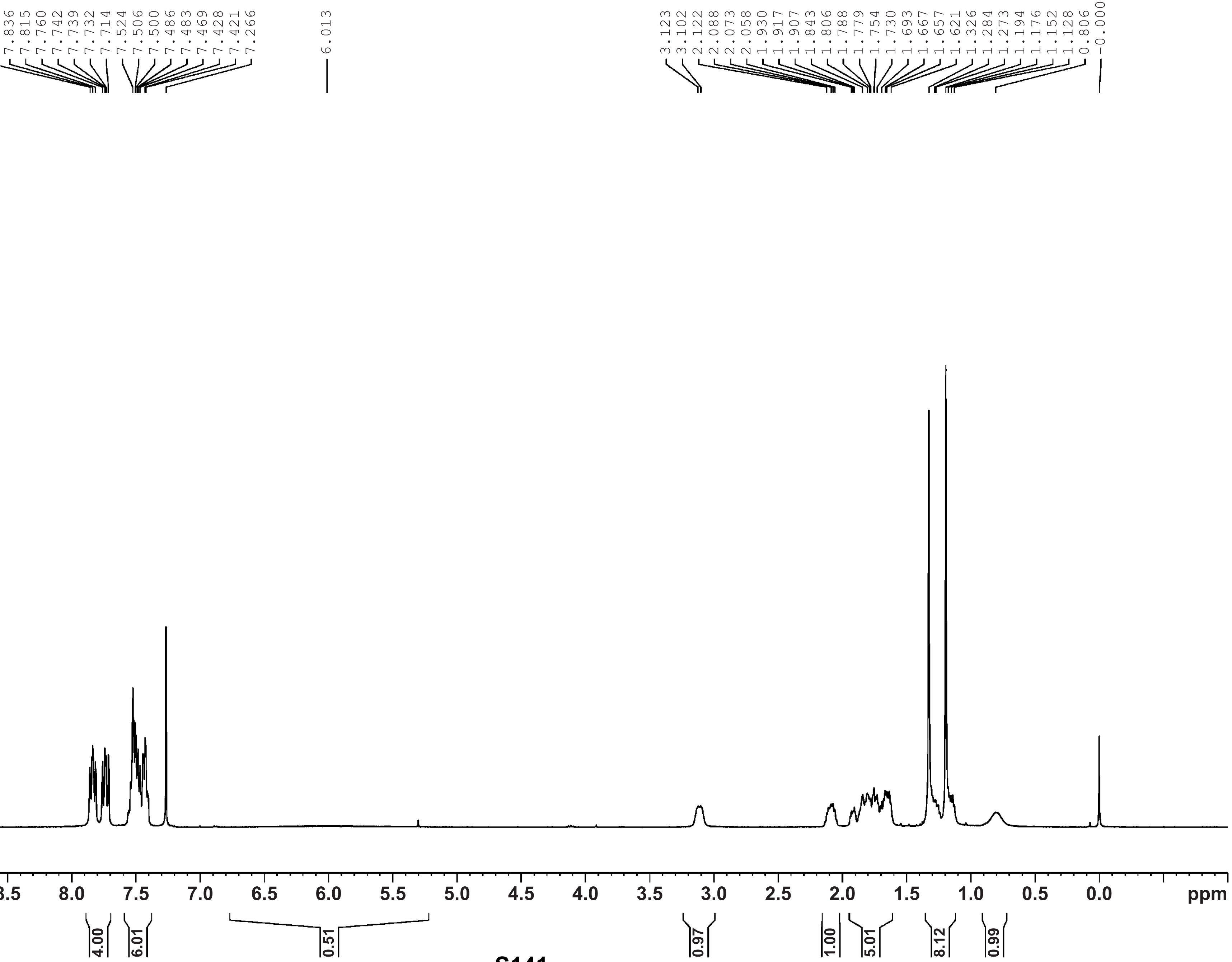

^{13}C NMR (100.6 MHz, $CDCl_3$)

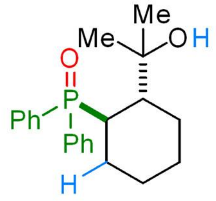

2ae

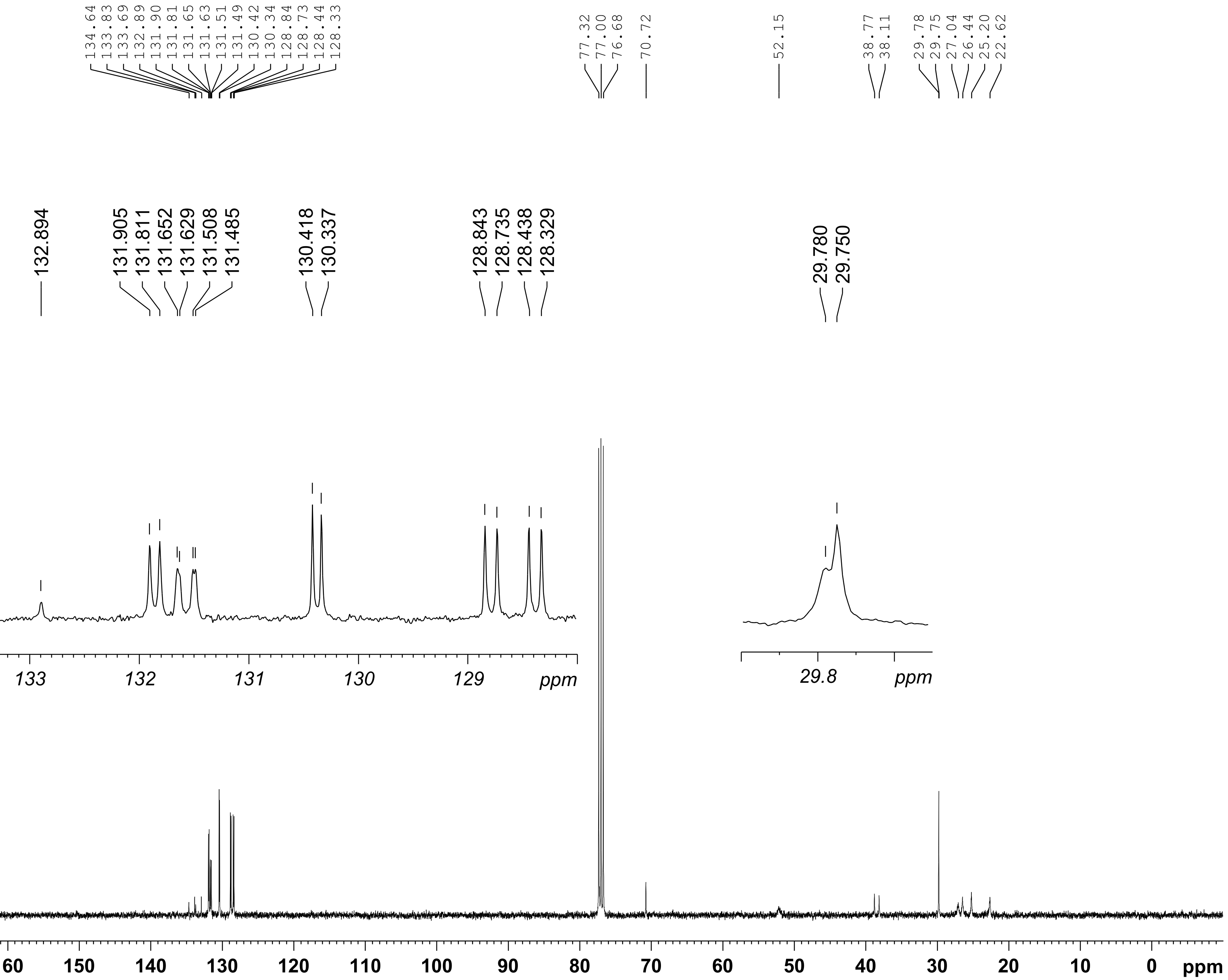

^{31}P NMR (162 MHz, CDCl$_3$)

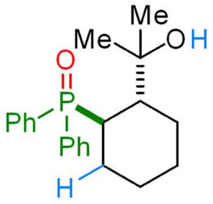

2ae

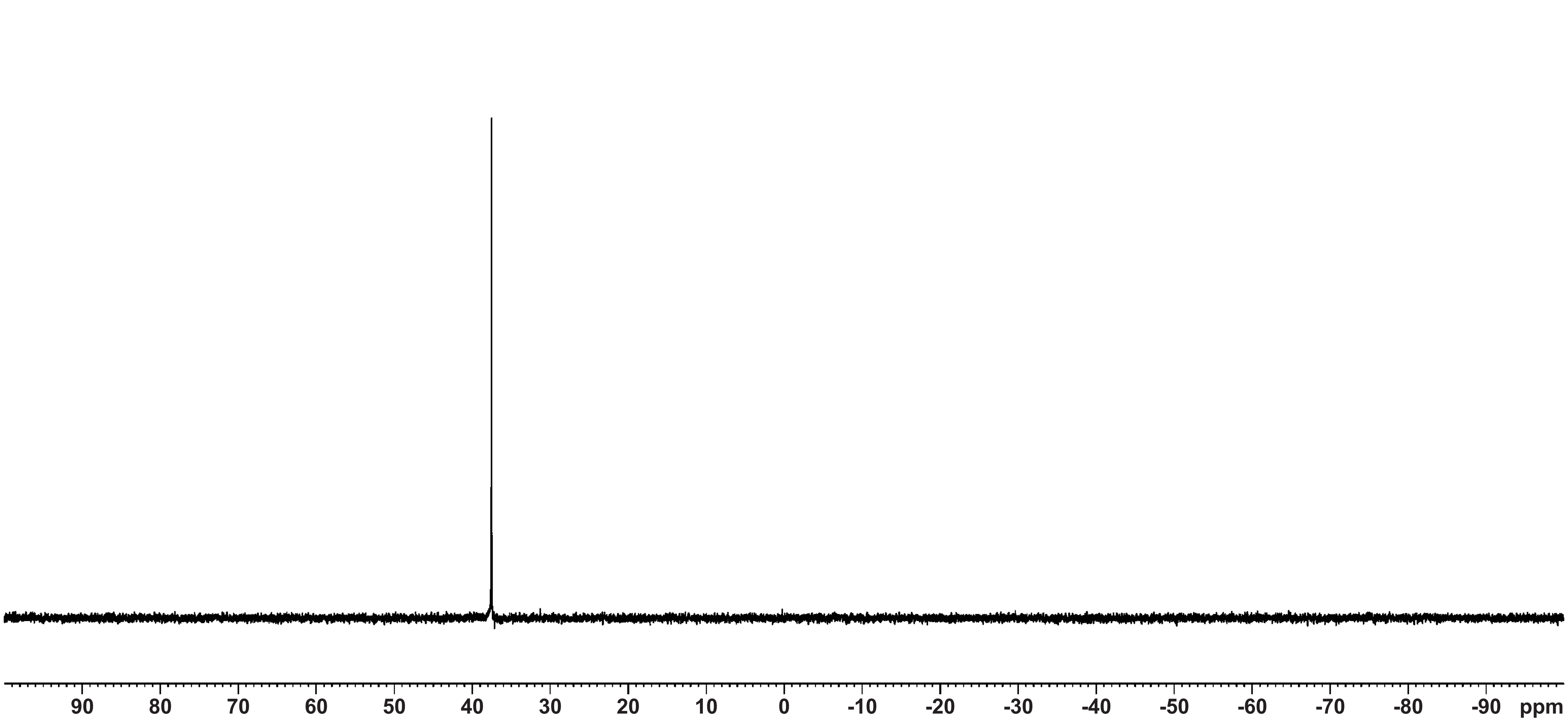

^{1}H NMR (400 MHz, $CDCl_3$)

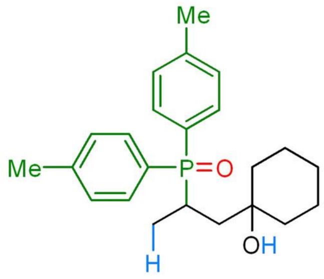

2af

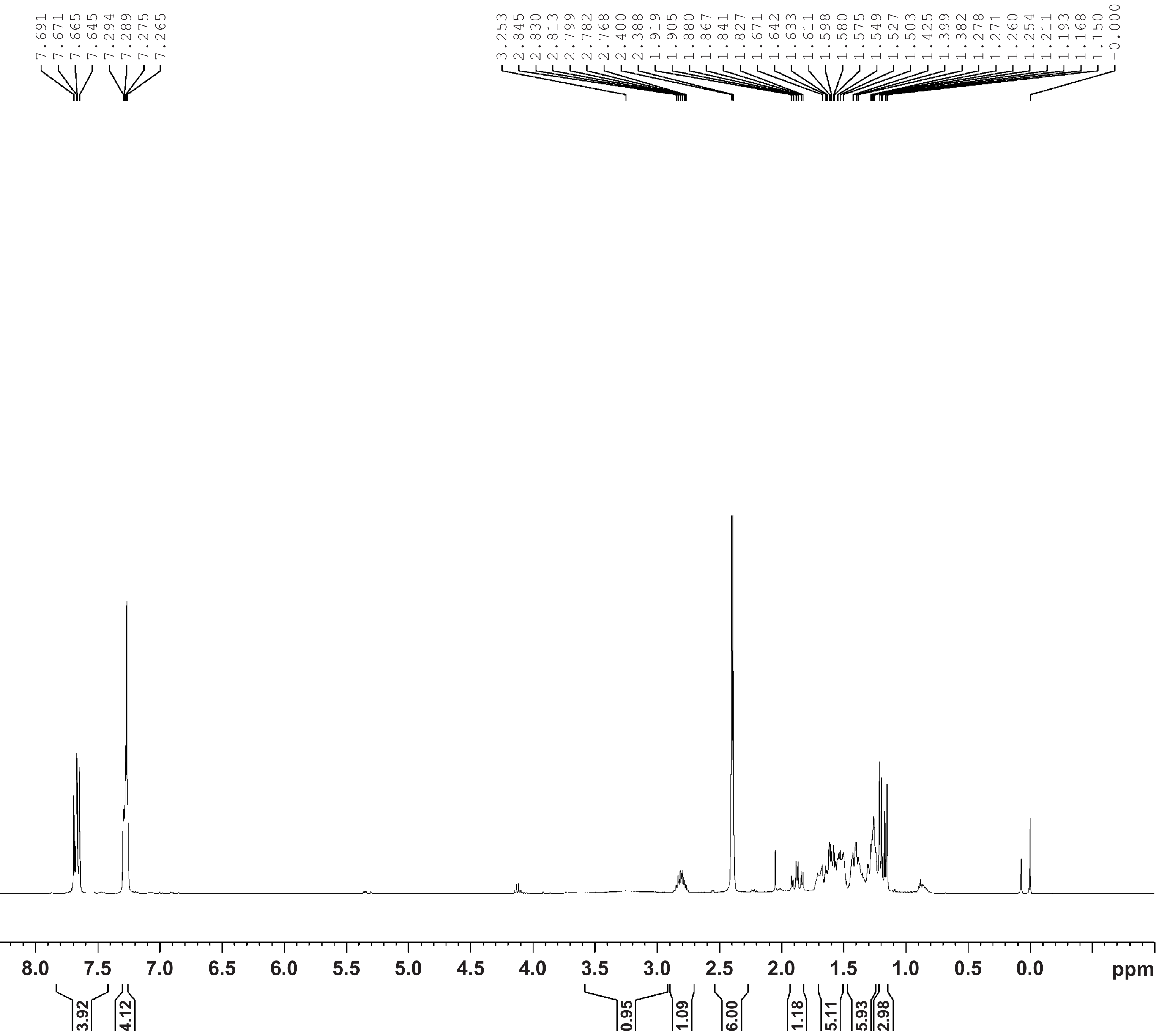

^{13}C NMR (100.6 MHz, $CDCl_3$)

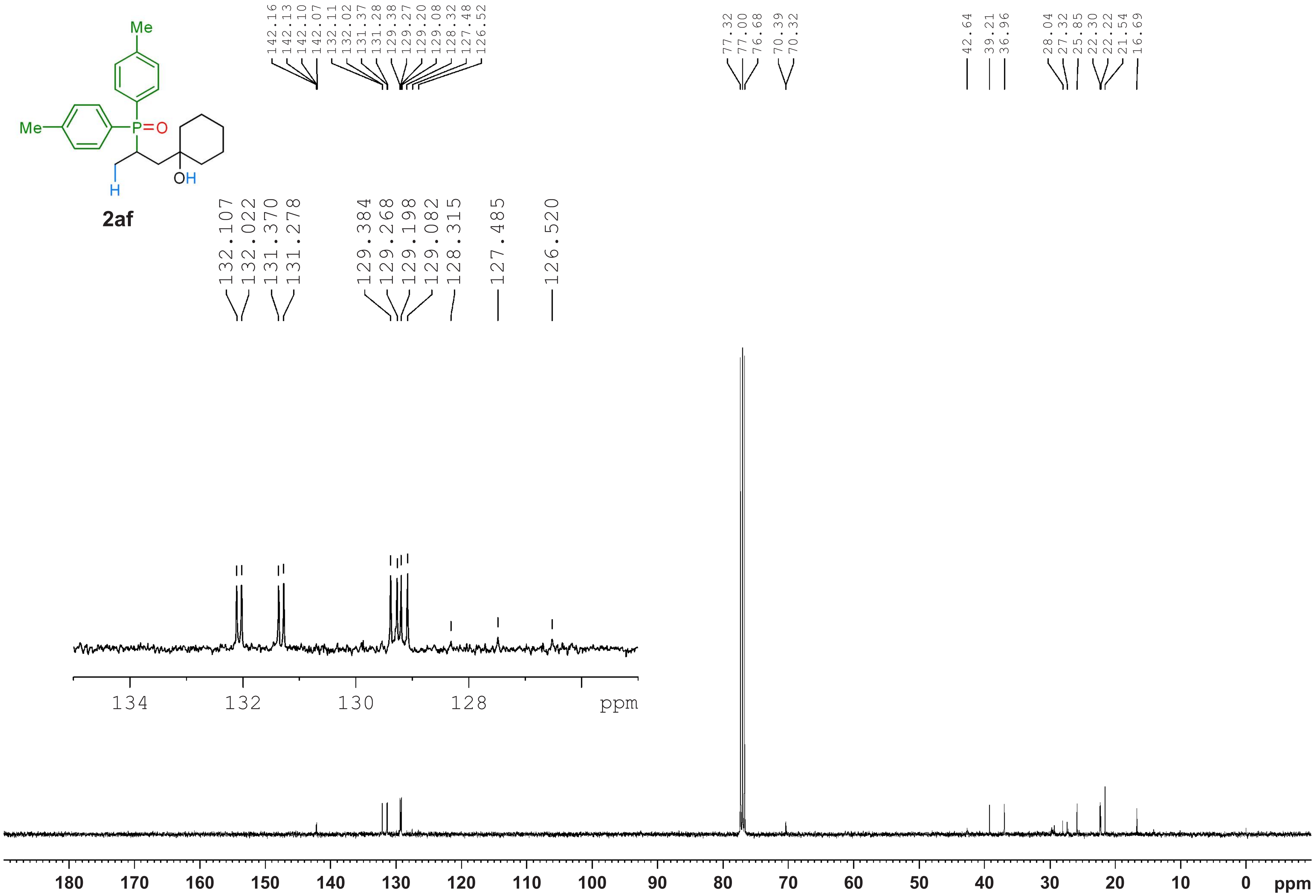

^{31}P NMR (162 MHz, $CDCl_3$)

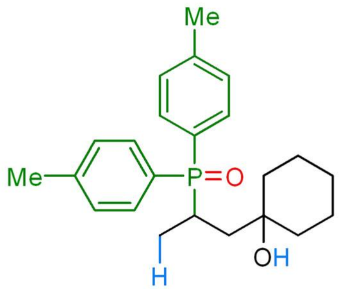

2af

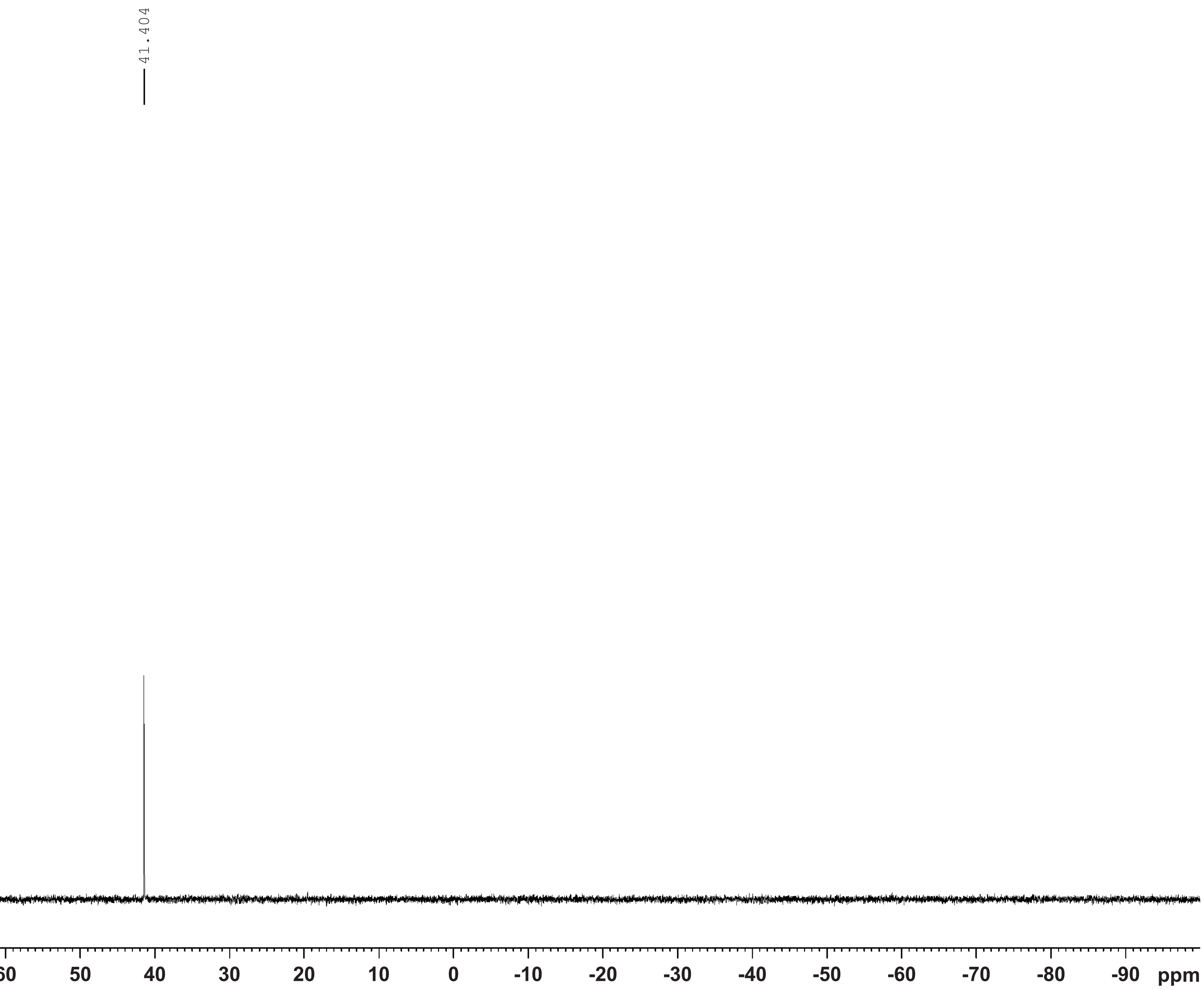

^{1}H NMR (400 MHz, $CDCl_3$)

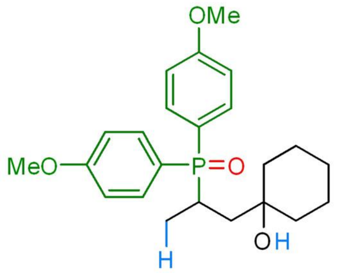

2ag

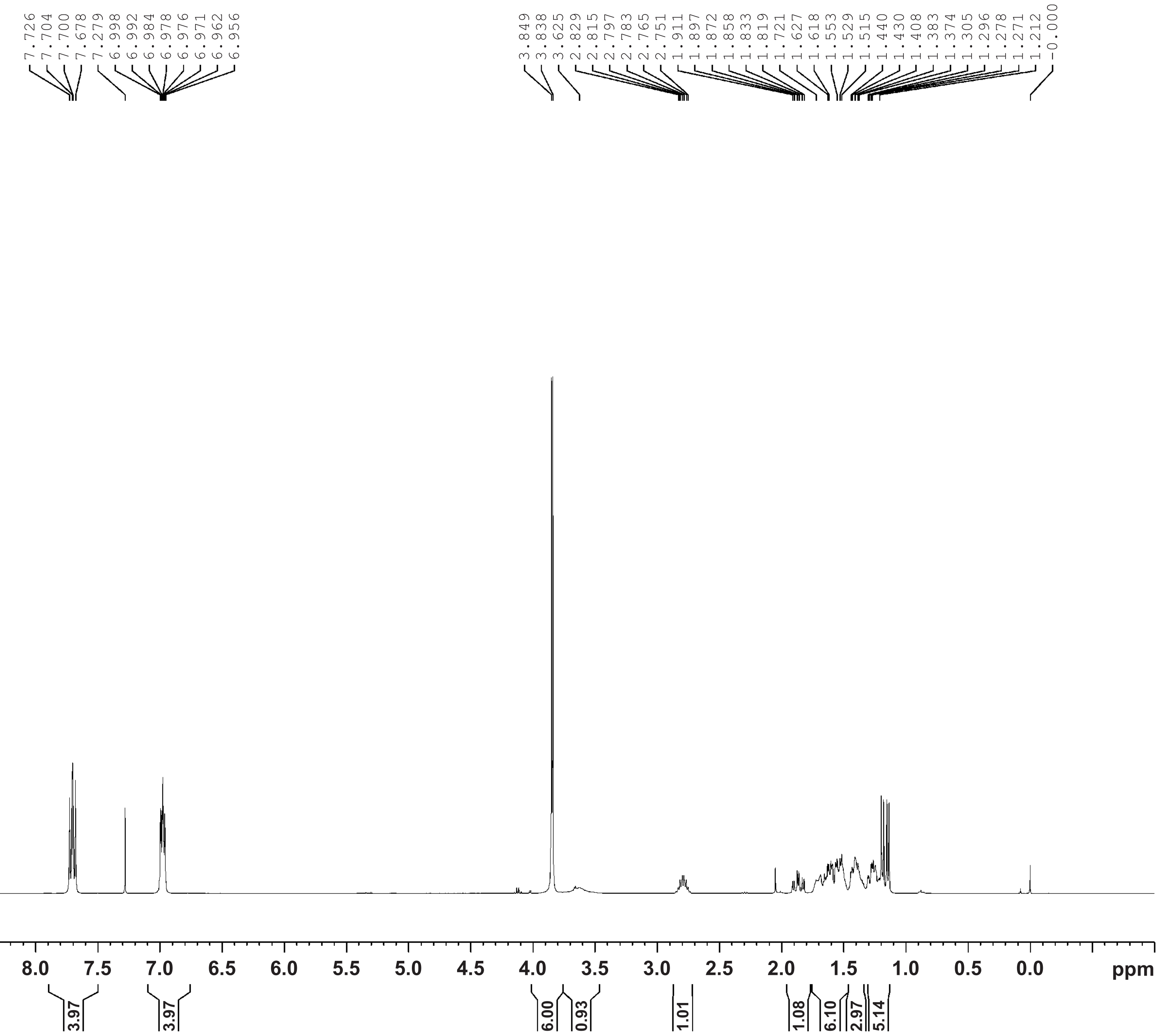

^{13}C NMR (100.6 MHz, $CDCl_3$)

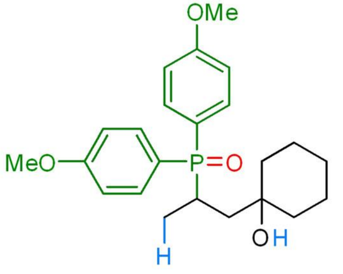

2ag

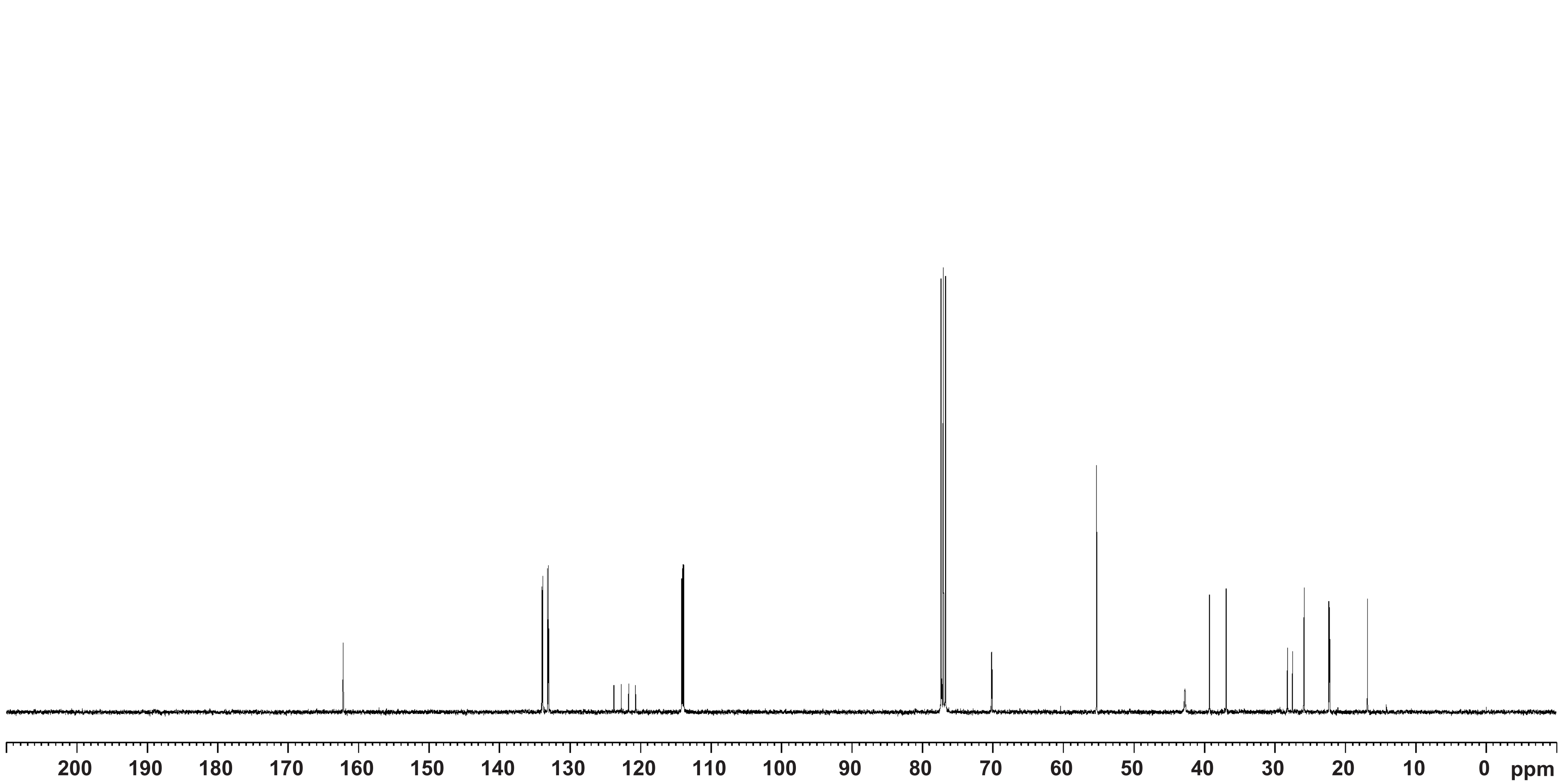

^{31}P NMR (162 MHz, $CDCl_3$)

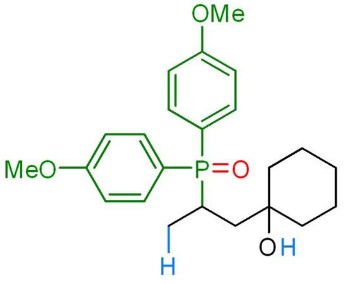

2ag

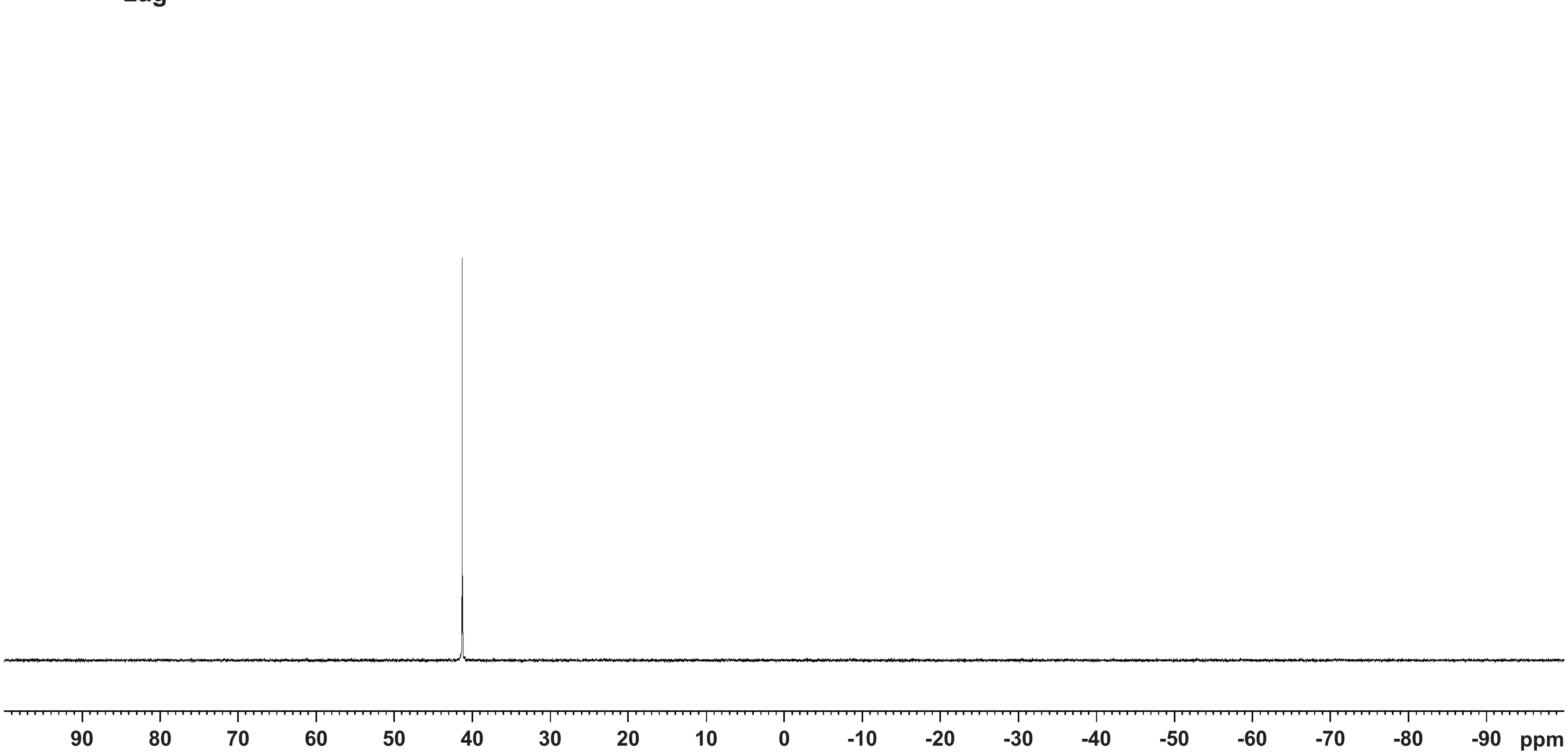

^{1}H NMR (400 MHz, $CDCl_3$)

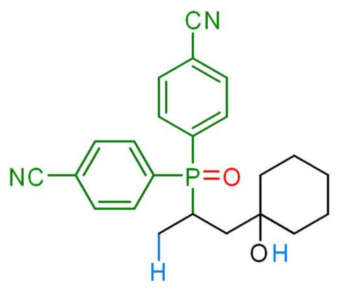

2ah

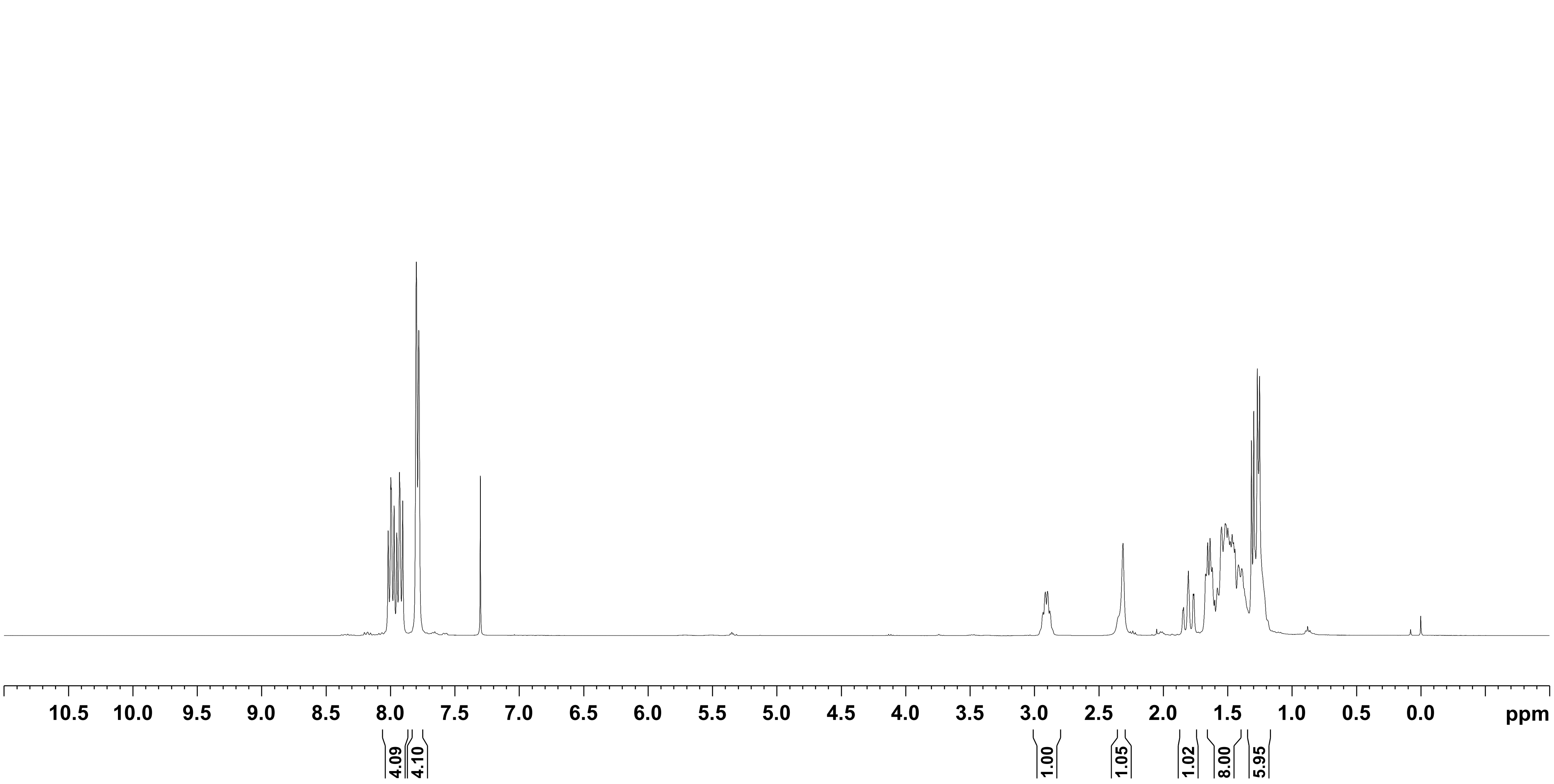

^{13}C NMR (100.6 MHz, $CDCl_3$)

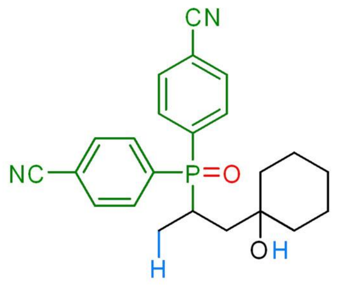

2ah

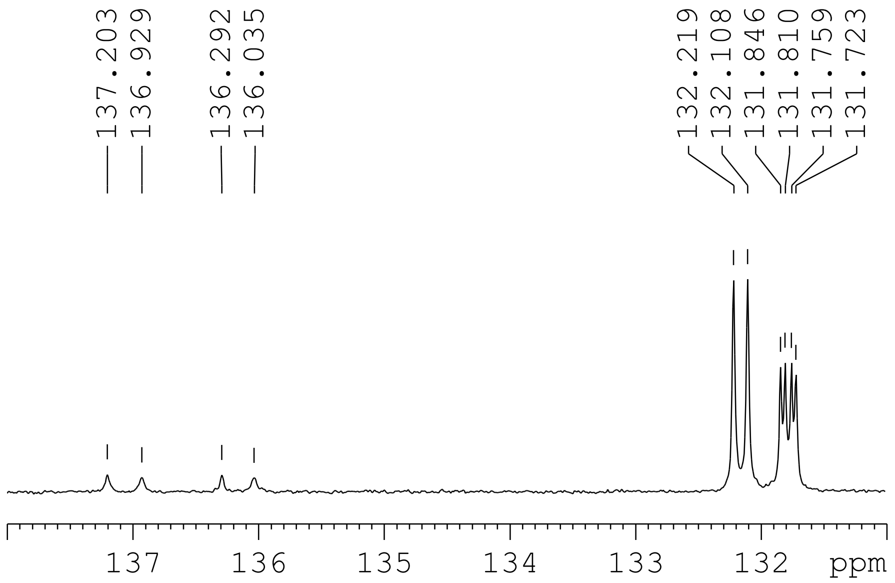

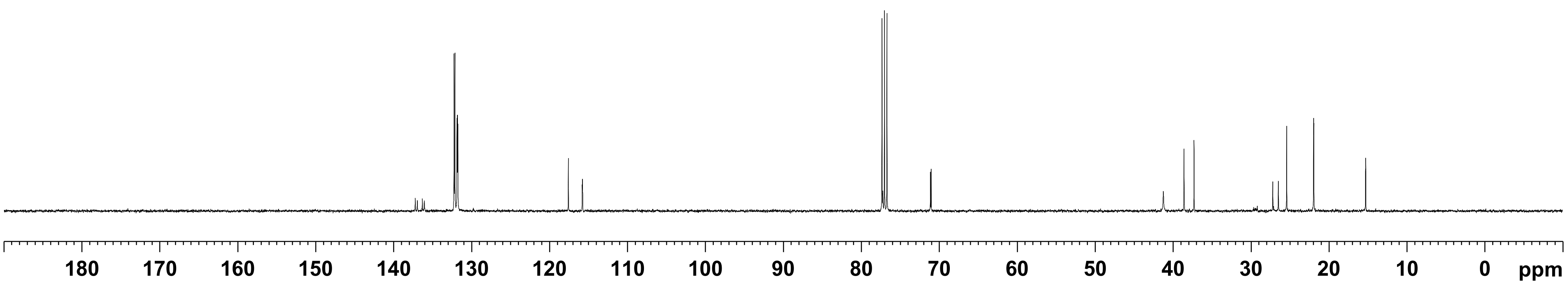

^{31}P NMR (162 MHz, $CDCl_3$)

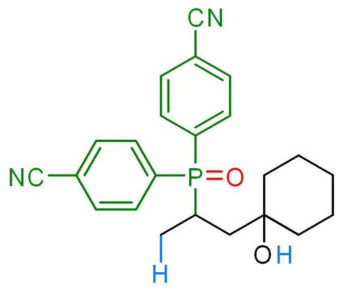

2ah

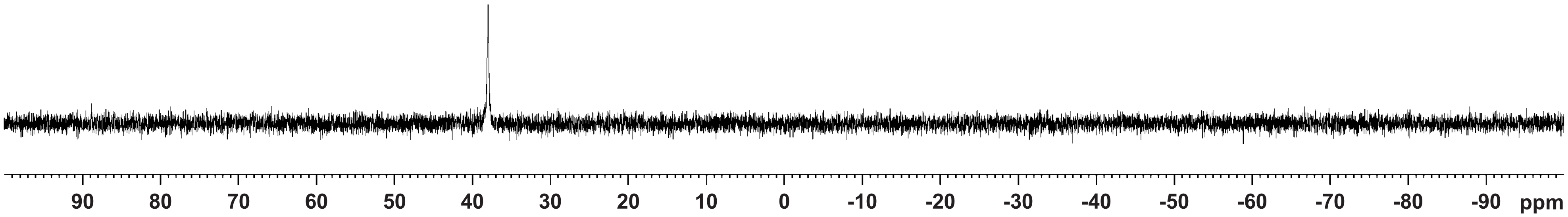

^{1}H NMR (400 MHz, CDCl$_3$)

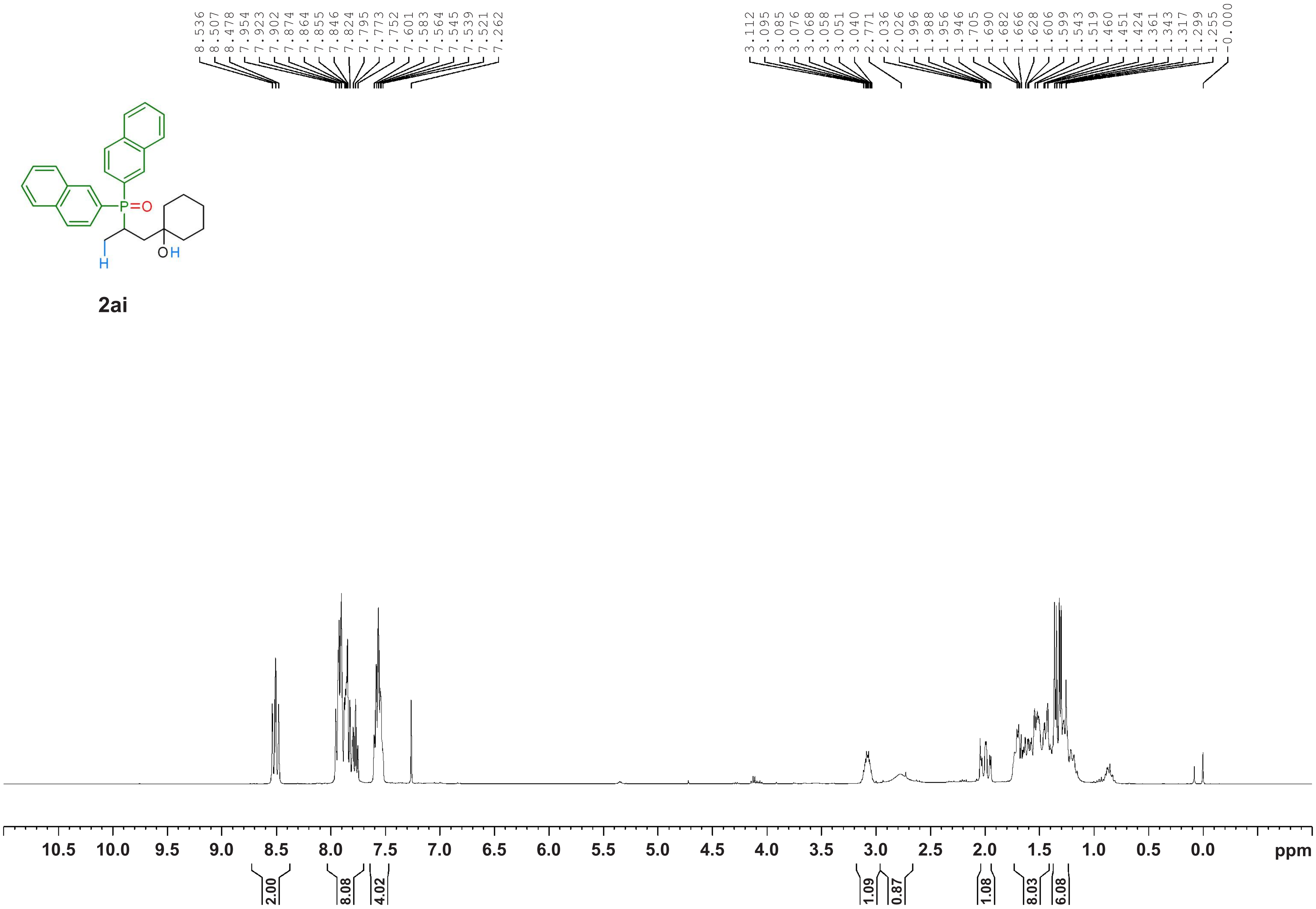

2ai

^{13}C NMR (100.6 MHz, $CDCl_3$)

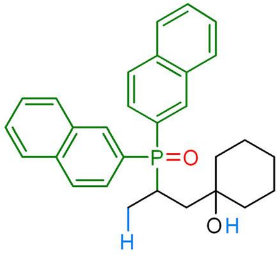

2ai

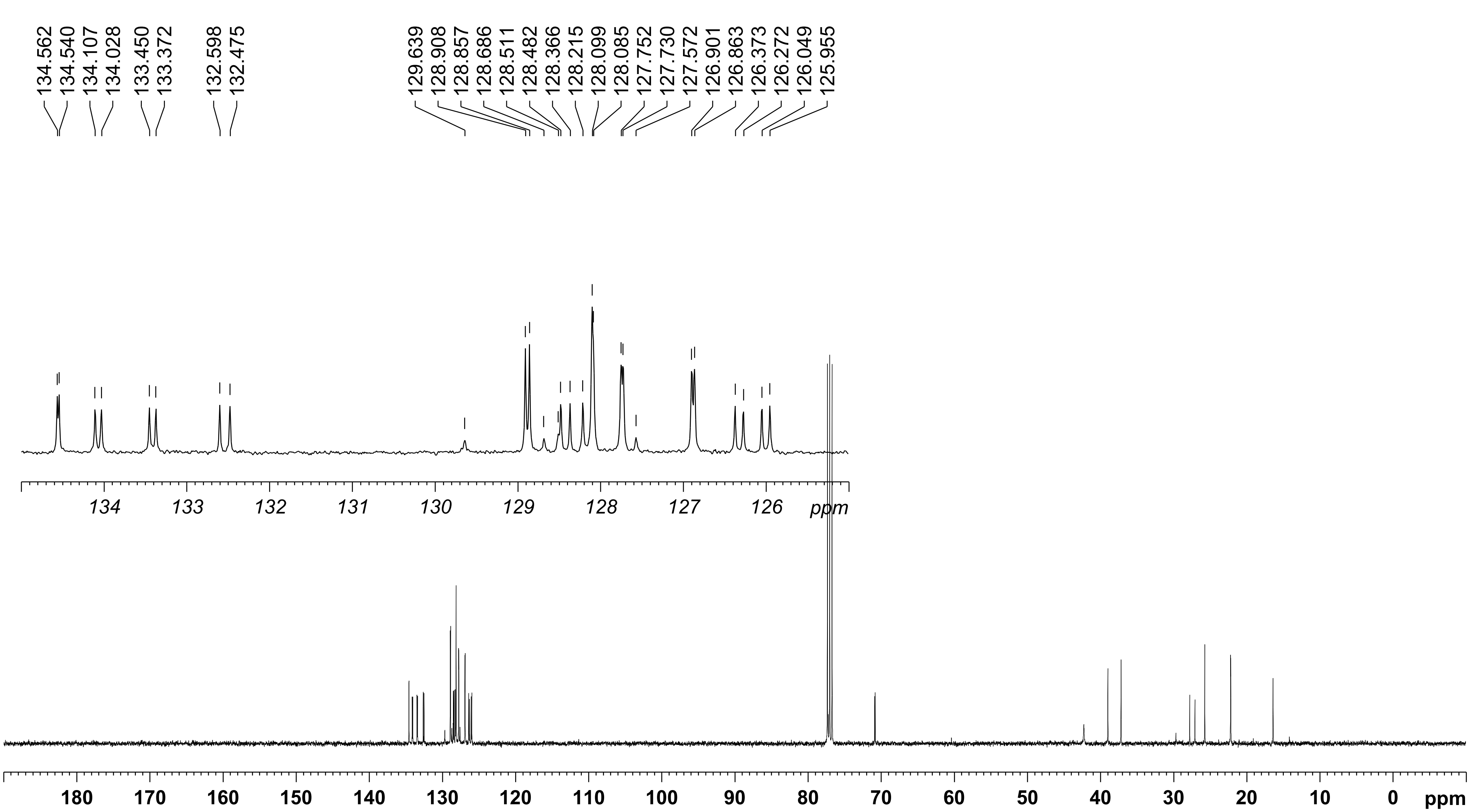

^{31}P NMR (162 MHz, $CDCl_3$)

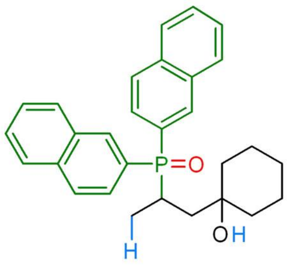

2ai

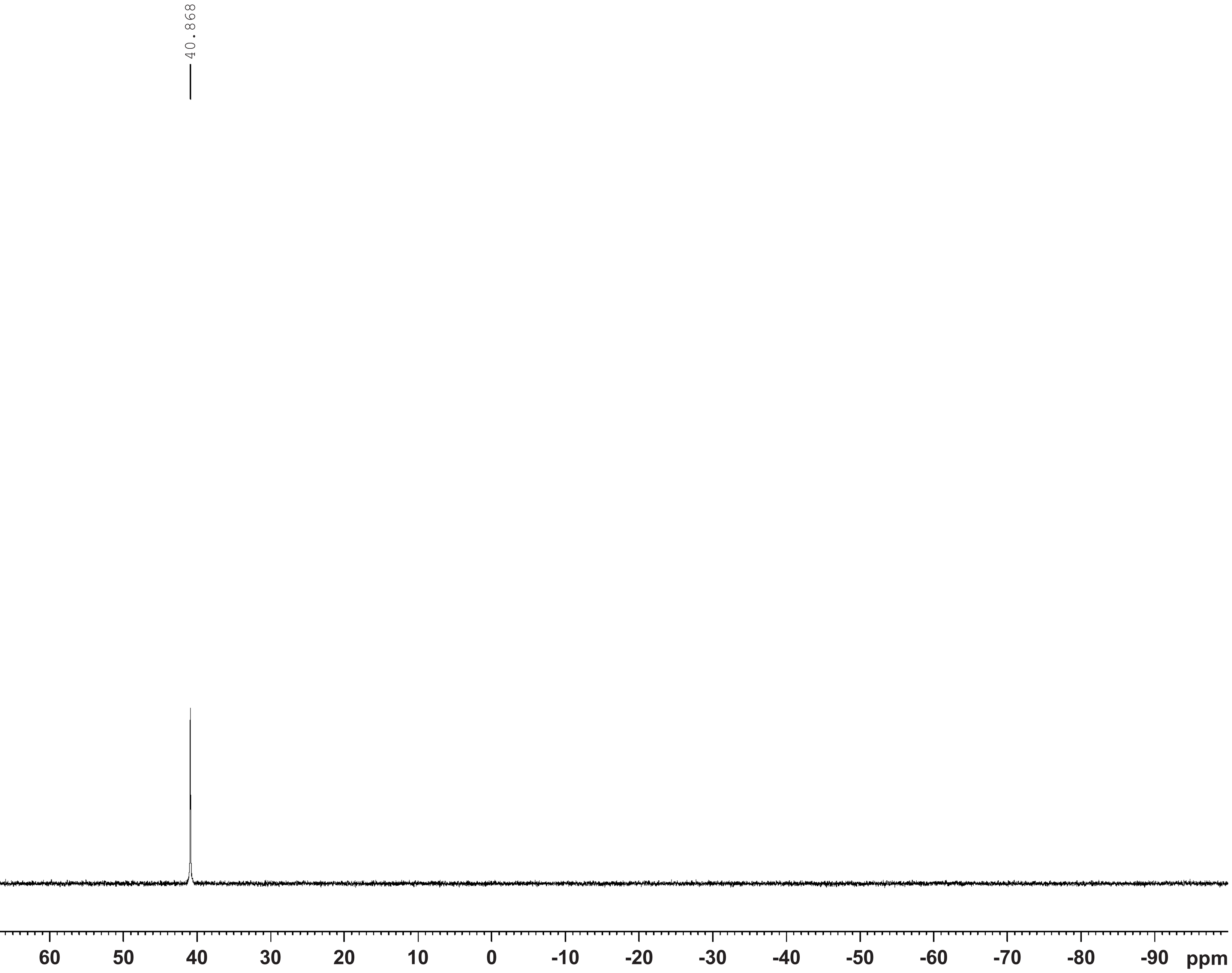

^{1}H NMR (400 MHz, $CDCl_3$)

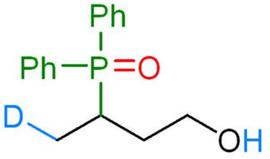

2a-D

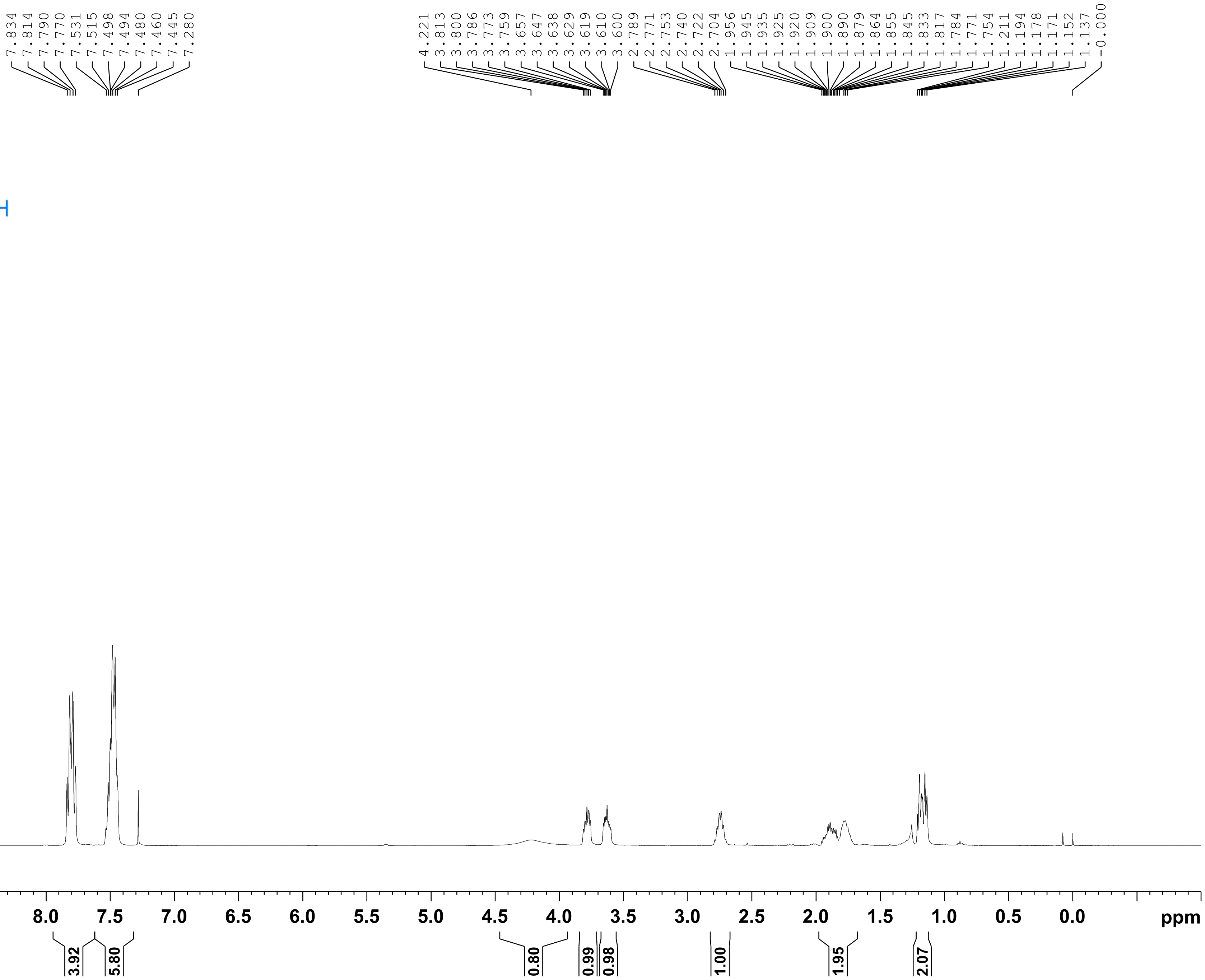

^{2}H NMR (92 MHz, $CHCl_3$)

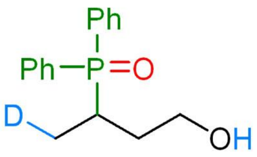

2a-D

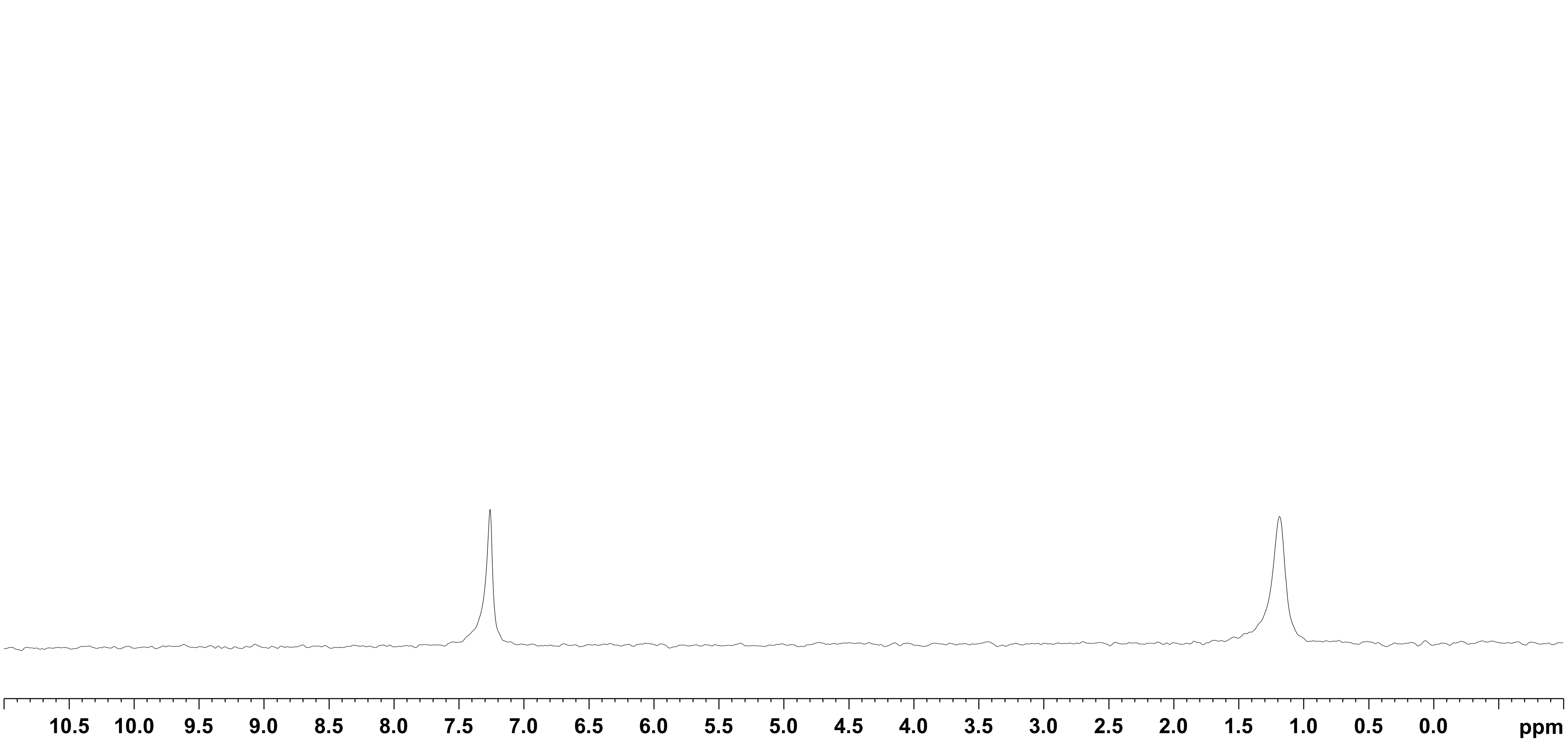

^{13}C NMR (100.6 MHz, $CDCl_3$)

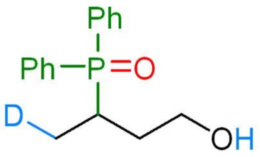

2a-D

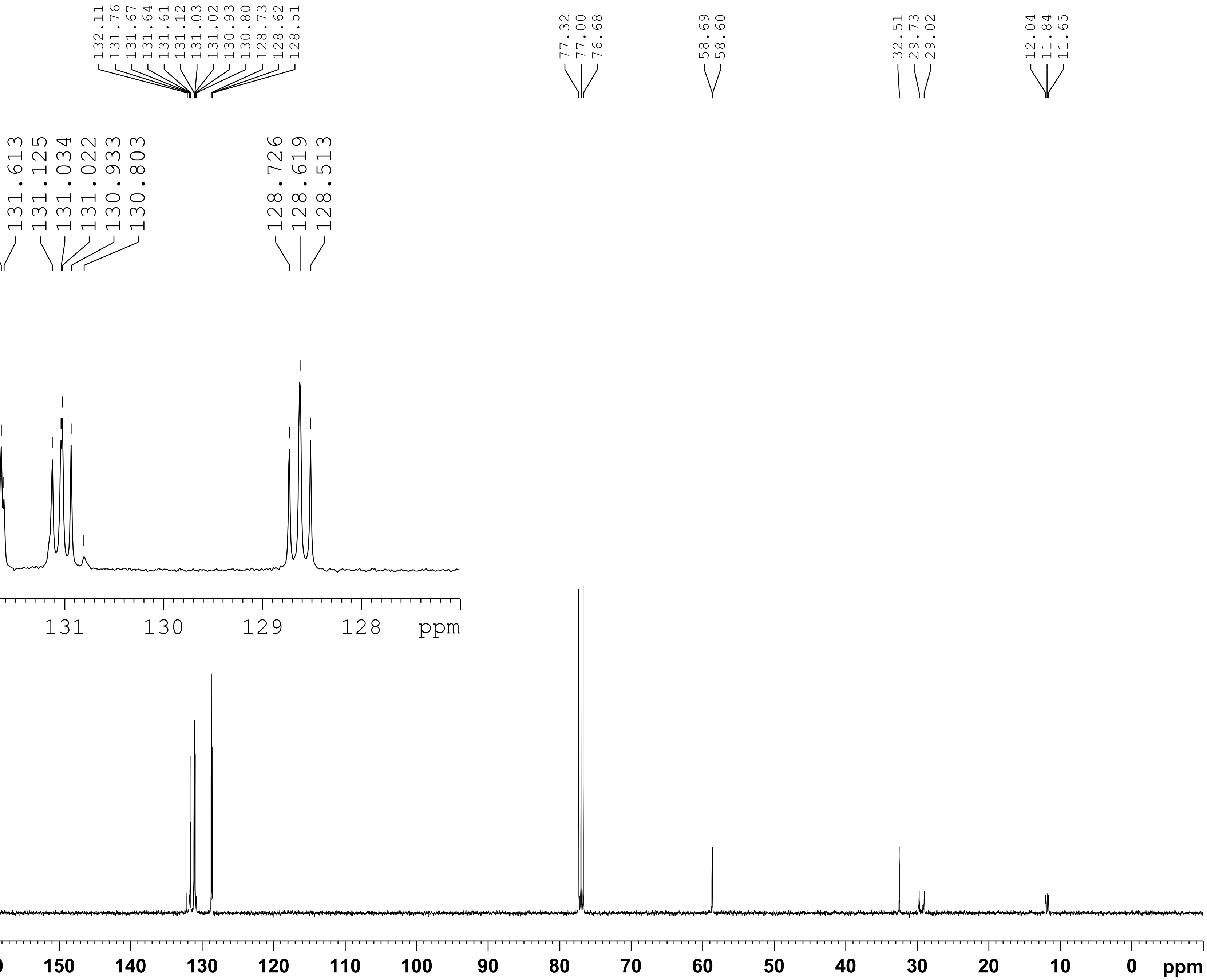

^{31}P NMR (162 MHz, $CDCl_3$)

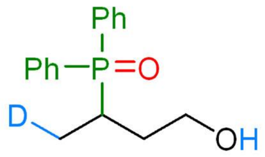

2a-D

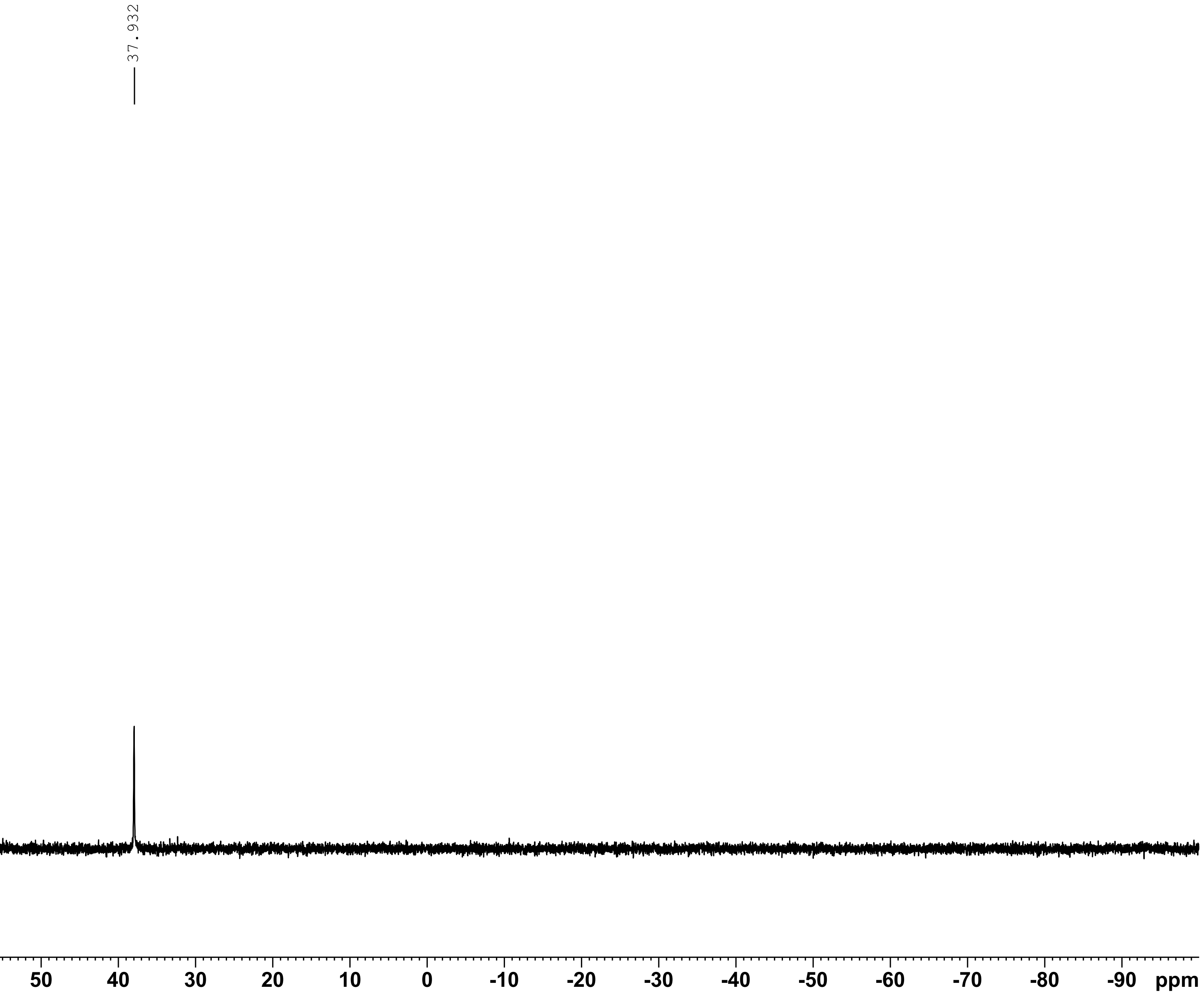

^{1}H NMR (400 MHz, $CDCl_3$)

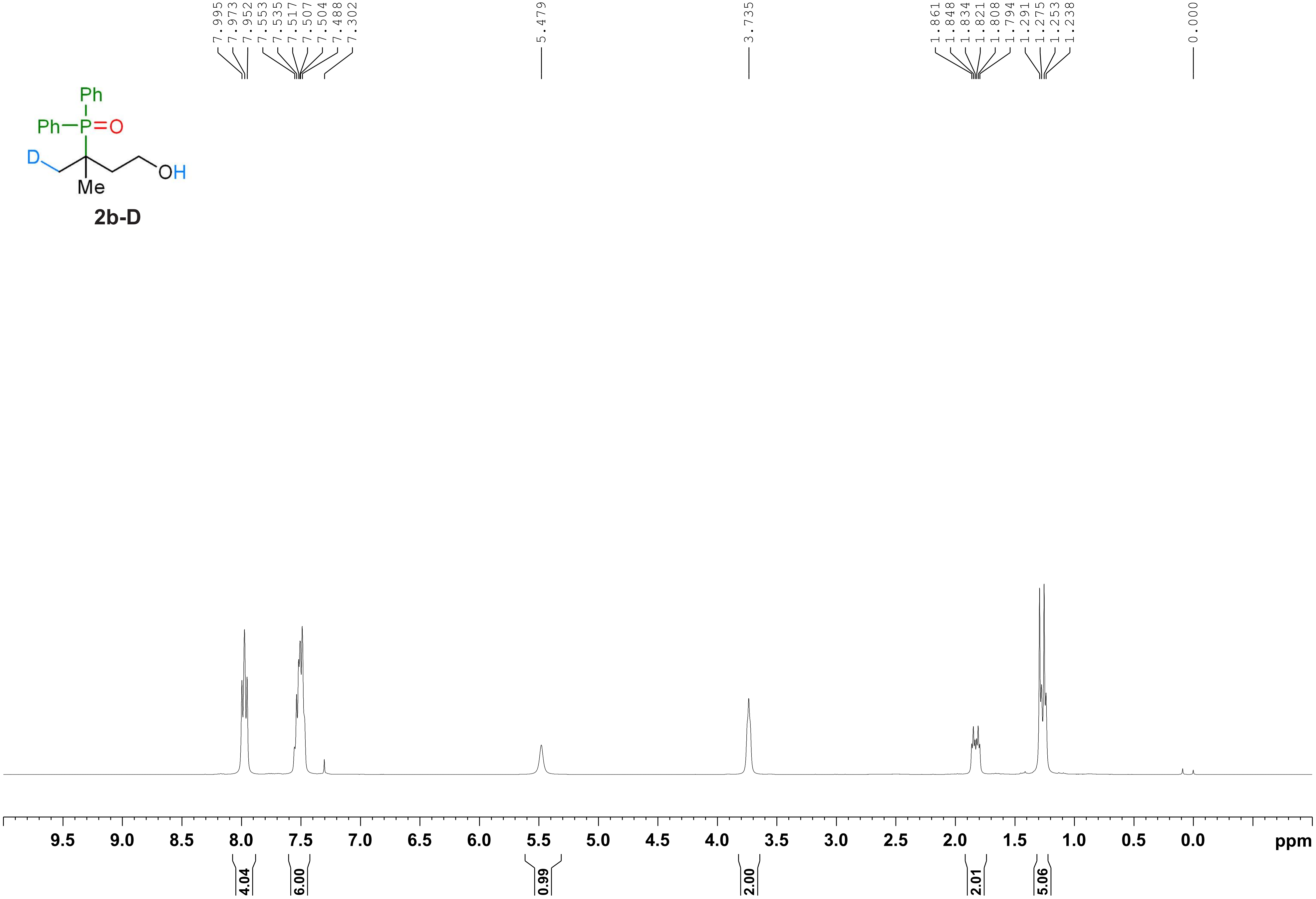

^{2}H NMR (92 MHz, $CHCl_3$)

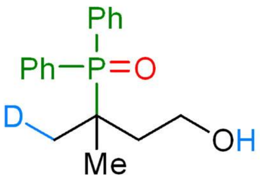

2b-D

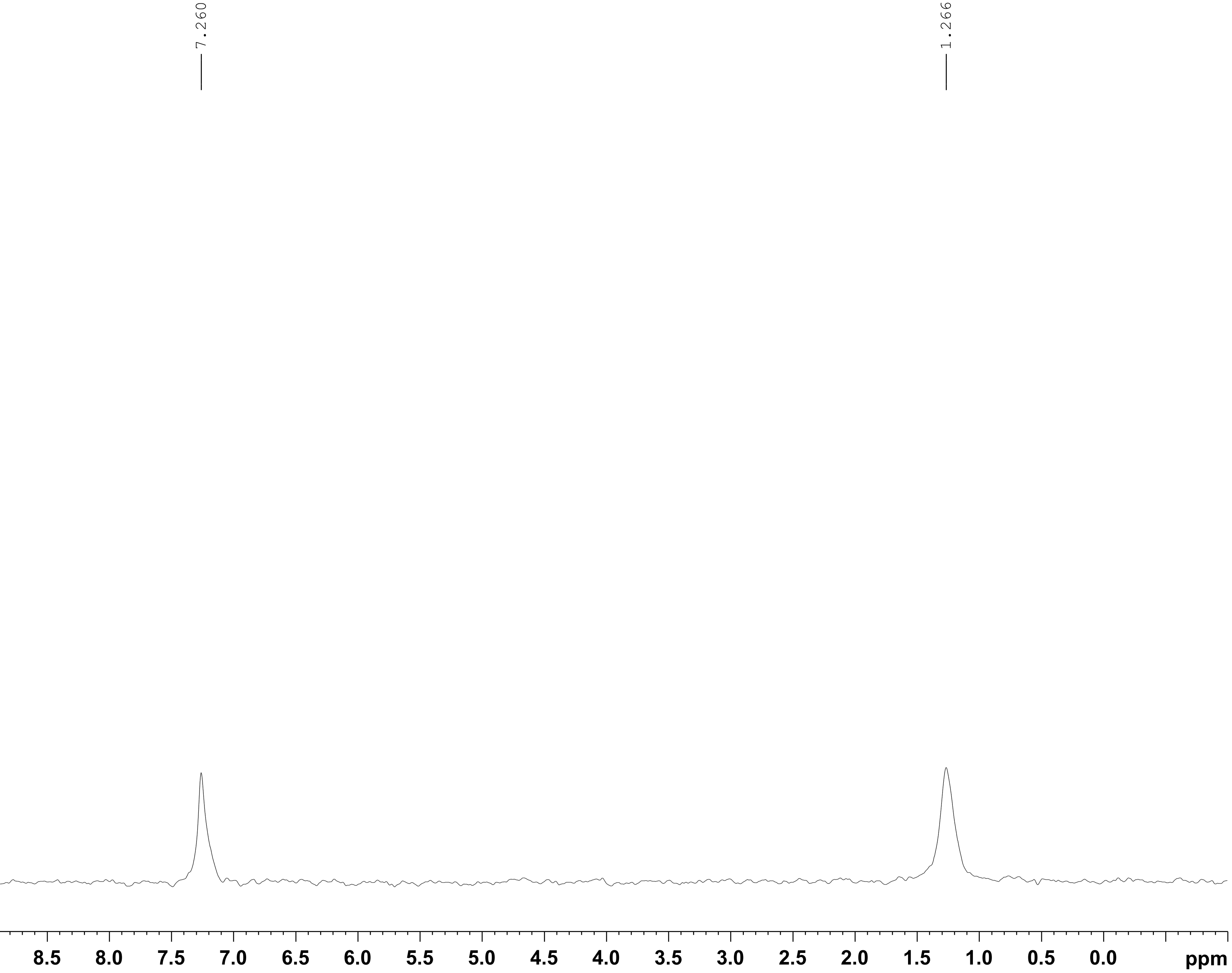

^{13}C NMR (100.6 MHz, $CDCl_3$)

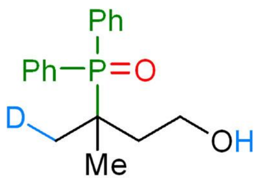

2b-D

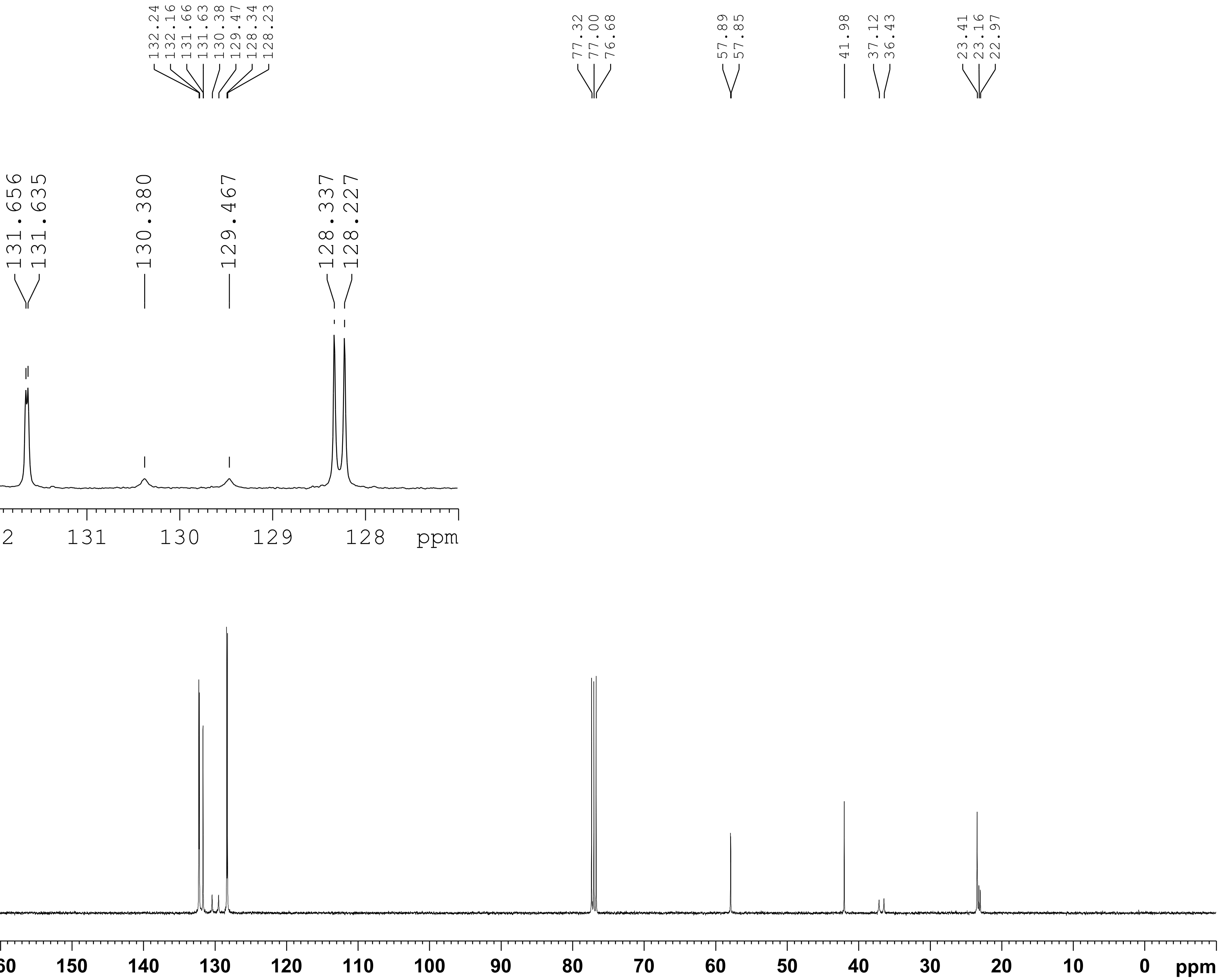

^{31}P NMR (162 MHz, $CDCl_3$)

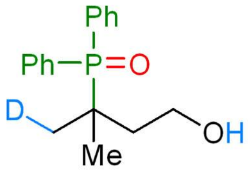

2b-D

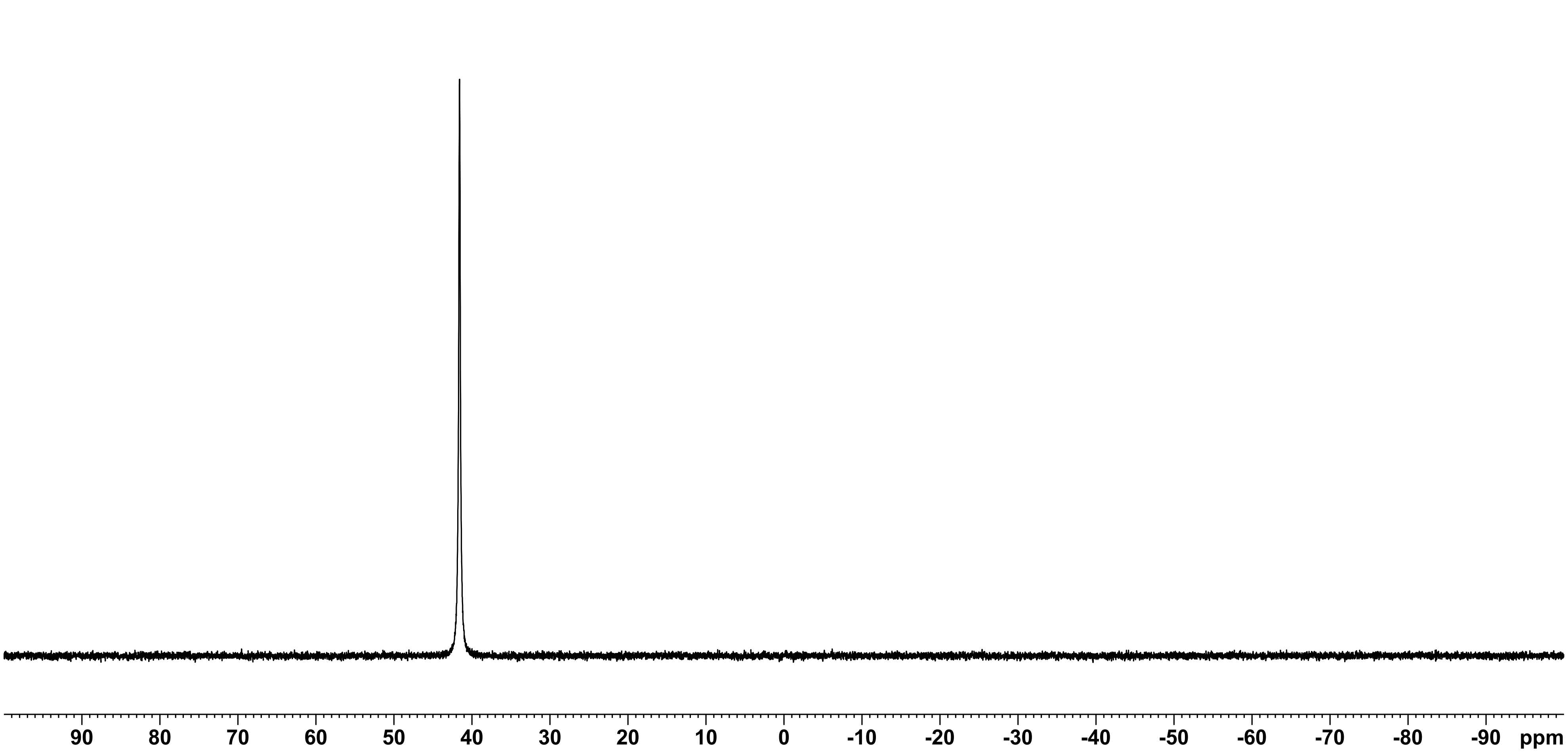

^{1}H NMR (400 MHz, $CDCl_3$)

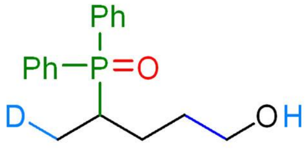

2c-D

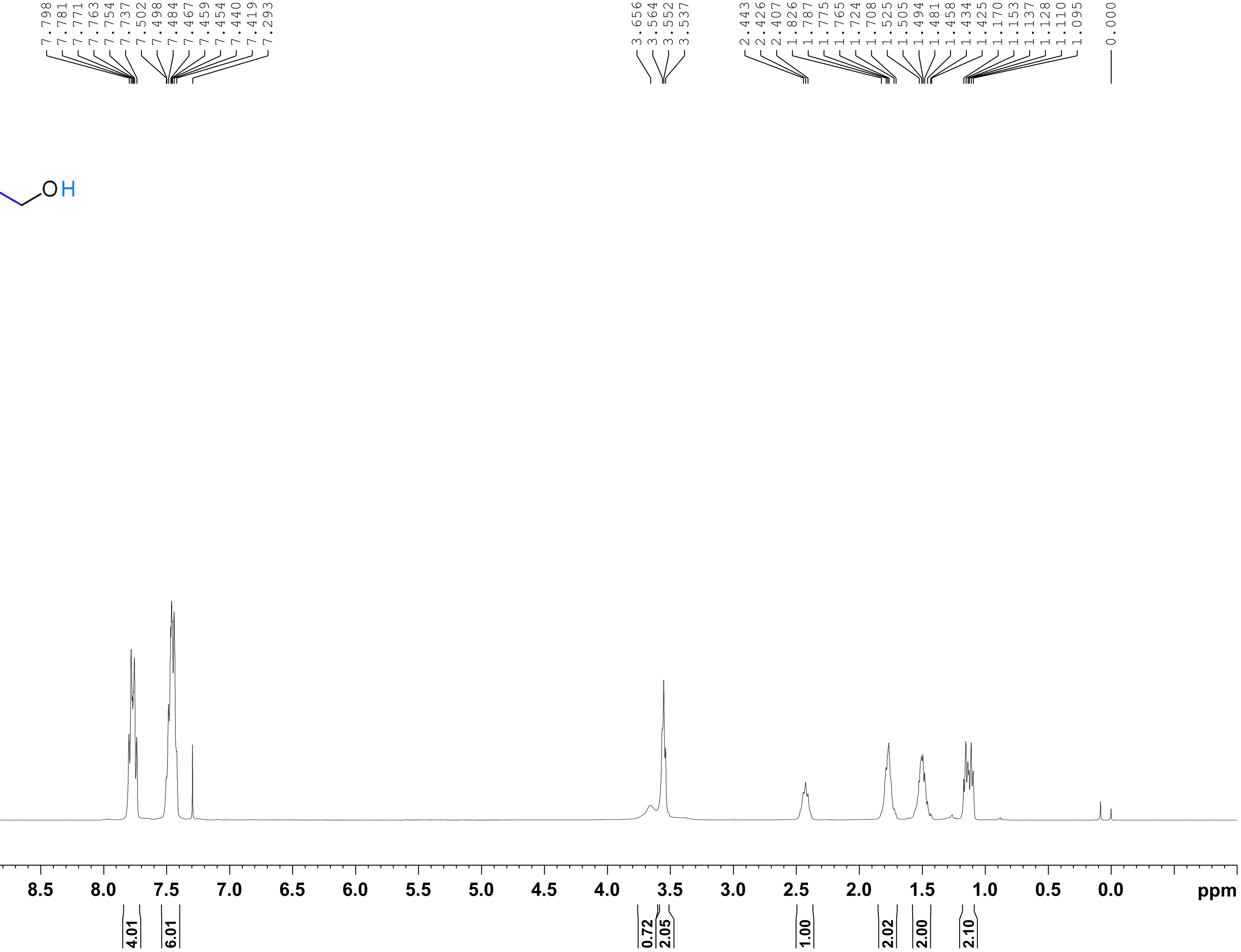

^2H NMR (92 MHz, $CHCl_3$)

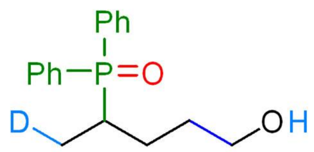

2c-D

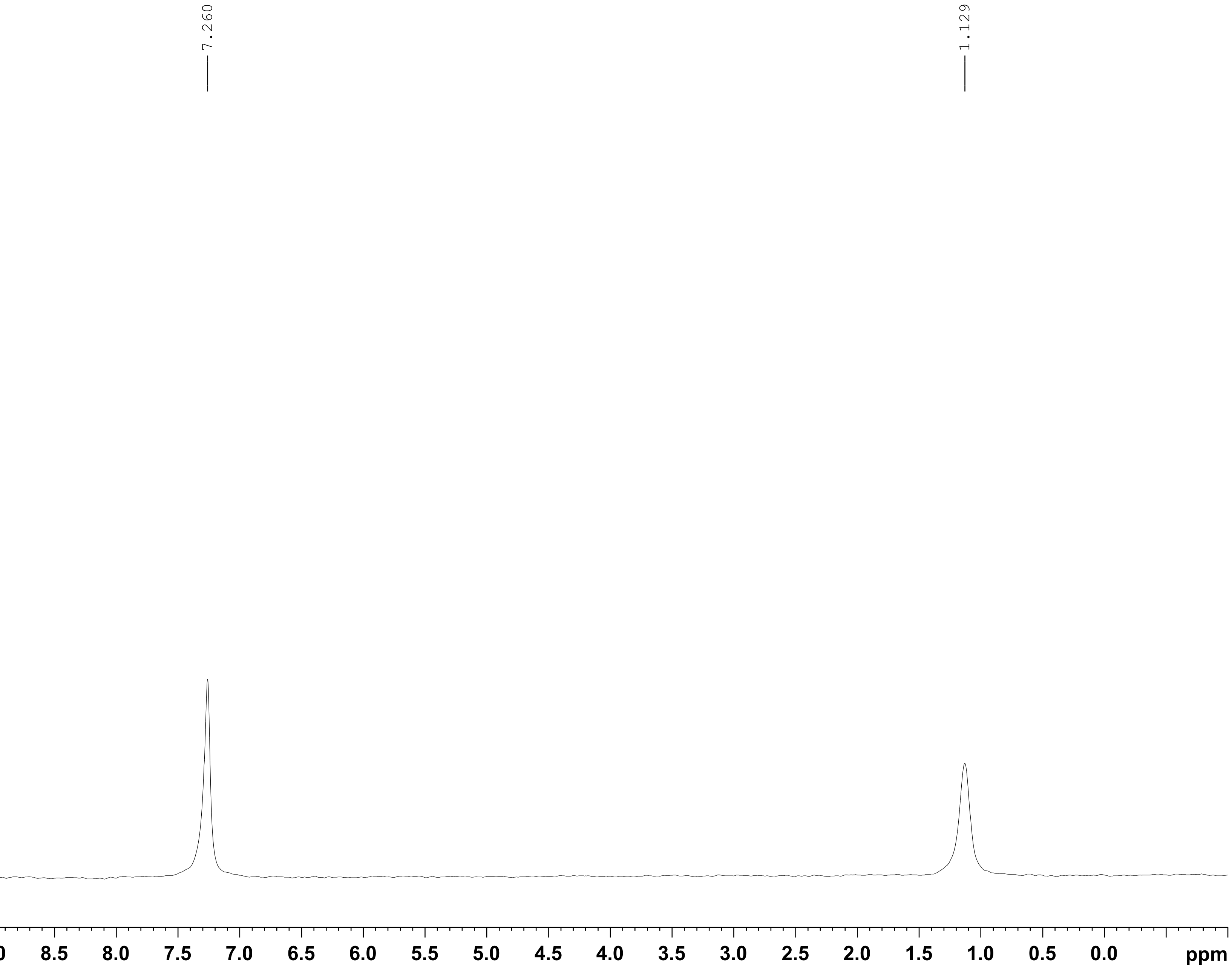

^{13}C NMR (100.6 MHz, $CDCl_3$)

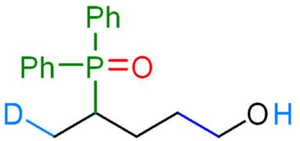

2c-D

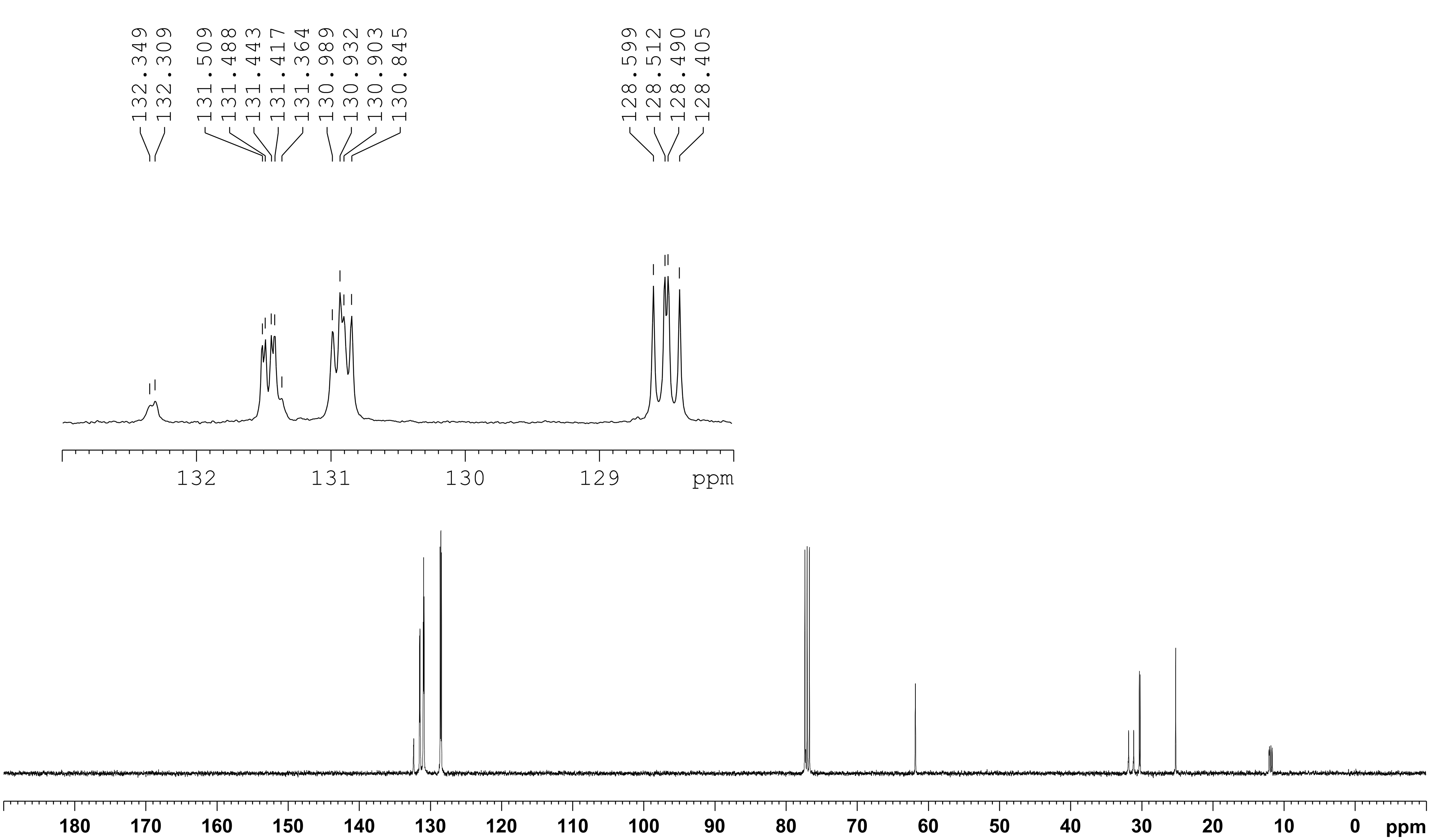

^{31}P NMR (162 MHz, $CDCl_3$)

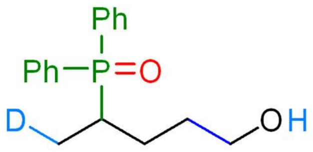

2c-D

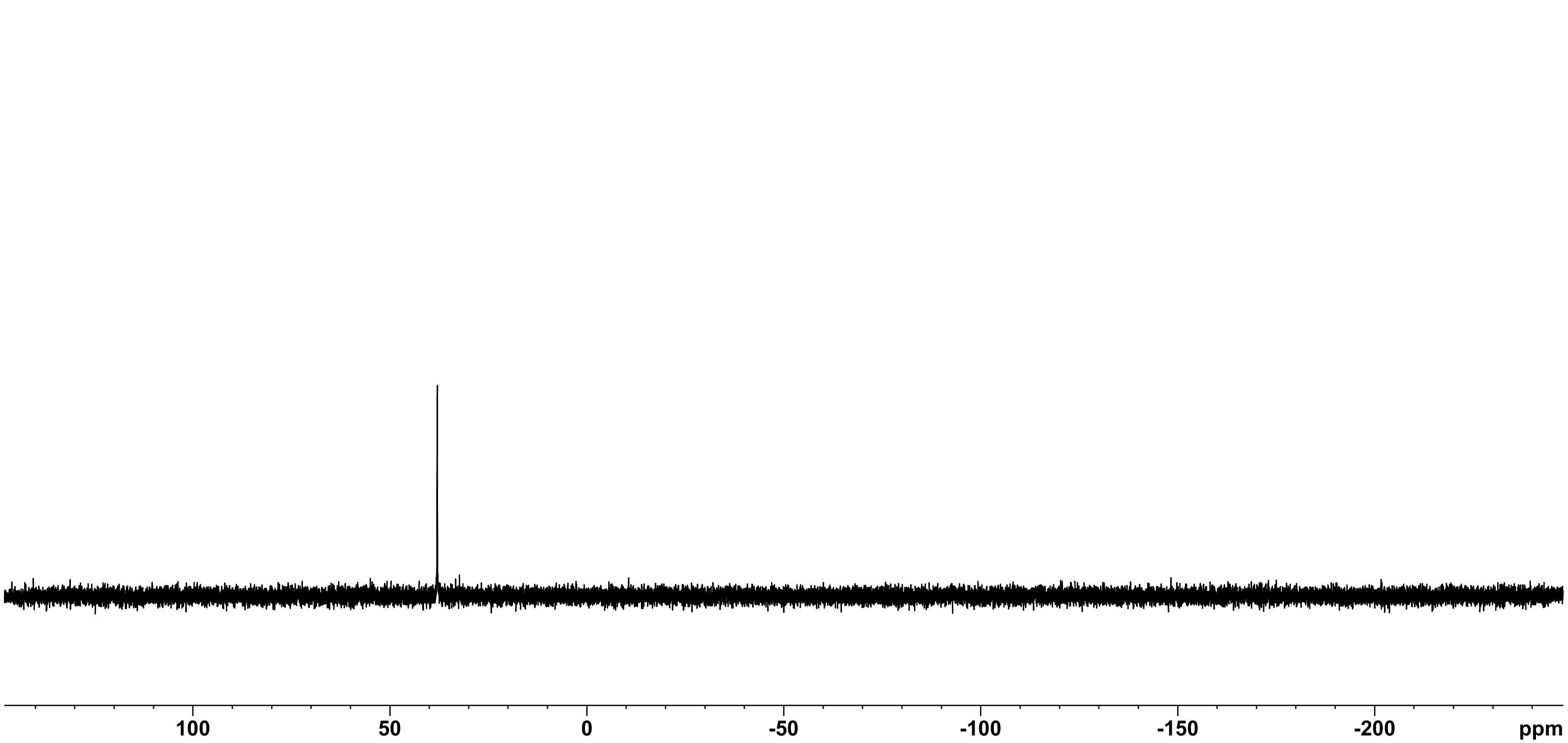

^{1}H NMR (400 MHz, $CDCl_3$)

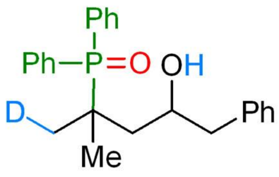

2h-D

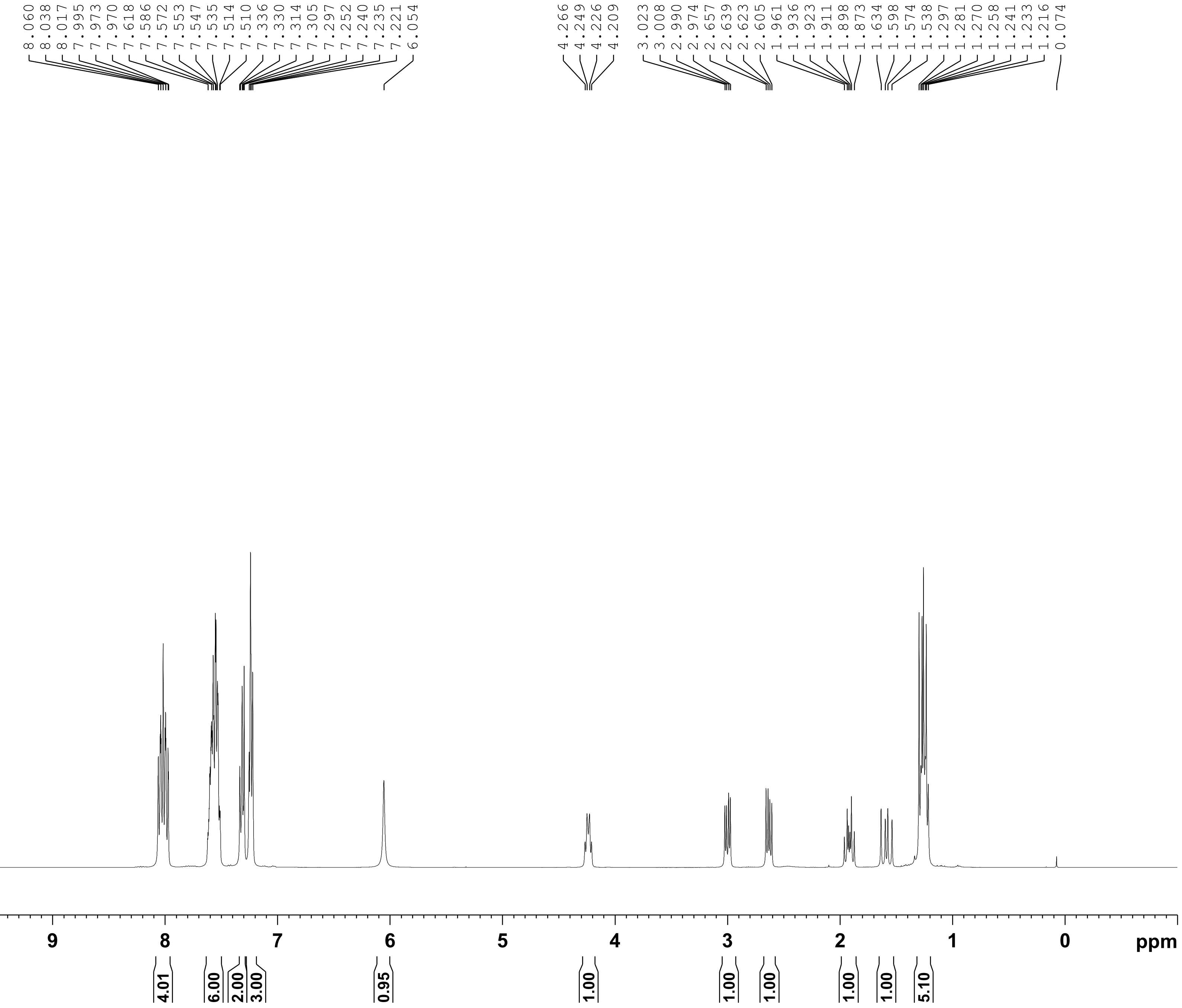

^{2}H NMR (92 MHz, $CHCl_3$)

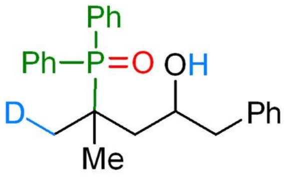

2h-D

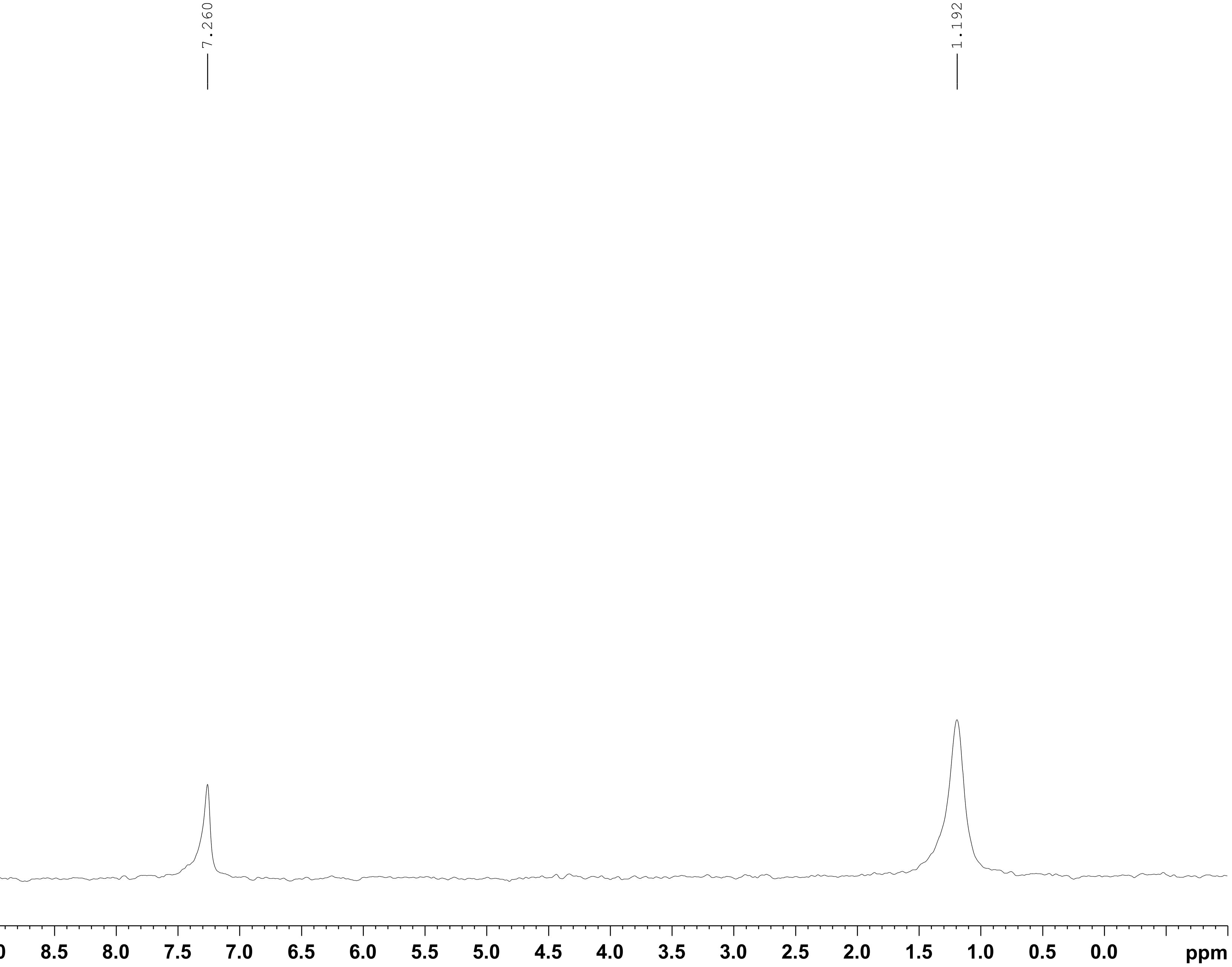

^{13}C NMR (100.6 MHz, $CDCl_3$)

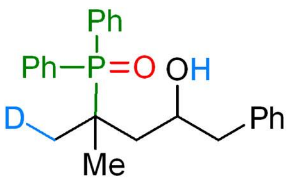

2h-D

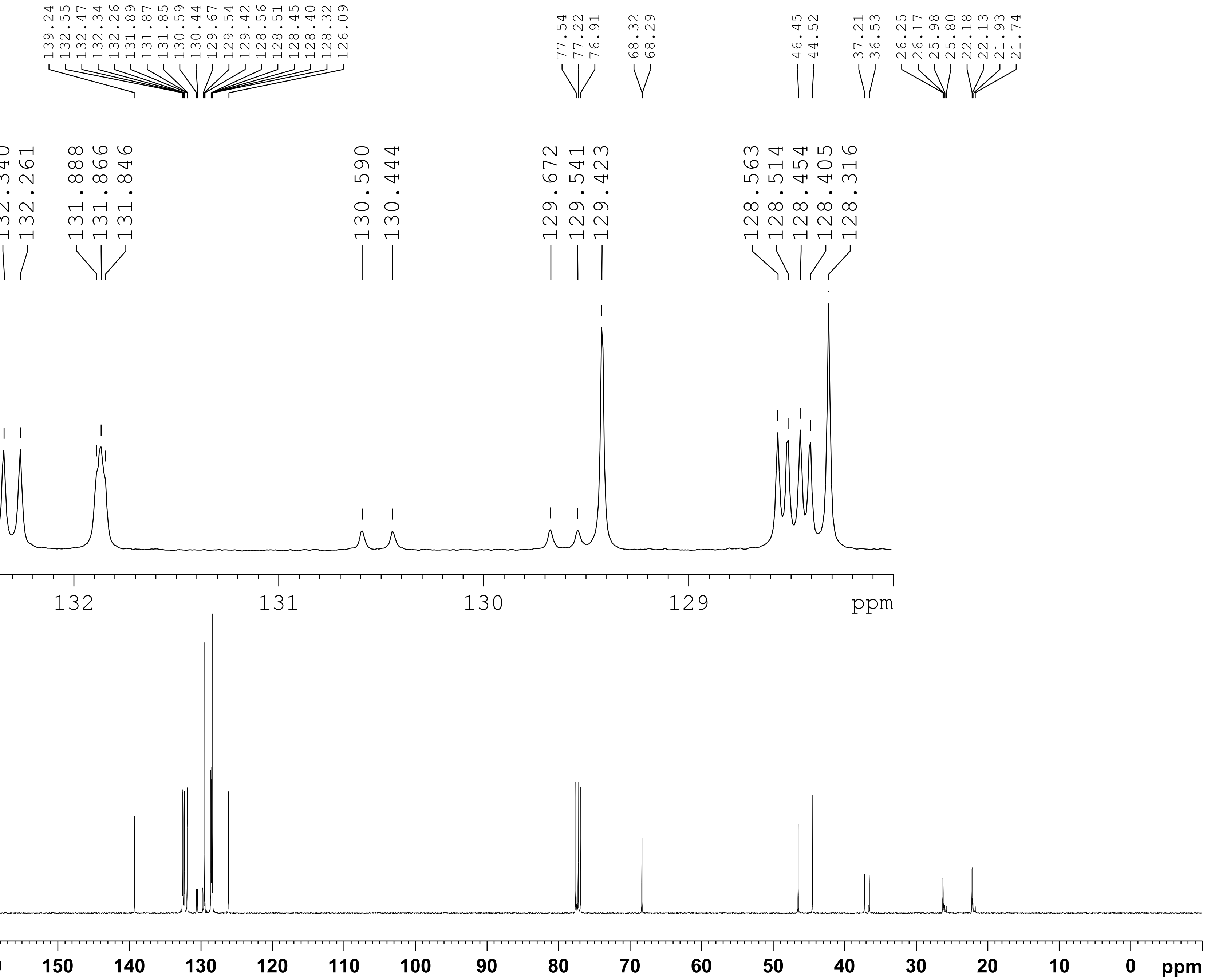

^{31}P NMR (162 MHz, $CDCl_3$)

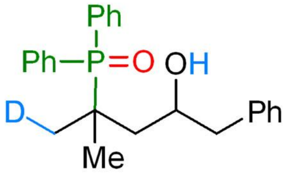

2h-D

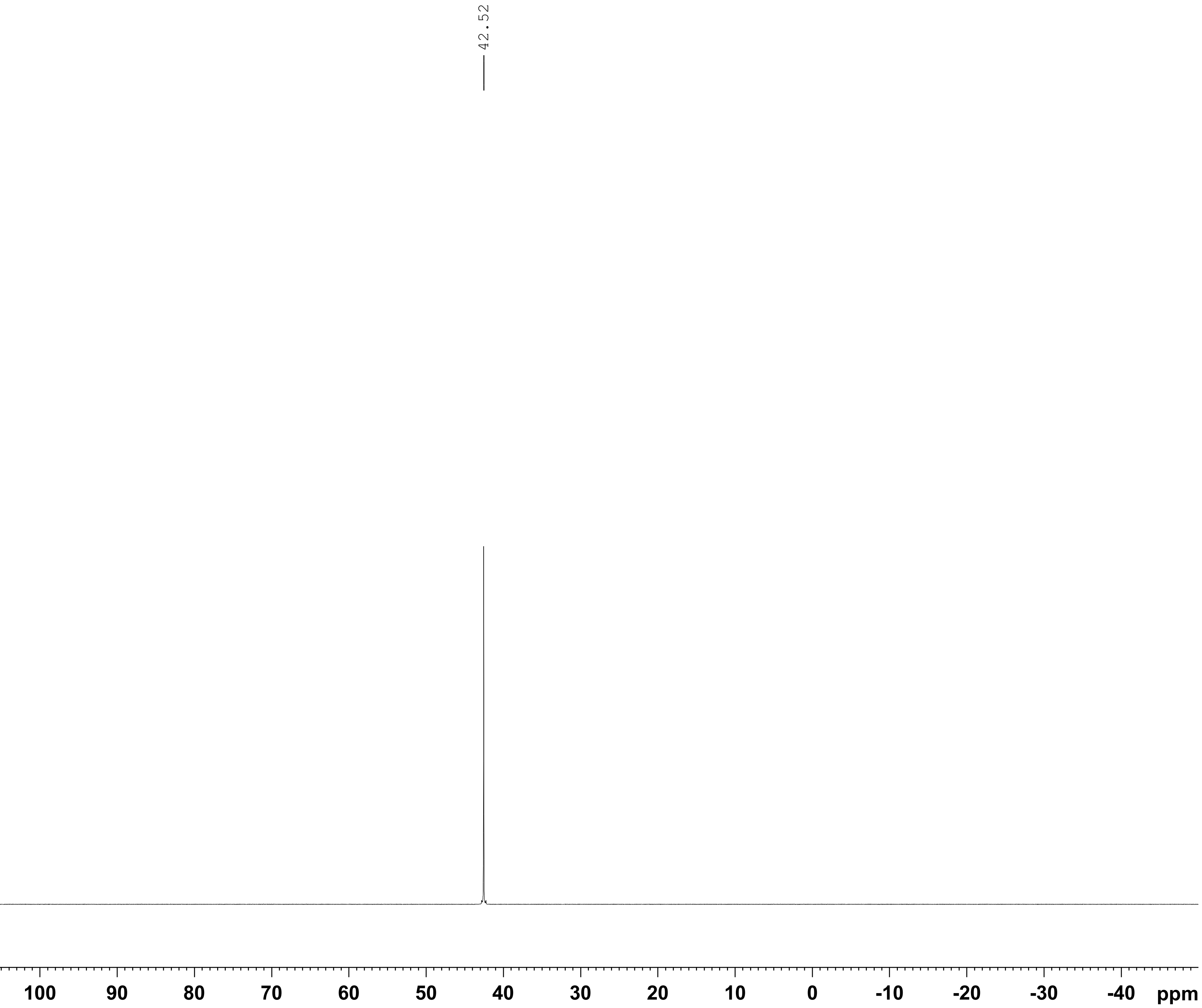

^{1}H NMR (400 MHz, $CDCl_3$)

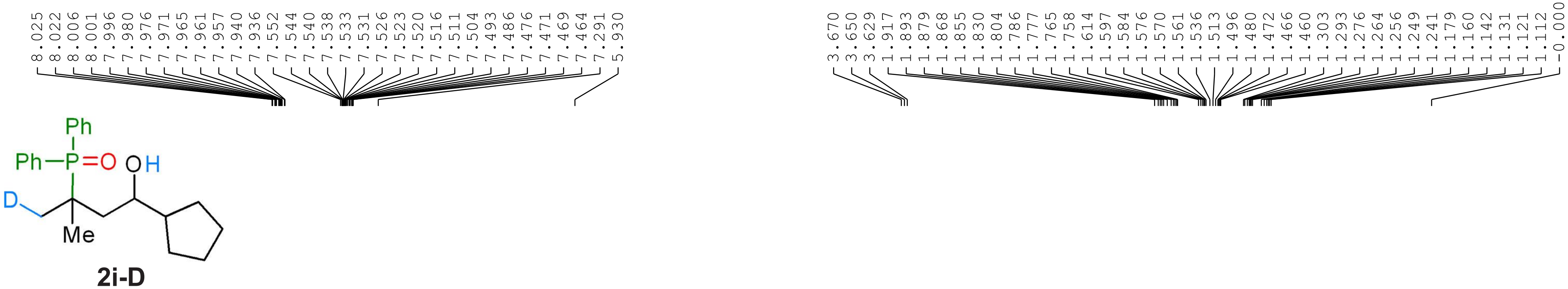

2i-D

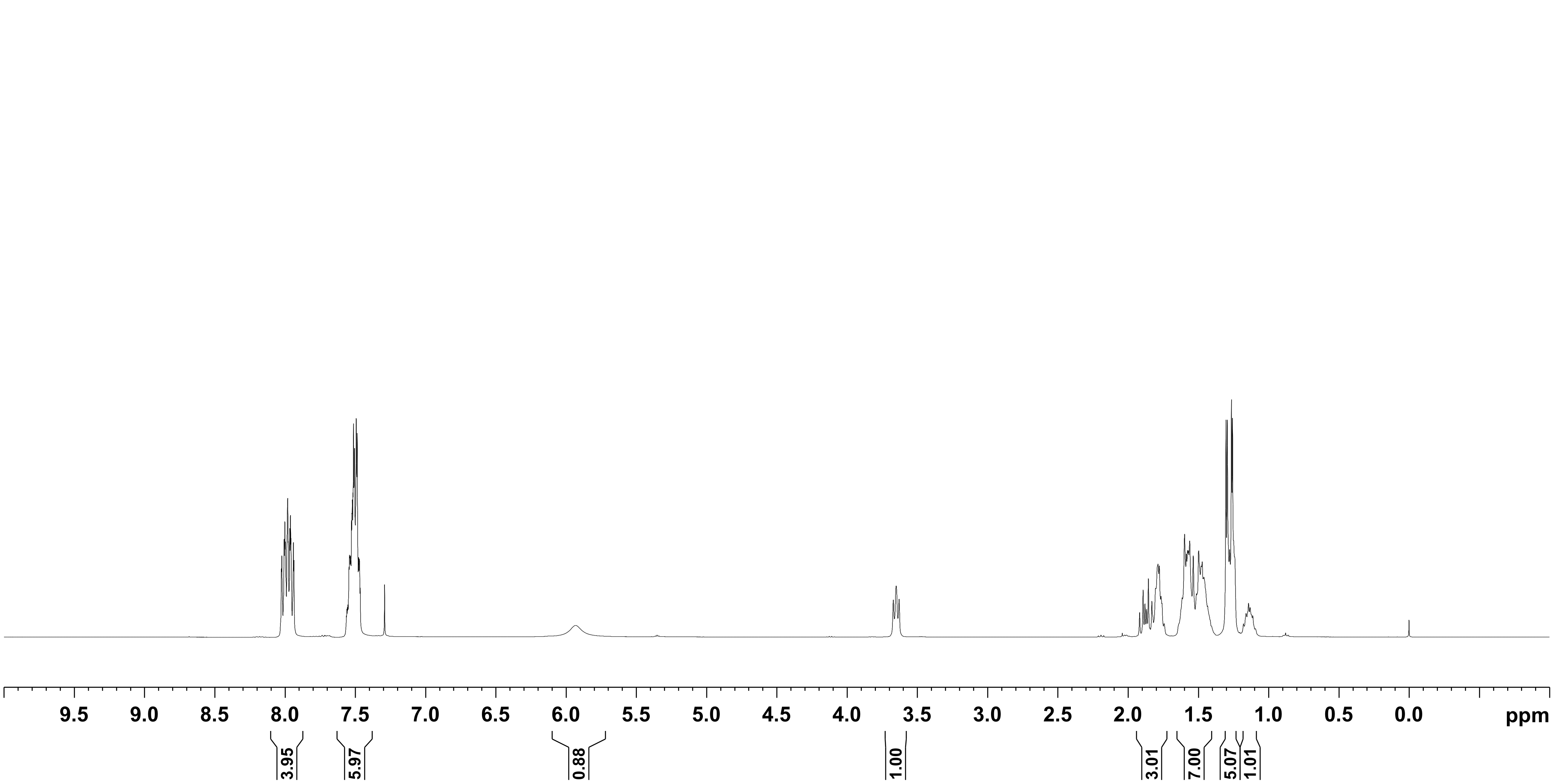

^{2}H NMR (92 MHz, $CHCl_3$)

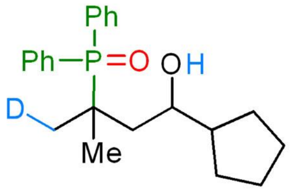

2i-D

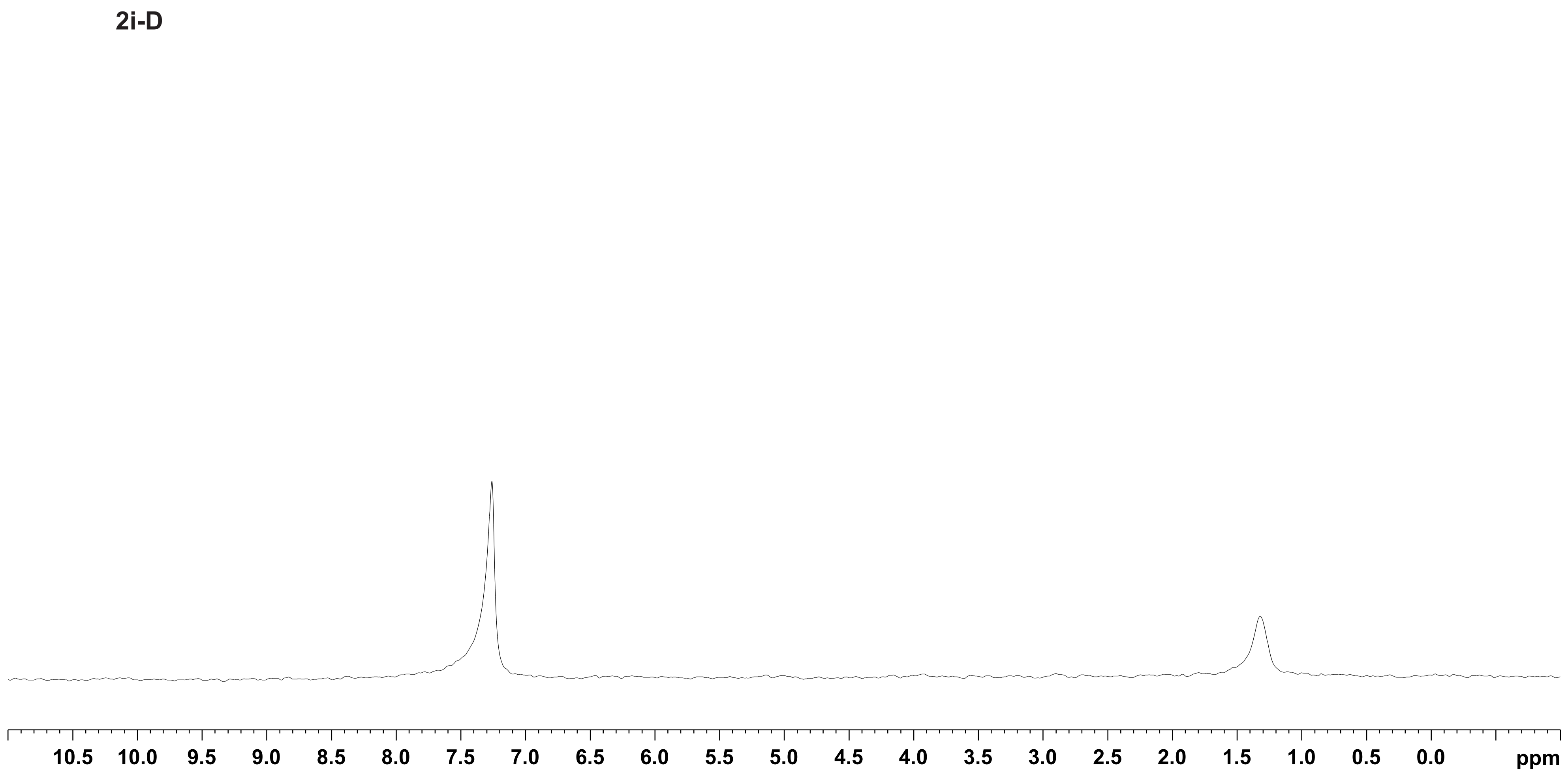

^{13}C NMR (100.6 MHz, $CDCl_3$)

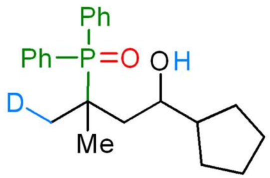

2i-D

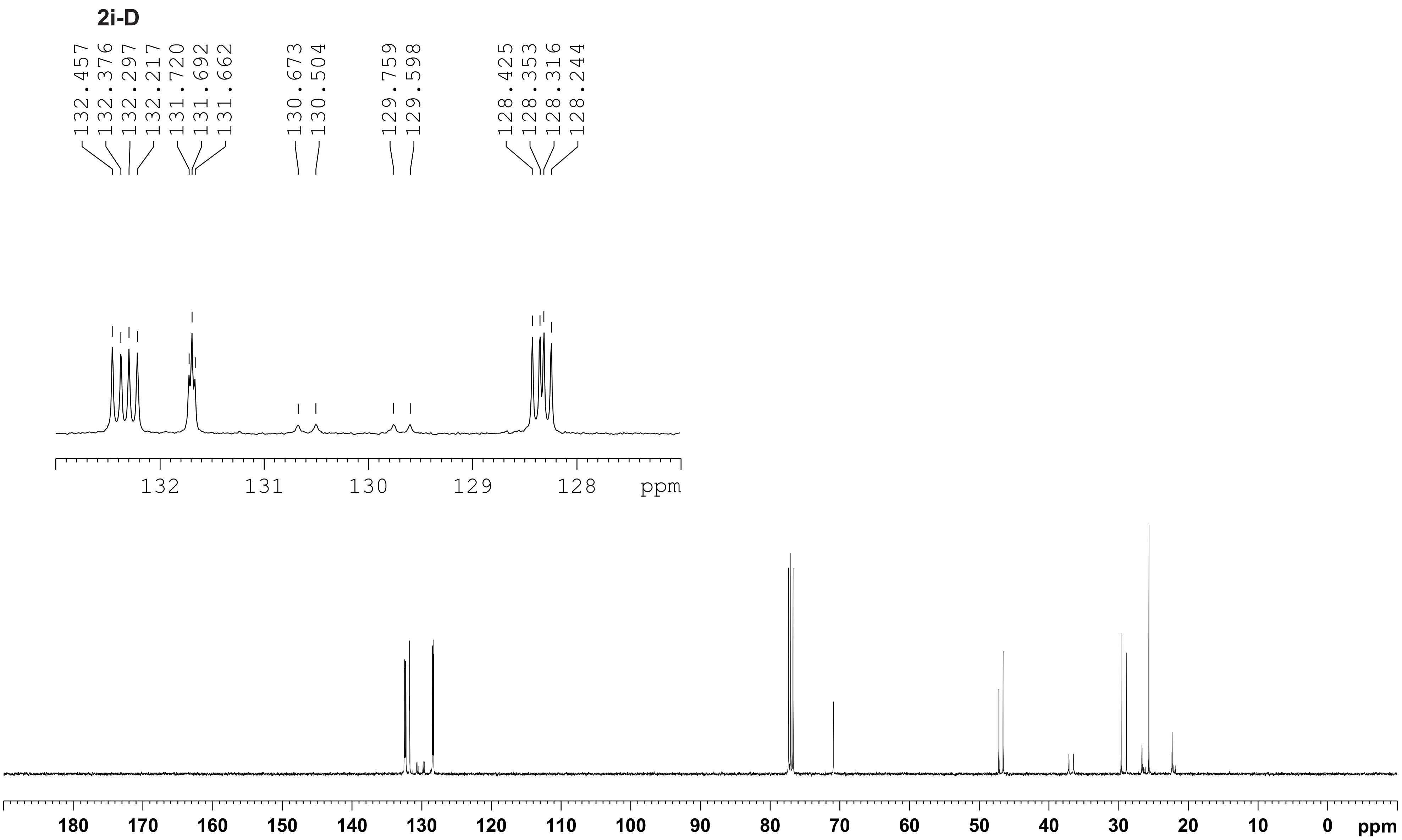

^{31}P NMR (162 MHz, $CDCl_3$)

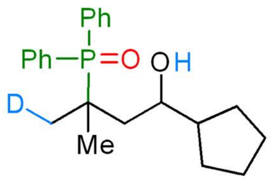

2i-D

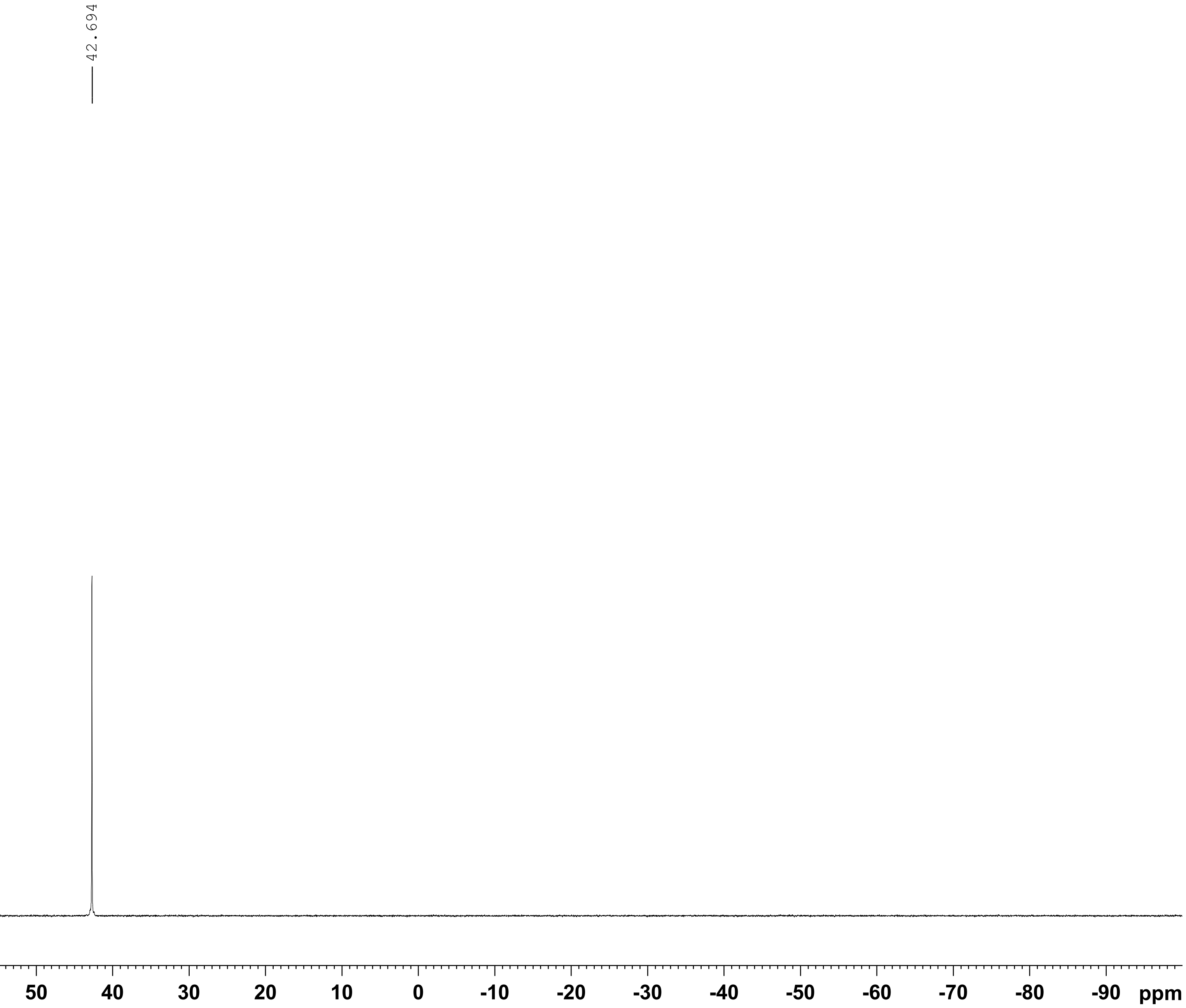

^{1}H NMR (400 MHz, $CDCl_3$)

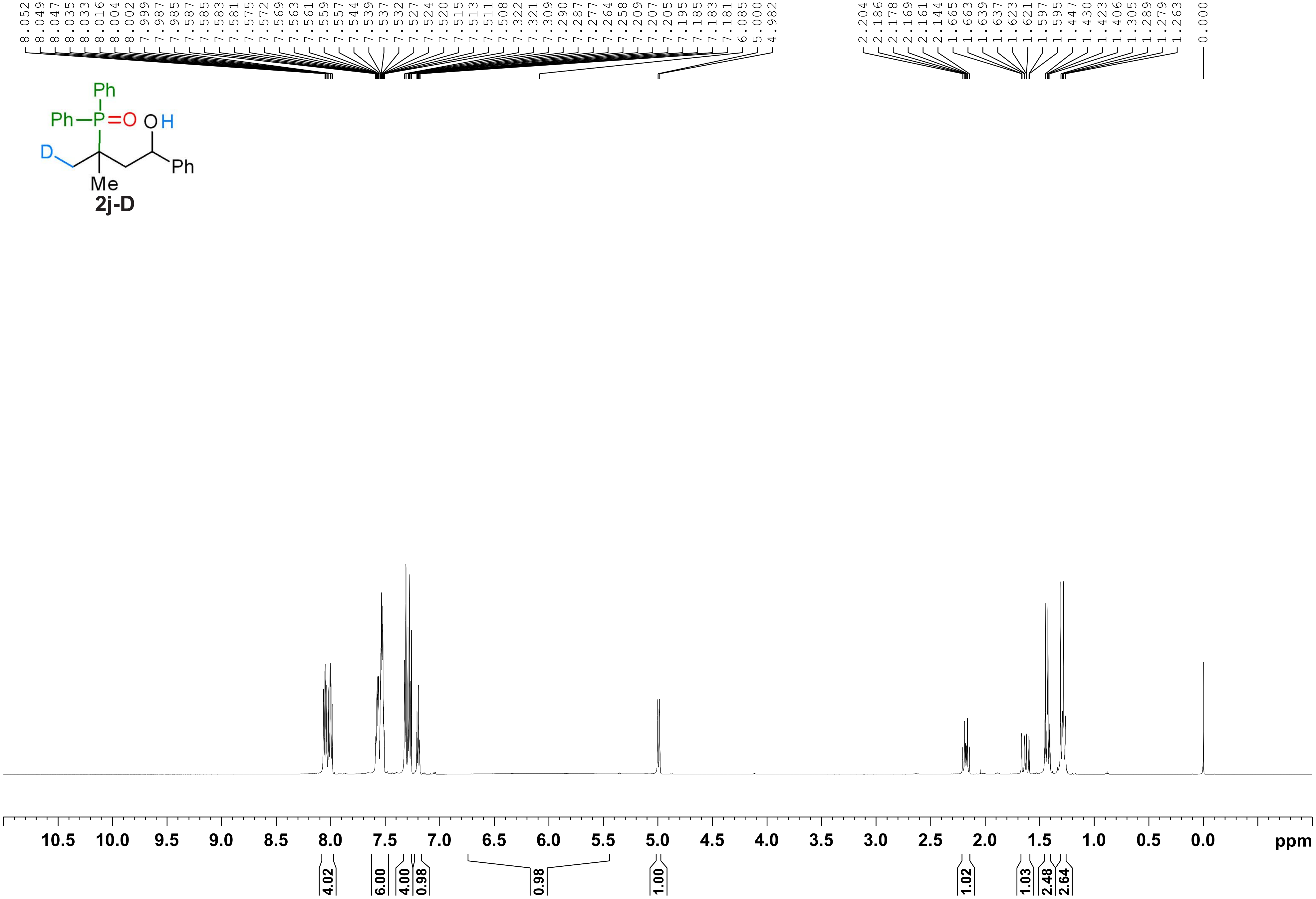

^{2}H NMR (92 MHz, $CHCl_3$)

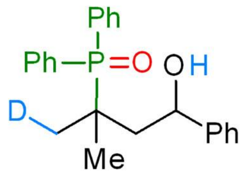

2j-D

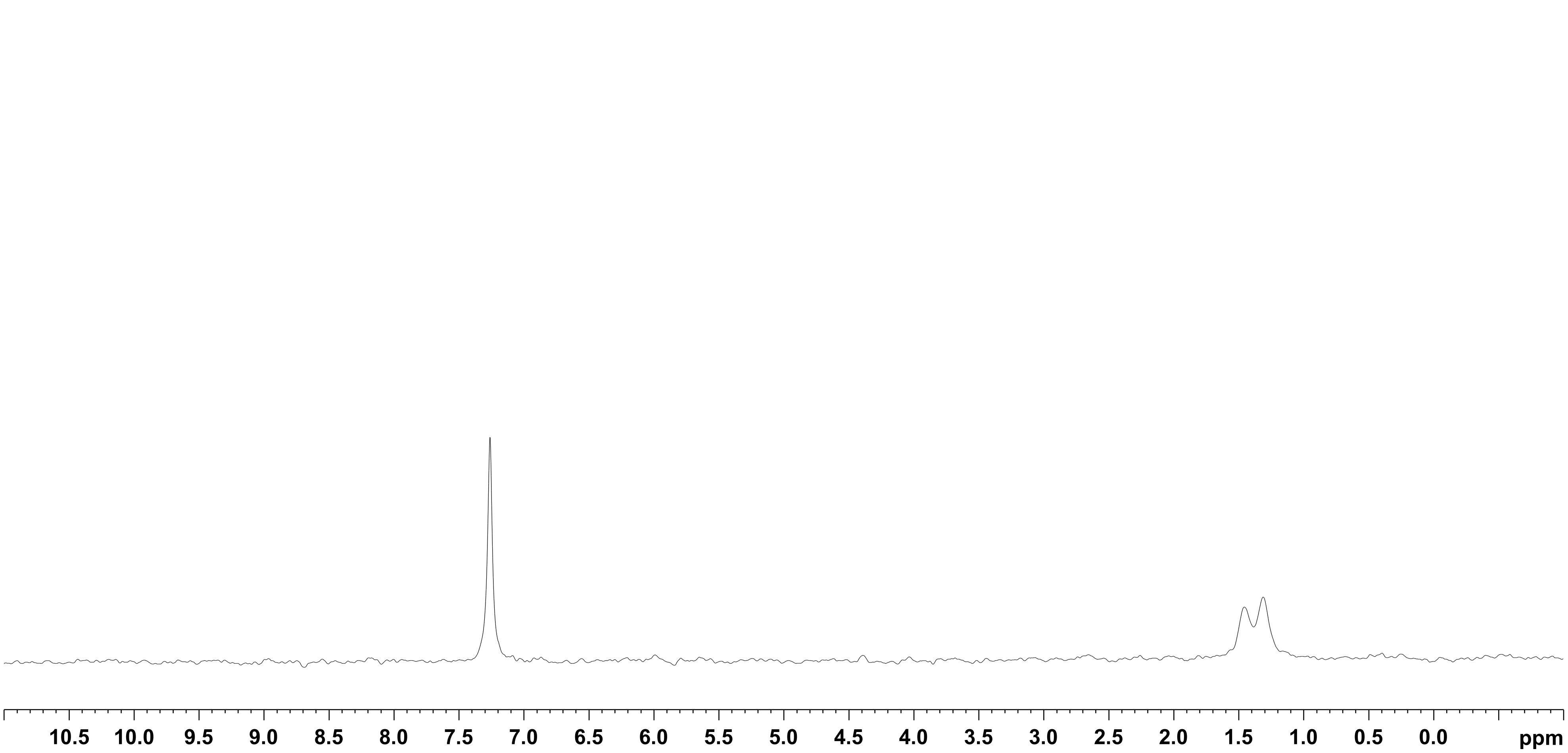

^{13}C NMR (100.6 MHz, $CDCl_3$)

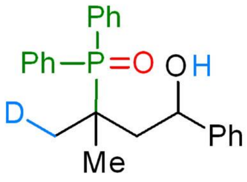

2j-D

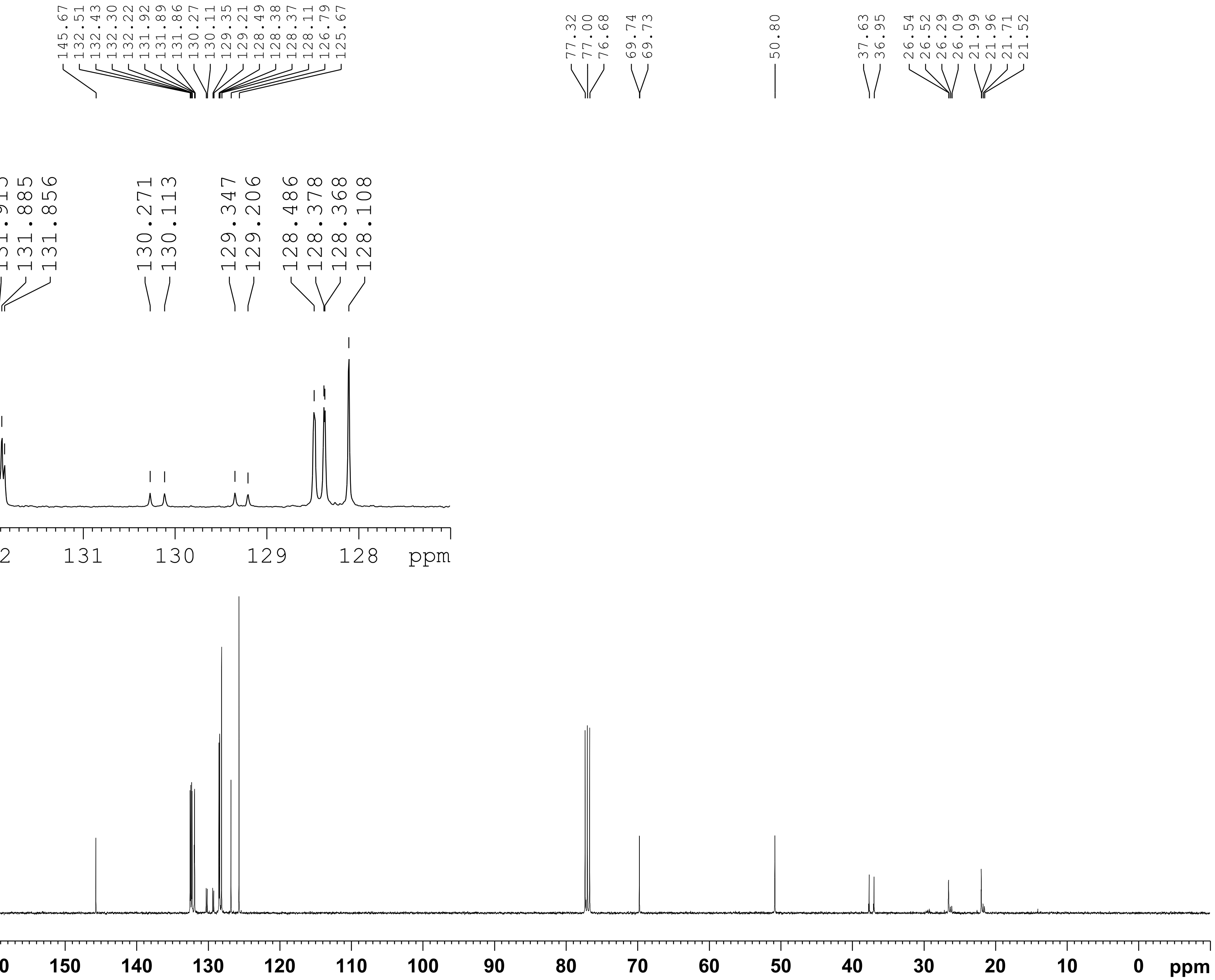

^{31}P NMR (162 MHz, CDCl$_3$)

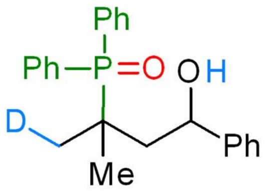

2j-D

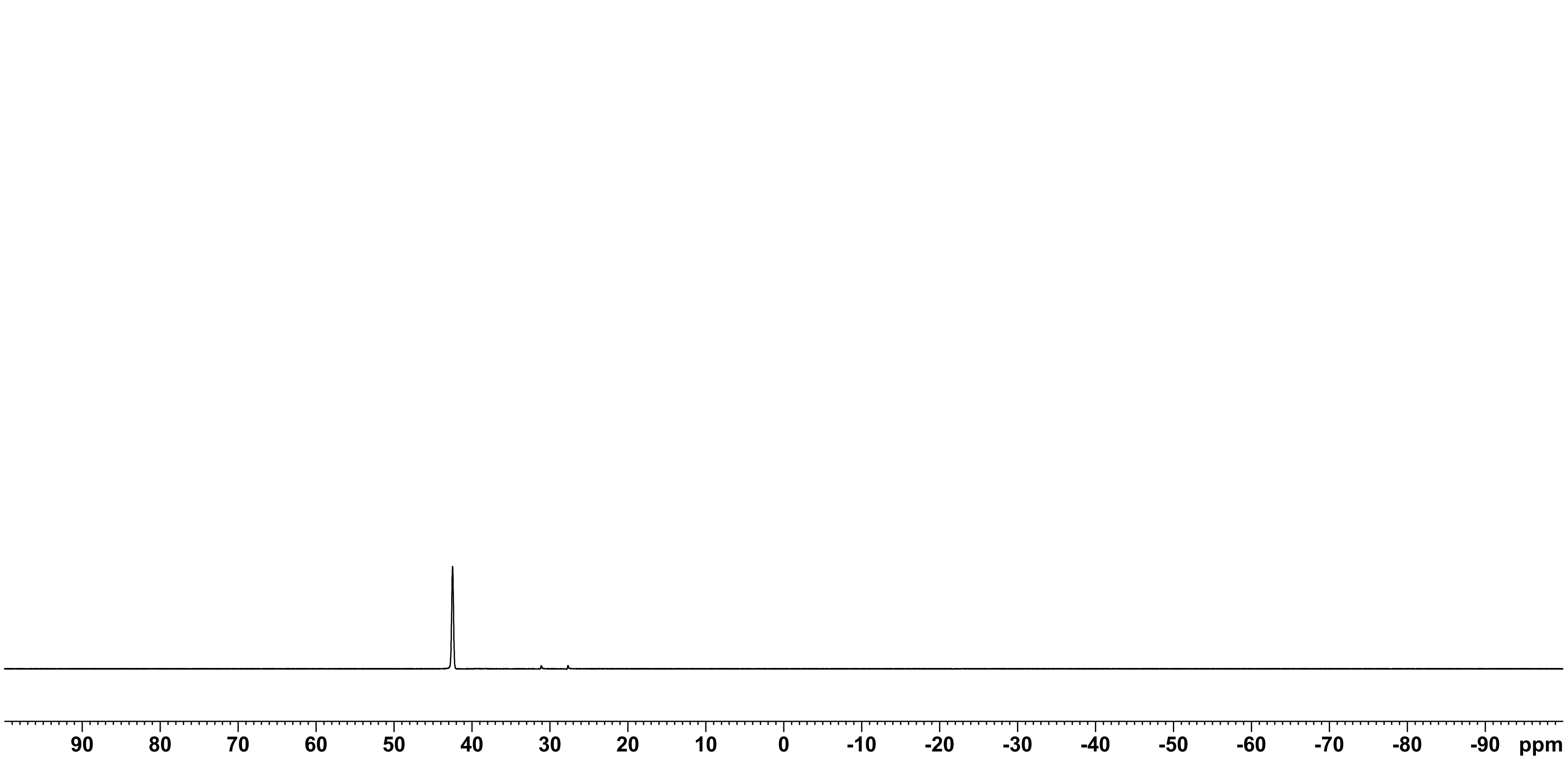

^{1}H NMR (400 MHz, $CDCl_3$)

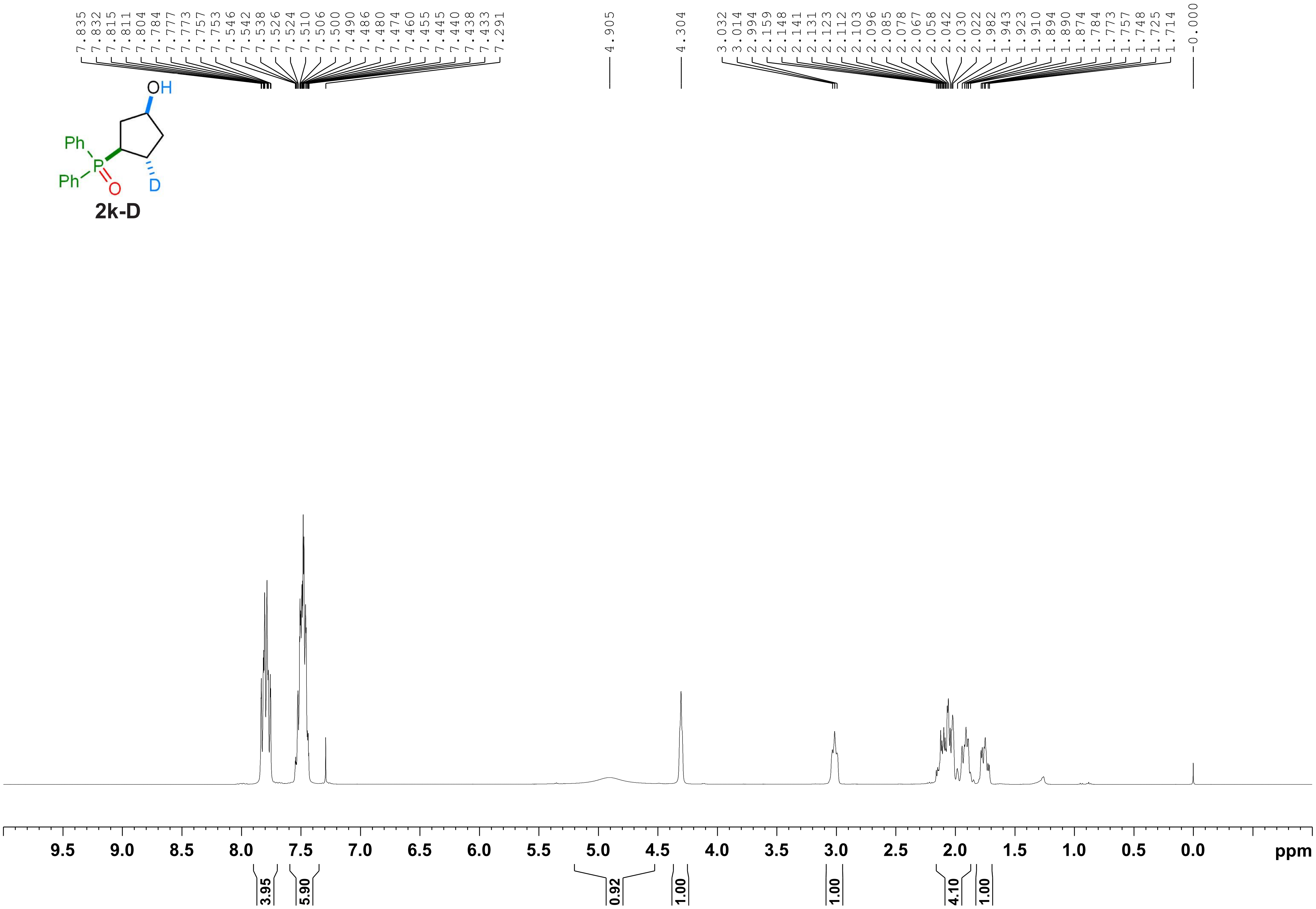

^{2}H NMR (92 MHz, $CHCl_3$)

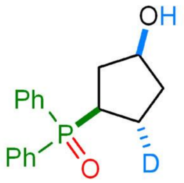

2k-D

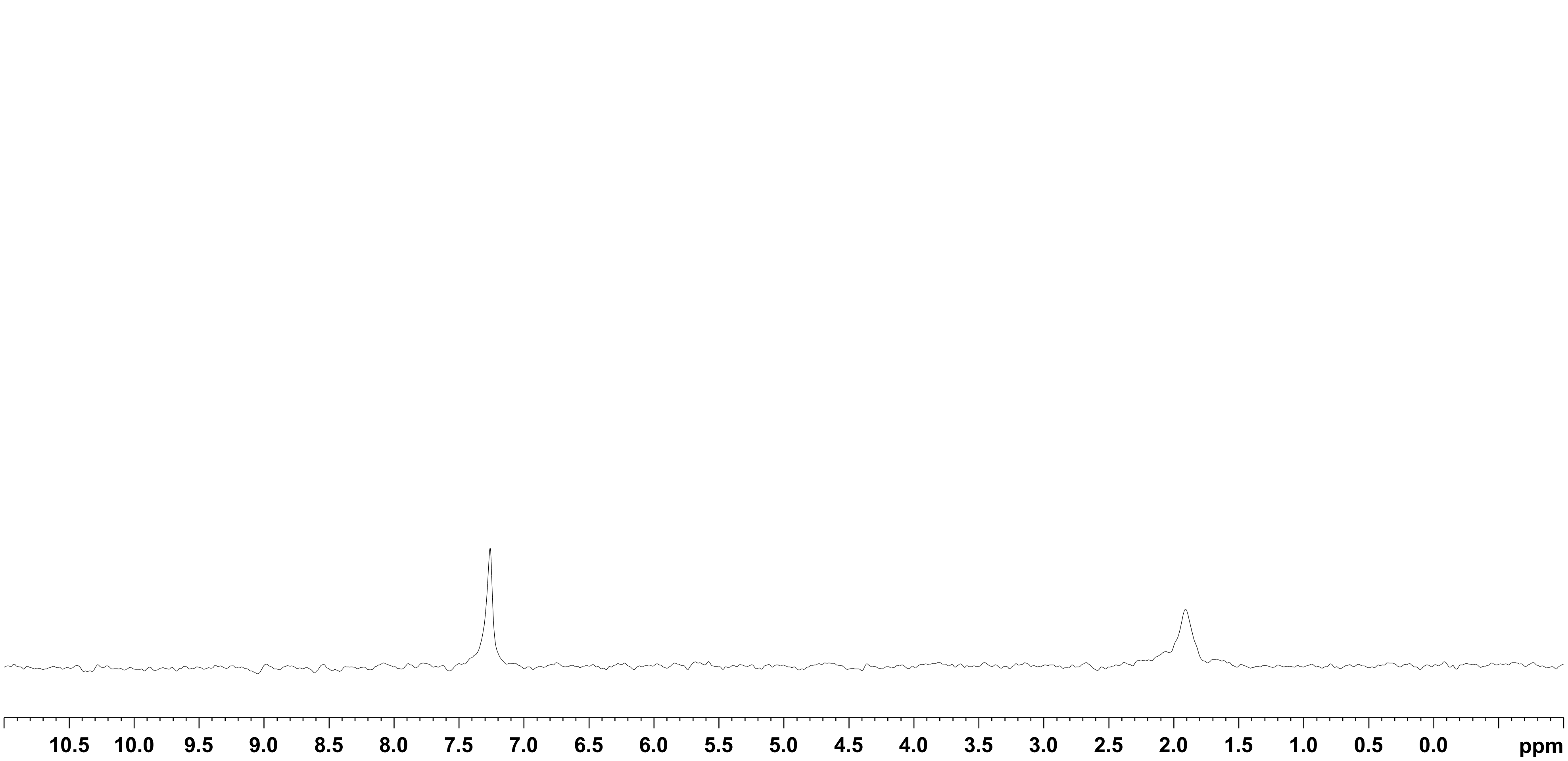

^{13}C NMR (100.6 MHz, $CDCl_3$)

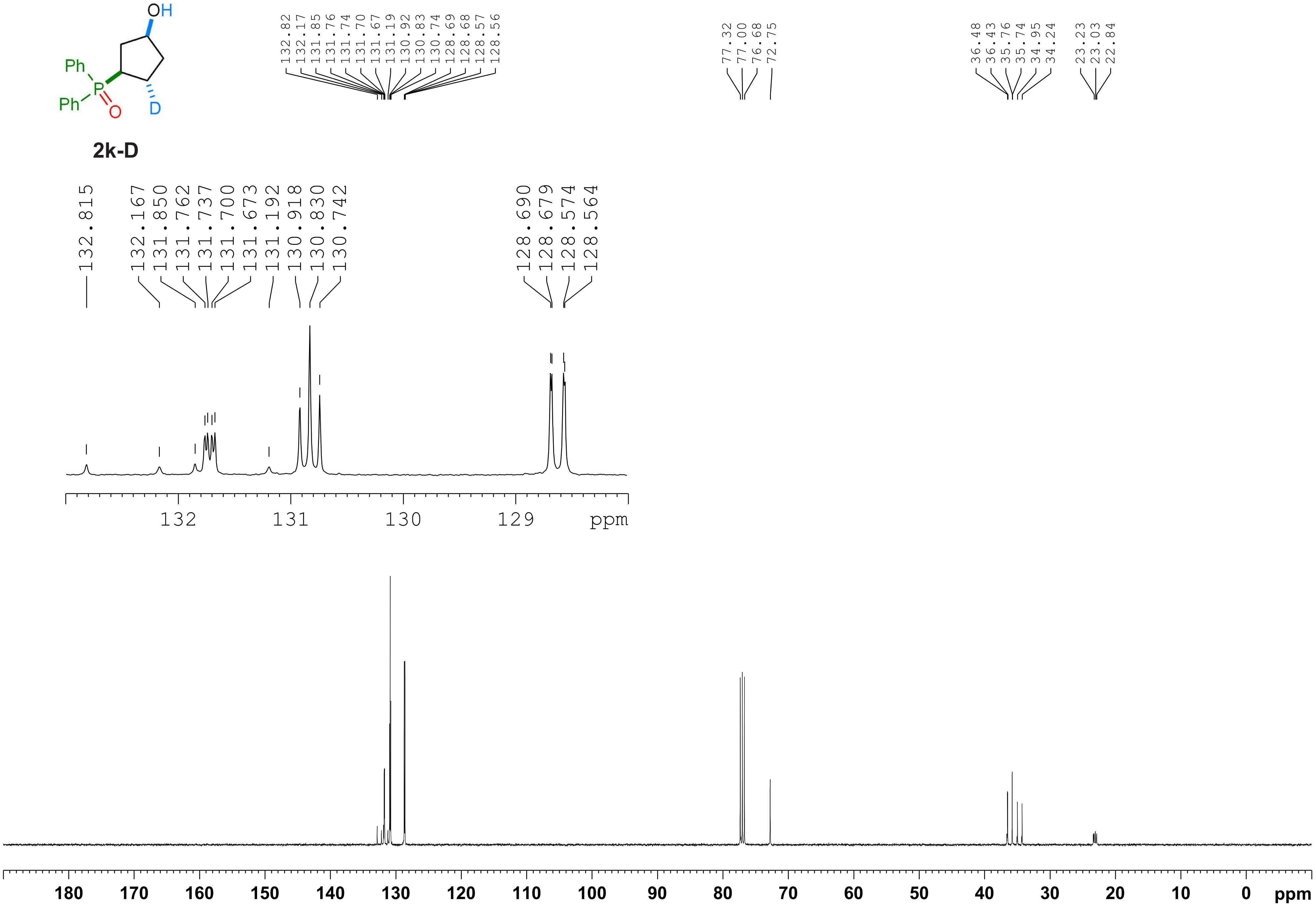

^{31}P NMR (162 MHz, $CDCl_3$)

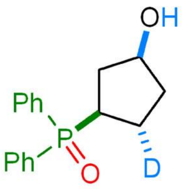

2k-D

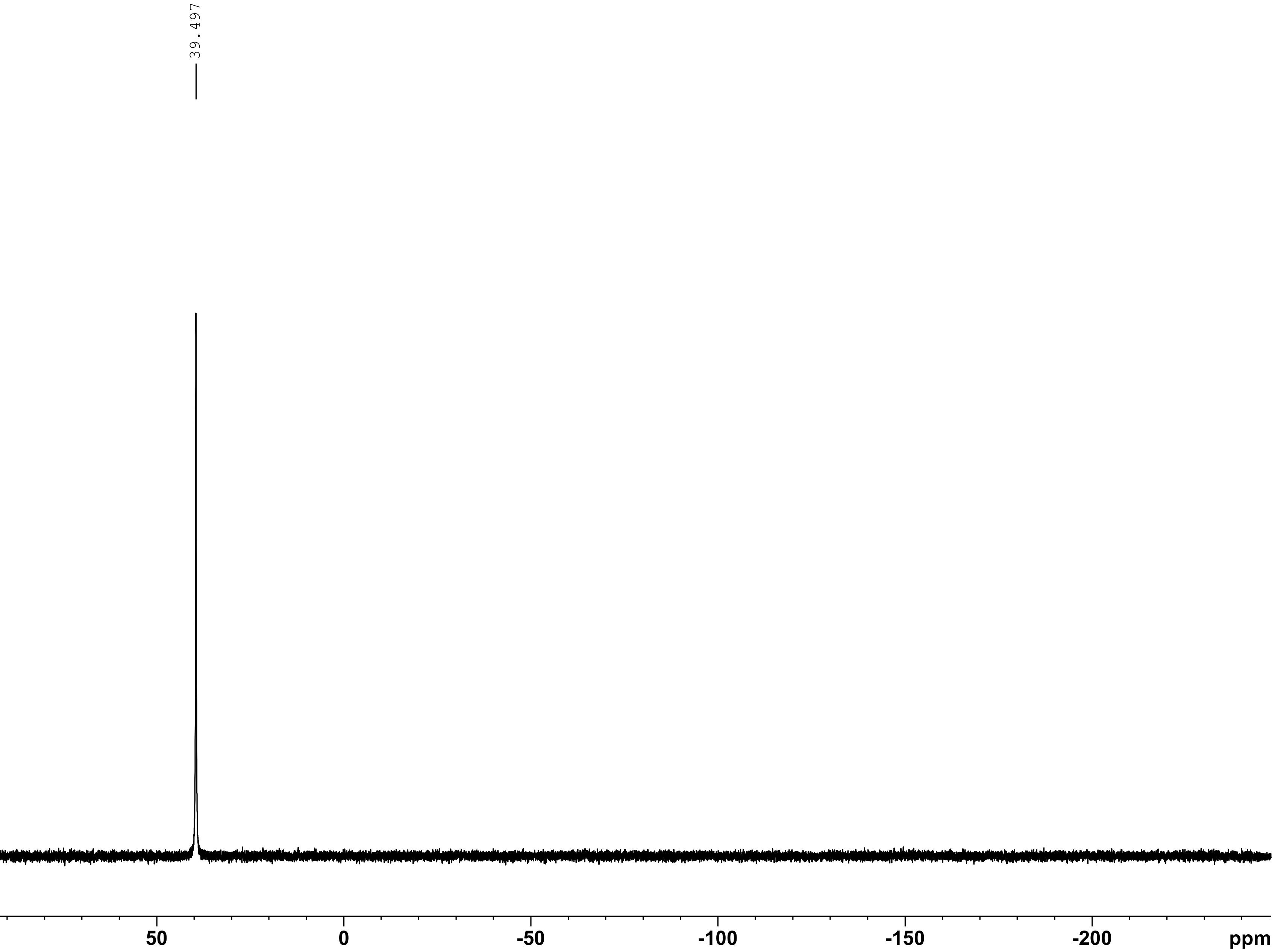

^{1}H NMR (400 MHz, $CDCl_3$)

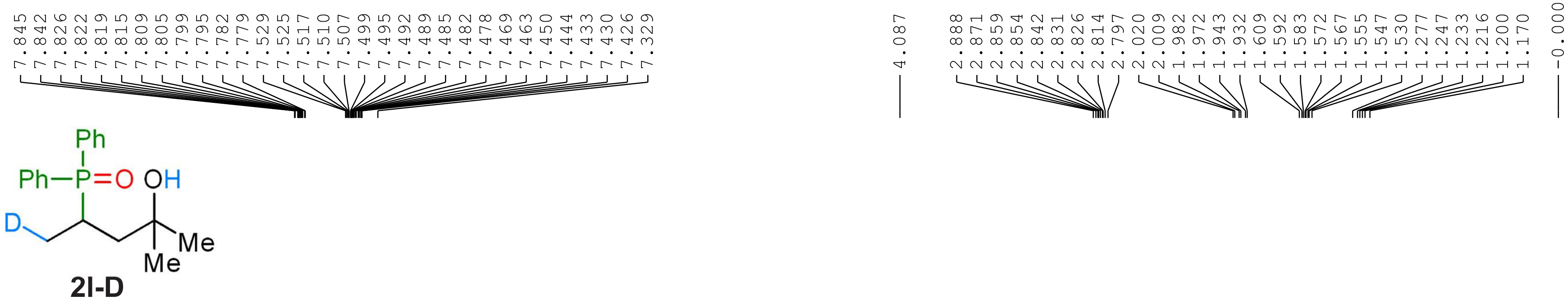

2l-D

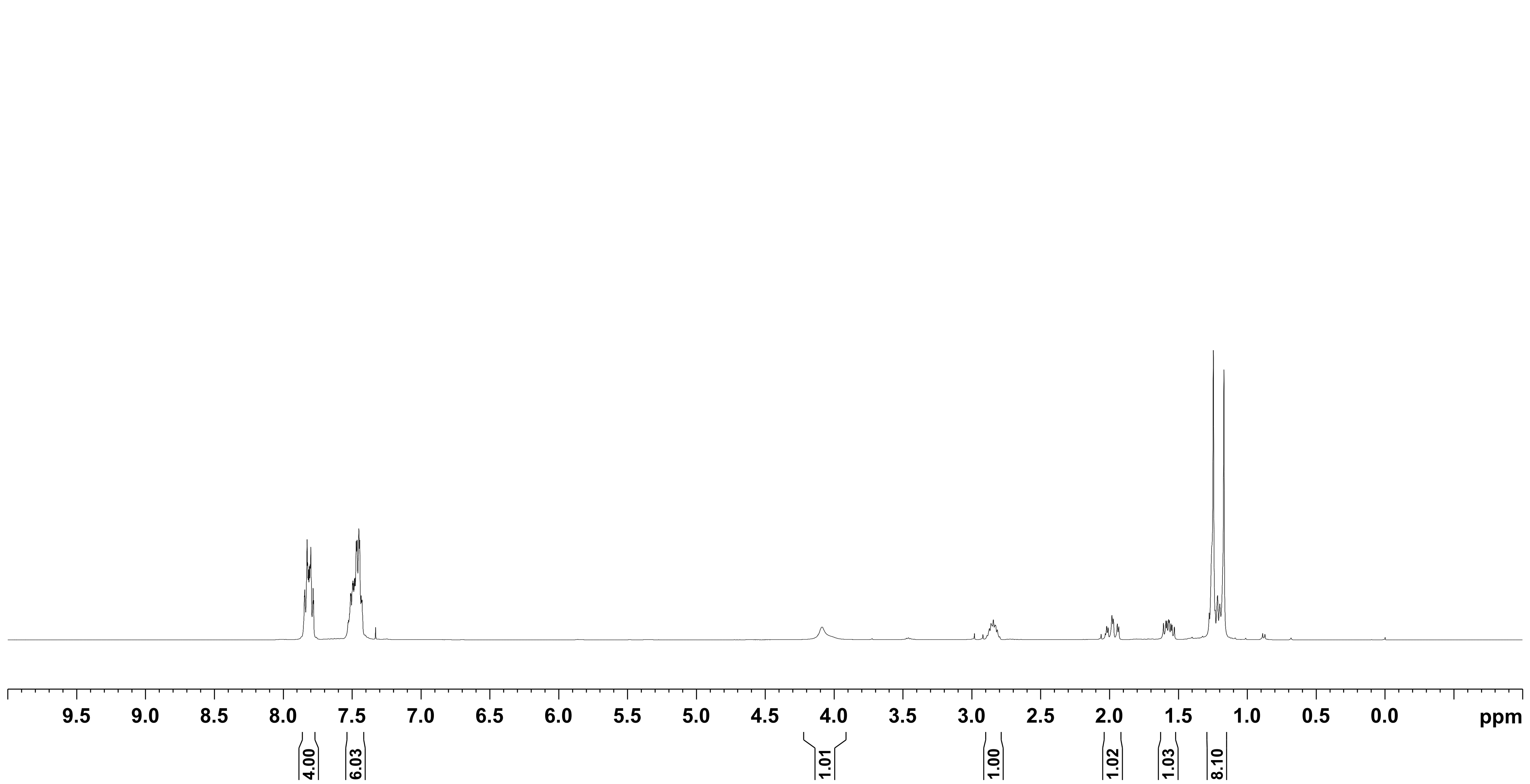

^2H NMR (92 MHz, $CHCl_3$)

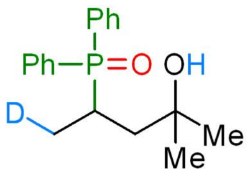

2l-D

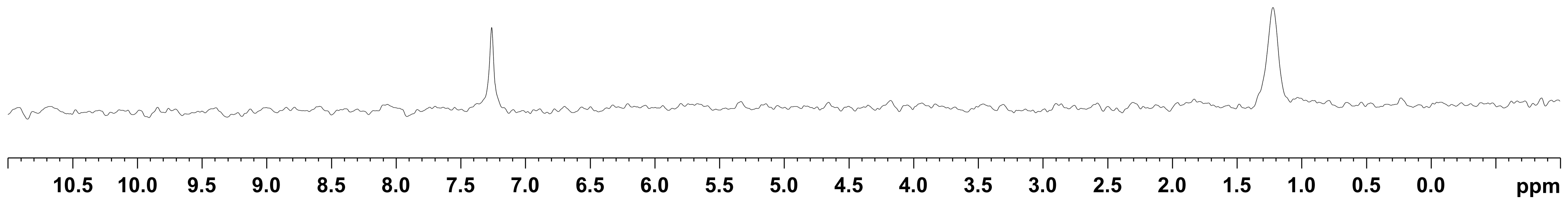

^{13}C NMR (100.6 MHz, $CDCl_3$)

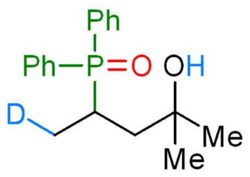

2l-D

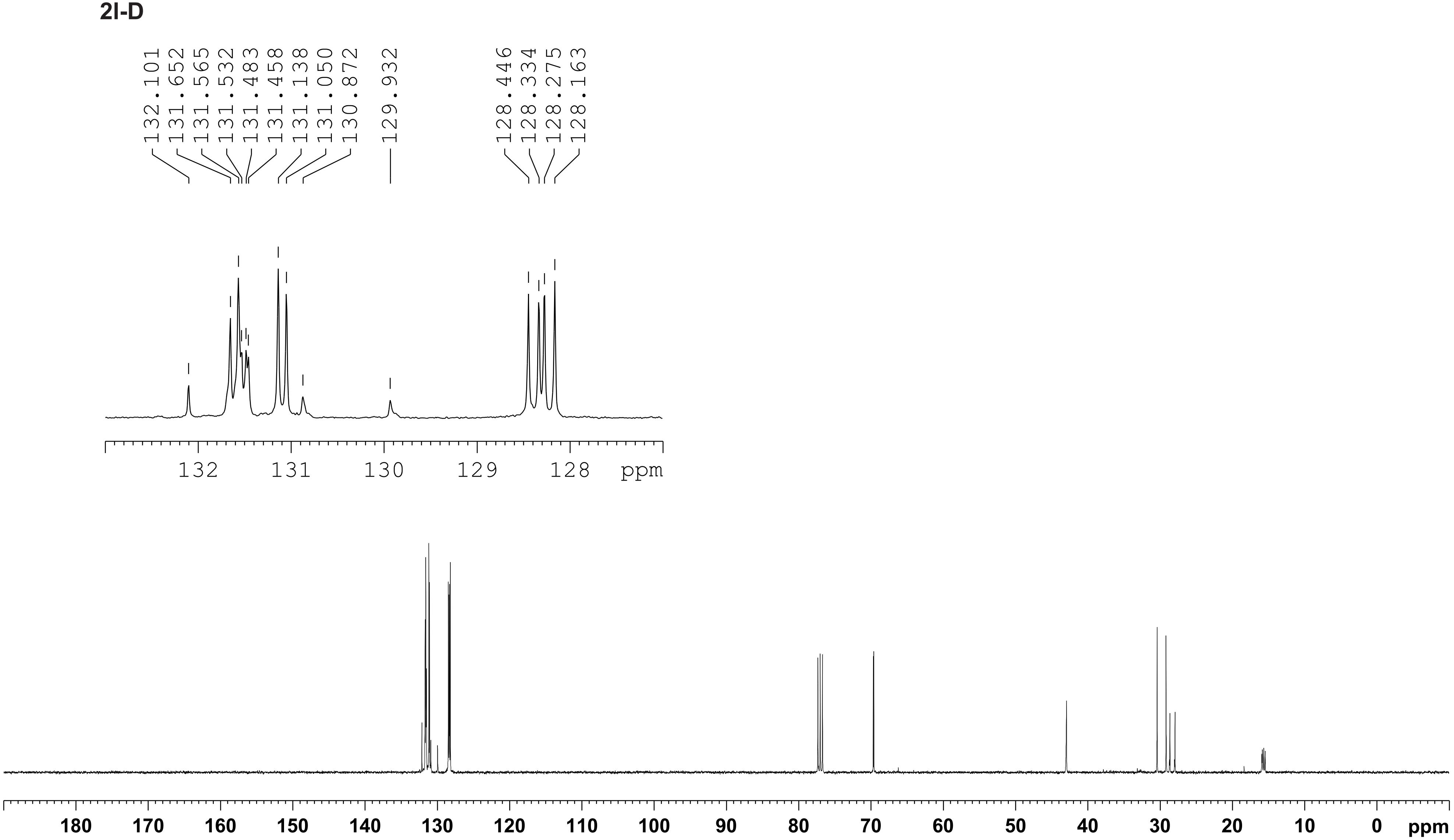

^{31}P NMR (162 MHz, $CDCl_3$)

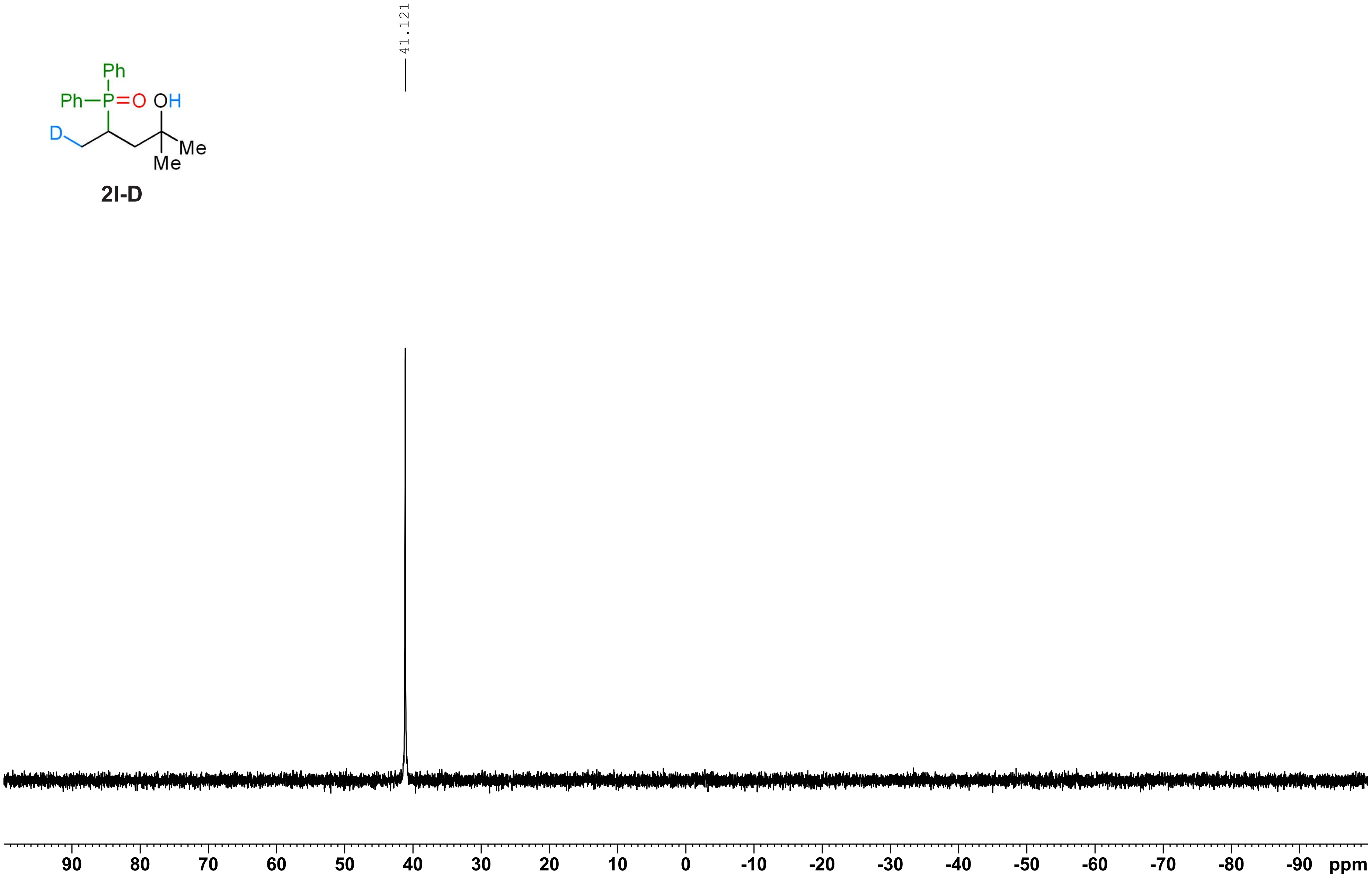

^{1}H NMR (400 MHz, $CDCl_3$)

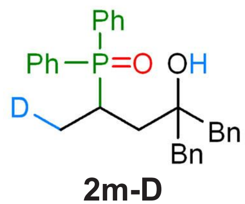

2m-D

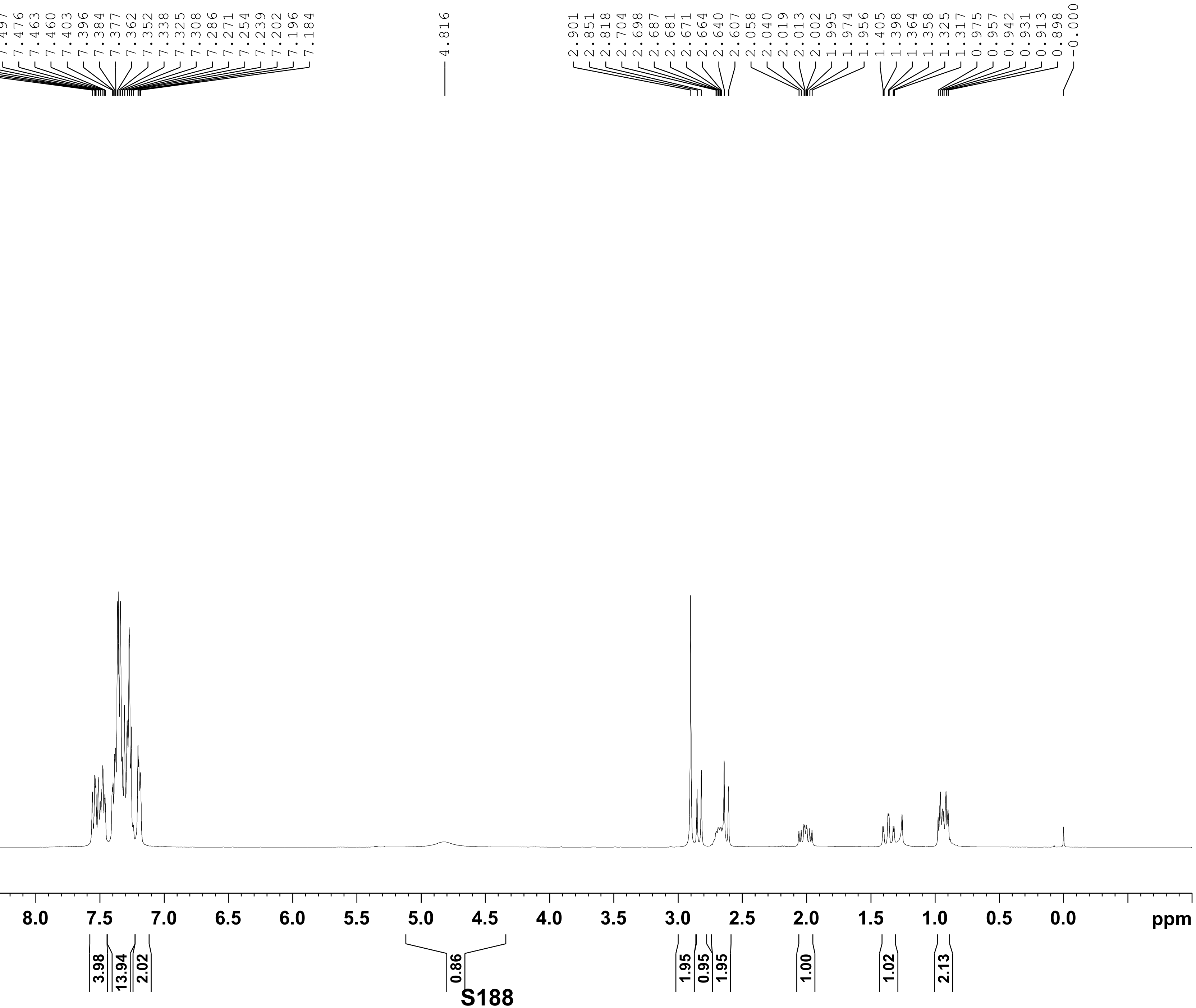

^{2}H NMR (92 MHz, $CHCl_3$)

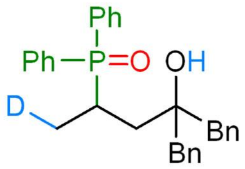

2m-D

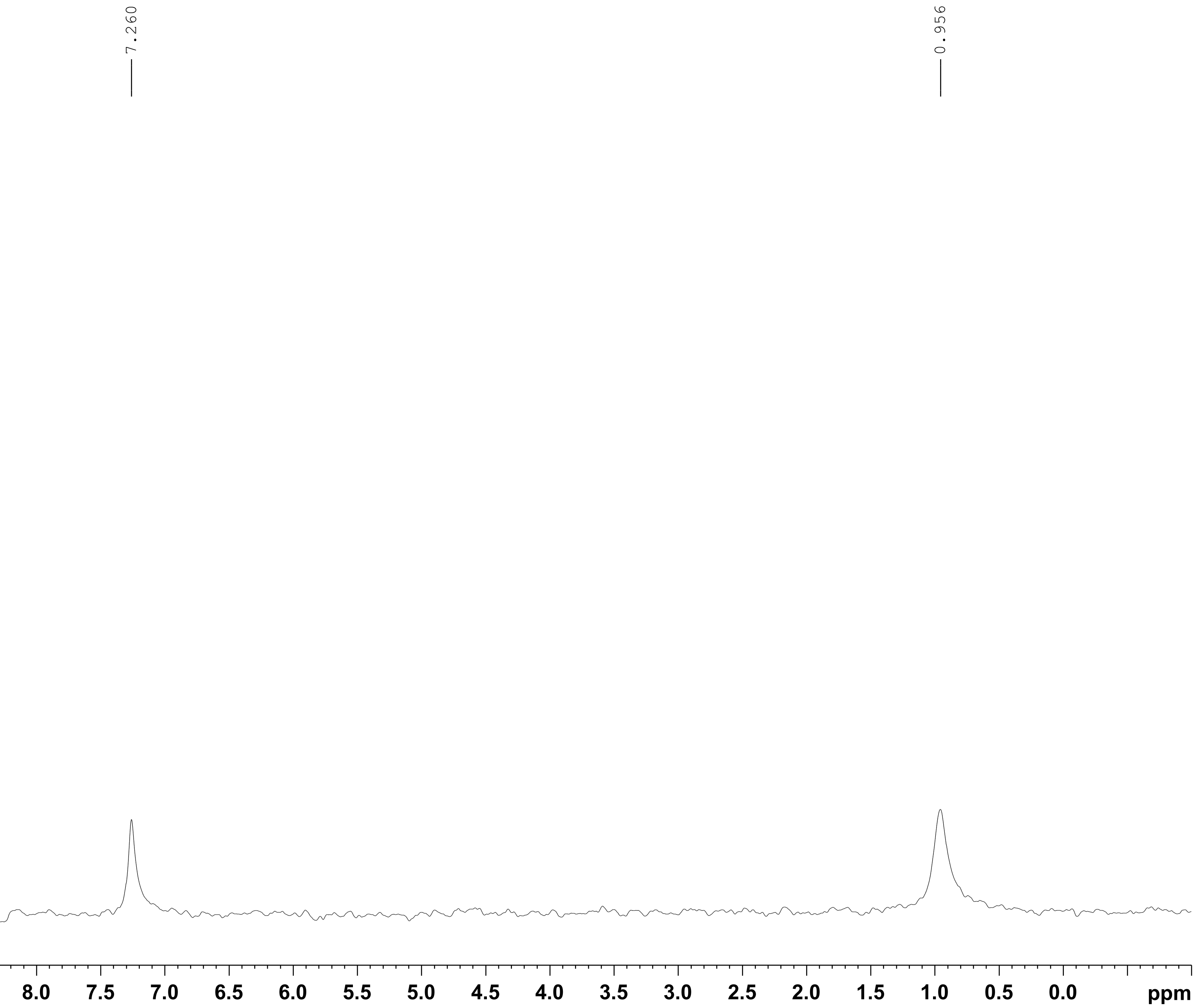

^{13}C NMR (100.6 MHz, $CDCl_3$)

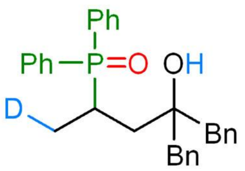

2m-D

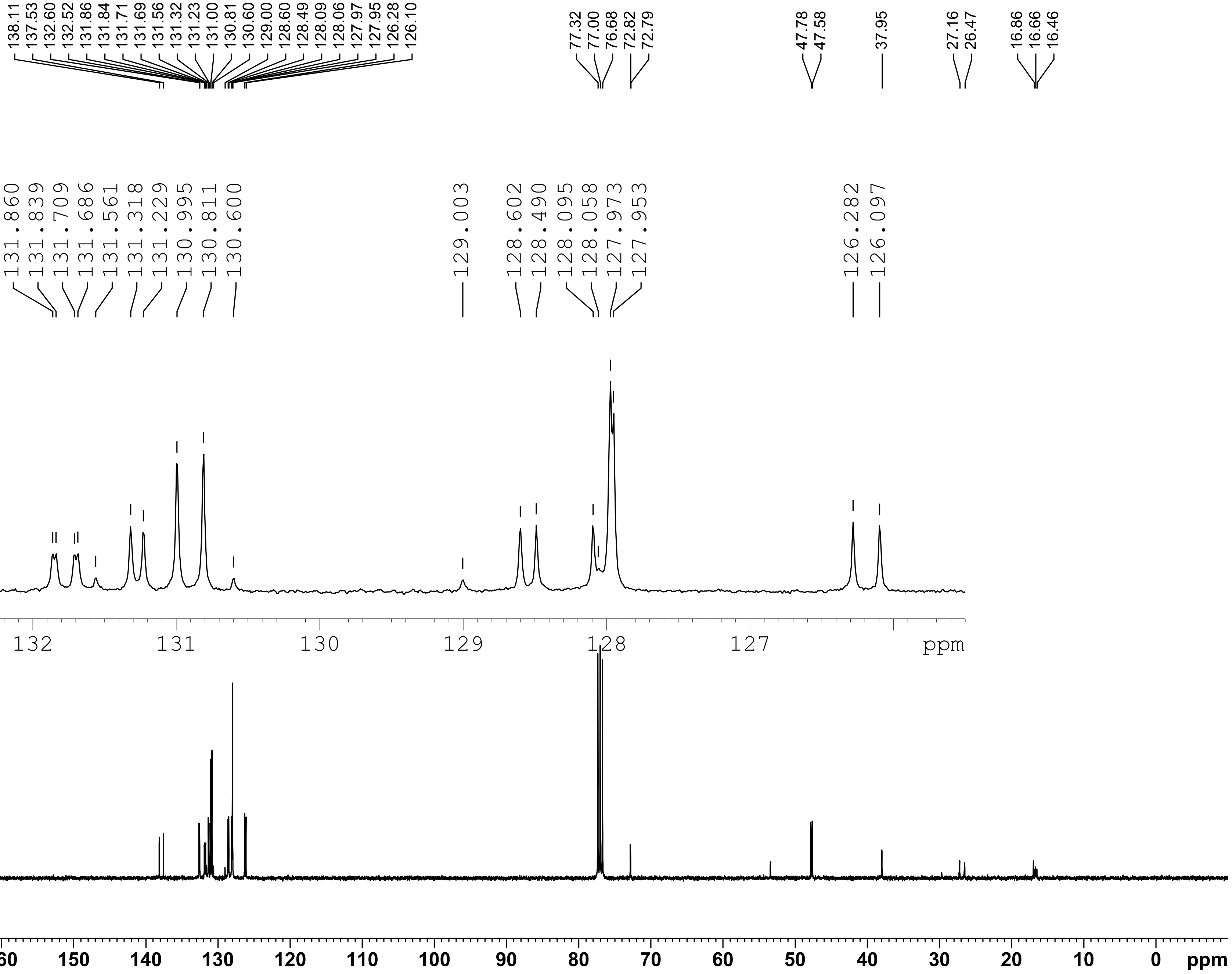

^{31}P NMR (162 MHz, $CDCl_3$)

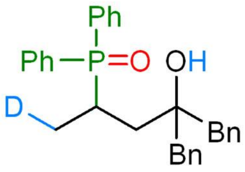

2m-D

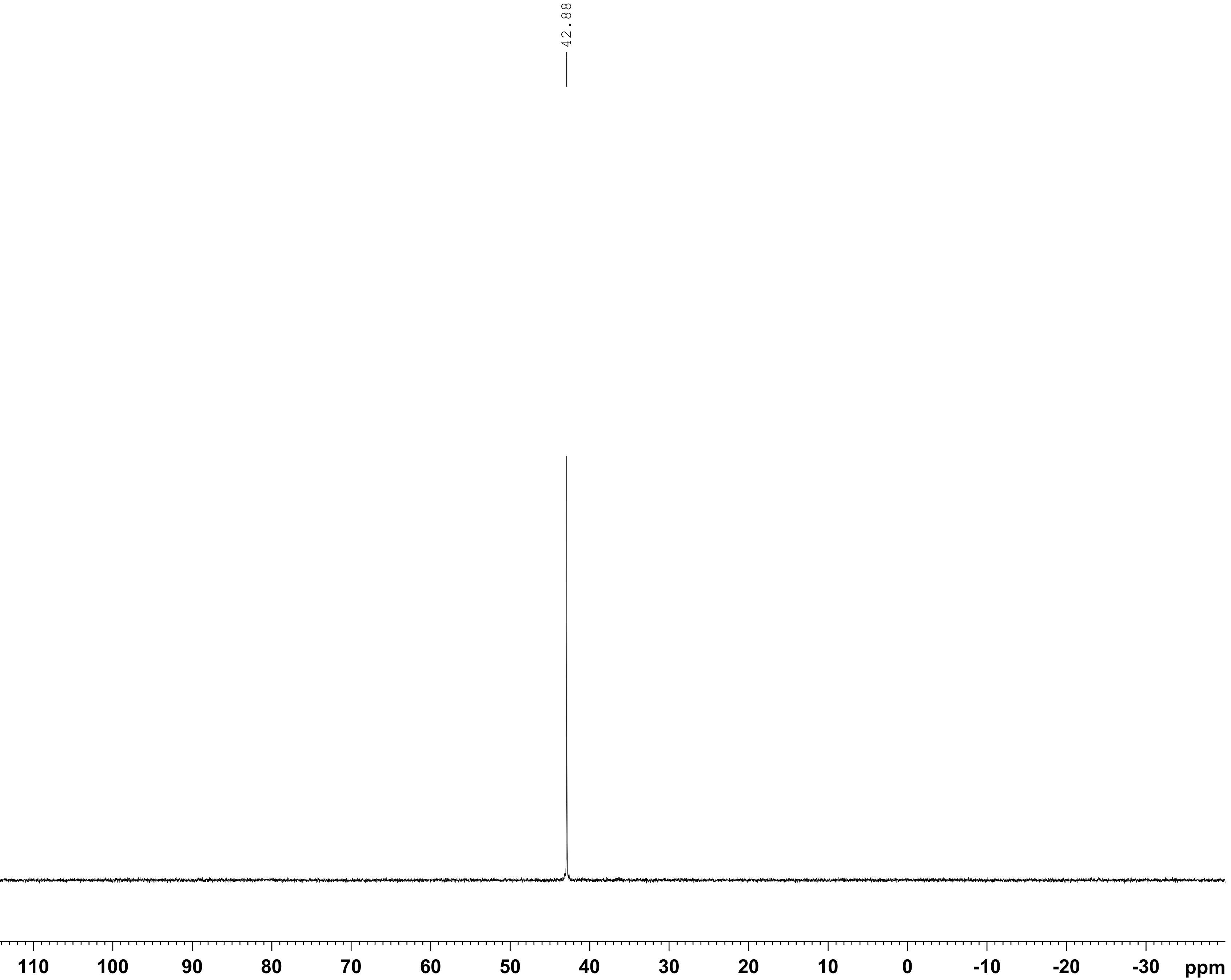

^{1}H NMR (400 MHz, $CDCl_3$)

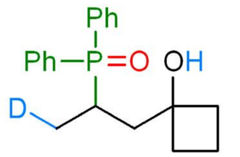

2p-D

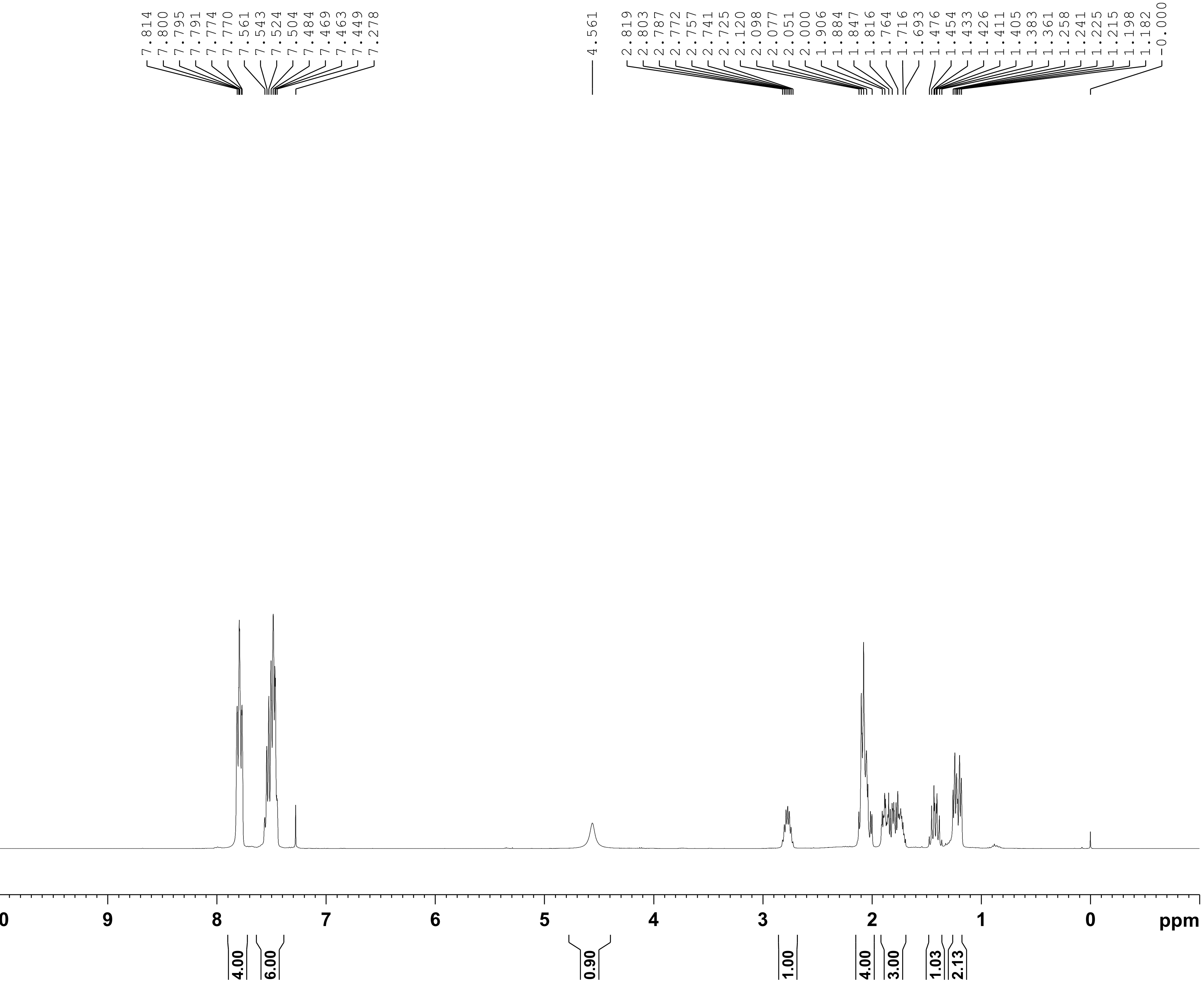

^{2}H NMR (92 MHz, $CHCl_3$)

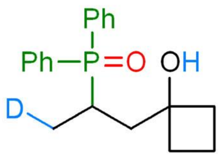

2p-D

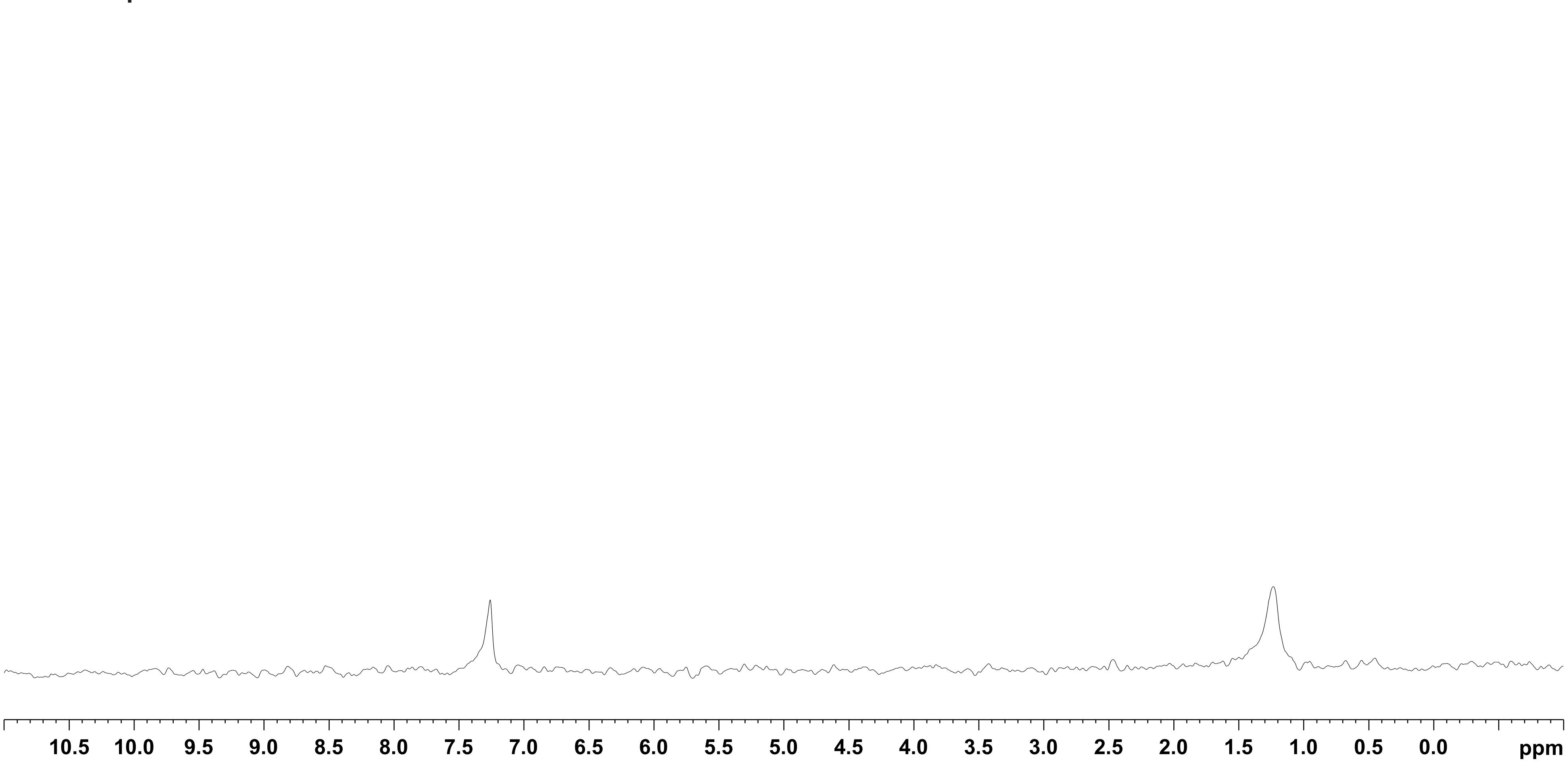

^{13}C NMR (100.6 MHz, $CDCl_3$)

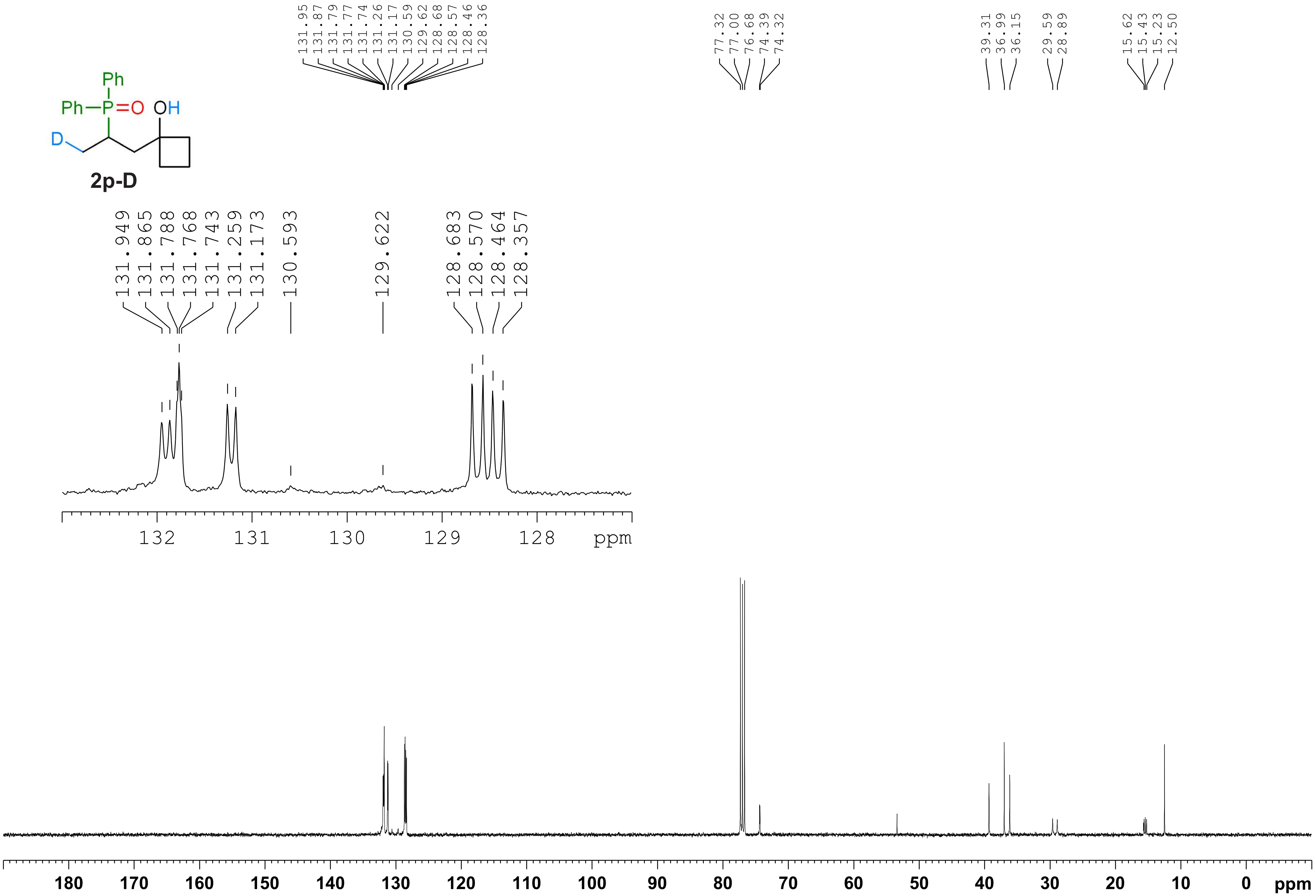

^{31}P NMR (162 MHz, $CDCl_3$)

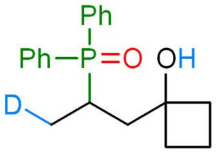

2p-D

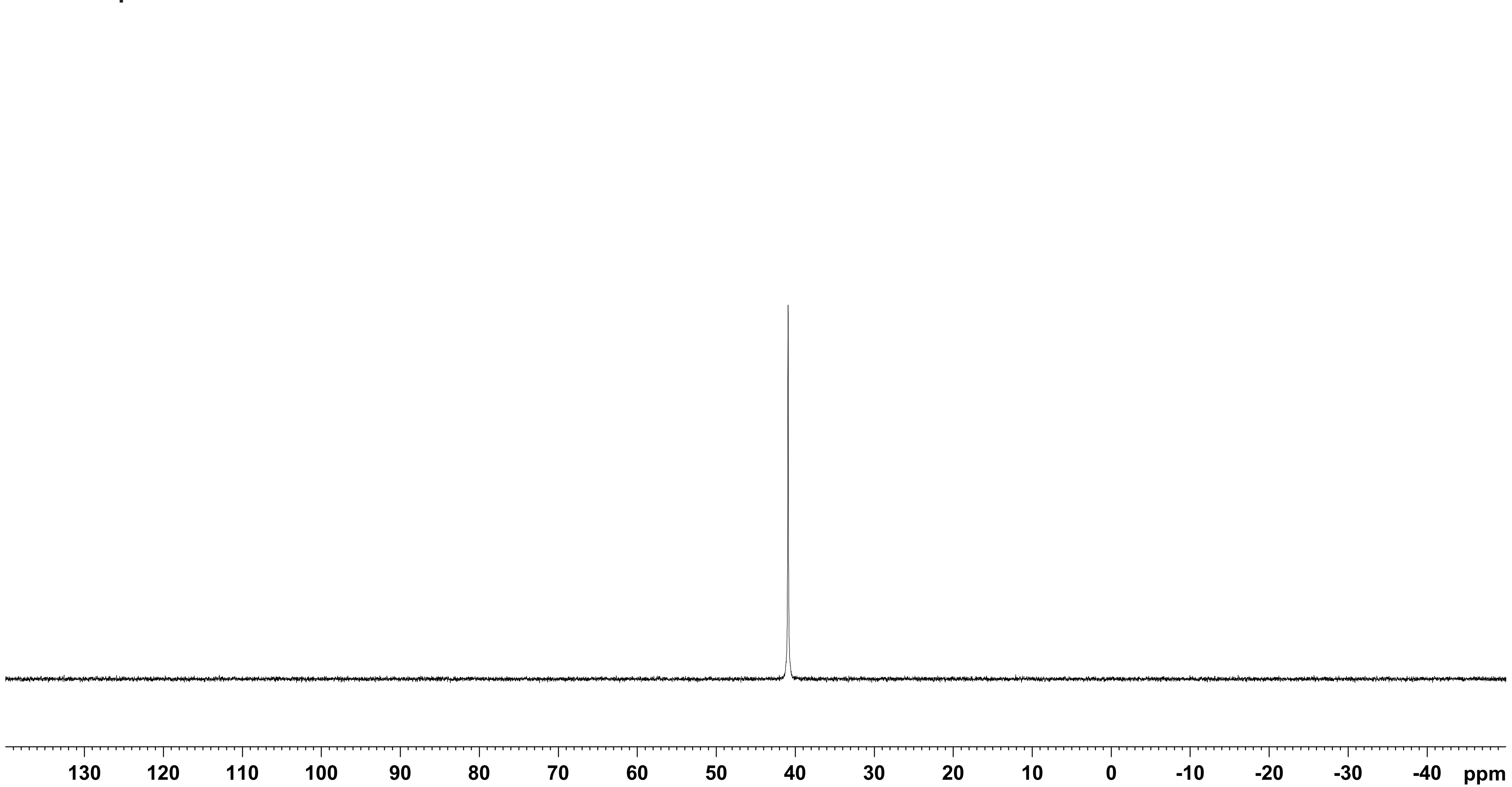

^{1}H NMR (400 MHz, $CDCl_3$)

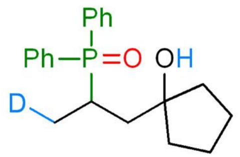

2q-D

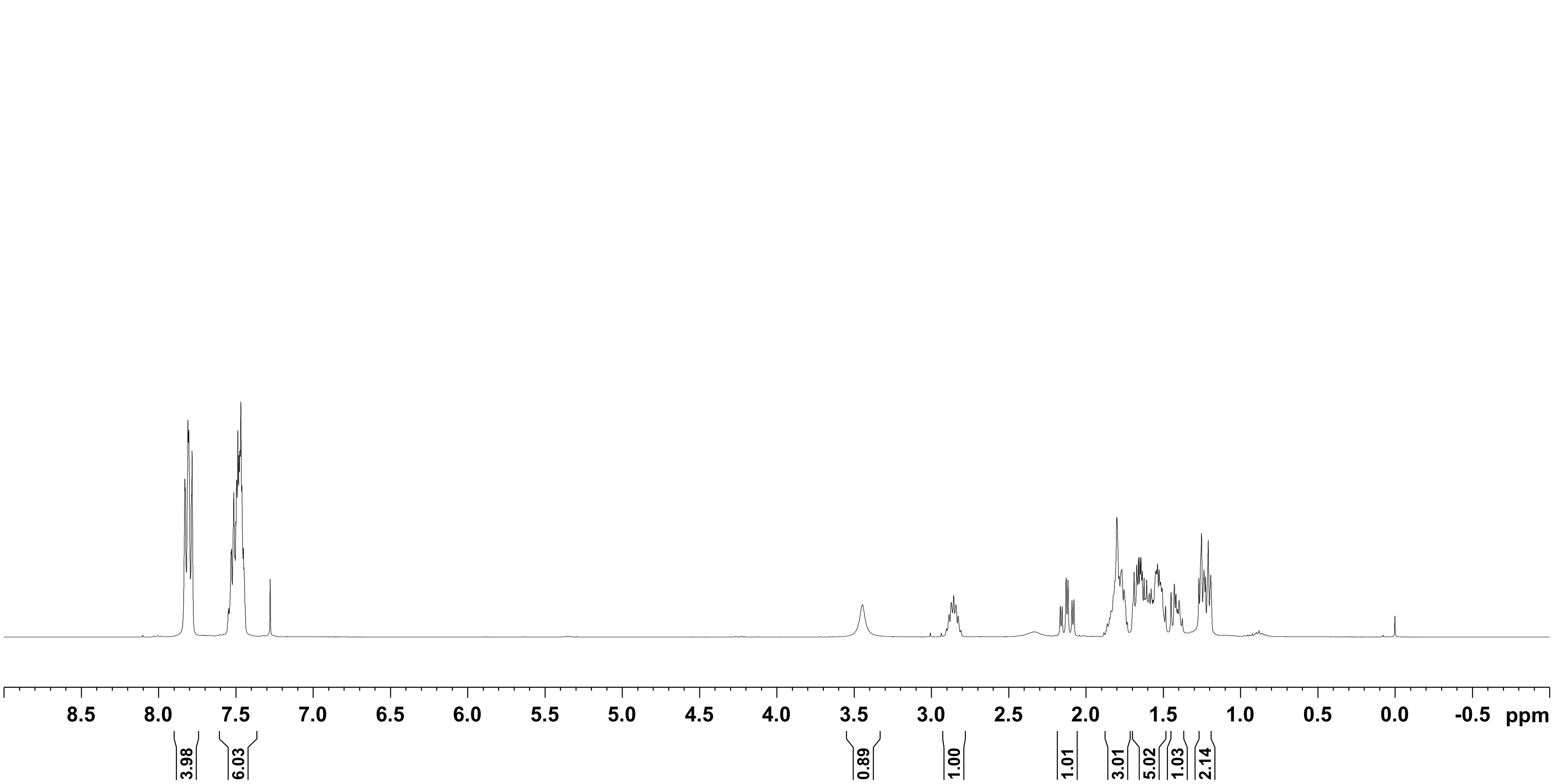

^{2}H NMR (92 MHz, $CHCl_3$)

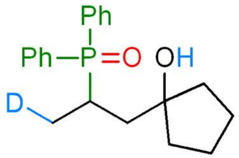

2q-D

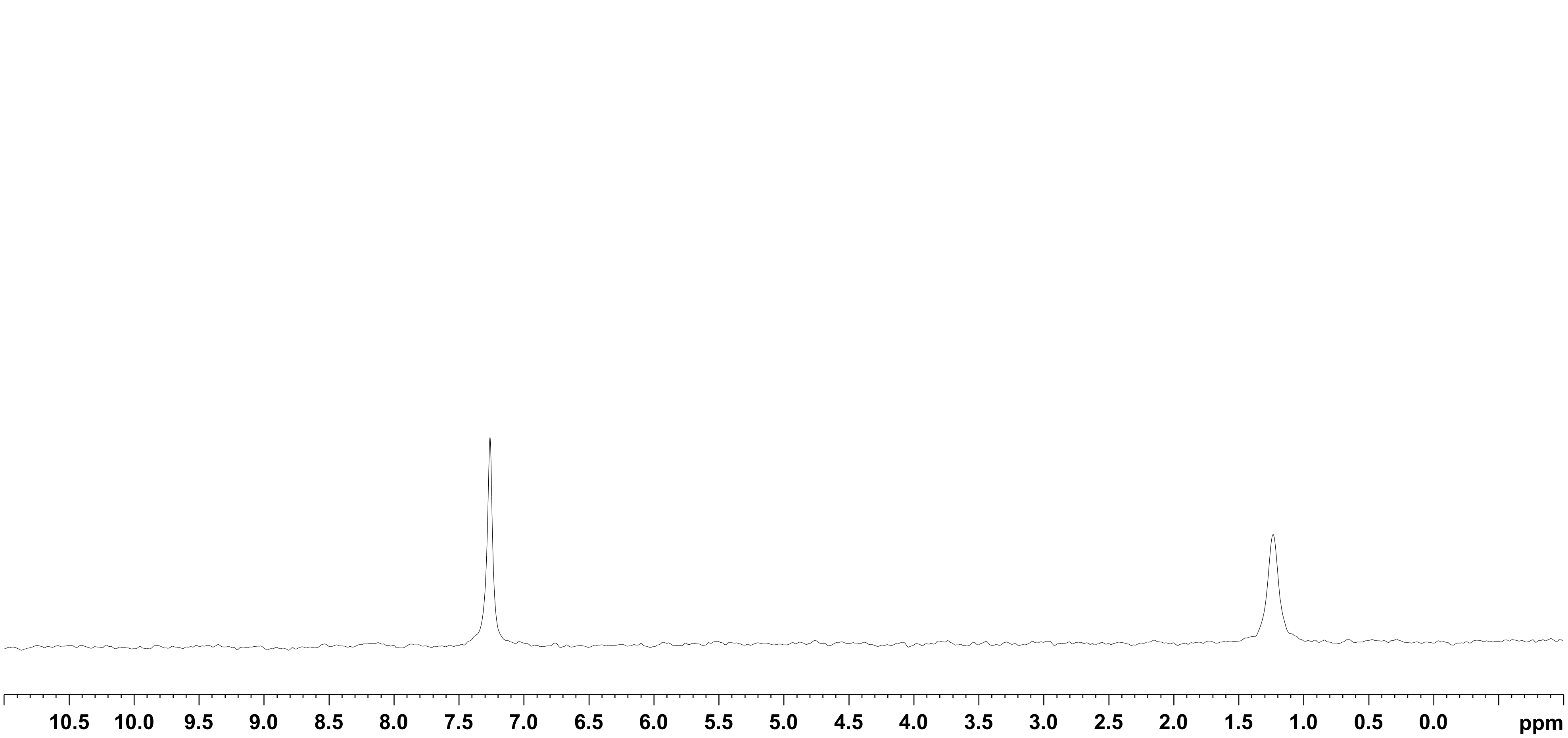

^{13}C NMR (100.6 MHz, $CDCl_3$)

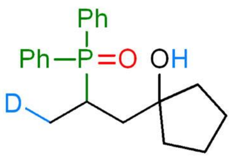

2q-D

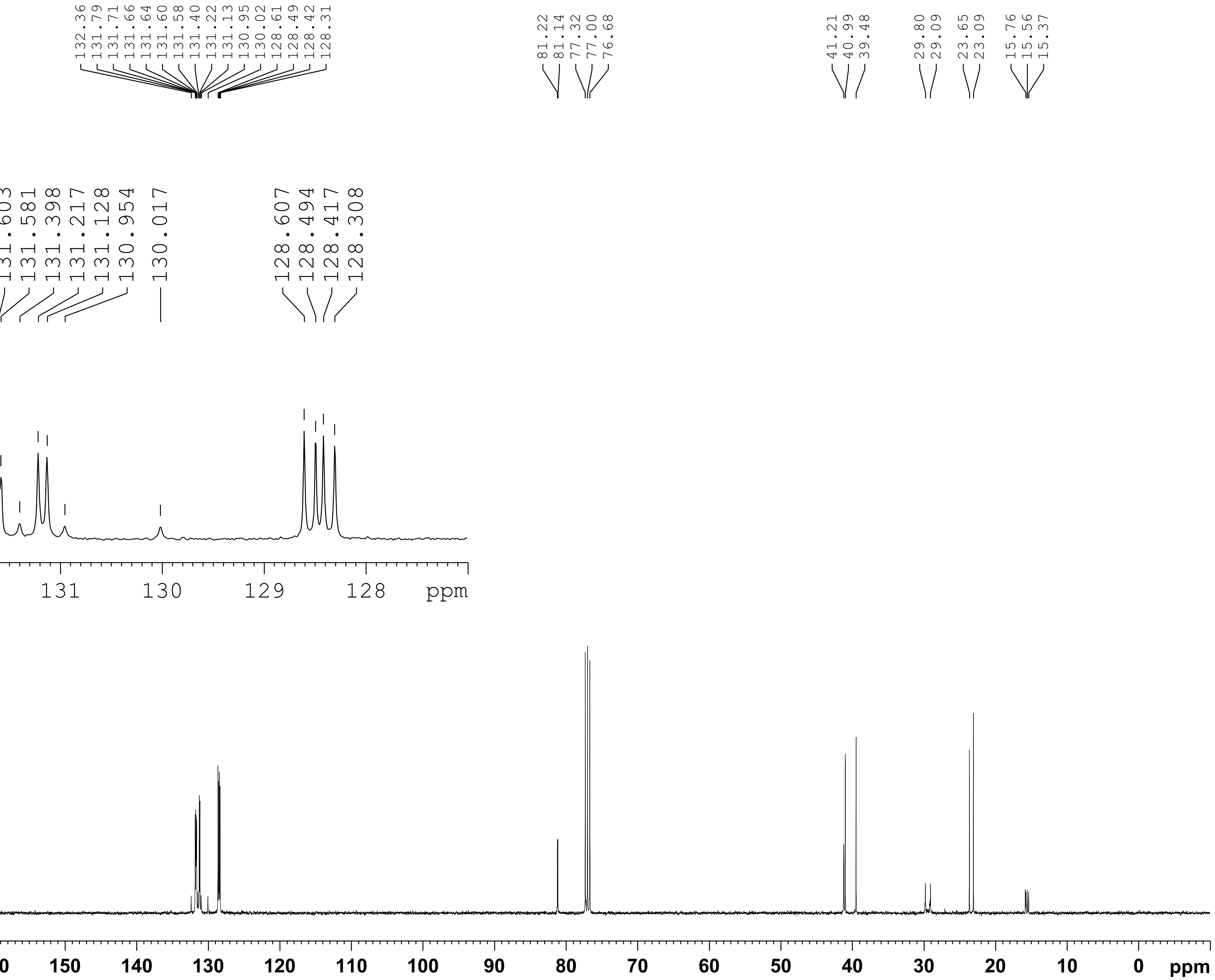

^{31}P NMR (162 MHz, $CDCl_3$)

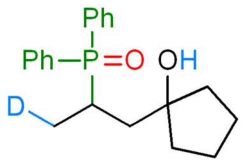

2q-D

40.71

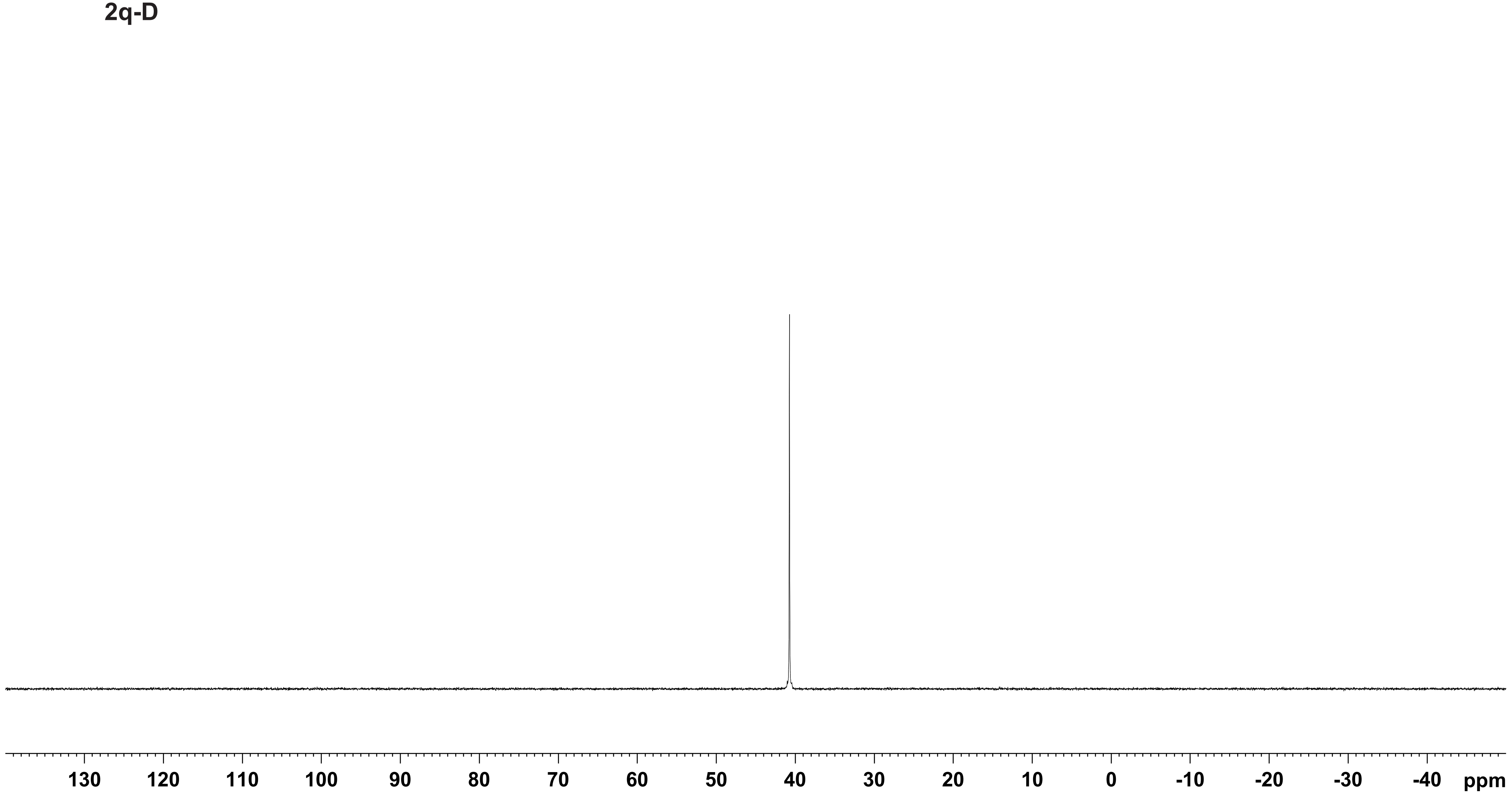

^{1}H NMR (400 MHz, $CDCl_3$)

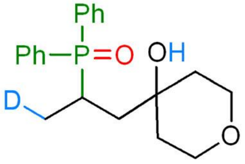

2aa-D

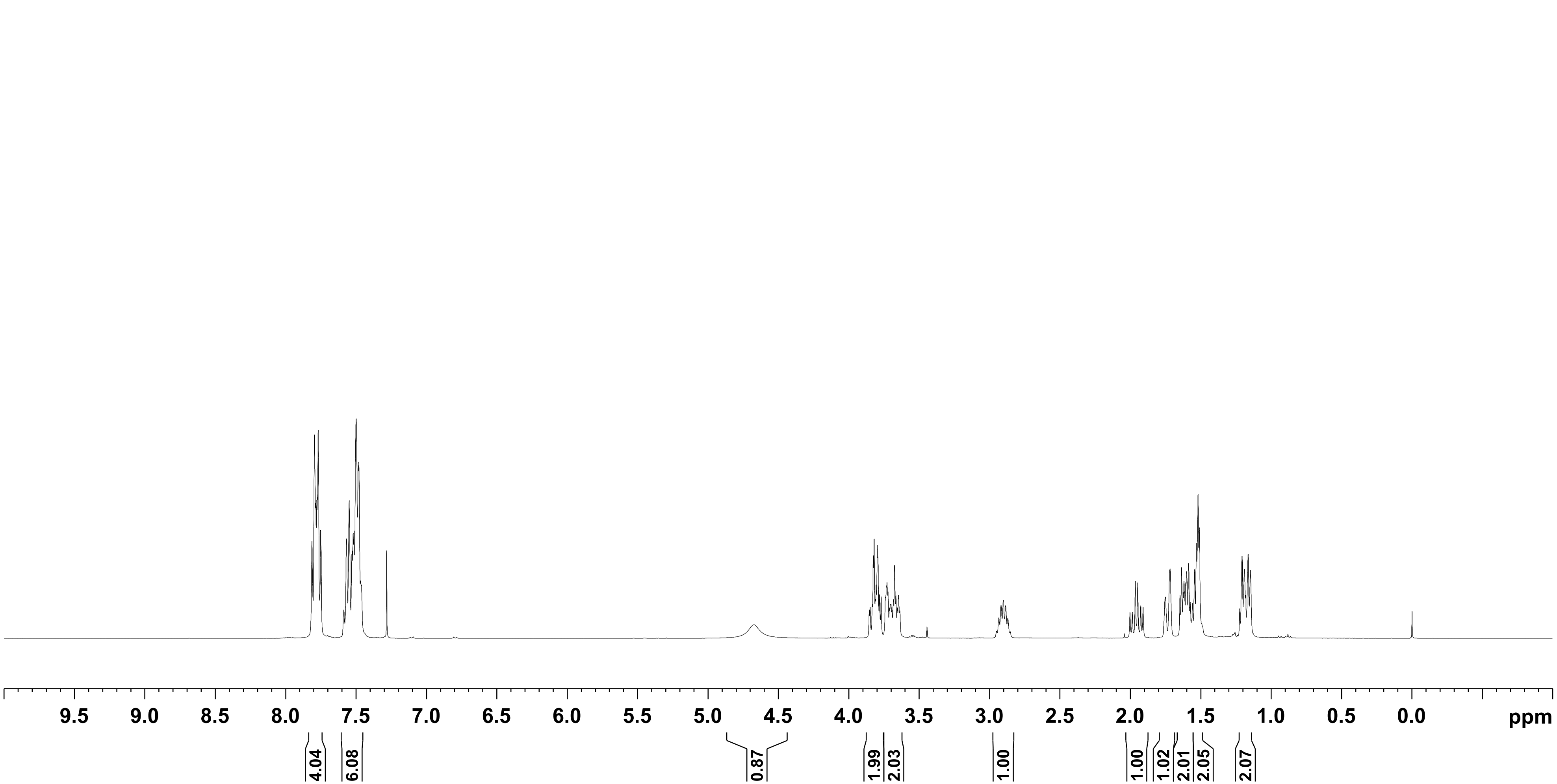

^2H NMR (92 MHz, $CHCl_3$)

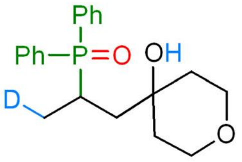

2aa-D

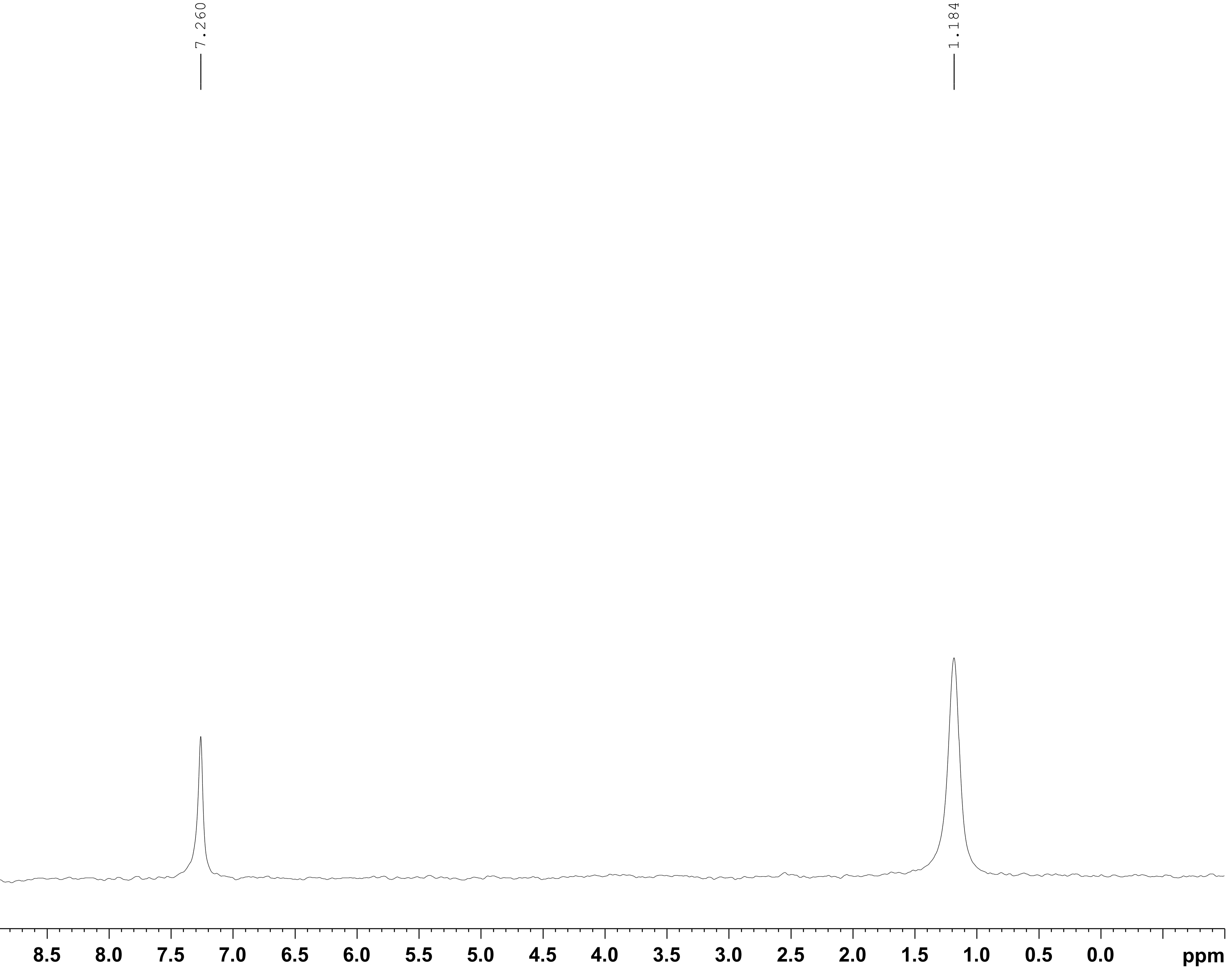

^{13}C NMR (100.6 MHz, $CDCl_3$)

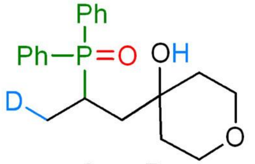

2aa-D

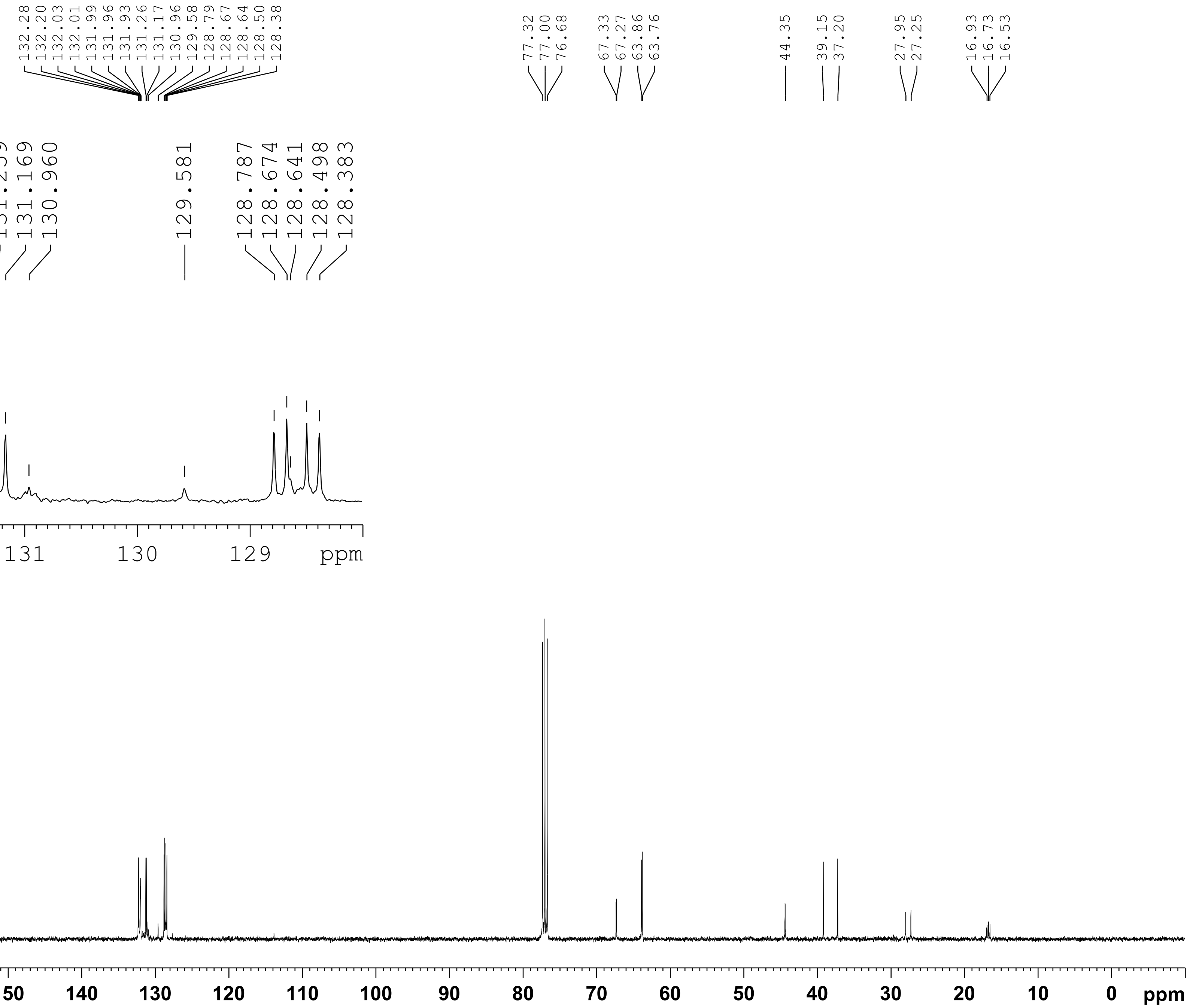

^{31}P NMR (162 MHz, $CDCl_3$)

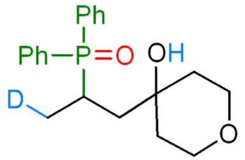

2aa-D

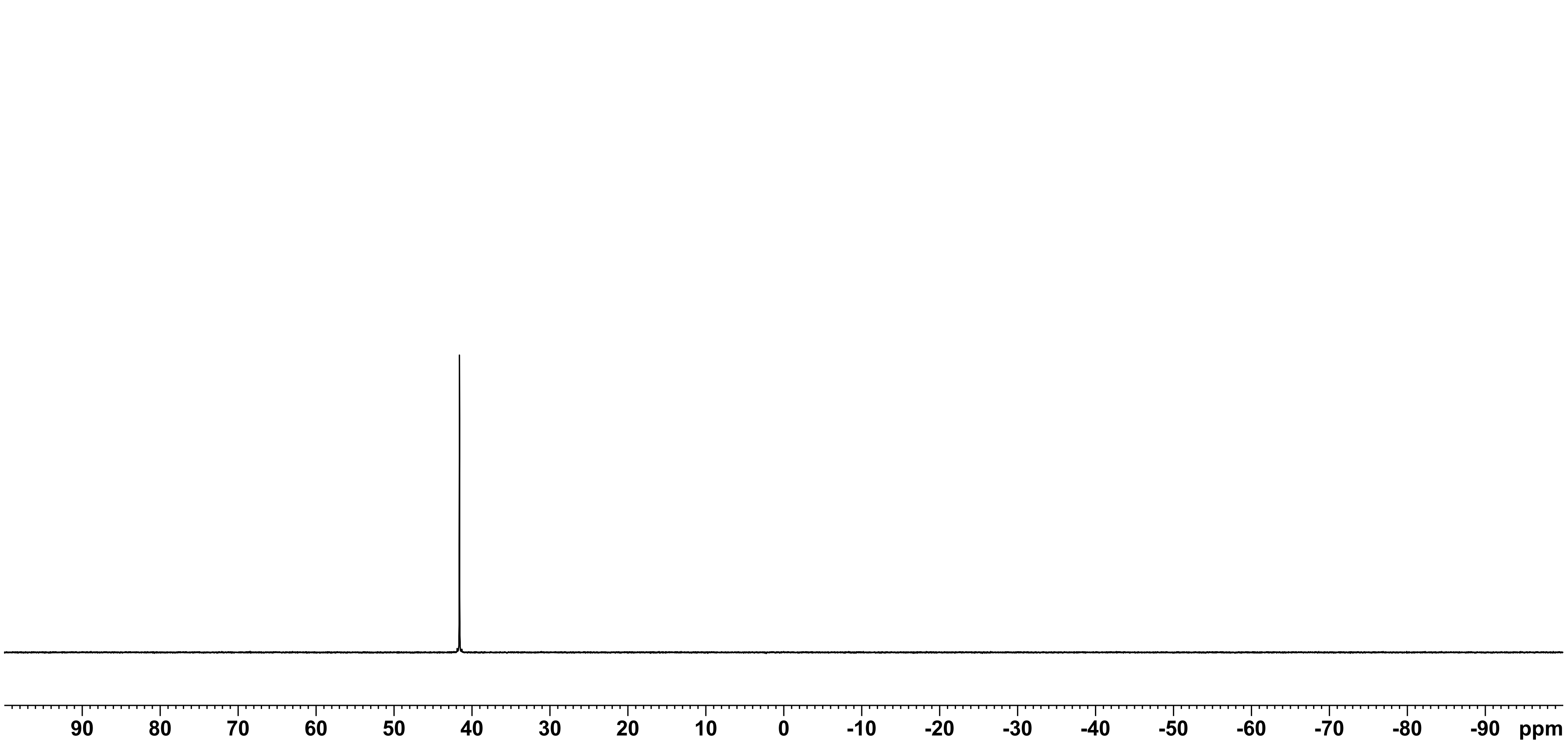

^{1}H NMR (400 MHz, $CDCl_3$)

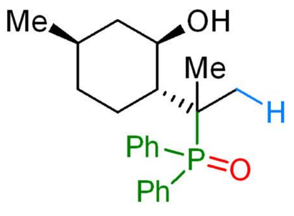

2al

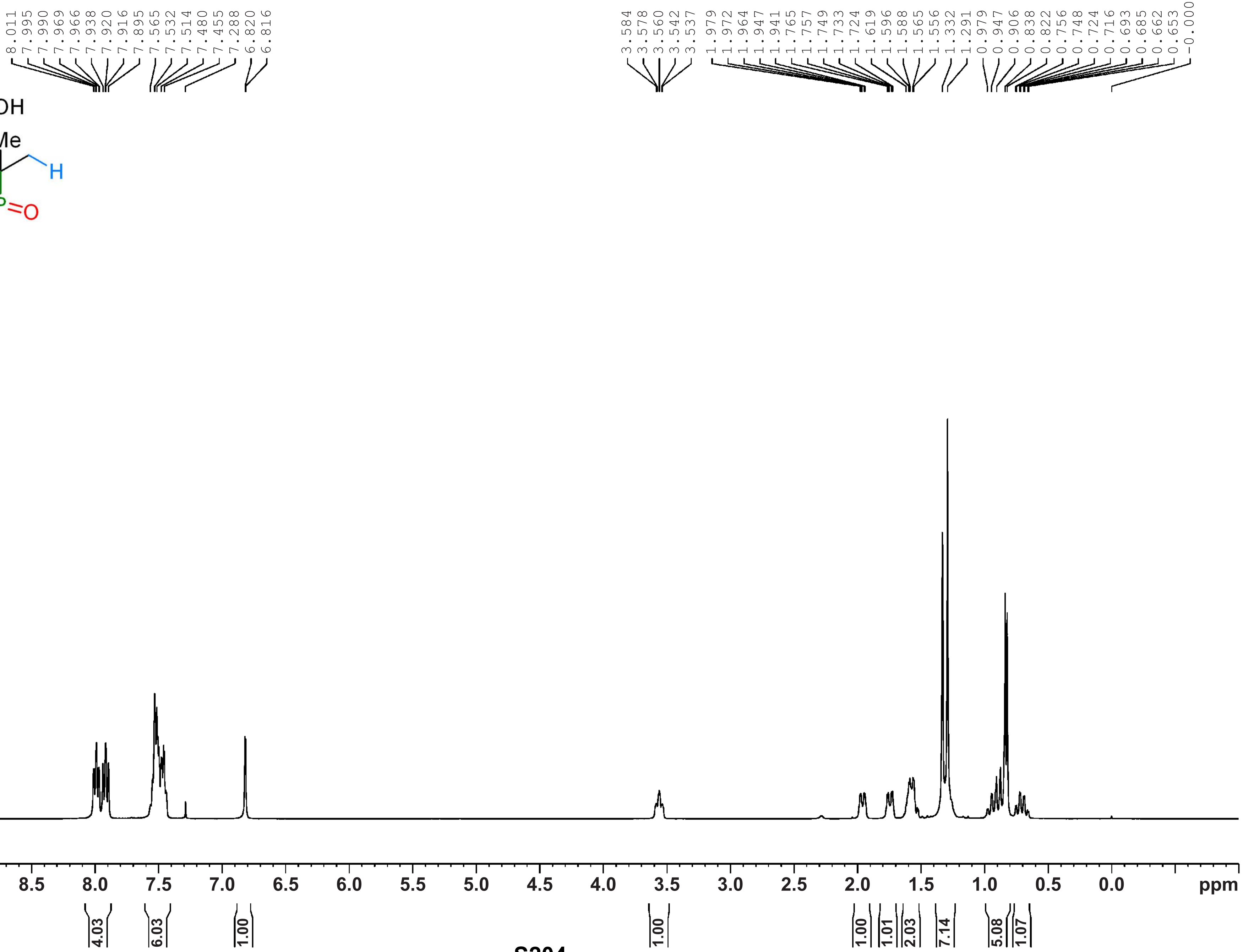

^{13}C NMR (100.6 MHz, $CDCl_3$)

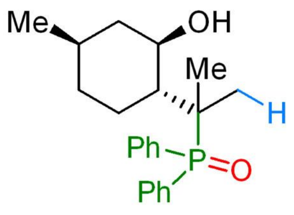

2al

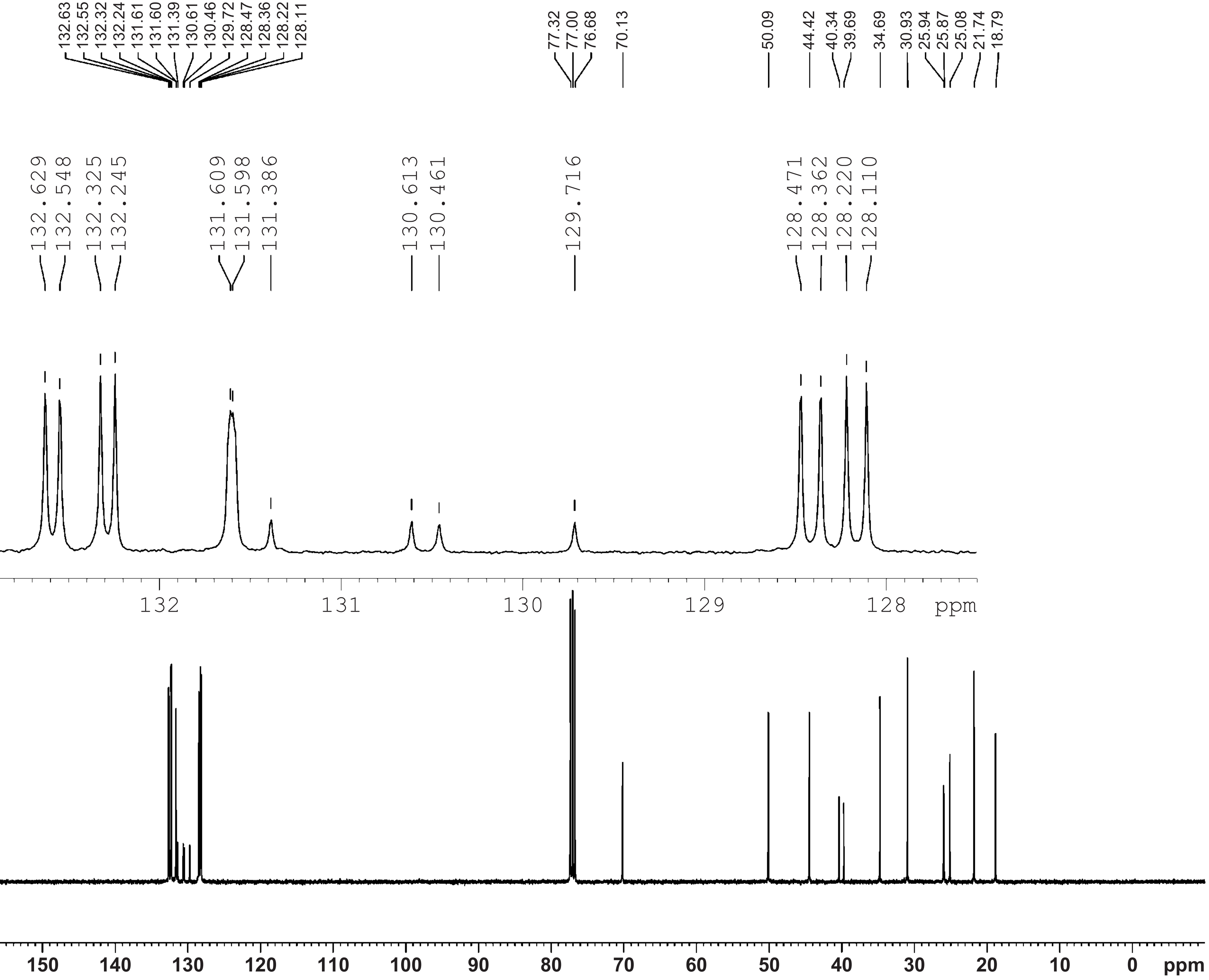

^{31}P NMR (162 MHz, $CDCl_3$)

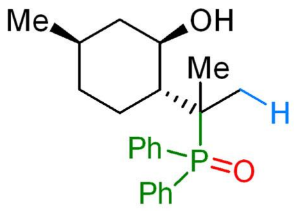

2al

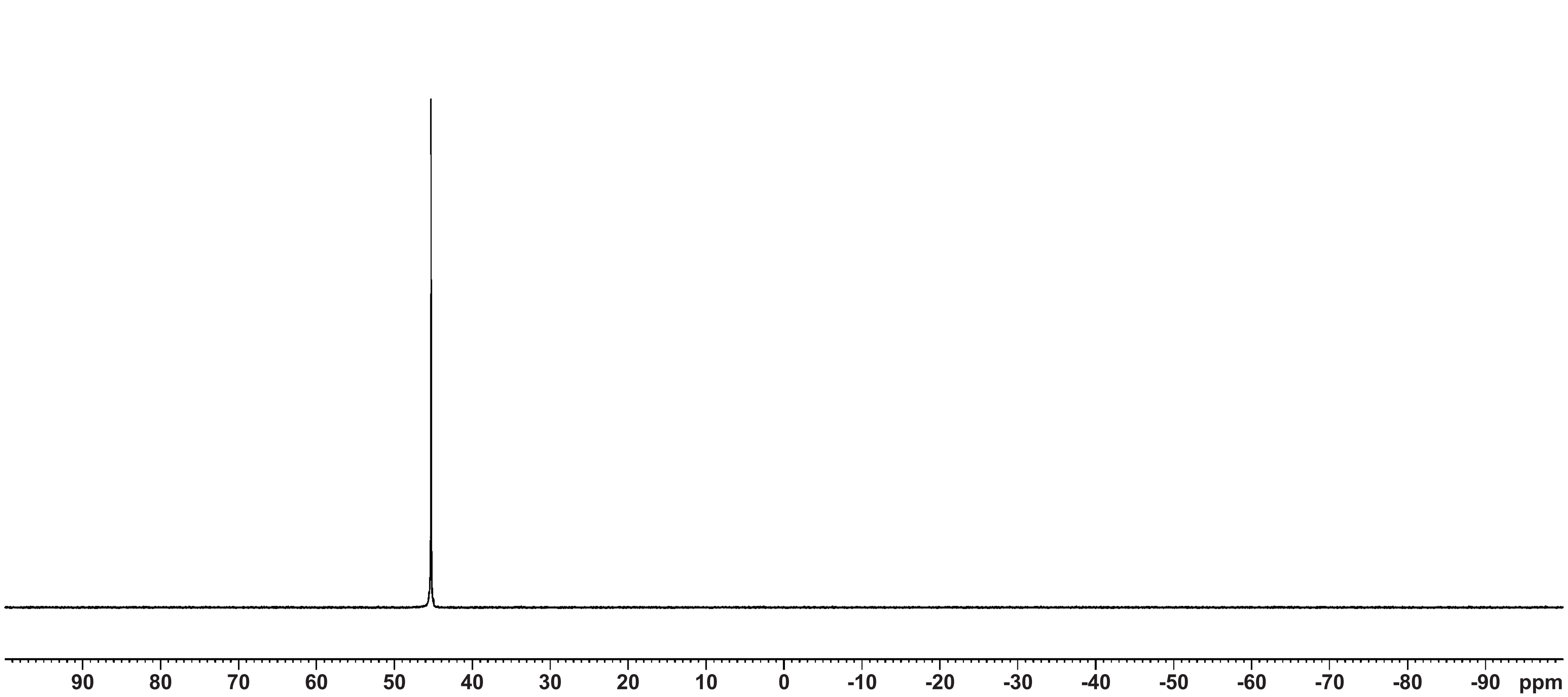

^{1}H NMR (400 MHz, $CDCl_3$)

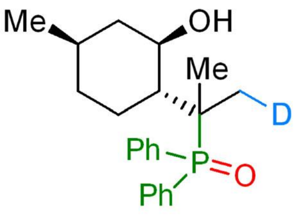

2al-D

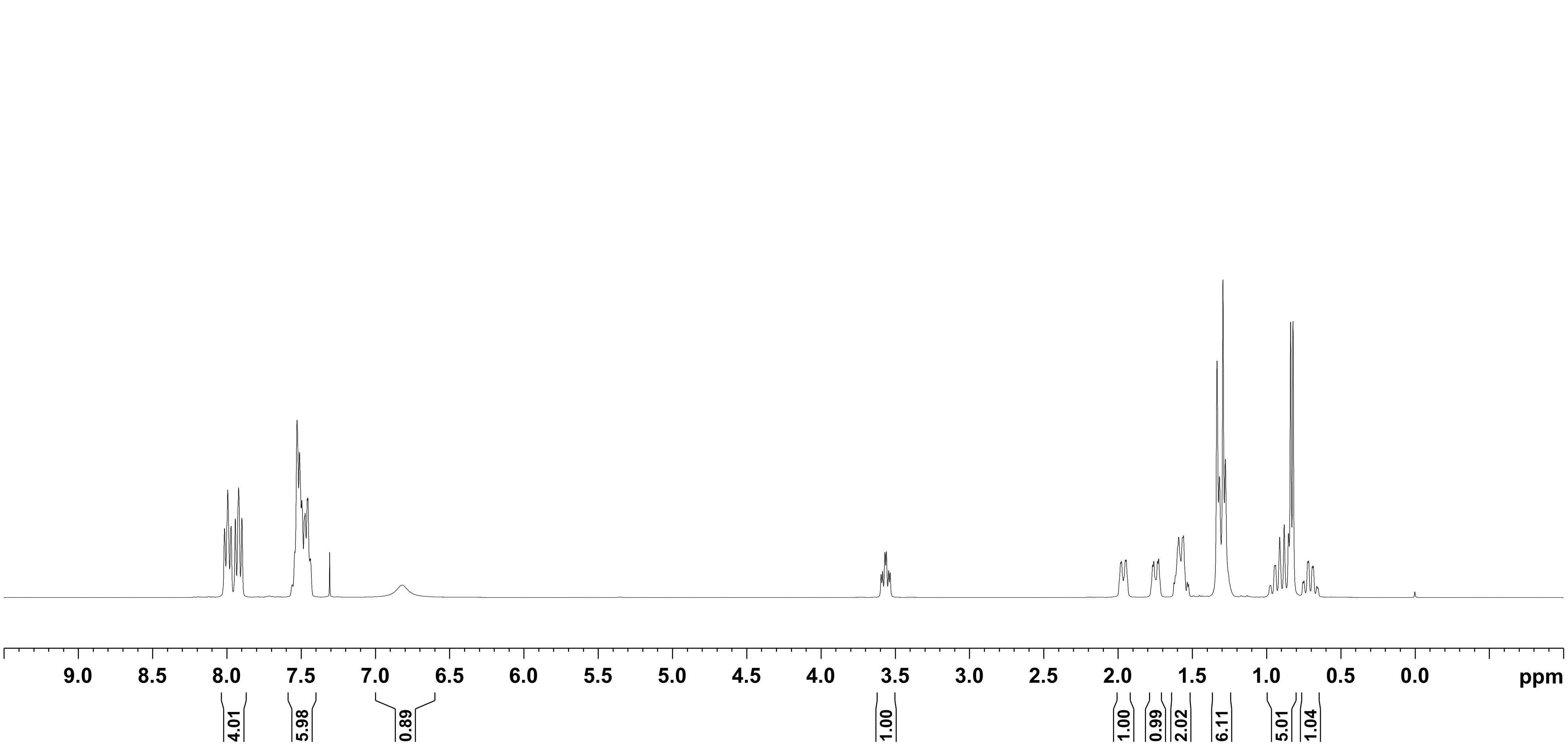

^{2}H NMR (92 MHz, $CHCl_3$)

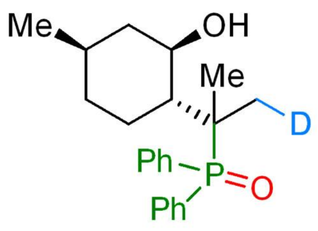

2al-D

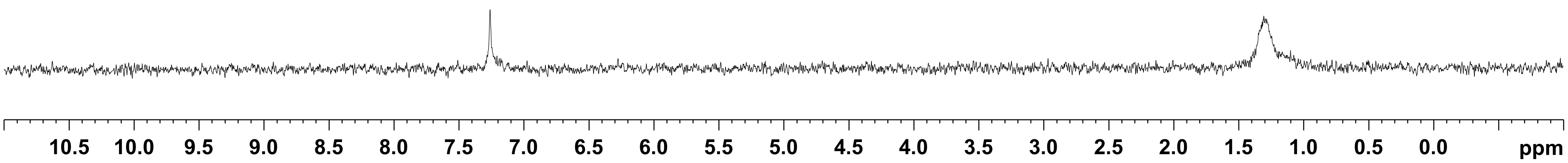

^{13}C NMR (100.6 MHz, CDCl$_3$)

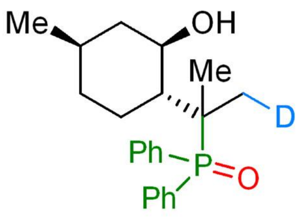

2al-D

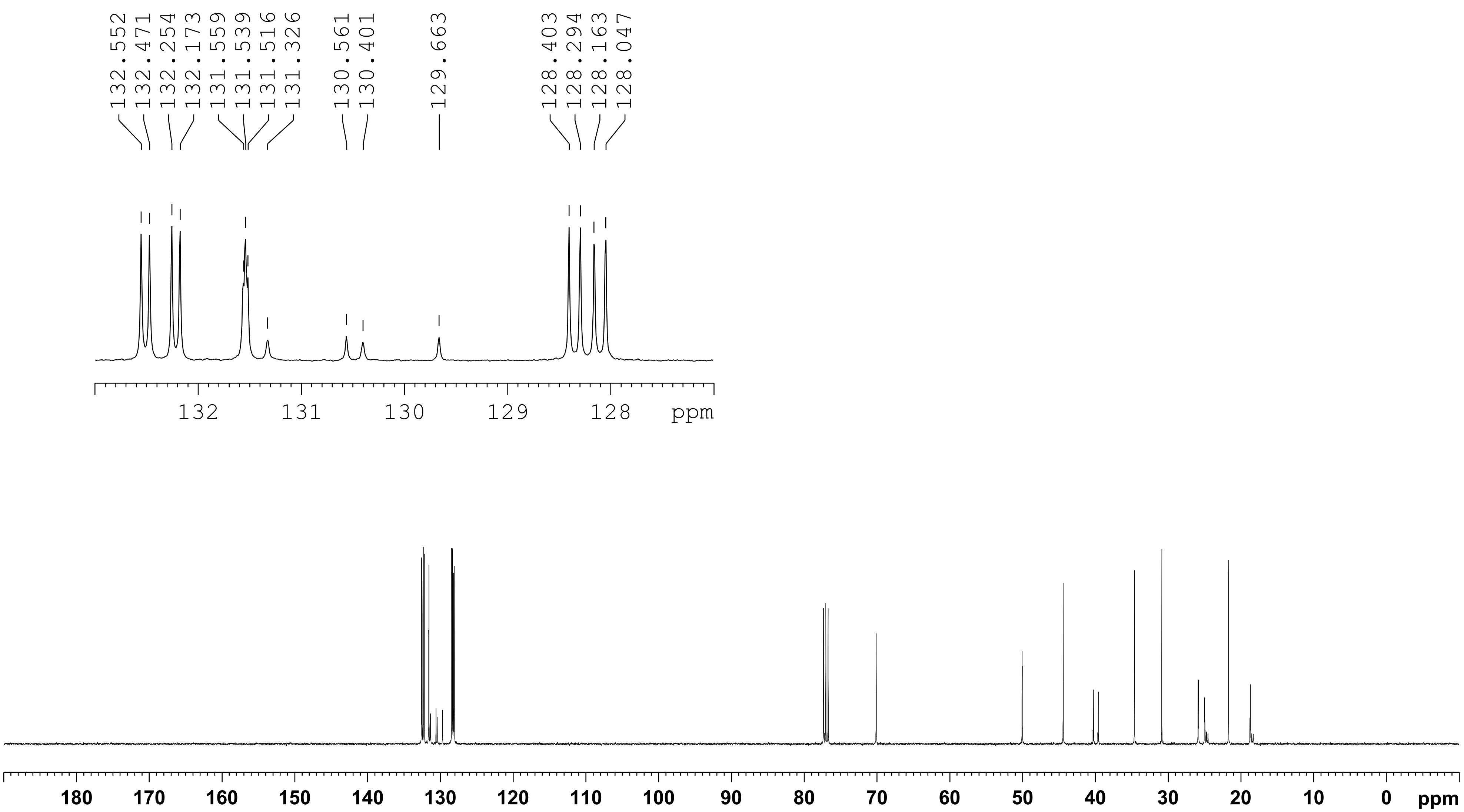

^{31}P NMR (162 MHz, $CDCl_3$)

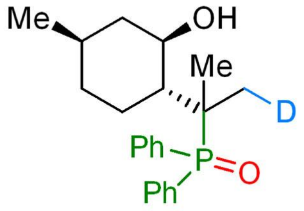

2al-D

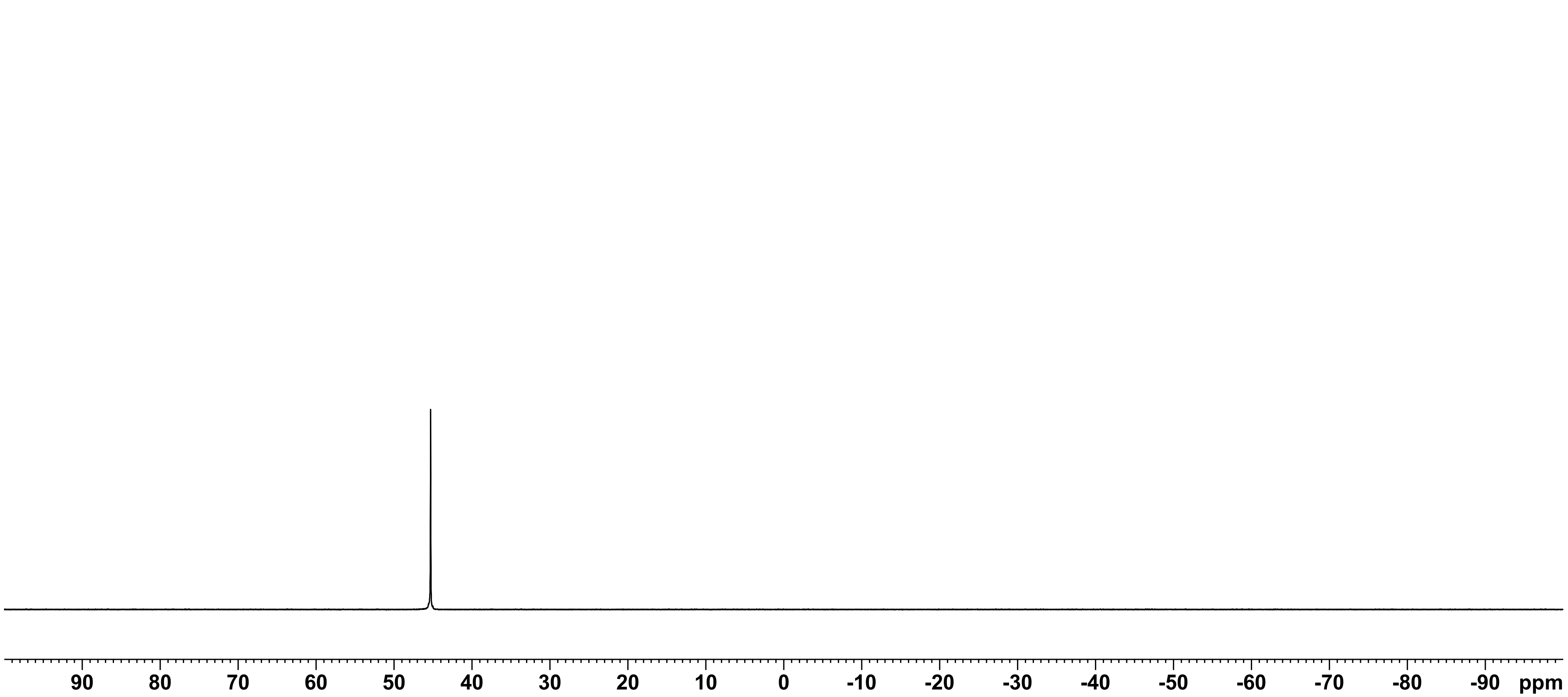

^{1}H NMR (400 MHz, $CDCl_3$)

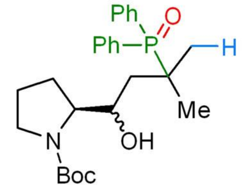

2am

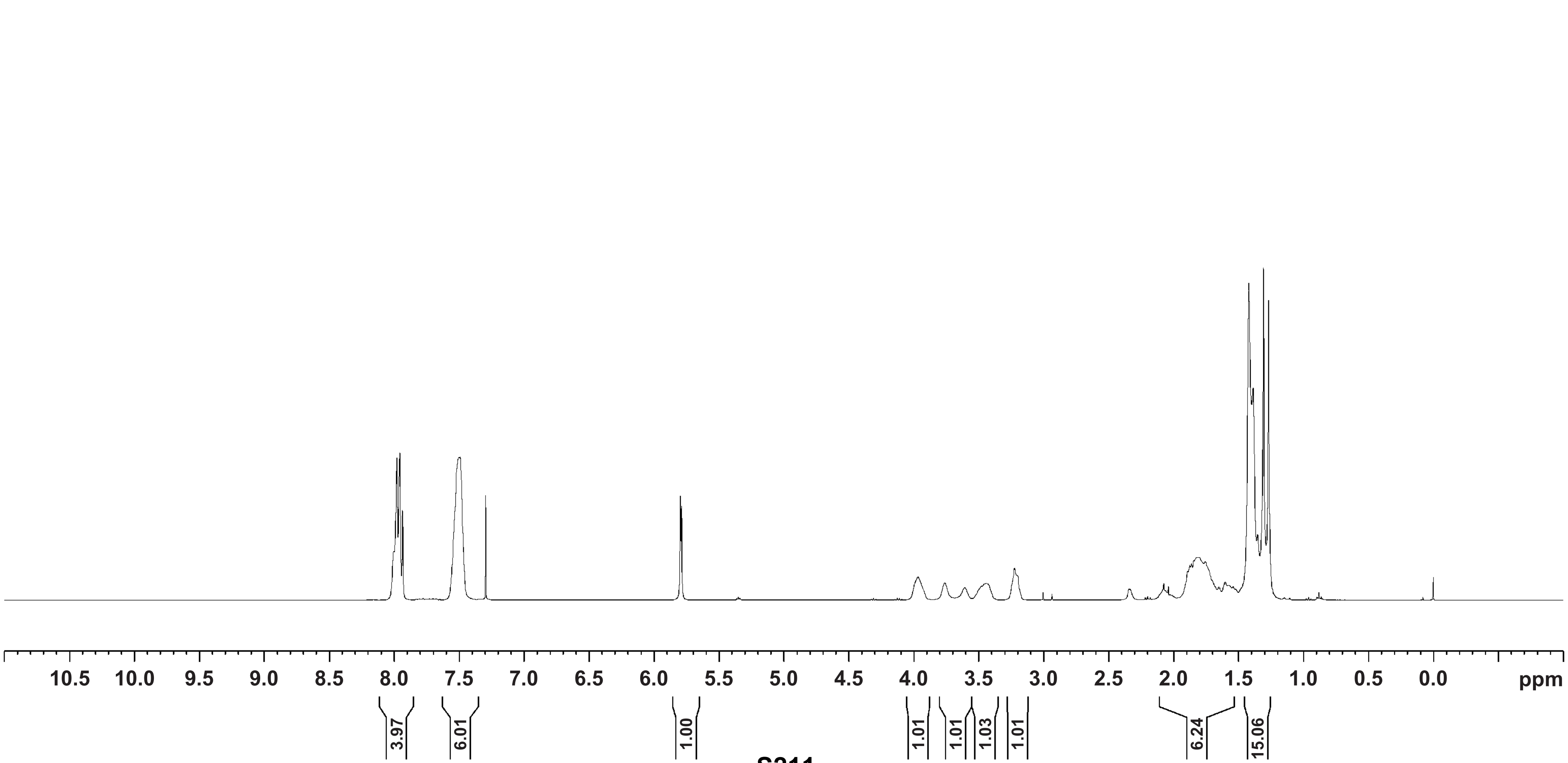

^{13}C NMR (100.6 MHz, $CDCl_3$)

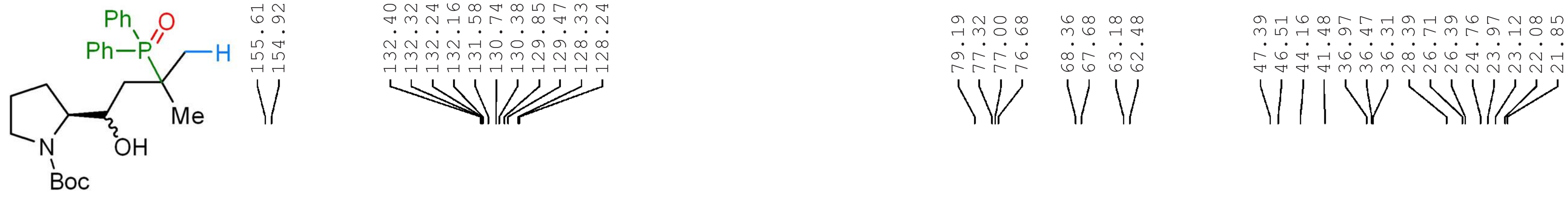

2am

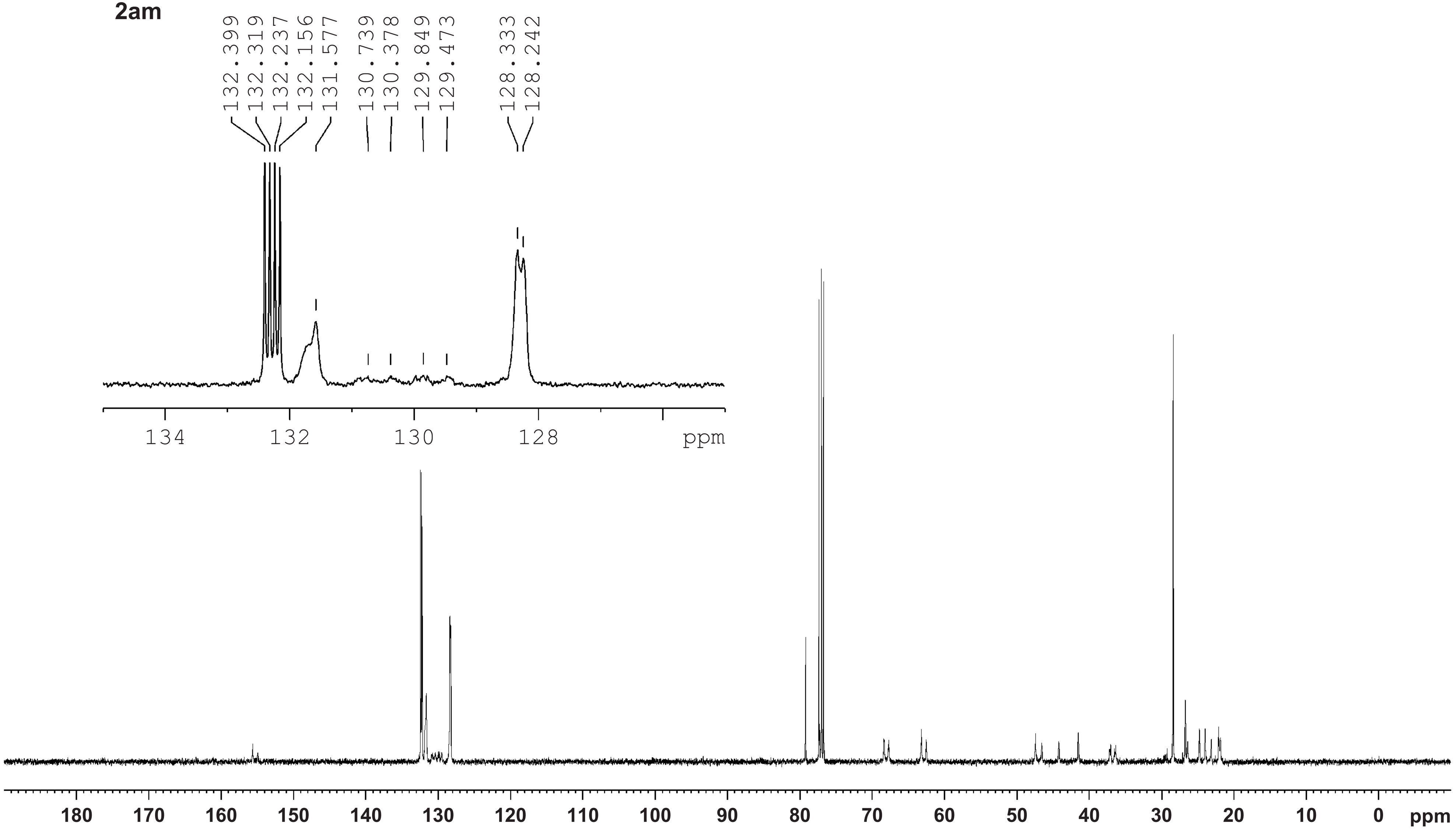

^{31}P NMR (162 MHz, $CDCl_3$)

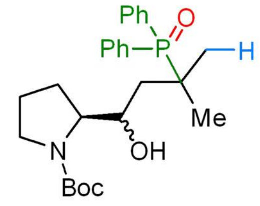

2am

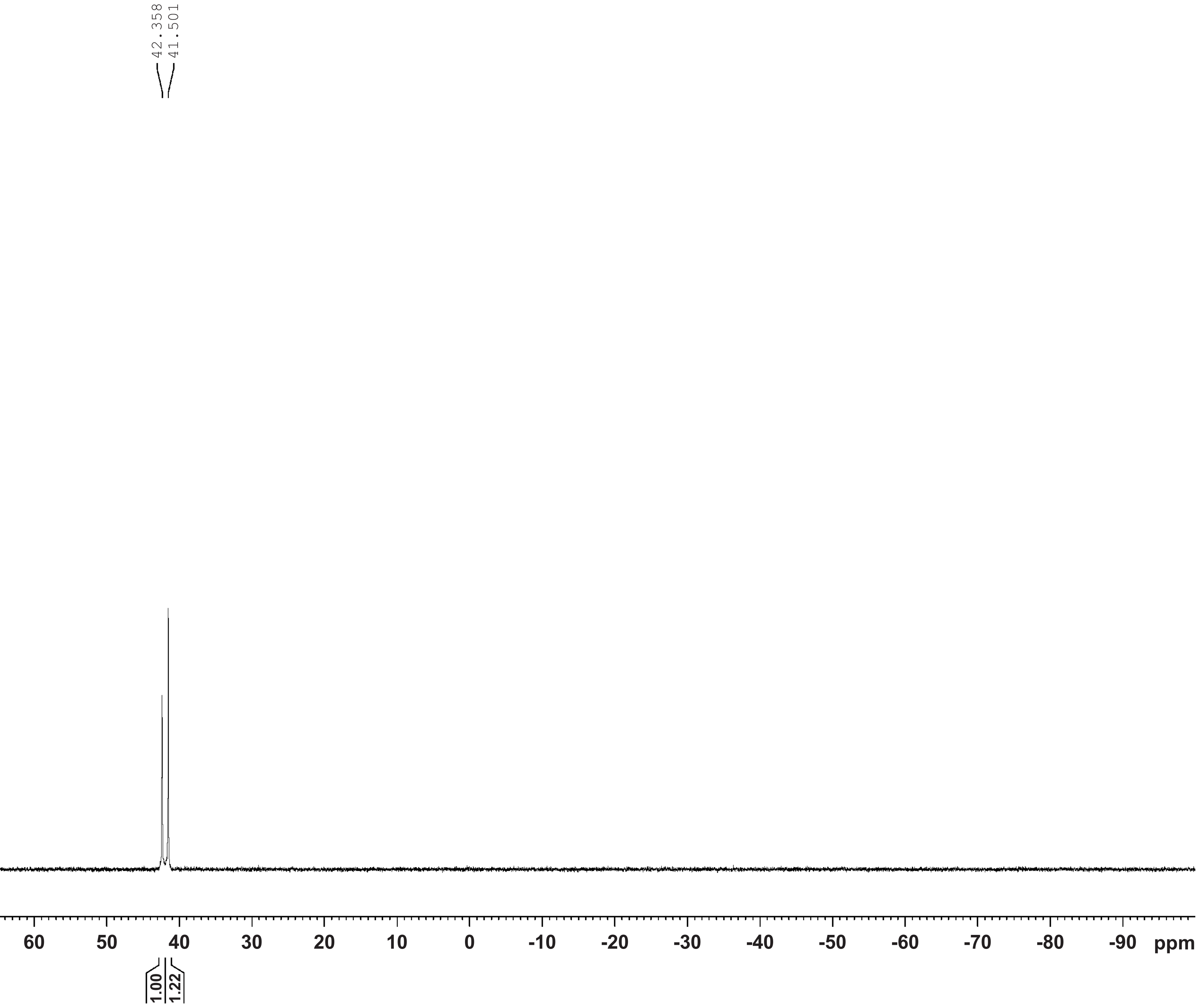

^{1}H NMR (400 MHz, $CDCl_3$)

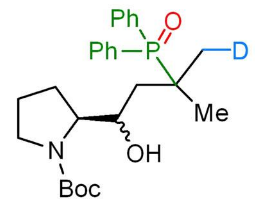

2am-D

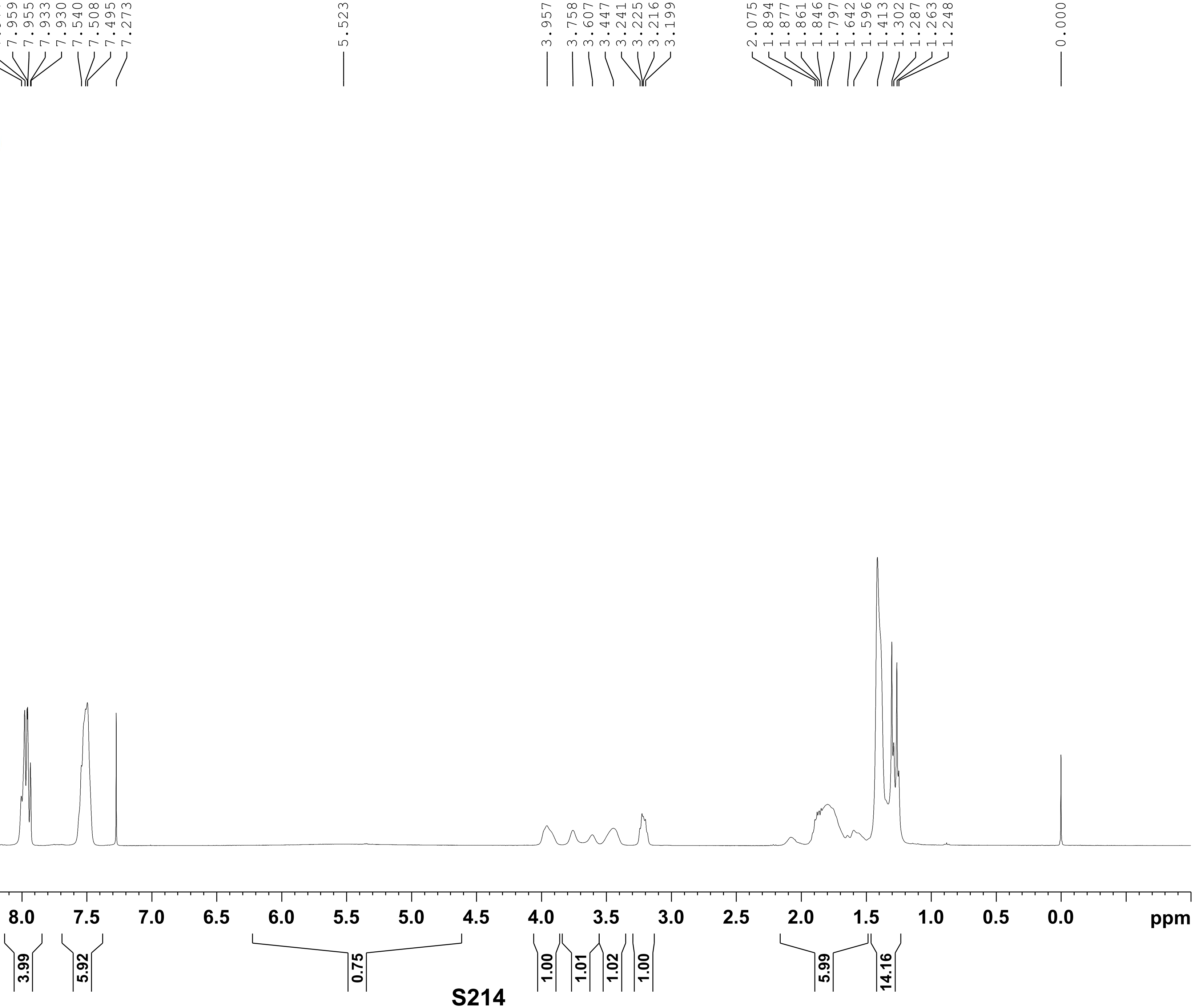

^{2}H NMR (92 MHz, $CHCl_3$)

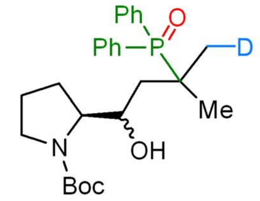

2am-D

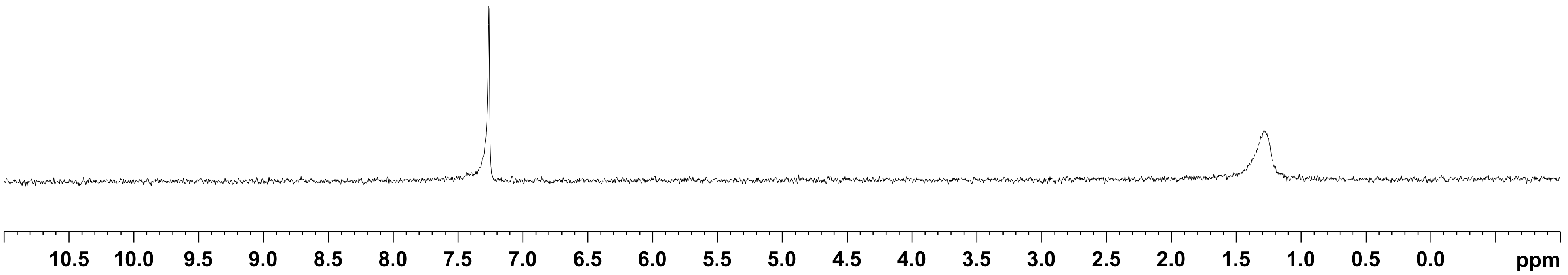

^{13}C NMR (100.6 MHz, CDCl$_3$)

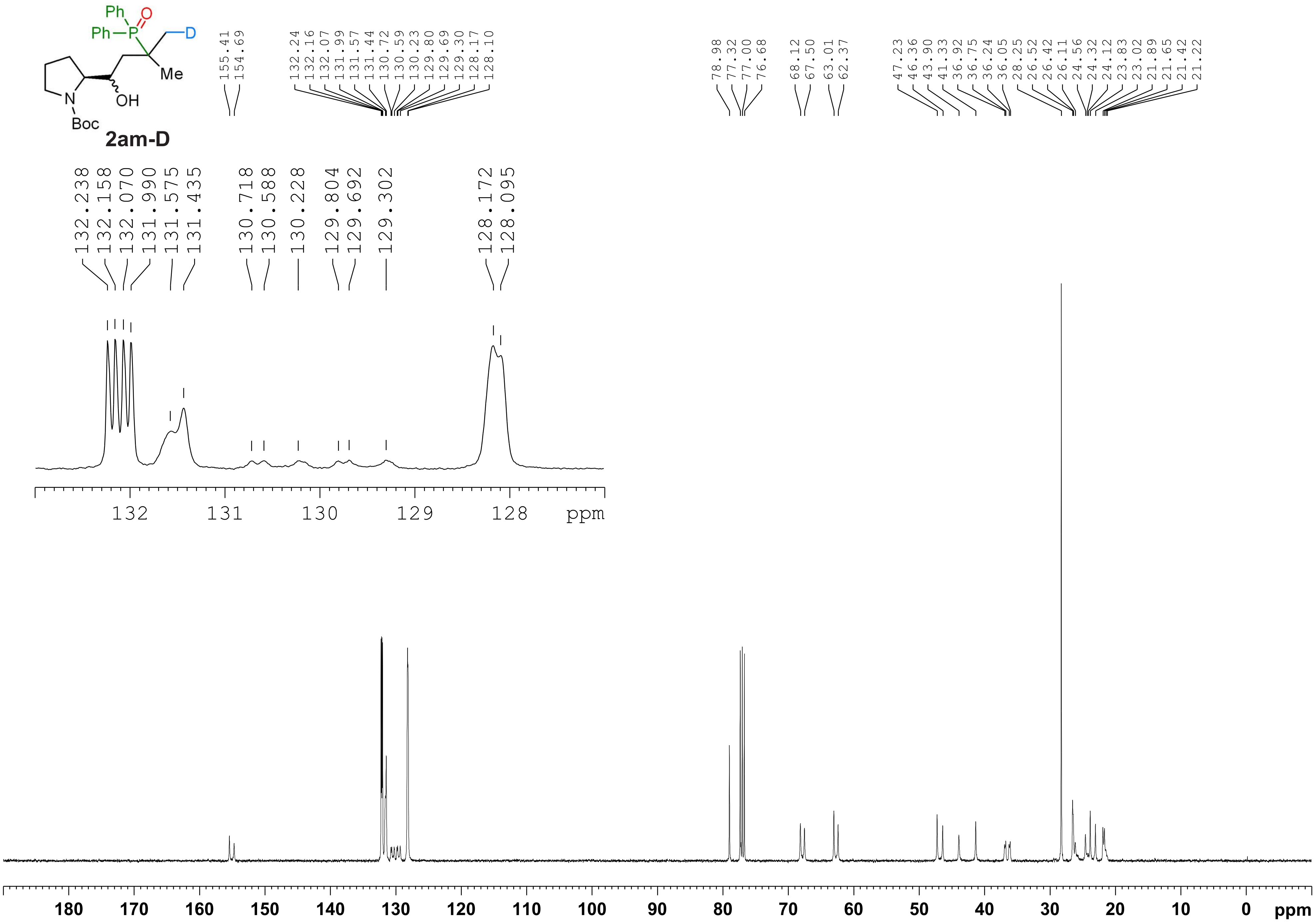

^{31}P NMR (162 MHz, $CDCl_3$)

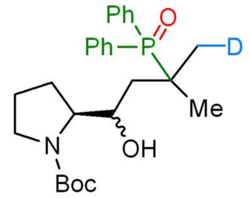

2am-D

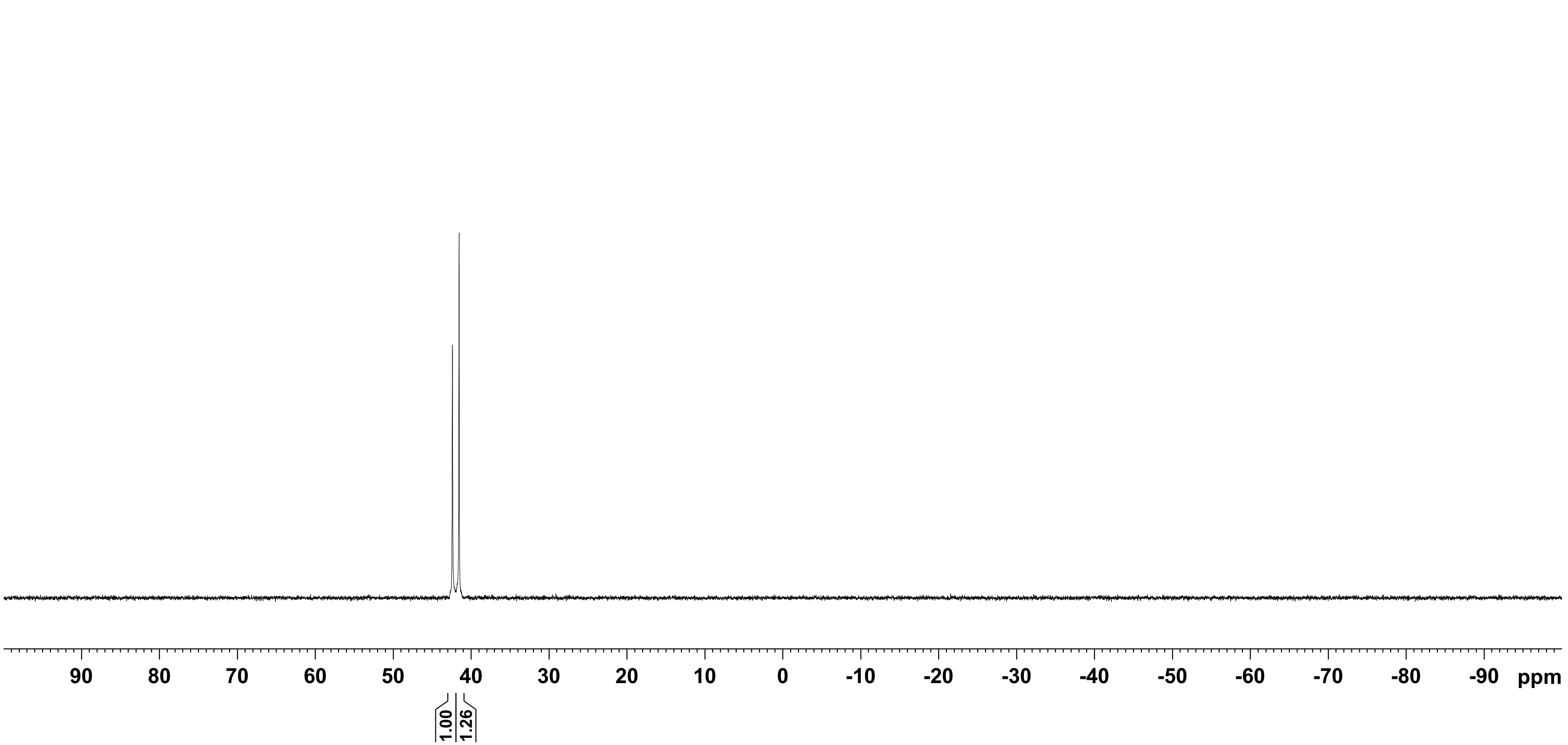

^{1}H NMR (400 MHz, $CDCl_3$)

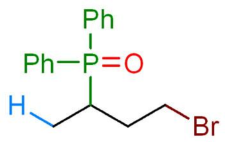

3a

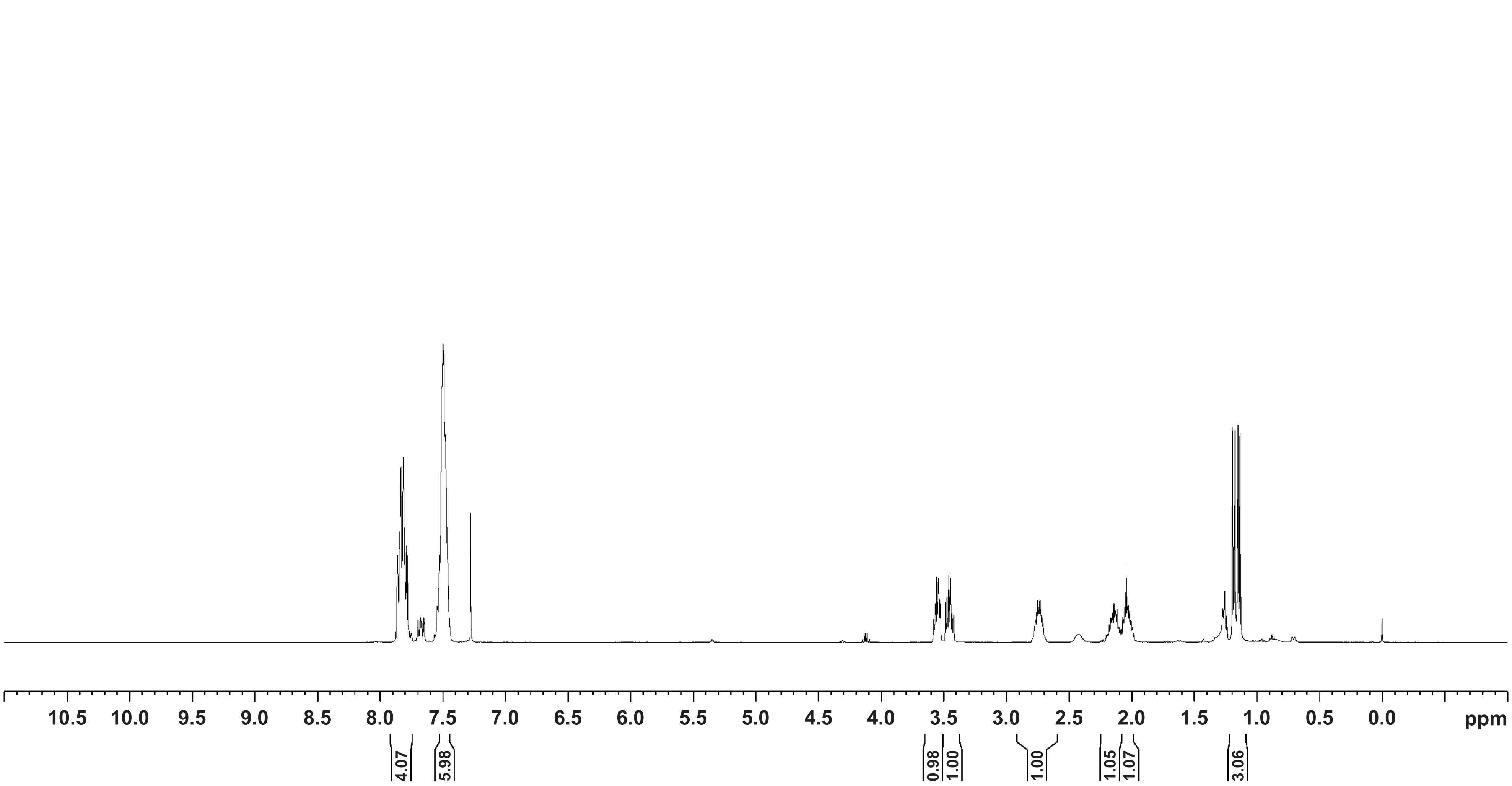

^{13}C NMR (100.6 MHz, $CDCl_3$)

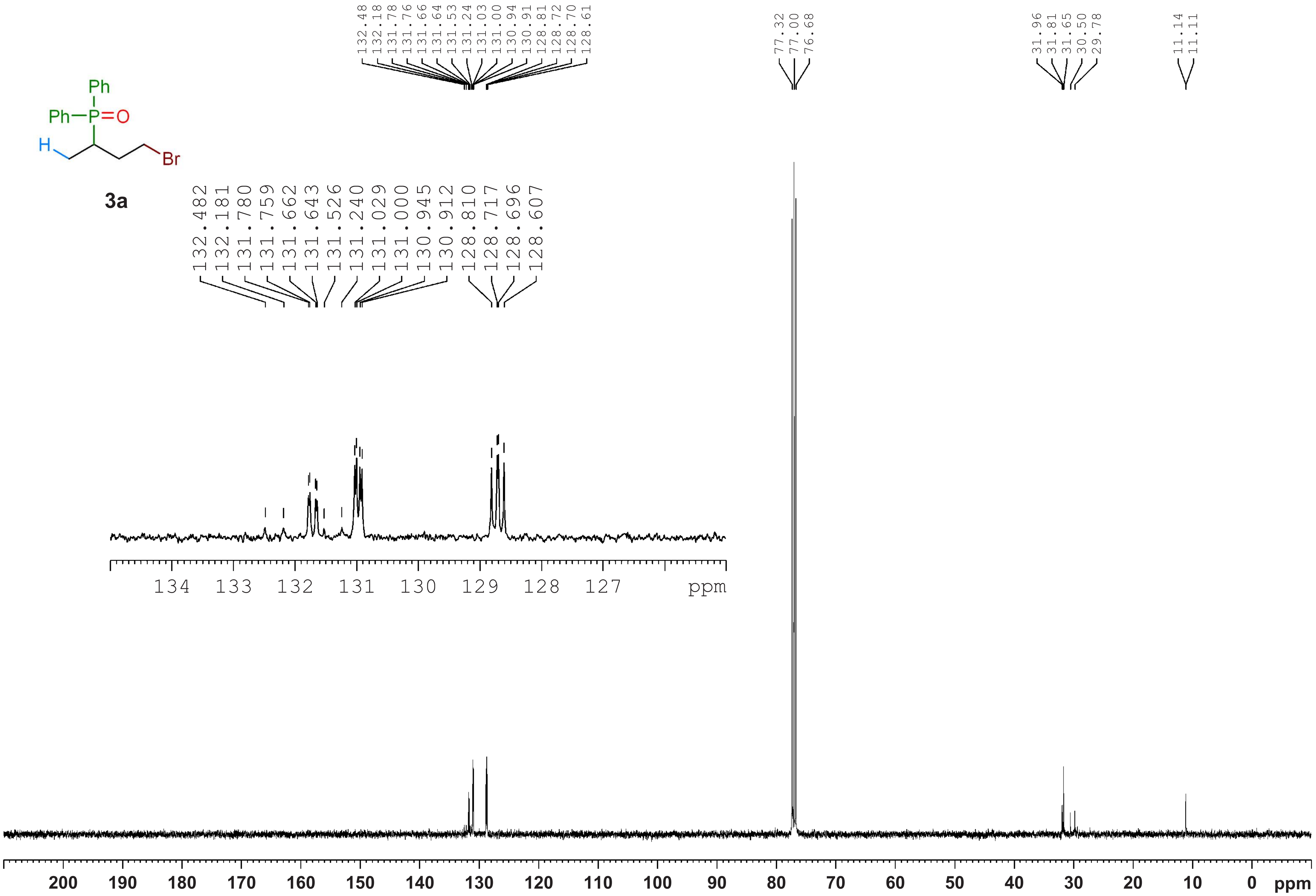

^{31}P NMR (162 MHz, $CDCl_3$)

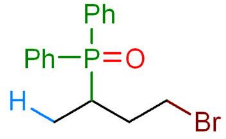

3a

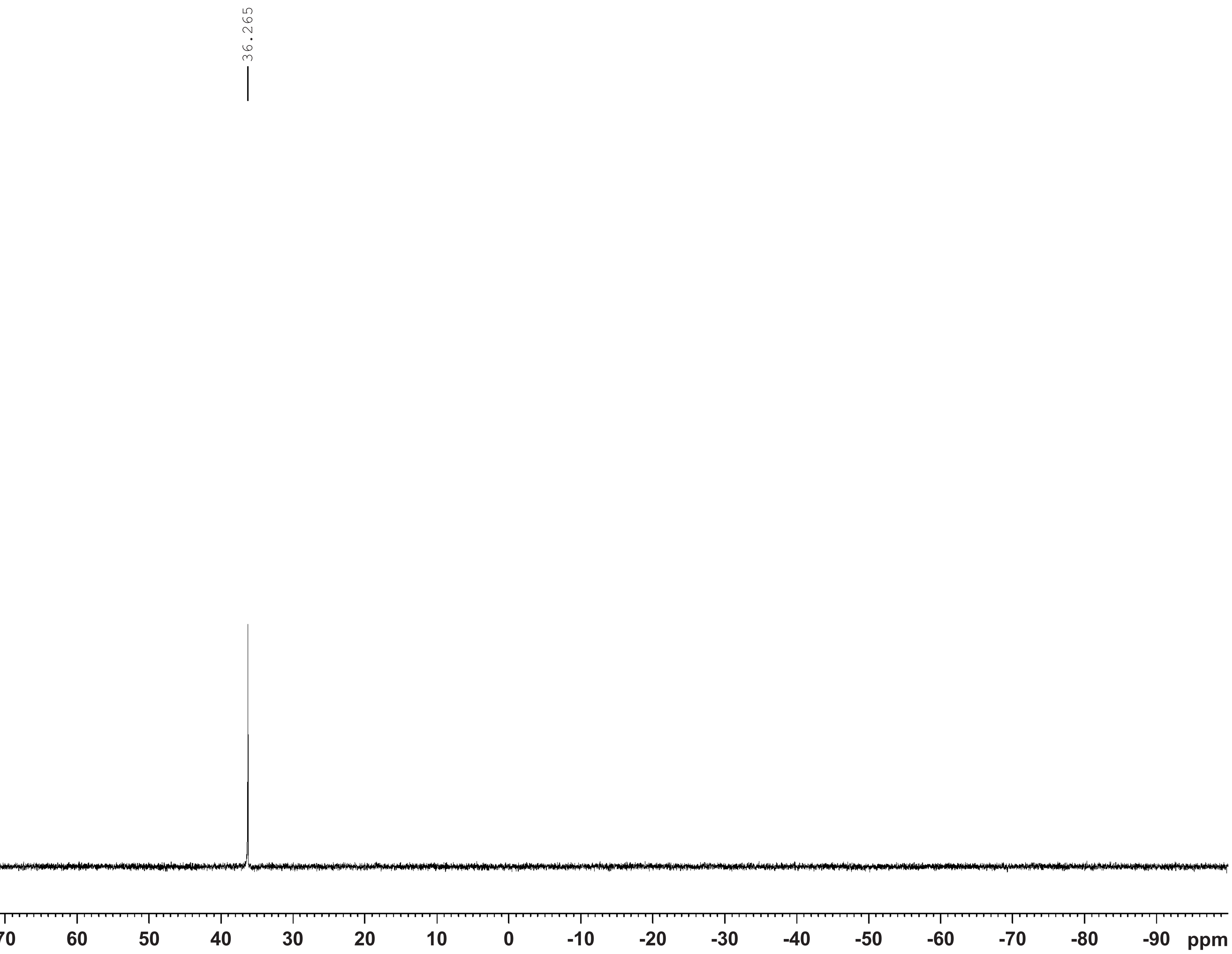

^{1}H NMR (400 MHz, $CDCl_3$)

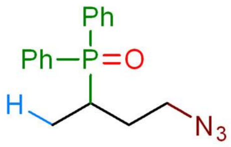

3b

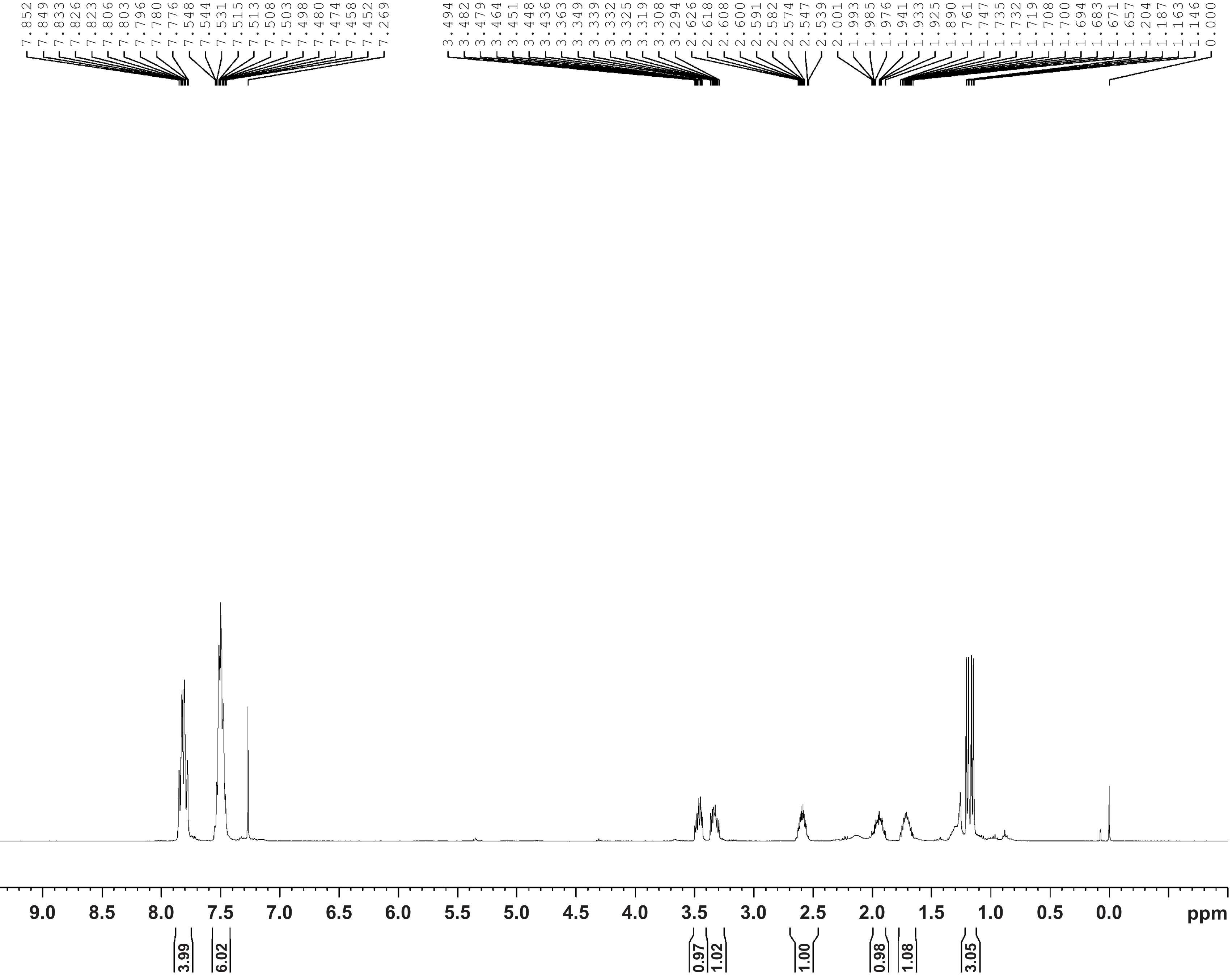

^{13}C NMR (100.6 MHz, $CDCl_3$)

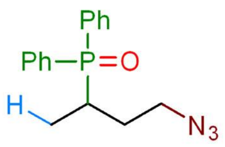

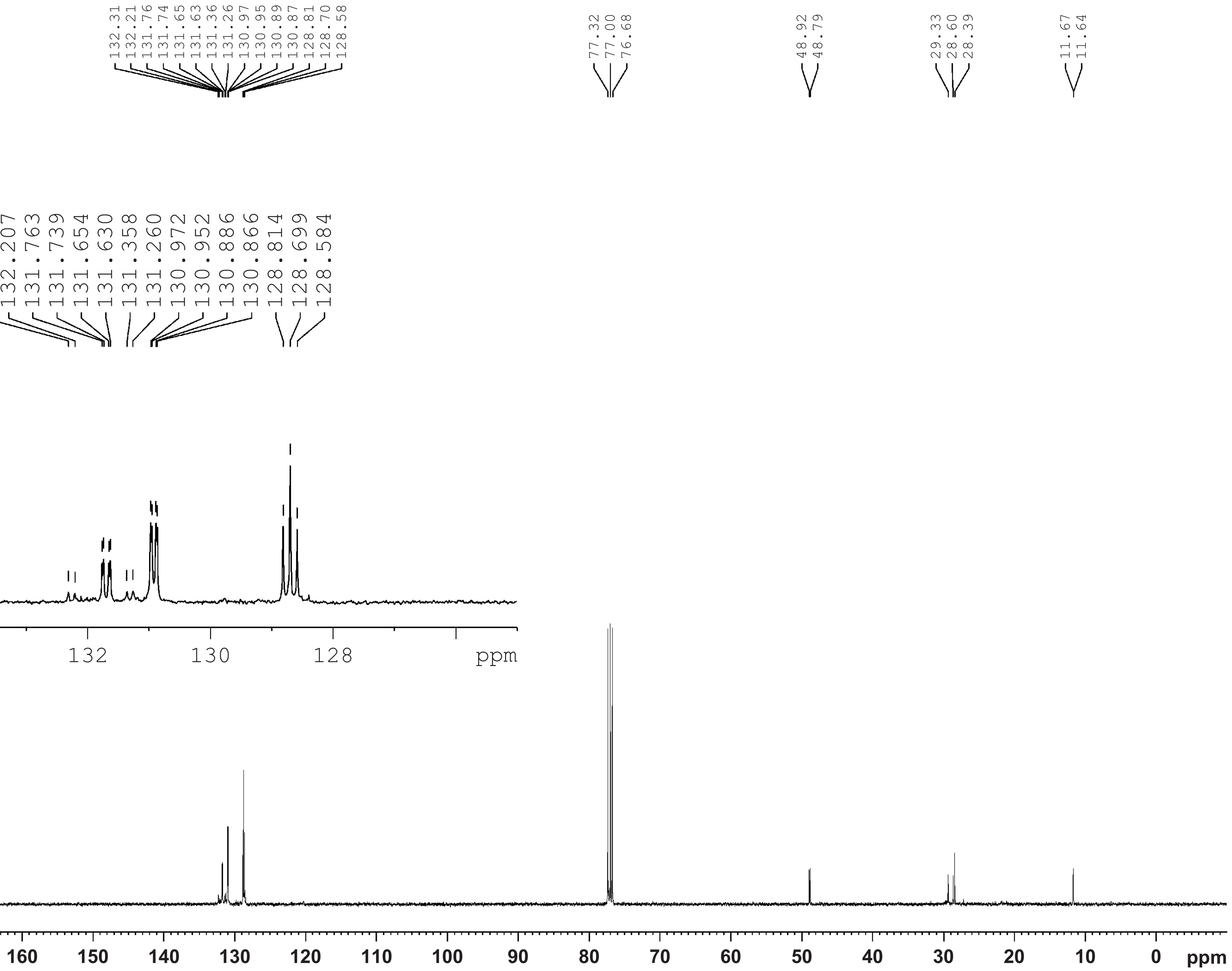

^{31}P NMR (162 MHz, $CDCl_3$)

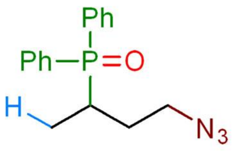

3b

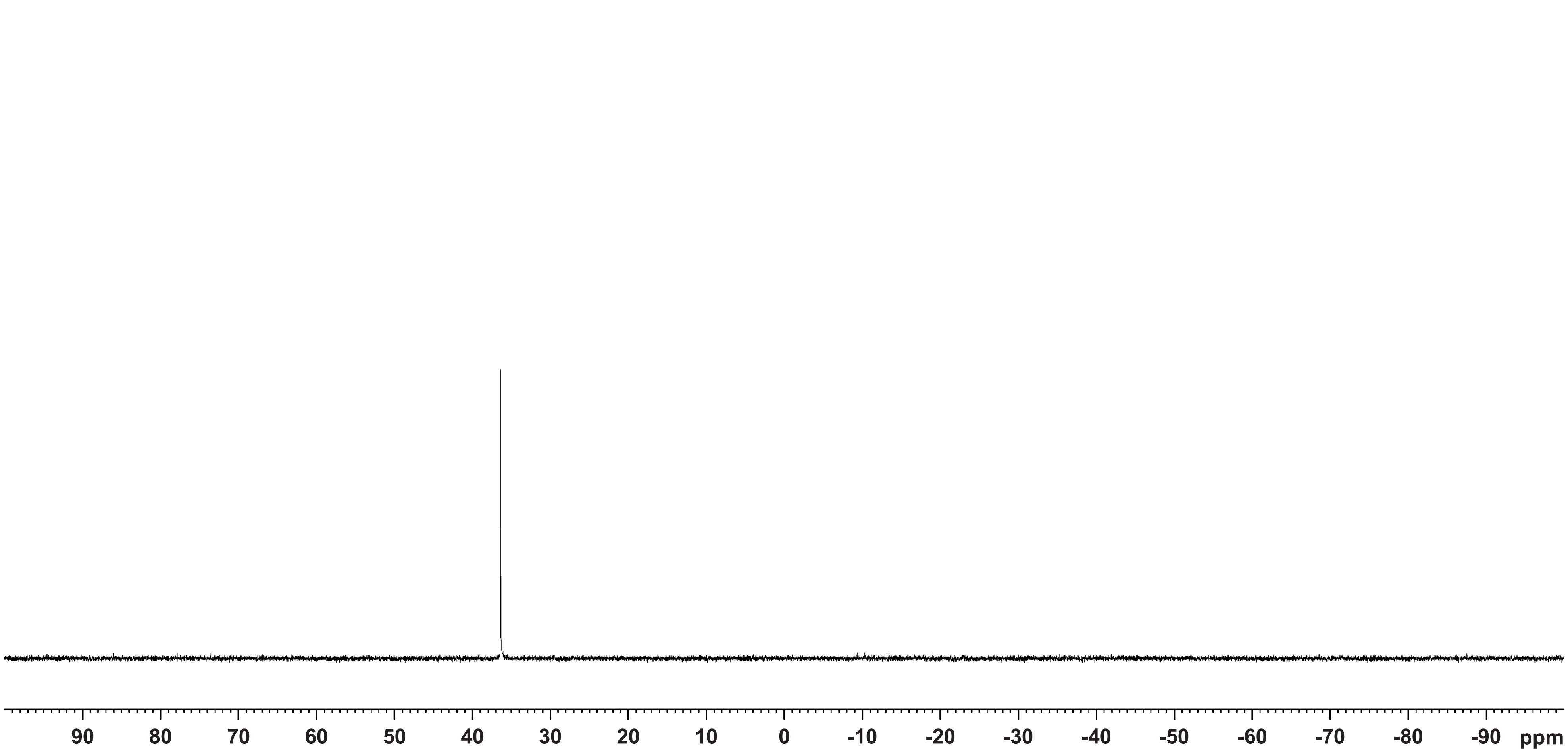

^{1}H NMR (400 MHz, $CDCl_3$)

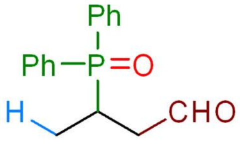

3c

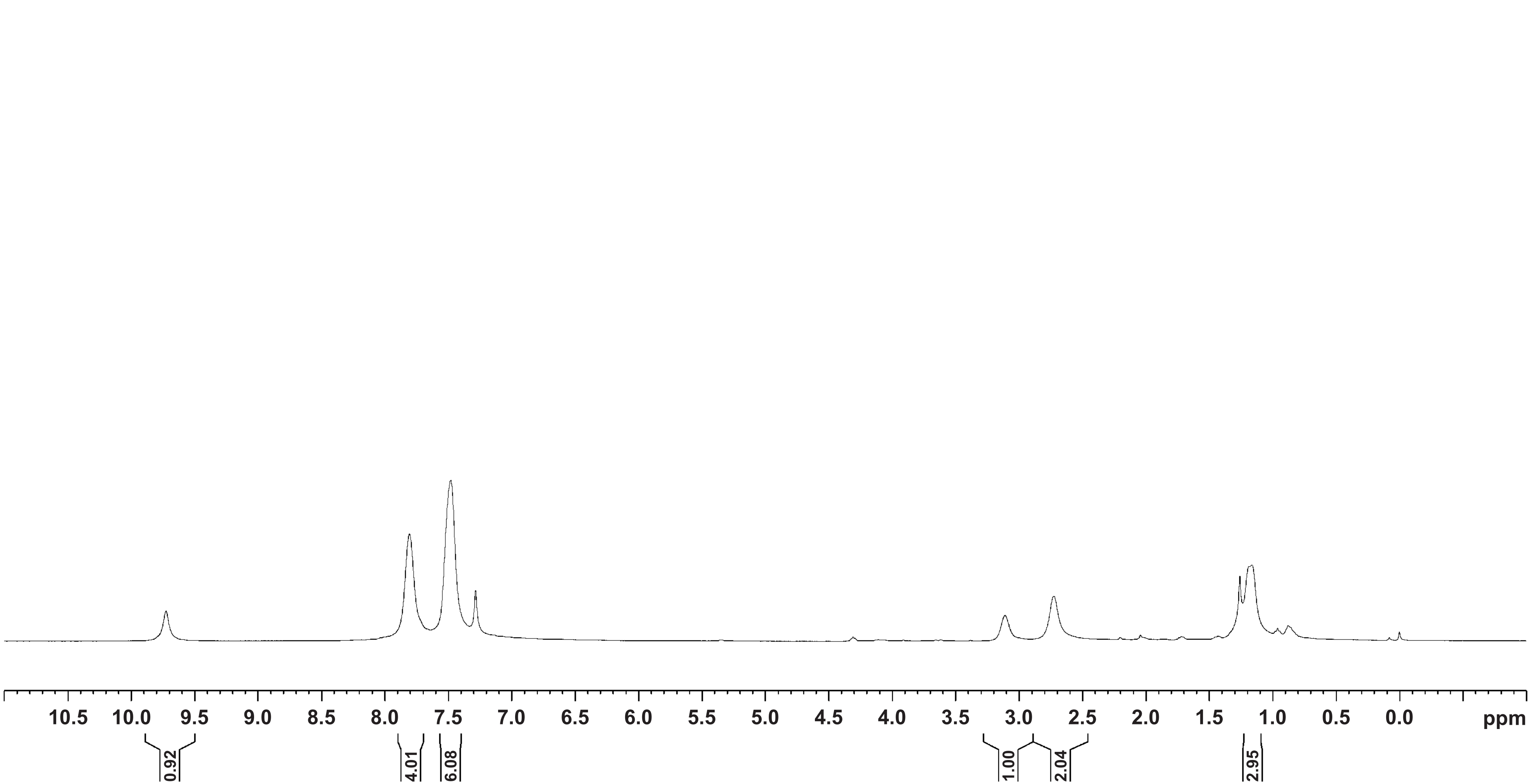

^{13}C NMR (100.6 MHz, $CDCl_3$)

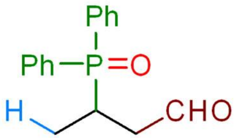

3c

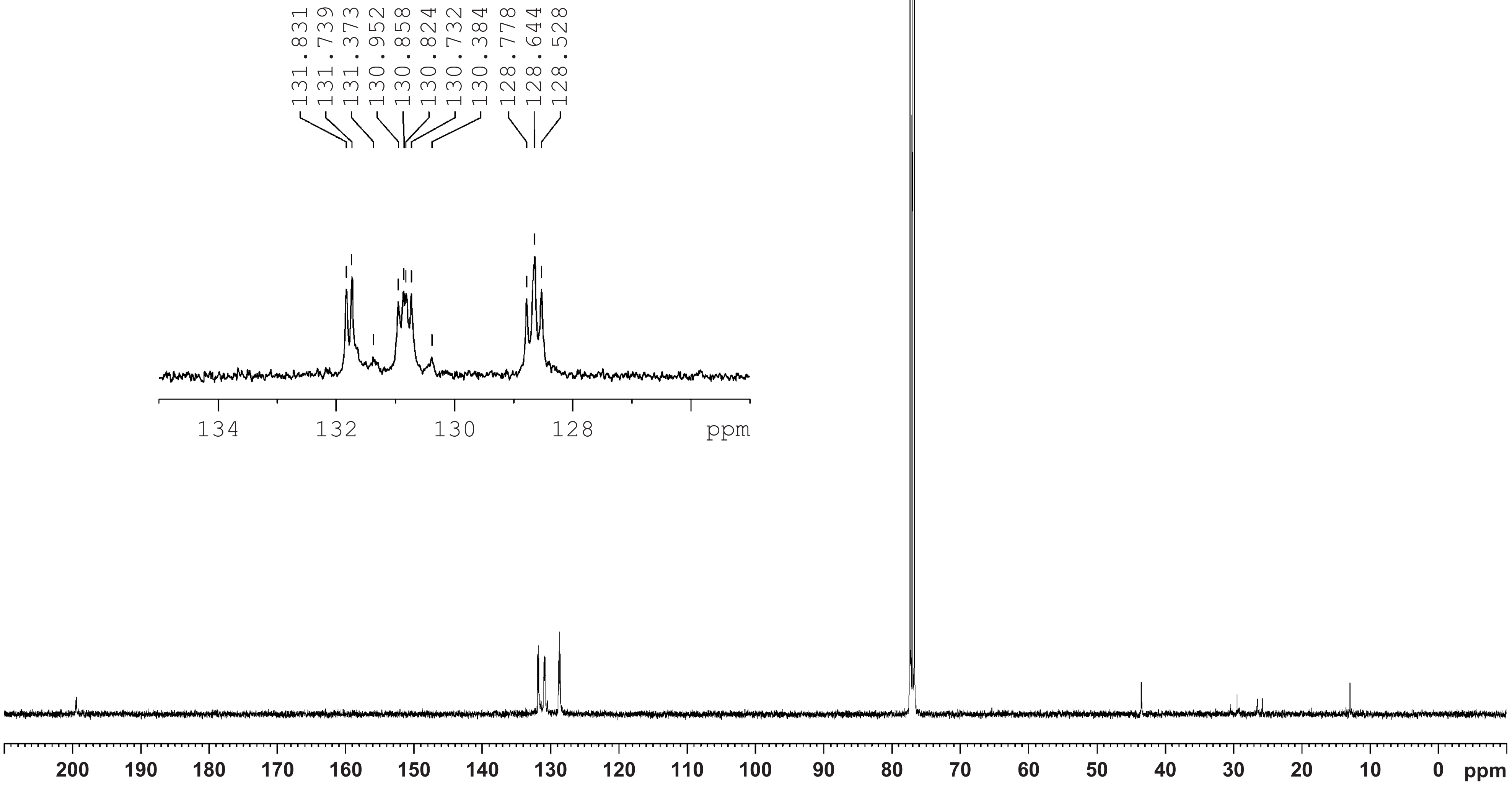

^{31}P NMR (162 MHz, $CDCl_3$)

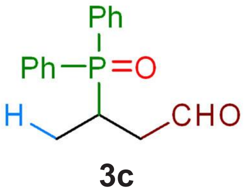

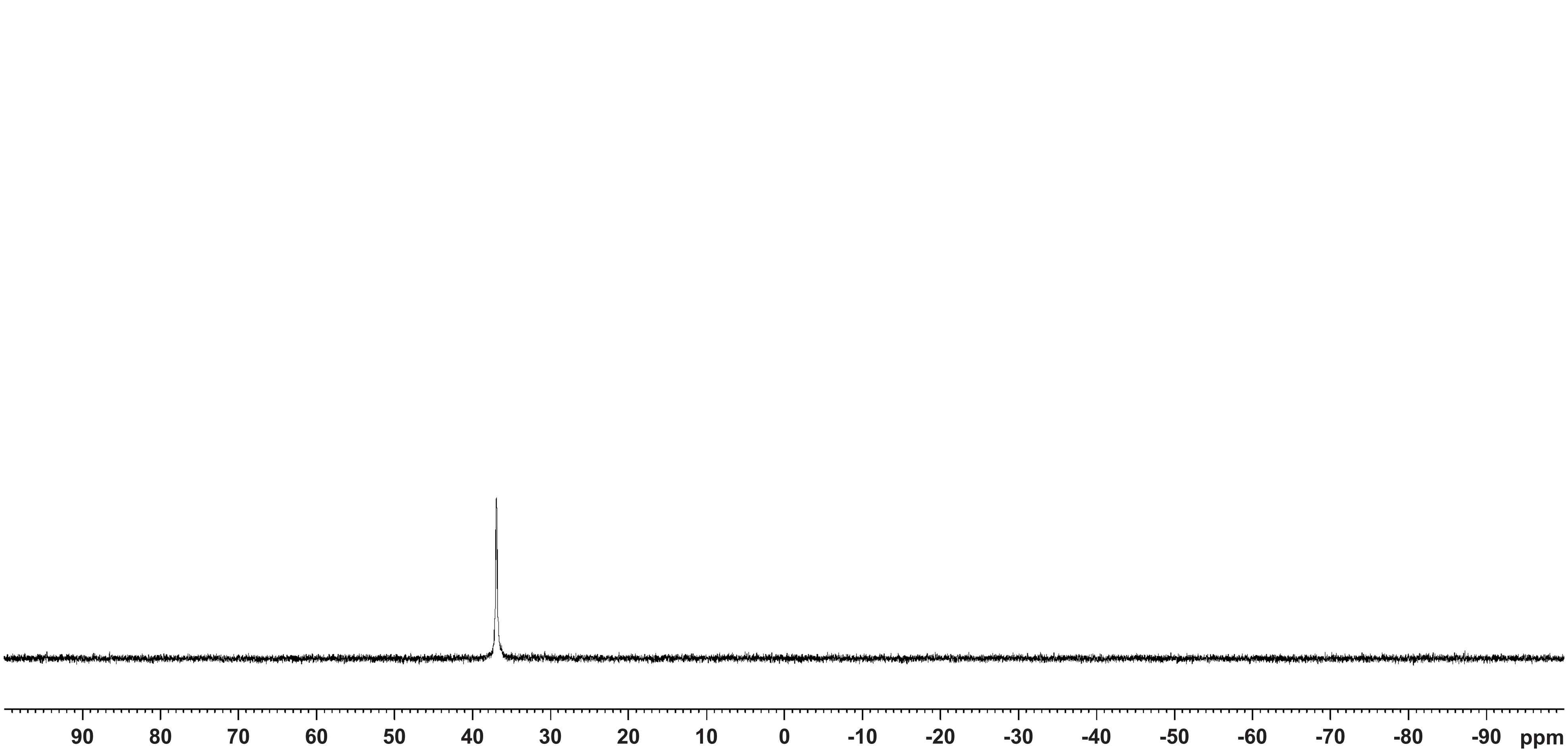

^{1}H NMR (400 MHz, $CDCl_3$)

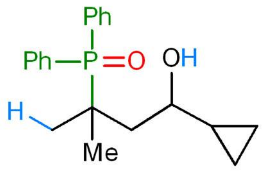

2an

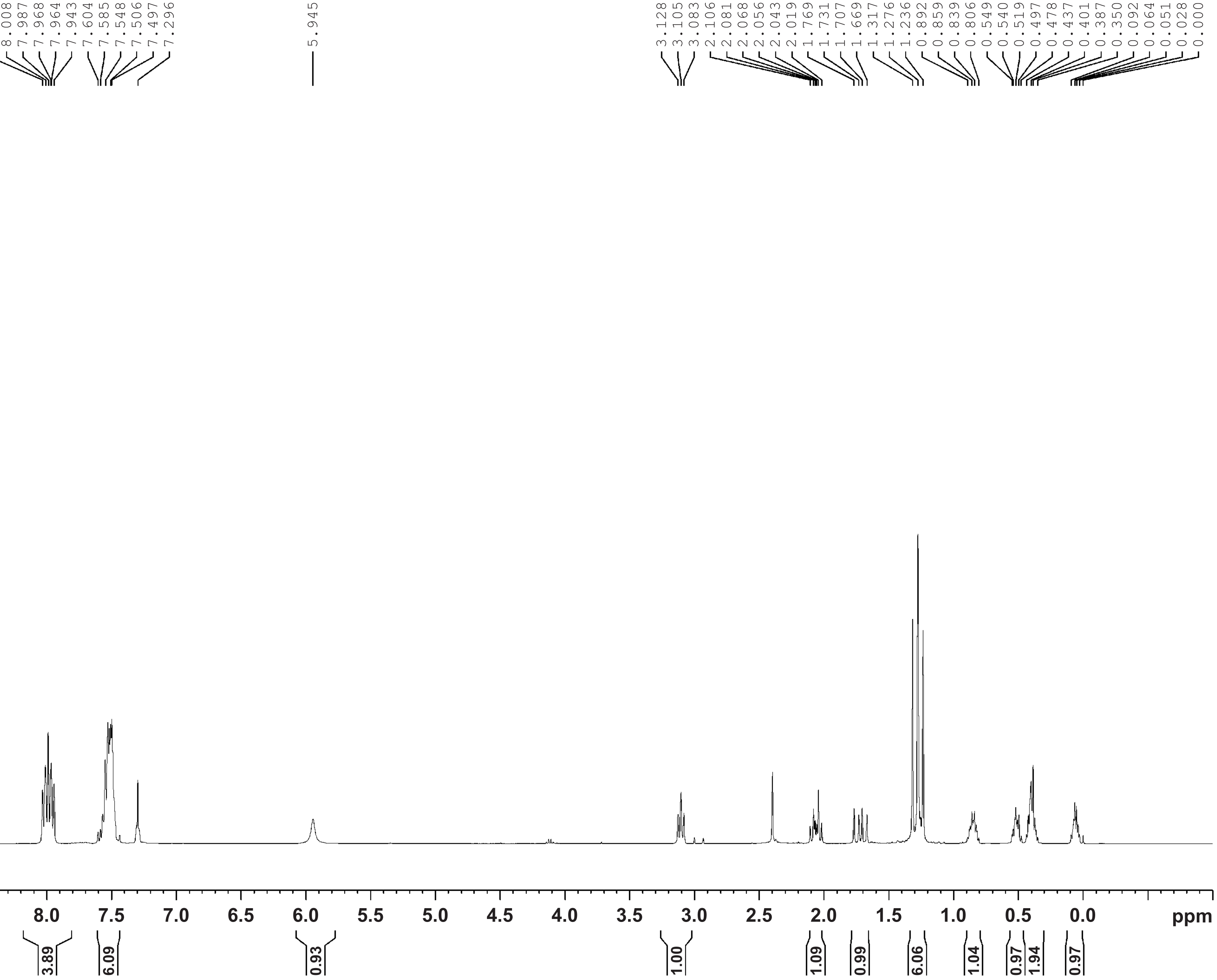

^{13}C NMR (100.6 MHz, $CDCl_3$)

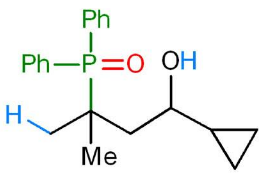

2an

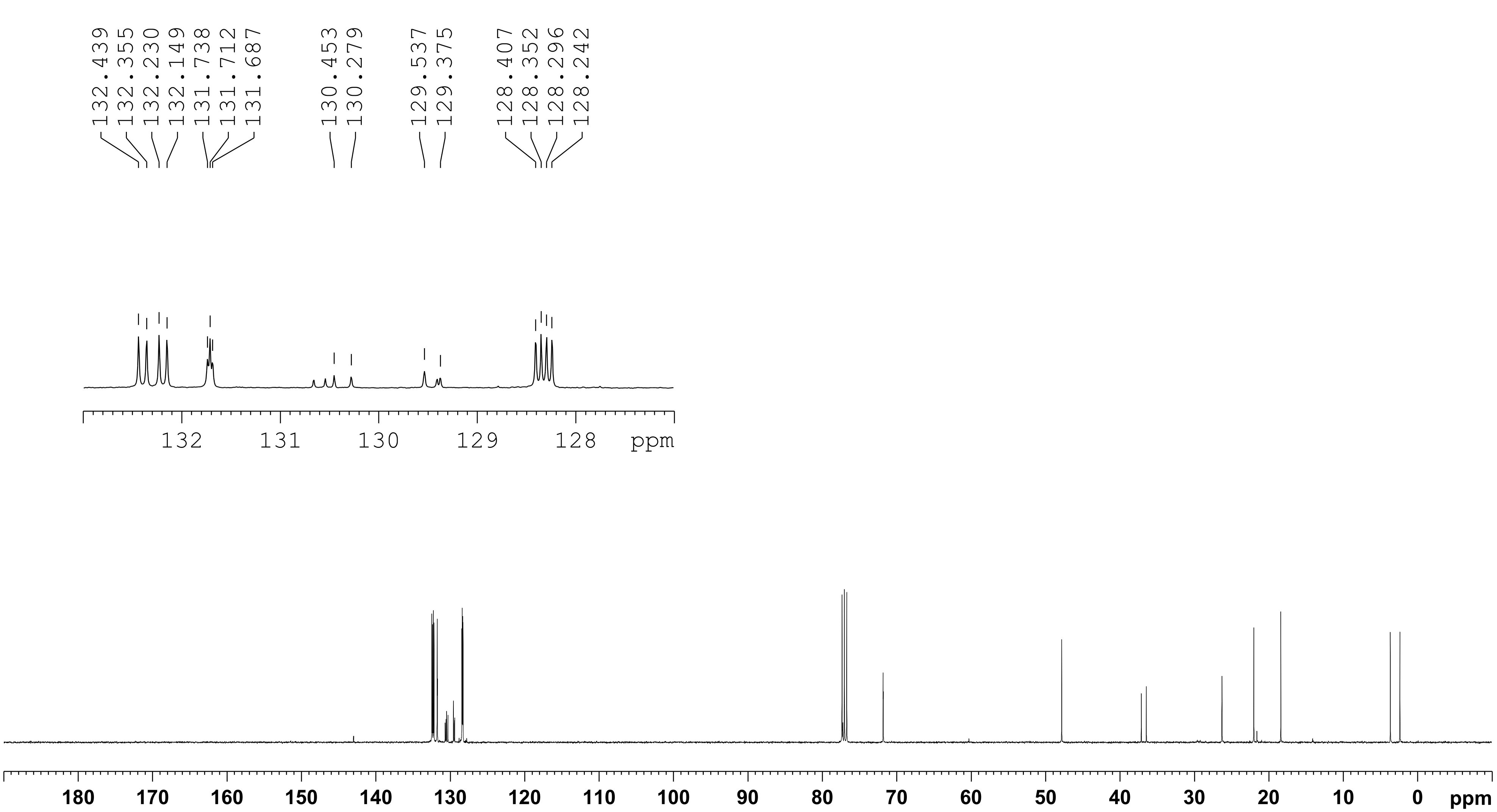

^{31}P NMR (162 MHz, $CDCl_3$)

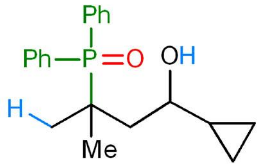

2an

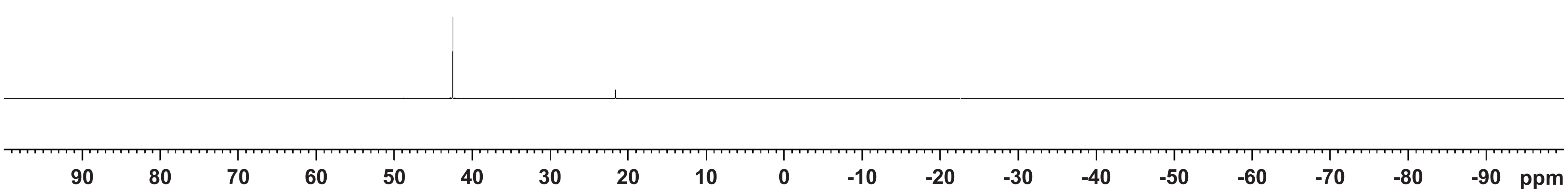

10 Supplementary References

[1] Wei, B.-Y., et al. Electrochemically Tuned Oxidative [4+2] Annulation and Dioxygenation of Olefins with Hydroxamic Acids. *Angew. Chem. Int. Ed.* **60**, 3182–3188 (2021).

[2] Lai, S.-Q., et al. Photocatalytic Anti-Markovnikov Radical Hydro- and Aminooxygenation of Unactivated Alkenes Tuned by Ketoxime Carbonates. *Angew. Chem. Int. Ed.* **60**, 21997–22003 (2021).

[3] Liu, T.-M., et al. Photosensitized diradical rearrangement of alkenyl oxime ethers towards amino-featured oxiranes: reaction, mechanism, and structural prediction. *Chem. Sci.* **16**, 14811–14821 (2025).

[4] Murai, M.; Mizuta, C.; Taniguchi, R.; Takai, K. Synthesis of Borylcyclopropanes by Chromium-Promoted Cyclopropanation of Unactivated Alkenes. *Org. Lett.* **19**, 6104–6107 (2017).

[5] Katherine M. B., et al. Design and synthesis of a crosslinker for studying intracellular steroid trafficking pathways. *Bioorg. Med. Chem.* **23**, 3843–3851 (2015).

[6] Ashley, L. R., et al. Discovery of Small Molecule Interleukin 17A Inhibitors with Novel Binding Mode and Stoichiometry: Optimization of DNA-Encoded Chemical Library Hits to In Vivo Active Compounds. *J. Med. Chem.* **67**, 6456–6494 (2024).
