# Supplementary material for: Catalytic Markovnikov hydrophosphorylation of unactivated olefins via a radical-polar crossover rearrangement
Source: Nat Commun. 2026 May 16;17:6500. doi: 10.1038/s41467-026-72870-2 (PMC13376919; doi:10.1038/s41467-026-72870-2)
Supplement: Supplementary file 2 — Peer Review file [file 41467_2026_72870_MOESM2_ESM.pdf]

nature portfolio

Peer Review File

Catalytic Markovnikov Hydrophosphorylation of Unactivated Olefins via a Radical-Polar Crossover Rearrangement

Corresponding Author: Professor Bing Han

This file contains all reviewer reports in order by version, followed by all author rebuttals in order by version.

Version 0:

Reviewer comments:

Reviewer #1

(Remarks to the Author)
This manuscript presents a Markovnikov-type hydrophosphorylation of unactivated alkenes achieved through a water-mediated radical–polar crossover rearrangement of phosphinites. Mechanistically, we believe this transformation essentially constitutes an intramolecular radical Arbuzov reaction. The system demonstrates broad substrate scope and very good regioselectivity. Moreover, the authors have successfully prepared various deuterated compounds using their established conditions. The successful realization of hydrophosphorylation for unactivated alkenes is impressive and highly commendable. I recommend acceptance after the following revisions:
1. Relevant literature on the radical Arbuzov reaction should be cited.
2. The structure of compound VI in Scheme 5 appears to be incorrectly drawn.
3. For compound 2k, the C–H bond is highlighted in blue and drawn in bold. Does this indicate a cis relationship between the newly formed C–H bond and the phosphorus group? The same depiction is used for 2k-D. It is crucial to determine the relative configuration of 2k-D (e.g., by H NMR analysis), as it could have significant mechanistic implications.
4. The authors state that "the cis-fused 5,6-bicyclic radical intermediates are more easily formed than the trans-fused counterpart." However, the trans product is obtained. If cis-fused intermediates are more accessible, why is the trans product favored?
5. What is the rate-determining step of the reaction? Is the reaction between Co–H and the alkene to generate a carbon radical reversible? The results from deuterophosphonation experiments appear to suggest irreversibility. If so, how is the regioselectivity in 2ad rationalized, given that the formation of secondary carbon radicals at both internal alkene carbons is considered comparable? Could the phosphine group be directing the regioselectivity?
6. The formation of 2r' 18, along with other examples in the Supporting Information (S7–S8), demonstrates that C–O bond cleavage is also feasible, although it may not proceed via a radical pathway. The authors should discuss this alternative pathway.
7. The molecular weight of triphenylphosphine oxide is 278. However, in the Supporting Information, the authors used 277 and 279 for quantification. Which ionization method was employed—ESI or EI?
8. The signal-to-noise ratio in the 13C NMR spectra of compounds 2t and 2ab is unacceptably low.
9. The 13C NMR data require careful analysis. Several errors in coupling patterns and constants are present throughout the Supporting Information. All 13C NMR interpretations must be thoroughly reviewed and corrected. In addition, for the HRMS compound 2o, detected species should be $[M+H]^+$ rather than $[M+Na]^+$ (Calcd for $C_{22}H_{31}NaO_2P^+$: 381.1954; $[M+Na]^+$: 359.1940).

Reviewer #2

(Remarks to the Author)
Hydrophosphorylation of alkenes provides access to a wide range of organophosphorus compounds, however, controlling regioselectivity remains a significant challenge, particularly under radical conditions. While anti-Markovnikov selectivity is well studied in radical hydrophosphorylation, Markovnikov selectivity has not yet been achieved. In this manuscript, Han and co-workers have developed a well-designed triple catalytic system for the first-time realization of radical Markovnikov hydrophosphorylation of unactivated alkenes. Notably, all three atoms (H, H, O) of a water molecule are fully incorporated into the organic product, an uncommon transformation in the context of water utilization in organic synthesis. When D2O was employed, the valuable deuterated products could be obtained in a site-selective manner with a high deuterium

incorporation. The substrate scope is broad, and a variety of functional groups are well tolerated. Gram-scale reactions and late-stage modification of natural product proceed smoothly, demonstrating the practical scalability of this method. The proposed catalytic cycle is supported by mechanistic studies, and the Supporting Information has been carefully prepared. In summary, this manuscript represents a significant advancement in the field of Markovnikov hydrophosphorylation of unactivated olefins. The reviewer supports its publication in Nature Communications, pending satisfactory consideration of the comments provided.
Comments:
1. In Figure 1c, the substrate structure depicted for the "phosphinite rearrangement" is incorrect, as it lacks a methyl group. Secondary not primary alkyl radicals are usually generated through MHAT.
2. In entry 10 of Table 1, the molecular formula of the parent Brønsted acids should be clearly specified to facilitate reader comprehension.
3. Various homoallylic and bishomoallylic alcohol perform effectively in this markovnikov hydrophosphorylation. How about homoallylic and bishomoallylic amine in this transformation?
4. In the substrate study, the results show that hex-5-en-1-ol and hept-6-en-1-ol, which contain longer carbon chains between the olefin and hydroxyl functional groups, yielded only trace amounts of the corresponding products. However, since Co(III)–H is capable of promoting olefin isomerization, could other product isomers be detected in the reactions involving hex-5-en-1-ol and hept-6-en-1-ol?
5. In the mechanistic study, there is currently no direct experimental evidence supporting cyclic P-atom-centered radical intermediate IV or quaternary phosphonium ion intermediate V. The detection of the corresponding intermediates via 31P NMR, HRMS and radical trapping with TEMPO would provide stronger support for the proposed mechanism.
6. The stereochemical outcome of compound 2al should be characterized, particularly the configuration of stereogenic α-carbon adjacent to hydroxyl group. Confirmation of its stereochemistry would provide further insight into the phosphorus migration mechanism, supporting P–O(C) bond cleavage rather than C–O bond cleavage.
7. The 2H NMR spectra and associated analytical data for the deuterated compounds are currently unavailable and should be provided.
8. The selected and general reviews on cobalt catalysis involving cobalt hydride should be cited, see: Acc. Chem. Res. 2021, 54, 2701; Chem. Rev. 2019, 119, 2876-2953.

Reviewer #3

(Remarks to the Author)

In this report, Han and colleagues describe a triple-catalytic Markovnikov hydrophosphorylation of unactivated olefins via a water-mediated radical–polar crossover rearrangement of phosphinites. The design employs a sequential reaction of (bis)homoallylic alcohols, Ph_2PCl, and water, leveraging synergistic photoredox and Co(II)-mediated hydrogen atom transfer (HAT) catalysis. This strategy builds upon recent advances in Co(III)-hydride-mediated radical–polar crossover MHAT processes and their applications in remote functional group migration (see, e.g., Chem. Asian J. 2025, 0, e00448; CCS Chem. 2025, org/10.31635/ccschem.025.202505922, though these are not cited). Water serves as an ideal hydrogen and oxygen source in the oxygenation of trivalent phosphorus, enabling a radical Markovnikov hydrophosphorylation of alkenes through the combination of potent photoreduction and cobalt catalysis. The disclosed methodology operates under mild conditions, exhibits broad functional group tolerance, and delivers good-to-excellent yields, at least for diarylphosphine products.
Despite these noteworthy contributions—including potential applications in olefin deuterophosphorylation using D_2O—the manuscript offers limited educational value. The Markovnikov-selective hydrophosphorylation of olefins is impressive and represents a meaningful advance. However, most examples in this work focus on homoallylic alcohols that readily form five- or six-membered cyclic intermediates, raising concerns about the generality and mechanistic novelty of the catalytic system. This reliance also imposes constraints on the allowable chain length of the olefin substrates.
Overall, the manuscript is clearly written and reflects a solid scholarly investigation. The findings are substantive and will be of interest to the field. That said, the conceptual approach does not convey significant novelty. Therefore, I recommend submission to a more specialized journal, where the important results can be suitably highlighted without overstating the scientific advance for a broad readership such as that of Nature Communications.

Please address the following comments prior to resubmission:

1. The core strategy of this work involves generating carbon radicals via cobalt-hydride-mediated MHAT, followed by intramolecular cyclization to form five- or six-membered cyclic intermediates that facilitate phosphinite group migration, ultimately achieving Markovnikov-selective radical hydrophosphination of alkenes. Given that MHAT-mediated hydrofunctionalization of alkenes—including recent advances in photoredox/cobalt co-catalyzed MHAT and even enantioselective variants—has been extensively explored in modern organic synthesis, it would be valuable to introduce and highlight these related developments in the introduction. Citing key examples of cobalt-catalyzed MHAT processes would help better position the present work within the existing literature and clarify its conceptual advance.
2. The authors describe the transformation as a “water-mediated radical-polar crossover phosphinite rearrangement.” It would be helpful if the authors could elaborate more explicitly on what is meant by “radical-polar crossover” in this specific context. For instance, does the migration of the phosphinite group proceed through a process involving radical addition followed by β-cleavage? Clarifying the sequence of events and the nature of the crossover step would enhance the mechanistic discussion and prevent potential misinterpretation.
3. Page 1, line 29, the author says that “the REDOX activation of water” that means the water undergoes a redox reaction.

So, in the current alkene hydrophosphorylation reaction, how should we understand this process?
4. Page 1, line 39，The author believes that the reasons why the Markovnikov selectivity was not achieved was that it was extremely difficult to install a phosphoryl group on the inner side of olefins. Could the author provide specific challenges or difficulties associated with this reaction?
5. This photoredox/cobalt/pyridinium triple-catalytic protocol employed by the authors is not an original design. This triple-catalytic system was first reported by Ohmiya et al. in their study on the Markovnikov hydroxylation reaction of alkenes (JACS 2022, 144, 7953).
6. Based on compound 2o, the process of radical cyclization followed by cleavage appears to lack stereoselectivity. Why then do compounds 2g-2j not exhibit diastereomers? Theoretically, a nearly 1:1 mixture of isomers should be expected. If the product is obtained as a single isomer, the relative configuration of the C–P bond with respect to the alcohol hydroxyl group should be determined.
7. Scope limitations: the authors mentioned that dialkyl-/dialkoxychlorophosphines represented by diethyl and diethoxy were not good partners in this process. Would the use of a photosensitizer with a higher oxidative potential improve this situation?
8. Regarding Scheme 2: The yield for cyano-substituted diarylchlorophosphine is only 13%. Have other strongly electron-withdrawing substituents been explored?
9. -Table 1, entries 7-9: Could the authors explain the influence of the counteranion in Hpy salts on the reaction? Why do counterions have such a significant impact?
10. Is 2ah-O18 obtained from the mechanistic experiment a single, discrete structure? Theoretically, it should exist as a mixture with a certain diastereomeric ratio (dr). It is recommended that the corresponding dr value be provided.
11. In the supporting information, the NMR spectra of these compounds (2b, 2j, 2k, 2ah, 2i-D) are impure.

Version 1:

Reviewer comments:

Reviewer #1

(Remarks to the Author)
The authors have addressed all of my concerns. This work is now suitable for publication in Nature Communications, and I recommend its acceptance.

Reviewer #2

(Remarks to the Author)
The authors have conducted thorough experiments to address the comments raised. Based on the satisfactory revision, I recommend publishing this manuscript in Nature Communications.
A minor revision is required in the manuscript: the molecular structures labeled as VIII and 2 in Figure 5 are incorrectly depicted.

Reviewer #3

(Remarks to the Author)
The authors have thoroughly addressed all previous comments and suggestions in their revised manuscript. The manuscript is scientifically sound, clearly written, and ready for publication in Nat.Commun.

Open Access This Peer Review File is licensed under a Creative Commons Attribution 4.0 International License, which permits use, sharing, adaptation, distribution and reproduction in any medium or format, as long as you give appropriate credit to the original author(s) and the source, provide a link to the Creative Commons license, and indicate if changes were made.
In cases where reviewers are anonymous, credit should be given to 'Anonymous Referee' and the source.
The images or other third party material in this Peer Review File are included in the article's Creative Commons license, unless indicated otherwise in a credit line to the material. If material is not included in the article's Creative Commons license and your intended use is not permitted by statutory regulation or exceeds the permitted use, you will need to obtain permission directly from the copyright holder.
To view a copy of this license, visit https://creativecommons.org/licenses/by/4.0/

Point-by-Point Response to the Reviewers' Comments

We thank the referees for their highly positive comments on this manuscript, and we do believe that the updated paper will benefit from their suggestions. Attached please find the revised paper in which all questions and comments raised by the referees have been considered. The modification and supplements are highlighted by yellow in this response letter and in the revised manuscript.

Please find below the point-by-point responses to referees' comments.

I. In response to the comments of reviewer 1

General Comment: *This manuscript presents a Markovnikov-type hydrophosphorylation of unactivated alkenes achieved through a water-mediated radical–polar crossover rearrangement of phosphinites. Mechanistically, we believe this transformation essentially constitutes an intramolecular radical Arbuzov reaction. The system demonstrates broad substrate scope and very good regioselectivity. Moreover, the authors have successfully prepared various deuterated compounds using their established conditions. The successful realization of hydrophosphorylation for unactivated alkenes is impressive and highly commendable. I recommend acceptance after the following revisions:*

Response: We thank you for your highly positive comments.

Comment 1: *1. Relevant literature on the radical Arbuzov reaction should be cited.*

Response: Thank you for your comment. Per your request, we have cited some relevant literature on the radical Arbuzov reaction in the revised manuscript as shown below.

"25. Yin, J. et al. Phosphonylation of alkyl radicals. *Chem* **9**, 1945–1954, (2023).

26. Pagire,S. K., Shu, C., Reich, D., Noble, A. & Aggarwal, V. K. Convergent Deboronative and Decarboxylative Phosphonylation Enabled by the Phosphite Radical Trap "BecaP". *J. Am. Chem. Soc.* **145**, 18649-18657 (2023).

27. Wang, J., Zhang, Y., Zhu, L., Xue, X. S. & Li, C. Photoinduced Remote C(sp3) −H Phosphonylation of Amides. *Angew. Chem. Int. Ed.* **63**, e202400856 (2024).

28. Cheng, Y. et al. Photoinduced Decarboxylative Radical Phosphinylation. *Angew. Chem. Int. Ed*. **63**, e202316764 (2024).

29. Shennan, B. D. A., Fukuta, T., Yamane, M., Koyama, T., Mitsunuma, H. & Kanai, M. Catalytic Phosphorylation of Tyrosine via a Radical Arbuzov Reaction. *J. Am. Chem. Soc.* **147**, 6349–6354 (2025)."

Comment 2: *The structure of compound VI in Scheme 5 appears to be incorrectly drawn.*

Response: Thank you for pointing out this error, we have corrected the structure of intermediate **VI** as shown below.

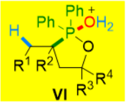

Comment 3: *For compound 2k, the C–H bond is highlighted in blue and drawn in bold. Does this indicate a cis relationship between the newly formed C–H bond and the phosphorus group? The same depiction is used for 2k-D. It is crucial to determine the relative configuration of 2k-D (e.g., by H NMR analysis), as it could have significant mechanistic implications.*

Response: Thank you for your comment. In fact, for compounds **2k** and **2K-D**, the representation of the newly formed C-H/C-D bond (marked in bold blue) is only to highlight the newly formed bond, so as to be consistent with the representation of the newly formed bond of other products, rather than to indicate its relative configuration with the adjacent phosphoryl group.

In addition, for **2k**, since the newly introduced atom is H-atom, it is indistinguishable from the original H-atom on the same carbon. However, based on the well-recognized radical *trans*-addition mechanism on cyclic alkenes, the relative configuration of newly introduced H-atom with the adjacent phosphonyl group in our case should be identified as *trans*-.

According to the well-recognized radical *trans*-addition mechanism, we have assigned the relative configuration between the newly formed C–H/D bond and the phosphorus group to *trans*-, as show below.

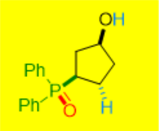

2k, 56%
single isomer

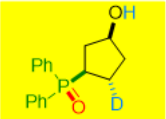

2k-D, 70%, 90%-D[f]
single isomer

For **2k-D**, as the newly introduced D-atom is different from the original H-atom on the same carbon, its relative configuration with the adjacent P-group can theoretically be determined through the H-H coupling constant of 1H NMR. However, since this gem-H-atom is completely overlapped with other H-atoms of cyclopentanol, it cannot be analyzed by this method. But according to the same radical addition reaction mechanism, the relative configuration between the newly introduced D-atom and vicinal phosphoryl should also be *trans*-.

In fact, what is of great significance to the reaction mechanism is not the relative configuration of H-atom or D-atom with the phosphonyl group, but the relative configuration between the phosphonyl group and the hydroxyl group, because it directly points to whether the reaction undergoes the process of C-O bond cleavage for secondary alcohol. Since there is only one stereoisomer was obtained in the reaction, which indicates that this reaction does not involve the cleavage of C-O bonds, but does indeed involve the cleavage of P-O bonds.

Comment 4: *The authors state that "the cis-fused 5,6-bicyclic radical intermediates are more easily formed than the trans-fused counterpart." However, the trans product is obtained. If cis-fused intermediates are more accessible, why is the trans product favored?*

Response: Thank you for pointing out this problem. Obviously, due to a clerical error, the meaning expressed in this

sentence is exactly reversed. In fact, since the *trans*-product is produced, the formation of *trans*-fused 5,6-bicyclic radical intermediates are more favored. We have corrected the description in the main text as bellow.

"the trans-fused 5,6-bicyclic radical intermediates are more easily formed than the cis-fused counterpart."

Comment 5: *What is the rate-determining step of the reaction? Is the reaction between Co–H and the alkene to generate a carbon radical reversible? The results from deuterophosphonation experiments appear to suggest irreversibility. If so, how is the regioselectivity in 2ad rationalized, given that the formation of secondary carbon radicals at both internal alkene carbons is considered comparable? Could the phosphine group be directing the regioselectivity?*

Response: Thank you for your insightful comments. We believe that the MHAT between Co-H and the alkene to generate a carbon radical is the rate-determining step while the subsequent 5-/6-exo carbon radical cyclization onto phosphorous is a fast step, which leads to the former looks like irreversible in our reaction. This view has been supported by the deuterophosphonation experiments as only mono-deuteration products were obtained. As for the reason of the excellent regioselectivity of **2ad**, it should be attributed to the stereo-hindrance effect of the phosphite group rather than its directional effect. The larger the phosphite ester group, the greater the steric hindrance between it and the Co-H complex, and the more difficult it is for the olefin carbon atoms nearby to undergo the MHAT reaction. As a very bulky tertiary alcohol phosphite ester is involved in the case of **2ad**, only one regioisomer was detected. In contrast, when a primary alcohol phosphite ester is involved as in the case of **2ac**, another regioisomer **2ac'** as the minor product was also obtained.

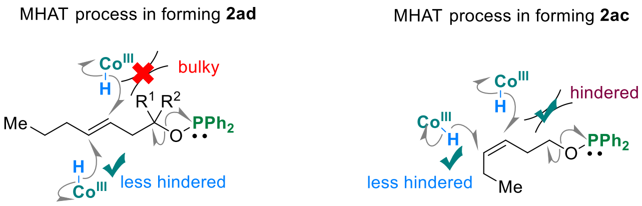

"Apparently, the good regioselectivity can be attributed to the stereo-hindrance effect between the phosphite group and the Co-H complex. The larger the phosphinite group, the greater the steric hindrance between them, and the more difficult it is for the alkene carbon atoms closer to the phosphinite to undergo the MHAT reaction."

Comment 6: *The formation of 2r' 18, along with other examples in the Supporting Information (S7–S8), demonstrates that C–O bond cleavage is also feasible, although it may not proceed via a radical pathway. The authors should discuss this alternative pathway.*

Response: Thank for your comments, we have added the corresponding discussion in the revised manuscript as

shown below.

In the ^{18}O isotope tracing experiments of mechanism study part, we have added a sentence to discuss the C-O bond cleavage for tertiary alcohol as shown below.

"It can be clearly seen that for primary and secondary alcohols, the O-atom of the phosphoryl moiety is almost derived from $H_2{}^{18}O$ rather than the hydroxyl group of alcohols, indicating that the reaction mainly involves the cleavage of the P-O bond, with almost no cleavage of the C-O bond. While for tertiary alcohols, the O-atom of the phosphoryl moiety is derived from both $H_2{}^{18}O$ and the hydroxyl group, which indicates that in addition to the cleavage of the P-O bond, the cleavage of the C-O bond is also feasible due to the formation of a relative stable tertiary carbocation."

In the proposed mechanism part, we have added a sentence to discuss the C-O bond cleavage for tertiary alcohol as shown below.

"Alternatively, **2** could also be generated by the secondary pathway of a C-O bond cleavage of the intermediate **V** according to ^{18}O isotope tracing experiments, especially for tertiary alcohols."

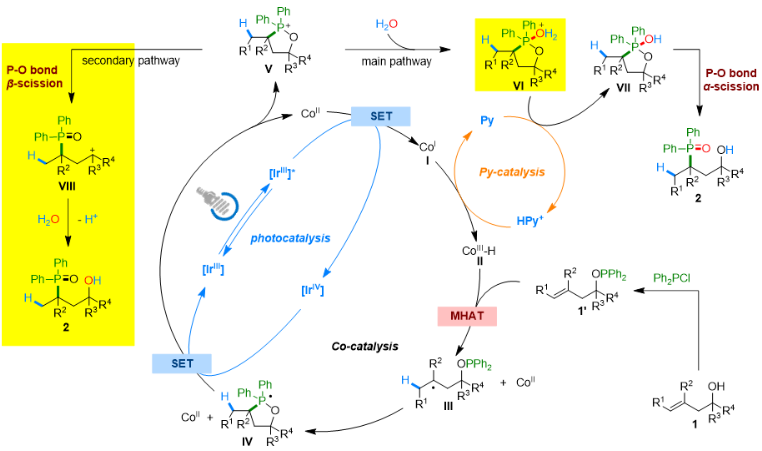

Comment 7: *The molecular weight of triphenylphosphine oxide is 278. However, in the Supporting Information, the authors used 277 and 279 for quantification. Which ionization method was employed—ESI or EI?*

Response: Thank you for your comment. The used ionization method is EI in our case, and the obtained molecular weight of triphenylphosphine oxide is 277 by this method (left), which is exactly matched with that in the standard database (right), as shown below. In addition, the used EI method has also been noted in the revised SI.

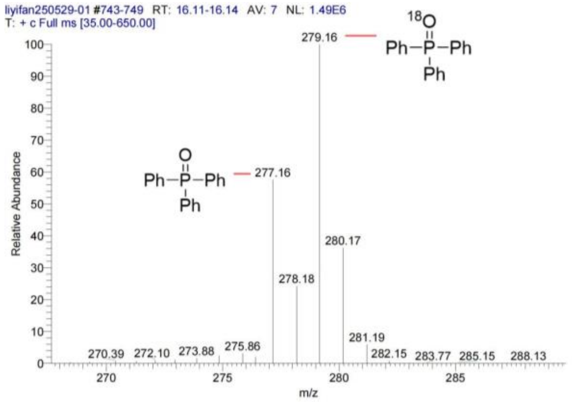

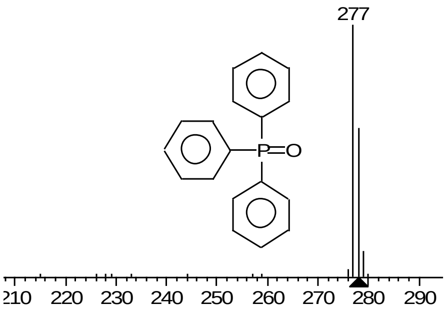

Comment 8: *The signal-to-noise ratio in the 13C NMR spectra of compounds 2t and 2ab is unacceptably low.*

Response: Thank you for your comment, we have rerecorded the 13C NMR of compounds **2t** and **2ab** and the obtained spectra have been updated in the revised SI.

Comment 9: *The 13C NMR data require careful analysis. Several errors in coupling patterns and constants are present throughout the Supporting Information. All 13C NMR interpretations must be thoroughly reviewed and corrected. In addition, for the HRMS compound 2o, detected species should be [M+H]$^+$ rather than [M+Na]$^+$ (Calcd for $C_{22}H_{31}NaO_2P^+$: 381.1954; [M+Na]$^+$: 359.1940).*

Response: Thank you for pointing out these errors. We have corrected the improper coupling patterns and constants of 13C NMR and carefully reviewed all 13C NMR interpretations to ensure that they are all correct. In addition, we have corrected [M+Na]$^+$ to [M+H]$^+$ for compound **2o.**

II. In response to the comments of reviewer 2

General Comment: *Hydrophosphorylation of alkenes provides access to a wide range of organophosphorus compounds, however, controlling regioselectivity remains a significant challenge, particularly under radical conditions. While anti-Markovnikov selectivity is well studied in radical hydrophosphorylation, Markovnikov selectivity has not yet been achieved. In this manuscript, Han and co-workers have developed a well-designed triple catalytic system for the first-time realization of radical Markovnikov hydrophosphorylation of unactivated alkenes. Notably, all three atoms (H, H, O) of a water molecule are fully incorporated into the organic product, an uncommon transformation in the context of water utilization in organic synthesis. When D2O was employed, the valuable deuterated products could be obtained in a site-selective manner with a high deuterium incorporation. The substrate scope is broad, and a variety of functional groups are well tolerated. Gram-scale reactions and late-stage modification of natural product proceed smoothly, demonstrating the practical scalability of this method. The proposed catalytic cycle is supported by mechanistic studies, and the Supporting Information has been carefully prepared. In summary, this manuscript represents a significant advancement in the field of Markovnikov hydrophosphorylation of unactivated olefins. The reviewer supports its publication in Nature Communications, pending satisfactory consideration of the comments provided.*

Response: Thank you for your highly positive comments on our work.

Comment 1: *In Figure 1c, the substrate structure depicted for the "phosphinite rearrangement" is incorrect, as it*

lacks a methyl group. Secondary not primary alkyl radicals are usually generated through MHAT.

Response: Thank you for your comment. In fact, the rearrangement of phosphinites depicted in Figure 1c is a broad radical rearrangement process, applicable to primary, secondary and tertiary carbon radicals, rather than specifically referring to the secondary carbon radical produced after MHAT. However, in accordance with your request, we have changed it to a secondary radical as shown below.

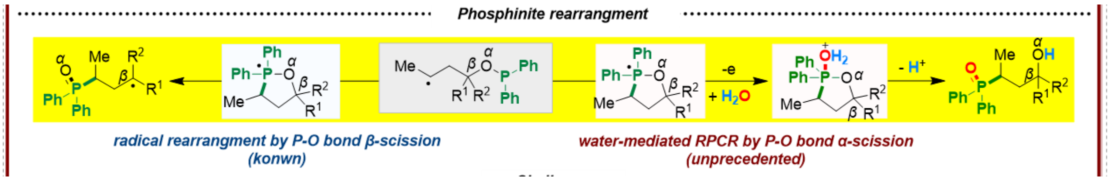

Comment 2: *In entry 10 of Table 1, the molecular formula of the parent Brønsted acids should be clearly specified to facilitate reader comprehension.*

Response: Thank you for your suggestion, we have added a table footnote to describe which Bronsted acids were used as shown below.

10	parent Bronsted acids $HClO_4$ / HOTf / HPF_6 / HCl instead of **HPy**	0[c]

Comment 3: *Various homoallylic and bishomoallylic alcohol perform effectively in this markovnikov hydrophosphorylation. How about homoallylic and bishomoallylic amine in this transformation?*

Response: Thank you for your suggestion. Per your request, we have tested homoallylic and bishomoallylic amines under the standard conditions. However, the reaction only gave a mixture of the mono- and disubstituted N-P condensation intermediates, and they were inert in subsequent triple catalysis and not any reaction occurred. The failure of hydrophosphorylation may be due to the strong basicity of the amine intermediate, which will grab protons from the pyridinium catalyst HPy, eventually causing the latter to lose its catalytic ability.

Comment 4. *In the substrate study, the results show that hex-5-en-1-ol and hept-6-en-1-ol, which contain longer carbon chains between the olefin and hydroxyl functional groups, yielded only trace amounts of the corresponding products. However, since Co(III)–H is capable of promoting olefin isomerization, could other product isomers be detected in the reactions involving hex-5-en-1-ol and hept-6-en-1-ol?*

Response: Thank you for your comment. Actually, we did not observe olefin isomerization when terminal olefin hex-5-en-1-ol and hept-6-en-1-ol were involved in our case. In fact, when hex-5-en-1-ol was involved in the reaction, the unreacted phosphinite intermediate of hex-5-en-1-ol was recovered in about 60% yield under the standard conditions, accompanied by the formation of diphenylphosphine oxide in 30% yield and a mixture of alkane including *n*-hexane/hexene was detected by GC-MS. Similar result was obtained for hept-6-en-1-ol. These results indicate that there is indeed a competitive MHAT reaction between Co-H with olefin and P-atom of phosphinite intermediate, which has been drawn in the Fig. 1 as a challenge of the desired reaction. In addition, if this olefin isomerization can occur in our reaction, hex-5-en-1-ol and hept-6-en-1-ol will isomerize into the appropriately located internal alkenes, which can form favorable five- or six-membered ring intermediates, and ultimately produce the desired products **2d** and **2e** in high yields. However, the desired products were obtained only in trace amounts, indicating that olefin isomerization has indeed not occurred.

***Comment 5.** In the mechanistic study, there is currently no direct experimental evidence supporting cyclic P-atom-centered radical intermediate IV or quaternary phosphonium ion intermediate V. The detection of the corresponding intermediates via 31P NMR, HRMS and radical trapping with TEMPO would provide stronger support for the proposed mechanism.*

Response: Thank you for your suggestion. Per your request, we have conducted some additional experiments to support that the key intermediates IV and V are involved in the reaction.

When TEMPO was added in the sample reaction, the reaction was completely inhibited but the TEMPO-trapped adduct was detected by HRMS as show below. This result confirms that the reaction involves a radical process.

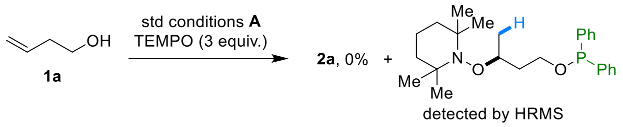

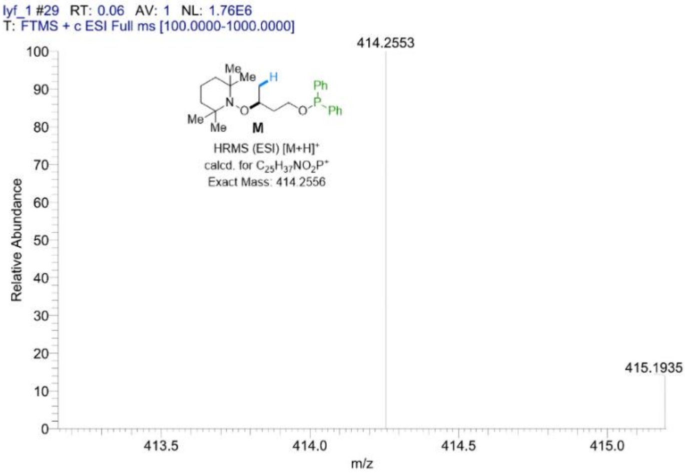

Moreover, when N-tert-Butyl-α-phenylnitrone (PBN) was used as the spin-trapping reagent, the adduct of cyclic P-atom-centered radical intermediate **IV** with PBN was detected by EPR experiment as well as HRMS as shown below.

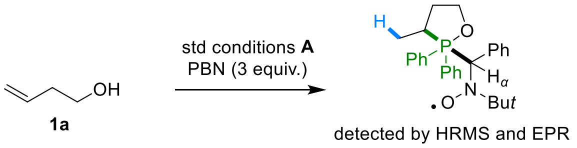

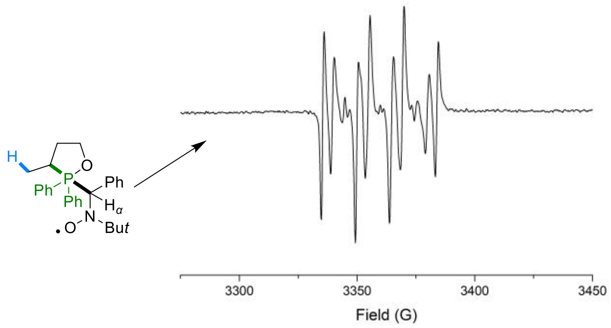

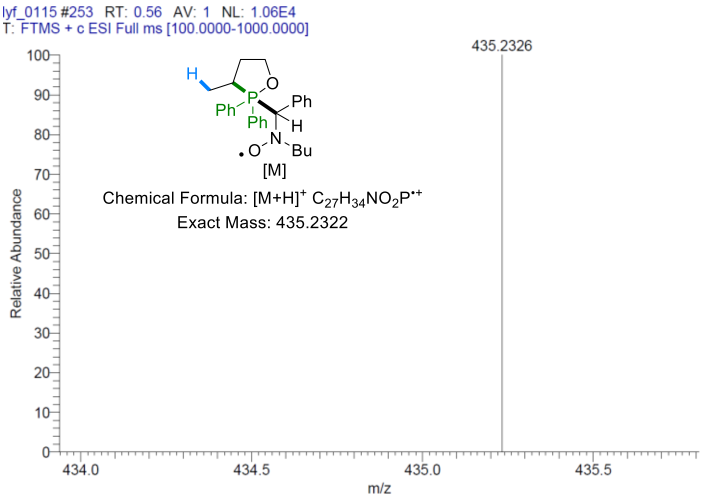

In addition, the quaternary phosphonium ion intermediate **V** can be directly detected via HRMS by using the sample reaction mixture under standard conditions, as show below.

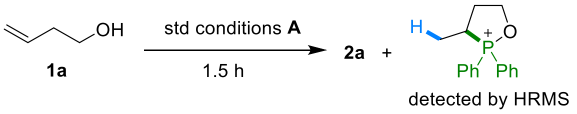

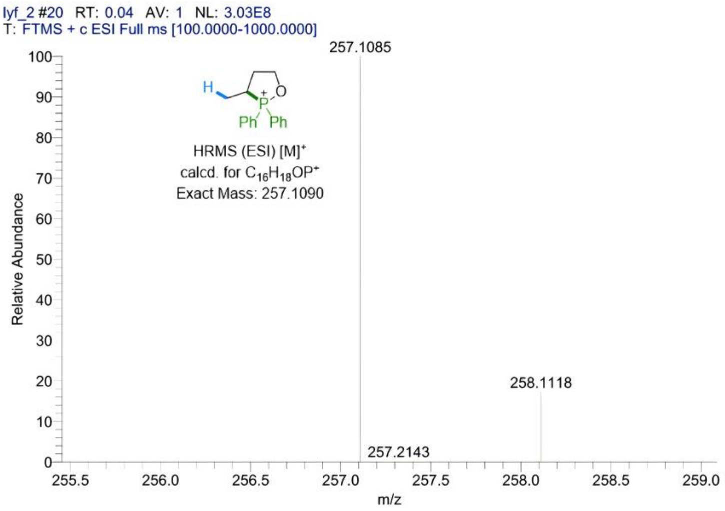

These results have been added in the revised SI.

In addition, we have added these results and the corresponding descriptions in the revised manuscript as shown

below.

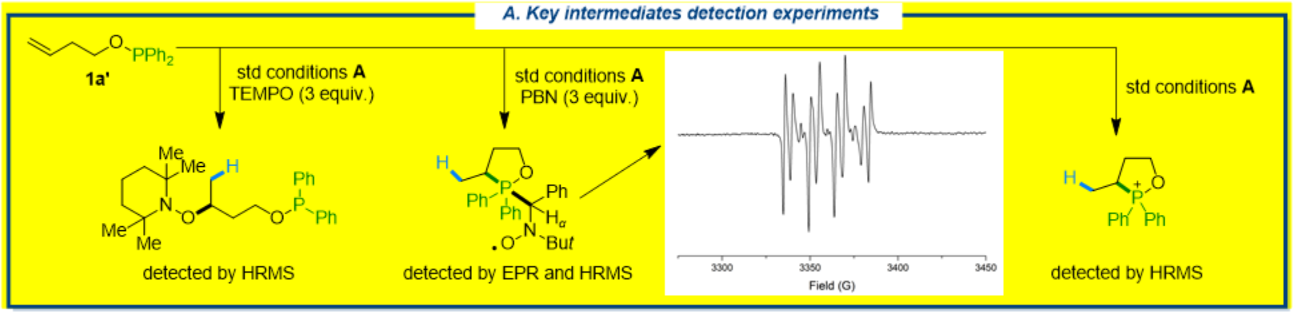

"To elucidate the reaction mechanism more deeply, a series of control experiments were conducted as shown in Fig. 4. When 2,2,6,6-tetramethylpiperidin-1-oxy (TEMPO) was used as the C-atom-centered radical scavenger, the sample reaction was totally inhibited and the TEMPO-trapped alkyl-adduct was detected by high resolution mass spectrometer (HRMS), which indicates that the reaction was involves a radical process (Fig. 4A). When *N*-tert-butyl-*α*-phenylnitrone (PBN), a spin trapping reagent, was involved in the sample reaction, a radical adduct of cyclic P-atom-centered radical intermediate with PBN was detected by electron paramagnetic resonance (EPR) experiment as well as HRMS. In addition, the quaternary phosphonium ion intermediate was detected by HRMS by directly using the sample reaction mixture. These results fully indicate that the reaction involves a radical cyclization of an alkyl radical derived from MHAT onto P-atom."

Comment 6. *The stereochemical outcome of compound 2al should be characterized, particularly the configuration of stereogenic α-carbon adjacent to hydroxyl group. Confirmation of its stereochemistry would provide further insight into the phosphorus migration mechanism, supporting P–O(C) bond cleavage rather than C–O bond cleavage.*

Response: Thank you for your comment. In fact, compound **2al** was obtained as a sole isomer, its stereochemistry had been clearly depicted in Scheme 3 of the original manuscript and was consistent with that of the used substrate isopulegol. Obviously, in the reaction, the only carbon atom that may change the stereocenter is the one connected to the hydroxyl group, which could change its stereocenter through the C-O bond cleavage; however, its configuration is not changed, which has been confirmed by its H-H coupling constant of 1H NMR spectrum.

The same result was also obtained in the case of **2k** as only the cis-isomer was obtained, which is further confirmed by its X-ray single-crystal diffraction analysis. These results fully suggest that in the reaction, the C-O bond of secondary alcohol is not broken. To avoid the misunderstanding, we have added a footnote to describe the characterization of the configuration of **2al** in the revised Fig. 3 as show below.

"[c]Configuration was determined by the coupling constant of ^{1}H NMR."

Comment 7. *The 2H NMR spectra and associated analytical data for the deuterated compounds are currently unavailable and should be provided.*

Response: Thank you for your comment. Per your request, we have conducted the 2H NMR experiments for all deuterated compounds and the obtained spectra have been added in the revised SI with the corresponding analytical data.

Comment 8. *The selected and general reviews on cobalt catalysis involving cobalt hydride should be cited, see: Acc. Chem. Res. 2021, 54, 2701; Chem. Rev. 2019, 119, 2876-2953.*

Response: Thank you for reminding us these relevant and valuable reviews on cobalt catalysis involving cobalt hydride, we have cited them as refs. 33 and 34 in the revised manuscript as shown below.

"33. Ai, W., Zhong, R., Liu, X., Liu, Q. Hydride Transfer Reactions Catalyzed by Cobalt Complexes. *Chem. Rev.* **119**, 4, 2876–2953 (2019).
34. Guo, J., Cheng, Z., Chen, J., Chen, X., Lu, Z. Iron- and Cobalt-Catalyzed Asymmetric Hydrofunctionalization of Alkenes and Alkynes. *Acc. Chem. Res.* **54**, 2701–2716 (2021)."

III. In response to the comments of reviewer 3

General Comment: *In this report, Han and colleagues describe a triple-catalytic Markovnikov hydrophosphorylation of unactivated olefins via a water-mediated radical–polar crossover rearrangement of phosphinites. The design employs a sequential reaction of (bis)homoallylic alcohols, Ph_2PCl, and water, leveraging synergistic photoredox and Co(II)-mediated hydrogen atom transfer (HAT) catalysis. This strategy builds upon recent advances in Co(III)-hydride-mediated radical–polar crossover MHAT processes and their applications in remote functional group migration (see, e.g., Chem. Asian J. 2025, 0, e00448; CCS Chem. 2025, org/10.31635/ccschem.025.202505922, though these are not cited). Water serves as an ideal hydrogen and oxygen source in the oxygenation of trivalent phosphorus, enabling a radical Markovnikov hydrophosphorylation of alkenes through the combination of potent photoreduction and cobalt catalysis. The disclosed methodology operates under mild conditions, exhibits broad functional group tolerance, and delivers good-to-excellent yields, at least for diarylphosphine products.*

Despite these noteworthy contributions—including potential applications in olefin deuterophosphorylation using D_2O—the manuscript offers limited educational value. The Markovnikov-selective hydrophosphorylation of olefins is impressive and represents a meaningful advance. However, most examples in this work focus on homoallylic alcohols that readily form five- or six-membered cyclic intermediates, raising concerns about the generality and mechanistic novelty of the catalytic system. This reliance also imposes constraints on the allowable chain length of the olefin substrates.

Overall, the manuscript is clearly written and reflects a solid scholarly investigation. The findings are substantive and will be of interest to the field. That said, the conceptual approach does not convey significant novelty. Therefore, I recommend submission to a more specialized journal, where the important results can be suitably highlighted without overstating the scientific advance for a broad readership such as that of Nature Communications.

Response: We appreciate the time and effort you have spent on our manuscript. However, we cannot agree with the reasons you raised that our work is not suitable for publication in Nature Communications. The following are our arguments.

First, although our work focuses on (bis)homoallylic alcohols that readily form five- or six-membered cyclic intermediates, it represents the first example for the radical Markovnikov-selective hydrophosphorylation and

deuterophosphorylation of unactivated olefins. The reason for this dependence is that Co-H is competitive with the MHAT reaction of alkenes and the P center. When the radical cyclization rate is slow, a side reaction of the MHAT reaction between Co-H and the P center will occur, resulting in the required reaction being unable to proceed effectively. This is also the challenge and difficulty of achieving this reaction. Therefore, the successful realization of radical Markovnikov-selective hydrophosphorylation and deuterophosphorylation should be indeed regarded as an important breakthrough.

Secondly, although the Co(III)-H-mediated radical polar crossover-MHAT process and its application in the migration of remote functional groups have been developed, these reactions are limited to the carbon-centered functional groups migration such as aryls migration. These migrations have been extensively studied and well-established, including two literatures you mentioned (Chem. Asian J. 2025, 0, e00448; CCS Chem. 2025, org/10.31635/ccschem.025.202505922). In contrast, in our case, the migrated functionality is the P-centered functional group, and its migration itself has received little research, especially an unprecedented new rearrangement mode of phosphite is developed for the first time, that is, the water-mediated radical-polar crossover rearrangement experiences the α-scission of the P-O bond. Thus, the study not only realizes the radical Markovnikov-selective hydrophosphorylation and deuterophosphorylation, but also broadens the boundary of P-atom rearrangement reaction. In addition, these two works you mentioned (Chem. Asian J. 2025, 0, e00448; CCS Chem. 2025, org/10.31635/ccschem.025.202505922) have also cited in the revised manuscript as shown below.

"38. Yamashita, M. et al. Distal Heteroaryl Ipso-Migration of Unactivated Alkenes via Cobalt/Photoredox-Catalyzed Hydrogen/Deuterium Atom Transfer. *Chem. Asian J.* **20**, e00448 (2025).

39. Xiao, M., Li, W., Jiang, D. & Cheng, L. J. Cobalt-Catalyzed Markovnikov Hydroarylcarbonylation of Unactivated Alkenes via Distal Aryl Migration. *CCS Chem.* 1−11 (2025)."

Thirdly, most Co-H-mediated alkene hydrogenation reaction including the works you mentioned rely on the additional excess of silicane, borane, Hantzsch ester etc. as the sacrificial hydrogen source, and still require additional strong oxidants or reductants to maintain the REDOX cycle. However, Co-H-mediated alkene hydrofunctionalization reaction employing water as the ideal hydrogen source is rarely developed. Herein, we ingeniously design and develop a Co-H mediated hydrophosphonylation of olefins strategy, which utilizes water as an ideal hydrogen as well as oxygen source. Thus, our protocol not only achieves the radical hydrophosphonylation of olefins for the first time but also realizes the radical reductive activation and all three-atom splitting utilization of water molecules, which greatly broadens the application scope of Co-H catalysis and should be indeed regarded as a novel mechanism advance.

Thus, on the basis of the abovementioned reasons, we believe that our work is worthy of publication in Nature Communications.

Our above arguments are strongly supported by referees 1 and 2, as they both give highly positive assessments of our work and support its publication in Nature Communications.

Comment 1: *The core strategy of this work involves generating carbon radicals via cobalt-hydride-mediated MHAT, followed by intramolecular cyclization to form five- or six-membered cyclic intermediates that facilitate phosphinite group migration, ultimately achieving Markovnikov-selective radical hydrophosphination of alkenes. Given that*

MHAT-mediated hydrofunctionalization of alkenes—including recent advances in photoredox/cobalt co-catalyzed MHAT and even enantioselective variants—has been extensively explored in modern organic synthesis, it would be valuable to introduce and highlight these related developments in the introduction. Citing key examples of cobalt-catalyzed MHAT processes would help better position the present work within the existing literature and clarify its conceptual advance.

Response: Thank you for your suggestion. Per your request, we have added a paragraph in the introduction part to briefly describe progress on this cobalt-catalyzed MHAT processes as shown below.

"Recently, the Co^{III}-H species-mediated metal-hydride atom transfer (MHAT) reaction provides a powerful tool for the generation of hydrogen-atom and the realization of the Markovnikov hydrofunctionalization reaction of alkenes.[31-48] Although most reactions use additional hydrogen gas, boranes and silanes etc. as the terminal hydrogen sources,[39-43] reactions that utilize protons as hydrogen sources have also received increasing attention.[44-48] Among them, Ohmiya et al. realized a Markovnikov olefin hydroetherization using alcohols as the substrate as well as the hydrogen source under a triple catalysis of photoredox/cobalt/Brønsted acid catalysis.[48] Inspired by this elegant work, we assumed that this catalytic system may be also feasible in the activation of water molecules to realize the olefin hydrophosphonylation reaction.. ……."

Comment 2: *The authors describe the transformation as a "water-mediated radical-polar crossover phosphinite rearrangement." It would be helpful if the authors could elaborate more explicitly on what is meant by "radical-polar crossover" in this specific context. For instance, does the migration of the phosphinite group proceed through a process involving radical addition followed by β-cleavage? Clarifying the sequence of events and the nature of the crossover step would enhance the mechanistic discussion and prevent potential misinterpretation.*

Response: Thank you for your comment. In fact, the water-mediated radical-polar crossover phosphinite rearrangement in this study has been clearly depicted in Fig. 1 and compared with the traditional radical mode of phosphinite rearrangement. In addition, the proposed radical-polar crossover sequence is also clearly drawn in Figure 5. Undoubtedly, the present study indeed involves MHAT radical addition on alkenes as well as the subsequent radical cyclization on P-atom which have been confirmed by the suitability of olefinic alcohols as well as the supplementary EPR experiment (please see our response to the comment 5 raised by referee 2). For you raised the β-cleavage, we had also clearly discussed according to the formation of sole stereoisomer of **2k** and **2al** (please also see our response to the comment 6 raised by referee 2), the radical probe experiment, as well as the ^{18}O tracing experiments in mechanism discussion part, and the discussion is copied as below.

"It can be clearly seen that for primary and secondary alcohols, the O-atom of the phosphoryl moiety is almost derived from $H_2{}^{18}O$ rather than the hydroxyl group of alcohols, indicating that the reaction mainly involves the cleavage of the P-O bond, with almost no cleavage of the C-O bond. While for tertiary alcohols, the O-atom of the phosphoryl moiety is derived from both $H_2{}^{18}O$ and the hydroxyl group, which indicates that in addition to the cleavage of the P-O bond, the cleavage of the C-O bond is also feasible due to the formation of a relative stable tertiary carbocation."

Comment 3: *Page 1, line 29, the author says that "the REDOX activation of water" that means the water undergoes a redox reaction. So, in the current alkene hydrophosphorylation reaction, how should we understand this process?*

Response: Thank you for your comment. Apparently, although protonated pyridinium salt HPy was used as the catalyst in the current hydrophosphorylation reaction, the terminal H-atom source is the proton of water, which is derived from water by photoreduction reduction. Therefore, the current hydrophosphorylation reaction undoubtedly undergoes a reduction process of water.

***Comment 4**: Page 1, line 39，The author believes that the reasons why the Markovnikov selectivity was not achieved was that it was extremely difficult to install a phosphoryl group on the inner side of olefins. Could the author provide specific challenges or difficulties associated with this reaction?*

Response: Thank you for your comment. Per your request, we have added a sentence to describe the challenge the of the to install a phosphoryl group on the inner side of olefins as shown below.

"One important reason is that it is still not easy to install a phosphoryl group onto the inner side of olefins by a radical Arbuzov reaction,[25-29] due to the competitive addition of the attacking functionality radical onto the alkene and the phosphite, as well as the inert addition ability of the formed secondary/tertiary carbon radical onto the phosphite."

***Comment 5**: This photoredox/cobalt/pyridinium triple-catalytic protocol employed by the authors is not an original design. This triple-catalytic system was first reported by Ohmiya et al. in their study on the Markovnikov hydroxylation reaction of alkenes (JACS 2022, 144, 7953).*

Response: Thank you for your comment. In fact, we did not claim that such a triple catalysis system is our original design in our manuscript, but it is a fact that we first applied it to the reduction and three-atom splitting and utilization of water. In addition, one elegant work on Co-catalysis by Ohmiya et al. had been cited in our original manuscript, but well still thank you for reminding this work which has also been cited as ref. 48 as shown below.

"48. Nakagawa, M., Matsuki, Y., Nagao K., Ohmiya, H. A Triple Photoredox/Cobalt/Brønsted Acid Catalysis Enabling Markovnikov Hydroalkoxylation of Unactivated Alkenes, *J. Am. Chem. Soc.* **144**, 7953–7959 (2022)"

***Comment 6**: Based on compound 2o, the process of radical cyclization followed by cleavage appears to lack stereoselectivity. Why then do compounds 2g-2j not exhibit diastereomers? Theoretically, a nearly 1:1 mixture of isomers should be expected. If the product is obtained as a single isomer, the relative configuration of the C–P bond with respect to the alcohol hydroxyl group should be determined.*

Response: Thank you for your question. In fact, **2g-2j** possesses only one chiral center, that is the carbon with hydroxyl group, and the carbon connected with the phosphate moiety is not a chiral center as it tethers two same methyl groups. Thus, compounds **2g-2j** are simply impossible to produce diastereomers.

***Comment** 7: Scope limitations: the authors mentioned that dialkyl-/dialkoxychlorophosphines represented by diethyl and diethoxy were not good partners in this process. Would the use of a photosensitizer with a higher oxidative potential improve this situation?*

Response: Thank you for your comment and suggestion. According to your suggestion, we have tested some photocatalysts with higher oxidative potentials such as 4CzIPN and [Ir(ppy)$_2$(dtbbpy)]PF_6 in the reaction involving dialkyl-/dialkoxychlorophosphines. Unfortunately, the reaction only gave a complicated mixture and still yielded trace amounts of the desired products **2aj** and **2ak**, indicating that the higher oxidative potential of photocatalyst may result in other undesired side reactions probably due to overoxidation.

***Comment 8**: Regarding Scheme 2: The yield for cyano-substituted diarylchlorophosphine is only 13%. Have other strongly electron-withdrawing substituents been explored?*

Response: Thank you for your comment and suggestion. Per your request, we also consider other Ph_2PCl with strongly electron-withdrawing substituents such as -SO_2Me and -CO_2Me, attached at the para position of the benzene ring. Unfortunately, such compounds are not commercially available. Despite much effort, the synthesis of such substrates still failed due to their high activity and instability, resulting in the inability to complete subsequent tests. However, we have successfully improved the yield of cyano-substituted diarylchlorophosphine to 40% yield by increasing the usage amount of catalyst **HPy-1** from 20 mmol% to 40 mmol%. The obvious yield improvement of the product indicates that although the electron-withdrawing group has an impact on the reaction, the substrates with it can also be achieved in a moderate yield by slightly adjusting the reaction conditions.

We have added a foot note for this case and updated the yield of the corresponding product and in the revised manuscript.

***Comment 9**: Table 1, entries 7-9: Could the authors explain the influence of the counteranion in Hpy salts on the reaction? Why do counterions have such a significant impact?*

Response: Thank you for your comment. Although there are differences in product yields when using pyridine salts with different counterions (75% yield for ClO_4^-, whereas 48% for OTf^-, 52% for PF_6^-, and 45% for Cl^-), such differences are not so significant. For the yield differences caused by counterions, we believe that counterions regulate the stability and lifetime of cations formed in the reaction by forming ion pairs, such as the oxidation photocatalyst Ir^{Iv}(PPy)$_3$ and cyclic quaternary phosphonium ions, especially the latter. Among them, the ClO^- ion probably show a better stabilization effect and provides better yields of the products.

***Comment 10**: Is 2h-^{18}O obtained from the mechanistic experiment a single, discrete structure? Theoretically, it should exist as a mixture with a certain diastereomeric ratio (dr). It is recommended that the corresponding dr value be provided.*

Response: Thank you for your question, which is the same as the question raised in your comment 6. In fact, 2h-^{18}O possesses only one chiral center, that is the carbon with hydroxyl group, and the carbon connected with the phosphate moiety is not a chiral center as it tethers two methyl groups. Thus, 2h-^{18}O is simply impossible to produce diastereomers.

***Comment 11**: In the supporting information, the NMR spectra of these compounds (2b, 2j, 2k, 2ah, 2i-D) are impure.*

Response: Thank you for your comment. Per your request, we have repurified these compounds and updated their NMR spectra in the revised SI.
